# Theory-guided development of homogeneous catalysts for the reduction of CO_2_ to formate, formaldehyde, and methanol derivatives†

**DOI:** 10.1039/d2sc06793e

**Published:** 2023-02-09

**Authors:** Hanna H. Cramer, Shubhajit Das, Matthew D. Wodrich, Clémence Corminboeuf, Christophe Werlé, Walter Leitner

**Affiliations:** a Max Planck Institute for Chemical Energy Conversion Stiftstr. 34–36, 45470 Mülheim an der Ruhr Germany christophe.werle@cec.mpg.de walter.leitner@cec.mpg.de; b Laboratory for Computational Molecular Design Institute of Chemical Sciences and Engineering, École Polytechnique Fédérale de Lausanne (EPFL) 1015 Lausanne Switzerland clemence.corminboeuf@epfl.ch; c National Centre for Competence in Research – Catalysis (NCCR-Catalysis), École Polytechnique Fédérale de Lausanne (EPFL) 1015 Lausanne Switzerland; d National Centre for Computational Design and Discovery of Novel Materials (MARVEL), Ecole Polytechnique Fédérale de Lausanne (EPFL) 1015 Lausanne Switzerland; e Ruhr University Bochum, Universitätsstr. 150 44801 Bochum Germany; f Institut für Technische und Makromolekulare Chemie (ITMC), RWTH Aachen University Worringer Weg 2 52074 Aachen Germany

## Abstract

The stepwise catalytic reduction of carbon dioxide (CO_2_) to formic acid, formaldehyde, and methanol opens non-fossil pathways to important platform chemicals. The present article aims at identifying molecular control parameters to steer the selectivity to the three distinct reduction levels using organometallic catalysts of earth-abundant first-row metals. A linear scaling relationship was developed to map the intrinsic reactivity of 3d transition metal pincer complexes to their activity and selectivity in CO_2_ hydrosilylation. The hydride affinity of the catalysts was used as a descriptor to predict activity/selectivity trends in a composite volcano picture, and the outstanding properties of cobalt complexes bearing bis(phosphino)triazine PNP-type pincer ligands to reach the three reduction levels selectively under different reaction conditions could thus be rationalized. The implications of the composite volcano picture were successfully experimentally validated with selected catalysts, and the challenging intermediate level of formaldehyde could be accessed in over 80% yield with the cobalt complex 6. The results underpin the potential of tandem computational-experimental approaches to propel catalyst design for CO_2_-based chemical transformations.

## Abstract 2

Computational volcano plots are used to predict selectivity in the context of (first-row) transition metal-catalyzed CO_2_ reduction. The expected trends were tested experimentally and allowed for systematic improvement of the catalyst.

## Graphical abstract

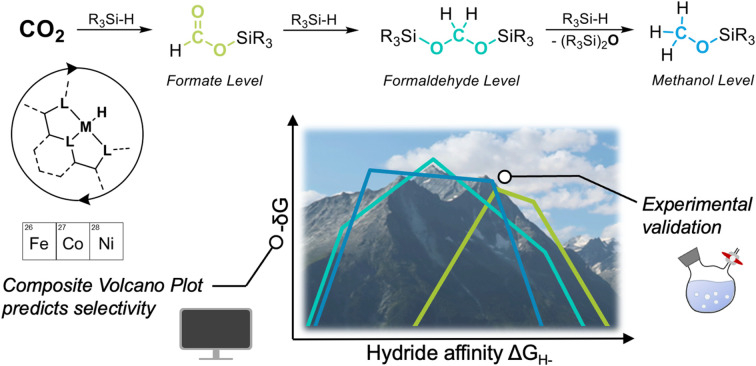

## Introduction

**Fig. 1:**
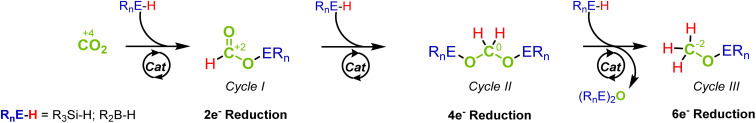
Stepwise reduction of CO_2_ to the formic acid, formaldehyde, and methanol level *via* hydrosilylation or hydroboration.

The catalytic reduction of carbon dioxide (CO_2_) is key when targeting sustainable chemical industries based on renewable energy and non-fossil carbon feedstocks.^1^ The stepwise two-electron reduction of CO_2_ (oxidation state of C +4) provides access to products on the formal reduction level of formic acid (HCOOH, +2), formaldehyde- (H_2_CO, ±0) and methanol (CH_3_OH, −2) as the respective C1 products (Fig. 1).^2^ “Green hydrogen” is essential as a reducing agent to achieve the goal of low or even net-zero greenhouse gas emissions in large-scale industrial applications.^1*c*^ Organometallic complexes have emerged as excellent catalysts for such transformations whereby the activation and transfer of hydrogen typically involve heterolytic cleavage of the H_2_ molecule. In order to obtain fundamental insight into the control factors to reach the various reduction levels, hydrosilanes and boranes offer suitable model systems due to the lower bond dissociation energy and the pre-polarization of the Si–H and B–H bonds as compared to the H–H bond.^3^ Since strong Si–O and B–O bonds provide additional thermodynamic driving force, hydrosilanes and hydroboranes often reduce CO_2_ already at ambient pressure and temperature and make the challenging reduction of CO_2_ beyond the formate level significantly more facile.

The reduction sequence can be described as a three-step cascade reaction (Fig. 1) that involves the first two-electron reduction to formate derivatives (cycle I), followed by the second and third reduction to acetals (cycle II) and methoxides (cycle III). While many catalytic systems facilitate the reduction to the formate level, those that overcome the kinetic barriers of further reduction to the formaldehyde and methanol levels are scarcer.^4^ Furthermore, the overreduction of formaldehyde to methanol derivatives is difficult to suppress, and obtaining high selectivity for this important product level remains particularly challenging.^5^

**Fig. 2:**
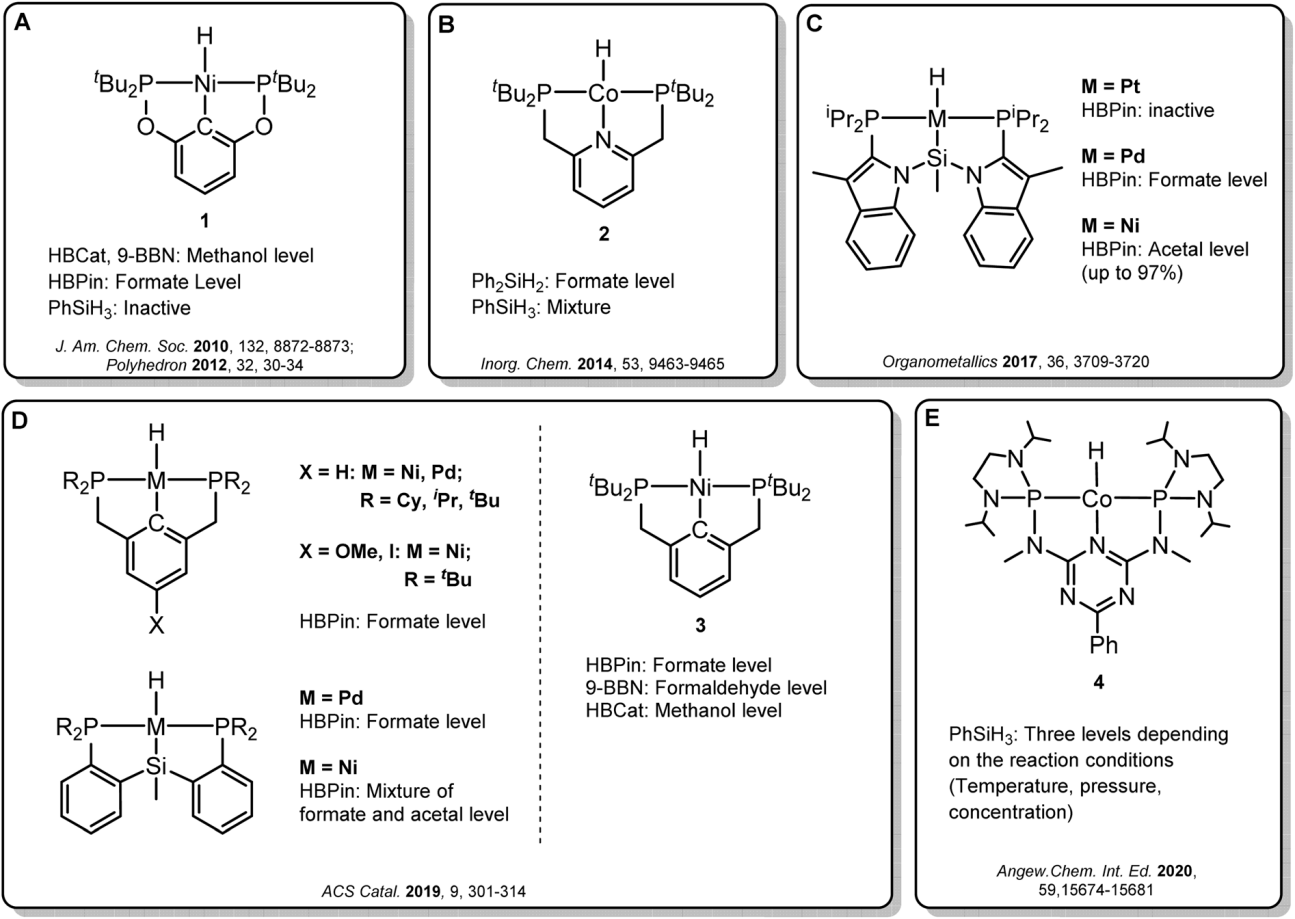
Selected examples of pincer complexes for the catalytic hydroboration and hydrosilylation of CO_2_.

Transition metal pincer complexes have emerged as promising candidates to overcome these obstacles since they offer many possibilities for precise steric and electronic tuning to fulfill the requirements of the desired catalytic pathway (Fig. 2).^6^ Early examples are the nickel and cobalt pincer complexes 1 and 2, for which the product distribution was primarily determined by the chosen reductant (Fig. 2A and B). Turculet and co-workers studied a series of group 10 PSiP pincer hydride complexes in the hydroboration of CO_2_ with HBPin (Fig. 2C).^4*j*^ While the platinum complexes were largely inactive, palladium analogs were found to generate mainly the formate derivatives, and nickel derivatives lead to the formaldehyde level. A variety of PCP and PSiP pincer hydride complexes catalyzed the hydroboration of CO_2_ with HBPin mainly to the formate level, as reported by Hazari and co-workers (Fig. 2D, left).^4*q*^ They showed that (^*t*Bu^PCP)NiH 3 (Fig. 2D, right) even selectively facilitated the two-, four- or six-electron reduction of CO_2_, whereby the choice of the reducing agent largely determined the preferred product level. Our group reported a cobalt PNP triazine pincer complex 4 that selectively accessed the formate-, formaldehyde-, or methanol reduction level with the same reducing agent, depending on the appropriate adjustment of the reaction conditions (Fig. 2E).^4*r*,*t*^

While these studies provide valuable mechanistic insight for a few individual catalytic systems, general conclusions about the relationship between catalyst design, activity, and product distribution remain limited. The systematic evaluation of molecular control factors that influence product distribution is of high interest, since it can reveal how the catalyst architecture dictates the preferred reduction level. These insights are very valuable for developing efficient catalysts that (a) specifically target one particular product or (b) allow access to multiple product levels in an adaptive manner. The present study, therefore, goes beyond mechanistic studies on individual catalysts and aims to unravel general structure–reactivity relationships. To achieve this, it combines computational screening using a volcano-type scaling relationship with experimental validation for selected catalysts to contribute to this fundamental challenge in catalyst design for CO_2_ conversion.

## Results and discussion

**Fig. 3:**
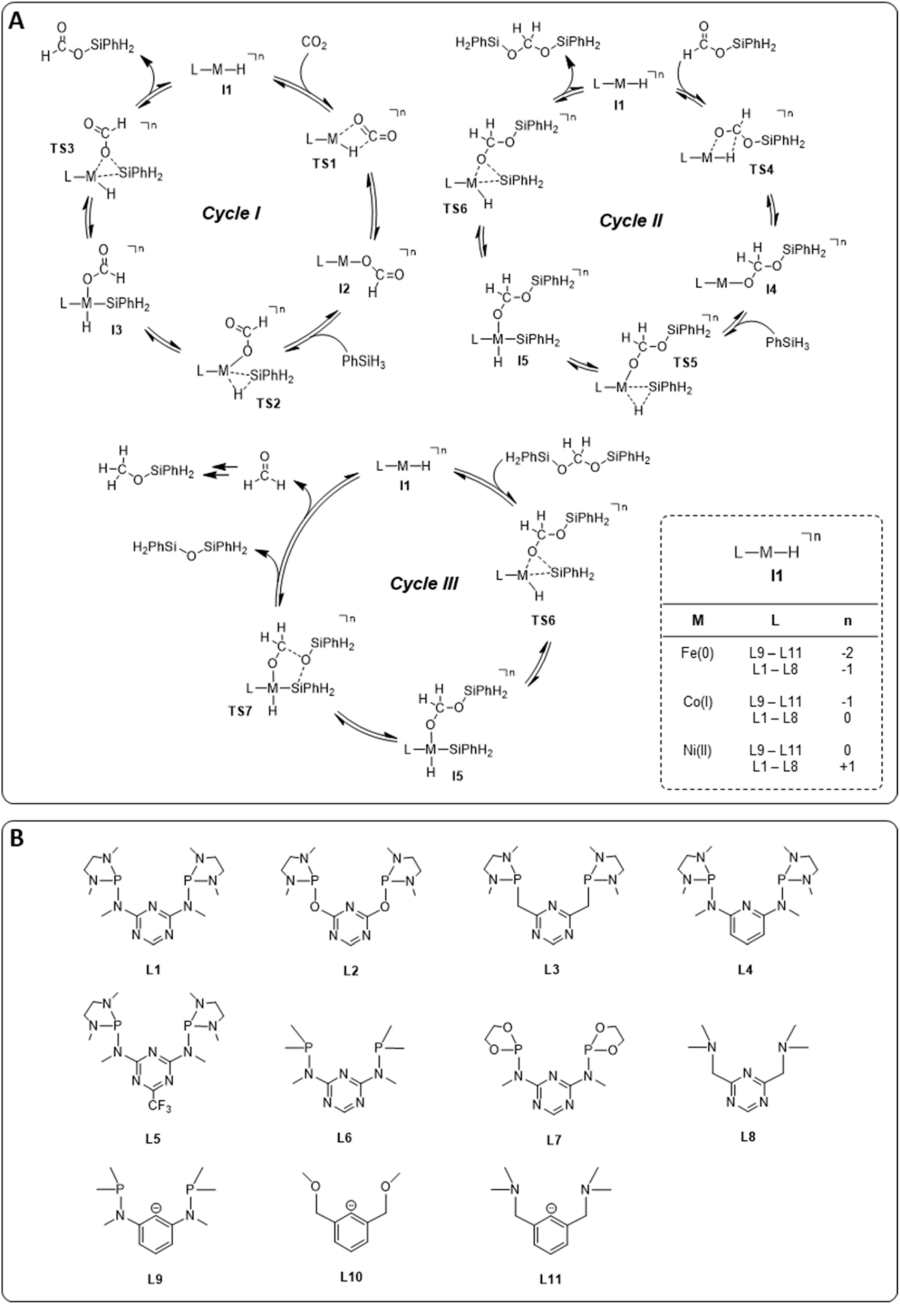
Catalytic cycles for hydrosilylation of CO_2_ to silyl formate, silyl formate to bis(silyl)acetal, and bis(silyl)acetal to formaldehyde (A) library of pincer ligands (B). The total charge *n* arises from the combination of M and L. Counter anions were not considered.

The hydrosilylation of CO_2_ with phenylsilane (PhSiH_3_) was chosen as a prototypical reduction in the present study because it is experimentally verified to reach all three reduction levels. While more than one Si–H bond can participate in the reaction under experimental conditions, only the reaction of a single Si–H bond is considered in the computational analysis for consistency. The mechanisms for the three individual catalytic cycles were based on the pertinent literature involving a repeated sequence of elementary steps for the activation of Si–H bonds and the transfer of formal hydrides to carbon and protons to oxygen.^4*e*,*l*,*o*,3*b*^ The detailed outline of the three cycles as the starting point was adopted from our previous study on the operating mode of the cobalt triazine pincer hydride complex 4 (Fig. 3A).^4*t*^ Cycles I and II feature a hydride transfer step *via*TS1 and TS4, followed by oxidative addition of phenylsilane (TS2 and TS5) and reductive elimination (TS3 and TS6) of the product. The third cycle involves a rearrangement step (TS7) from the intermediate I5 present in cycles II and III, leading to the formation of diphenylsiloxane and formaldehyde. The previous reports all conclude that the reduction of formaldehyde to methanol is rapid and not rate-determining. Specifically, the calculated reaction profile of 4 indicates a slow bis(silyl)acetal to formaldehyde rearrangement step, while the subsequent hydrosilylation of formaldehyde is facile.^4*t*^ Therefore, we omit this latter step from our analysis. In all three cycles, the Si–H activation occurs *via* oxidative addition and the hydride transfer *via* migratory insertion, while product formation involves reductive elimination.

Generally, the relative ratios of the energy spans of cycles I–III determine the observed product distribution in addition to external parameters such as temperature. These energy span ratios will differ significantly for different metals and ligand frameworks, however. Screening potential catalyst candidates would therefore require calculating the free energy profile for all catalyst candidates individually at a high expense of time and computational resources. These computational efforts can be significantly reduced by estimating the free energies of the intermediates and transition states with molecular volcano plots, which relate the catalytic activity to a unique descriptor for each catalyst candidate.^7^ Calculating the descriptor estimates the entire free energy profile by linear free energy scaling relationships (LFESRs) used to construct the volcano plot. This way, the descriptor value of an individual catalyst allows for a rapid assessment of the relative activity by its position on the plot. Expanding this analysis to multiple catalytic cycles and comparing the corresponding activities for an individual catalyst provides insight into its expected selectivity.

**Fig. 4:**
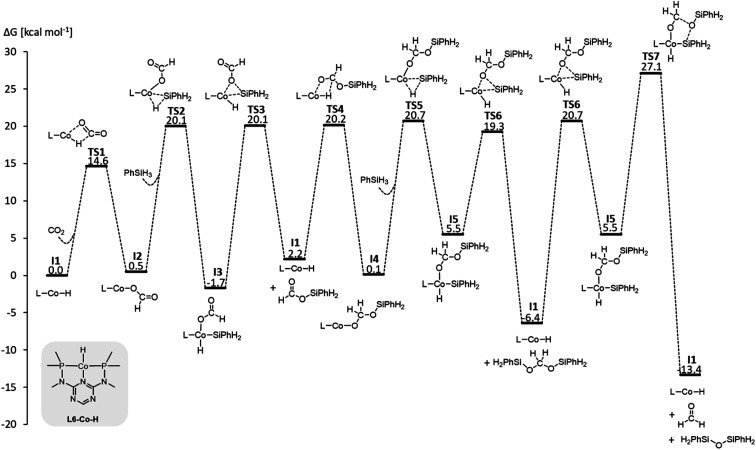
Gibbs free energy profile of L6-Co-H (SMD_(benzene)_/M06/def2-TZVP//M06/def2-SVPD; computational details are available in the ESI†).

We examined a series of isoelectronic d^8^ pincer complexes with Fe(0), Co(i), and Ni(ii) metal centers selected due to their natural abundance and low costs (computational details can be found in the ESI†).^8^ The combination of eleven pincer ligands L1–L11 (see Fig. 3B) and three metals furnishes 33 complexes with the general formula [L–M–H]*^n^* as I1. The charge *n* of the complexes results from the combination of neutral or anionic ligands and the formal oxidation state of the metal center. The ligand library includes PNP (L1–L7), NNN (L8), PCP (L9), OCO (L10), and NCN pincer ligands (L11), featuring various substitution patterns which provide catalysts with diverse steric and electronic environments around the metal center. Fig. 4 depicts the Gibbs free energy profile for a representative pincer catalyst, L6-Co-H. The respective oxidative addition products, I3 and I5, undergo an increase of oxidation state by +2 upon their formation.

The hydride affinity (Δ*G*_H-_) was chosen as a descriptor to represent the intrinsic reactivity of the complexes to map out the activity of the pincer complexes towards the individual cycles and hence also selectivity. The correlation of Δ*G*_H-_ with the catalytic performance of transition metal hydrides, including hydride transfer and hydrogen splitting barriers in the hydrogenation of CO_2_ to formate, has been demonstrated in previous studies.^9^ This work establishes an expanded theoretical framework that features the hydride affinity as a descriptor for multiple product levels beyond formate, while it provides quantitative information not only on the activity but also on the selectivity. Δ*G*_H-_ can be easily estimated from the free energy change of the hydride exchange equilibrium concerning the trityl cation as the reference hydride acceptor (eqn (1)). According to the sign convention used here, a more negative Δ*G*_H-_ value indicates a weaker metal–hydride bond.I1 + [Ph_3_C]^+^ ⇌ [I1−H]^+^ + Ph_3_CH

1 Δ*G*_H−_ = (Δ*G*_[I1−H]^+^_ + Δ*G*_Ph_3_CH_) − (Δ*G*_I1_ + Δ*G*_[Ph_3_C]^+^_)

**Fig. 5:**
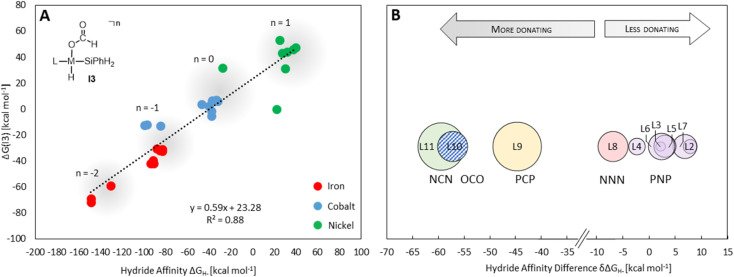
Correlation of the Δ*G*(I3) with the hydride affinity as a descriptor (A) average hydride affinity differences of each pincer ligand relative to L1 as reference (set to zero (B)). The average over the three metals was obtained by subtracting Δ*G*_H-_ (L1–M–H) from Δ*G*_H-_ of the ligand for each metal. The size of the circles reflects the difference in the magnitude of the shifts for each metal.

Providing a measure of the metal–hydride interaction strength, Δ*G*_H-_ is connected with the relative Gibbs free energies for the intermediates and transition states of all three cycles by LFESRs (see Fig. S1†), as exemplified for I3 in Fig. 5A. Note that it is a solely catalyst-based property and, thus, independent of a specific chemical transformation which not only streamlines the screening process but also showcases the transferability of our approach. For the present case, Δ*G*_H-_ can also act as a descriptor in the reduction of formaldehyde and methanol derivatives, including elementary steps that do not directly involve a hydride, such as the reductive eliminations involving TS3, TS6, or TS7. As shown in Fig. 5A, the descriptor Δ*G*_H-_ gradually decreases for catalysts with a more negative charge, as expected from a weaker metal hydride bond. Accordingly, anionic NCN, OCO, and PCP ligands exhibit Δ*G*_H-_ significantly more negative relative to the ligand L1 for each metal (Fig. 5B). While the differences are less pronounced for ligands with the same charge, the influence of the electronic properties of the coordinating atoms is apparent. For example, replacing triazine (L1) with the stronger donor pyridine (L4) leads to a more negative Δ*G*_H-_ in agreement with a higher electron density at the metal and a weakened M–H bond. Replacing phosphine (L3) with nitrogen arms (L8) leads to a substantial decrease of Δ*G*_H-_, reflecting the higher basicity of amines compared to phosphines. In contrast, electronegative oxygen atoms, as in L2 and L7, or *para*-CF_3_ substitution, as in L5, induce a higher Δ*G*_H-_.

**Fig. 6:**
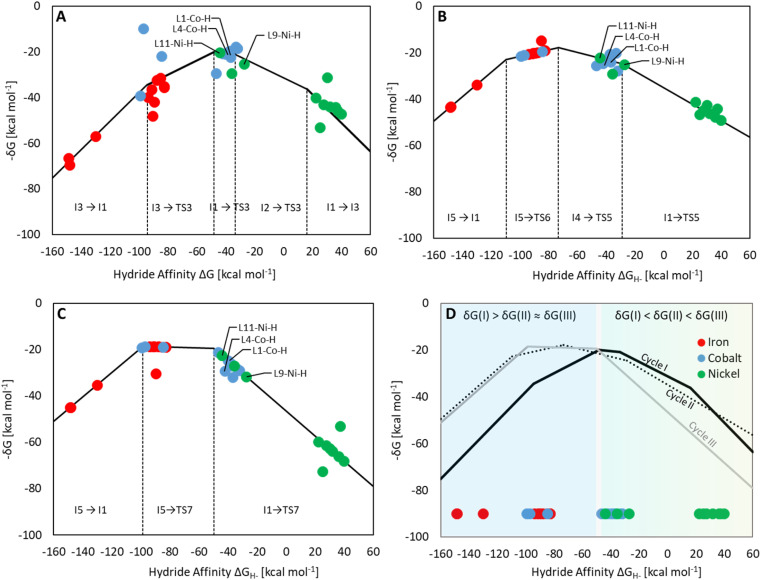
Volcano plots for the reaction of CO_2_ to silyl formate (A), silyl formate to bis(silyl)acetal (B) and bis(silyl)acetal to formaldehyde (C) composite volcano with superposition of three volcano plots (D). The position on the volcano plot is highlighted for selected examples discussed in the text.

Three individual volcano plots (one for each cycle) are derived from the LFESRs (Fig. 6) to analyze the general trends in hydrosilylation activity and selectivity. The step-by-step procedure for constructing molecular volcano plots can be found in a previously published extensive and general protocol.^10^ While LFESRs and volcano plots were recently employed in homogeneous catalysis by some of us,^7*j*,*k*,11^ Norskov and co-workers have previously applied them in the fields of solid-state heterogeneous catalysis and electrocatalysis.^12^ Here, Δ*G*_H-_ is plotted along the *X*-axes of these volcanoes while the *Y*-axes correspond to the negative of the respective catalytic cycle's energy span (δ*G*), as defined by eqn (2).^13^ δ*G* is calculated from the relative energies *T*_TDTS_ and *I*_TDI_ of the turnover-determining transition state (TDTS) and of the turnover-determining intermediate (TDI), respectively, which are the pair of transition state and intermediate that constitutes the largest barrier in the catalytic cycle. The total Gibbs free energy Δ*G*_R_ is added if the TDI appears after the TDTS in the catalytic cycle. Consequently, the catalysts having a lower energy span are more active than the others and are positioned higher in the volcano plots of Fig. 6.2
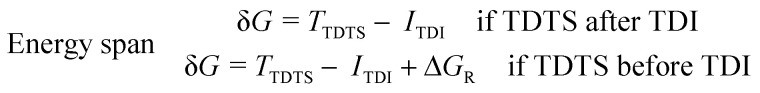


Each volcano plot in Fig. 6 can be divided into different regions depending on the combinations of TDI/TDTS that determine the overall barrier under the framework of the energy span model in the cycle. The oxidative addition products I3 and I5 on the left side of the volcano (more negative Δ*G*_H-_) are too strongly stabilized and thus control the activity together with the high-lying transition states TS3 and TS6. These features align with the expected behavior for catalysts with a high electron density at the metal center since the stabilization of the oxidative addition products renders the forward step of reductive elimination energetically unfavorable. In contrast, complexes with a low electron density at the metal center (more positive or positive Δ*G*_H-_ values) have stronger metal–hydride bonds, and thus, I1 dominates the right sides of the volcano plots. The volcano tops for cycles I (Fig. 6A) and II (Fig. 6B) exhibit peaks at Δ*G*_H-_ = −48 kcal mol^−1^ and –73 kcal mol^−1^, respectively. This suggests that electron-rich complexes are more active in catalyzing the second reduction step in comparison to the first one. Cycle III (Fig. 6C) features a plateau (Δ*G*_H-_ = −99 kcal mol^−1^ to −50 kcal mol^−1^) that is governed by I5 and TS7 involved in the formation of formaldehyde and diphenylsiloxane. This reaction step is associated with bond-forming and breaking events that do not occur in direct proximity to the metal center, and therefore it is less affected by the electronic properties of the metal.

Since the overall product yield of all three C1 products is mainly determined by the activity of the first reduction step of CO_2_ to formate, candidates that appear close to the volcano peak of the cycle I would be expected to form the most productive catalysts. This area includes the neutral complexes of cobalt and nickel L1-Co-H, L4-Co-H, and L11-Ni-H. The complexes 2, 3, and 4 that were previously reported as active CO_2_ hydrosilylation and hydroboration catalysts (Fig. 2) structurally resemble these complexes in accordance with the predictions from the volcano plots. Notably, the catalyst L9-Ni-H appears at a larger distance to the volcano top and is thus expected to be less active. This aligns with the observation that the related complex 1 was reported inactive in the hydrosilylation of CO_2_ with phenylsilane at room temperature.^4*d*^

To obtain insights into the selectivity of pincer catalysts in CO_2_ hydrosilylation, we superimposed the three graphs to produce a single composite volcano plot (Fig. 6D). The non-overlaying positions of the three volcano tops emphasize that the ideal hydride affinity value is distinct for each product level. Based on the relative magnitude of the energy spans of the three cycles δ*G*_I_, δ*G*_II_, and δ*G*_III_, the composite volcano can be divided into two distinct regions. In the green region (*i.e.*, –45 kcal mol^−1^ < Δ*G*_H-_ < 33 kcal mol^−1^), the energy span of the cycles gradually increases in the order I < II < III, suggesting that stepwise access to the three product levels should be feasible by control of external parameters. The sequential ascending energy barriers in the three cycles imply that the catalysts located in this region will be responsive to changes in reaction temperature: At elevated temperature, the selectivity could gradually shift from the formate to the formaldehyde and the methanol level. As the hydride affinity goes down, the decrease in δ*G*_II_ and δ*G*_III_ is steeper (indicated by the higher slope of the volcano lines) compared to δ*G*_I_, gradually favoring the formation of bis(silyl)acetal and formaldehyde. Below Δ*G*_H-_ < −52 kcal mol^−1^ (blue region), the relative span ordering changes to I > II ≈ III, suggesting that formate production is more challenging than the subsequent formation of further reduced products. Thus, any generated silyl formate is expected to be rapidly further reduced to bis(silyl)acetal and formaldehyde, preventing isolation of the silyl formate independently of the temperature.

### Experimental validation

**Fig. 7:**
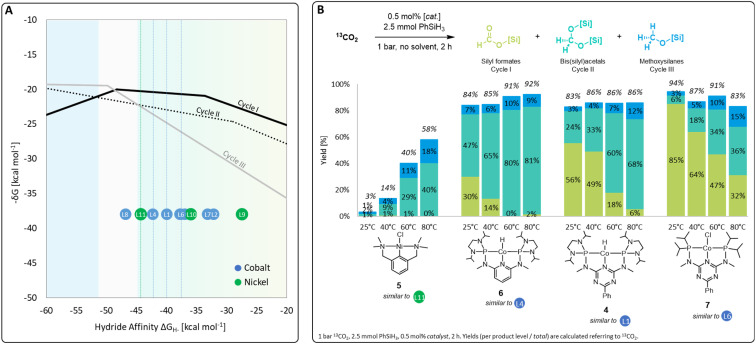
Top region of three volcano plots with indicated positions of selected catalyst candidates (A) and practical application of selected catalysts in CO_2_ hydrosilylation (B).

A set of metal complexes matching the key structural features of the computationally analyzed metal–ligand frameworks was synthesized and tested in catalysis to validate the trends and predictions resulting from the analysis of the volcano plots (Fig. 7). The catalysts were chosen by their close proximity to the top of the volcano plots of cycle I and thus by their high expected catalytic competence. The nickel chloride complex 5 was selected as a precursor to the active species [L11-Ni-H], while complexes 4, 6, and 7 correspond to [L1-Co-H], [L4-Co-H], and [L6-Co-H], respectively. Details on their preparation are given in the ESI.† Their catalytic performance was comparatively assessed using 0.5 mol% catalyst loading, 2.5 mmol PhSiH_3_ at 1 bar ^13^CO_2_ in the absence of solvents for 2 h. To elucidate to what extent the temperature influences the product distribution, catalytic experiments were performed at 25 °C, 40 °C, 60 °C, and 80 °C for each catalyst (Fig. 7B). Control experiments under similar conditions with only CoCl_2_ did not result in any product formation.^4*r*^

In the case of the nickel NCN pincer complex 5, the overall yield for C1 products drastically increases with the temperature from 3% (r.t.) to 58% (80 °C). This suggests that forming the active hydride complex from precursor 5 may require higher temperatures or longer reaction times. In accordance with the prediction, 5 turns out to be more suitable than the related PCP complex 1 which was reported inactive in the hydrosilylation of CO_2_ with phenylsilane.^4*d*^ In contrast to the use of the cobalt catalysts 6, 4, and 7, only traces of silyl formate are observed with complex 5 but mixtures of acetal and methoxide are obtained directly. The relatively highest activity for reduction beyond the formate level is in line with the lowest hydride affinity of 5 among the four examined catalysts. To increase the catalyst selectivity towards the methanol level, consequently, an ideal catalyst would employ an even lower hydride affinity and appear close to the volcano tops of all cycles. This would apply to the start of the plateau region of cycle III at −50 kcal mol^−1^. As shown previously for complex 4,^4*r*^ the methanol product yield might be increased additionally by employing a higher reaction temperature and/or longer reaction time.

The cobalt complexes 6, 4, and 7 reach overall yields between 83% and 94% of C1 reduction products already at room temperature as predicted from their position on the volcano plot. The selectivity is strongly temperature-dependent and the quantities of bis(silyl)acetals and methoxysilanes increase at the expense of the silyl formate yield at higher temperatures in all cases, which is in line with the relative energy spans of the three catalytic cycles I–III. Notably, the reduction capability beyond the formate level drastically increases in the series of 7, 4, and 6, as reflected in room temperature yields for bis(silyl)acetals increasing substantially from 6% (7), 24% (4), to 47% (6). In particular, complex 6 reaches 80% and 81% yield towards the formaldehyde level as the most challenging product at 60 °C and 80 °C, respectively. This significant improvement over the previously reported complex 4 would have been difficult to predict using conventional ligand effects due to the complexity of the reaction network. Notably, only a few catalytic systems are reported to facilitate the reduction of CO_2_ to formaldehyde and they often require precious metals, elevated pressure, (Lewis) acidic additives, or long reaction times.^4*f*,*g*,4*n*,14*a*–*d*^ In contrast, the base-metal catalyst 6 operates at ambient pressure, in the absence of additives, and reaches 81% yield at 80 °C in only 2 h.

## Conclusions

In summary, we mapped out the intrinsic reactivity of transition metal pincer hydride complexes to their activity and selectivity in the hydrosilylation of CO_2_ to the C1 products at the formate, formaldehyde, and methanol level. The approach utilized linear scaling relationships corroborating the hydride affinity of the catalysts with the energy spans of each of the three catalytic cycles corresponding to different product levels. The activity/selectivity trends were captured in a composite volcano picture to identify ligand/metal frameworks with high productivity for C1 products, their relative accessibility, and the possibility to control their relative production *via* external reaction parameters such as temperature. Leveraging this knowledge, a set of cobalt- and nickel-based catalyst candidates was experimentally examined and the observed activity and selectivity trends fully aligned with the prediction from the composite volcano. These results underpin the ability of such tandem computational-experimental efforts to drive the discovery of new selective catalysts even for complex reaction networks involving a series of intertwined catalytic cycles.

## Conflicts of interest

There are no conflicts to declare.

## Data availability

The data that support the findings of this study are available in the ESI.†

## Author contributions

H. H. C. performed the experiments, conducted the analytical characterization, and executed the theoretical calculations together with S. D. Together, H. H. C. and S. D. prepared the first draft of the manuscript. M. D. W. provided assistance with the theoretical calculations. C. C., C. W., and W. L. formulated, supervised, and directed the overall project. C. W. and W. L. revised the manuscript with the contribution of the other authors. All authors have approved the final version of the manuscript.

## Supplementary Material

SC-014-D2SC06793E-s001
