# Supplementary material for: Theory-guided development of homogeneous catalysts for the reduction of CO2 to formate, formaldehyde, and methanol derivatives
Source: Chem Sci. 2023 Feb 9;14(11):2799–807. doi: 10.1039/d2sc06793e (PMC10016328; doi:10.1039/d2sc06793e)
Supplement: SC-014-D2SC06793E-s001 [file SC-014-D2SC06793E-s001.pdf]

Electronic Supplementary Material (ESI) for Chemical Science.
This journal is © The Royal Society of Chemistry 2023

Supporting Information

Theory-Guided Development of Homogeneous Catalysts for the Reduction of CO_2 to Formate, Formaldehyde, and Methanol Derivatives

Hanna H. Cramer,[1,#] Shubhajit Das,[2,#] Matthew D. Wodrich,[2,3] Clémence Corminboeuf,[2,3,4,*] Christophe Werlé[1,5,*] and Walter Leitner*[1,6]

1 Max Planck Institute for Chemical Energy Conversion, Stiftstr. 34 – 36, 45470 Mülheim an der Ruhr, Germany.
2 Laboratory for Computational Molecular Design Institute of Chemical Sciences and Engineering, Ecole Polytechnique Fédérale de Lausanne (EPFL), 1015 Lausanne (Switzerland)
3 National Centre for Competence in Research – Catalysis (NCCR-Catalysis), École Polytechnique Fédérale de Lausanne (EPFL), 1015 Lausanne (Switzerland)
4 National Centre for Computational Design and Discovery of Novel Materials (MARVEL), Ecole Polytechnique Fédérale de Lausanne (EPFL), 1015 Lausanne (Switzerland)
5 Ruhr University Bochum, Universitätsstr. 150, 44801 Bochum, Germany.
6 Institut für Technische und Makromolekulare Chemie (ITMC), RWTH Aachen University, Worringer Weg 2, 52074 Aachen, Germany.

#H. H. C. and S. D. contributed equally to this work.
*Emails: clemence.corminboeuf@epfl.ch; christophe.werle@cec.mpg.de; walter.leitner@cec.mpg.de

Table of Contents

1. Molecular Volcano Plots S3

1.1. Computational Details S3

1.2. Linear Free Energy Scaling Relations (LFESRs) S4

2. Experimental Procedures S21

2.1. General Procedures S21

2.2. Synthesis pathways for **4**, **5**, and **6** S22

2.3. Synthetic Protocols S23

3. Hydrosilylation Experiments S44

4. Cartesian Coordinates of the Optimized Structures S53

4.1. Reactants and Products S53

4.2. Trityl Cation and Triphenylmethane S55

4.3. (**I1-H**)$^+$ S56

4.4. Intermediates S67

4.5. Transition States S128

5. References S193

1. Molecular Volcano Plots

1.1. Computational Details

All computations were performed using the Gaussian 16 program[1] with the M06 density functional.[2] For all intermediates and transition states, geometry optimizations and frequency calculations were conducted using the def2-SVPD basis set.[3] Loose optimization criteria, as implemented in Gaussian, were employed for the geometry optimization of selected challenging transition states. The final single point energies were obtained using the def2-TZVP basis set[3a] in implicit benzene solvation by using the SMD solvation model[4] and the UltraFine integration grid. Frequency calculations were performed to obtain the zero-point vibrational energies, the thermal corrections at 298.15 K, and the entropy contributions. All transition state geometries were identified as saddle points on the potential energy surface by their single imaginary frequency. A detailed procedure for the construction of the molecular volcano plots is explained in previous reports.[5]

1.2. Linear Free Energy Scaling Relations (LFESRs)

Table S1: Mathematical equations to describe the relative free energies of the intermediates and transition states with the hydride affinity ΔG_{H-}.

Structure		Slope				Intercept
ΔG(TS1)	=	0.29	·	ΔG_{H-}	+	21.22
ΔG(I2)	=	0.25	·	ΔG_{H-}	+	8.28
ΔG(TS2)	=	0.40	·	ΔG_{H-}	+	30.03
ΔG(I3)	=	0.62	·	ΔG_{H-}	+	26.34
ΔG(TS3)	=	0.31	·	ΔG_{H-}	+	31.35
ΔG(TS4)	=	0.26	·	ΔG_{H-}	+	27.00
ΔG(I4)	=	0.20	·	ΔG_{H-}	+	5.86
ΔG(TS5)	=	0.36	·	ΔG_{H-}	+	34.99
ΔG(I5)	=	0.53	·	ΔG_{H-}	+	26.26
ΔG(TS6)	=	0.39	·	ΔG_{H-}	+	33.84
ΔG(TS7)	=	0.54	·	ΔG_{H-}	+	46.51

Table S2: Energy difference equations for cycle I.

TDI	TDTS	δG		Slope				Intercept
I1	TS1	ΔG(I1->TS1)	=	-0.29	·	ΔG_{H-}	+	-21.22
	I2	ΔG(I1->I2)	=	-0.25	·	ΔG_{H-}	+	-8.28
	TS2	ΔG(I1->TS2)	=	-0.40	·	ΔG_{H-}	+	-30.03
	I3	ΔG(I1->I3)	=	-0.62	·	ΔG_{H-}	+	-26.34
	TS3	ΔG(I1->TS3)	=	-0.31	·	ΔG_{H-}	+	-31.35
I2	TS1	ΔG(I2->TS1)	=	-0.04	·	ΔG_{H-}	+	-15.12
	I1	ΔG(I2->I1)	=	0.25	·	ΔG_{H-}	+	6.11
	TS2	ΔG(I2->TS2)	=	-0.16	·	ΔG_{H-}	+	-21.76
	I3	ΔG(I2->I3)	=	-0.37	·	ΔG_{H-}	+	-18.07
	TS3	ΔG(I2->TS3)	=	-0.06	·	ΔG_{H-}	+	-23.07
I3	TS1	ΔG(I3->TS1)	=	0.34	·	ΔG_{H-}	+	2.95
	I1	ΔG(I3->I1)	=	0.62	·	ΔG_{H-}	+	24.17
	TS2	ΔG(I3->TS2)	=	0.22	·	ΔG_{H-}	+	-5.86
	I2	ΔG(I3->I3)	=	0.37	·	ΔG_{H-}	+	15.90
	TS3	ΔG(I3->TS3)	=	0.31	·	ΔG_{H-}	+	-5.01

Table S3: Energy difference equations for cycle II.

TDI	TDTS	δG		Slope				Intercept
I1	TS4	ΔG(I1->TS4)	=	-0.26	·	ΔG_{H-}	+	-27.00
	I4	ΔG(I1->I4)	=	-0.20	·	ΔG_{H-}	+	-5.86
	TS5	ΔG(I1->TS5)	=	-0.36	·	ΔG_{H-}	+	-34.99
	I5	ΔG(I1->I5)	=	-0.53	·	ΔG_{H-}	+	-26.26
	TS6	ΔG(I1->TS6)	=	-0.39	·	ΔG_{H-}	+	-33.84
I4	TS4	ΔG(I4->TS4)	=	-0.06	·	ΔG_{H-}	+	-12.59
	I1	ΔG(I4->I1)	=	0.26	·	ΔG_{H-}	+	35.56
	TS5	ΔG(I4->TS5)	=	-0.15	·	ΔG_{H-}	+	-29.13
	I5	ΔG(I4->I5)	=	-0.32	·	ΔG_{H-}	+	-20.39
	TS6	ΔG(I4->TS6)	=	-0.18	·	ΔG_{H-}	+	-27.98
I5	TS4	ΔG(I4->TS4)	=	0.26	·	ΔG_{H-}	+	7.80
	I4	ΔG(I5->I1)	=	0.32	·	ΔG_{H-}	+	28.95
	TS5	ΔG(I5->TS5)	=	0.17	·	ΔG_{H-}	+	-0.18
	I1	ΔG(I5->I1)	=	0.53	·	ΔG_{H-}	+	34.81
	TS6	ΔG(I5->TS6)	=	0.14	·	ΔG_{H-}	+	-7.59

Table S4: Energy difference equations for cycle III.

TDI	TDTS	δG		Slope				Intercept
I1	TS6	ΔG(I1->TS6)	=	-0.39	·	ΔG_{H-}	+	-33.84
	I5	ΔG(I1->I5)	=	-0.53	·	ΔG_{H-}	+	-26.26
	TS7	ΔG(I1->TS7)	=	-0.54	·	ΔG_{H}	+	-46.51
I5	TS6	ΔG(I5->TS6)	=	0.14	·	ΔG_{H-}	+	-0.61
	I1	ΔG(I5->I1)	=	0.53	·	ΔG_{H-}	+	33.23
	TS7	ΔG(I5->TS7)	=	-0.02	·	ΔG_{H}	+	-20.25

Table S5: Calculated descriptor values ΔG_{H^-} [kcal mol^{-1}].

Metal	Ligand	Descriptor ΔG_{H^-}
Co	L1	-39.9
	L2	-32.1
	L3	-37.7
	L4	-42.2
	L5	-36.9
	L6	-37.6
	L7	-33.1
	L8	-46.8
	L9	-84.6
	L10	-97.2
	L11	-99.4
Fe	L1	-91.4
	L2	-82.8
	L3	-89.7
	L4	-93.9
	L5	-82.9
	L6	-90.9
	L7	-85.2
	L8	-87.9
	L9	-130.3
	L10	-148.7
	L11	-148.4
Ni	L1	27.4
	L2	37.3
	L3	29.9
	L4	22.2
	L5	31.7
	L6	35.9
	L7	39.8
	L8	25.0
	L9	-27.4
	L10	-35.9
	L11	-44.3

Table S6: Electronic energies (def2-TZVP, PCM (benzene)), thermal corrections [Hartree], calculated free energies ΔG_{RRS} relative to the reference state **I1**, and ΔG_{RRS} estimated based on the descriptor value and the LFESRs. Entries of structures for which no convergence to the correct stationary point was achieved are left blank.

**Compound**			**E_{el} (def2-TZVP, PCM)**	**Therm. Corr.**		
CO_2			-188.5847934	-0.008534		
$PhSiH_3$			-522.8359384	0.083027		
$(PhH_2Si)OCHO$			-711.4385713	0.095787		
$(PhH_2SiO)_2CH_2$			-1234.316074	0.20675		
Formaldehyde			-114.4865327	0.004752		
$(PhH_2Si)_2O$			-1119.815742	0.177085		
$(PhH_2Si)OCH_2$			-637.3935999	0.112913		
$(Ph_3C)^+$			-732.535229	0.237293		
Ph_3CH			-733.3478882	0.244879		
**Structure**	**M**	**L**	**E_{el} (def2-TZVP, PCM)**	**Therm. Corr.**	**ΔG_{RRS}**	**ΔG_{RRS} (estimated, LFESRs)**
**[I1-H]$^+$**	Co	L1	-3069.501041	0.367241	-	-
		L2	-3030.672217	0.29379	-	-
		L3	-2958.735661	0.337464	-	-
		L4	-3037.377839	0.393747	-	-
		L5	-3406.572604	0.366641	-	-
		L6	-2693.3388	0.238612	-	-
		L7	-2991.882533	0.2213	-	-
		L8	-2009.315212	0.228866	-	-
		L9	-2644.67027	0.259488	-	-
		L10	-1921.812233	0.171339	-	-
		L11	-1960.677347	0.251319	-	-
	Fe	L1	-2950.524716	0.366676	-	-
		L2	-2911.712522	0.29265	-	-
		L3	-2839.767279	0.336211	-	-
		L4	-2918.39273	0.390849	-	-
		L5	-3287.602514	0.365679	-	-
		L6	-2574.360222	0.235634	-	-
		L7	-2872.921317	0.22054	-	-
		L8	-1890.325342	0.226824	-	-
		L9	-2525.60726	0.261438	-	-
		L10	-1802.735659	0.168306	-	-

		L11	-1841.589321	0.247583	-	-
	Ni	L1	-3194.833452	0.368346	-	-
		L2	-3155.980932	0.29162	-	-
		L3	-3084.064296	0.335096	-	-
		L4	-3162.721103	0.392805	-	-
		L5	-3531.899539	0.367785	-	-
		L6	-2818.663608	0.237837	-	-
		L7	-3117.189944	0.220437	-	-
		L8	-2134.642058	0.228445	-	-
		L9	-2770.105185	0.261159	-	-
		L10	-2047.244818	0.173475	-	-
		L11	-2086.127673	0.253436	-	-
**I1**	Co	L1	-3070.249213	0.373977	-	-
		L2	-3031.432501	0.300163	-	-
		L3	-2959.48415	0.341031	-	-
		L4	-3038.119866	0.397995	-	-
		L5	-3407.32597	0.373807	-	-
		L6	-2694.088855	0.24348	-	-
		L7	-2992.640021	0.22649	-	-
		L8	-2010.051672	0.234828	-	-
		L9	-2645.34375	0.262776	-	-
		L10	-1922.465694	0.174574	-	-
		L11	-1961.327085	0.254353	-	-
	Fe	L1	-2951.18974	0.372205	-	-
		L2	-2912.387126	0.294161	-	-
		L3	-2840.433625	0.340479	-	-
		L4	-2919.05345	0.396093	-	-
		L5	-3288.278164	0.368356	-	-
		L6	-2575.023143	0.238262	-	-
		L7	-2873.594049	0.223959	-	-
		L8	-1890.992362	0.228904	-	-
		L9	-2526.198844	0.255595	-	-
		L10	-1803.303645	0.16825	-	-
		L11	-1842.157918	0.247554	-	-
	Ni	L1	-3195.690179	0.376369	-	-
		L2	-3156.855184	0.301399	-	-

		L3	-3084.924909	0.342915	-	-
		L4	-3163.571101	0.402353	-	-
		L5	-3532.761232	0.373851	-	-
		L6	-2819.532751	0.244618	-	-
		L7	-3118.0664	0.228403	-	-
		L8	-2135.496469	0.238013	-	-
		L9	-2770.871268	0.265811	-	-
		L10	-2047.994643	0.175446	-	-
		L11	-2086.868948	0.260179	-	-
**TS1**	Co	L1	-3258.828985	0.383824	14.7	9.84
		L2	-3220.00987	0.307951	14.9	12.07
		L3	-3148.066637	0.352957	14.3	10.46
		L4	-3226.700426	0.408823	14.8	9.18
		L5	-3595.905061	0.383952	15.3	10.69
		L6	-2882.667295	0.25191	14.6	10.51
		L7				
		L8				
		L9				
		L10				
		L11				
	Fe	L1	-3139.812019	0.384603	-10.4	-4.83
		L2	-3101.005324	0.308626	-6.5	-2.39
		L3	-3029.053015	0.352391	-8.9	-4.36
		L4	-3107.676114	0.407509	-11.2	-5.55
		L5	-3476.893901	0.383598	-4.5	-2.41
		L6	-2763.639067	0.250672	-6.4	-4.68
		L7				
		L8	-2079.610467	0.241295	-7.8	-3.85
		L9	-2714.833579	0.269426	-17.3	-15.93
		L10				
		L11	-2030.796437	0.259763	-20.7	-21.09
	Ni	L1	-3384.251698	0.388665	27.7	29.03
		L2				
		L3	-3273.489971	0.353538	24.4	29.76
		L4				
		L5	-3721.322712	0.386428	27.9	30.27

		L6				
		L7	-3306.627357	0.238277	26.5	32.57
		L8	-2324.063547	0.248387	23.0	28.34
		L9				
		L10	-2236.565524	0.187066	21.4	10.99
		L11	-2275.436353	0.26849	21.5	8.60
**I2**	Co	L1	-3258.85824	0.389165	-0.3	-1.56
		L2	-3220.037387	0.312557	0.5	0.37
		L3	-3148.094291	0.357469	-0.2	-1.02
		L4	-3226.729481	0.412049	-1.4	-2.13
		L5	-3595.933819	0.387325	-0.6	-0.82
		L6	-2882.694929	0.257062	0.5	-0.98
		L7	-3181.24395	0.239325	1.4	0.12
		L8	-2198.672921	0.245896	-10.6	-3.25
		L9	-2833.964423	0.276754	-8.4	-12.57
		L10	-2111.102259	0.186429	-19.7	-15.66
		L11	-2149.964914	0.267381	-19.7	-16.20
	Fe	L1	-3139.81801	0.385356	-13.7	-14.22
		L2	-3101.011815	0.310975	-9.1	-12.12
		L3	-3029.060926	0.353857	-12.9	-13.82
		L4	-3107.680694	0.408069	-13.8	-14.84
		L5	-3476.905057	0.384645	-10.8	-12.14
		L6	-2763.649254	0.251702	-12.1	-14.10
		L7	-3062.217734	0.235882	-11.6	-12.71
		L8	-2079.628838	0.241529	-19.2	-13.38
		L9	-2714.836416	0.269893	-18.8	-23.82
		L10	-1991.954636	0.181858	-27.6	-28.36
		L11	-2030.809982	0.261316	-28.2	-28.27
	Ni	L1	-3384.271126	0.388984	15.7	15.02
		L2	-3345.430443	0.311847	17.9	17.46
		L3	-3273.506095	0.358411	17.3	15.65
		L4	-3352.152155	0.41346	14.7	13.74
		L5	-3721.340866	0.389016	18.1	16.09
		L6	-3008.108509	0.25712	18.9	17.13
		L7	-3306.639101	0.241025	20.9	18.08
		L8	-2324.086859	0.248765	8.6	14.42

		L9	-2959.47077	0.278142	3.9	1.53
		L10	-2236.61088	0.18801	-6.5	-0.57
		L11	-2275.486789	0.271038	-8.6	-2.63
**TS2**	Co	L1	-3781.693974	0.498974	16.6	13.88
		L2	-3742.870157	0.421117	18.5	17.05
		L3	-3670.928443	0.465582	16.6	14.77
		L4	-3749.565844	0.521899	15.2	12.96
		L5	-4118.770078	0.497763	16.4	15.09
		L6	-3405.521506	0.361863	20.1	14.84
		L7	-3704.076983	0.345695	17.9	16.64
		L8				
		L9	-3356.808536	0.382547	0.8	-4.19
		L10	-2633.938502	0.290306	-6.8	-9.26
		L11	-2672.802403	0.372542	-6.8	-10.15
	Fe	L1				
		L2	-3623.872861	0.420562	-8.2	-3.46
		L3				
		L4				
		L5				
		L6	-3286.509483	0.361874	-10.3	-6.70
		L7	-3585.082533	0.343041	-14.5	-4.42
		L8				
		L9	-3237.712715	0.37806	-28.3	-22.66
		L10				
		L11				
	Ni	L1	-3907.099151	0.500156	38.3	41.10
		L2	-3868.258766	0.422726	40.2	45.10
		L3	-3796.333767	0.465574	37.7	42.14
		L4				
		L5				
		L6	-3530.93289	0.364879	41.6	44.57
		L7	-3829.458247	0.347938	46.4	46.12
		L8	-2846.893623	0.356618	42.5	40.12
		L9	-3482.298716	0.387245	25.2	18.96
		L10	-2759.432991	0.298162	19.2	15.51
		L11				

**I3**	Co	L1	-3781.716089	0.498477	2.4	1.57
		L2	-3742.888148	0.418866	5.8	6.43
		L3	-3670.965812	0.467838	-5.4	2.93
		L4				
		L5	-4118.7844	0.496129	6.4	3.42
		L6	-3405.560048	0.365765	-1.7	3.03
		L7	-3704.096531	0.347682	6.9	5.80
		L8	-2721.513124	0.356269	3.9	-2.69
		L9	-3356.833252	0.385535	-12.9	-26.16
		L10	-2633.949896	0.293504	-11.9	-33.94
		L11	-2672.815388	0.376685	-12.4	-35.31
	Fe	L1	-3662.720143	0.493238	-39.6	30.33
		L2	-3623.90848	0.417349	-32.6	-25.03
		L3				
		L4	-3630.590885	0.520136	-42.1	-31.89
		L5	-3999.798796	0.493603	-30.8	-25.08
		L6	-3286.558619	0.360367	-42.1	-30.02
		L7	-3585.112228	0.344635	-32.2	-26.51
		L8	-2602.509219	0.350675	-30.7	-28.21
		L9	-3237.762826	0.379215	-59.1	-54.49
		L10	-2514.878243	0.286946	-68.8	-65.93
		L11	-2553.737445	0.366637	-71.7	-65.70
	Ni	L1	-3907.089441	0.49792	43.0	43.32
		L2	-3868.246295	0.419306	45.8	49.46
		L3	-3796.347118	0.468658	31.2	44.92
		L4				
		L5	-4244.162588	0.499164	44.0	46.02
		L6				
		L7	-3829.455565	0.346662	47.3	51.03
		L8	-2846.877805	0.357685	53.1	41.82
		L9	-3482.285301	0.384465	31.9	9.35
		L10				
		L11				
**TS3**	Co	L1	-3781.687261	0.498637	20.6	19.00
		L2	-3742.863805	0.420236	22.0	21.42
		L3	-3670.920342	0.464469	21.0	19.68

		L4	-3749.5591	0.521729	19.3	18.29
		L5	-4118.763071	0.499131	21.6	19.93
		L6	-3405.521506	0.361875	20.1	19.73
		L7	-3704.071629	0.346726	21.9	21.11
		L8	-2721.487858	0.354808	18.8	16.88
		L9	-3356.796497	0.383489	8.9	5.18
		L10				
		L11				
	Fe	L1	-3662.663485	0.494605	-3.2	3.11
		L2	-3623.854882	0.42054	3.1	5.74
		L3	-3551.900017	0.464022	2.1	3.61
		L4	-3630.524809	0.516487	-3.0	2.33
		L5	-3999.743662	0.494364	4.2	5.72
		L6	-3286.483098	0.361506	6.0	3.26
		L7				
		L8				
		L9				
		L10	-2514.790203	0.289178	-12.2	-14.64
		L11				
	Ni	L1				
		L2	-3868.251791	0.420625	43.2	42.87
		L3				
		L4	-3874.986792	0.522061	31.5	38.21
		L5				
		L6				
		L7	-3829.461264	0.347785	44.4	43.65
		L8				
		L9	-3482.297811	0.385963	25.0	22.88
		L10	-2759.425832	0.296721	22.8	20.25
		L11				
**TS4**	Co	L1				
		L2	-3742.873993	0.422977	17.3	18.56
		L3	-3670.929542	0.468156	17.6	17.08
		L4	-3749.56307	0.521219	16.5	15.90
		L5				
		L6	-3405.523851	0.364369	20.2	17.13

		L7	-3704.079378	0.348715	18.3	18.30
		L8	-2721.480886	0.353902	22.7	14.70
		L9	-3356.785482	0.383694	16.0	4.75
		L10				
		L11				
	Fe	L1	-3662.666357	0.496209	-4.0	2.99
		L2	-3623.860502	0.421337	0.0	5.23
		L3	-3551.907416	0.462258	-3.6	3.42
		L4	-3630.52848	0.518642	-3.9	2.33
		L5	-3999.75041	0.496541	1.4	5.21
		L6	-3286.486576	0.360477	3.2	3.12
		L7				
		L8	-2602.466641	0.350905	-3.8	3.89
		L9	-3237.671767	0.377631	-2.9	-7.25
		L10	-2514.790529	0.287847	-13.2	-12.10
		L11	-2553.644225	0.369945	-11.1	-12.00
	Ni	L1	-3907.109235	0.500991	32.5	34.20
		L2				
		L3	-3796.346641	0.471324	33.2	34.88
		L4	-3874.988305	0.524756	32.3	32.84
		L5	-4244.180872	0.499252	32.6	35.34
		L6	-3530.944236	0.366499	35.5	36.45
		L7	-3829.479001	0.348482	33.7	37.47
		L8	-2846.910803	0.35649	31.6	33.56
		L9	-3482.289017	0.386449	30.8	19.80
		L10				
		L11	-2798.288547	0.378986	28.5	15.37
**I4**	Co	L1	-3781.724929	0.502022	-0.9	-2.23
		L2	-3742.907898	0.426734	-1.6	-0.65
		L3	-3670.958127	0.470062	0.8	-1.79
		L4	-3749.595951	0.525255	-1.6	-2.70
		L5	-4118.801032	0.502091	-0.3	-1.63
		L6	-3405.560417	0.368977	0.1	-1.76
		L7	-3704.10923	0.351911	1.5	-0.85
		L8	-2721.532626	0.360092	-5.9	-3.62
		L9	-3356.830335	0.388229	-9.3	-11.29

		L10	-2633.967145	0.29844	-19.7	-13.83
		L11	-2672.825218	0.378833	-17.2	-14.28
	Fe	L1	-3662.687447	0.499825	-15.0	-12.66
		L2				
		L3				
		L4	-3630.549684	0.522442	-14.8	-13.17
		L5				
		L6	-3286.516052	0.365715	-12.1	-12.55
		L7	-3585.086245	0.350177	-12.4	-11.41
		L8	-2602.491921	0.355801	-16.6	-11.96
		L9	-3237.709883	0.384739	-22.4	-20.55
		L10	-2514.799997	0.289	-18.4	-24.29
		L11	-2553.655067	0.368325	-18.9	-24.21
	Ni	L1	-3907.143876	0.503211	12.2	11.41
		L2	-3868.312018	0.427274	9.6	13.42
		L3	-3796.375451	0.470515	14.6	11.93
		L4	-3875.024141	0.526236	10.7	10.36
		L5	-4244.214233	0.500017	12.2	12.29
		L6	-3530.983772	0.370508	13.2	13.15
		L7	-3829.510908	0.353104	16.6	13.93
		L8	-2846.951619	0.362581	9.8	10.92
		L9	-3482.338197	0.393278	4.3	0.31
		L10	-2759.478151	0.302978	-6.1	-1.42
		L11	-2798.34635	0.38404	-4.6	-3.11
**TS5**	Co	L1				
		L2	-4265.724771	0.535132	26.3	23.53
		L3	-4193.782981	0.578674	23.8	21.52
		L4	-4272.417872	0.633662	23.1	19.92
		L5	-4641.622581	0.609021	23.7	21.81
		L6	-3928.387649	0.476178	20.7	21.58
		L7	-4226.939832	0.461767	21.7	23.17
		L8	-3244.350171	0.465537	19.7	18.29
		L9	-3879.667533	0.496449	5.7	4.79
		L10				
		L11	-3195.645608	0.484231	6.6	-0.47
	Fe	L1				

		L2				
		L3				
		L4	-4153.391718	0.629795	-3.4	1.50
		L5				
		L6				
		L7				
		L8				
		L9	-3760.562917	0.490873	-18.6	-11.50
		L10				
		L11				
	Ni	L1				
		L2	-4391.11921	0.536802	44.3	48.29
		L3	-4319.192764	0.579509	42.6	45.67
		L4	-4397.837749	0.635654	41.3	42.91
		L5				
		L6				
		L7				
		L8	-3369.752571	0.469153	46.6	43.89
		L9				
		L10	-3282.29015	0.408627	23.1	22.18
		L11				
**I5**	Co	L1	-4304.572504	0.608576	6.6	5.23
		L2	-4265.751535	0.5345	9.1	9.35
		L3				
		L4				
		L5	-4641.648717	0.611318	8.7	6.80
		L6	-3928.412522	0.476827	5.5	6.47
		L7	-4226.952415	0.460484	13.0	8.82
		L8	-3244.357909	0.466986	15.7	1.61
		L9				
		L10	-3156.802663	0.405522	-4.3	-24.91
		L11				
	Fe	L1	-4185.571222	0.608521	-28.9	-21.85
		L2	-4146.763365	0.530088	-25.8	-17.35
		L3	-4074.81223	0.572606	-29.7	-20.99
		L4				

		L5				
		L6	-3809.408131	0.4729	-32.1	-21.58
		L7	-4107.958902	0.454487	-22.1	-18.60
		L8	-3125.3492	0.459947	-16.7	-20.05
		L9				
		L10				
		L11				
	Ni	L1	-4429.954414	0.610441	43.3	40.67
		L2				
		L3	-4319.202201	0.576586	34.8	42.02
		L4				
		L5				
		L6	-4053.79197	0.477312	45.6	45.19
		L7	-4352.325626	0.460696	45.3	47.21
		L8				
		L9				
		L10				
		L11				
**TS6**	Co	L1	-4304.558802	0.610896	16.6	18.39
		L2				
		L3				
		L4	-4272.429377	0.634483	16.4	17.50
		L5				
		L6				19.30
		L7	-4226.95419	0.46178	12.7	21.03
		L8	-3244.351546	0.46587	19.0	15.73
		L9	-3879.66557	0.495979	6.6	1.09
		L10				
		L11	-3195.647084	0.485763	6.6	-4.61
	Fe	L1				
		L2				
		L3				
		L4				
		L5				
		L6	-3809.362469	0.47652	-1.2	-1.31
		L7	-4107.940359	0.459384	-7.4	0.88

		L8				
		L9	-3760.56581	0.490372	-20.7	-16.58
		L10	-3037.677171	0.401166	-26.0	-23.72
		L11				
	Ni	L1				
		L2				
		L3				
		L4				
		L5				
		L6				
		L7				
		L8				
		L9	-4005.174344	0.502232	20.4	23.24
		L10	-3282.285616	0.412821	28.6	19.95
		L11	-3321.174022	0.492618	16.6	16.71
**TS7**	Co	L1				
		L2				
		L3				
		L4	-4272.408582	0.634082	29.2	23.59
		L5	-4641.610046	0.609561	31.9	26.46
		L6	-3928.373771	0.472491	27.1	26.11
		L7				
		L8				
		L9				
		L10				
		L11				
	Fe	L1				
		L2				
		L3	-4074.76155	0.570478	0.8	-2.20
		L4	-4153.392245	0.626296	-5.9	-4.45
		L5				
		L6				
		L7				
		L8				
		L9				
		L10				

	L11	-3076.547905	0.473221	-40.9	-34.03
Ni	L1				
	L2	-4391.103839	0.535501	53.1	66.74
	L3				
	L4	-4397.808213	0.635764	59.9	58.56
	L5				
	L6	-4053.770577	0.47749	59.1	66.02
	L7				
	L8	-3369.712111	0.470206	72.6	60.06
	L9				
	L10				
	L11				

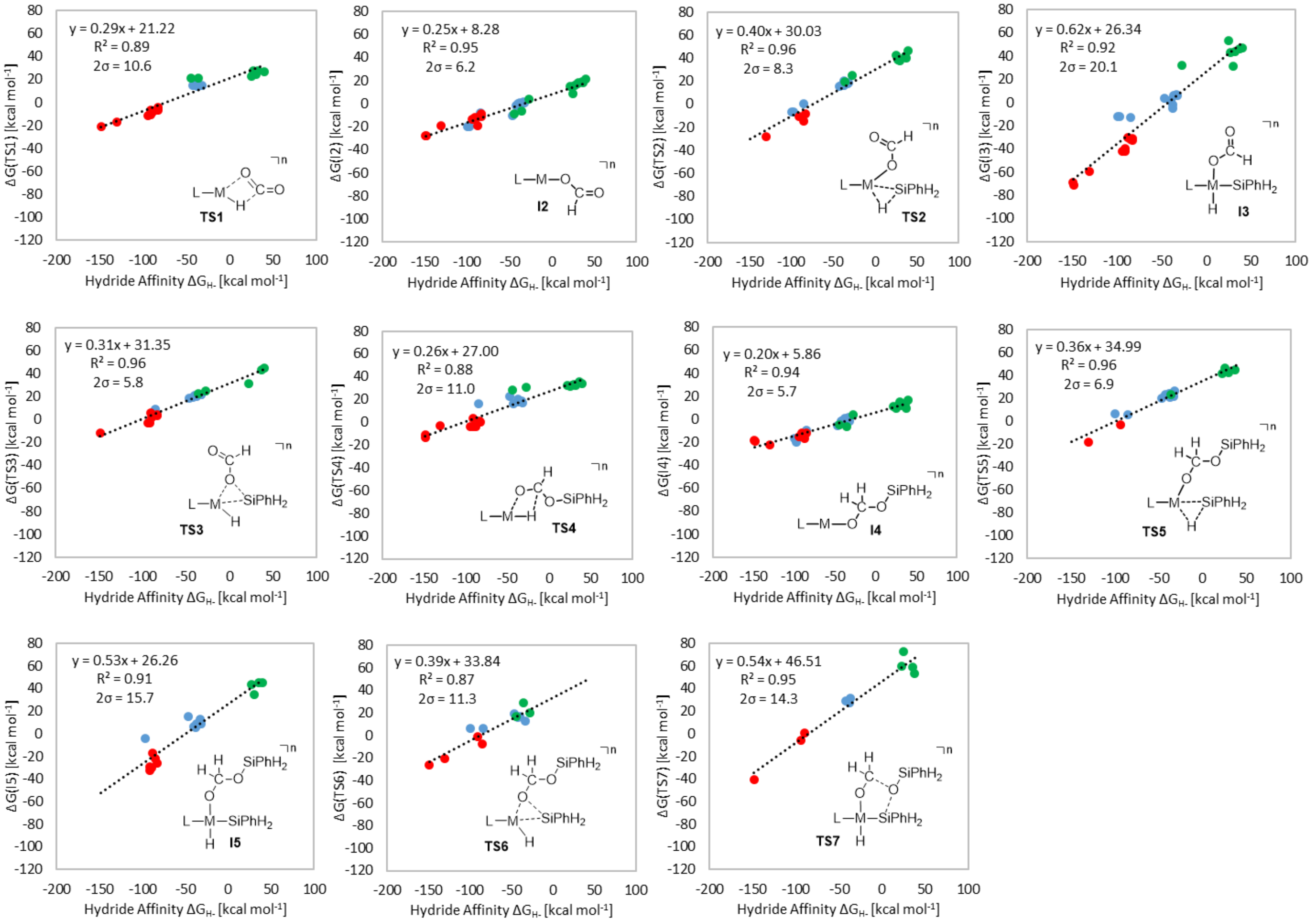

Figure S1: Linear free energy scaling relationships of ΔG [kcal mol^{-1}] on the hydride affinity ΔG_{H^-} [kcal mol^{-1}].

2. Experimental Procedures

2.1. General Procedures

All air and moisture-sensitive experiments were conducted under a dry argon atmosphere using standard Schlenk techniques or an MBraun inert-gas glovebox. The solvents for air- and moisture-sensitive experiments were purified with a two-column solvent purification system (MBraun-SPS-7) and transferred to the glovebox under an atmosphere of purified argon. Deuterated solvents were degassed prior to storage over activated molecular sieves. All chemicals including the starting materials **7** and **11** were purchased from ABCR chemicals, Sigma-Aldrich, and TCI and used without further purification. The compounds **3**, **8**, and **12** were prepared following literature procedures.[6]

NMR spectra were recorded on Bruker AVANCE III HD and Bruker AV-400 spectrometers. Chemical shifts (δ) are given in ppm relative to tetramethylsilane (TMS) and coupling constants (*J*) in Hz (s: singlet, d: duplet, t: triplet, sept: septet, m: multiplet, br.: broad). The solvent signals were used as references and the chemical shifts converted to the TMS scale (C_6D_6: δ^1H = 7.16 ppm, $\delta^{13}C$ = 128.1 ppm; toluene-d_8: δ^1H = 2.09, 6.98, 7.00, 7.09, $\delta^{13}C$ = 125.5, 128.3, 129.2, 137.9; CD_3CN: δ^1H = 1.94 ppm, $\delta^{13}C$ = 118.26 ppm; DMSO-d_6: δ^1H = 2.50 ppm, $\delta^{13}C$ = 39.5 ppm; THF-d_8: δ^1H = 1.72, 3.58 ppm, $\delta^{13}C$ = 25.3, 67.2 ppm). For quantification, the $^{13}C\{^1H\}$ spectra were recorded using a 90° pulse with ^1H inverse gated decoupling (pulse program zgig), a recycling delay of 60 s, and by accumulating 64 scans. All spectra were thoroughly phased, baseline-corrected, and integrated to provide quantitative results referring to mesitylene as the internal reference.

HR-MS spectra were recorded on a Bruker ESQ3000 spectrometer, and CHN elemental microanalyses were measured at “Mikroanalytisches Labor Kolbe” (c/o Fraunhofer Institut UMSICHT). Infrared spectra were measured on a Thermo Scientific Nicolet™ iS5 Spectrometer with an ID7 ATR accessory, and UV-vis spectra were recorded with a Thermo Scientific Evolution 201 UV-visible spectrophotometer.

2.2. Synthesis pathways for 4, 5, and 6

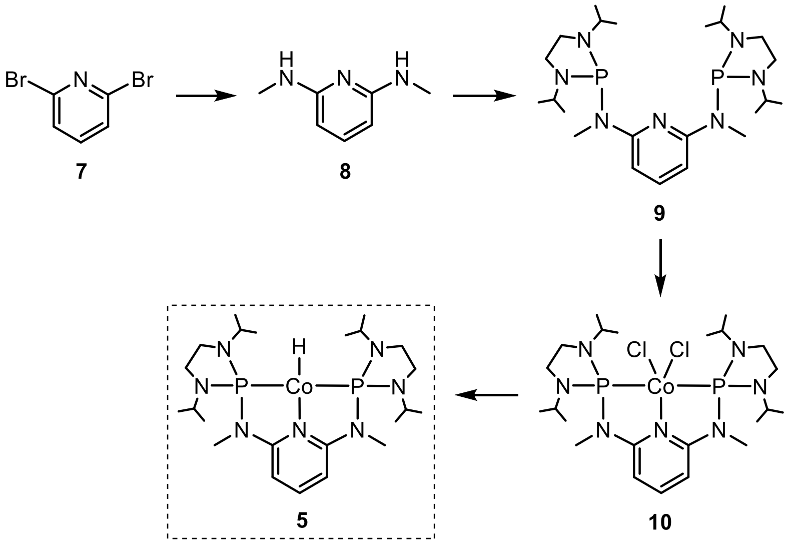

Scheme 1: Synthesis of **5**.

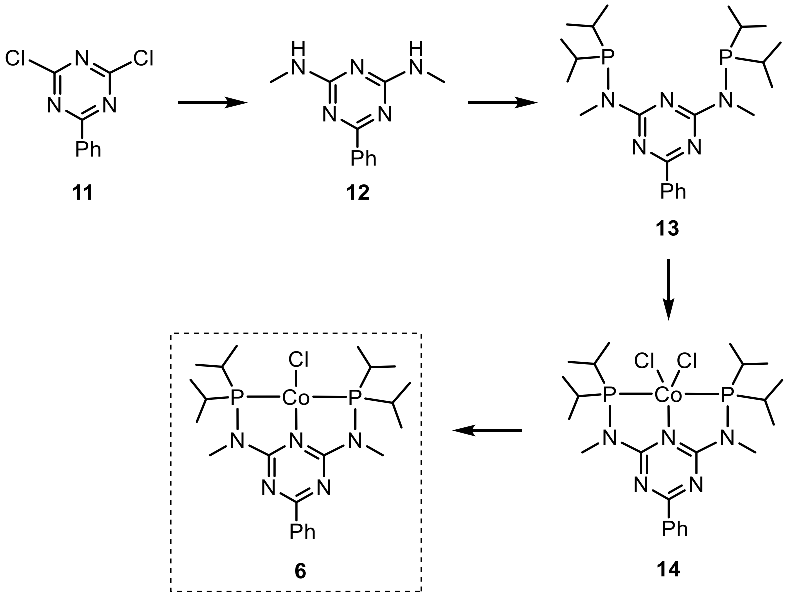

Scheme 2: Synthesis of **6**.

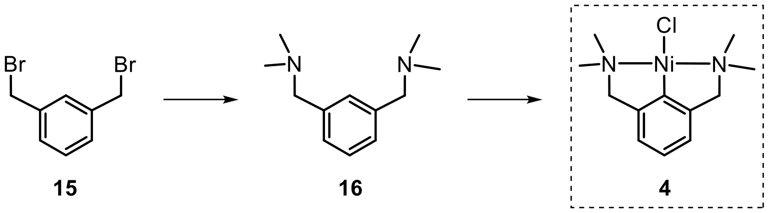

Scheme 3: Synthesis of **4**.

2.3. Synthetic Protocols

2.3.1. Synthesis of 9

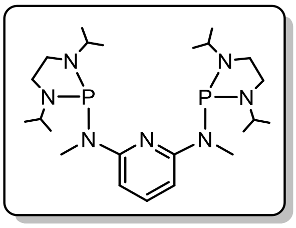

N,N-Diisopropylethylenediamine (1.3 ml, 7.15 mmol) was added to triethylamine (2.49 ml, 17.9 mmol) in toluene solution (20 ml). After stirring the reaction mixture at room temperature for 10 minutes, PCl_3 (0.62 ml, 7.15 mmol) was added dropwise to the flask. The mixture was stirred for 2 h and subsequently filtrated over a glass frit before adding 2,6-bis(methylamino)pyridine **8** (0.446 g, 3.25 mmol) and lithium bis(trimethylsilyl)amide (1.25 g, 7.47 mmol) to the solution. The mixture was stirred for 12 h at room temperature. Filtration and evaporation of the solvent gave the crude product **9** as brown oil (1.40 g, 2.91 mmol, 90%) that was used in the following reactions without further purification.

^{1}H NMR (400 MHz, CD_2Cl_2, 298 K): δ 1.15 (d, $^3J_{H,H}$ = 6.6 Hz, 12 H, ^{i}Pr-C***H***$_3$), 1.17 (d, $^3J_{H,H}$ = 6.6 Hz, 12 H, ^{i}Pr-C***H***$_3$), 2.90 (s, 6 H, NC***H***$_3$,), 3.04-3.13 (m, 4 H, C***H***$_2$), 3.33-3.45 (overlapping, 8 H, ^{i}Pr-C***H***, C***H***$_2$), 6.70 (d, $^3J_{H,H}$ = 8.0 Hz, 2 H, Ar-*m*-C***H***), 7.20 (t, $^3J_{H,H}$ = 7.9 Hz, 1 H, Ar-*p*-C***H***).

^{13}C{^{1}H} NMR (101 MHz, C_6D_6, 298 K): δ 22.2 (d, $^3J_{P,C}$ = 8.3 Hz, ^{i}Pr-***C***H$_3$), 22.6 (d, $^3J_{P,C}$ = 8.5 Hz, ^{i}Pr-***C***H$_3$), 31.2 (d, $^2J_{P,C}$ = 6.5 Hz, N-***C***H$_3$), 46.5 (d, $^2J_{P,C}$ = 8.9 Hz, ***C***H$_2$), 48.5 (d, $^2J_{P,C}$ = 22.1 Hz, ^{i}Pr-***C***H), 100.0 (d, $^2J_{P,C}$ = 27.7 Hz, Ar-*m*-***C***H), 137.3 (t, $^4J_{P,C}$ = 3.5 Hz, Ar-*p*-***C***H), 159.5 (d, $^2J_{P,C}$ = 22.3 Hz, Ar-*o*-***C***).

^{31}P{^{1}H} NMR (162 MHz, C_6D_6, 298 K): δ 95.4 (s).

HR-MS (ESI$^+$): m/z: calcd. for $[C_{23}H_{46}N_7P_2]^+$: 482.328444; found: 482.328640.

IR (Diamond ATR cell, cm^{-1}): 2960, 2924, 1571, 1392, 1360, 1329, 1267, 1246, 1176, 1151, 1115, 1059, 952, 934, 858, 843, 807, 779, 727, 702, 587, 551.

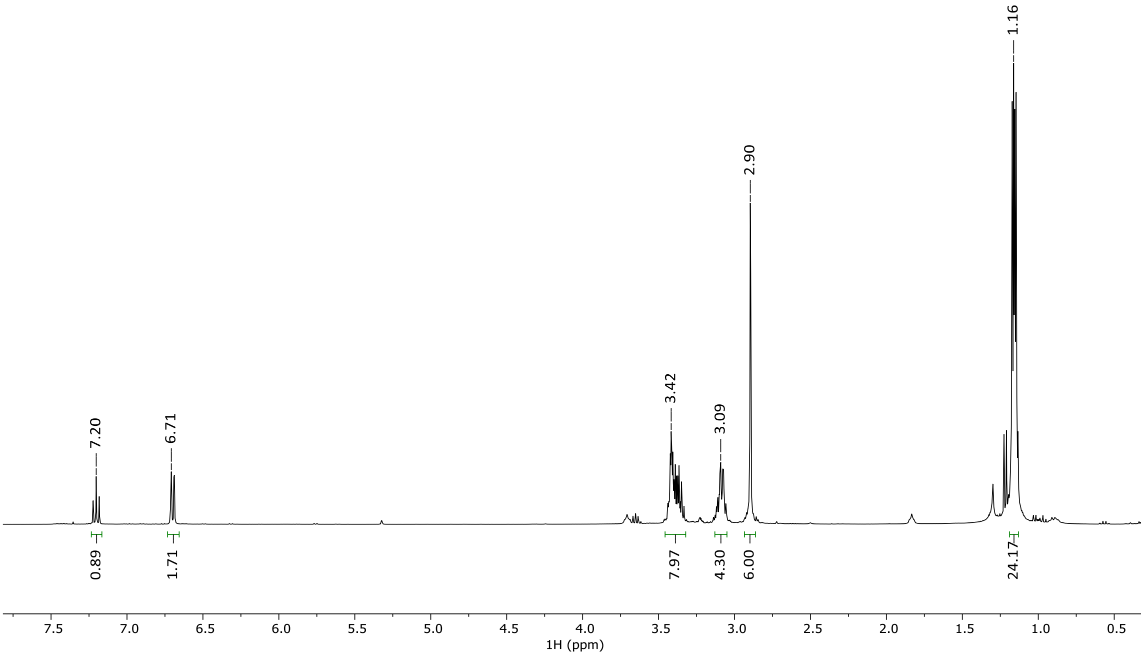

Figure S2: ^{1}H NMR spectrum (400 MHz, CD_2Cl_2, 298 K) of **9**.

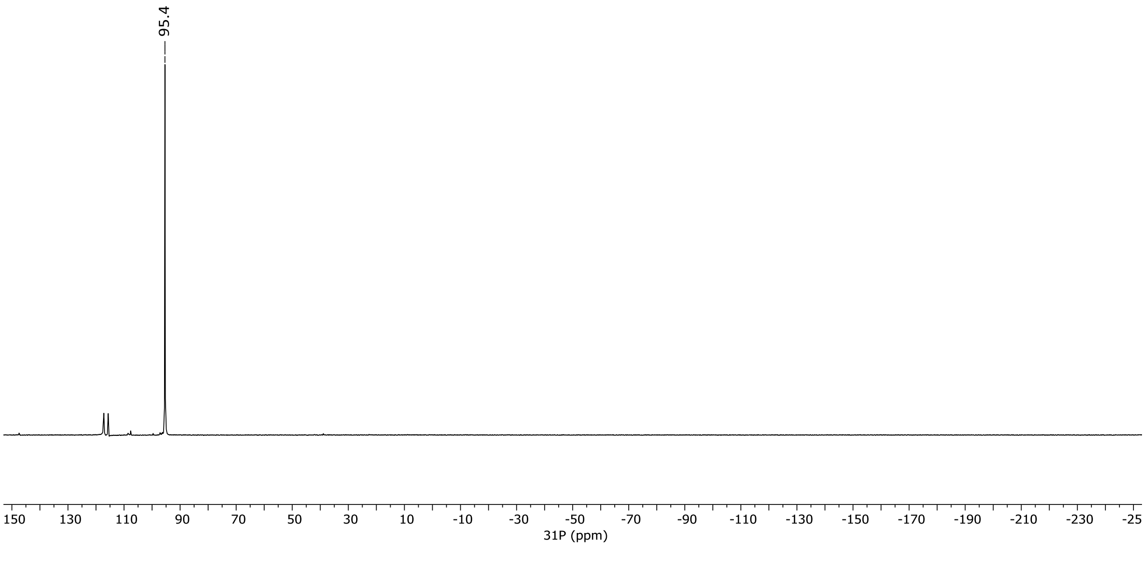

Figure S3: $^{31}P\{^{1}H\}$ NMR spectrum (162 MHz, CD_2Cl_2, 298 K) of **9**.

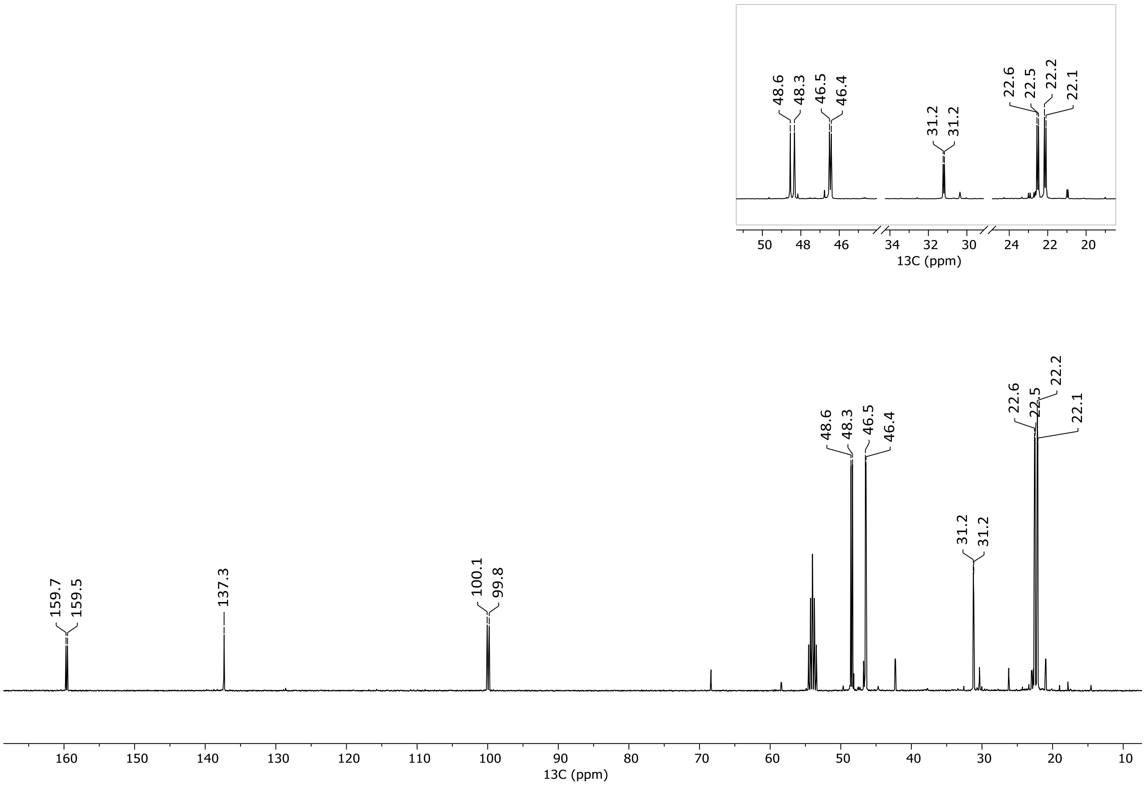

Figure S4: $^{13}C\{^{1}H\}$ NMR spectrum (101 MHz, 298 K, CD_2Cl_2) of **9**.

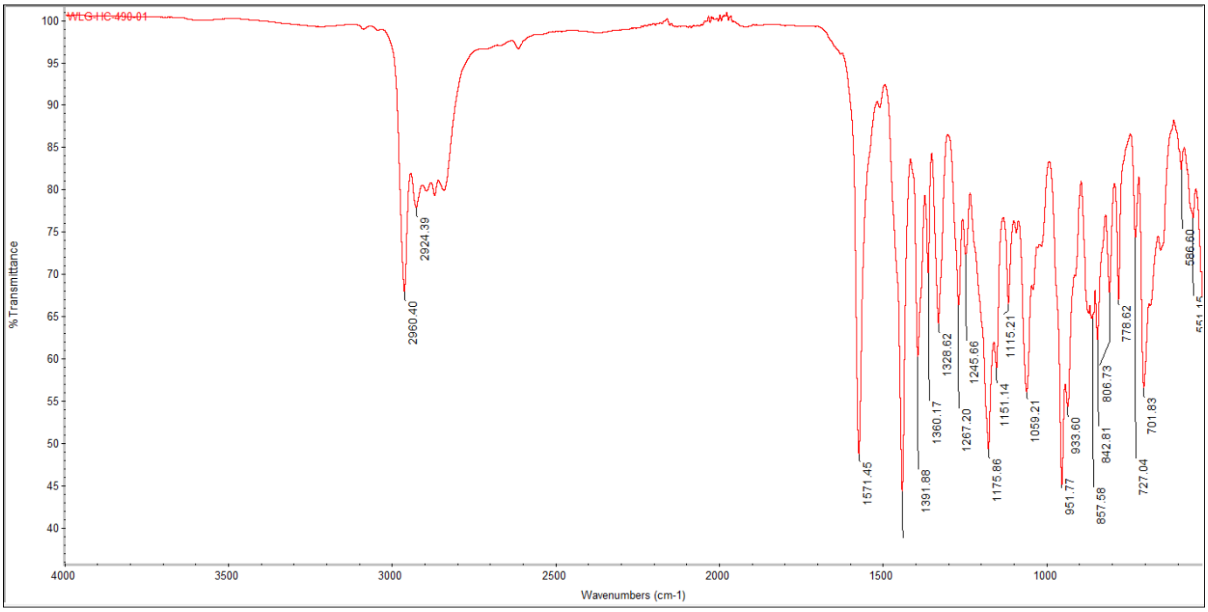

Figure S5: IR spectrum of **9**.

2.3.2. Synthesis of 10

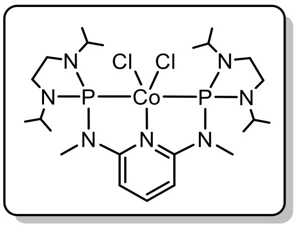

A solution of **9** (200 mg, 0.416 mmol, 10% in THF) was added to a suspension of $CoCl_2$ (54.0 mg, 0.416 mmol) in THF (20 ml) and the mixture was stirred at room temperature for 12 h. After filtration through Celite, the solvent was removed *in vacuo* and the residue washed with pentane (3 x 8 ml) and dichloromethane (1 x 8 ml). Removal of the solvent *in vacuo* gave the product **10** as a brown solid (198 mg, 0.324 mmol, 78%).

The resonances in the ^{1}H, ^{13}C{^{1}H} and ^{31}P{^{1}H} NMR spectra could not be assigned due to the line broadening and wide shift range caused by the paramagnetism of the product.

HR-MS (ESI$^+$): m/z: calcd. for $[C_{23}H_{45}N_7P_2CoCl_2]^+$: 610.19152; found: 610.19067.

Anal. calcd. (%) for $[(C_{23}H_{45}Cl_2CoN_7P_2)(CH_2Cl_2)_{0.2}]$: C 44.14, 7.25, N 15.50; found: C 44.34, H 7.39, N 15.74.

IR (Diamond ATR cell, cm^{-1}): 2962, 2866, 2158, 1590, 1567, 1462, 1406, 1390, 1363, 1299, 1220, 1170, 1121, 1104, 1090, 1054, 1003, 966, 928, 854, 834, 769, 739, 678.

UV/vis (1 µM in dichloromethane): 322.23 nm.

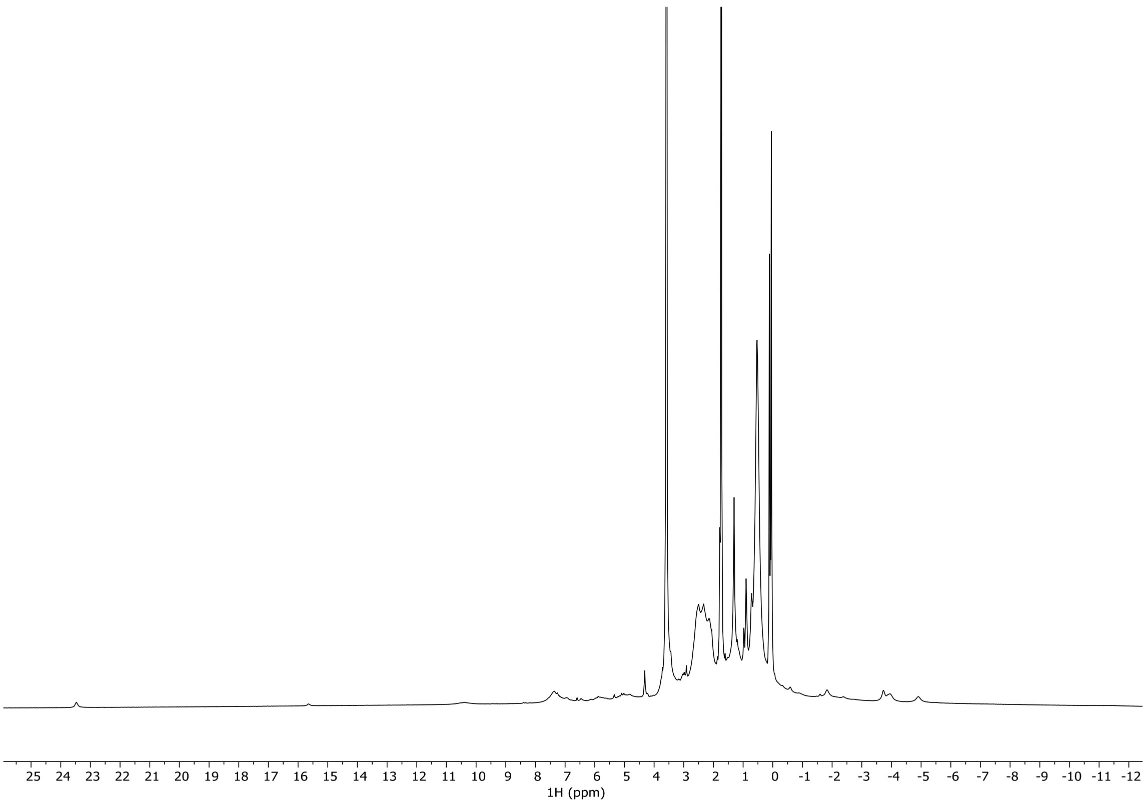

Figure S6: ^{1}H NMR spectrum (500 MHz, C_6D_6, 298 K) of **10**.

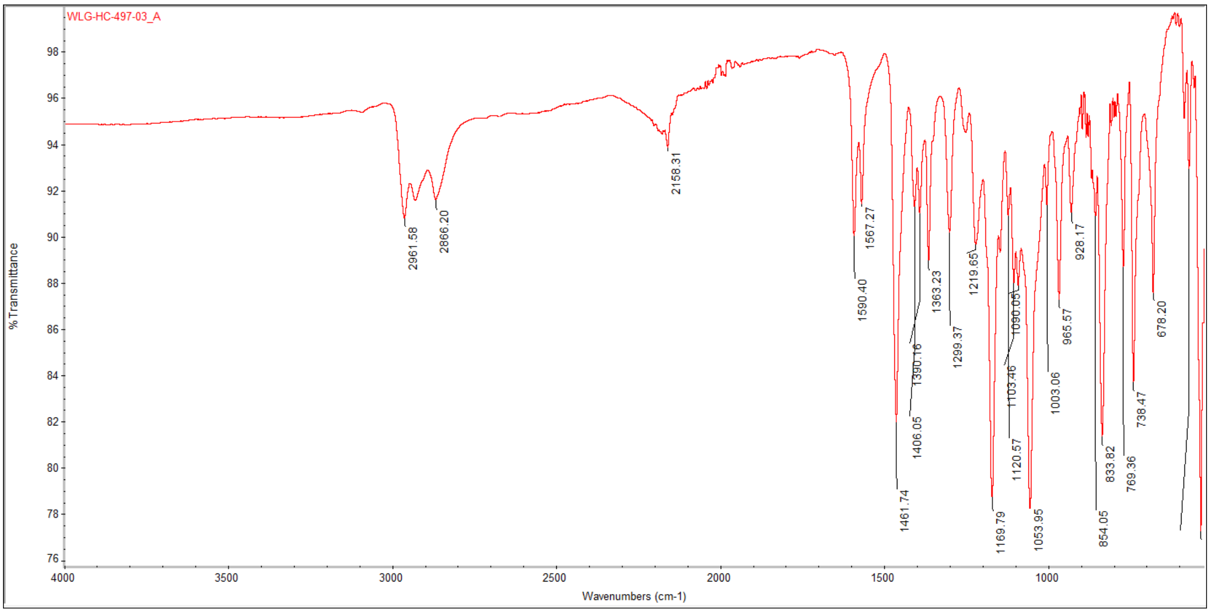

Figure S7: IR spectrum of **10**.

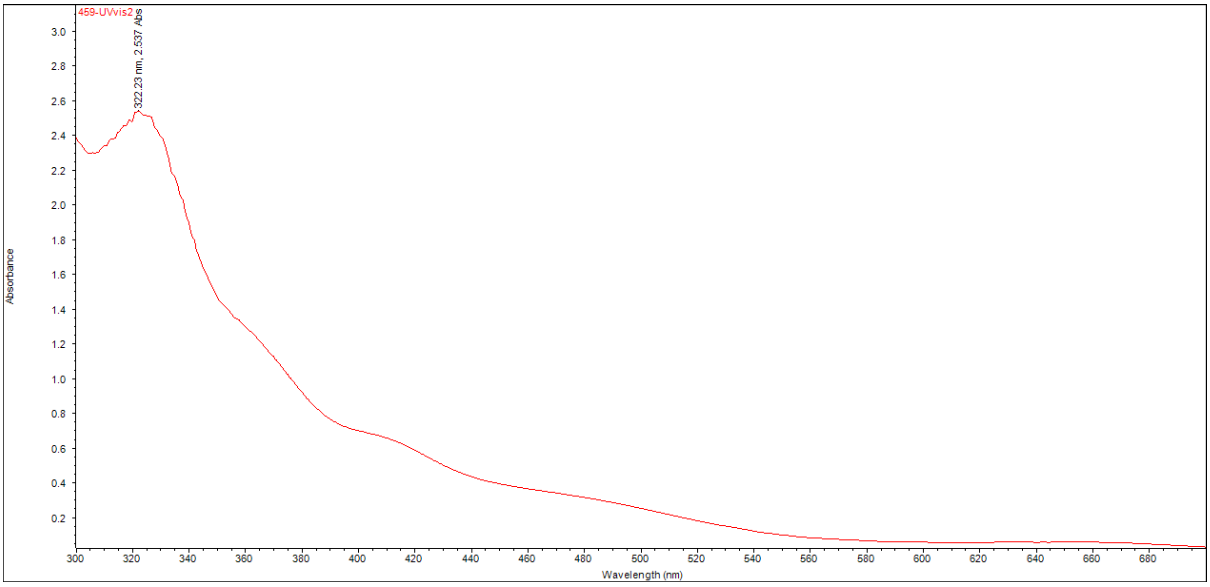

Figure S8: UV-vis spectrum of **10** (1 µM in dichloromethane).

2.3.3. Synthesis of 5

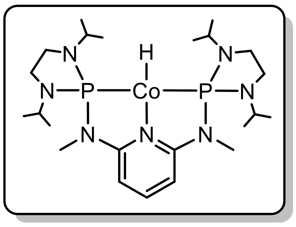

A solution of $NaBEt_3H$ (69 µl, 0.069 mmol, 1M in toluene) was added to a solution of complex **10** (20.0 mg, 0.0327 mmol) in toluene (5 ml). The mixture was stirred for 10 min at room temperature and filtered through a syringe filter. After removing the solvent *in vacuo*, the residue was washed with pentane (5 ml) and dried under reduced pressure. The product **5** was isolated as a dark green solid (6.5 mg, 0.012 mmol, 37%).

^{1}H NMR (400 MHz, C_6D_6, 298 K): δ -13.63 (t, $^2J_{P,H}$ = 58 Hz, Co-***H***, 1 H), 0.99 (d, $^3J_{H,H}$ = 6.7 Hz, 12 H, ^{i}Pr-C***H***$_3$), 1.29 (d, $^3J_{H,H}$ = 6.7 Hz, 12 H, ^{i}Pr-C***H***$_3$), 2.65 (s, 6 H, N-C***H***$_3$), 2.90-2.98 (m, 4 H, C***H***$_2$), 3.04-3.14 (m, 4 H, C***H***$_2$), 4.62 (sept, $^3J_{H,H}$ = 6.5 Hz, 4 H, ^{i}Pr-C***H***), 5.72 (d, $^3J_{H,H}$ = 8.3 Hz, 2 H, Ar-*m*-C***H***), 7.11-7.13 (d, $^3J_{H,H}$ = 8.5 Hz, 1 H, Ar-*p*-C***H***).

^{13}C{^{1}H} NMR (101 MHz, C_6D_6, 298 K): δ 19.8 (^{i}Pr-***C***H$_3$), 21.0 (^{i}Pr-***C***H$_3$), 30.2 (N-***C***H$_3$), 39.8 (***C***H$_2$), 44.0 (t, $^2J_{P,C}$ = 8.3 Hz, ^{i}Pr-***C***H), 94.5 (t, $^3J_{P,C}$ = 2.7 Hz, Ar-*m*-***C***H), 134.0 (m, Ar-*p*-***C***H), 159.0 (t, $^2J_{P,C}$ = 12.7 Hz, Ar-*o*-***C***).

Several ^{13}C NMR resonances appear as triplets due to a large $^2J_{P,P}$ coupling of the two phosphorus atoms in *trans* configuration (virtual coupling).[7]

^{31}P{^{1}H} NMR (162 MHz, C_6D_6, 298 K): δ 181.2 (s).

HR-MS (ESI$^+$): m/z: calcd. for $[C_{23}H_{46}N_7P_2Co]^+$: 541.26164; found: 541.26113.

IR (Diamond ATR cell, cm^{-1}): 2958, 2922, 2862, 1745, 1579, 1563, 1465, 1409, 1396, 1386, 1363, 1306, 1225, 1166, 1145, 1101, 1119, 1086, 1052, 1010, 966, 923, 861, 850, 813, 813, 766, 737, 718, 674, 584.

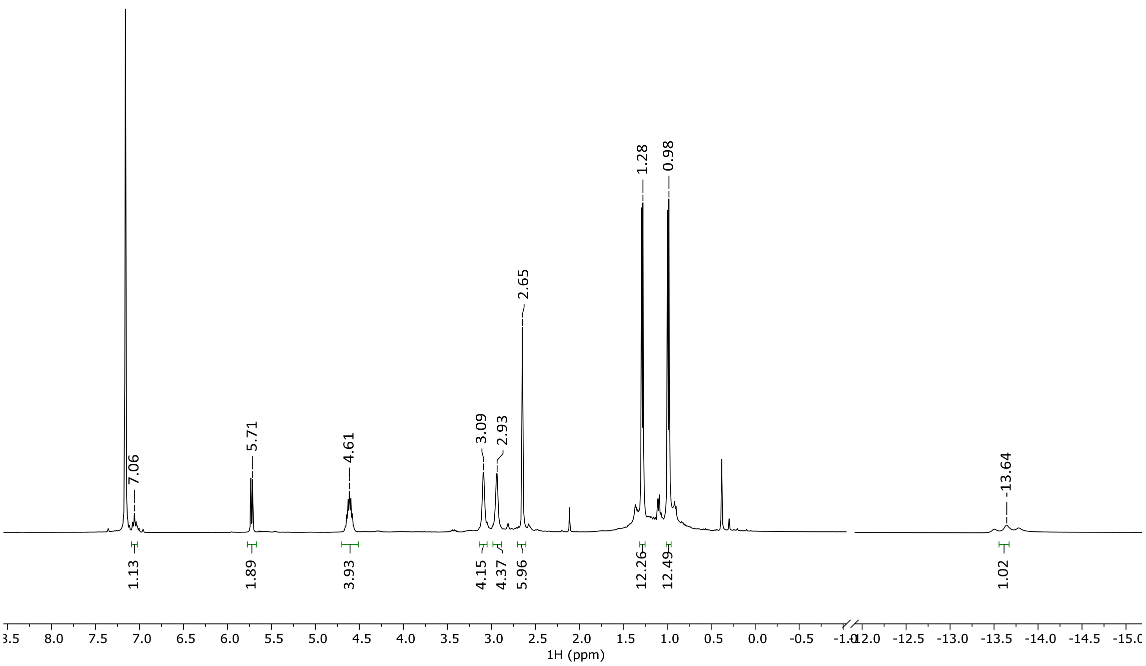

Figure S9: ^{1}H NMR (400 MHz, C_6D_6, 298 K) of **5**.

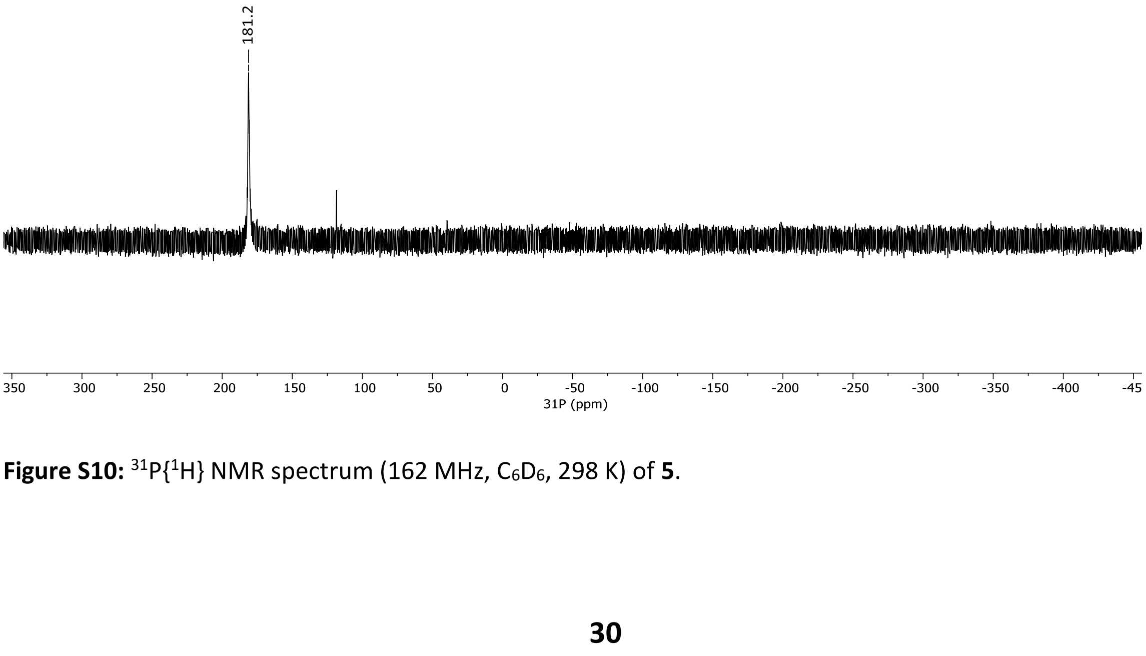

Figure S10: $^{31}P\{^{1}H\}$ NMR spectrum (162 MHz, C_6D_6, 298 K) of **5**.

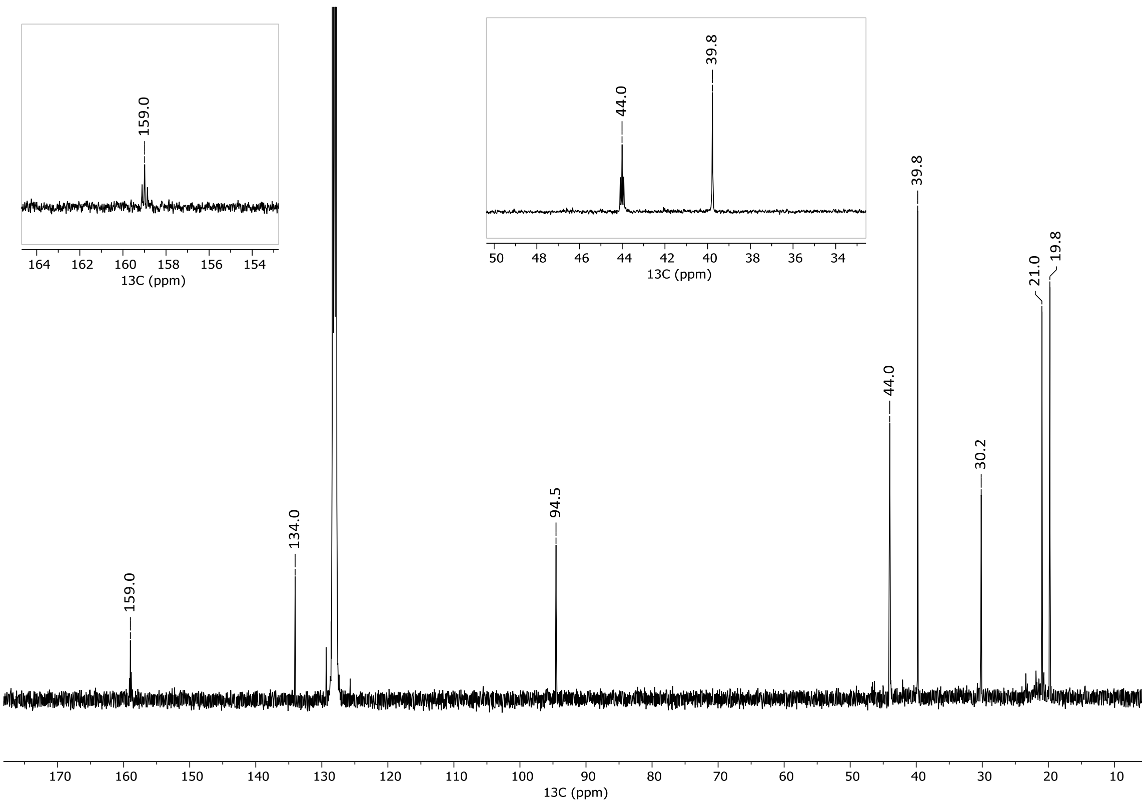

Figure S11: $^{13}C\{^{1}H\}$ NMR spectrum (101 MHz, 298 K, C_6D_6) of **5**.

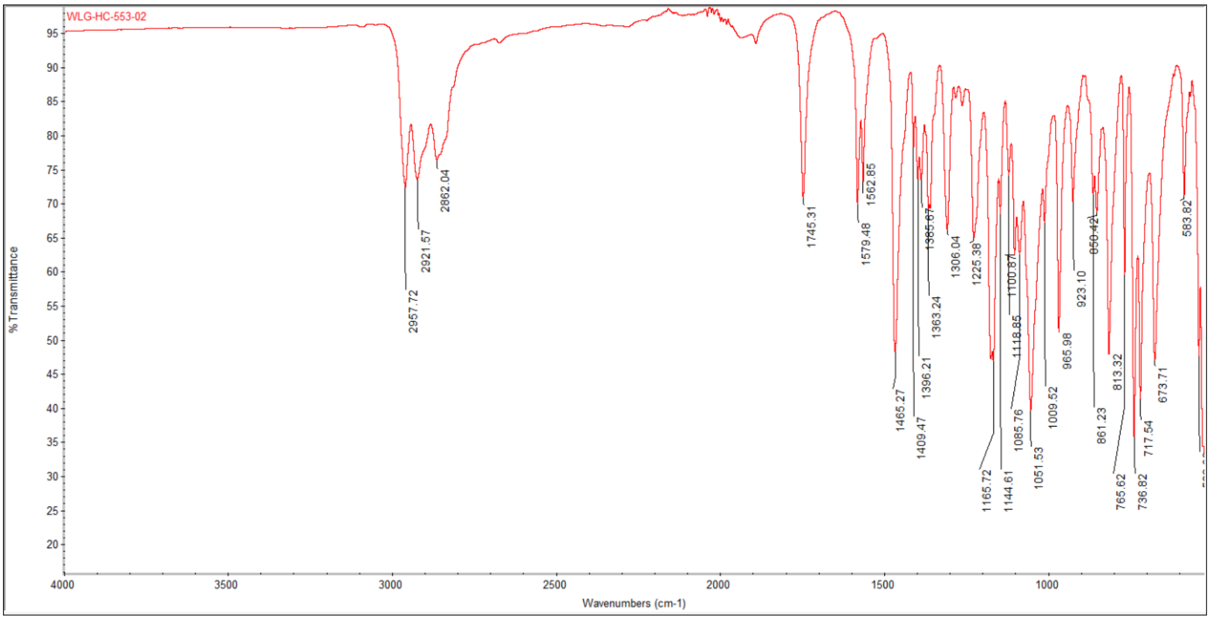

Figure S12: IR spectrum of **5**.

2.3.4. Synthesis of 13

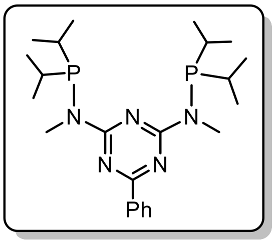

Lithium bis(trimethylsilyl)amide (894 mg, 5.34 mmol) and 2,6-bis(methylamino)-4-phenyltriazine **12** (500 mg, 2.32 mmol) were added to a solution of diisopropylchloro-phosphine (815 mg, 5.34 mmol) in toluene (10 ml). The mixture was stirred at room temperature for 12 h and subsequently filtered using a glass frit, and the volatiles were removed *in vacuo*. The product **13** was obtained as a clear oil (953 mg, 2.13 mmol, 92%) and used in the following reactions without further purification.

^{1}H NMR (400 MHz, toluene-d$_8$, 298 K): δ 0.86 (d, $^{3}J_{H,H}$ = 6.8 Hz, 6 H, ^{i}Pr-C***H***$_3$), 0.89 (d, $^{3}J_{H,H}$ = 7.0 Hz, 6 H, ^{i}Pr-C***H***$_3$), 0.95-1.07 (overlapping, 16 H, ^{i}Pr-C***H***$_3$, ^{i}Pr-C***H***), 3.20 (br., 6 H, N-C***H***$_3$), 7.11-7.21 (overlapping, 3 H, Ar-*m*-C***H***, Ar-*p*-C***H***), 8.70 (d, $^{3}J_{H,H}$ = 7.5 Hz, 2 H, Ar-*o*-C***H***).

^{13}C{^{1}H} NMR (101 MHz, CD_2Cl_2, 298 K): δ 20.0 (^{i}Pr-***C***H$_3$), 20.1 (^{i}Pr-***C***H$_3$), 20.2 (^{i}Pr-***C***H$_3$), 20.3 (^{i}Pr-***C***H$_3$), 26.6 (br., 20.1 (^{i}Pr-***C***H), 37.6 (N-***C***H$_3$), 128.1 (Ar-*o*-***C***H), 129.0 (Ar-*m*-***C***H), 131.7 (Ar-*p*-***C***H), 138.3 (Ar-*i*-***C***), 169.0 (br., Triazine-*o*-***C***), 171.0 (br., Triazine-*p*-***C***).

^{31}P{^{1}H} NMR (162 MHz, $CDCl_3$, 298 K): 92.60 (s).

HR-MS (ESI^{+}): m/z: calcd. for $[C_{23}H_{40}N_5P_2]^+$: 448.27535; found: 448.27548.

IR (Diamond ATR cell, cm^{-1}): 2947, 2863, 1589, 1448, 1432, 1305, 1239, 1173, 1097, 1009, 979, 921, 875, 828, 783, 7698, 751, 728, 703, 685, 652, 618.

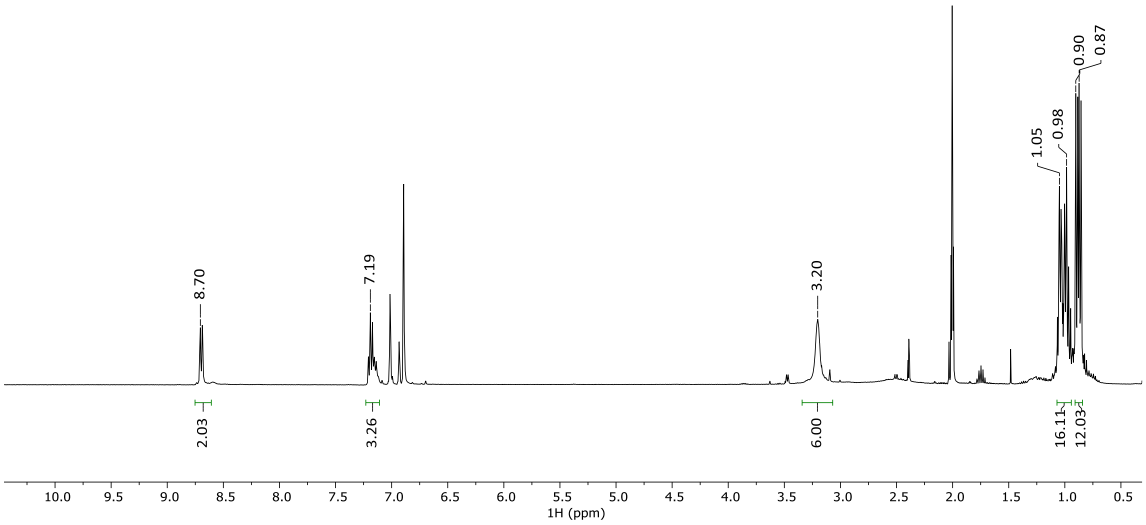

Figure S13: ^{1}H NMR spectrum (toluene-d_8, 400 MHz, 298 K) of **13**.

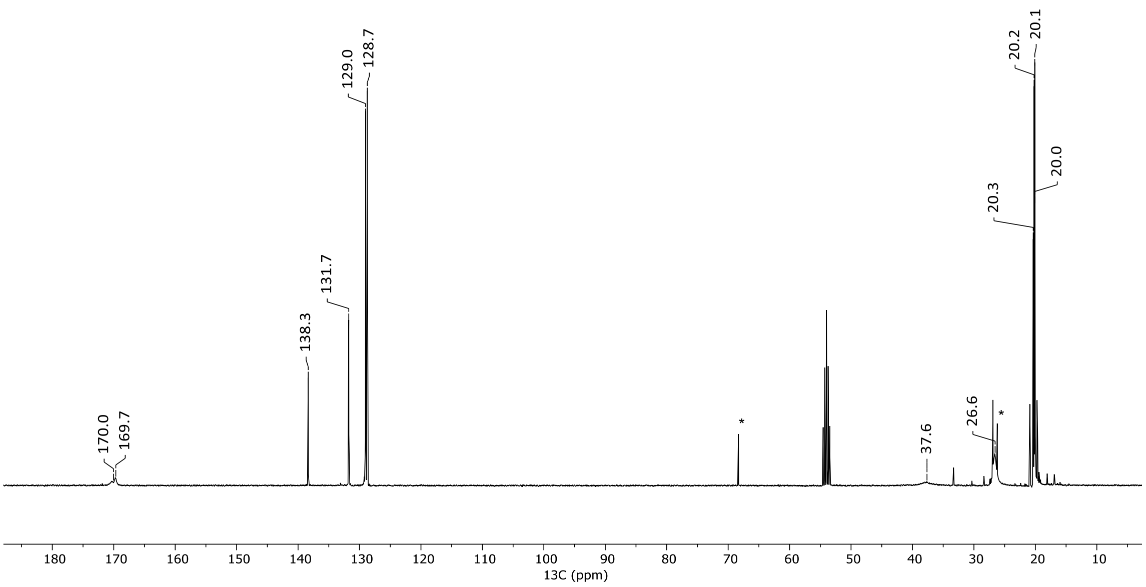

Figure S14: $^{13}C\{^{1}H\}$ NMR spectrum (CD_2Cl_2, 101 MHz, 298 K) of **13** (* - THF).

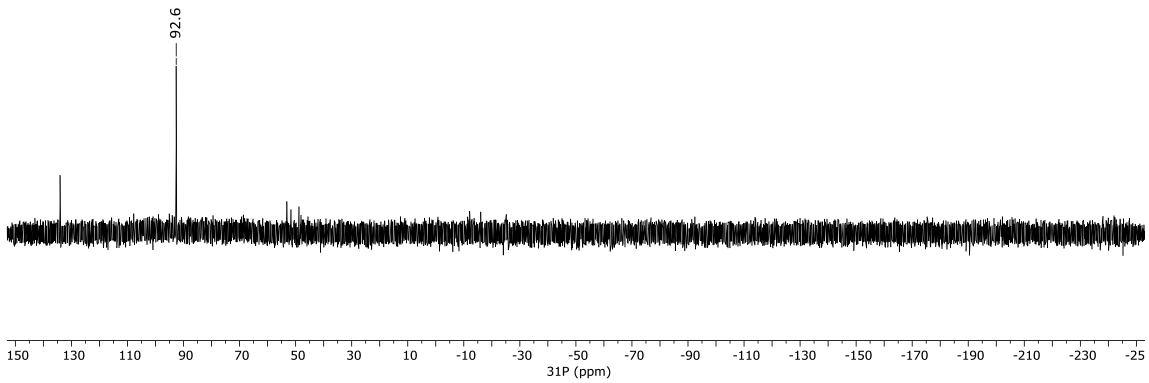

Figure S15: $^{31}P\{^{1}H\}$ NMR spectrum ($CDCl_3$, 162 MHz, 298 K) of **13**.

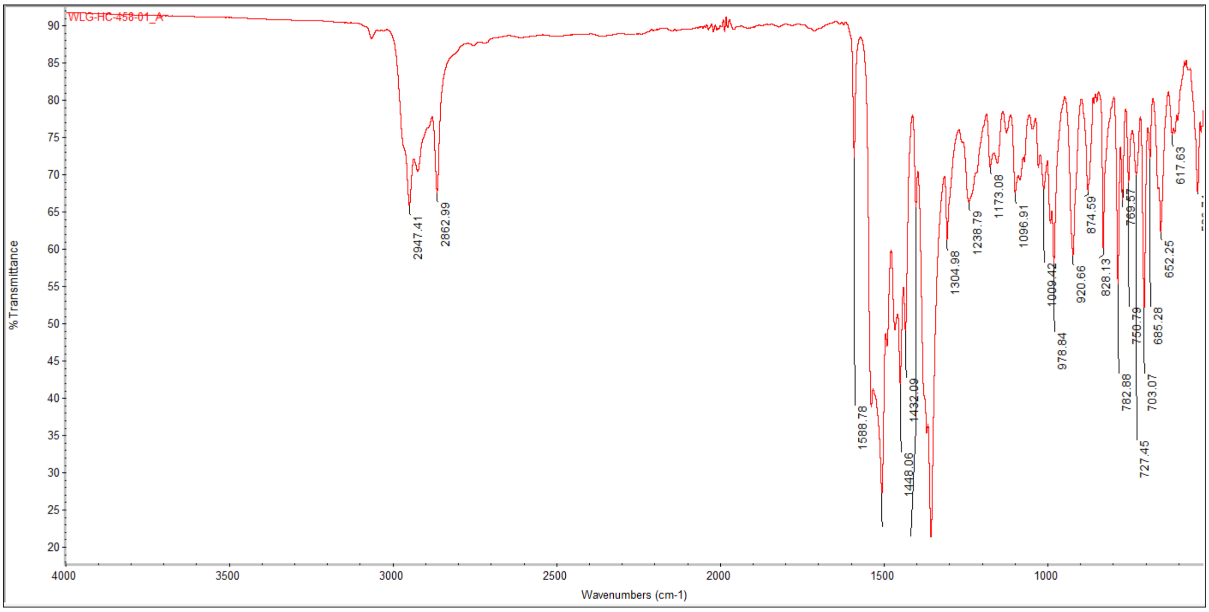

Figure S16: IR spectrum of **13**.

2.3.5. Synthesis of 14

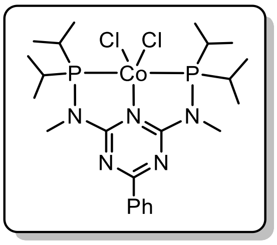

A solution of **13** (100 mg, 0.223 mmol, 10% in THF) was added to a suspension of $CoCl_2$ (29.1 mg, 0.223 mmol) in THF (10 ml) and stirred at room temperature for 12 h before filtration over Celite. After solvent removal *in vacuo*, and the residue was washed with pentane (3 x 3 ml) and dichloromethane (1 x 3 ml), giving the product **14** as dark purple solid (77 mg, 0.13 mmol, 60%).

The resonances in the ^{1}H, $^{13}C\{^{1}H\}$, and $^{31}P\{^{1}H\}$ NMR spectra could not be assigned due to the line broadening and wide shift range caused by the paramagnetism of the product.

HR-MS (ESI$^+$): m/z: calcd. for $[C_{23}H_{39}N_5P_2Cl_2]^+$: 576.13843; found: 576.13802.

Anal. calcd. (%) for $[C_{23}H_{39}Cl_2CoN_5P_2]$: C 47.85, 6.81, N 12.13; found: C 47.51, H 6.89, N 12.06.

IR (Diamond ATR cell, cm^{-1}): 2961, 2157, 1546, 1513, 1474, 1243, 1206, 1165, 1091, 1027, 998, 926, 878, 866, 849, 818, 789, 777, 757, 707, 676, 655, 636, 619, 609, 551.

UV/vis (1 µM in dichloromethane): 459.29 nm.

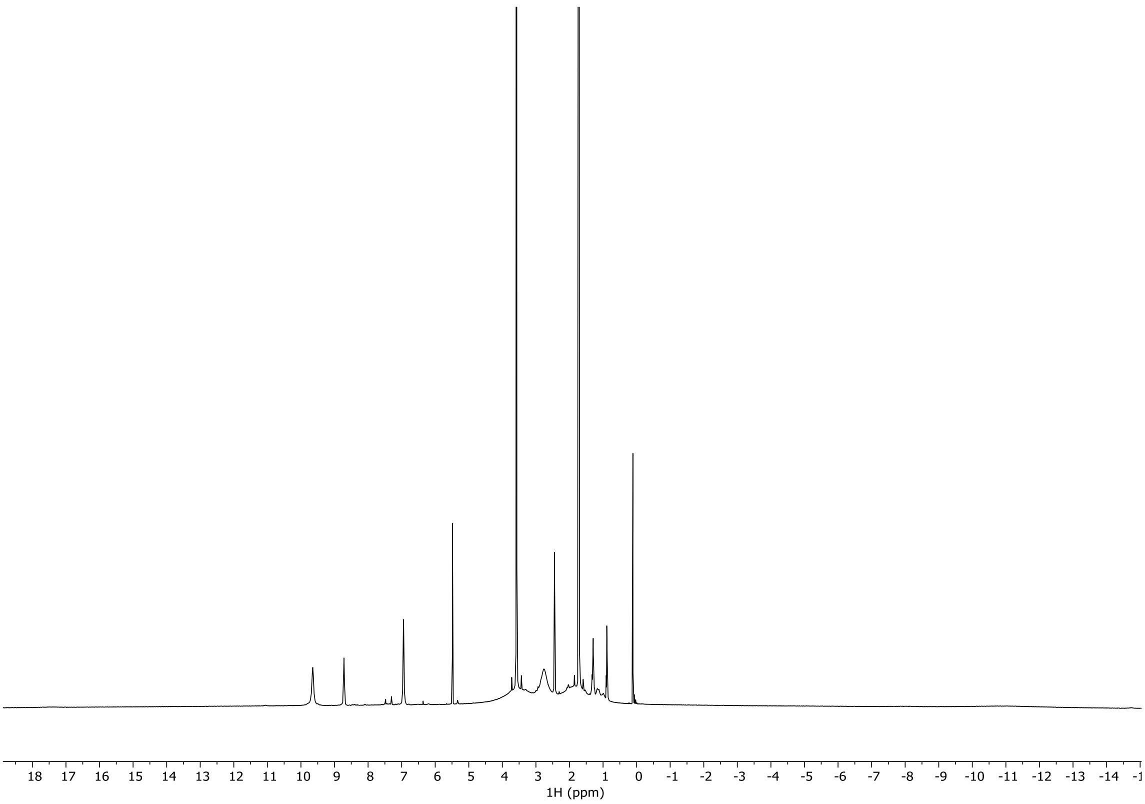

Figure S17: ^{1}H NMR spectrum (400 MHz, C_6D_6, 298 K) of **14**.

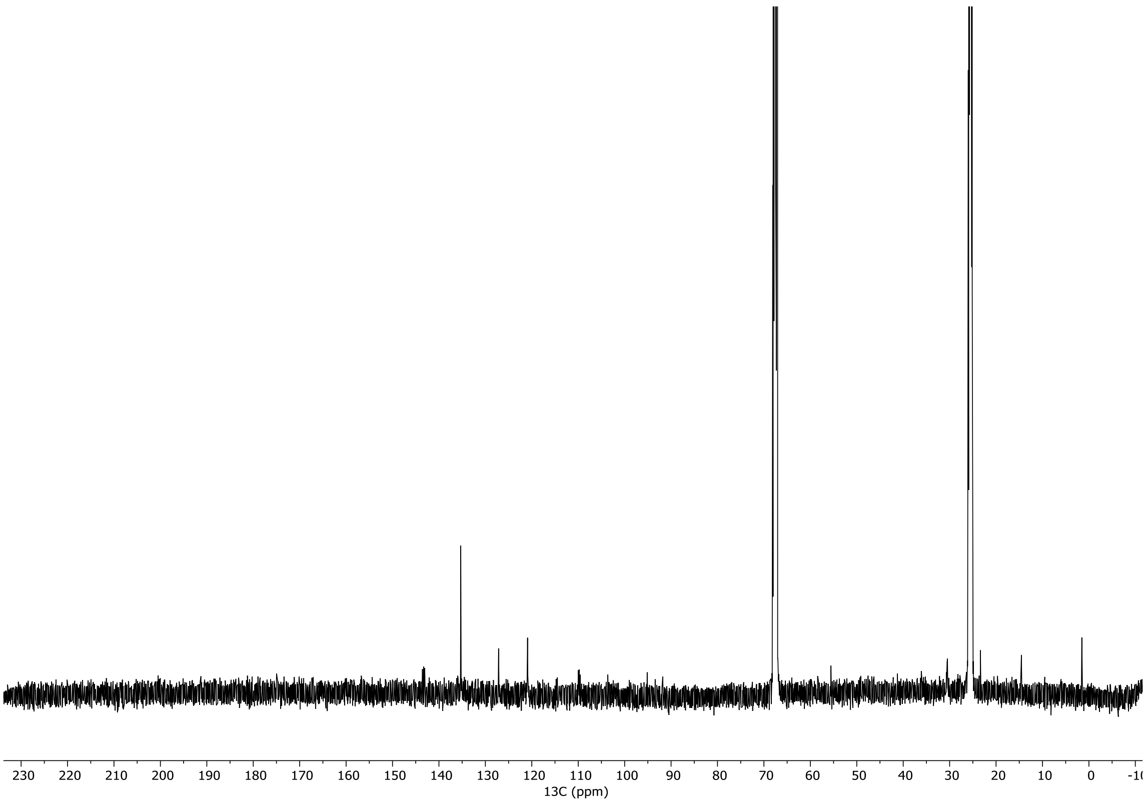

Figure S18: $^{13}C\{^{1}H\}$ NMR spectrum (126 MHz, THF-d_8, 298 K) of **14**.

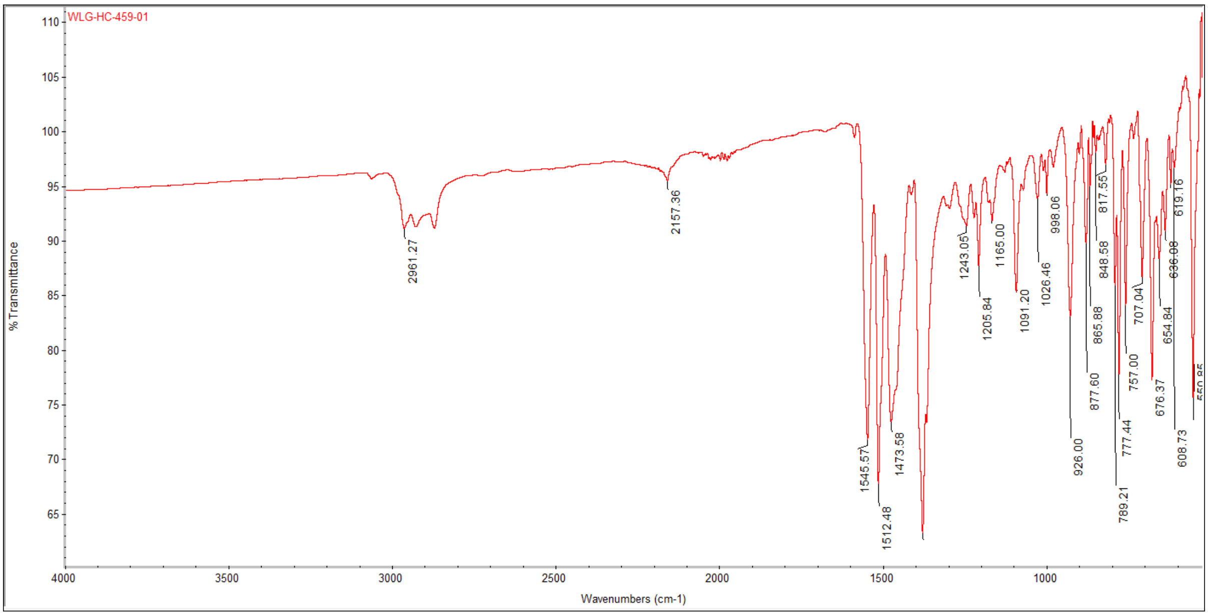

Figure S19: IR spectrum of **14**.

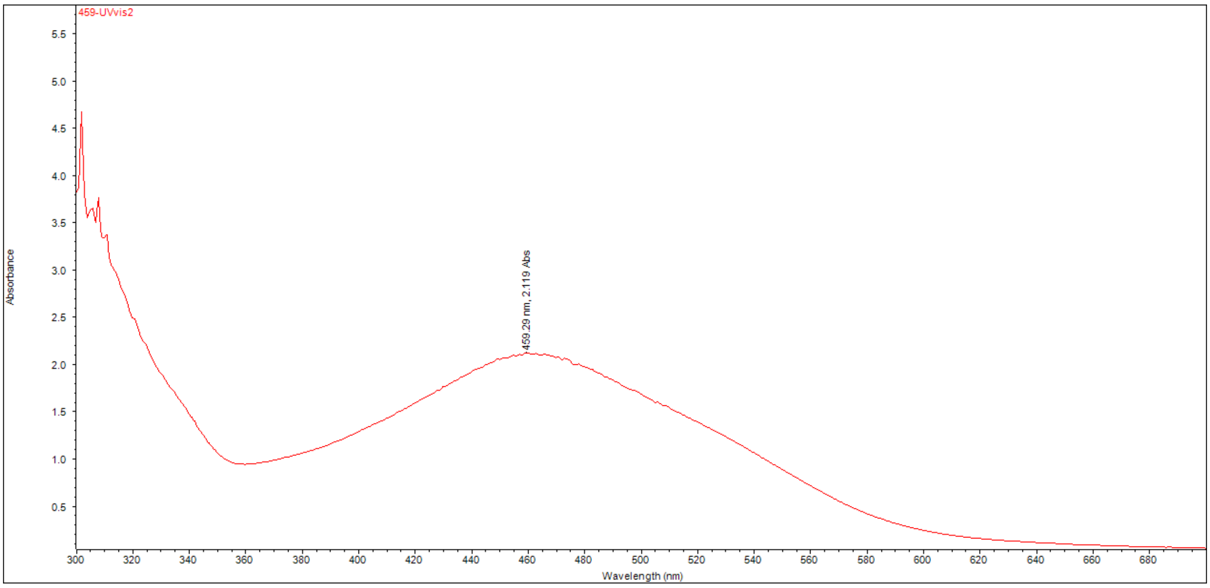

Figure S20: UV-vis spectrum of **14** (1 µM in dichloromethane).

2.3.6. Synthesis of 6

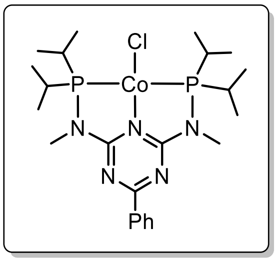

A solution of $NaBEt_3H$ (90 µl, 0.090 mmol, 1M in toluene) was added to a solution of complex **14** (50.0 mg, 0.086 mmol) in toluene (6 ml). The mixture was stirred at room temperature for 10 min and subsequently filtered using a syringe filter. Subsequently, the volatiles was removed *in vacuo* and the residue was washed with pentane (5 ml). The solvent was removed under reduced pressure and the product **6** was isolated as a dark green solid (6.5 mg, 0.012 mmol, 37%).

^{1}H NMR (400 MHz, C_6D_6, 298 K): 1.26 (dd, $^3J_{H,H}$ = 7.9 Hz, $^3J_{P,H}$ = 7.4 Hz, 12 H, ^{i}Pr-C***H***$_3$), 1.57 (q, $^3J_{H,H}$ = 7.9 Hz, $^3J_{P,H}$ = 7.4 Hz, 12 H, ^{i}Pr-C***H***$_3$), 2.54 (sept, $^3J_{H,H}$ = 6.9 Hz, 4 H, ^{i}Pr-C***H***), 2.92 (s, 6 H, N-C***H***$_3$), 7.20 (t, $^3J_{H,H}$ = 7.3 Hz, 2 H, *m*-Ph-C***H***), 7.68 (t, $^3J_{H,H}$ = 7.3 Hz, 1 H, *p*-Ph-C***H***), 8.93 (d, $^3J_{H,H}$ = 7.8 Hz, 2 H, *o*-Ph-C***H***).

^{13}C{^{1}H} NMR (101 MHz, C_6D_6, 298 K): 18.2 (t, $^2J_{P,C}$ = 3.2 Hz, ^{i}Pr-***C***H$_3$), 18.5 (^{i}Pr-***C***H$_3$), 25.2 (t, $^1J_{P,C}$ = 8.3 Hz, ^{i}Pr-***C***H), 31.5 (N-***C***H$_3$), 126.2 (*o*-Ph-***C***H), 130.1 (*m*-Ph-***C***H), 141.8 (*i*-Ph-***C***), 150.2 (*p*-Triazine-***C***), 168.4 (t, $^2J_{P,C}$ = 15.3 Hz, *o*-Triazine-***C***).

A signal corresponding to the *m*-Ph-***C***H resonance is not visible and might be overlapping with the solvent residual signal of C_6H_6.

^{31}P{^{1}H} NMR (162 MHz, C_6D_6, 298 K): 109.9 (s).

HR-MS (ESI$^+$): m/z: calcd. for $[C_{23}H_{39}N_5P_2]^+$: 541.16957; found: 541.17008.

IR (Diamond ATR cell, cm^{-1}): 2953, 2922, 2867, 1595, 1538, 1469, 1425, 1379, 1361, 1242, 1200, 1156, 1120, 1097, 1025, 1009, 970, 926, 878, 786, 755, 693, 671, 640, 619, 602.

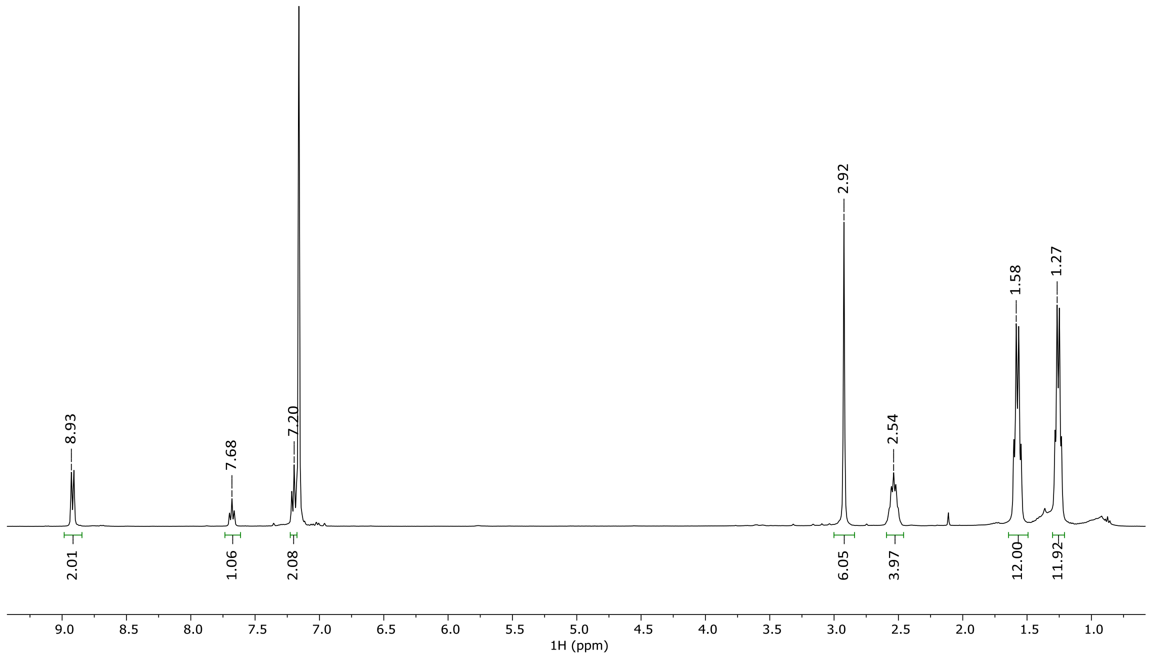

Figure S21: ^{1}H NMR spectrum (400 MHz, C_6D_6, 298 K) of **6**.

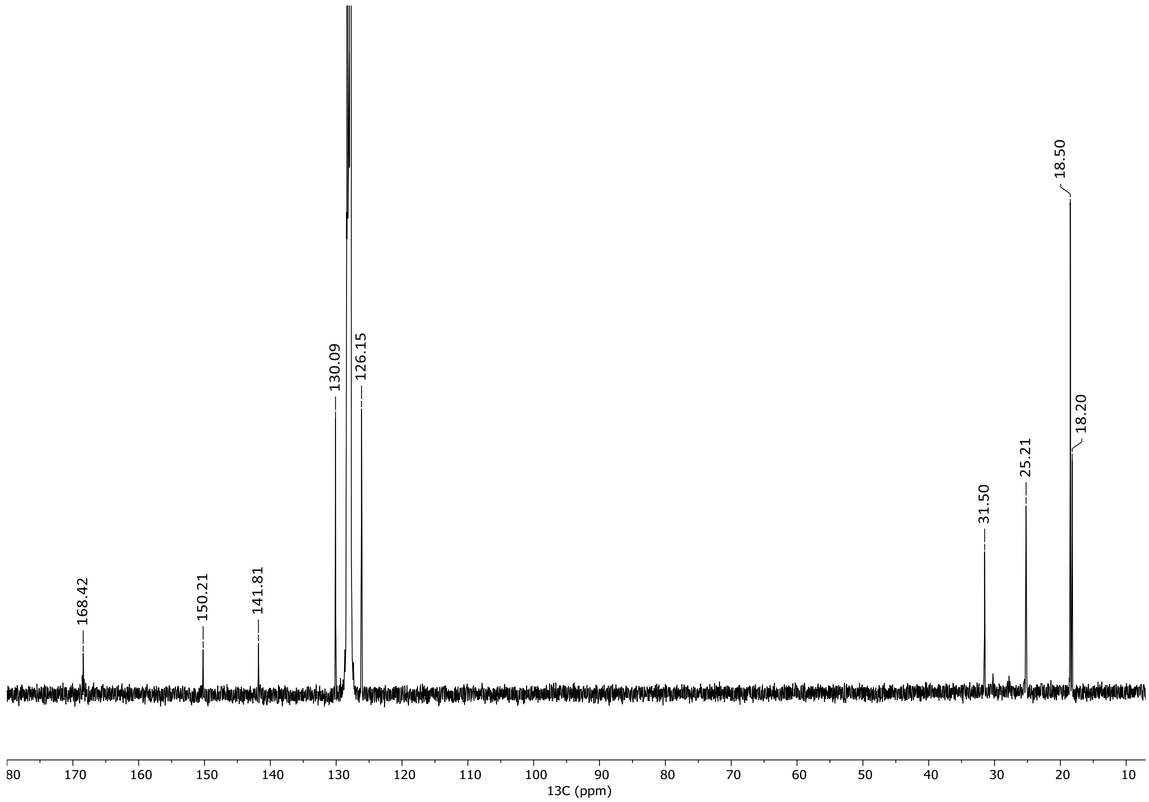

Figure S22: $^{13}C\{^{1}H\}$ NMR spectrum (101 MHz, C_6D_6, 298 K) of **6**.

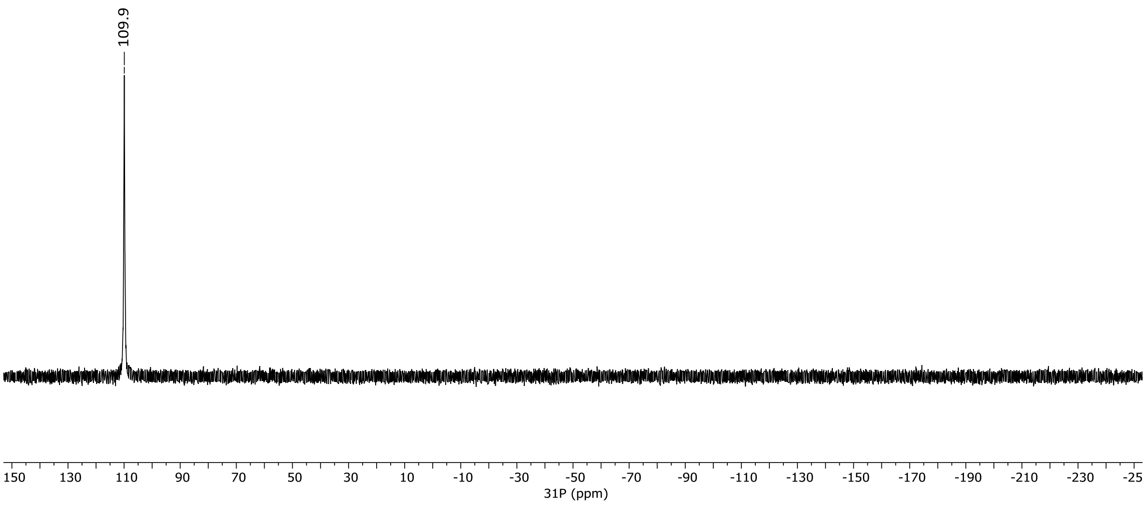

Figure S23: $^{31}P\{^{1}H\}$ NMR spectrum (162 MHz, C_6D_6, 298 K) of **6**.

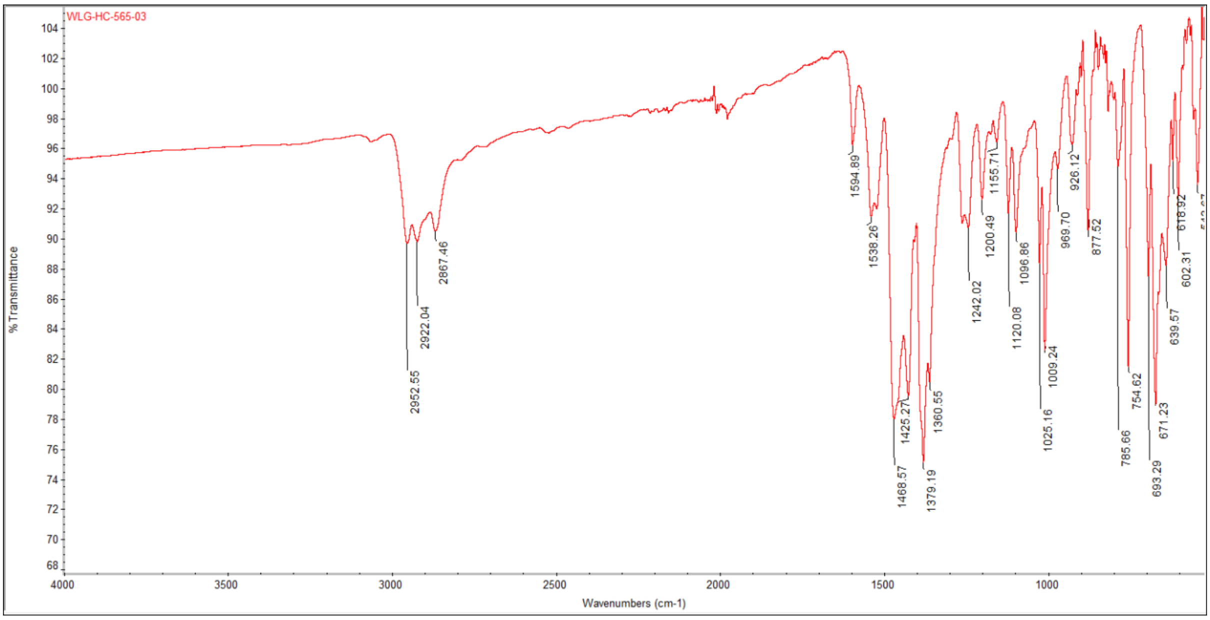

Figure S24: IR spectrum of **6**.

2.3.7. Synthesis of 16

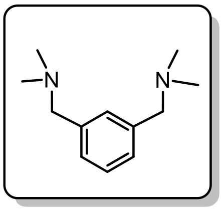

This compound was prepared following modified literature procedures.[8]

A solution of dimethylamine (8.54 ml, 75.7 mmol, 40% in water) was added to α,α'-dibromo-xylene (2.00 g, 7.58 mmol) dissolved in dichloromethane (40 ml) at 0°C. The mixture was allowed to warm to room temperature and stirred for 12 h. Water (10 ml) was added, and the product was extracted with dichloromethane (3 x 10 ml). The organic phase was dried over magnesium sulfate and separated from the solid by filtration. The volatiles were removed under reduced pressure, giving the product **16** as colorless oil (1.198 g, 6.23 mmol, 82%).

^{1}H NMR (400 MHz, $CDCl_3$, 298 K): δ 2.26 (s, 12 H, C***H***$_3$), 3.46 (s, 4 H, C***H***$_2$), 7.21-7.31 (overlapping, 4 H, Ar-C***H***).

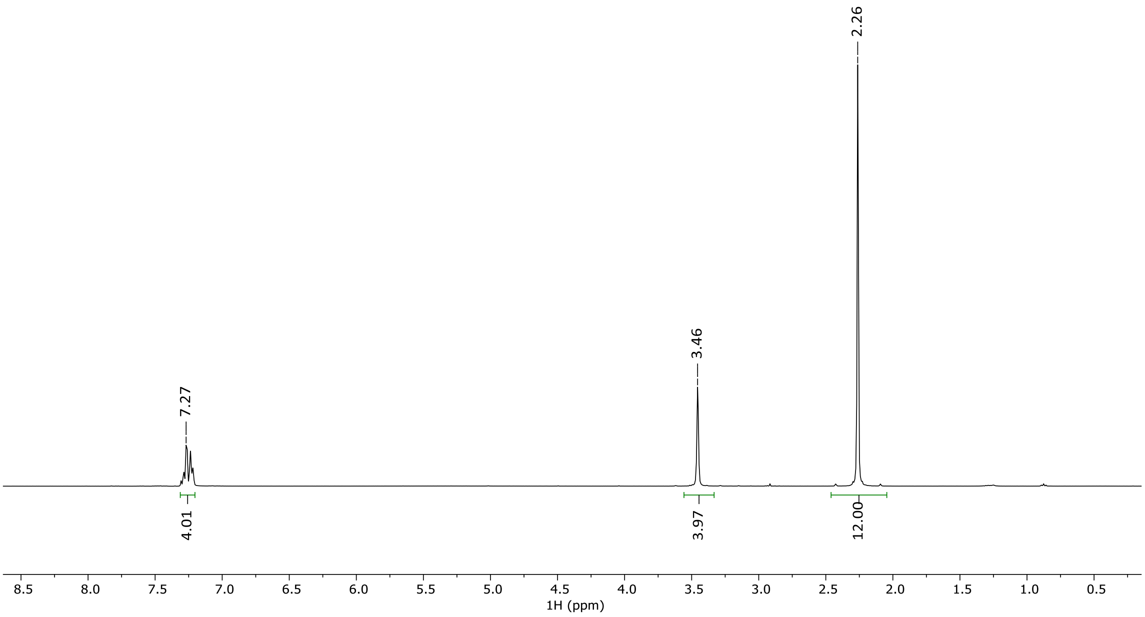

Figure S25: ^{1}H NMR spectrum (400 MHz, $CDCl_3$, 298 K) of **16**.

2.3.8. Synthesis of 4

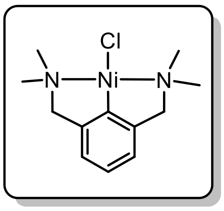

This compound was prepared following modified literature procedures.[9]

A solution of *n*-butyllithium (1.77 ml, 2.83 mmol, 1.6 M in hexane) was added dropwise to **16** (500 mg, 2.600 mmol) dissolved in heptane (5 ml) at -78°C. The mixture was stirred for 2 h while slowly warming to room temperature. The volatiles were removed *in vacuo* and the residue was dissolved in THF (5 ml). Subsequently, the solution was added to $NiCl_2$(dme) (687 mg, 3.15 mmol) dissolved in THF (5 ml) and the mixture was allowed to stir for 12 h. The volatiles were removed *in vacuo* and diethylether (10 ml) was added to the residue. After filtration over Celite, the solvent was removed *in vacuo* and the residue was washed with pentane (3 x 5 ml). The product **4** was isolated as yellow solid (117.7 mg, 0.414 mmol, 16%).

^{1}H NMR (400 MHz, CD_2Cl_2, 298 K): δ 2.64 (s, 12 H, C***H***3), 3.65 (s, 4 H, C***H***2), 6.59 (d, $^3J_{H,H}$ = 7.1 Hz, 2 H, Ph-*m*-C***H***), 6.92 (t, $^3J_{C,H}$ = 7.4 Hz, 2 H, Ph-*p*-C***H***).

^{13}C{^{1}H} NMR (101 MHz, CD_2Cl_2, 298 K): δ 51.6 (***C***H3), 74.1 (***C***H2), 118.9 (Ph-*m*-***C***H), 125.0 (Ph-*p*-***C***H), 147.5 (Ph-*o*-***C***).

A ^{13}C resonance corresponding to the Ph-*i*-***C*** carbon atom is not visible.

HRMS (ESI$^+$): m/z: calcd. for [$C_{12}H_{19}NiClN_2$]: [M]+ = 284.05836 (exp.), 284.05847 (theor.).

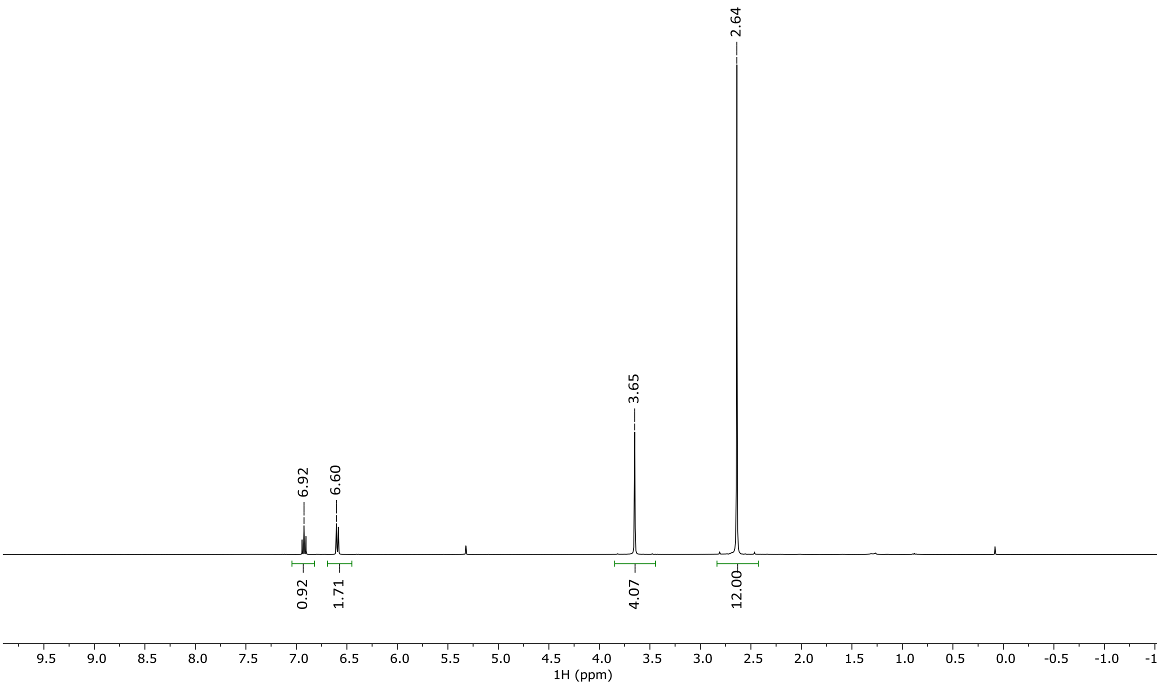

Figure S26: ^{1}H NMR spectrum (400 MHz, CD_2Cl_2, 298 K) of **4**.

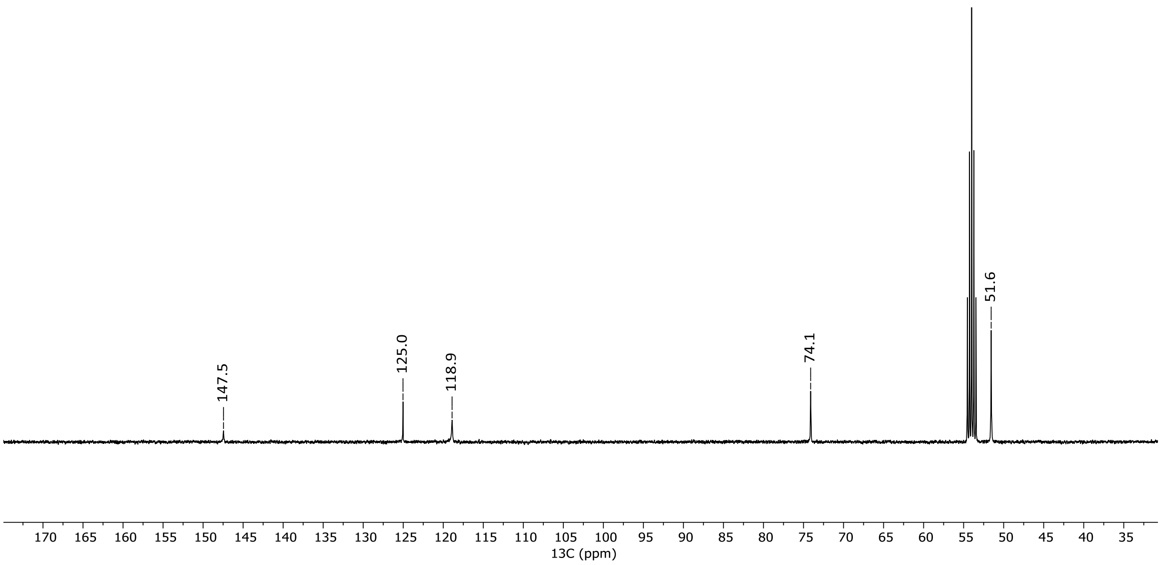

Figure S27: $^{13}C\{^{1}H\}$ NMR spectrum (101 MHz, 298 K, CD_2Cl_2) of **4**.

3. Hydrosilylation Experiments

In a flame-dried Schlenk tube, phenylsilane (2.5 mmol) was added to the respective catalyst (12.5 µmol), followed by the addition of $^{13}CO_2$ (1 bar, 0.5 mmol) by one freeze-pump-thaw cycle. The exact amount of $^{13}CO_2$ was determined by weighing the flask after evacuation and again after refilling and thawing. The reaction mixture was stirred at the required temperature for 2 h, and mesitylene (20 mg) was added as an internal standard after that time. C_6D_6 (0.5 ml) were added, the mixture was filtered through a syringe filter and transferred into an NMR tube. The amount of silylated products was determined by quantitative $^{13}C\{^1H\}$ NMR spectroscopy.[10]

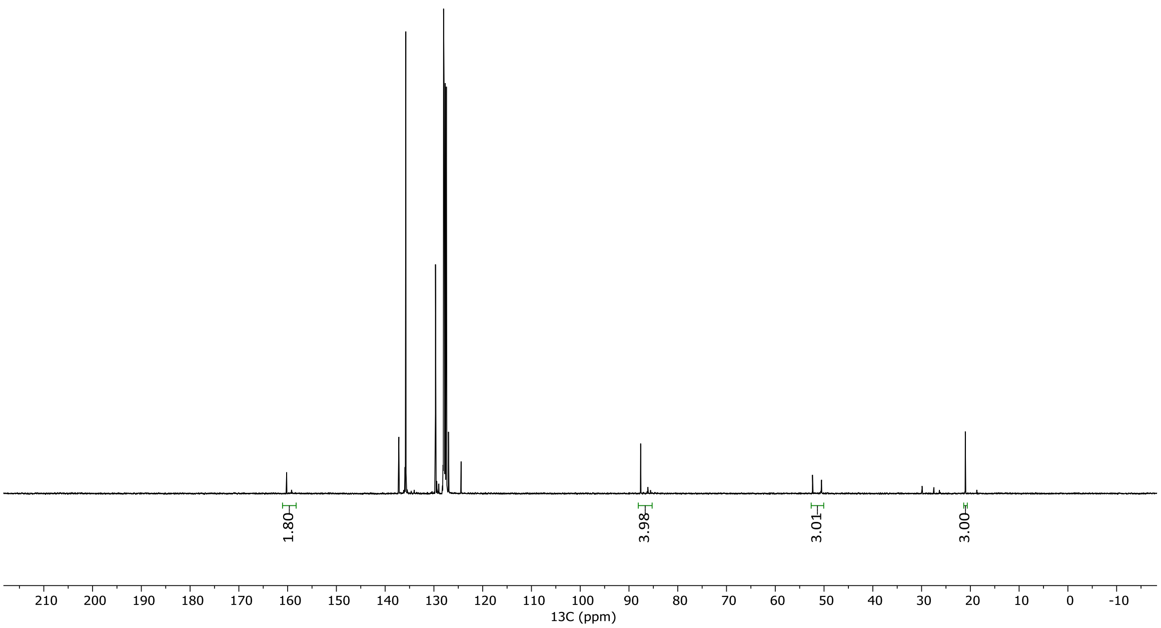

Figure S28: $^{13}C\{^{1}H\}$ NMR spectrum (101 MHz, C_6D_6, 298 K) of the reaction mixture resulting from the catalytic hydrosilylation of CO_2 using **5** at 25°C (Figure 7B).

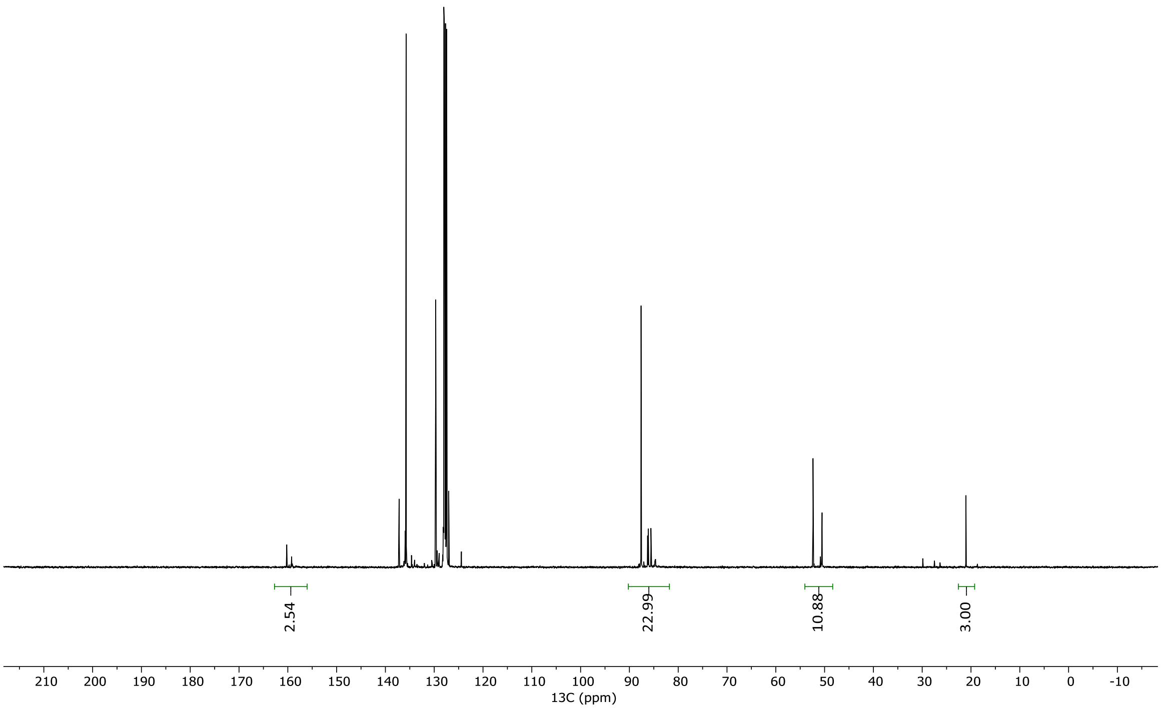

Figure S29: $^{13}C\{^{1}H\}$ NMR spectrum (101 MHz, C_6D_6, 298 K) of the reaction mixture resulting from the catalytic hydrosilylation of CO_2 using **5** at 40°C (Figure 7B).

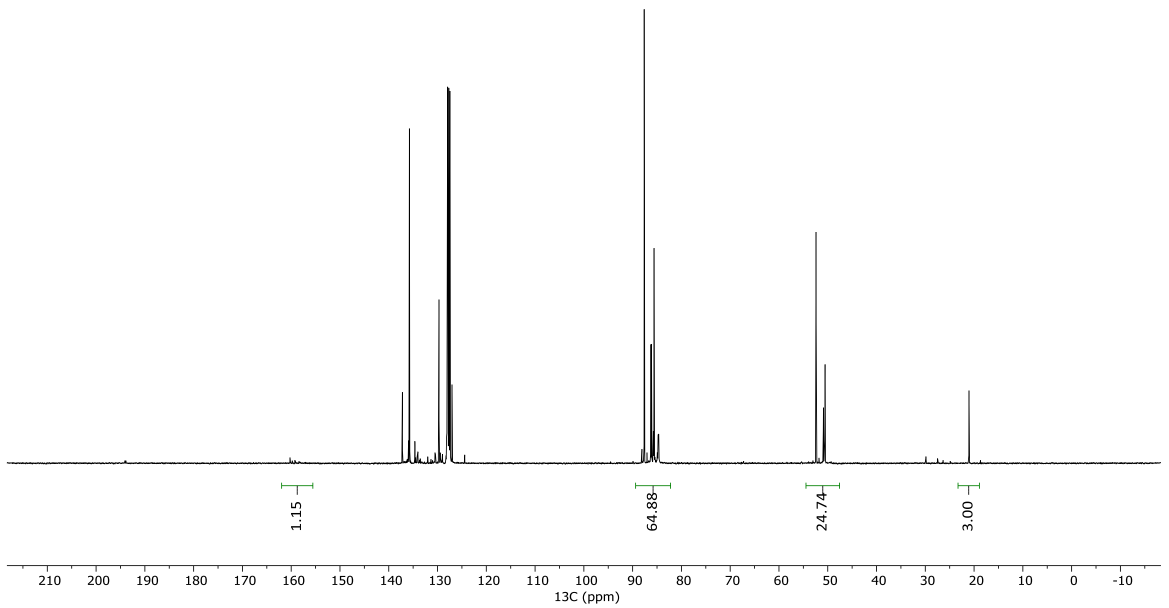

Figure S30: $^{13}C\{^1H\}$ NMR spectrum (101 MHz, C_6D_6, 298 K) of the reaction mixture resulting from the catalytic hydrosilylation of CO_2 using **5** at 60°C (Figure 7B).

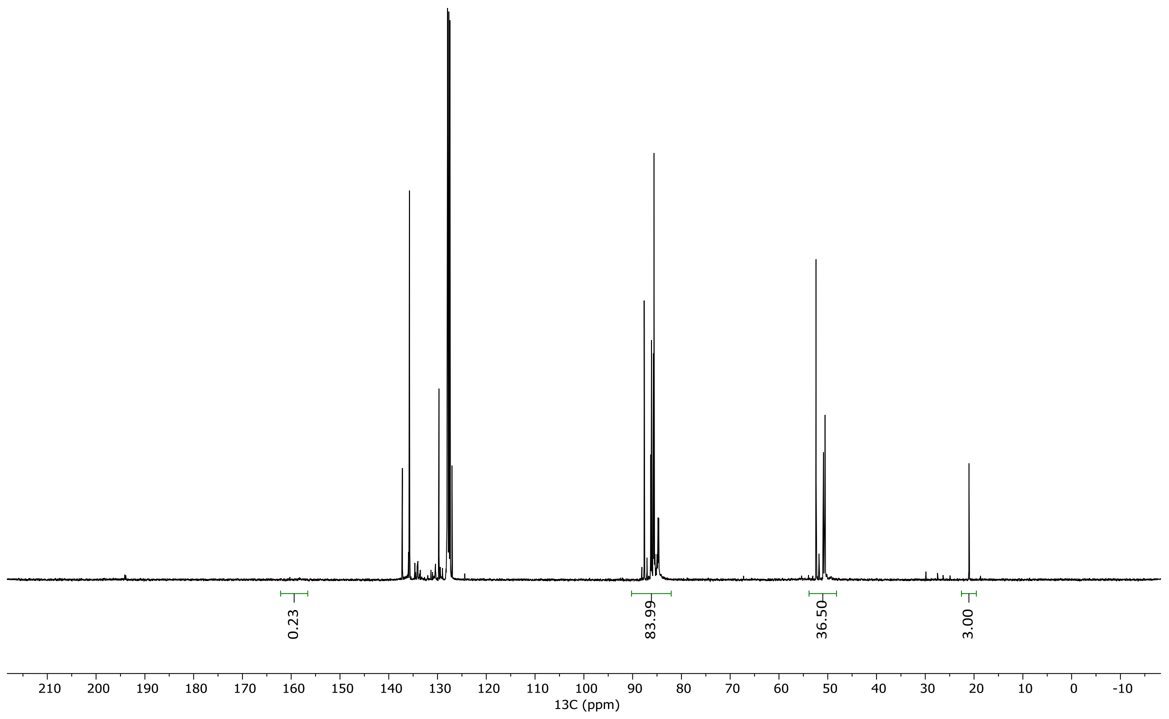

Figure S 31: $^{13}C\{^1H\}$ NMR spectrum (101 MHz, C_6D_6, 298 K) of the reaction mixture resulting from the catalytic hydrosilylation of CO_2 using **5** at 80°C (Figure 7B).

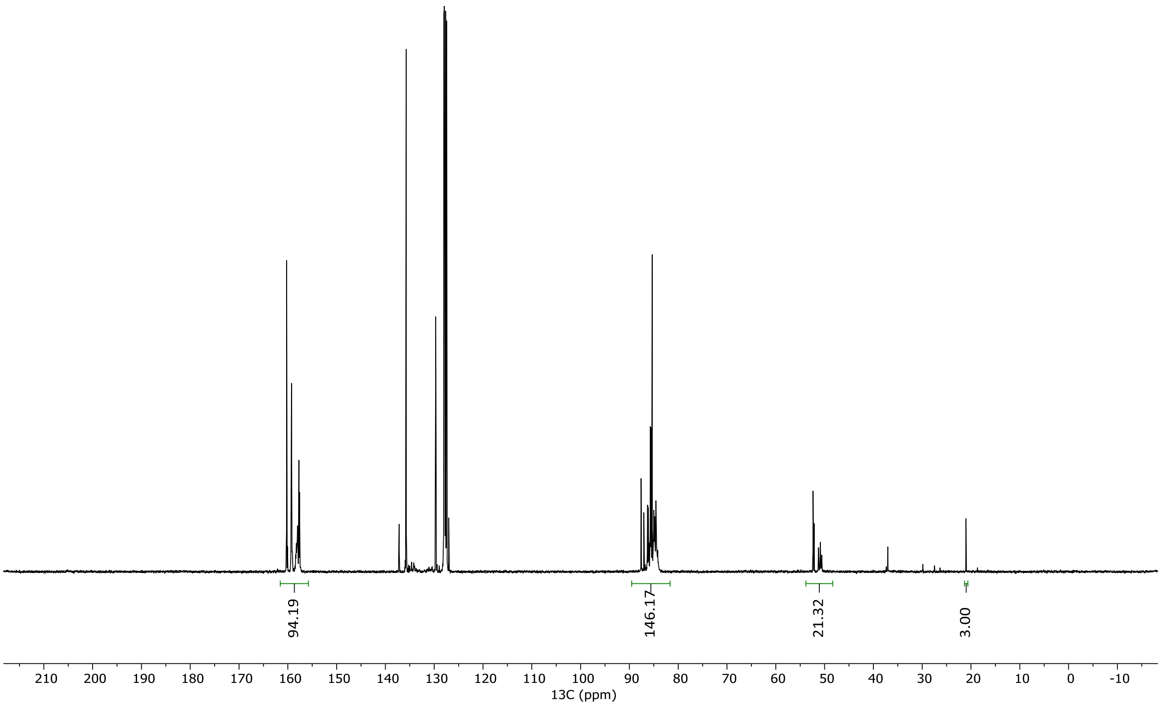

Figure S 32: $^{13}C\{^1H\}$ NMR spectrum (101 MHz, C_6D_6, 298 K) of the reaction mixture resulting from the catalytic hydrosilylation of CO_2 using **6** at 25°C (Figure 7B).

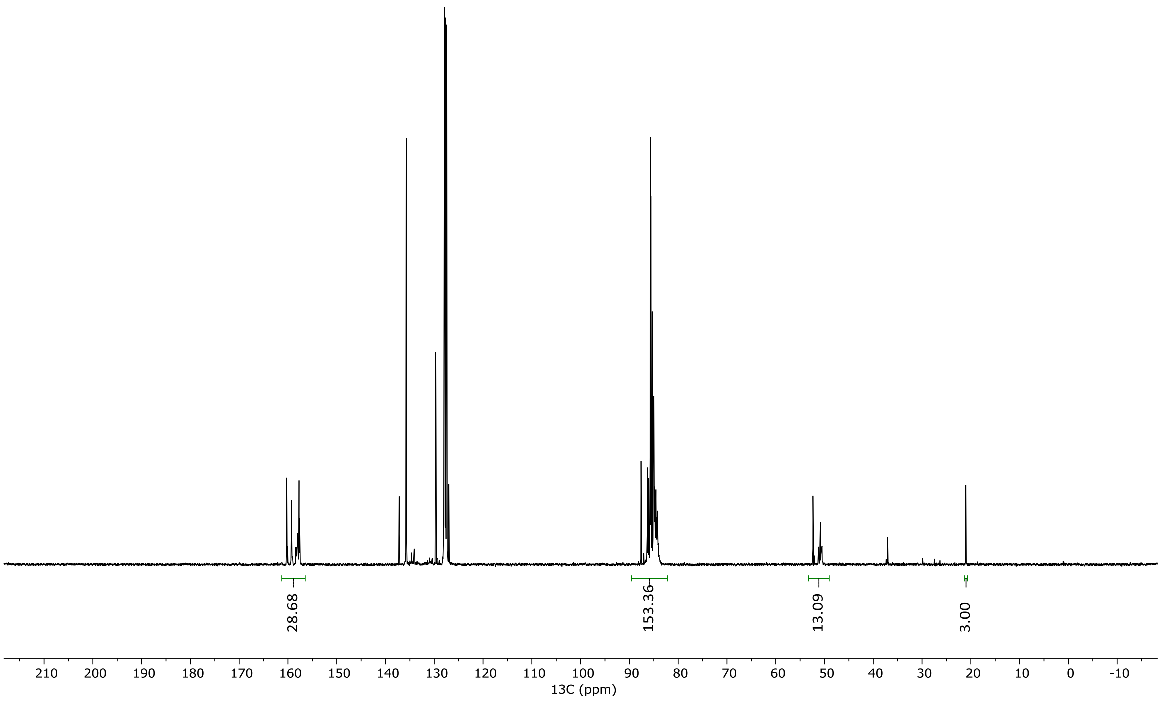

Figure S 33: $^{13}C\{^1H\}$ NMR spectrum (101 MHz, C_6D_6, 298 K) of the reaction mixture resulting from the catalytic hydrosilylation of CO_2 using **6** at 40°C (Figure 7B).

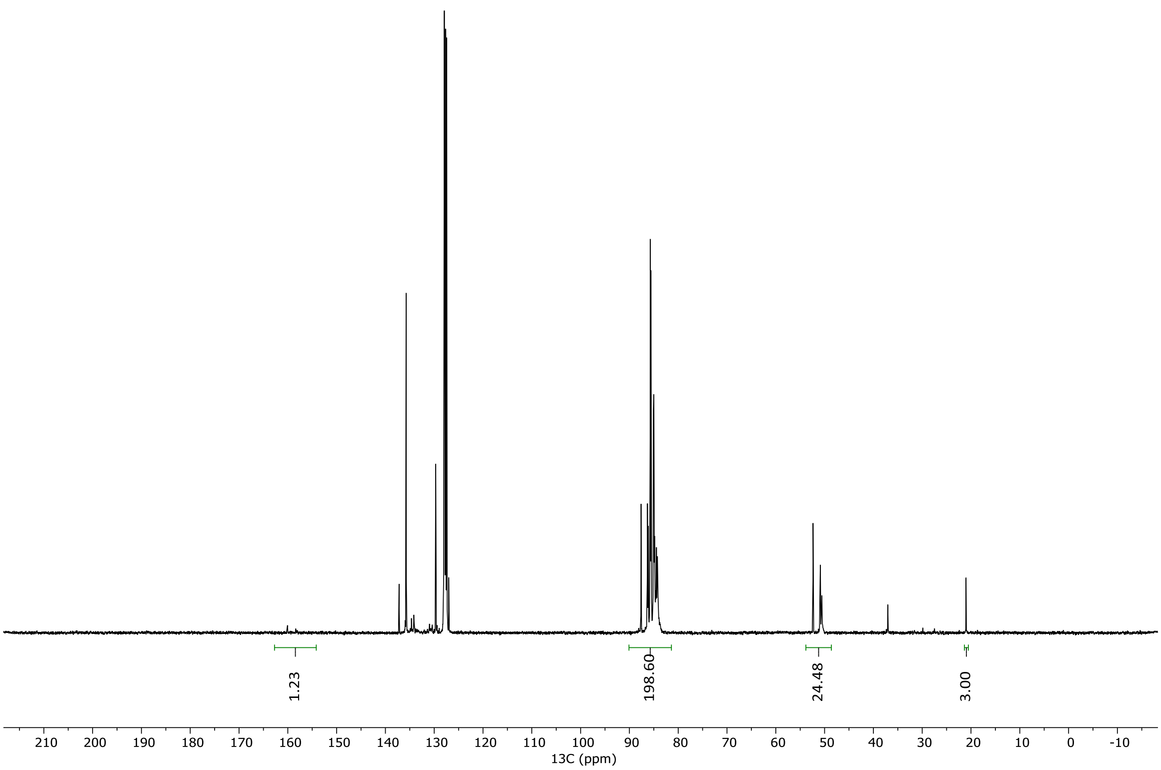

Figure S34: $^{13}C\{^1H\}$ NMR spectrum (101 MHz, C_6D_6, 298 K) of the reaction mixture resulting from the catalytic hydrosilylation of CO_2 using **6** at 60°C (Figure 7B).

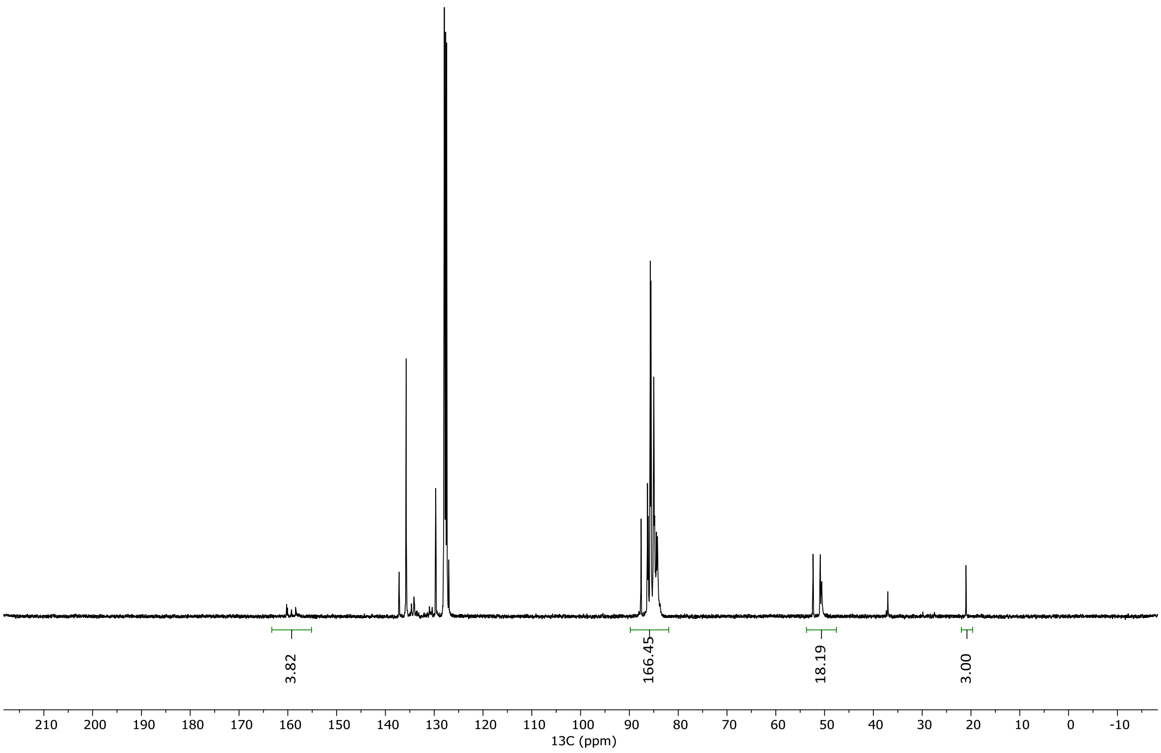

Figure S 35: $^{13}C\{^1H\}$ NMR spectrum (101 MHz, C_6D_6, 298 K) of the reaction mixture resulting from the catalytic hydrosilylation of CO_2 using **6** at 80°C (Figure 7B).

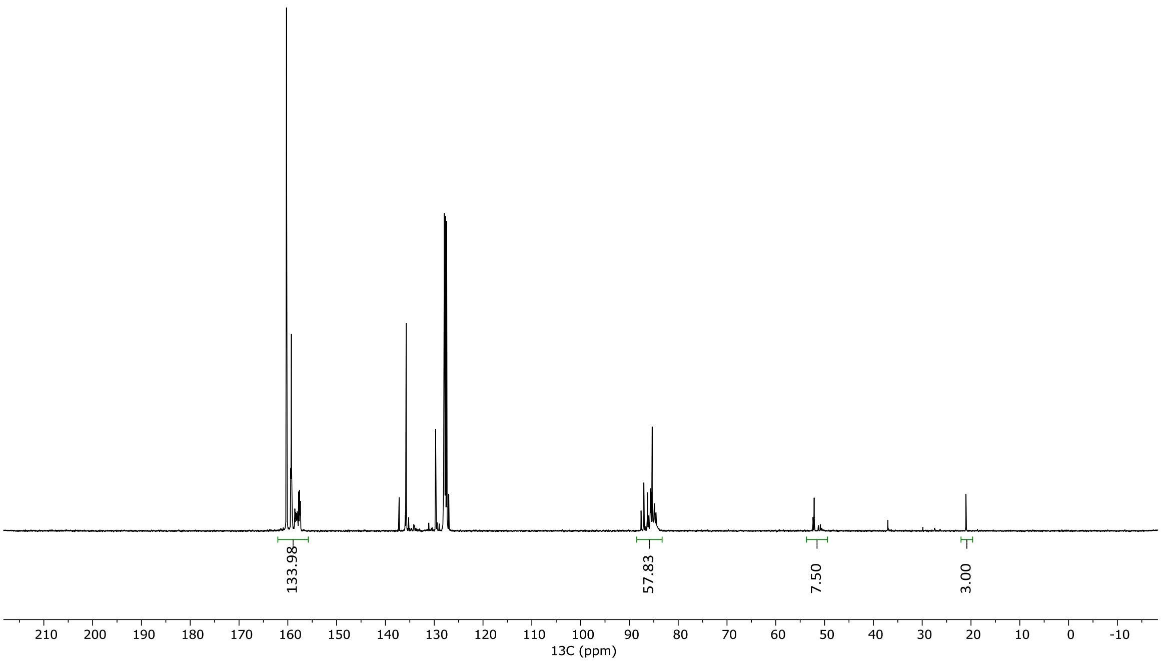

Figure S36: $^{13}C\{^{1}H\}$ NMR spectrum (101 MHz, C_6D_6, 298 K) of the reaction mixture resulting from the catalytic hydrosilylation of CO_2 using **4** at 25°C (Figure 7B).

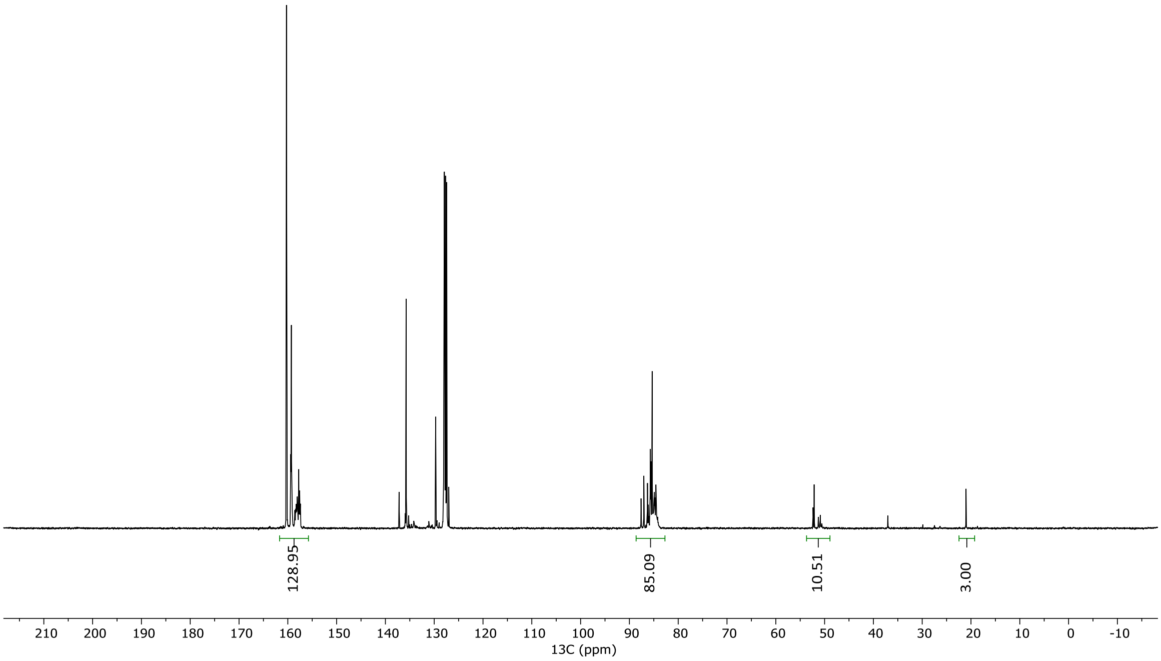

Figure S37: $^{13}C\{^{1}H\}$ NMR spectrum (101 MHz, C_6D_6, 298 K) of the reaction mixture resulting from the catalytic hydrosilylation of CO_2 using **4** at 40°C (Figure 7B).

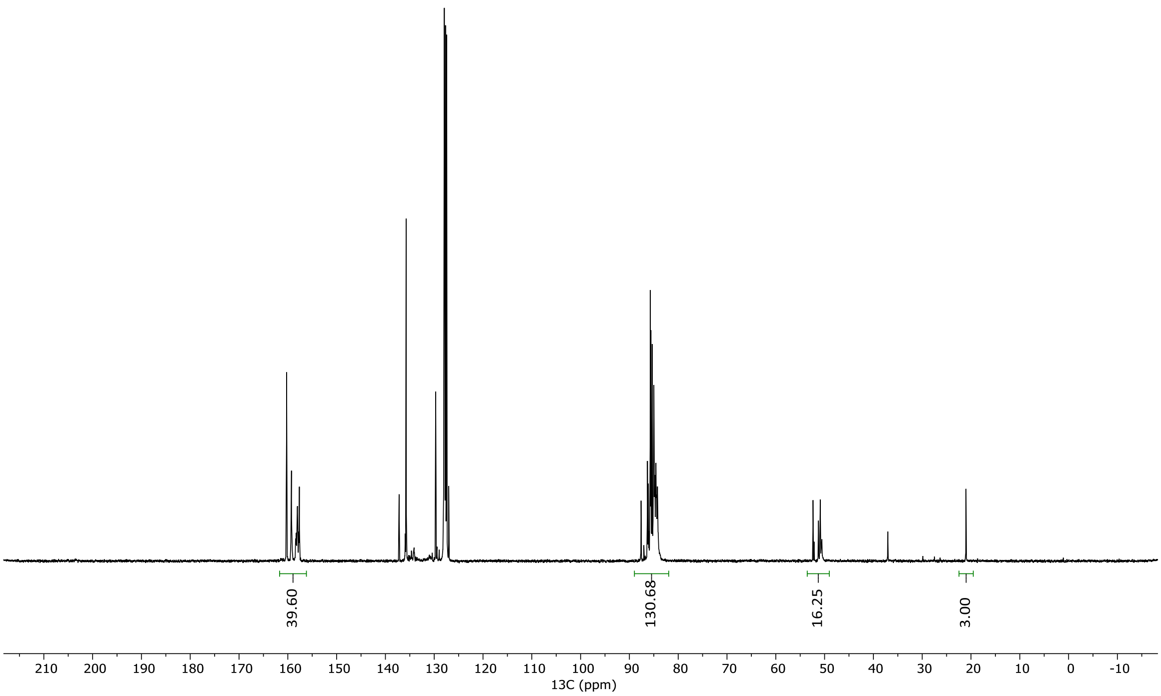

Figure S38: $^{13}C\{^{1}H\}$ NMR spectrum (101 MHz, C_6D_6, 298 K) of the reaction mixture resulting from the catalytic hydrosilylation of CO_2 using **4** at 60°C (Figure 7B).

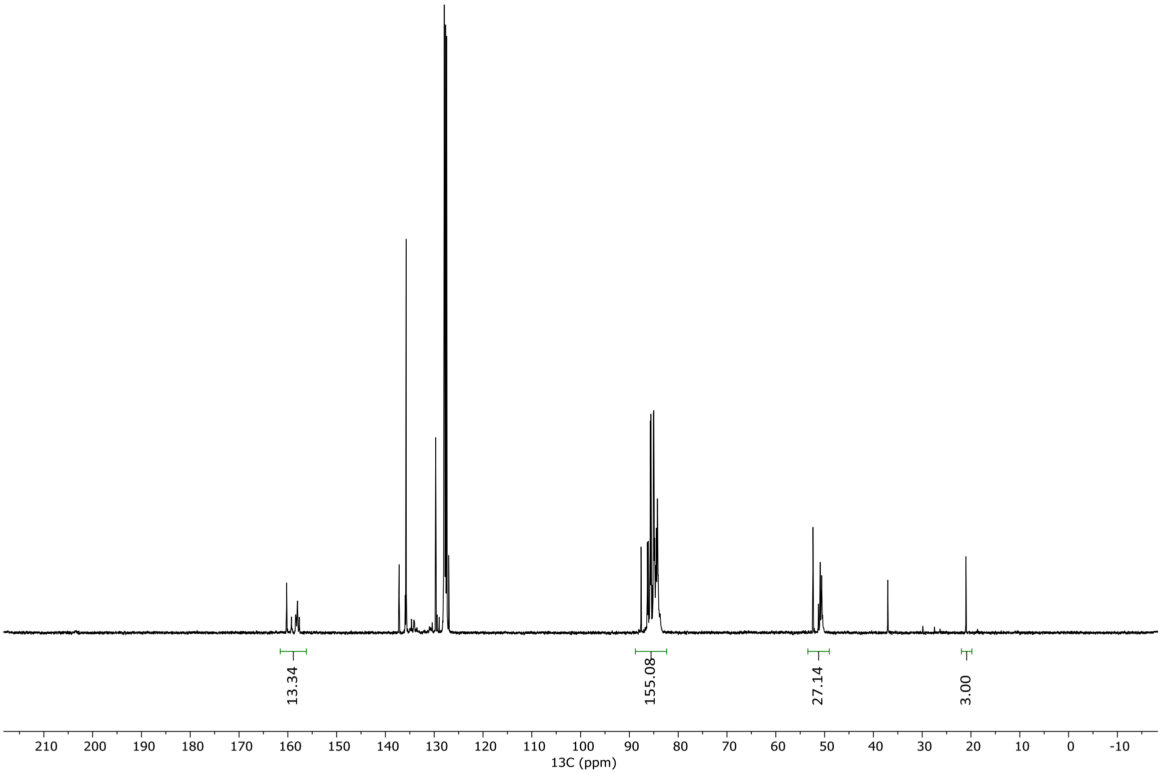

Figure S39: $^{13}C\{^{1}H\}$ NMR spectrum (101 MHz, C_6D_6, 298 K) of the reaction mixture resulting from the catalytic hydrosilylation of CO_2 using **4** at 80°C (Figure 7B).

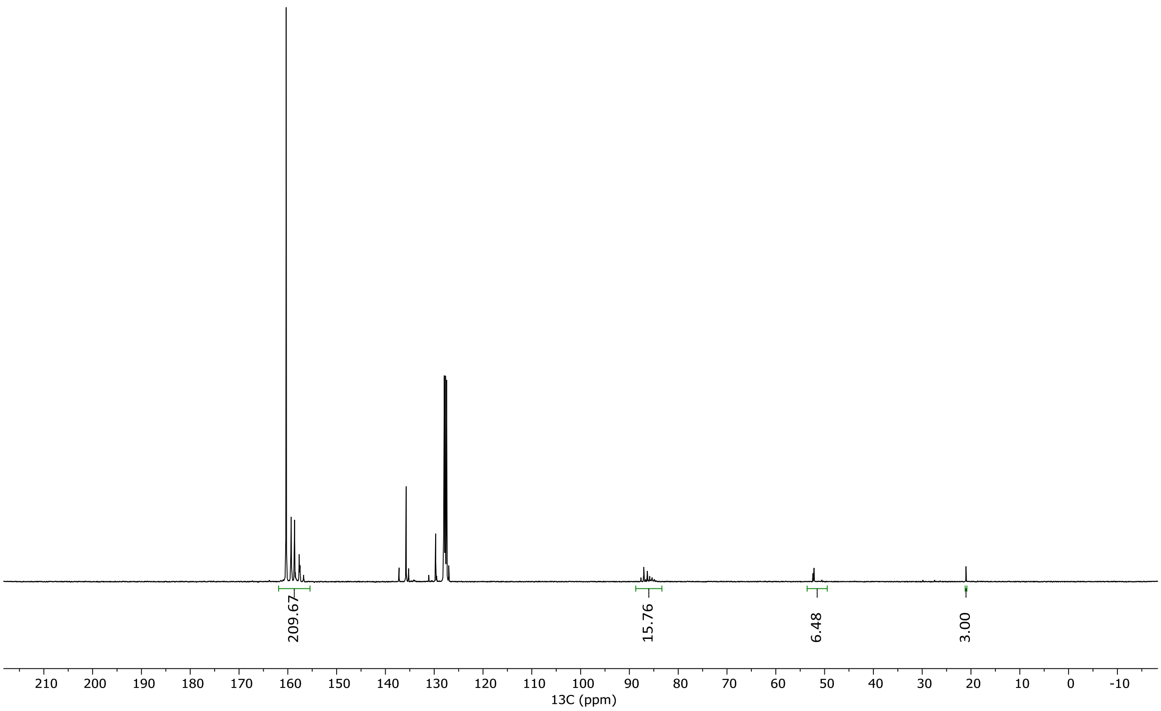

Figure S40: $^{13}C\{^{1}H\}$ NMR spectrum (101 MHz, C_6D_6, 298 K) of the reaction mixture resulting from the catalytic hydrosilylation of CO_2 using **7** at 25°C (Figure 7B).

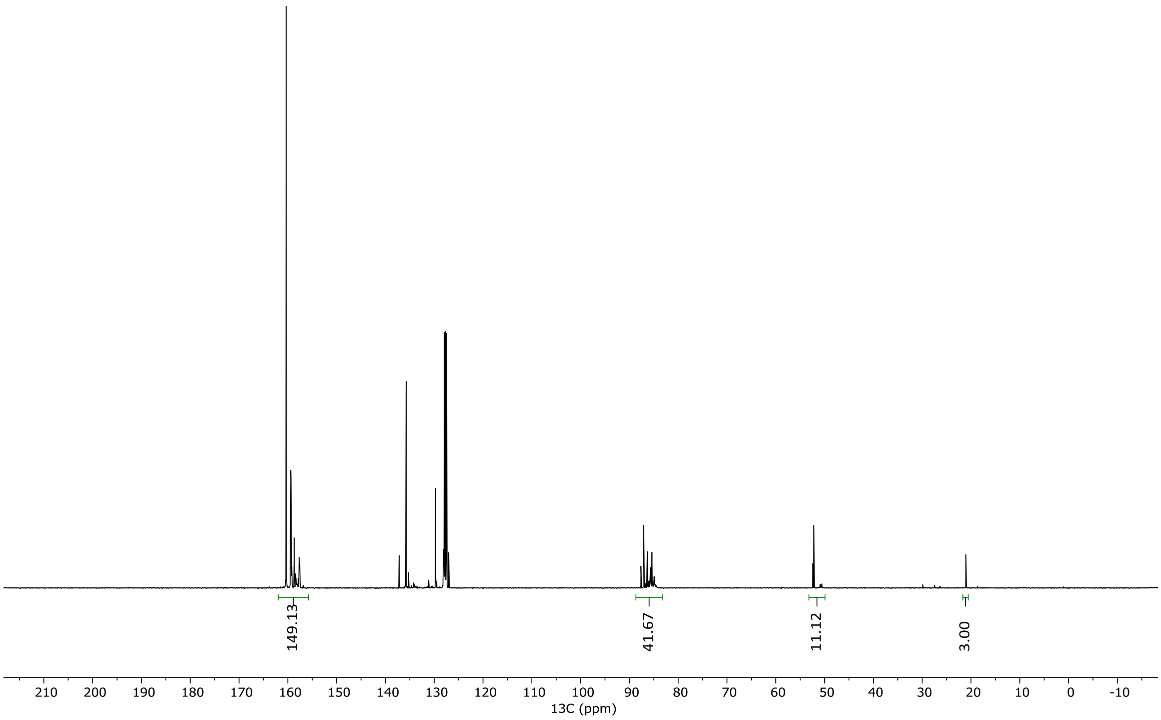

Figure S 41: $^{13}C\{^{1}H\}$ NMR spectrum (101 MHz, C_6D_6, 298 K) of the reaction mixture resulting from the catalytic hydrosilylation of CO_2 using **7** at 40°C (Figure 7B).

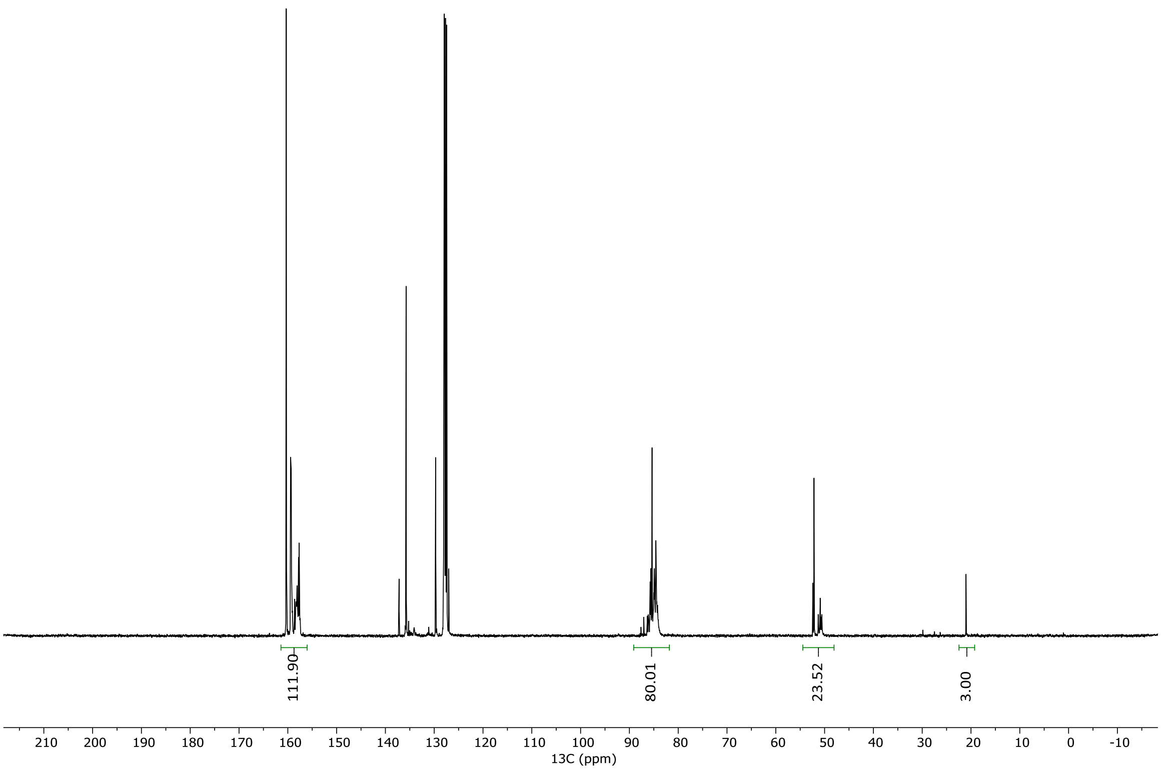

Figure S42: $^{13}C\{^1H\}$ NMR spectrum (101 MHz, C_6D_6, 298 K) of the reaction mixture resulting from the catalytic hydrosilylation of CO_2 using **7** at 60°C (Figure 7B).

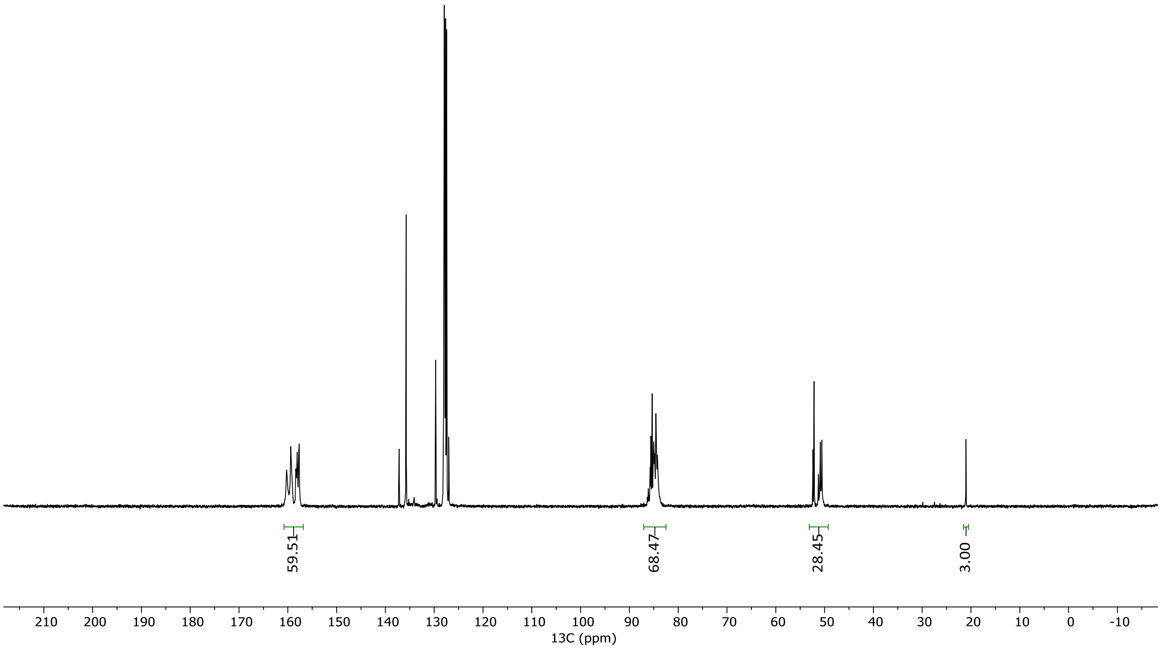

Figure S43: $^{13}C\{^1H\}$ NMR spectrum (101 MHz, C_6D_6, 298 K) of the reaction mixture resulting from the catalytic hydrosilylation of CO_2 using **7** at 80°C (Figure 7B).

4. Cartesian Coordinates of the Optimized Structures

4.1. Reactants and Products

CO_2

C	0.00000	0.00000	0.00000
O	0.00000	0.00000	1.15891
O	0.00000	0.00000	-1.15891

$PhSiH_3$

C	2.34584	-0.00593	0.00182
C	1.65355	1.20183	0.00050
C	1.64179	-1.20925	0.00047
H	2.20170	2.14746	0.00068
H	2.18203	-2.15946	0.00052
C	0.25986	1.20545	-0.00171
C	0.25053	-1.19934	-0.00169
H	-0.27209	2.16255	-0.00373
H	-0.28888	-2.15337	-0.00356
C	-0.46599	0.00702	-0.00230
H	3.43873	-0.01118	0.00330
Si	-2.34403	0.00135	0.00099
H	-2.86179	-0.76871	-1.16674
H	-2.86177	-0.64181	1.24327
H	-2.83498	1.40697	-0.07012

$(PhSiH_2)OCHO$

C	-3.44499	0.41215	-0.00036
C	-2.48285	1.41888	0.00006
C	-3.05305	-0.92538	-0.00031
H	-2.78886	2.46790	0.00011
H	-3.80542	-1.71770	-0.00057
C	-1.12898	1.09141	0.00028
C	-1.70027	-1.24867	-0.00009
H	-0.37966	1.88769	0.00066
H	-1.40622	-2.30509	-0.00009
C	-0.71769	-0.24767	0.00022
H	-4.50698	0.67021	-0.00083
Si	1.07438	-0.75887	0.00051
H	1.43566	-1.51731	1.22369
H	1.43485	-1.51882	-1.22197
O	1.93549	0.73501	0.00005
C	3.26021	0.71566	-0.00028
H	3.68185	1.74182	-0.00011
O	3.92691	-0.28286	-0.00069

$(PhSiH_2O)_2CH_2$

H	1.47651	2.07368	-0.55045
C	4.99138	-0.36464	0.81064
C	4.77076	-0.59249	-0.54646
C	3.91255	-0.26131	1.68583
H	5.61719	-0.67511	-1.23276
H	4.08542	-0.08067	2.74961
C	3.46939	-0.71695	-1.02414
C	2.61288	-0.38869	1.20179
H	3.30513	-0.89785	-2.09217
H	1.76902	-0.30538	1.89520
C	2.36894	-0.61833	-0.15988
H	6.01228	-0.26757	1.18898
Si	0.62167	-0.73328	-0.81244
H	0.52675	-1.66847	-1.96270
H	-0.28391	-1.13338	0.29777
O	0.17514	0.75769	-1.46465
C	0.40674	1.95972	-0.80563
H	0.11567	2.76924	-1.50180
O	-0.31158	2.05391	0.38373
C	-3.00044	-0.21558	-0.96551
C	-2.73825	0.38221	0.27575
C	-3.46403	-1.52521	-1.04602
H	-3.65706	-1.97888	-2.02124
C	-2.97603	-0.36619	1.43617
C	-3.68042	-2.25772	0.12037
H	-2.78526	0.08025	2.41830
H	-4.04289	-3.28710	0.05928
C	-3.43986	-1.67744	1.36339
H	-3.61360	-2.24935	2.27819
H	-2.81973	0.34763	-1.88726
Si	-1.99531	2.09163	0.38636
H	-2.42292	2.90648	-0.78860
H	-2.36180	2.71250	1.68430

Formaldehyde

O	0.00000	0.67275	-0.00000
C	-0.00000	-0.52318	0.00000
H	0.94591	-1.12147	0.00000
H	-0.94591	-1.12147	0.00000

$(PhSiH_2)_2O$

C	2.34055	2.26876	0.40982
C	1.62235	2.13402	-0.77572
C	2.71132	1.13583	1.13296
H	1.32500	3.02094	-1.34088
H	3.27724	1.23947	2.06211
C	1.27402	0.86687	-1.23557
C	2.36425	-0.12791	0.66428
H	0.69379	0.77340	-2.15974
H	2.66216	-1.01231	1.23971
C	1.63511	-0.28458	-0.52434
H	2.61327	3.26256	0.77420
Si	1.09584	-1.98511	-1.08778
H	0.45763	-1.86452	-2.42943

H	2.24502	-2.92758	-1.13595
O	0.00604	-2.61764	0.00093
C	-2.36391	-0.13679	-0.66426
C	-1.63348	-0.29062	0.52390
C	-2.71645	1.12555	-1.13265
H	-3.28331	1.22707	-2.06146
C	-1.27671	0.86226	1.23511
C	-2.34997	2.25989	-0.40956
H	-0.69559	0.77096	2.15893
H	-2.62700	3.25264	-0.77354
C	-1.63053	2.12799	0.77558
H	-1.33655	3.01605	1.34071
Si	-1.08826	-1.98921	1.08754
H	-2.23416	-2.93579	1.13344
H	-0.45254	-1.86664	2.43018
H	-2.65860	-1.02234	-1.23958

$(PhSiH_2)OCH_3$

C	-2.91962	0.31643	0.29817
C	-2.38188	-0.92124	0.64034
C	-2.13338	1.26056	-0.36160
H	-2.99650	-1.66081	1.15958
H	-2.55440	2.23317	-0.62838
C	-1.05623	-1.21272	0.32374
C	-0.81359	0.95997	-0.68194
H	-0.63971	-2.18654	0.60318
H	-0.20319	1.70748	-1.20121
C	-0.25176	-0.28166	-0.34563
H	-3.95802	0.54902	0.54775
Si	1.53725	-0.64943	-0.75242
H	1.84763	-2.01884	-0.24230
H	1.82529	-0.52509	-2.20266
O	2.53080	0.47669	-0.02069
C	2.47488	0.69504	1.36246
H	2.53439	-0.25176	1.93275
H	3.32451	1.32503	1.66153
H	1.54144	1.20862	1.65594

4.2. Trityl Cation and Triphenylmethane

$(Ph_3C)^+$

C	-0.00041	-0.00033	-0.00063
C	0.45960	1.36669	-0.00056
C	-0.27190	2.37350	-0.67321
C	1.65089	1.72579	0.67258
C	0.18060	3.68130	-0.68160
H	-1.16746	2.10118	-1.23504
C	2.08131	3.04102	0.68208
H	2.19946	0.96693	1.23386
C	1.35183	4.01747	0.00057
H	-0.37412	4.44604	-1.22785
H	2.98549	3.31431	1.22860
H	1.70023	5.05264	0.00097
C	0.95361	-1.08200	-0.00054
C	0.66941	-2.29388	0.67167
C	2.19150	-0.95062	-0.67251
C	1.59399	-3.32356	0.68100
H	-0.26235	-2.39043	1.23237
C	3.09882	-1.99551	-0.68077
H	2.40293	-0.03827	-1.23353
C	2.80477	-3.17857	0.00050
H	1.37895	-4.24374	1.22684
H	4.03889	-1.89632	-1.22606
H	3.52778	-3.99724	0.00115
C	-1.41412	-0.28535	-0.00042
C	-2.32061	0.56669	0.67288
C	-1.92015	-1.42229	-0.67310
C	-3.67483	0.28190	0.68227
H	-1.93746	1.42093	1.23439
C	-3.27897	-1.68439	-0.68147
H	-1.23622	-2.06167	-1.23462
C	-4.15571	-0.83808	0.00066
H	-4.36360	0.92828	1.22882
H	-3.66388	-2.54738	-1.22738
H	-5.22638	-1.05398	0.00109

Ph_3CH

C	-0.00205	-0.00267	-0.76423
H	-0.00140	-0.00029	-1.87064
C	0.11096	1.45098	-0.34230
C	-0.62273	1.99420	0.71337
C	1.01875	2.27210	-1.01930
C	-0.44505	3.32495	1.08897
H	-1.34439	1.37156	1.24820
C	1.19773	3.59917	-0.64817
H	1.59982	1.85086	-1.84641
C	0.46364	4.13140	0.41140
H	-1.02686	3.73332	1.91920
H	1.91057	4.22545	-1.19064
H	0.59932	5.17522	0.70501
C	-1.31645	-0.63412	-0.34569
C	-1.42251	-1.51277	0.73377
C	-2.47806	-0.28576	-1.04240
C	-2.66281	-2.02373	1.11264
H	-0.52489	-1.80317	1.28563
C	-3.71608	-0.79429	-0.66804
H	-2.40288	0.40869	-1.88554
C	-3.81245	-1.66758	0.41473
H	-2.72785	-2.70953	1.96117
H	-4.61263	-0.51054	-1.22511
H	-4.78380	-2.07150	0.71057
C	1.20234	-0.82398	-0.34257
C	1.50221	-1.99209	-1.05117
C	2.00000	-0.48195	0.75037
C	2.56500	-2.80438	-0.67344
H	0.87850	-2.26790	-1.90794
C	3.06520	-1.29499	1.13314
H	1.78662	0.43220	1.31026
C	3.35177	-2.45737	0.42423
H	2.78482	-3.71254	-1.24059
H	3.67856	-1.01272	1.99259
H	4.18999	-3.09198	0.72237

4.3. (I1-H)$^+$

(I1-H)$^+$_Co_L1

Co	0.00000	-0.71393	-0.00001
P	2.18754	-0.56767	0.03232
N	2.31110	1.18969	0.11947
C	1.15191	1.88322	0.05553
N	1.18082	3.21370	0.05156
C	-0.00001	3.80897	0.00008
N	-1.18083	3.21370	-0.05158
C	-1.15191	1.88322	-0.05558
N	-0.00000	1.16451	-0.00002
N	-2.31110	1.18968	-0.11952
P	-2.18754	-0.56768	-0.03233
N	-3.27437	-1.12426	-1.18670
C	-4.39827	-1.86375	-0.63017
H	-4.21777	-2.95334	-0.69870
H	-5.31540	-1.64627	-1.20168
C	-4.51862	-1.41747	0.81812
H	-5.19085	-0.54241	0.91858
H	-4.92581	-2.21429	1.45923
N	-3.16391	-1.08661	1.23920
C	-2.97234	-0.57917	2.57666
H	-1.90471	-0.38371	2.75594
C	-2.92568	-1.33365	-2.57072
N	3.16394	-1.08663	-1.23918
C	4.51864	-1.41749	-0.81807
H	5.19087	-0.54243	-0.91852
H	4.92584	-2.21432	-1.45916
C	4.39825	-1.86375	0.63022
H	4.21775	-2.95333	0.69877
H	5.31538	-1.64625	1.20175
N	3.27435	-1.12424	1.18672
C	2.92562	-1.33362	2.57073
H	2.71007	-2.39530	2.78756
C	2.97240	-0.57921	-2.57666
H	3.53008	0.36078	-2.74899
C	3.58225	1.89118	0.17363
H	3.51022	2.75386	0.84618
H	3.88295	2.25492	-0.82041
H	4.34331	1.20314	0.56041
C	-3.58225	1.89117	-0.17367
H	-3.88293	2.25495	0.82036
H	-4.34332	1.20313	-0.56042
H	-3.51023	2.75384	-0.84625
H	0.00000	4.90517	-0.00010
H	3.74778	-1.01114	3.22810
H	-3.31098	-1.31827	3.31737
H	-2.03793	-0.73963	-2.83185
H	-2.71013	-2.39534	-2.78754
H	-3.74785	-1.01119	-3.22807
H	3.31104	-1.31832	-3.31735
H	1.90478	-0.38373	-2.75596
H	2.03786	-0.73959	2.83183
H	-3.53000	0.36083	2.74899

(I1-H)$^+$_Co_L2

Co	0.00000	-0.62511	0.28811
P	-2.18403	-0.48789	0.17649
O	-2.27713	1.18689	-0.37272
C	-1.15219	1.82071	-0.56722
N	-1.18351	3.07271	-0.97564
C	0.00000	3.64330	-1.15832
N	1.18351	3.07271	-0.97562
C	1.15219	1.82072	-0.56720
N	0.00000	1.13985	-0.33541
O	2.27713	1.18690	-0.37268
P	2.18403	-0.48789	0.17650
N	3.29403	-0.49571	1.42156
C	4.64999	-0.63071	0.88305
H	5.30955	-1.06809	1.64646
H	5.05170	0.36651	0.61797
C	4.52702	-1.51655	-0.34762
H	5.27593	-1.25416	-1.11143
H	4.66040	-2.58455	-0.09898
N	3.17794	-1.30062	-0.86382
C	2.78945	-1.89712	-2.11838
H	1.71527	-1.74234	-2.29524
C	3.12727	0.38205	2.56149
N	-3.29400	-0.49569	1.42158
C	-4.64997	-0.63069	0.88309
H	-5.05169	0.36654	0.61801
H	-5.30952	-1.06805	1.64651
C	-4.52704	-1.51654	-0.34757
H	-4.66042	-2.58454	-0.09892
H	-5.27596	-1.25415	-1.11137
N	-3.17797	-1.30062	-0.86381
C	-2.78950	-1.89714	-2.11837
H	-2.98560	-2.98185	-2.11320
C	-3.12722	0.38208	2.56149
H	-3.47637	1.40736	2.34247
H	0.00000	4.68599	-1.49413
H	-3.34109	-1.44972	-2.96011
H	2.98556	-2.98183	-2.11323
H	2.06795	0.42737	2.85234
H	3.69696	-0.00604	3.41717
H	3.47642	1.40734	2.34247
H	-3.69689	-0.00600	3.41719
H	-2.06789	0.42740	2.85231
H	-1.71533	-1.74234	-2.29527
H	3.34100	-1.44969	-2.96014

(I1-H)$^+$_Co_L3

Co	-0.00006	-0.62926	-0.00121
P	-2.20319	-0.46707	0.09187
C	-2.23590	1.20850	0.92966
C	-1.07108	1.97858	0.43318
N	-1.10134	3.29974	0.42072
C	0.00027	3.90976	0.00186
N	1.10187	3.30014	-0.41758
C	1.07155	1.97899	-0.43149
N	0.00016	1.26458	0.00033
C	2.23651	1.20943	-0.92846
P	2.20316	-0.46723	-0.09290
N	3.19943	-0.32904	1.27061
C	4.44992	-1.06461	1.11414
H	4.38227	-2.04298	1.62823
H	5.28636	-0.50718	1.56551
C	4.64184	-1.25620	-0.37789
H	5.12704	-0.36836	-0.83487
H	5.27112	-2.12990	-0.60465
N	3.30144	-1.45652	-0.92044
C	3.19508	-1.64018	-2.34824
H	2.14177	-1.76240	-2.64075
C	2.67279	-0.20956	2.61053
N	-3.30037	-1.45768	0.91931
C	-4.64139	-1.25706	0.37843
H	-5.12636	-0.36992	0.83701
H	-5.27018	-2.13122	0.60476

C	-4.45105	-1.06356	-1.11355
H	-4.38351	-2.04126	-1.62891
H	-5.28816	-0.50591	-1.56340
N	-3.20105	-0.32723	-1.27032
C	-2.67582	-0.20584	-2.61063
H	-2.54820	-1.19134	-3.09691
C	-3.19243	-1.64327	2.34675
H	-3.61936	-0.79428	2.91683
H	0.00022	5.00508	0.00226
H	-3.35471	0.39636	-3.23333
H	3.73365	-2.54948	-2.65133
H	1.69600	0.29469	2.59666
H	2.54600	-1.19571	3.09572
H	3.35025	0.39294	3.23449
H	-3.73055	-2.55305	2.64918
H	-2.13878	-1.76574	2.63794
H	-1.69973	0.29976	-2.59725
H	3.62252	-0.79036	-2.91669
H	2.08659	1.00921	-2.00593
H	-3.16447	1.78506	0.81644
H	3.16507	1.78576	-0.81392
H	-2.08537	1.00675	2.00677

(I1-H)$^{+}$_Co_L4

Co	0.00000	-0.67059	-0.14509
P	2.17230	-0.56043	-0.04026
N	2.33296	1.17179	-0.17152
C	1.17668	1.91310	-0.17145
C	1.20428	3.31126	-0.17386
C	-0.00000	3.99600	-0.17714
C	-1.20428	3.31126	-0.17386
C	-1.17668	1.91310	-0.17145
N	-0.00000	1.22719	-0.16928
N	-2.33297	1.17179	-0.17152
P	-2.17230	-0.56043	-0.04026
N	-3.36208	-1.17398	-1.06623
C	-4.36415	-1.97831	-0.38454
H	-4.11110	-3.05446	-0.44360
H	-5.34726	-1.84578	-0.86525
C	-4.37122	-1.49911	1.05765
H	-5.07896	-0.65799	1.20254
H	-4.67388	-2.29794	1.75232
N	-3.00290	-1.09030	1.33372
C	-2.70381	-0.53642	2.63119
H	-2.94332	-1.26262	3.42195
C	-3.14013	-1.39697	-2.47366
H	-2.85325	-2.44188	-2.69170
N	3.36207	-1.17398	-1.06624
C	4.36415	-1.97831	-0.38456
H	5.34725	-1.84578	-0.86528
H	4.11109	-3.05446	-0.44361
C	4.37122	-1.49912	1.05763
H	4.67389	-2.29795	1.75230
H	5.07897	-0.65799	1.20252
N	3.00291	-1.09030	1.33372
C	2.70383	-0.53643	2.63119
H	3.27459	0.39055	2.83265
C	3.14011	-1.39696	-2.47367
H	4.05191	-1.16795	-3.04664
C	3.62420	1.82620	-0.18133
H	3.71850	2.50937	-1.03901
H	4.40460	1.06505	-0.28550
H	3.80326	2.39410	0.74622
C	-3.62421	1.82619	-0.18134
H	-3.80327	2.39409	0.74621
H	-4.40460	1.06505	-0.28551
H	-3.71850	2.50937	-1.03901
H	-4.05193	-1.16796	-3.04663
H	-2.34161	-0.73544	-2.83955
H	-1.63117	-0.30034	2.70072
H	-3.27456	0.39056	2.83264
H	2.34158	-0.73544	-2.83955
H	2.85324	-2.44188	-2.69170
H	2.94333	-1.26263	3.42195
H	1.63119	-0.30033	2.70073
H	-0.00000	5.08792	-0.17755
H	-2.15096	3.84653	-0.16547
H	2.15095	3.84653	-0.16547

(I1-H)$^{+}$_Co_L5

Co	0.05629	-1.42667	0.00727
P	-2.13698	-1.35695	-0.02881
N	-2.31781	0.40015	-0.11764
C	-1.18448	1.12807	-0.05459
N	-1.25419	2.45708	-0.05245
C	-0.09279	3.08132	-0.00206
N	1.10484	2.53907	0.04870
C	1.12214	1.20434	0.05454
N	-0.00627	0.45066	0.00223
N	2.30249	0.55673	0.11632
P	2.23927	-1.21063	0.03431
N	3.34544	-1.72341	1.18683
C	4.50144	-2.41306	0.63014
H	4.37134	-3.50894	0.70714
H	5.40899	-2.14936	1.19734
C	4.59723	-1.97094	-0.82164
H	5.23543	-1.07218	-0.93154
H	5.03004	-2.75586	-1.46044
N	3.22871	-1.69280	-1.23862
C	3.01146	-1.20297	-2.57918
H	1.93655	-1.04786	-2.75419
C	3.01354	-1.93552	2.57498
N	-3.10027	-1.90193	1.23885
C	-4.44294	-2.27603	0.81296
H	-5.14280	-1.42332	0.91426
H	-4.82516	-3.08668	1.45193
C	-4.30555	-2.71588	-0.63608
H	-4.09812	-3.80021	-0.70734
H	-5.22520	-2.51896	-1.21084
N	-3.19705	-1.94846	-1.18736
C	-2.83961	-2.14372	-2.57161
H	-2.60165	-3.19971	-2.79159
C	-2.92844	-1.39260	2.57876
H	-3.51751	-0.47247	2.75241
C	-3.61240	1.05941	-0.17357
H	-3.92864	1.40536	0.82175
H	-4.34756	0.34842	-0.56843
H	-3.56714	1.92816	-0.84048
C	3.55129	1.29957	0.16383
H	3.84689	1.64998	-0.83630
H	4.33009	0.64298	0.56949
H	3.44848	2.17346	0.81732
H	-3.66610	-1.83531	-3.23014
H	3.37270	-1.93523	-3.31581
H	2.09736	-1.38552	2.83410
H	2.85420	-3.00454	2.80268
H	3.82055	-1.56434	3.22530
H	-3.24419	-2.14505	3.31597
H	-1.86840	-1.16266	2.76167
H	-1.96348	-1.53045	-2.82731
H	3.53411	-0.24517	-2.76163
C	-0.18774	4.60877	-0.00183
F	-0.87925	5.00813	1.05994
F	1.00250	5.17555	0.03030
F	-0.82381	5.01469	-1.09471

(I1-H)$^+$_Co_L6

Co	0.00000	-1.21350	-0.00300
P	-2.20122	-1.03590	0.00065
N	-2.31733	0.70313	-0.00873
C	-1.14908	1.38841	-0.00459
N	-1.18075	2.71693	0.00028
C	-0.00001	3.31501	0.00219
N	1.18075	2.71694	0.00027
C	1.14909	1.38842	-0.00466
N	0.00000	0.66466	-0.00556
N	2.31733	0.70314	-0.00895
P	2.20121	-1.03590	0.00055
C	-3.57153	1.44483	-0.00163
H	-3.66473	2.04575	0.91310
H	-4.41072	0.74222	-0.05263
H	-3.62395	2.12275	-0.86337
C	3.57155	1.44481	-0.00157
H	3.62360	2.12362	-0.86261
H	4.41071	0.74224	-0.05370
H	3.66517	2.04476	0.91376
H	-0.00001	4.41081	0.00664
C	3.21287	-1.52854	1.43430
H	2.75724	-1.14921	2.35857
H	3.23893	-2.62731	1.48415
H	4.24774	-1.16473	1.35439
C	-3.21267	-1.52865	1.43451
H	-3.23954	-2.62744	1.48365
H	-2.75645	-1.15022	2.35886
H	-4.24730	-1.16403	1.35518
C	-3.22227	-1.54707	-1.41952
H	-2.77846	-1.17106	-2.35085
H	-3.23845	-2.64643	-1.46047
H	-4.25999	-1.19313	-1.33329
C	3.22205	-1.54718	-1.41973
H	4.25957	-1.19252	-1.33407
H	3.23895	-2.64655	-1.46003
H	2.77768	-1.17198	-2.35112

(I1-H)$^+$_Co_L7

Co	0.00000	-0.90701	0.00000
P	-2.16784	-0.71266	-0.01345
N	-2.31676	1.00302	-0.06614
C	-1.15015	1.70222	-0.03003
N	-1.17927	3.02710	-0.02784
C	0.00000	3.62685	-0.00000
N	1.17927	3.02710	0.02784
C	1.15015	1.70222	0.03003
N	0.00000	0.97953	0.00000
N	2.31676	1.00302	0.06614
P	2.16784	-0.71266	0.01345
O	3.19292	-1.19440	1.18915
C	4.31507	-1.92531	0.68462
H	4.12792	-3.00126	0.81911
H	5.19982	-1.64357	1.27015
C	4.44146	-1.54363	-0.78638
H	5.09792	-0.67202	-0.93447
H	4.80621	-2.36939	-1.40874
O	3.11600	-1.21380	-1.21577
O	-3.11600	-1.21380	1.21577
C	-4.44146	-1.54363	0.78638
H	-5.09792	-0.67202	0.93447
H	-4.80621	-2.36939	1.40874
C	-4.31507	-1.92531	-0.68463
H	-4.12792	-3.00126	-0.81911
H	-5.19982	-1.64357	-1.27015
O	-3.19291	-1.19440	-1.18915
C	-3.60917	1.67588	-0.07997
H	-3.47644	2.70592	-0.42467
H	-4.05052	1.69237	0.92689
H	-4.27966	1.15221	-0.77354
C	3.60917	1.67588	0.07997
H	4.05052	1.69238	-0.92689
H	4.27967	1.15221	0.77354
H	3.47643	2.70592	0.42468
H	0.00000	4.72241	0.00000

(I1-H)$^+$_Co_L8

Co	0.00000	1.06174	0.00001
N	1.99679	0.85955	-0.02452
C	2.29585	-0.50997	0.46991
C	1.13252	-1.40938	0.19711
N	1.17178	-2.72462	0.19537
C	-0.00001	-3.33391	0.00002
N	-1.17179	-2.72461	-0.19538
C	-1.13252	-1.40938	-0.19712
N	-0.00000	-0.72531	-0.00000
C	-2.29584	-0.50996	-0.46992
N	-1.99679	0.85955	0.02451
H	-0.00000	-4.42816	-0.00003
H	-2.43333	-0.47417	-1.56370
H	3.23184	-0.91206	0.04493
H	-3.23184	-0.91206	-0.04495
H	2.43334	-0.47418	1.56368
C	-2.69886	1.86079	-0.78833
H	-2.49688	2.86247	-0.38479
H	-2.34125	1.81525	-1.82550
H	-3.79080	1.69510	-0.77229
C	2.41908	0.97201	-1.43189
H	1.93569	0.19181	-2.03406
H	2.12603	1.95125	-1.83209
H	3.51549	0.86607	-1.51760
C	-2.41908	0.97200	1.43189
H	-1.93570	0.19180	2.03406
H	-2.12603	1.95124	1.83210
H	-3.51549	0.86606	1.51759
C	2.69887	1.86078	0.78834
H	3.79080	1.69509	0.77229
H	2.49689	2.86246	0.38480
H	2.34126	1.81523	1.82550

(I1-H)$^+$_Co_L9

Co	0.00000	1.20358	-0.00003
P	-2.15862	1.01366	-0.00002
N	-2.39668	-0.68376	0.00010
C	-1.20295	-1.42141	0.00004
C	-1.21213	-2.82420	0.00001
C	0.00000	-3.50700	-0.00004
C	1.21213	-2.82420	-0.00003
C	1.20295	-1.42141	-0.00001
C	0.00000	-0.68374	0.00002
N	2.39668	-0.68376	-0.00002
P	2.15862	1.01366	0.00001
C	-3.67439	-1.33828	0.00001
H	-3.80924	-1.97524	-0.89266
H	-4.48305	-0.59603	0.00016
H	-3.80919	-1.97554	0.89246
C	3.67439	-1.33828	0.00003
H	3.80916	-1.97542	0.89258
H	4.48305	-0.59603	0.00010
H	3.80927	-1.97537	-0.89254
H	0.00000	-4.60026	-0.00007
C	3.17645	1.60258	-1.41270

H	2.74276	1.22046	-2.34686
H	3.15210	2.70251	-1.44096
H	4.22723	1.28164	-1.33732
C	-3.17632	1.60245	-1.41286
H	-3.15182	2.70237	-1.44136
H	-2.74265	1.22008	-2.34693
H	-4.22715	1.28166	-1.33747
C	-3.17638	1.60265	1.41271
H	-2.74263	1.22059	2.34687
H	-3.15207	2.70257	1.44090
H	-4.22716	1.28166	1.33741
C	3.17626	1.60252	1.41288
H	4.22708	1.28169	1.33757
H	3.15179	2.70243	1.44130
H	2.74252	1.22021	2.34694
H	-2.14936	-3.38404	-0.00000
H	2.14936	-3.38404	-0.00006

(I1-H)⁺_Co_L10

Co	-0.00002	-1.23563	0.00002
O	1.95620	-1.00187	0.24631
C	2.40900	0.34369	0.01205
C	1.21229	1.24257	0.02632
C	1.21783	2.63612	0.03569
C	0.00004	3.32451	-0.00001
C	-1.21776	2.63615	-0.03572
C	-1.21226	1.24261	-0.02630
C	0.00001	0.55329	0.00002
C	-2.40899	0.34375	-0.01201
O	-1.95622	-1.00181	-0.24634
H	0.00006	4.41744	-0.00003
H	-2.91852	0.36596	0.97189
H	3.16245	0.59611	0.78194
H	-3.16247	0.59621	-0.78185
H	2.91858	0.36594	-0.97182
C	-2.88702	-1.98196	0.13596
H	-2.50068	-2.95721	-0.18817
H	-3.02168	-1.98988	1.23234
H	-3.86096	-1.80050	-0.35048
C	2.88697	-1.98203	-0.13602
H	3.86090	-1.80062	0.35046
H	2.50060	-2.95728	0.18806
H	3.02165	-1.98990	-1.23240
H	2.15834	3.19644	0.06419
H	-2.15826	3.19650	-0.06425

(I1-H)⁺_Co_L11

Co	-0.00002	-1.08387	0.00001
N	-2.00614	-0.88239	-0.03962
C	-2.31997	0.45466	0.54865
C	-1.19036	1.39547	0.24397
C	-1.19809	2.78904	0.23360
C	0.00006	3.47670	0.00002
C	1.19819	2.78900	-0.23359
C	1.19040	1.39543	-0.24402
C	0.00001	0.71123	-0.00004
C	2.31997	0.45458	-0.54869
N	2.00611	-0.88244	0.03962
H	0.00008	4.56998	0.00004
H	2.40648	0.31239	-1.63932
H	-3.31015	0.80196	0.18973
H	3.31017	0.80186	-0.18980
H	-2.40650	0.31251	1.63929
C	2.71003	-1.93653	-0.68077
H	2.48721	-2.90855	-0.21613
H	2.36888	-1.96165	-1.72451
H	3.80758	-1.78066	-0.65787
C	-2.40718	-0.89556	-1.44911
H	-1.92685	-0.06236	-1.97663
H	-2.08753	-1.83364	-1.92209
H	-3.50862	-0.80053	-1.53965
C	2.40716	-0.89558	1.44911
H	1.92686	-0.06236	1.97660
H	2.08749	-1.83364	1.92212
H	3.50861	-0.80058	1.53965
C	-2.71010	-1.93644	0.68080
H	-3.80764	-1.78054	0.65789
H	-2.48730	-2.90848	0.21618
H	-2.36895	-1.96154	1.72454
H	-2.12062	3.35103	0.41441
H	2.12074	3.35096	-0.41437

(I1-H)⁺_Fe_L1

Fe	0.00000	-0.77050	-0.00006
P	-2.13094	-0.60560	-0.03763
N	-2.29716	1.17609	-0.14810
C	-1.14576	1.88058	-0.07165
N	-1.18095	3.21818	-0.07102
C	-0.00001	3.81001	0.00006
N	1.18094	3.21819	0.07107
C	1.14576	1.88058	0.07166
N	-0.00000	1.16011	-0.00001
N	2.29716	1.17610	0.14809
P	2.13094	-0.60559	0.03759
N	3.31225	-1.11382	1.17427
C	4.46466	-1.76528	0.58925
H	4.34442	-2.86858	0.61177
H	5.38052	-1.52806	1.16039
C	4.55119	-1.26646	-0.84337
H	5.16243	-0.34135	-0.91252
H	5.02714	-2.01124	-1.50395
N	3.18211	-1.03889	-1.24758
C	2.94376	-0.52121	-2.56590
H	1.85974	-0.41610	-2.72869
C	2.94246	-1.50846	2.50670
N	-3.18203	-1.03893	1.24760
C	-4.55114	-1.26646	0.84347
H	-5.16236	-0.34134	0.91267
H	-5.02706	-2.01125	1.50405
C	-4.46471	-1.76525	-0.58918
H	-4.34451	-2.86855	-0.61174
H	-5.38059	-1.52798	-1.16026
N	-3.31231	-1.11381	-1.17425
C	-2.94261	-1.50842	-2.50672
H	-2.79614	-2.60337	-2.59261
C	-2.94361	-0.52124	2.56590
H	-3.41252	0.47143	2.72449
C	-3.56563	1.86112	-0.24053
H	-3.46707	2.76351	-0.85690
H	-3.94093	2.16887	0.74964
H	-4.29324	1.18727	-0.71089
C	3.56562	1.86112	0.24055
H	3.94096	2.16883	-0.74962
H	4.29322	1.18727	0.71095
H	3.46706	2.76353	0.85688
H	-0.00001	4.90753	0.00002
H	-3.71870	-1.21190	-3.23248
H	3.34077	-1.20824	-3.33086
H	2.00130	-1.01874	2.79588
H	2.79590	-2.60340	2.59253
H	3.71853	-1.21203	3.23252
H	-3.34060	-1.20826	3.33088
H	-1.85958	-0.41615	2.72864

H	-2.00144	-1.01875	-2.79592
H	3.41270	0.47145	-2.72447

(I1-H)$^{+}$_Fe_L2

Fe	0.00000	-0.58620	0.54599
P	-2.10762	-0.51687	0.27707
O	-2.26733	1.15887	-0.39192
C	-1.14929	1.78466	-0.61874
N	-1.18524	3.00148	-1.14710
C	-0.00000	3.55010	-1.37175
N	1.18524	3.00148	-1.14710
C	1.14929	1.78465	-0.61877
N	-0.00000	1.14011	-0.30245
O	2.26733	1.15887	-0.39192
P	2.10762	-0.51687	0.27707
N	3.40969	-0.41891	1.36886
C	4.67878	-0.48989	0.66155
H	5.47634	-0.82678	1.34309
H	4.96474	0.51187	0.27970
C	4.45907	-1.46233	-0.48673
H	5.09605	-1.21336	-1.35358
H	4.70168	-2.50155	-0.18801
N	3.05425	-1.36296	-0.82539
C	2.54345	-2.14317	-1.91603
H	1.44630	-2.06957	-1.94061
C	3.33998	0.53872	2.44489
N	-3.40970	-0.41891	1.36886
C	-4.67878	-0.48989	0.66155
H	-4.96474	0.51187	0.27969
H	-5.47634	-0.82679	1.34309
C	-4.45907	-1.46233	-0.48673
H	-4.70168	-2.50155	-0.18801
H	-5.09604	-1.21337	-1.35358
N	-3.05425	-1.36296	-0.82539
C	-2.54345	-2.14317	-1.91603
H	-2.81442	-3.20903	-1.80426
C	-3.33998	0.53872	2.44489
H	-3.59437	1.56190	2.10637
H	-0.00000	4.56230	-1.79404
H	-2.93490	-1.79289	-2.88686
H	2.81442	-3.20903	-1.80426
H	2.32229	0.55748	2.86223
H	4.03623	0.25753	3.25012
H	3.59437	1.56191	2.10637
H	-4.03624	0.25753	3.25011
H	-2.32230	0.55748	2.86223
H	-1.44630	-2.06957	-1.94061
H	2.93491	-1.79289	-2.88686

(I1-H)$^{+}$_Fe_L3

Fe	-0.00003	-0.68375	0.00004
P	-2.15404	-0.53714	-0.12185
C	-2.19664	1.14824	-0.98397
C	-1.05539	1.93546	-0.46583
N	-1.09201	3.25958	-0.44669
C	-0.00005	3.86956	-0.00001
N	1.09189	3.25954	0.44672
C	1.05524	1.93545	0.46587
N	-0.00008	1.21199	-0.00003
C	2.19642	1.14822	0.98408
P	2.15411	-0.53716	0.12176
N	3.33467	-1.43260	1.00303
C	4.59595	-1.55962	0.29994
H	4.66050	-2.54597	-0.20555
H	5.45022	-1.49091	0.99688
C	4.61723	-0.44428	-0.72774
H	4.96346	0.50821	-0.26687
H	5.30043	-0.66707	-1.56417
N	3.25322	-0.34653	-1.20406
C	2.98158	0.69139	-2.16203
H	1.92388	0.65167	-2.46410
C	2.95567	-2.53406	1.84733
N	-3.25309	-0.34680	1.20410
C	-4.61711	-0.44422	0.72769
H	-4.96316	0.50845	0.26706
H	-5.30037	-0.66709	1.56404
C	-4.59601	-1.55934	-0.30026
H	-4.66103	-2.54579	0.20496
H	-5.45018	-1.49014	-0.99729
N	-3.33456	-1.43271	-1.00311
C	-2.95561	-2.53435	-1.84720
H	-2.97423	-3.50241	-1.30667
C	-2.98139	0.69107	2.16215
H	-3.19439	1.70924	1.77015
H	0.00000	4.96520	-0.00005
H	-3.63614	-2.61827	-2.71123
H	3.59834	0.54703	-3.06317
H	1.93758	-2.37998	2.23401
H	2.97248	-3.50205	1.30661
H	3.63737	-2.61884	2.71035
H	-3.59858	0.54697	3.06303
H	-1.92382	0.65092	2.46462
H	-1.93679	-2.38128	-2.23238
H	3.19513	1.70948	-1.77015
H	3.13671	1.71593	0.91393
H	-2.03290	0.90228	-2.04638
H	2.03257	0.90205	2.04643
H	-3.13691	1.71597	-0.91375

(I1-H)$^{+}$_Fe_L4

Fe	-0.00000	-0.75257	-0.17363
P	2.11851	-0.61009	-0.03517
N	2.31967	1.14062	-0.26545
C	1.17172	1.88950	-0.27629
C	1.20368	3.29234	-0.30905
C	0.00000	3.97979	-0.32527
C	-1.20368	3.29234	-0.30904
C	-1.17172	1.88950	-0.27629
N	0.00000	1.20023	-0.25429
N	-2.31967	1.14062	-0.26544
P	-2.11851	-0.61009	-0.03517
N	-3.41789	-1.22209	-0.98576
C	-4.45023	-1.88431	-0.21786
H	-4.26215	-2.97718	-0.16126
H	-5.43833	-1.75026	-0.69437
C	-4.40408	-1.26616	1.16872
H	-5.04401	-0.35875	1.22786
H	-4.77729	-1.96539	1.93686
N	-3.01082	-0.96316	1.39573
C	-2.65505	-0.30005	2.61809
H	-2.96804	-0.89720	3.49003
C	-3.18073	-1.71326	-2.31645
H	-2.94924	-2.79719	-2.33037
N	3.41789	-1.22209	-0.98575
C	4.45023	-1.88431	-0.21785
H	5.43833	-1.75026	-0.69437
H	4.26216	-2.97718	-0.16125
C	4.40408	-1.26615	1.16872
H	4.77729	-1.96539	1.93687
H	5.04401	-0.35875	1.22786
N	3.01081	-0.96316	1.39573
C	2.65505	-0.30004	2.61809
H	3.11845	0.70463	2.70835

C	3.18073	-1.71327	-2.31645
H	4.06417	-1.54667	-2.95574
C	3.60454	1.78685	-0.32429
H	3.66502	2.48118	-1.17852
H	4.37693	1.02445	-0.47361
H	3.83425	2.35356	0.59658
C	-3.60454	1.78685	-0.32428
H	-3.83425	2.35357	0.59659
H	-4.37693	1.02445	-0.47360
H	-3.66502	2.48119	-1.17851
H	-4.06417	-1.54666	-2.95575
H	-2.33025	-1.18148	-2.76705
H	-1.56164	-0.17528	2.66324
H	-3.11845	0.70462	2.70835
H	2.33025	-1.18150	-2.76705
H	2.94925	-2.79720	-2.33036
H	2.96804	-0.89719	3.49003
H	1.56163	-0.17527	2.66324
H	0.00000	5.07242	-0.34568
H	-2.15164	3.82680	-0.30767
H	2.15164	3.82680	-0.30767

(I1-H)$^+$_Fe_L5

Fe	0.05468	-1.48150	0.01632
P	-2.08294	-1.38951	-0.03076
N	-2.30415	0.38808	-0.13668
C	-1.17636	1.12404	-0.06428
N	-1.24937	2.45903	-0.06688
C	-0.08638	3.07813	-0.00161
N	1.11043	2.53617	0.06561
C	1.11910	1.19562	0.07020
N	-0.00523	0.44027	0.00667
N	2.29175	0.53409	0.14164
P	2.18203	-1.25631	0.04146
N	3.38069	-1.72158	1.17246
C	4.55766	-2.32817	0.58697
H	4.48523	-3.43492	0.62246
H	5.46505	-2.04517	1.15044
C	4.61518	-1.84011	-0.85114
H	5.19350	-0.89556	-0.93425
H	5.11022	-2.57424	-1.50920
N	3.23554	-1.66187	-1.24733
C	2.97098	-1.16385	-2.56889
H	1.88319	-1.09499	-2.72498
C	3.03990	-2.10474	2.51606
N	-3.12779	-1.86168	1.24192
C	-4.48542	-2.13424	0.82418
H	-5.12787	-1.23103	0.89460
H	-4.94001	-2.89894	1.47658
C	-4.37068	-2.62066	-0.61091
H	-4.21883	-3.71959	-0.64048
H	-5.28677	-2.40490	-1.18970
N	-3.23100	-1.93277	-1.18040
C	-2.83903	-2.30116	-2.51434
H	-2.65854	-3.38990	-2.61022
C	-2.91825	-1.34846	2.56751
H	-3.42610	-0.37652	2.73213
C	-3.59446	1.03342	-0.22879
H	-3.98388	1.31481	0.76326
H	-4.29662	0.34217	-0.71215
H	-3.52207	1.94601	-0.83326
C	3.53964	1.25919	0.22322
H	3.90449	1.56144	-0.77209
H	4.28654	0.61396	0.70319
H	3.41539	2.16725	0.82599
H	-3.61824	-2.02056	-3.24275
H	3.38447	-1.84480	-3.33021
H	2.07529	-1.65980	2.80025
H	2.95369	-3.20353	2.62663
H	3.80216	-1.75095	3.23057
H	-3.29432	-2.05786	3.32227
H	-1.84048	-1.20395	2.74006
H	-1.91148	-1.77937	-2.79104
H	3.40717	-0.15859	-2.73927
C	-0.17759	4.59884	-0.00318
F	-0.87586	5.02328	1.05234
F	1.01287	5.17785	0.03655
F	-0.80601	5.02785	-1.09919

(I1-H)$^+$_Fe_L6

Fe	-0.00000	-1.25607	-0.00276
P	-2.13981	-1.07081	0.00056
N	-2.30728	0.69872	-0.00647
C	-1.14548	1.39435	-0.00386
N	-1.18316	2.73069	-0.00080
C	0.00002	3.32415	0.00012
N	1.18319	2.73068	-0.00069
C	1.14548	1.39434	-0.00363
N	-0.00000	0.67100	-0.00428
N	2.30728	0.69871	-0.00585
P	2.13982	-1.07082	0.00038
C	-3.55665	1.42890	0.00066
H	-3.65237	2.04715	0.90598
H	-4.39425	0.72252	-0.03147
H	-3.62831	2.09917	-0.86843
C	3.55663	1.42895	0.00071
H	3.62949	2.09673	-0.87023
H	4.39429	0.72252	-0.02818
H	3.65103	2.04982	0.90434
H	0.00002	4.42124	0.00343
C	3.23471	-1.50851	1.41385
H	2.77921	-1.14524	2.34523
H	3.29344	-2.60588	1.46923
H	4.25671	-1.10934	1.31705
C	-3.23447	-1.50767	1.41449
H	-3.29372	-2.60499	1.47023
H	-2.77855	-1.14431	2.34563
H	-4.25628	-1.10799	1.31781
C	-3.24131	-1.52281	-1.40305
H	-2.79527	-1.15990	-2.33914
H	-3.29001	-2.62095	-1.45298
H	-4.26662	-1.13353	-1.30167
C	3.24105	-1.52203	-1.40370
H	4.26619	-1.13226	-1.30251
H	3.29025	-2.62013	-1.45397
H	2.79457	-1.15905	-2.33955

(I1-H)$^+$_Fe_L7

Fe	0.01303	-0.86739	-0.66611
P	-2.02223	-0.77866	-0.15011
N	-2.29574	0.96998	-0.08862
C	-1.16373	1.71044	-0.10013
N	-1.21874	3.03103	0.08360
C	-0.04289	3.63564	0.14587
N	1.14467	3.05369	0.13307
C	1.12703	1.73095	-0.04598
N	-0.00718	1.02581	-0.28221
N	2.26721	1.00574	0.02913
P	2.03904	-0.73818	-0.12796
O	2.82395	-1.21675	1.27503
C	4.01438	-1.93642	1.04500
H	3.80778	-3.02082	1.07550
H	4.73512	-1.70031	1.84228

C	4.51443	-1.50661	-0.32983
H	5.19410	-0.63923	-0.26027
H	5.04441	-2.31583	-0.85222
O	3.35996	-1.16369	-1.06830
O	-2.71514	-1.29548	1.28182
C	-4.10295	-1.54023	1.19300
H	-4.66991	-0.64937	1.51619
H	-4.36013	-2.37001	1.86633
C	-4.37380	-1.87893	-0.26986
H	-4.29870	-2.96476	-0.45167
H	-5.37079	-1.54371	-0.59534
O	-3.38924	-1.20432	-1.02381
C	-3.57895	1.59743	0.13720
H	-3.59206	2.59033	-0.32754
H	-3.78781	1.72172	1.21215
H	-4.36313	0.98118	-0.32041
C	3.54525	1.61851	0.31341
H	4.22722	1.50918	-0.54335
H	3.99829	1.14641	1.19772
H	3.39927	2.68502	0.51619
H	-0.05528	4.72846	0.23978

(I1-H)$^{+}$_Fe_L8

Fe	0.00000	1.10274	-0.00000
N	2.05706	0.85690	-0.03058
C	2.27948	-0.47850	0.57251
C	1.13086	-1.37353	0.23417
N	1.16586	-2.68259	0.22487
C	0.00000	-3.30722	-0.00001
N	-1.16586	-2.68259	-0.22486
C	-1.13086	-1.37353	-0.23417
N	0.00000	-0.65085	-0.00000
C	-2.27949	-0.47850	-0.57251
N	-2.05706	0.85690	0.03058
H	-0.00001	-4.40003	0.00003
H	-2.30105	-0.34969	-1.66846
H	3.24923	-0.91663	0.26952
H	-3.24923	-0.91663	-0.26952
H	2.30105	-0.34969	1.66846
C	-2.79961	1.87677	-0.70140
H	-2.63400	2.85512	-0.22755
H	-2.43349	1.92586	-1.73582
H	-3.88841	1.66452	-0.70333
C	2.48751	0.83662	-1.43068
H	1.96173	0.04001	-1.97244
H	2.23757	1.79232	-1.90967
H	3.58113	0.66418	-1.50052
C	-2.48750	0.83662	1.43068
H	-1.96173	0.04001	1.97244
H	-2.23756	1.79232	1.90967
H	-3.58113	0.66418	1.50053
C	2.79961	1.87677	0.70140
H	3.88841	1.66452	0.70333
H	2.63400	2.85512	0.22755
H	2.43349	1.92586	1.73582

(I1-H)$^{+}$_Fe_L9

Fe	0.00000	-1.27243	-0.00037
P	2.12280	-1.04463	-0.00014
N	2.39025	0.68351	0.00150
C	1.19908	1.42490	0.00069
C	1.21220	2.83134	0.00047
C	-0.00000	3.52029	-0.00024
C	-1.21220	2.83134	-0.00071
C	-1.19908	1.42490	-0.00064
C	-0.00000	0.67322	0.00006
N	-2.39025	0.68351	-0.00117
P	-2.12280	-1.04463	0.00009
C	3.65827	1.33898	0.00112
H	3.79835	1.98222	-0.89122
H	4.47235	0.60149	0.00239
H	3.79760	1.98424	0.89208
C	-3.65827	1.33898	-0.00097
H	-3.79798	1.98302	0.89085
H	-4.47235	0.60149	-0.00118
H	-3.79797	1.98344	-0.89245
H	-0.00000	4.61555	-0.00040
C	-3.22553	-1.57086	-1.39912
H	-2.78103	-1.21622	-2.34011
H	-3.24958	-2.67137	-1.42498
H	-4.26031	-1.19532	-1.31270
C	3.22461	-1.56842	-1.40102
H	3.24880	-2.66888	-1.42884
H	2.77943	-1.21219	-2.34109
H	4.25939	-1.19284	-1.31463
C	3.22473	-1.57100	1.39963
H	2.77953	-1.21672	2.34042
H	3.24909	-2.67151	1.42523
H	4.25946	-1.19512	1.31396
C	-3.22381	-1.56856	1.40153
H	-4.25853	-1.19265	1.31589
H	-3.24831	-2.66902	1.42909
H	-2.77793	-1.21270	2.34140
H	2.15083	3.39271	0.00082
H	-2.15083	3.39271	-0.00122

(I1-H)$^{+}$_Fe_L10

Fe	0.00002	1.32278	0.00001
O	-2.05977	0.96633	0.27605
C	-2.42300	-0.35476	-0.12317
C	-1.20602	-1.22311	-0.03514
C	-1.21406	-2.61441	-0.02321
C	0.00010	-3.31824	0.00001
C	1.21424	-2.61436	0.02322
C	1.20612	-1.22306	0.03514
C	0.00004	-0.49463	0.00000
C	2.42303	-0.35462	0.12315
O	2.05963	0.96643	-0.27607
H	0.00013	-4.41279	0.00001
H	2.80329	-0.30477	1.16660
H	-3.26084	-0.70580	0.51522
H	3.26090	-0.70558	-0.51525
H	-2.80325	-0.30495	-1.16662
C	3.04150	1.91843	0.00570
H	2.69757	2.87776	-0.40458
H	3.18300	2.02943	1.09843
H	4.00655	1.63323	-0.46132
C	-3.04177	1.91821	-0.00571
H	-4.00679	1.63287	0.46132
H	-2.69797	2.87758	0.40457
H	-3.18330	2.02919	-1.09844
H	-2.16199	-3.16865	-0.03884
H	2.16219	-3.16854	0.03885

(I1-H)$^{+}$_Fe_L11

Fe	0.00001	1.17626	0.00000
N	2.09871	0.87042	-0.04331
C	2.31854	-0.44552	0.60967
C	1.18010	-1.36341	0.27113
C	1.19057	-2.75415	0.25627

C	-0.00001	-3.45703	-0.00000
C	-1.19059	-2.75415	-0.25627
C	-1.18011	-1.36340	-0.27114
C	-0.00000	-0.64135	-0.00001
C	-2.31854	-0.44550	-0.60968
N	-2.09870	0.87043	0.04331
H	-0.00002	-4.55189	-0.00000
H	-2.33174	-0.25239	-1.69750
H	3.32046	-0.84754	0.33676
H	-3.32046	-0.84752	-0.33677
H	2.33174	-0.25242	1.69749
C	-2.88163	1.90104	-0.61315
H	-2.71128	2.86338	-0.10760
H	-2.54804	2.00432	-1.65590
H	-3.97156	1.66298	-0.58708
C	2.47384	0.78900	-1.44972
H	1.91628	-0.02412	-1.93274
H	2.20754	1.72753	-1.95583
H	3.56829	0.60154	-1.55298
C	-2.47383	0.78901	1.44972
H	-1.91628	-0.02412	1.93274
H	-2.20753	1.72753	1.95584
H	-3.56829	0.60154	1.55298
C	2.88164	1.90102	0.61316
H	3.97157	1.66295	0.58709
H	2.71129	2.86336	0.10762
H	2.54805	2.00429	1.65591
H	2.11608	-3.31067	0.45597
H	-2.11611	-3.31066	-0.45595

(I1-H)$^{+}$_Ni_L1

Ni	0.00000	-0.65132	0.00000
P	-2.23300	-0.50931	-0.02383
N	-2.32853	1.22507	-0.06028
C	-1.16377	1.90923	-0.02787
N	-1.18089	3.23186	-0.02499
C	-0.00000	3.83322	-0.00000
N	1.18089	3.23186	0.02499
C	1.16377	1.90923	0.02787
N	0.00000	1.19321	-0.00000
N	2.32854	1.22507	0.06028
P	2.23300	-0.50931	0.02383
N	3.23037	-1.09780	1.21021
C	4.21595	-2.04997	0.69071
H	3.86286	-3.08703	0.82810
H	5.15480	-1.94172	1.25429
C	4.39681	-1.70614	-0.78078
H	5.20360	-0.96501	-0.93124
H	4.64405	-2.59211	-1.38264
N	3.11558	-1.14694	-1.22758
C	2.99352	-0.68993	-2.59794
H	1.98502	-0.29111	-2.78076
C	2.94941	-1.05457	2.63030
N	-3.11558	-1.14694	1.22758
C	-4.39682	-1.70613	0.78078
H	-5.20360	-0.96501	0.93124
H	-4.64405	-2.59210	1.38265
C	-4.21595	-2.04997	-0.69071
H	-3.86286	-3.08703	-0.82809
H	-5.15480	-1.94172	-1.25429
N	-3.23037	-1.09781	-1.21021
C	-2.94941	-1.05458	-2.63030
H	-2.58144	-2.02300	-3.00701
C	-2.99352	-0.68992	2.59794
H	-3.72705	0.10383	2.82338
C	-3.60011	1.94847	-0.06438
H	-3.64253	2.63817	-0.91629
H	-3.71479	2.52649	0.86179
H	-4.41474	1.22121	-0.15176
C	3.60011	1.94847	0.06438
H	3.71479	2.52650	-0.86178
H	4.41474	1.22121	0.15174
H	3.64253	2.63816	0.91630
H	-0.00000	4.92907	-0.00000
H	-3.86332	-0.79463	-3.18364
H	3.16018	-1.52444	-3.29265
H	2.19821	-0.28092	2.84633
H	2.58144	-2.02300	3.00702
H	3.86332	-0.79463	3.18364
H	-3.16017	-1.52444	3.29265
H	-1.98502	-0.29111	2.78076
H	-2.19821	-0.28093	-2.84633
H	3.72705	0.10383	-2.82338

(I1-H)$^{+}$_Ni_L2

Ni	-0.00000	-0.58809	-0.08205
P	-2.23648	-0.42370	-0.03192
O	-2.28924	1.30524	-0.10157
C	-1.16330	1.96943	-0.09943
N	-1.18203	3.27728	-0.11009
C	0.00000	3.88415	-0.11481
N	1.18203	3.27728	-0.11009
C	1.16330	1.96943	-0.09943
N	0.00000	1.25093	-0.08945
O	2.28924	1.30524	-0.10157
P	2.23648	-0.42370	-0.03192
N	3.08061	-0.92646	1.28145
C	4.47523	-1.24506	0.92303
H	4.83697	-2.05780	1.56780
H	5.11085	-0.35909	1.09640
C	4.45272	-1.64578	-0.54640
H	5.37583	-1.34580	-1.06404
H	4.32750	-2.73305	-0.67927
N	3.31511	-0.93352	-1.15039
C	3.16667	-0.87379	-2.59174
H	2.26405	-0.30676	-2.86028
C	2.77839	-0.53095	2.64615
N	-3.08061	-0.92646	1.28145
C	-4.47523	-1.24506	0.92303
H	-5.11085	-0.35909	1.09640
H	-4.83697	-2.05780	1.56780
C	-4.45272	-1.64578	-0.54640
H	-4.32750	-2.73305	-0.67927
H	-5.37583	-1.34580	-1.06404
N	-3.31511	-0.93352	-1.15039
C	-3.16667	-0.87379	-2.59174
H	-3.09455	-1.88238	-3.02629
C	-2.77839	-0.53095	2.64615
H	-3.35120	0.36447	2.93754
H	0.00000	4.97975	-0.12341
H	-4.03077	-0.35948	-3.03667
H	3.09455	-1.88238	-3.02629
H	1.70612	-0.30885	2.75220
H	3.02577	-1.34927	3.33549
H	3.35120	0.36447	2.93754
H	-3.02577	-1.34927	3.33549
H	-1.70612	-0.30885	2.75220
H	-2.26405	-0.30675	-2.86028
H	4.03077	-0.35948	-3.03667

(I1-H)$^{+}$_Ni_L3

Ni	0.00005	-0.45943	0.00054
P	2.24543	-0.34275	-0.11523
C	2.34090	1.40129	-0.73401
C	1.11164	2.13568	-0.33380
N	1.13113	3.45189	-0.32327
C	-0.00004	4.06891	-0.00077
N	-1.13120	3.45202	0.32202
C	-1.11169	2.13581	0.33318
N	-0.00001	1.42063	-0.00013
C	-2.34097	1.40158	0.73359
P	-2.24541	-0.34277	0.11569
N	-3.27196	-0.51149	-1.19267
C	-4.14863	-1.67548	-1.02810
H	-3.69867	-2.57556	-1.48566
H	-5.10353	-1.48125	-1.53862
C	-4.33247	-1.84272	0.46989
H	-5.15653	-1.21042	0.84812
H	-4.54983	-2.88432	0.74613
N	-3.06056	-1.42957	1.07718
C	-2.89812	-1.53912	2.51206
H	-1.88847	-1.22192	2.81259
C	-2.95613	-0.05106	-2.52865
N	3.05954	-1.43037	-1.07674
C	4.33171	-1.84365	-0.47006
H	5.15573	-1.21179	-0.84909
H	4.54856	-2.88545	-0.74594
C	4.14884	-1.67563	1.02796
H	3.69874	-2.57529	1.48621
H	5.10413	-1.48160	1.53784
N	3.27283	-0.51114	1.19245
C	2.95854	-0.04931	2.52831
H	2.42322	-0.81138	3.12143
C	2.89631	-1.54021	-2.51151
H	3.63408	-0.92467	-3.05518
H	-0.00003	5.16444	-0.00094
H	3.88749	0.20652	3.05695
H	-3.02645	-2.58410	2.82654
H	-2.34188	0.86113	-2.48673
H	-2.42022	-0.81378	-3.12041
H	-3.88446	0.20430	-3.05858
H	3.02432	-2.58529	-2.82582
H	1.88652	-1.22296	-2.81155
H	2.34429	0.86287	2.48613
H	-3.63611	-0.92335	3.05519
H	-2.36796	1.34385	1.83860
H	3.24207	1.95174	-0.42504
H	-3.24211	1.95182	0.42415
H	2.36770	1.34293	-1.83899

(I1-H)$^{+}$_Ni_L4

Ni	-0.00000	-0.59529	-0.05586
P	2.21422	-0.50103	-0.02131
N	2.34776	1.21099	-0.07867
C	1.18717	1.94695	-0.08449
C	1.20615	3.34002	-0.09812
C	0.00000	4.02336	-0.10570
C	-1.20615	3.34002	-0.09812
C	-1.18717	1.94695	-0.08449
N	-0.00000	1.26673	-0.07523
N	-2.34776	1.21099	-0.07867
P	-2.21422	-0.50103	-0.02131
N	-3.25154	-1.14336	-1.15020
C	-4.15505	-2.14278	-0.57816
H	-3.75175	-3.16088	-0.72227
H	-5.12436	-2.09501	-1.09690
C	-4.28471	-1.79374	0.89748
H	-5.12204	-1.09411	1.07954
H	-4.45946	-2.68521	1.51666
N	-3.01616	-1.16560	1.27575
C	-2.84798	-0.69199	2.63407
H	-2.92615	-1.52808	3.34272
C	-3.03885	-1.10559	-2.58156
H	-2.63846	-2.05873	-2.96497
N	3.25154	-1.14336	-1.15020
C	4.15505	-2.14278	-0.57816
H	5.12436	-2.09501	-1.09690
H	3.75175	-3.16088	-0.72227
C	4.28471	-1.79374	0.89748
H	4.45946	-2.68521	1.51666
H	5.12204	-1.09411	1.07954
N	3.01616	-1.16560	1.27575
C	2.84799	-0.69199	2.63406
H	3.61449	0.05833	2.89688
C	3.03885	-1.10559	-2.58156
H	3.98946	-0.90182	-3.09542
C	3.64913	1.86656	-0.05593
H	3.79241	2.49372	-0.94766
H	4.43125	1.09984	-0.05904
H	3.76623	2.48192	0.84826
C	-3.64913	1.86656	-0.05593
H	-3.76623	2.48192	0.84826
H	-4.43125	1.09984	-0.05904
H	-3.79241	2.49372	-0.94766
H	-3.98946	-0.90181	-3.09542
H	-2.33889	-0.29804	-2.84069
H	-1.85730	-0.23051	2.75901
H	-3.61449	0.05832	2.89688
H	2.33889	-0.29804	-2.84069
H	2.63846	-2.05873	-2.96497
H	2.92615	-1.52808	3.34272
H	1.85730	-0.23051	2.75901
H	0.00000	5.11502	-0.11745
H	-2.15269	3.87615	-0.10326
H	2.15269	3.87615	-0.10326

(I1-H)$^{+}$_Ni_L5

Ni	-0.06762	-1.36330	0.00288
P	2.17073	-1.31997	0.02386
N	2.34239	0.41071	0.05880
C	1.20999	1.14286	0.02711
N	1.28266	2.46253	0.02380
C	0.12784	3.10720	-0.00202
N	-1.07560	2.56920	-0.02727
C	-1.11753	1.24365	-0.02885
N	0.01399	0.47983	-0.00010
N	-2.31036	0.61772	-0.06029
P	-2.29502	-1.12456	-0.02118
N	-3.31643	-1.66392	-1.20863
C	-4.35166	-2.56318	-0.68944
H	-4.05352	-3.61701	-0.82821
H	-5.28296	-2.40472	-1.25362
C	-4.51528	-2.21164	0.78248
H	-5.28598	-1.43358	0.93461
H	-4.80297	-3.08530	1.38432
N	-3.20872	-1.71314	1.22974
C	-3.06282	-1.26841	2.60214
H	-2.03774	-0.91380	2.78375
C	-3.03477	-1.63300	-2.62926
N	3.01949	-1.99724	-1.22822
C	4.27370	-2.61731	-0.78339
H	5.11469	-1.91690	-0.94037
H	4.47455	-3.51646	-1.38290
C	4.08214	-2.94661	0.69030
H	3.68315	-3.96590	0.83384

H	5.02658	-2.87799	1.25067
N	3.14215	-1.94851	1.20960
C	2.87389	-1.88111	2.63151
H	2.46513	-2.82876	3.01844
C	2.91143	-1.54570	-2.60189
H	3.68745	-0.79765	-2.84019
C	3.64453	1.08003	0.06471
H	3.77570	1.66731	-0.85323
H	4.42803	0.31763	0.13386
H	3.72155	1.75372	0.92706
C	-3.54860	1.39881	-0.06593
H	-3.63333	1.98762	0.85649
H	-4.39514	0.70820	-0.14520
H	-3.56271	2.08360	-0.92269
H	3.80308	-1.65887	3.17571
H	-3.26296	-2.09836	3.29344
H	-2.24422	-0.89958	-2.84517
H	-2.71867	-2.61888	-3.00740
H	-3.93442	-1.32451	-3.18098
H	3.02369	-2.39413	-3.29061
H	1.92621	-1.09049	-2.77984
H	2.16052	-1.07228	2.84675
H	-3.76117	-0.44532	2.83333
C	0.23964	4.63962	-0.00264
F	0.92527	5.01222	-1.07118
F	-0.94610	5.20354	-0.02306
F	0.88984	5.01661	1.08606

(I1-H)$^+$_Ni_L6

Ni	0.00000	1.17248	0.00013
P	2.24812	1.00394	0.00078
N	2.33248	-0.71634	-0.00374
C	1.16425	-1.39360	-0.00162
N	1.17797	-2.71237	-0.00097
C	-0.00001	-3.31862	-0.00001
N	-1.17799	-2.71236	0.00068
C	-1.16426	-1.39359	0.00114
N	0.00000	-0.66891	-0.00018
N	-2.33248	-0.71633	0.00299
P	-2.24812	1.00395	-0.00061
C	3.59628	-1.46559	-0.00121
H	3.66951	-2.08236	0.90323
H	4.43493	-0.76075	-0.02547
H	3.64941	-2.11836	-0.88114
C	-3.59626	-1.46563	0.00093
H	-3.66778	-2.08544	-0.90153
H	-4.43498	-0.76073	0.02115
H	-3.65098	-2.11544	0.88297
H	-0.00002	-4.41432	-0.00014
C	-3.16015	1.57990	1.45054
H	-2.71164	1.18615	2.37243
H	-3.13528	2.68003	1.47759
H	-4.21370	1.26593	1.38908
C	3.16139	1.57131	1.45456
H	3.14385	2.67147	1.48437
H	2.70958	1.17799	2.37502
H	4.21276	1.24991	1.39297
C	3.16000	1.58082	-1.45010
H	2.71182	1.18704	-2.37214
H	3.13438	2.68093	-1.47685
H	4.21377	1.26758	-1.38867
C	-3.16122	1.57227	-1.45412
H	-4.21282	1.25162	-1.39258
H	-3.14294	2.67242	-1.48362
H	-2.70974	1.17893	-2.37473

(I1-H)$^+$_Ni_L7

Ni	0.00000	0.83624	0.00000
P	2.22176	0.64531	-0.01535
N	2.33771	-1.04870	-0.05053
C	1.16543	-1.73628	-0.02318
N	1.17734	-3.05230	-0.02193
C	0.00000	-3.65942	0.00003
N	-1.17734	-3.05230	0.02193
C	-1.16543	-1.73628	0.02317
N	0.00000	-1.01578	-0.00000
N	-2.33771	-1.04870	0.05052
P	-2.22176	0.64531	0.01535
O	-3.15648	1.18417	1.20077
C	-4.18614	2.08035	0.70366
H	-3.84710	3.11360	0.86028
H	-5.09021	1.90157	1.29749
C	-4.36303	1.72996	-0.77008
H	-5.10657	0.93635	-0.93213
H	-4.61413	2.59571	-1.39258
O	-3.07014	1.23124	-1.21087
O	3.07015	1.23124	1.21087
C	4.36303	1.72996	0.77007
H	5.10658	0.93634	0.93213
H	4.61413	2.59570	1.39259
C	4.18614	2.08036	-0.70366
H	3.84710	3.11360	-0.86027
H	5.09020	1.90157	-1.29749
O	3.15647	1.18417	-1.20077
C	3.64014	-1.72938	-0.04283
H	3.49538	-2.77233	-0.34118
H	4.07758	-1.69670	0.96399
H	4.30277	-1.23840	-0.76634
C	-3.64014	-1.72938	0.04282
H	-4.07758	-1.69670	-0.96400
H	-4.30277	-1.23841	0.76632
H	-3.49538	-2.77233	0.34116
H	-0.00000	-4.75518	-0.00004

(I1-H)$^+$_Ni_L8

Ni	0.00000	1.01362	0.00000
N	1.93593	0.85120	-0.01952
C	2.31718	-0.54181	0.37881
C	1.15204	-1.44779	0.15794
N	1.17861	-2.75659	0.15547
C	-0.00001	-3.36965	0.00002
N	-1.17862	-2.75659	-0.15547
C	-1.15204	-1.44778	-0.15794
N	-0.00000	-0.77744	-0.00000
C	-2.31718	-0.54180	-0.37881
N	-1.93592	0.85120	0.01952
H	0.00000	-4.46521	-0.00003
H	-2.56991	-0.56789	-1.45249
H	3.21481	-0.88663	-0.16018
H	-3.21481	-0.88663	0.16017
H	2.56991	-0.56790	1.45248
C	-2.54597	1.85369	-0.88099
H	-2.27604	2.86407	-0.54267
H	-2.19891	1.69775	-1.91110
H	-3.64503	1.76918	-0.85600
C	2.33446	1.10496	-1.42615
H	1.92098	0.32851	-2.08390
H	1.97101	2.09141	-1.74676
H	3.43325	1.09892	-1.51110
C	-2.33446	1.10496	1.42615
H	-1.92099	0.32850	2.08390
H	-1.97101	2.09141	1.74676
H	-3.43325	1.09891	1.51109

C	2.54598	1.85369	0.88099
H	3.64504	1.76917	0.85600
H	2.27605	2.86406	0.54268
H	2.19892	1.69774	1.91111

(I1-H)$^{+}$_Ni_L9

Ni	0.00000	-1.15497	0.00001
P	2.20708	-0.98867	-0.00049
N	2.41145	0.69321	0.00150
C	1.21928	1.42083	0.00078
C	1.21394	2.82064	0.00075
C	0.00000	3.49625	0.00013
C	-1.21394	2.82064	-0.00061
C	-1.21928	1.42083	-0.00083
C	-0.00000	0.71359	-0.00005
N	-2.41145	0.69321	-0.00170
P	-2.20708	-0.98867	0.00048
C	3.69734	1.35567	0.00118
H	3.82387	1.98533	-0.89403
H	4.50413	0.61258	0.00193
H	3.82350	1.98654	0.89554
C	-3.69734	1.35567	-0.00126
H	-3.82407	1.98486	0.89424
H	-4.50413	0.61258	-0.00264
H	-3.82330	1.98700	-0.89533
H	0.00000	4.58826	0.00022
C	-3.12541	-1.63945	-1.43001
H	-2.69685	-1.23897	-2.35827
H	-3.05251	-2.73705	-1.44638
H	-4.19032	-1.36835	-1.37258
C	3.12387	-1.63573	-1.43370
H	3.05106	-2.73329	-1.45299
H	2.69427	-1.23277	-2.36042
H	4.18880	-1.36461	-1.37665
C	3.12547	-1.63928	1.43004
H	2.69687	-1.23880	2.35829
H	3.05271	-2.73689	1.44647
H	4.19035	-1.36805	1.37258
C	-3.12393	-1.63556	1.43373
H	-4.18883	-1.36431	1.37665
H	-3.05126	-2.73312	1.45307
H	-2.69430	-1.23261	2.36043
H	2.14952	3.38054	0.00123
H	-2.14952	3.38055	-0.00097

(I1-H)$^{+}$_Ni_L10

Ni	-0.00000	-1.18881	-0.00004
O	-1.89052	-1.00168	-0.00006
C	-2.41108	0.34636	0.00002
C	-1.22773	1.25261	0.00002
C	-1.22074	2.64814	0.00002
C	0.00000	3.32610	0.00002
C	1.22074	2.64814	0.00000
C	1.22773	1.25261	0.00000
C	0.00000	0.61370	0.00001
C	2.41108	0.34636	-0.00000
O	1.89052	-1.00168	-0.00011
H	0.00000	4.41777	0.00002
H	3.04774	0.47105	-0.89396
H	-3.04773	0.47104	-0.89394
H	3.04764	0.47095	0.89404
H	-3.04765	0.47096	0.89406
C	2.87982	-2.02259	0.00011
H	2.36972	-2.99283	-0.00012
H	3.50494	-1.93814	-0.90151
H	3.50441	-1.93828	0.90212
C	-2.87982	-2.02259	0.00006
H	-3.50482	-1.93816	-0.90165
H	-2.36972	-2.99283	-0.00009
H	-3.50452	-1.93826	0.90199
H	-2.15891	3.20865	0.00003
H	2.15891	3.20865	-0.00001

(I1-H)$^{+}$_Ni_L11

Ni	0.00000	1.03249	-0.00000
N	1.94456	0.87691	-0.03753
C	2.33036	-0.46879	0.50392
C	1.20582	-1.41542	0.22788
C	1.20314	-2.81049	0.21968
C	-0.00001	-3.48803	0.00000
C	-1.20316	-2.81048	-0.21968
C	-1.20583	-1.41541	-0.22789
C	-0.00000	-0.77351	-0.00001
C	-2.33037	-0.46877	-0.50393
N	-1.94455	0.87692	0.03753
H	-0.00001	-4.58005	0.00001
H	-2.47902	-0.35925	-1.59042
H	3.30073	-0.77741	0.07626
H	-3.30074	-0.77739	-0.07626
H	2.47902	-0.35927	1.59041
C	-2.59171	1.95027	-0.72737
H	-2.33517	2.92477	-0.28837
H	-2.25506	1.92267	-1.77207
H	-3.68958	1.83906	-0.70163
C	2.34064	0.96645	-1.45622
H	1.92603	0.11645	-2.01181
H	1.96365	1.89972	-1.89602
H	3.44071	0.95640	-1.54326
C	-2.34063	0.96645	1.45622
H	-1.92602	0.11644	2.01180
H	-1.96364	1.89972	1.89603
H	-3.44070	0.95641	1.54326
C	2.59172	1.95025	0.72738
H	3.68959	1.83905	0.70164
H	2.33518	2.92476	0.28838
H	2.25506	1.92265	1.77207
H	2.12483	-3.37319	0.38847
H	-2.12485	-3.37318	-0.38846

4.4. Intermediates

4.4.1. I1

I1_Co_L1

Co	0.00001	-0.69248	0.00012
H	0.00001	-2.22189	0.00043
P	2.09174	-0.55482	0.03397
N	2.29372	1.22783	0.14299
C	1.14816	1.94594	0.06881
N	1.18434	3.28420	0.06642
C	-0.00004	3.87262	0.00017
N	-1.18440	3.28418	-0.06623
C	-1.14816	1.94593	-0.06887
N	0.00000	1.23619	-0.00003
N	-2.29369	1.22780	-0.14332
P	-2.09174	-0.55482	-0.03392
N	-3.18052	-1.10738	-1.23274
C	-4.28111	-1.87728	-0.68453
H	-4.03036	-2.95828	-0.66743
H	-5.18571	-1.75830	-1.30599
C	-4.49181	-1.36304	0.72853
H	-5.17622	-0.48828	0.74208
H	-4.94177	-2.13356	1.37719
N	-3.16823	-1.02207	1.19983
C	-3.00989	-0.53879	2.54209
H	-1.94260	-0.36842	2.74953
C	-2.66213	-1.54636	-2.50518
N	3.16814	-1.02223	-1.19979
C	4.49178	-1.36302	-0.72852
H	5.17609	-0.48817	-0.74216
H	4.94181	-2.13354	-1.37712
C	4.28119	-1.87712	0.68459
H	4.03044	-2.95812	0.66760
H	5.18583	-1.75808	1.30599
N	3.18060	-1.10720	1.23282
C	2.66225	-1.54629	2.50524
H	2.32784	-2.60150	2.47657
C	3.00970	-0.53908	-2.54209
H	3.55159	0.41151	-2.72093
C	3.57553	1.88745	0.22967
H	3.48616	2.81925	0.80204
H	3.97812	2.13926	-0.76553
H	4.27854	1.21930	0.74482
C	-3.57548	1.88740	-0.23032
H	-3.97810	2.13975	0.76474
H	-4.27846	1.21900	-0.74514
H	-3.48610	2.81892	-0.80315
H	-0.00006	4.97020	0.00032
H	3.43265	-1.44551	3.28672
H	-3.38194	-1.28058	3.26734
H	-1.79996	-0.92830	-2.79380
H	-2.32770	-2.60157	-2.47659
H	-3.43250	-1.44554	-3.28669
H	3.38107	-1.28119	-3.26736
H	1.94246	-0.36806	-2.74921
H	1.80007	-0.92827	2.79394
H	-3.55125	0.41215	2.72065

I1_Co_L2

Co	0.00003	-0.58779	0.36143
H	0.00015	-2.01066	0.90215
P	-2.08046	-0.49253	0.21446
O	-2.26683	1.21709	-0.38628
C	-1.15259	1.85834	-0.59626
N	-1.18840	3.10322	-1.05784
C	-0.00018	3.65817	-1.25598
N	1.18810	3.10296	-1.05882
C	1.15237	1.85813	-0.59715
N	-0.00005	1.20373	-0.33006
O	2.26671	1.21665	-0.38805
P	2.08057	-0.49227	0.21413
N	3.30056	-0.44389	1.38329
C	4.60890	-0.54289	0.75384
H	5.35085	-0.91413	1.47850
H	4.94842	0.45350	0.40343
C	4.42870	-1.49585	-0.41713
H	5.14333	-1.27962	-1.22951
H	4.58622	-2.54730	-0.10552
N	3.05995	-1.31761	-0.86333
C	2.55966	-2.14128	-1.93037
H	1.46806	-2.03089	-2.00337
C	3.18247	0.47439	2.48914
N	-3.30069	-0.44513	1.38335
C	-4.60889	-0.54314	0.75350
H	-4.94820	0.45373	0.40427
H	-5.35106	-0.91514	1.47755
C	-4.42868	-1.49473	-0.41863
H	-4.58699	-2.54649	-0.10848
H	-5.14280	-1.27701	-1.23106
N	-3.05960	-1.31683	-0.86399
C	-2.55925	-2.14023	-1.93121
H	-2.78081	-3.20670	-1.74545
C	-3.18263	0.47175	2.49036
H	-3.46923	1.50292	2.20741
H	-0.00022	4.69008	-1.62798
H	-3.00271	-1.86003	-2.90135
H	2.78149	-3.20765	-1.74446
H	2.14401	0.48910	2.85054
H	3.82811	0.15047	3.31959
H	3.46920	1.50518	2.20494
H	-3.82839	0.14686	3.32033
H	-2.14420	0.48590	2.85187
H	-1.46771	-2.02955	-2.00436
H	3.00288	-1.86115	-2.90065

I1_Co_L3

Co	-0.01646	-0.56198	-0.15974
H	-0.06447	-2.07707	-0.25816
P	-2.11634	-0.41804	0.00009
C	-2.27261	1.33216	0.69756
C	-1.05334	2.09633	0.32160
N	-1.04993	3.42105	0.38262
C	0.10028	4.01461	0.08804
N	1.21990	3.39627	-0.26625
C	1.15255	2.07428	-0.34580
N	0.02876	1.36992	-0.05470
C	2.33476	1.28243	-0.77226
P	2.09824	-0.47584	-0.11457
N	3.00787	-0.48501	1.34675
C	4.28934	-1.15298	1.19208
H	4.22665	-2.19025	1.58097
H	5.08226	-0.63423	1.75898
C	4.58512	-1.17943	-0.29730

H	5.05752	-0.22689	-0.62663
H	5.27935	-1.99412	-0.56146
N	3.29939	-1.38921	-0.93004
C	3.27175	-1.48522	-2.36280
H	2.23524	-1.62764	-2.70421
C	2.34070	-0.68900	2.60951
N	-3.14790	-1.35552	1.00122
C	-4.52512	-1.15101	0.60550
H	-4.93792	-0.20142	1.01524
H	-5.15900	-1.97000	0.98353
C	-4.49162	-1.12003	-0.91277
H	-4.48808	-2.15691	-1.30764
H	-5.37500	-0.60814	-1.33335
N	-3.26144	-0.44112	-1.28583
C	-2.81250	-0.65427	-2.64006
H	-2.69765	-1.73056	-2.87865
C	-2.86374	-1.48365	2.40242
H	-3.18542	-0.59952	2.99388
H	0.12815	5.10888	0.14402
H	-3.52959	-0.21930	-3.35561
H	3.86142	-2.35254	-2.69938
H	1.34486	-0.22311	2.59067
H	2.20755	-1.76440	2.84291
H	2.91871	-0.22981	3.42835
H	-3.38036	-2.36333	2.81825
H	-1.78153	-1.62482	2.54778
H	-1.83890	-0.16906	-2.79692
H	3.68686	-0.58484	-2.86408
H	2.30281	1.18713	-1.87398
H	-3.18025	1.88715	0.41949
H	3.26862	1.79711	-0.50406
H	-2.27582	1.22923	1.79932

I1_Co_L4

Co	0.00003	-0.66613	-0.15613
H	0.00027	-2.19726	-0.15334
P	2.07789	-0.55134	-0.01567
N	2.31604	1.19857	-0.22547
C	1.17412	1.96013	-0.25308
C	1.20719	3.36244	-0.30159
C	-0.00005	4.04591	-0.32751
C	-1.20729	3.36241	-0.30159
C	-1.17418	1.96011	-0.25306
N	-0.00002	1.28224	-0.22839
N	-2.31608	1.19851	-0.22536
P	-2.07786	-0.55138	-0.01574
N	-3.30678	-1.20083	-1.02775
C	-4.24833	-2.02123	-0.29097
H	-3.89675	-3.07317	-0.23613
H	-5.23205	-2.02720	-0.79182
C	-4.32099	-1.42196	1.10152
H	-5.06037	-0.59272	1.14583
H	-4.63533	-2.17054	1.84894
N	-2.97791	-0.96852	1.37499
C	-2.69328	-0.35656	2.64059
H	-2.94058	-1.04127	3.46829
C	-2.92924	-1.68526	-2.33297
H	-2.48307	-2.69834	-2.29047
N	3.30646	-1.20095	-1.02791
C	4.24852	-2.02095	-0.29131
H	5.23212	-2.02658	-0.79240
H	3.89731	-3.07300	-0.23639
C	4.32134	-1.42163	1.10116
H	4.63585	-2.17015	1.84856
H	5.06065	-0.59233	1.14533
N	2.97825	-0.96831	1.37484
C	2.69351	-0.35691	2.64069
H	3.25923	0.58454	2.79800
C	2.92860	-1.68580	-2.33288
H	3.80805	-1.71814	-2.99609
C	3.61120	1.82569	-0.26052
H	3.71260	2.49528	-1.13070
H	4.37661	1.04797	-0.36176
H	3.81810	2.41336	0.65194
C	-3.61126	1.82561	-0.26048
H	-3.81801	2.41360	0.65182
H	-4.37667	1.04784	-0.36129
H	-3.71278	2.49489	-1.13088
H	-3.80904	-1.71819	-2.99568
H	-2.18803	-1.01038	-2.78473
H	-1.61936	-0.12316	2.70378
H	-3.25931	0.58477	2.79757
H	2.18661	-1.01155	-2.78429
H	2.48323	-2.69921	-2.28999
H	2.94106	-1.04187	3.46810
H	1.61951	-0.12386	2.70399
H	-0.00006	5.13825	-0.36306
H	-2.15459	3.89886	-0.30946
H	2.15449	3.89891	-0.30945

I1_Co_L5

Co	-0.09947	-1.40495	0.03404
H	-0.19817	-2.92967	0.05018
P	1.99860	-1.40861	0.04435
N	2.31418	0.36328	0.14696
C	1.21841	1.14850	0.07664
N	1.33613	2.48182	0.06636
C	0.19041	3.13408	-0.00090
N	-1.02635	2.63537	-0.06325
C	-1.07822	1.29419	-0.05685
N	0.02368	0.51551	0.01784
N	-2.26554	0.65623	-0.12863
P	-2.17914	-1.14206	-0.02076
N	-3.27399	-1.62047	-1.24123
C	-4.44097	-2.30711	-0.71881
H	-4.27263	-3.40370	-0.70558
H	-5.32140	-2.11607	-1.35650
C	-4.64011	-1.78620	0.69380
H	-5.26400	-0.86771	0.70193
H	-5.15076	-2.52777	1.33079
N	-3.30448	-1.53644	1.18999
C	-3.13474	-1.08511	2.54242
H	-2.06218	-0.98317	2.76608
C	-2.76489	-2.08864	-2.50727
N	3.01765	-1.93939	-1.20878
C	4.33225	-2.34625	-0.76237
H	5.05915	-1.50745	-0.79495
H	4.72809	-3.14083	-1.41686
C	4.12578	-2.84389	0.65754
H	3.82739	-3.91245	0.65263
H	5.04631	-2.76116	1.26097
N	3.06975	-2.02429	1.22278
C	2.55536	-2.43173	2.50747
H	2.17918	-3.47282	2.49279
C	2.85709	-1.45372	-2.55071
H	3.44103	-0.53165	-2.74401
C	3.63741	0.93996	0.21823
H	4.05261	1.13956	-0.78319
H	4.29536	0.23864	0.74843
H	3.61075	1.88856	0.76841
C	-3.50528	1.39221	-0.22738
H	-3.92480	1.62447	0.76505
H	-4.22754	0.78563	-0.79017
H	-3.34572	2.33899	-0.75750
H	3.34156	-2.35588	3.27570
H	-3.56028	-1.81464	3.25027

H	-1.84874	-1.54093	-2.77065
H	-2.51911	-3.16779	-2.48221
H	-3.50652	-1.91817	-3.30411
H	3.17826	-2.21618	-3.27845
H	1.79564	-1.23216	-2.73879
H	1.72370	-1.77737	2.80499
H	-3.62025	-0.10669	2.73030
C	0.33147	4.65257	-0.00546
F	1.06355	5.04711	-1.04855
F	-0.83802	5.26981	-0.07086
F	0.95178	5.06394	1.10157

I1_Co_L6

Co	-0.00005	-1.19667	0.00380
H	-0.00027	-2.73234	-0.00042
P	2.09615	-1.03768	-0.00056
N	2.30458	0.72850	0.00856
C	1.14691	1.43636	0.00560
N	1.18550	2.77267	0.00082
C	0.00008	3.36431	-0.00120
N	-1.18538	2.77279	0.00007
C	-1.14694	1.43650	0.00537
N	-0.00006	0.72038	0.00724
N	-2.30468	0.72869	0.00872
P	-2.09618	-1.03744	-0.00008
C	3.56416	1.43974	0.00011
H	3.67787	2.04276	-0.91360
H	4.38999	0.71975	0.05023
H	3.63977	2.12185	0.85954
C	-3.56438	1.43960	-0.00124
H	-3.63993	2.12361	0.85663
H	-4.38999	0.71949	0.05104
H	-3.67878	2.04040	-0.91638
H	0.00014	4.46144	-0.00518
C	-3.14648	-1.52802	-1.42087
H	-2.70425	-1.13950	-2.34798
H	-3.14203	-2.62688	-1.47313
H	-4.18767	-1.18244	-1.32999
C	3.14626	-1.52760	-1.42172
H	3.14532	-2.62655	-1.47222
H	2.70216	-1.14180	-2.34905
H	4.18645	-1.17869	-1.33216
C	3.15778	-1.54639	1.40498
H	2.72794	-1.16303	2.34000
H	3.14533	-2.64564	1.44732
H	4.20085	-1.20872	1.30682
C	-3.15717	-1.54623	1.40583
H	-4.19976	-1.20663	1.30922
H	-3.14662	-2.64561	1.44616
H	-2.72573	-1.16532	2.34108

I1_Co_L7

Co	0.00002	-0.89433	0.00034
H	0.00001	-2.41121	0.00054
P	2.06999	-0.71470	0.00534
N	2.29790	1.02849	0.04490
C	1.14732	1.75341	0.01898
N	1.18437	3.08688	0.01495
C	-0.00003	3.67862	-0.00011
N	-1.18441	3.08685	-0.01506
C	-1.14736	1.75337	-0.01884
N	-0.00001	1.04034	0.00015
N	-2.29791	1.02843	-0.04464
P	-2.06993	-0.71479	-0.00511
O	-3.13771	-1.15533	-1.19871
C	-4.18986	-1.96612	-0.71147
H	-3.91297	-3.03014	-0.80401
H	-5.08756	-1.78172	-1.31855
C	-4.38003	-1.56747	0.74618
H	-5.08211	-0.72145	0.84642
H	-4.74608	-2.39864	1.36435
O	-3.09646	-1.18706	1.20885
O	3.09616	-1.18686	-1.20901
C	4.37977	-1.56756	-0.74674
H	5.08199	-0.72166	-0.84711
H	4.74550	-2.39875	-1.36508
C	4.18996	-1.96632	0.71092
H	3.91277	-3.03026	0.80344
H	5.08788	-1.78223	1.31777
O	3.13818	-1.15524	1.19854
C	3.59743	1.66435	0.04059
H	3.48992	2.71745	0.32266
H	4.05961	1.61373	-0.95777
H	4.25177	1.16387	0.76806
C	-3.59746	1.66426	-0.04089
H	-4.06021	1.61329	0.95718
H	-4.25139	1.16404	-0.76892
H	-3.48978	2.71746	-0.32252
H	-0.00004	4.77545	-0.00021

I1_Co_L8

Co	0.00001	1.07961	-0.00001
H	0.00001	2.65781	0.00009
N	2.01010	0.83054	-0.02360
C	2.29216	-0.53450	0.47806
C	1.13272	-1.43592	0.18697
N	1.17531	-2.75082	0.18001
C	-0.00001	-3.36532	-0.00007
N	-1.17536	-2.75081	-0.17995
C	-1.13275	-1.43591	-0.18698
N	-0.00000	-0.73276	-0.00004
C	-2.29217	-0.53447	-0.47806
N	-2.01009	0.83057	0.02360
H	-0.00006	-4.45918	0.00017
H	-2.39654	-0.48010	-1.57555
H	3.24269	-0.93908	0.08347
H	-3.24271	-0.93904	-0.08346
H	2.39651	-0.48014	1.57555
C	-2.73466	1.82371	-0.76582
H	-2.54177	2.81822	-0.34710
H	-2.36200	1.81399	-1.79845
H	-3.82419	1.61894	-0.75933
C	2.39868	0.93293	-1.43220
H	1.90072	0.14818	-2.01723
H	2.08309	1.90804	-1.82360
H	3.49624	0.82298	-1.54317
C	-2.39866	0.93295	1.43220
H	-1.90071	0.14819	2.01723
H	-2.08305	1.90806	1.82361
H	-3.49622	0.82301	1.54318
C	2.73469	1.82367	0.76583
H	3.82421	1.61888	0.75935
H	2.54182	2.81818	0.34712
H	2.36201	1.81395	1.79846

I1_Co_L9

Co	-0.00000	1.22267	-0.00007
H	-0.00001	2.81262	-0.00032
P	-2.07539	1.00535	0.00007
N	-2.38709	-0.71454	0.00008

C	-1.20000	-1.46594	0.00001
C	-1.21381	-2.87210	-0.00005
C	0.00000	-3.56011	-0.00004
C	1.21381	-2.87210	0.00003
C	1.20000	-1.46594	0.00004
C	0.00000	-0.71789	0.00003
N	2.38709	-0.71454	0.00004
P	2.07539	1.00535	-0.00010
C	-3.66408	-1.35075	-0.00010
H	-3.81502	-1.99272	-0.89163
H	-4.46504	-0.59788	0.00026
H	-3.81487	-1.99340	0.89096
C	3.66408	-1.35075	0.00019
H	3.81486	-1.99301	0.89153
H	4.46504	-0.59788	0.00024
H	3.81504	-1.99310	-0.89106
H	-0.00000	-4.65536	-0.00008
C	3.13307	1.58447	-1.40425
H	2.70241	1.20608	-2.34199
H	3.09538	2.68447	-1.42508
H	4.18528	1.26252	-1.32391
C	-3.13292	1.58447	-1.40421
H	-3.09478	2.68445	-1.42549
H	-2.70242	1.20553	-2.34181
H	-4.18525	1.26296	-1.32374
C	-3.13292	1.58456	1.40430
H	-2.70210	1.20632	2.34203
H	-3.09532	2.68457	1.42500
H	-4.18511	1.26252	1.32414
C	3.13277	1.58456	1.40426
H	4.18510	1.26299	1.32396
H	3.09469	2.68455	1.42541
H	2.70213	1.20575	2.34185
H	-2.15328	-3.43294	-0.00013
H	2.15328	-3.43294	0.00004

I1_Co_L10

Co	-0.00069	-1.28494	0.00010
H	-0.00144	-2.95592	-0.00030
O	2.00314	-0.94384	0.27038
C	2.41155	0.38868	-0.04214
C	1.20621	1.27995	0.01066
C	1.21796	2.67240	0.02143
C	0.00175	3.37344	-0.00039
C	-1.21519	2.67360	-0.02183
C	-1.20485	1.28116	-0.01020
C	0.00033	0.55824	0.00047
C	-2.41100	0.39103	0.04292
O	-2.00418	-0.94172	-0.27059
H	0.00230	4.46812	-0.00078
H	-2.85226	0.38278	1.06355
H	3.21983	0.68966	0.65792
H	-3.21952	0.69317	-0.65635
H	2.85359	0.38062	-1.06242
C	-2.94251	-1.91407	0.08957
H	-2.54241	-2.88201	-0.23532
H	-3.07725	-1.93889	1.18836
H	-3.92090	-1.70921	-0.39008
C	2.94043	-1.91700	-0.09032
H	3.91897	-1.71366	0.38971
H	2.53913	-2.88475	0.23370
H	3.07542	-1.94110	-1.18910
H	2.16356	3.23116	0.03811
H	-2.16022	3.23330	-0.03898

I1_Co_L11

Co	-0.00040	-1.12744	0.00014
H	-0.00061	-2.80437	0.00214
N	-2.03756	-0.84588	-0.03209
C	-2.33431	0.50828	0.50771
C	-1.19054	1.43536	0.20477
C	-1.20277	2.82758	0.19296
C	0.00140	3.52697	0.00038
C	1.20499	2.82669	-0.19270
C	1.19154	1.43451	-0.20581
C	0.00015	0.71607	-0.00097
C	2.33470	0.50656	-0.50831
N	2.03698	-0.84715	0.03213
H	0.00188	4.62202	0.00084
H	2.43286	0.38993	-1.60292
H	-3.32289	0.86041	0.13463
H	3.32348	0.85816	-0.13528
H	-2.43241	0.39212	1.60236
C	2.77425	-1.86266	-0.69899
H	2.56722	-2.84407	-0.25322
H	2.42236	-1.88944	-1.73980
H	3.86975	-1.65883	-0.67976
C	-2.38538	-0.90455	-1.44595
H	-1.89096	-0.08257	-1.97933
H	-2.03021	-1.85519	-1.86672
H	-3.48853	-0.82048	-1.58288
C	2.38465	-0.90540	1.44603
H	1.89085	-0.08278	1.97898
H	2.02872	-1.85556	1.86723
H	3.48788	-0.82206	1.58295
C	-2.77579	-1.86043	0.69942
H	-3.87109	-1.65553	0.68018
H	-2.56971	-2.84220	0.25402
H	-2.42381	-1.88719	1.74020
H	-2.13452	3.38865	0.34657
H	2.13727	3.38708	-0.34554

I1_Fe_L1

Fe	0.01392	-0.62935	-0.76606
H	0.03796	-2.04067	-1.53195
P	1.98466	-0.57956	-0.15142
N	2.25905	1.21016	0.08224
C	1.13301	1.95448	0.00540
N	1.15393	3.27531	0.23875
C	-0.03003	3.86923	0.22898
N	-1.21060	3.28055	0.09047
C	-1.15863	1.96090	-0.13847
N	-0.00044	1.26373	-0.29438
N	-2.29080	1.22569	-0.19913
P	-1.97989	-0.57912	-0.21542
N	-3.45840	-1.09543	-1.01385
C	-4.39623	-1.78472	-0.16717
H	-4.24578	-2.88657	-0.22514
H	-5.44346	-1.58945	-0.47428
C	-4.13188	-1.29593	1.24648
H	-4.71722	-0.37277	1.46580
H	-4.44364	-2.04781	1.99682
N	-2.71557	-1.06433	1.30535
C	-2.19729	-0.50529	2.51519
H	-1.11889	-0.31643	2.38785
C	-3.37447	-1.56761	-2.36575
N	3.45659	-0.98427	-1.00865
C	4.58881	-1.33206	-0.20301
H	5.24249	-0.45881	0.02711
H	5.22909	-2.07245	-0.72079
C	3.99333	-1.91541	1.06783
H	3.80685	-3.00553	0.93474

H	4.68371	-1.81112	1.92851
N	2.75850	-1.21187	1.30026
C	1.93197	-1.75072	2.34107
H	1.76477	-2.84214	2.22060
C	3.67510	-0.48583	-2.32629
H	4.22741	0.48019	-2.34656
C	3.51024	1.78452	0.49324
H	3.35219	2.80562	0.86534
H	4.23451	1.82783	-0.33916
H	3.94863	1.17199	1.29797
C	-3.56577	1.86160	-0.00979
H	-3.75392	2.12899	1.04661
H	-4.35148	1.17529	-0.35449
H	-3.62334	2.79220	-0.59345
H	-0.03646	4.95899	0.37059
H	2.38251	-1.58418	3.33735
H	-2.33012	-1.19597	3.36767
H	-2.50524	-1.11326	-2.86379
H	-3.24058	-2.66886	-2.41681
H	-4.28394	-1.30795	-2.93950
H	4.25049	-1.20731	-2.93616
H	2.70107	-0.32653	-2.81690
H	0.94464	-1.26562	2.31351
H	-2.68026	0.46001	2.78502

I1_Fe_L2

Fe	-0.00001	-0.36124	0.89023
H	0.00003	-1.45591	2.04795
P	-1.95337	-0.54180	0.32398
O	-2.25413	1.24401	-0.34669
C	-1.15836	1.89353	-0.53568
N	-1.18829	3.08698	-1.14581
C	0.00002	3.62302	-1.37868
N	1.18833	3.08701	-1.14575
C	1.15839	1.89350	-0.53572
N	0.00002	1.31474	-0.11955
O	2.25417	1.24405	-0.34658
P	1.95336	-0.54182	0.32400
N	3.44314	-0.52984	1.21787
C	4.59743	-0.62417	0.35867
H	5.47637	-0.99573	0.91656
H	4.86455	0.37055	-0.06089
C	4.18838	-1.57861	-0.75099
H	4.74251	-1.37387	-1.68768
H	4.40682	-2.63226	-0.46883
N	2.77359	-1.38746	-0.92582
C	2.11024	-2.15961	-1.92524
H	1.02598	-1.98988	-1.85028
C	3.54972	0.38421	2.31805
N	-3.44315	-0.52974	1.21785
C	-4.59744	-0.62410	0.35865
H	-4.86453	0.37060	-0.06099
H	-5.47640	-0.99560	0.91657
C	-4.18841	-1.57864	-0.75092
H	-4.40684	-2.63227	-0.46866
H	-4.74256	-1.37399	-1.68762
N	-2.77361	-1.38750	-0.92580
C	-2.11027	-2.15984	-1.92507
H	-2.29630	-3.24574	-1.79679
C	-3.54971	0.38437	2.31798
H	-3.80152	1.41361	1.98780
H	0.00003	4.62346	-1.83571
H	-2.43610	-1.88226	-2.94572
H	2.29625	-3.24554	-1.79714
H	2.58856	0.42153	2.85418
H	4.32748	0.05038	3.02736
H	3.80156	1.41345	1.98794
H	-4.32748	0.05061	3.02731
H	-2.58855	0.42171	2.85411
H	-1.02601	-1.99012	-1.85014
H	2.43608	-1.88185	-2.94584

I1_Fe_L3

Fe	0.03751	-0.44849	-0.94170
H	0.08291	-1.87661	-1.66752
P	-1.96255	-0.43993	-0.32865
C	-2.40485	1.37935	-0.69300
C	-1.17083	2.14479	-0.41280
N	-1.24239	3.40969	-0.02532
C	-0.09713	3.99668	0.30829
N	1.09345	3.39880	0.28808
C	1.11907	2.13536	-0.10367
N	0.00418	1.42524	-0.48653
C	2.40233	1.38879	-0.09954
P	1.98011	-0.45451	-0.17847
N	2.37366	-1.02675	1.44123
C	3.71384	-1.54386	1.57068
H	3.69480	-2.65659	1.59135
H	4.20193	-1.21392	2.50966
C	4.48796	-1.07846	0.34705
H	4.91198	-0.05770	0.50858
H	5.34394	-1.74634	0.13429
N	3.53268	-1.10375	-0.72607
C	4.01403	-0.73397	-2.01875
H	3.17451	-0.72325	-2.73240
C	1.36852	-1.70723	2.20917
N	-2.50206	-0.61873	1.34827
C	-3.93442	-0.75528	1.40045
H	-4.44716	0.23212	1.30770
H	-4.25605	-1.19301	2.36378
C	-4.29500	-1.64898	0.22953
H	-4.13012	-2.71505	0.50550
H	-5.36415	-1.54843	-0.04433
N	-3.42384	-1.26456	-0.85270
C	-3.34363	-2.19016	-1.94692
H	-3.16356	-3.23152	-1.60345
C	-1.94177	0.29212	2.30673
H	-2.41806	1.29721	2.27443
H	-0.13521	5.04422	0.63013
H	-4.27488	-2.18828	-2.54402
H	4.76447	-1.45938	-2.38231
H	0.37299	-1.45740	1.81028
H	1.48117	-2.81059	2.14927
H	1.40396	-1.42447	3.27870
H	-2.05751	-0.09957	3.33277
H	-0.86574	0.42199	2.10911
H	-2.50392	-1.91753	-2.60316
H	4.49679	0.27110	-2.03988
H	2.93913	1.59666	-1.04449
H	-2.61667	1.33622	-1.77492
H	3.04106	1.74333	0.72488
H	-3.27382	1.83969	-0.19494

I1_Fe_L4

Fe	0.00000	-0.72629	-0.69469
H	0.00001	-2.21647	-1.30272
P	1.97260	-0.61829	-0.10859
N	2.30201	1.15580	-0.33995
C	1.17328	1.92493	-0.38834
C	1.20446	3.32860	-0.32039
C	-0.00001	4.02328	-0.31081
C	-1.20447	3.32860	-0.32040
C	-1.17330	1.92492	-0.38836

N	-0.00001	1.23226	-0.47801
N	-2.30202	1.15579	-0.33998
P	-1.97260	-0.61830	-0.10861
N	-3.46540	-1.25769	-0.80271
C	-4.34134	-1.89259	0.14563
H	-4.11956	-2.98122	0.22818
H	-5.40379	-1.80591	-0.15969
C	-4.08678	-1.20883	1.47716
H	-4.71496	-0.29220	1.58093
H	-4.35628	-1.86706	2.32608
N	-2.68408	-0.90883	1.47858
C	-2.17757	-0.16313	2.58691
H	-2.29189	-0.72010	3.53487
C	-3.39132	-1.88375	-2.09121
H	-3.18245	-2.97236	-2.01890
N	3.46538	-1.25768	-0.80273
C	4.34133	-1.89261	0.14558
H	5.40377	-1.80594	-0.15977
H	4.11954	-2.98124	0.22811
C	4.08681	-1.20887	1.47712
H	4.35630	-1.86712	2.32603
H	4.71501	-0.29225	1.58090
N	2.68412	-0.90884	1.47857
C	2.17763	-0.16315	2.58692
H	2.68323	0.82096	2.71219
C	3.39128	-1.88368	-2.09125
H	4.33510	-1.75353	-2.65377
C	3.58700	1.77378	-0.20864
H	3.76875	2.50686	-1.01529
H	4.35834	0.99803	-0.29321
H	3.71621	2.30247	0.75806
C	-3.58701	1.77377	-0.20866
H	-3.71623	2.30246	0.75803
H	-4.35836	0.99802	-0.29324
H	-3.76876	2.50686	-1.01531
H	-4.33512	-1.75360	-2.65374
H	-2.57198	-1.43701	-2.67364
H	-1.10387	0.02785	2.42930
H	-2.68315	0.82099	2.71216
H	2.57196	-1.43690	-2.67367
H	3.18239	-2.97229	-2.01899
H	2.29197	-0.72014	3.53488
H	1.10394	0.02785	2.42934
H	-0.00001	5.11659	-0.26751
H	-2.15561	3.85776	-0.26186
H	2.15559	3.85777	-0.26184

I1_Fe_L5

Fe	0.08967	-1.44438	-0.03689
H	0.18104	-3.04905	-0.07821
P	-2.00492	-1.37064	-0.05349
N	-2.32124	0.36230	-0.16301
C	-1.20498	1.14898	-0.08375
N	-1.31467	2.46262	-0.07590
C	-0.16725	3.14144	-0.00126
N	1.04606	2.59734	0.07263
C	1.08368	1.27782	0.06698
N	-0.01676	0.45379	-0.01689
N	2.28106	0.61871	0.15019
P	2.16194	-1.13670	0.03116
N	3.30669	-1.65599	1.24643
C	4.40938	-2.40661	0.69709
H	4.15952	-3.48944	0.62620
H	5.30694	-2.31954	1.33862
C	4.64800	-1.84085	-0.69053
H	5.30506	-0.94167	-0.64664
H	5.15747	-2.57428	-1.34340
N	3.33542	-1.53584	-1.18890
C	3.22075	-0.96464	-2.49528
H	2.15871	-0.78241	-2.72256
C	2.77487	-2.15739	2.48540
N	-3.07750	-1.91047	1.20284
C	-4.36582	-2.35660	0.74953
H	-5.12046	-1.53638	0.74164
H	-4.76451	-3.14819	1.41139
C	-4.11780	-2.87985	-0.65304
H	-3.74294	-3.92728	-0.60473
H	-5.04328	-2.89128	-1.25969
N	-3.13045	-2.00251	-1.23318
C	-2.59173	-2.42834	-2.49724
H	-2.15691	-3.44836	-2.44695
C	-2.97419	-1.35064	2.51518
H	-3.62030	-0.45700	2.65368
C	-3.62336	0.96863	-0.24280
H	-3.98530	1.31362	0.74221
H	-4.32952	0.23412	-0.65282
H	-3.59690	1.84516	-0.90580
C	3.51164	1.35685	0.24693
H	3.88912	1.67261	-0.74258
H	4.26642	0.72401	0.73428
H	3.36435	2.26463	0.84834
H	-3.37680	-2.42326	-3.27413
H	3.62193	-1.64878	-3.26438
H	1.88498	-1.57712	2.77124
H	2.46790	-3.22148	2.41083
H	3.52326	-2.06796	3.29287
H	-3.25669	-2.09034	3.28568
H	-1.93207	-1.04637	2.70070
H	-1.78991	-1.74369	-2.81113
H	3.76029	0.00268	-2.59032
C	-0.29514	4.63163	0.01187
F	-0.99979	5.07865	1.07533
F	0.88313	5.25524	0.04566
F	-0.95297	5.10152	-1.06923

I1_Fe_L6

Fe	-0.00001	-1.22918	0.00226
H	-0.00032	-2.84860	0.00148
P	2.08842	-1.03178	-0.00017
N	2.31645	0.72160	0.00696
C	1.14375	1.43449	0.00382
N	1.18239	2.75849	0.00025
C	-0.00003	3.37732	-0.00130
N	-1.18243	2.75848	0.00031
C	-1.14374	1.43448	0.00371
N	0.00000	0.67210	0.00423
N	-2.31645	0.72159	0.00645
P	-2.08844	-1.03174	-0.00045
C	3.56113	1.43899	-0.00013
H	3.66685	2.06480	-0.90322
H	4.39742	0.72731	0.03037
H	3.64369	2.11443	0.86813
C	-3.56112	1.43901	0.00034
H	-3.64506	2.11134	0.87094
H	-4.39744	0.72721	0.02705
H	-3.66544	2.06806	-0.90060
H	-0.00004	4.47361	-0.00422
C	-3.19810	-1.52770	-1.40301
H	-2.76574	-1.15357	-2.34137
H	-3.20973	-2.62801	-1.44362
H	-4.23478	-1.16485	-1.29869
C	3.19769	-1.52747	-1.40319
H	3.20958	-2.62777	-1.44384
H	2.76484	-1.15342	-2.34136
H	4.23430	-1.16430	-1.29926
C	3.20470	-1.54060	1.39225

H	2.78013	-1.16986	2.33550
H	3.20995	-2.64119	1.42594
H	4.24301	-1.18369	1.28406
C	-3.20418	-1.54048	1.39244
H	-4.24254	-1.18358	1.28463
H	-3.20936	-2.64107	1.42625
H	-2.77922	-1.16963	2.33547

I1_Fe_L7

Fe	0.00701	-0.83751	-0.77759
H	0.01349	-2.33663	-1.29348
P	-1.93976	-0.75275	-0.16481
N	-2.27528	1.01285	-0.07842
C	-1.15327	1.76764	-0.11141
N	-1.20430	3.08571	0.11882
C	-0.02410	3.68622	0.17856
N	1.16242	3.09914	0.14166
C	1.13488	1.77823	-0.07960
N	-0.00257	1.08705	-0.35816
N	2.25788	1.02804	-0.00375
P	1.94850	-0.73471	-0.15549
O	2.69558	-1.21961	1.31088
C	3.83662	-2.00425	1.13015
H	3.56460	-3.07735	1.08258
H	4.52178	-1.86313	1.98359
C	4.45955	-1.54926	-0.18540
H	5.16919	-0.71405	-0.01629
H	5.01404	-2.36429	-0.68110
O	3.39412	-1.14414	-0.99321
O	-2.64297	-1.27782	1.30509
C	-4.00309	-1.59660	1.23343
H	-4.62550	-0.73870	1.55859
H	-4.22042	-2.44378	1.90531
C	-4.27191	-1.94086	-0.22689
H	-4.07277	-3.01438	-0.41655
H	-5.31721	-1.73229	-0.51515
O	-3.40733	-1.14204	-0.97642
C	-3.54478	1.60665	0.24691
H	-3.60395	2.62140	-0.16853
H	-3.69836	1.68104	1.33851
H	-4.34653	0.99288	-0.18651
C	3.53583	1.60389	0.31620
H	4.25697	1.42932	-0.49908
H	3.93753	1.15370	1.23882
H	3.42458	2.68454	0.46962
H	-0.03139	4.77826	0.29794

I1_Fe_L8

Fe	0.00001	1.13287	-0.00000
H	-0.00001	2.81826	0.00019
N	2.07870	0.82339	-0.02547
C	2.28284	-0.51908	0.56519
C	1.14665	-1.41627	0.19433
N	1.18071	-2.71559	0.18599
C	-0.00003	-3.36178	0.00001
N	-1.18076	-2.71557	-0.18596
C	-1.14667	-1.41626	-0.19436
N	0.00000	-0.65854	-0.00005
C	-2.28286	-0.51905	-0.56520
N	-2.07869	0.82342	0.02547
H	-0.00004	-4.45436	0.00003
H	-2.26409	-0.37693	-1.66365
H	3.27021	-0.94594	0.29432
H	-3.27022	-0.94590	-0.29432
H	2.26407	-0.37698	1.66364
C	-2.82778	1.83142	-0.70081
H	-2.66311	2.80917	-0.22883
H	-2.45230	1.89141	-1.73202
H	-3.91669	1.59788	-0.71401
C	2.46617	0.81993	-1.42860
H	1.94463	0.00789	-1.95264
H	2.17421	1.77420	-1.88903
H	3.56454	0.67252	-1.53604
C	-2.46614	0.81995	1.42860
H	-1.94461	0.00789	1.95263
H	-2.17415	1.77421	1.88904
H	-3.56451	0.67256	1.53605
C	2.82781	1.83137	0.70083
H	3.91671	1.59780	0.71403
H	2.66317	2.80913	0.22886
H	2.45232	1.89136	1.73204

I1_Fe_L9

Fe	-0.00001	-1.28556	-0.00105
H	-0.00033	-2.95115	-0.00478
P	2.06828	-1.01554	-0.00002
N	2.39155	0.71735	0.00163
C	1.19724	1.45905	0.00061
C	1.21028	2.86288	0.00013
C	0.00001	3.57044	-0.00001
C	-1.21027	2.86289	-0.00059
C	-1.19723	1.45906	-0.00036
C	0.00000	0.67409	-0.00009
N	-2.39155	0.71736	-0.00054
P	-2.06829	-1.01550	0.00018
C	3.65635	1.36090	0.00066
H	3.80731	2.00940	-0.89180
H	4.46622	0.61582	0.00262
H	3.80652	2.01294	0.89060
C	-3.65635	1.36092	-0.00017
H	-3.80682	2.01096	0.89123
H	-4.46623	0.61584	-0.00030
H	-3.80701	2.01142	-0.89118
H	0.00001	4.66740	-0.00012
C	-3.20892	-1.55951	-1.39054
H	-2.78695	-1.19110	-2.33711
H	-3.20167	-2.66146	-1.41317
H	-4.25331	-1.20744	-1.28480
C	3.20850	-1.55763	-1.39188
H	3.20097	-2.65954	-1.41636
H	2.78646	-1.18755	-2.33778
H	4.25300	-1.20599	-1.28575
C	3.20741	-1.56007	1.39166
H	2.78410	-1.19262	2.33799
H	3.20098	-2.66204	1.41347
H	4.25167	-1.20717	1.28735
C	-3.20694	-1.55823	1.39299
H	-4.25134	-1.20588	1.28828
H	-3.20008	-2.66016	1.41666
H	-2.78360	-1.18905	2.33865
H	2.15605	3.41792	0.00019
H	-2.15604	3.41793	-0.00099

I1_Fe_L10

Fe	0.00002	-1.34260	-0.00002
H	0.00003	-3.09478	-0.00015
O	2.10458	-0.92420	0.30127
C	2.42658	0.39902	-0.11782
C	1.20538	1.25959	-0.00084
C	1.21626	2.64756	0.01674

C	-0.00004	3.36804	-0.00007
C	-1.21632	2.64754	-0.01672
C	-1.20541	1.25957	0.00089
C	-0.00001	0.50096	0.00005
C	-2.42658	0.39897	0.11791
O	-2.10460	-0.92421	-0.30133
H	-0.00005	4.46471	-0.00015
H	-2.75649	0.33700	1.18177
H	3.29426	0.76917	0.47680
H	-3.29433	0.76916	-0.47658
H	2.75662	0.33716	-1.18165
C	-3.06607	-1.86713	0.04146
H	-2.72264	-2.83511	-0.34852
H	-3.16388	-1.94765	1.14476
H	-4.05891	-1.60094	-0.38904
C	3.06613	-1.86706	-0.04145
H	4.05889	-1.60090	0.38925
H	2.72264	-2.83508	0.34839
H	3.16414	-1.94748	-1.14473
H	2.17015	3.19869	0.03264
H	-2.17022	3.19864	-0.03266

I1_Fe_L11

Fe	-0.00005	-1.17871	0.00001
H	0.00005	-2.94350	0.00071
N	-2.11687	-0.83913	-0.03450
C	-2.33852	0.50502	0.55045
C	-1.19303	1.41312	0.20600
C	-1.20498	2.79999	0.19113
C	0.00019	3.51955	0.00008
C	1.20528	2.79988	-0.19105
C	1.19314	1.41301	-0.20621
C	0.00000	0.65941	-0.00022
C	2.33856	0.50479	-0.55057
N	2.11679	-0.83928	0.03451
H	0.00026	4.61641	0.00020
H	2.36074	0.35021	-1.64714
H	-3.34406	0.89397	0.25050
H	3.34413	0.89369	-0.25065
H	-2.36072	0.35055	1.64703
C	2.86212	-1.84841	-0.68336
H	2.69487	-2.82445	-0.20575
H	2.47768	-1.91277	-1.71429
H	3.95897	-1.61644	-0.70100
C	-2.47229	-0.84803	-1.44051
H	-1.95311	-0.02437	-1.94966
H	-2.13781	-1.79337	-1.89415
H	-3.58098	-0.73161	-1.57248
C	2.47217	-0.84810	1.44052
H	1.95305	-0.02434	1.94959
H	2.13760	-1.79338	1.89423
H	3.58086	-0.73175	1.57252
C	-2.86230	-1.84812	0.68347
H	-3.95913	-1.61604	0.70109
H	-2.69515	-2.82422	0.20595
H	-2.47786	-1.91243	1.71440
H	-2.14473	3.35338	0.34739
H	2.14511	3.35317	-0.34712

I1_Ni_L1

Ni	-0.00000	-0.65290	-0.00002
H	-0.00000	-2.12389	-0.00005
P	2.15475	-0.53196	0.03475
N	2.29830	1.23259	0.15088
C	1.15299	1.95359	0.07238
N	1.18582	3.28443	0.06893
C	0.00001	3.87486	-0.00006
N	-1.18582	3.28443	-0.06890
C	-1.15299	1.95360	-0.07235
N	0.00000	1.25337	0.00000
N	-2.29830	1.23259	-0.15086
P	-2.15476	-0.53196	-0.03476
N	-3.17230	-1.12558	-1.22947
C	-4.23619	-1.96158	-0.68205
H	-3.93398	-3.02612	-0.69009
H	-5.14336	-1.86659	-1.29914
C	-4.46232	-1.47327	0.73866
H	-5.19359	-0.64206	0.77119
H	-4.84580	-2.27429	1.38880
N	-3.15244	-1.04043	1.20842
C	-3.01681	-0.54200	2.55516
H	-1.96410	-0.29960	2.76299
C	-2.67570	-1.44260	-2.55030
N	3.15246	-1.04041	-1.20842
C	4.46234	-1.47325	-0.73865
H	5.19361	-0.64204	-0.77116
H	4.84583	-2.27426	-1.38879
C	4.23618	-1.96158	0.68206
H	3.93399	-3.02612	0.69008
H	5.14335	-1.86659	1.29917
N	3.17228	-1.12560	1.22947
C	2.67565	-1.44264	2.55028
H	2.25905	-2.46485	2.60219
C	3.01684	-0.54199	-2.55517
H	3.62247	0.36706	-2.72726
C	3.58672	1.90143	0.21488
H	3.51464	2.79562	0.84491
H	3.92974	2.21034	-0.78410
H	4.31614	1.21279	0.65818
C	-3.58672	1.90145	-0.21483
H	-3.92970	2.21039	0.78415
H	-4.31617	1.21279	-0.65807
H	-3.51466	2.79561	-0.84490
H	-0.00001	4.97111	0.00013
H	3.48830	-1.35891	3.28627
H	-3.33364	-1.30798	3.27789
H	-1.88938	-0.73103	-2.84102
H	-2.25908	-2.46481	-2.60223
H	-3.48837	-1.35887	-3.28627
H	3.33369	-1.30796	-3.27789
H	1.96414	-0.29958	-2.76301
H	1.88932	-0.73108	2.84099
H	-3.62243	0.36705	2.72726

I1_Ni_L2

Ni	-0.00000	-0.58428	0.24272
H	-0.00000	-1.98267	0.68239
P	-2.15225	-0.46959	0.16164
O	-2.26713	1.22970	-0.36302
C	-1.15539	1.89025	-0.55758
N	-1.18961	3.14972	-0.94628
C	0.00000	3.71770	-1.11874
N	1.18961	3.14973	-0.94626
C	1.15539	1.89025	-0.55756
N	0.00000	1.22350	-0.34323
O	2.26713	1.22971	-0.36299
P	2.15225	-0.46959	0.16164
N	3.24328	-0.50502	1.41131
C	4.59805	-0.66806	0.87361
H	5.24341	-1.12636	1.63664
H	5.02409	0.32007	0.61409
C	4.45603	-1.54673	-0.36038
H	5.22372	-1.31384	-1.11424

H	4.54025	-2.61904	-0.10914
N	3.12056	-1.27972	-0.89381
C	2.68233	-1.95300	-2.09380
H	1.60676	-1.78829	-2.25093
C	3.08307	0.35079	2.56886
N	-3.24327	-0.50499	1.41131
C	-4.59804	-0.66804	0.87363
H	-5.02408	0.32009	0.61410
H	-5.24340	-1.12632	1.63668
C	-4.45604	-1.54673	-0.36034
H	-4.54026	-2.61903	-0.10909
H	-5.22373	-1.31384	-1.11420
N	-3.12058	-1.27973	-0.89379
C	-2.68236	-1.95304	-2.09378
H	-2.85319	-3.03889	-2.01345
C	-3.08303	0.35084	2.56885
H	-3.43756	1.37820	2.37119
H	0.00000	4.76563	-1.43840
H	-3.21925	-1.57835	-2.97839
H	2.85317	-3.03885	-2.01350
H	2.02474	0.39180	2.86369
H	3.65142	-0.06063	3.41416
H	3.43759	1.37816	2.37121
H	-3.65138	-0.06058	3.41416
H	-2.02471	0.39184	2.86366
H	-1.60680	-1.78832	-2.25093
H	3.21920	-1.57828	-2.97842

I1_Ni_L3

Ni	-0.00647	-0.53058	-0.09074
H	-0.02576	-1.99024	-0.14881
P	-2.16575	-0.41155	0.04514
C	-2.27534	1.32381	0.76907
C	-1.07671	2.11353	0.37586
N	-1.09379	3.43545	0.42301
C	0.04895	4.04251	0.12116
N	1.18000	3.43876	-0.22865
C	1.13222	2.11904	-0.30089
N	0.01768	1.41637	0.00001
C	2.31999	1.33668	-0.73905
P	2.15892	-0.43397	-0.12244
N	3.04667	-0.50809	1.31719
C	4.24643	-1.32920	1.15491
H	4.05436	-2.35834	1.51439
H	5.07663	-0.91300	1.74690
C	4.55602	-1.33599	-0.33022
H	5.13310	-0.43461	-0.62341
H	5.14523	-2.21771	-0.62342
N	3.25460	-1.37225	-0.98883
C	3.21508	-1.44127	-2.42901
H	2.17195	-1.49607	-2.77421
C	2.38421	-0.57762	2.60204
N	-3.15989	-1.41067	0.96350
C	-4.52124	-1.36766	0.44228
H	-5.08083	-0.49540	0.83901
H	-5.06353	-2.27632	0.74367
C	-4.36045	-1.27560	-1.06374
H	-4.18864	-2.27936	-1.49759
H	-5.25118	-0.84516	-1.54779
N	-3.19804	-0.41918	-1.29439
C	-2.68578	-0.35816	-2.64560
H	-2.37001	-1.35053	-3.01850
C	-2.96239	-1.57650	2.38183
H	-3.40705	-0.75396	2.97567
H	0.06032	5.13710	0.16644
H	-3.46003	0.03427	-3.32108
H	3.72797	-2.35039	-2.77393
H	1.51278	0.09277	2.62413
H	2.04339	-1.60271	2.84161
H	3.07285	-0.24867	3.39396
H	-3.42160	-2.51778	2.71635
H	-1.88624	-1.63247	2.60668
H	-1.82457	0.32340	-2.70054
H	3.70477	-0.57279	-2.91161
H	2.29784	1.27833	-1.84403
H	-3.20312	1.86298	0.52982
H	3.25450	1.84239	-0.45679
H	-2.24614	1.19926	1.86857

I1_Ni_L4

Ni	0.00000	-0.56903	0.16306
H	0.00001	-2.03258	0.33128
P	2.13986	-0.49996	0.09345
N	2.32057	1.22729	-0.12525
C	1.17972	1.99443	-0.19816
C	1.21155	3.38190	-0.36839
C	-0.00000	4.05514	-0.44832
C	-1.21155	3.38189	-0.36842
C	-1.17972	1.99442	-0.19819
N	-0.00000	1.33691	-0.10418
N	-2.32057	1.22728	-0.12530
P	-2.13986	-0.49997	0.09345
N	-3.05780	-1.22257	-1.12623
C	-4.01877	-2.17121	-0.57626
H	-3.56571	-3.17827	-0.48695
H	-4.89015	-2.25465	-1.24429
C	-4.39836	-1.63188	0.79201
H	-5.24049	-0.91469	0.72601
H	-4.70963	-2.43805	1.47419
N	-3.19448	-0.98998	1.29775
C	-3.19651	-0.41445	2.61849
H	-3.44842	-1.17775	3.36976
C	-2.42704	-1.57135	-2.38039
H	-1.85753	-2.51758	-2.31201
N	3.05779	-1.22254	-1.12625
C	4.01877	-2.17118	-0.57632
H	4.89015	-2.25458	-1.24436
H	3.56573	-3.17825	-0.48705
C	4.39837	-1.63190	0.79197
H	4.70964	-2.43809	1.47411
H	5.24050	-0.91470	0.72598
N	3.19449	-0.99001	1.29773
C	3.19652	-0.41453	2.61849
H	3.92383	0.41358	2.71154
C	2.42701	-1.57129	-2.38042
H	3.18981	-1.67884	-3.16487
C	3.62497	1.84481	-0.24425
H	3.74434	2.35338	-1.21340
H	4.39507	1.06895	-0.18206
H	3.80053	2.57164	0.56444
C	-3.62497	1.84479	-0.24434
H	-3.80055	2.57162	0.56435
H	-4.39507	1.06892	-0.18217
H	-3.74432	2.35336	-1.21348
H	-3.18984	-1.67895	-3.16482
H	-1.73864	-0.77198	-2.69281
H	-2.19814	-0.02235	2.86055
H	-3.92384	0.41364	2.71151
H	1.73860	-0.77192	-2.69280
H	1.85753	-2.51753	-2.31207
H	3.44846	-1.17786	3.36973
H	2.19814	-0.02247	2.86058
H	-0.00001	5.13916	-0.58100
H	-2.15560	3.91819	-0.44093
H	2.15559	3.91820	-0.44088

I1_Ni_L5

Ni	0.09383	-1.36461	-0.00726
H	0.18509	-2.83174	-0.00812
P	-2.06535	-1.38320	-0.03636
N	-2.31854	0.37511	-0.14769
C	-1.22222	1.16214	-0.07233
N	-1.33390	2.48894	-0.06638
C	-0.18588	3.14200	0.00068
N	1.03145	2.63941	0.06526
C	1.08373	1.30490	0.06615
N	-0.02482	0.53812	-0.00473
N	2.27018	0.66292	0.14066
P	2.23820	-1.11517	0.03075
N	3.28373	-1.63403	1.23283
C	4.40939	-2.39381	0.69628
H	4.18595	-3.47727	0.71395
H	5.30415	-2.22654	1.31599
C	4.60590	-1.90284	-0.72830
H	5.28177	-1.02662	-0.76767
H	5.04095	-2.68262	-1.37159
N	3.27153	-1.55893	-1.20479
C	3.10495	-1.08979	-2.55901
H	2.03954	-0.91410	-2.76883
C	2.80769	-1.97103	2.55660
N	-3.02369	-1.95005	1.20963
C	-4.30911	-2.45807	0.74563
H	-5.08779	-1.67194	0.78786
H	-4.63929	-3.28324	1.39464
C	-4.06446	-2.92612	-0.67908
H	-3.70575	-3.97265	-0.69532
H	-4.97842	-2.87594	-1.29124
N	-3.04891	-2.03191	-1.22801
C	-2.54501	-2.31237	-2.55465
H	-2.07380	-3.30985	-2.61629
C	-2.90585	-1.45725	2.56053
H	-3.56462	-0.58922	2.74717
C	-3.64850	0.96039	-0.20158
H	-4.01628	1.21232	0.80438
H	-4.32779	0.23946	-0.67234
H	-3.63196	1.87684	-0.80252
C	3.51724	1.40807	0.20193
H	3.85974	1.69968	-0.80220
H	4.27625	0.77766	0.68086
H	3.38366	2.31760	0.79859
H	-3.36643	-2.26841	-3.28420
H	3.46665	-1.84617	-3.27058
H	1.96703	-1.31928	2.83521
H	2.47223	-3.02177	2.62077
H	3.60809	-1.81421	3.29398
H	-3.16810	-2.24837	3.27790
H	-1.86811	-1.15270	2.76164
H	-1.80136	-1.55670	-2.84614
H	3.65504	-0.14912	-2.74525
C	-0.32573	4.66707	0.00129
F	-1.05860	5.04269	1.04322
F	0.84542	5.26861	0.06766
F	-0.94223	5.05456	-1.10927

I1_Ni_L6

Ni	-0.00002	-1.16367	-0.00043
H	-0.00009	-2.63571	0.00334
P	-2.15280	-1.01713	0.00073
N	-2.30736	0.73145	-0.00685
C	-1.15213	1.44424	-0.00401
N	-1.18684	2.77328	-0.00081
C	0.00014	3.36612	0.00048
N	1.18706	2.77314	-0.00026
C	1.15218	1.44411	-0.00336
N	-0.00002	0.73949	-0.00450
N	2.30737	0.73125	-0.00545
P	2.15279	-1.01731	0.00061
C	-3.57791	1.44497	-0.00066
H	-3.68234	2.04826	0.91141
H	-4.40211	0.72401	-0.04545
H	-3.64772	2.11692	-0.86612
C	3.57779	1.44511	0.00025
H	3.64831	2.11505	-0.86673
H	4.40219	0.72424	-0.04188
H	3.68102	2.05060	0.91097
H	0.00021	4.46207	0.00292
C	3.12423	-1.55133	1.43837
H	2.67334	-1.16164	2.36038
H	3.10194	-2.65037	1.47593
H	4.17143	-1.22304	1.36885
C	-3.12350	-1.54961	1.43959
H	-3.10086	-2.64861	1.47852
H	-2.67227	-1.15867	2.36091
H	-4.17081	-1.22169	1.37017
C	-3.12604	-1.56528	-1.43026
H	-2.68059	-1.17861	-2.35615
H	-3.09560	-2.66441	-1.46064
H	-4.17563	-1.24509	-1.36016
C	3.12523	-1.56374	-1.43163
H	4.17506	-1.24434	-1.36120
H	3.09406	-2.66276	-1.46439
H	2.67981	-1.17471	-2.35656

I1_Ni_L7

Ni	0.00000	-0.84462	-0.00001
H	0.00001	-2.30756	-0.00002
P	-2.13460	-0.68249	-0.00891
N	-2.30677	1.04174	-0.04144
C	-1.15290	1.77052	-0.01787
N	-1.18589	3.09569	-0.01496
C	-0.00000	3.69027	0.00005
N	1.18589	3.09569	0.01497
C	1.15290	1.77052	0.01786
N	-0.00000	1.06851	-0.00001
N	2.30677	1.04175	0.04142
P	2.13460	-0.68249	0.00890
O	3.12758	-1.16368	1.20119
C	4.17124	-2.01766	0.71409
H	3.86269	-3.06606	0.84147
H	5.06945	-1.83224	1.31594
C	4.35792	-1.64833	-0.75251
H	5.08291	-0.83076	-0.88631
H	4.66444	-2.50105	-1.36964
O	3.06922	-1.21040	-1.20796
O	-3.06919	-1.21041	1.20796
C	-4.35790	-1.64833	0.75254
H	-5.08289	-0.83074	0.88636
H	-4.66442	-2.50104	1.36968
C	-4.17126	-2.01765	-0.71406
H	-3.86273	-3.06606	-0.84145
H	-5.06948	-1.83221	-1.31590
O	-3.12760	-1.16369	-1.20118
C	-3.61590	1.68249	-0.03445
H	-3.49492	2.74456	-0.27010
H	-4.08638	1.58880	0.95465
H	-4.25422	1.21748	-0.79706
C	3.61590	1.68250	0.03444
H	4.08639	1.58879	-0.95466
H	4.25421	1.21750	0.79706
H	3.49491	2.74457	0.27007
H	-0.00000	4.78590	-0.00004

I1_Ni_L8

Ni	0.00001	1.06128	0.00000
H	0.00002	2.54201	0.00005
N	1.96348	0.83966	-0.02028
C	2.29533	-0.55031	0.41356
C	1.13687	-1.46745	0.16832
N	1.18100	-2.78524	0.16735
C	-0.00003	-3.38767	0.00000
N	-1.18105	-2.78522	-0.16733
C	-1.13690	-1.46743	-0.16834
N	-0.00000	-0.80202	-0.00003
C	-2.29534	-0.55027	-0.41358
N	-1.96346	0.83970	0.02029
H	-0.00005	-4.48261	0.00004
H	-2.48645	-0.55274	-1.50004
H	3.21885	-0.91777	-0.06539
H	-3.21887	-0.91771	0.06537
H	2.48644	-0.55281	1.50002
C	-2.69534	1.80887	-0.81334
H	-2.51020	2.81937	-0.43335
H	-2.33765	1.75296	-1.84898
H	-3.77887	1.59960	-0.78433
C	2.34898	1.01322	-1.43505
H	1.84873	0.26059	-2.05947
H	2.04839	2.01114	-1.77482
H	3.44151	0.90219	-1.54797
C	-2.34896	1.01324	1.43506
H	-1.84872	0.26059	2.05947
H	-2.04835	2.01115	1.77484
H	-3.44149	0.90221	1.54797
C	2.69537	1.80881	0.81337
H	3.77889	1.59952	0.78434
H	2.51025	2.81932	0.43338
H	2.33768	1.75290	1.84900

I1_Ni_L9

Ni	-0.00000	1.17602	0.00010
H	-0.00001	2.70537	0.00042
P	-2.11522	0.98290	-0.00024
N	-2.38990	-0.71716	0.00167
C	-1.20547	-1.47259	0.00078
C	-1.21692	-2.87590	0.00072
C	-0.00000	-3.55349	0.00010
C	1.21691	-2.87590	-0.00066
C	1.20547	-1.47260	-0.00091
C	0.00000	-0.74945	-0.00008
N	2.38990	-0.71716	-0.00195
P	2.11523	0.98289	0.00031
C	-3.67825	-1.35207	0.00117
H	-3.82332	-1.98589	-0.89203
H	-4.47495	-0.59652	0.00242
H	-3.82273	-1.98800	0.89292
C	3.67826	-1.35207	-0.00140
H	3.82383	-1.98503	0.89235
H	4.47495	-0.59652	-0.00389
H	3.82223	-1.98887	-0.89260
H	-0.00000	-4.64703	0.00020
C	3.07582	1.61642	-1.42040
H	2.64059	1.22534	-2.34983
H	2.99795	2.71352	-1.43153
H	4.13831	1.33417	-1.36496
C	-3.07390	1.61271	-1.42398
H	-2.99583	2.70976	-1.43827
H	-2.63759	1.21880	-2.35171
H	-4.13650	1.33075	-1.36899
C	-3.07606	1.61619	1.42040
H	-2.64084	1.22517	2.34986
H	-2.99840	2.71331	1.43157
H	-4.13848	1.33373	1.36484
C	3.07413	1.61250	1.42397
H	4.13667	1.33033	1.36888
H	2.99626	2.70956	1.43827
H	2.63784	1.21868	2.35175
H	-2.15247	-3.43917	0.00125
H	2.15246	-3.43917	-0.00101

I1_Ni_L10

Ni	0.00017	-1.24922	0.00023
H	0.00036	-2.81258	0.00013
C	2.40414	0.39252	0.00209
C	1.21095	1.29597	0.00187
C	1.21923	2.69143	0.00200
C	-0.00028	3.37571	-0.00044
C	-1.21968	2.69124	-0.00257
C	-1.21122	1.29579	-0.00167
C	-0.00008	0.61450	0.00025
C	-2.40424	0.39212	-0.00145
H	-0.00037	4.46875	-0.00075
H	-3.03965	0.52500	0.89609
H	3.05317	0.53975	0.88740
H	-3.05312	0.53854	-0.88700
H	3.03935	0.52488	-0.89568
H	2.15798	3.25477	0.00367
H	-2.15854	3.25442	-0.00455
O	1.92063	-0.95991	0.01739
C	2.93489	-1.93975	-0.01057
H	3.52961	-1.84614	-0.93536
H	3.60018	-1.82153	0.86181
H	2.44287	-2.91642	0.02099
O	-1.92046	-0.96026	-0.01544
C	-2.93469	-1.94023	0.00847
H	-3.53694	-1.84252	0.92795
H	-3.59287	-1.82625	-0.86984
H	-2.44218	-2.91689	-0.01475

I1_Ni_L11

Ni	-0.00008	-1.08643	0.00003
H	-0.00016	-2.65780	0.00027
N	-1.96513	-0.85209	-0.03246
C	-2.32706	0.50902	0.47951
C	-1.19520	1.45427	0.20396
C	-1.20589	2.84952	0.19626
C	0.00026	3.53289	0.00005
C	1.20631	2.84935	-0.19622
C	1.19539	1.45411	-0.20411
C	0.00003	0.77497	-0.00014
C	2.32711	0.50868	-0.47963
N	1.96501	-0.85234	0.03247
H	0.00035	4.62629	0.00012
H	2.47633	0.41273	-1.56862
H	-3.30170	0.81916	0.05367
H	3.30181	0.81872	-0.05384
H	-2.47634	0.41318	1.56851
C	2.69714	-1.87604	-0.71350
H	2.49479	-2.85876	-0.27272
H	2.35330	-1.89031	-1.75574
H	3.78507	-1.67068	-0.68737
C	-2.31025	-0.94531	-1.45624
H	-1.83905	-0.12087	-2.00597
H	-1.94451	-1.89850	-1.85850
H	-3.40787	-0.88736	-1.58822
C	2.31016	-0.94549	1.45624

H	1.83907	-0.12095	2.00592
H	1.94432	-1.89860	1.85859
H	3.40779	-0.88764	1.58818
C	-2.69744	-1.87563	0.71357
H	-3.78534	-1.67011	0.68738
H	-2.49521	-2.85841	0.27287
H	-2.35363	-1.88986	1.75581
H	-2.13119	3.41455	0.34893
H	2.13171	3.41424	-0.34878

4.4.2. I2

I2_Co_L1

Co	0.03426	-0.38131	0.01202
H	-1.68375	-2.79733	-0.09246
P	2.17695	-0.23377	0.02850
N	2.37167	1.51898	0.17317
C	1.22485	2.23649	0.09375
N	1.28346	3.57115	0.10147
C	0.11367	4.18487	0.02091
N	-1.07643	3.61442	-0.06595
C	-1.06551	2.27750	-0.07414
N	0.06625	1.53782	0.00595
N	-2.22818	1.59338	-0.16578
P	-2.10343	-0.18010	-0.04576
N	-3.19612	-0.71281	-1.23891
C	-4.33730	-1.42858	-0.69591
H	-4.14608	-2.52080	-0.70009
H	-5.23450	-1.24995	-1.31291
C	-4.52016	-0.92334	0.72574
H	-5.18750	-0.03663	0.75841
H	-4.97476	-1.69234	1.37208
N	-3.18645	-0.60552	1.19054
C	-3.01564	-0.12337	2.53274
H	-1.94630	0.03541	2.73746
C	-2.71693	-1.13907	-2.53158
N	3.20999	-0.72790	-1.21304
C	4.47176	-1.26963	-0.75723
H	5.27053	-0.49845	-0.74543
H	4.80576	-2.07609	-1.43066
C	4.18537	-1.79184	0.63868
H	3.76322	-2.81559	0.58781
H	5.09435	-1.82926	1.26178
N	3.21641	-0.87229	1.21583
C	2.65780	-1.25897	2.49321
H	2.18084	-2.25507	2.45065
C	3.08326	-0.24383	-2.55755
H	3.74900	0.61793	-2.76231
C	3.65164	2.18781	0.25324
H	3.93808	2.63432	-0.71192
H	4.40662	1.45235	0.55716
H	3.62104	2.99212	0.99963
C	-3.49019	2.29356	-0.25685
H	-3.86240	2.59712	0.73501
H	-4.22297	1.62990	-0.73335
H	-3.38005	3.19778	-0.86770
C	-0.62513	-3.17793	0.01119
O	0.26473	-2.24976	0.07870
O	-0.42745	-4.37325	0.05188
H	0.13341	5.28181	0.02720
H	3.44985	-1.27480	3.25779
H	-3.39189	-0.86130	3.25886
H	-1.79733	-0.59553	-2.79081
H	-2.49241	-2.22240	-2.55365
H	-3.46858	-0.92692	-3.30858
H	3.32360	-1.03895	-3.28141
H	2.04583	0.07381	-2.74109
H	1.89821	-0.52906	2.80741
H	-3.54723	0.83280	2.71074

I2_Co_L2

Co	0.02010	-0.33998	0.10687
H	-1.80339	-2.67576	0.00646
P	2.16720	-0.21413	0.03394
O	2.36207	1.55021	0.21565
C	1.25883	2.24883	0.18436
N	1.33745	3.57052	0.21983
C	0.17521	4.20546	0.16983
N	-1.02627	3.65314	0.07864
C	-1.03305	2.32925	0.04711
N	0.08736	1.57230	0.10984
O	-2.17451	1.70359	-0.04965
P	-2.09669	-0.07818	-0.03535
N	-3.04474	-0.47957	-1.35630
C	-4.39953	-0.86869	-1.00000
H	-4.48086	-1.97398	-1.00027
H	-5.12716	-0.47718	-1.72961
C	-4.63606	-0.30972	0.39102
H	-4.99192	0.73993	0.34243
H	-5.38318	-0.89440	0.94985
N	-3.34335	-0.38281	1.05648
C	-3.26470	0.12593	2.40389
H	-2.23321	0.04023	2.77536
C	-2.49208	-0.99719	-2.58406
N	3.19252	-0.47780	-1.27335
C	4.57641	-0.55499	-0.82699
H	5.01582	0.46049	-0.74579
H	5.17702	-1.12546	-1.55238
C	4.52385	-1.23498	0.53058
H	4.54584	-2.33640	0.42500
H	5.37337	-0.93987	1.16832
N	3.26146	-0.82921	1.12842
C	2.86921	-1.43478	2.37870
H	2.92187	-2.53534	2.31823
C	2.93785	0.17307	-2.53458
H	3.30429	1.21755	-2.54515
C	-0.76153	-3.09780	0.13362
O	0.17013	-2.20608	0.18935
O	-0.61334	-4.29653	0.20595
H	0.21147	5.30063	0.20433
H	3.51734	-1.09099	3.20039
H	-3.91391	-0.46427	3.06767
H	-1.41601	-0.77764	-2.63312
H	-2.61939	-2.09313	-2.64845
H	-2.97914	-0.53807	-3.45881
H	3.43228	-0.37693	-3.34911
H	1.85672	0.17994	-2.73791
H	1.83208	-1.16420	2.61713
H	-3.57258	1.18713	2.46488

I2_Co_L3

Co	-0.02696	-0.28285	0.03555
H	1.74831	-2.58396	-0.53478
P	-2.19768	-0.20320	-0.09496
C	-2.33588	1.45903	-0.96412
C	-1.19593	2.29363	-0.51309
N	-1.27839	3.61579	-0.56617
C	-0.19298	4.28528	-0.20161
N	0.93622	3.74024	0.23154
C	0.94570	2.41810	0.32562
N	-0.09803	1.63559	-0.05342
C	2.13365	1.71479	0.86344
P	2.12308	-0.04036	0.18713
N	3.21891	-0.86680	1.20161
C	4.46277	-1.19124	0.52482
H	4.43043	-2.22784	0.13134
H	5.31605	-1.12527	1.22120
C	4.58638	-0.19864	-0.61502
H	5.00680	0.76599	-0.25331
H	5.24855	-0.56753	-1.41485

N	3.23529	-0.03689	-1.11933
C	3.04268	0.90065	-2.19332
H	1.98881	0.89613	-2.50966
C	2.76064	-1.80070	2.20350
N	-3.29654	-0.14554	1.21655
C	-4.62283	-0.51510	0.75689
H	-5.14374	0.34459	0.27944
H	-5.24101	-0.84658	1.60673
C	-4.39048	-1.62662	-0.24790
H	-4.23596	-2.59138	0.27497
H	-5.24811	-1.74832	-0.93131
N	-3.19634	-1.25121	-0.98767
C	-2.62163	-2.26929	-1.84396
H	-2.31492	-3.16797	-1.27991
C	-3.20073	0.93212	2.16364
H	-3.58524	1.89495	1.76425
C	0.73691	-3.02310	-0.28757
O	-0.14624	-2.16040	0.07655
O	0.56786	-4.22086	-0.38988
H	-0.23244	5.37862	-0.26297
H	-3.35416	-2.55443	-2.61527
H	3.65473	0.61144	-3.06135
H	1.74809	-1.53405	2.53708
H	2.72594	-2.83738	1.81873
H	3.42728	-1.77986	3.08043
H	-3.77963	0.69256	3.06880
H	-2.15187	1.07383	2.46483
H	-1.72881	-1.87874	-2.35070
H	3.32265	1.93948	-1.91603
H	3.05054	2.30563	0.72123
H	-2.18696	1.19535	-2.02629
H	1.99877	1.56815	1.94958
H	-3.28754	2.00537	-0.88759

I2_Co_L4

Co	-0.03647	-0.34783	0.00534
H	1.65009	-2.80673	0.10854
P	-2.16800	-0.22413	-0.02074
N	-2.38605	1.50464	-0.19247
C	-1.24335	2.26418	-0.10947
C	-1.29421	3.66456	-0.13121
C	-0.10373	4.37003	-0.04866
C	1.10509	3.70150	0.05949
C	1.09181	2.29918	0.08736
N	-0.06512	1.59273	-0.00060
N	2.24585	1.56828	0.20388
P	2.09540	-0.18375	0.05382
N	3.19952	-0.75933	1.22405
C	4.28814	-1.53292	0.65601
H	4.03939	-2.61366	0.65292
H	5.20431	-1.40982	1.25891
C	4.47404	-1.02465	-0.76434
H	5.18885	-0.17467	-0.80149
H	4.87903	-1.81044	-1.42392
N	3.15318	-0.63249	-1.20312
C	2.99333	-0.10375	-2.52795
H	1.93067	0.11092	-2.71661
C	2.72795	-1.16275	2.52623
N	-3.21054	-0.73270	1.21226
C	-4.41661	-1.38279	0.75203
H	-5.27590	-0.67937	0.71501
H	-4.69613	-2.20214	1.43524
C	-4.06998	-1.90511	-0.63021
H	-3.56460	-2.88895	-0.55399
H	-4.96464	-2.02907	-1.26293
N	-3.17525	-0.91686	-1.21330
C	-2.57759	-1.28277	-2.47941
H	-2.01050	-2.22902	-2.40986
C	-3.14926	-0.19345	2.53881
H	-3.87582	0.62934	2.69516
C	-3.68265	2.12424	-0.29931
H	-3.92952	2.72340	0.59415
H	-4.43855	1.33906	-0.41490
H	-3.74916	2.77601	-1.18552
C	3.51932	2.22924	0.33787
H	3.80724	2.77255	-0.57925
H	4.28538	1.47811	0.55920
H	3.51430	2.94353	1.17693
C	0.58518	-3.16522	-0.00290
O	-0.28547	-2.22090	-0.06616
O	0.36504	-4.35724	-0.05546
H	-0.11878	5.46223	-0.06987
H	-3.36260	-1.39011	-3.24405
H	3.32870	-0.83498	-3.28075
H	1.85168	-0.56328	2.81140
H	2.43515	-2.22985	2.55160
H	3.51172	-1.00303	3.28410
H	-3.35425	-0.97426	3.28913
H	-2.14057	0.20120	2.73345
H	-1.88760	-0.49327	-2.81042
H	3.56703	0.83266	-2.68356
H	2.04399	4.24871	0.11527
H	-2.24777	4.18268	-0.21181

I2_Co_L5

Co	-1.061504000	0.179860000	0.002259000
H	-3.717893000	-1.133980000	0.127783000
P	-0.583611000	2.275344000	-0.026076000
N	1.183292000	2.189607000	-0.155690000
C	1.706625000	0.946847000	-0.073640000
N	3.033211000	0.790107000	-0.075331000
C	3.443714000	-0.461182000	0.004780000
N	2.705723000	-1.546759000	0.082474000
C	1.383705000	-1.326461000	0.085159000
N	0.834742000	-0.090642000	0.010014000
N	0.530686000	-2.367063000	0.164948000
P	-1.206513000	-1.963728000	0.047453000
N	-1.898796000	-2.973085000	1.228375000
C	-2.776416000	-3.989795000	0.673994000
H	-3.828091000	-3.639133000	0.685589000
H	-2.730461000	-4.910950000	1.279409000
C	-2.306652000	-4.227364000	-0.751892000
H	-1.530680000	-5.019669000	-0.796868000
H	-3.136135000	-4.551191000	-1.401817000
N	-1.791616000	-2.950106000	-1.200650000
C	-1.297872000	-2.832169000	-2.544881000
H	-0.982238000	-1.795540000	-2.735499000
C	-2.242973000	-2.454187000	2.530445000
N	-0.916466000	3.385021000	1.198941000
C	-1.255666000	4.710334000	0.725730000
H	-0.371769000	5.381753000	0.717252000
H	-2.008639000	5.169013000	1.387346000
C	-1.801522000	4.493471000	-0.673809000
H	-2.879253000	4.238134000	-0.632339000
H	-1.688082000	5.389364000	-1.306231000
N	-1.039814000	3.384805000	-1.230626000
C	-1.493981000	2.879441000	-2.508227000
H	-2.550670000	2.557905000	-2.472515000
C	-0.477508000	3.198259000	2.552511000
H	0.469609000	3.731071000	2.766955000
C	2.047446000	3.348568000	-0.228593000
H	2.518532000	3.563519000	0.743093000
H	1.444380000	4.208672000	-0.543688000
H	2.846918000	3.188575000	-0.963257000
C	1.020601000	-3.726609000	0.241731000
H	1.230000000	-4.140696000	-0.757512000

H	0.259262000	-4.341910000	0.737894000
H	1.946957000	-3.765610000	0.826915000
C	-3.925630000	-0.029645000	0.012919000
O	-2.866877000	0.701200000	-0.064627000
O	-5.072999000	0.356484000	-0.028824000
H	-1.383412000	3.658635000	-3.277890000
H	-2.088888000	-3.082010000	-3.269324000
H	-1.573145000	-1.623885000	2.795166000
H	-3.283300000	-2.079234000	2.564233000
H	-2.133709000	-3.237449000	3.297234000
H	-1.239417000	3.559133000	3.261677000
H	-0.319450000	2.126987000	2.747404000
H	-0.882762000	2.016960000	-2.809957000
H	-0.433796000	-3.498275000	-2.738819000
C	4.959534000	-0.632897000	-0.004311000
F	5.508906000	0.051676000	0.998157000
F	5.322812000	-1.899578000	0.115450000
F	5.467221000	-0.162653000	-1.144157000

I2_Co_L6

Co	-0.13629	0.71428	0.00003
H	-2.32225	2.72844	-0.00009
P	1.99301	1.02529	-0.00021
N	2.57573	-0.62679	-0.00067
C	1.60360	-1.57523	-0.00018
N	1.95605	-2.86229	0.00030
C	0.94918	-3.72154	0.00061
N	-0.34027	-3.42532	0.00046
C	-0.62127	-2.11972	0.00000
N	0.31774	-1.14456	-0.00019
N	-1.91676	-1.72293	-0.00027
P	-2.18736	0.02088	-0.00023
C	3.96681	-1.03214	-0.00019
H	4.20432	-1.63375	0.88899
H	4.60185	-0.13782	-0.00223
H	4.20367	-1.63732	-0.88707
C	-2.95707	-2.73189	0.00009
H	-2.88184	-3.37740	-0.88679
H	-3.93892	-2.24469	-0.00154
H	-2.88361	-3.37513	0.88879
C	-1.35957	3.32551	0.00046
O	-0.29840	2.60222	0.00082
O	-1.42108	4.53583	0.00064
H	1.21054	-4.78674	0.00103
C	2.83150	1.81051	1.41681
H	3.92598	1.85004	1.30955
H	2.43934	2.83766	1.47436
H	2.56052	1.28653	2.34280
C	-3.33091	0.21842	1.41836
H	-2.80272	-0.03900	2.34616
H	-3.63032	1.27510	1.47490
H	-4.23811	-0.39694	1.32341
C	2.83121	1.81142	-1.41688
H	2.56051	1.28775	-2.34313
H	2.43852	2.83840	-1.47396
H	3.92566	1.85147	-1.30956
C	-3.33027	0.21867	-1.41930
H	-4.23763	-0.39653	-1.32479
H	-3.62949	1.27540	-1.47586
H	-2.80173	-0.03875	-2.34689

I2_Co_L7

Co	0.03889	-0.50284	-0.00500
H	-1.70727	-2.87405	-0.02084
P	2.16349	-0.32120	-0.00578
N	2.39348	1.39007	0.05556
C	1.24105	2.11604	0.02787
N	1.31351	3.44460	0.03345
C	0.14582	4.06942	0.00928
N	-1.04870	3.50299	-0.01835
C	-1.04810	2.17060	-0.02756
N	0.07919	1.42085	-0.00410
N	-2.22077	1.48657	-0.06220
P	-2.07243	-0.25207	-0.01482
O	-3.14727	-0.66816	-1.20104
C	-4.23930	-1.42820	-0.70832
H	-4.01933	-2.50259	-0.82093
H	-5.13043	-1.18676	-1.30393
C	-4.39660	-1.03781	0.75740
H	-5.07968	-0.18023	0.88030
H	-4.76443	-1.86921	1.37330
O	-3.09832	-0.68139	1.20716
O	3.13906	-0.85365	-1.22234
C	4.29133	-1.54411	-0.75582
H	5.17177	-0.88428	-0.83820
H	4.45197	-2.42835	-1.38655
C	3.99166	-1.90873	0.69200
H	3.43540	-2.85848	0.75958
H	4.89432	-1.97023	1.31339
O	3.18108	-0.84666	1.18103
C	3.67794	2.06166	0.08113
H	3.84740	2.62284	-0.84840
H	4.46494	1.30900	0.20263
H	3.72735	2.76509	0.92252
C	-3.50052	2.16615	-0.05826
H	-3.95089	2.15183	0.94605
H	-4.17666	1.66981	-0.76754
H	-3.36137	3.20772	-0.36608
C	-0.64996	-3.27436	0.01263
O	0.25793	-2.35828	0.01824
O	-0.46856	-4.46955	0.03859
H	0.17280	5.16544	0.01329

I2_Co_L8

Co	-0.48218	0.10476	0.10501
H	-2.93723	0.41682	-1.58042
N	-0.02786	2.07147	0.10664
C	1.33275	2.20965	-0.45862
C	2.10099	0.94086	-0.25879
N	3.40884	0.82688	-0.34843
C	3.89004	-0.41371	-0.22263
N	3.15800	-1.50945	0.00076
C	1.86041	-1.31607	0.10318
N	1.28720	-0.10647	-0.03432
C	0.85916	-2.36908	0.46141
N	-0.50282	-1.92556	0.08914
C	-3.28114	0.20672	-0.52362
O	-2.35422	0.29414	0.36059
O	-4.44771	-0.07824	-0.33014
H	4.97309	-0.54255	-0.30670
H	1.11762	-3.34936	0.02127
H	1.24283	2.37007	-1.54662
H	0.89649	-2.49329	1.55727
H	1.87061	3.08452	-0.05011
C	-0.79451	-2.32376	-1.29143
H	-0.82110	-3.42703	-1.38058
H	-1.76774	-1.91884	-1.59545
H	-0.02491	-1.92805	-1.96791
C	-0.98380	2.89633	-0.63180
H	-1.04112	2.55184	-1.67287
H	-1.97438	2.78813	-0.17488
H	-0.68486	3.96140	-0.61213
C	-1.50082	-2.50033	0.99620

H	-1.32585	-2.12820	2.01391
H	-2.50230	-2.17555	0.68990
H	-1.44720	-3.60518	0.98760
C	-0.03256	2.45291	1.52314
H	0.18491	3.53280	1.63623
H	-1.01710	2.23030	1.95358
H	0.72591	1.87720	2.07025

I2_Co_L9

Co	0.19076	-0.70327	0.00124
H	2.51530	-2.65948	-0.00176
P	-1.90080	-1.11013	0.00071
N	-2.68365	0.42320	0.00009
C	-1.76602	1.49076	0.00005
C	-2.20815	2.82419	-0.00086
C	-1.26590	3.85059	-0.00089
C	0.09775	3.56313	0.00022
C	0.51283	2.22058	0.00131
C	-0.39497	1.13651	0.00100
N	1.87351	1.87652	0.00271
P	2.14063	0.16268	0.00031
C	-4.09480	0.64726	-0.00188
H	-4.42966	1.21407	0.88888
H	-4.62996	-0.31281	-0.00161
H	-4.42747	1.21244	-0.89452
C	2.88897	2.88160	-0.00007
H	2.82954	3.53346	-0.89384
H	3.88537	2.41914	0.00188
H	2.82876	3.53858	0.88977
C	1.58347	-3.30502	-0.00097
O	0.49461	-2.65157	0.00103
O	1.73188	-4.52126	-0.00209
H	-1.59988	4.89306	-0.00176
C	-2.66175	-2.02230	1.40626
H	-3.75189	-2.15522	1.30772
H	-2.17916	-3.01122	1.44338
H	-2.43342	-1.48837	2.33905
C	3.32184	-0.04235	1.40814
H	2.78939	0.19090	2.34084
H	3.64708	-1.09279	1.44795
H	4.21337	0.60044	1.32215
C	-2.65949	-2.02241	-1.40602
H	-2.43050	-1.48787	-2.33830
H	-2.17603	-3.01090	-1.44304
H	-3.74964	-2.15625	-1.30882
C	3.31750	-0.03943	-1.41167
H	4.20982	0.60237	-1.32646
H	3.64165	-1.09004	-1.45556
H	2.78244	0.19700	-2.34209
H	0.82237	4.38180	0.00017
H	-3.27396	3.06766	-0.00169

I2_Co_L10

Co	0.57586	0.02053	-0.15036
H	2.99896	-0.88178	1.32564
O	0.22967	1.99910	-0.39453
C	-1.07893	2.40368	0.01003
C	-1.95867	1.18870	0.04733
C	-3.34545	1.17883	0.16970
C	-4.02186	-0.04716	0.26390
C	-3.30934	-1.25503	0.22455
C	-1.92469	-1.22727	0.07949
C	-1.23055	-0.01082	-0.00497
C	-1.02193	-2.41595	-0.05561
O	0.33354	-1.98296	0.11255
C	3.41681	-0.20160	0.51975
O	2.55540	0.20206	-0.32055
O	4.61860	0.05455	0.55337
H	-5.11098	-0.06095	0.36931
H	-1.22937	-3.22209	0.67777
H	-0.99774	2.86659	1.01686
H	-1.11007	-2.86582	-1.06750
H	-1.43938	3.19308	-0.68193
H	-3.85187	-2.20631	0.30006
H	-3.91679	2.11555	0.20249
C	1.27022	-2.88095	-0.41753
H	1.17105	-2.94302	-1.51742
H	2.27080	-2.50631	-0.17366
H	1.13161	-3.88830	0.01807
C	1.22930	2.94956	-0.13832
H	1.30910	3.14531	0.94766
H	2.17761	2.52818	-0.49360
H	1.00166	3.89771	-0.66061

I2_Co_L11

Co	0.50562	0.05305	0.11850
H	3.08467	0.90080	-1.32666
N	0.38137	-1.98794	0.12726
C	-0.90720	-2.36930	-0.51077
C	-1.92277	-1.28379	-0.28798
C	-3.30927	-1.37768	-0.37158
C	-4.09009	-0.21886	-0.22520
C	-3.48250	1.02312	0.02158
C	-2.09605	1.09520	0.12920
C	-1.30065	-0.05042	-0.03451
C	-1.25766	2.28340	0.50857
N	0.14551	2.06241	0.06193
C	3.40472	0.25896	-0.44572
O	2.49249	0.03289	0.40239
O	4.57196	-0.12953	-0.42679
H	-5.18009	-0.28492	-0.30156
H	-1.63217	3.25515	0.11689
H	-0.70397	-2.46731	-1.59210
H	-1.22494	2.37585	1.60890
H	-1.22560	-3.37379	-0.15388
H	-4.10810	1.91705	0.14190
H	-3.80115	-2.34023	-0.56241
C	0.27939	2.44523	-1.33991
H	0.14355	3.54335	-1.46035
H	1.27402	2.16112	-1.70931
H	-0.47606	1.91804	-1.93617
C	1.50108	-2.64391	-0.53338
H	1.56995	-2.29097	-1.57220
H	2.43464	-2.37611	-0.02126
H	1.37612	-3.74783	-0.52741
C	1.07343	2.82950	0.87741
H	1.03543	2.46545	1.91318
H	2.09570	2.67933	0.50695
H	0.83043	3.91389	0.85445
C	0.36752	-2.34556	1.54169
H	0.34827	-3.45157	1.66516
H	1.26416	-1.93999	2.02900
H	-0.51994	-1.91342	2.02180

I2_Fe_L1

Fe	0.00395	-0.49234	-0.39282
H	1.79658	-2.90713	-0.09216
P	-2.05800	-0.37754	0.05154
N	-2.41799	1.31680	-0.44561
C	-1.31903	2.09463	-0.58188
N	-1.44646	3.41339	-0.76429

C	-0.30665	4.08996	-0.79041
N	0.90368	3.59318	-0.58515
C	0.95746	2.26774	-0.41030
N	-0.12511	1.44487	-0.49383
N	2.12506	1.65332	-0.11150
P	1.99540	-0.11844	0.14861
N	2.79376	-0.30096	1.70202
C	4.05334	-1.00330	1.63816
H	3.89962	-2.09546	1.78836
H	4.74205	-0.66165	2.43474
C	4.62195	-0.74817	0.25207
H	5.24769	0.17377	0.23939
H	5.27803	-1.57886	-0.06916
N	3.47244	-0.65161	-0.59888
C	3.64581	-0.58856	-2.01381
H	2.65799	-0.60672	-2.50192
C	1.98451	-0.61065	2.84823
N	-2.83979	-0.47600	1.60993
C	-4.23596	-0.81922	1.55757
H	-4.88554	0.08355	1.49131
H	-4.54349	-1.35836	2.47306
C	-4.38593	-1.69353	0.32296
H	-4.19452	-2.75841	0.57821
H	-5.41420	-1.64222	-0.08641
N	-3.41489	-1.21851	-0.63049
C	-3.22701	-2.02457	-1.80759
H	-3.08499	-3.09360	-1.55713
C	-2.41768	0.41439	2.64733
H	-2.94620	1.39241	2.61786
C	-3.72876	1.90751	-0.49605
H	-3.97760	2.46060	0.42751
H	-4.46522	1.10722	-0.65013
H	-3.79650	2.61946	-1.33072
C	3.34688	2.38921	0.07131
H	4.05600	2.20758	-0.75454
H	3.82664	2.07641	1.01236
H	3.13211	3.46432	0.11548
C	0.93178	-3.23132	-0.74237
O	0.00029	-2.36029	-0.84908
O	0.95952	-4.34338	-1.24886
H	-0.37471	5.16932	-0.98098
H	-4.09065	-1.93929	-2.49235
H	4.22005	-1.45769	-2.38259
H	0.98522	-0.16775	2.72704
H	1.84648	-1.70497	2.97829
H	2.43933	-0.21268	3.77330
H	-2.58660	-0.02788	3.64519
H	-1.33900	0.61093	2.53601
H	-2.32261	-1.69980	-2.34093
H	4.17281	0.33069	-2.35115

I2_Fe_L2

Fe	-0.00231	0.60987	-0.48076
H	-1.82525	2.98343	-0.42161
P	1.95828	0.31983	0.13020
O	2.34649	-1.26026	-0.85212
C	1.26918	-1.92244	-1.11364
N	1.34649	-3.18587	-1.54115
C	0.18496	-3.81644	-1.63331
N	-1.00532	-3.35884	-1.27409
C	-1.01709	-2.08703	-0.86637
N	0.08337	-1.29369	-0.88892
O	-2.10830	-1.56905	-0.40585
P	-1.89553	0.17356	0.23921
N	-2.34089	-0.08432	1.87643
C	-3.74952	0.01164	2.16795
H	-3.96515	0.96818	2.69102
H	-4.08620	-0.80495	2.83440
C	-4.45696	-0.03704	0.82400
H	-4.67084	-1.08791	0.53040
H	-5.42071	0.50221	0.84837
N	-3.54442	0.58324	-0.10732
C	-3.96356	0.60122	-1.48077
H	-3.16036	1.01935	-2.10672
C	-1.42426	0.08901	2.96362
N	2.62122	-0.32365	1.60114
C	4.06839	-0.32873	1.55801
H	4.43940	-1.22742	1.01885
H	4.48949	-0.35662	2.57854
C	4.46148	0.93712	0.81405
H	4.55894	1.79696	1.51047
H	5.43575	0.82076	0.30312
N	3.39665	1.17653	-0.12693
C	3.46124	2.34036	-0.96766
H	3.69491	3.24359	-0.37294
C	2.02215	-1.51124	2.14786
H	2.40240	-2.43336	1.66156
C	-0.87320	3.34047	-0.90988
O	0.09068	2.49548	-0.85235
O	-0.84101	4.44969	-1.41875
H	0.21569	-4.84160	-2.02734
H	4.23750	2.23674	-1.74760
H	-4.85773	1.23566	-1.60367
H	-0.41366	0.26471	2.56261
H	-1.69994	0.96583	3.58202
H	-1.39493	-0.79475	3.62866
H	2.22392	-1.58613	3.22993
H	0.93127	-1.47654	2.00299
H	2.48617	2.50576	-1.44871
H	-4.20145	-0.41359	-1.86045

I2_Fe_L3

Fe	-0.00323	-0.33820	-0.35923
H	2.05560	-2.52279	-0.64904
P	-2.14575	-0.30019	-0.07298
C	-2.50530	1.30123	-0.99071
C	-1.33028	2.18124	-0.77328
N	-1.43021	3.48574	-0.94667
C	-0.35421	4.21767	-0.66486
N	0.76212	3.72195	-0.12627
C	0.80782	2.41798	0.05399
N	-0.17895	1.53931	-0.35445
C	1.94562	1.79136	0.77525
P	2.01671	0.00620	0.22622
N	2.84656	-0.77505	1.54693
C	4.23574	-1.05699	1.26959
H	4.36117	-2.10675	0.92374
H	4.86290	-0.93405	2.17349
C	4.64823	-0.10157	0.16575
H	4.90467	0.89860	0.59111
H	5.54317	-0.46119	-0.37400
N	3.50837	-0.03962	-0.71197
C	3.60921	0.85379	-1.82811
H	2.66376	0.84737	-2.39327
C	2.15427	-1.74883	2.34800
N	-2.93352	-0.07074	1.47555
C	-4.35690	-0.24851	1.31647
H	-4.84255	0.65478	0.87722
H	-4.83845	-0.42859	2.29432
C	-4.50630	-1.43943	0.38656
H	-4.40886	-2.38368	0.96550
H	-5.50195	-1.45636	-0.09754
N	-3.44108	-1.33398	-0.58386
C	-3.14728	-2.52808	-1.33767
H	-3.03285	-3.41380	-0.68124
C	-2.52386	1.04983	2.27473

H	-2.90579	2.02113	1.88889
C	1.06899	-2.99092	-0.93569
O	0.05994	-2.21062	-0.82591
O	1.05662	-4.15272	-1.31441
H	-0.40170	5.29648	-0.84800
H	-3.95106	-2.74168	-2.06517
H	4.41591	0.53458	-2.51062
H	1.07198	-1.55034	2.31833
H	2.31613	-2.78335	1.98107
H	2.48923	-1.70792	3.40035
H	-2.88903	0.93640	3.30983
H	-1.42397	1.09970	2.30143
H	-2.19742	-2.41328	-1.87678
H	3.82137	1.90526	-1.53006
H	2.86658	2.38519	0.66971
H	-2.55528	0.96626	-2.04197
H	1.69925	1.74529	1.85191
H	-3.44304	1.83667	-0.77012

I2_Fe_L4

Fe	0.02332	-0.49445	-0.43288
H	1.82260	-2.94919	-0.15314
P	-2.01278	-0.44866	0.07555
N	-2.45665	1.17436	-0.57792
C	-1.39679	2.02924	-0.69899
C	-1.55536	3.40560	-0.92552
C	-0.42405	4.21303	-0.94936
C	0.82587	3.66139	-0.69950
C	0.92011	2.27772	-0.47692
N	-0.16649	1.45496	-0.54611
N	2.09809	1.66610	-0.15409
P	1.97386	-0.08533	0.18578
N	2.71022	-0.18930	1.79164
C	3.88868	-1.02091	1.81975
H	3.61624	-2.08985	1.97469
H	4.55744	-0.73542	2.65432
C	4.56450	-0.85375	0.46933
H	5.28402	-0.00064	0.48541
H	5.15451	-1.75159	0.20366
N	3.48803	-0.65903	-0.45393
C	3.75516	-0.62530	-1.85409
H	2.80128	-0.58386	-2.40447
C	1.82502	-0.37370	2.90830
N	-2.74403	-0.39843	1.67189
C	-4.13190	-0.77449	1.69743
H	-4.80485	0.10093	1.54237
H	-4.40759	-1.20805	2.67732
C	-4.29168	-1.78960	0.57736
H	-4.05817	-2.81204	0.94694
H	-5.33413	-1.81691	0.20170
N	-3.36711	-1.40002	-0.45681
C	-3.19036	-2.32929	-1.54153
H	-3.02211	-3.36130	-1.17586
C	-2.33926	0.65126	2.55624
H	-2.90279	1.59676	2.39115
C	-3.79764	1.66631	-0.68940
H	-4.06915	2.36987	0.12345
H	-4.48687	0.81276	-0.66309
H	-3.95523	2.18765	-1.64991
C	3.28893	2.44043	0.03534
H	3.59407	2.95714	-0.89304
H	4.10637	1.77587	0.33374
H	3.17239	3.20385	0.82738
C	1.04554	-3.19035	-0.93537
O	0.10723	-2.32681	-1.02441
O	1.16124	-4.22175	-1.58356
H	-0.52126	5.28616	-1.13690
H	-4.07215	-2.34009	-2.20859
H	4.29247	-1.53396	-2.18160
H	0.89147	0.18227	2.74069
H	1.54604	-1.43926	3.05486
H	2.29050	-0.00938	3.84184
H	-2.47618	0.35944	3.61273
H	-1.27097	0.86797	2.39435
H	-2.30345	-2.05340	-2.12893
H	4.36143	0.25595	-2.16010
H	1.71815	4.28634	-0.67006
H	-2.54933	3.83017	-1.06351

I2_Fe_L5

Fe	-1.08389	0.24018	-0.02731
H	-3.81505	-0.96285	0.06411
P	-0.45929	2.28457	-0.02640
N	1.27674	2.13269	-0.16883
C	1.74608	0.85073	-0.09297
N	3.04675	0.63039	-0.09600
C	3.42026	-0.64533	-0.01509
N	2.59599	-1.68333	0.07045
C	1.30619	-1.39346	0.07258
N	0.77624	-0.12553	-0.01260
N	0.38328	-2.39390	0.16858
P	-1.29502	-1.87610	0.06040
N	-2.05587	-2.88041	1.25847
C	-2.99032	-3.83998	0.72015
H	-4.02184	-3.42513	0.70794
H	-3.01793	-4.75621	1.33990
C	-2.52586	-4.13503	-0.69608
H	-1.78284	-4.96483	-0.71002
H	-3.36792	-4.45437	-1.33712
N	-1.96403	-2.89857	-1.17094
C	-1.41632	-2.87244	-2.49348
H	-1.00460	-1.87248	-2.70052
C	-2.39344	-2.31552	2.53671
N	-0.71019	3.43029	1.23835
C	-0.90340	4.78647	0.79998
H	0.05135	5.36014	0.77753
H	-1.58555	5.32584	1.48203
C	-1.49568	4.65882	-0.59114
H	-2.59412	4.50475	-0.52426
H	-1.32259	5.56694	-1.19779
N	-0.85124	3.51088	-1.18983
C	-1.39622	3.08384	-2.45499
H	-2.48444	2.88866	-2.39561
C	-0.17839	3.19767	2.54602
H	0.82927	3.64516	2.68316
C	2.19809	3.23747	-0.23655
H	2.66504	3.44673	0.74137
H	1.65313	4.12657	-0.58005
H	3.00723	3.02009	-0.94762
C	0.81287	-3.76348	0.28200
H	1.08731	-4.19645	-0.69581
H	-0.00143	-4.35252	0.72439
H	1.69588	-3.83332	0.93198
C	-3.98310	0.14534	-0.07239
O	-2.90424	0.83565	-0.13665
O	-5.12602	0.56379	-0.14441
H	-1.21630	3.85184	-3.22693
H	-2.19470	-3.08349	-3.24749
H	-1.68637	-1.51077	2.78661
H	-3.41479	-1.88300	2.54806
H	-2.33997	-3.08195	3.33013
H	-0.84139	3.61642	3.32320
H	-0.09514	2.11357	2.72013
H	-0.90716	2.15272	-2.77608
H	-0.60051	-3.61372	-2.63383
C	4.89933	-0.89123	-0.03147

F	5.53118	-0.25370	0.97361
F	5.21096	-2.18228	0.07208
F	5.47556	-0.43919	-1.16372

I2_Fe_L6

Fe	0.17085	-0.72286	-0.00033
H	2.45605	-2.68917	0.00068
P	-1.94111	-1.08459	0.00002
N	-2.60362	0.52700	-0.00506
C	-1.66103	1.52561	-0.00164
N	-2.06443	2.78715	0.00070
C	-1.10183	3.70727	0.00217
N	0.20265	3.43914	0.00166
C	0.53146	2.15507	0.00002
N	-0.35345	1.10527	-0.00098
N	1.85291	1.80272	-0.00055
P	2.15497	0.06755	-0.00054
C	-3.99836	0.88053	-0.00284
H	-4.26903	1.45748	0.89699
H	-4.60657	-0.03367	-0.02999
H	-4.25706	1.50253	-0.87513
C	2.84401	2.84581	0.00005
H	2.74483	3.49647	-0.88443
H	3.84801	2.40228	-0.00322
H	2.74869	3.49233	0.88804
C	1.51966	-3.32755	0.00105
O	0.43617	-2.65720	0.00155
O	1.64793	-4.54399	0.00116
H	-1.40487	4.76061	0.00398
C	-2.81711	-1.90634	1.40220
H	-3.91207	-1.95722	1.28092
H	-2.41564	-2.93026	1.46236
H	-2.56499	-1.38376	2.33500
C	3.36380	-0.07020	1.40046
H	2.83989	0.16224	2.33788
H	3.71190	-1.11330	1.45011
H	4.24232	0.58735	1.29228
C	-2.81564	-1.91552	-1.39731
H	-2.56633	-1.39643	-2.33280
H	-2.40963	-2.93794	-1.45271
H	-3.91026	-1.97111	-1.27519
C	3.36352	-0.07029	-1.40180
H	4.24364	0.58495	-1.29261
H	3.70910	-1.11412	-1.45348
H	2.83995	0.16509	-2.33869

I2_Fe_L7

Fe	0.00515	-0.58900	-0.38909
H	1.89712	-2.90688	-0.30196
P	-2.02575	-0.48756	0.10707
N	-2.43184	1.20518	-0.17799
C	-1.34780	2.00606	-0.32876
N	-1.49573	3.33038	-0.40104
C	-0.36524	4.02031	-0.44597
N	0.85638	3.52228	-0.33478
C	0.92595	2.19094	-0.25748
N	-0.14939	1.36224	-0.36903
N	2.10699	1.56897	-0.03089
P	1.97490	-0.18553	0.13654
O	2.77678	-0.36363	1.62926
C	3.97166	-1.09000	1.54058
H	3.77594	-2.16916	1.68826
H	4.66221	-0.75557	2.33201
C	4.52683	-0.82594	0.14452
H	5.18795	0.06324	0.14281
H	5.10916	-1.68159	-0.23434
O	3.41411	-0.61592	-0.67904
O	-2.77686	-0.77817	1.60538
C	-4.08835	-1.27006	1.51851
H	-4.82017	-0.44595	1.63025
H	-4.26309	-1.99274	2.33145
C	-4.19619	-1.91429	0.14207
H	-3.85657	-2.96654	0.17091
H	-5.22742	-1.89424	-0.24748
O	-3.36681	-1.15837	-0.69644
C	-3.74964	1.77081	-0.03913
H	-3.95254	2.08773	0.99855
H	-4.49168	1.01918	-0.33968
H	-3.84835	2.65157	-0.68615
C	3.33716	2.29844	0.13553
H	4.04368	2.06276	-0.67643
H	3.80533	2.03339	1.09654
H	3.12703	3.37439	0.12595
C	0.95741	-3.29575	-0.79401
O	0.00133	-2.44436	-0.84411
O	0.93839	-4.44504	-1.20316
H	-0.45079	5.10838	-0.56251

I2_Fe_L8

Fe	-0.50493	-0.03073	0.14252
H	-3.18345	-0.20656	-1.66996
N	-0.31522	2.04609	0.14821
C	0.96761	2.31475	-0.54207
C	1.93807	1.21067	-0.26573
N	3.22913	1.28666	-0.39723
C	3.92986	0.13047	-0.28501
N	3.35110	-1.07021	-0.03680
C	2.05957	-1.08378	0.11371
N	1.25034	0.03759	0.00582
C	1.23989	-2.25368	0.55489
N	-0.15894	-2.08749	0.09479
C	-3.44736	-0.09468	-0.57549
O	-2.44397	-0.09772	0.20234
O	-4.63180	0.01417	-0.27315
H	5.01548	0.16911	-0.40005
H	1.66953	-3.22224	0.22955
H	0.74324	2.31031	-1.62597
H	1.20424	-2.25379	1.66129
H	1.37328	3.31397	-0.28518
C	-0.28195	-2.49481	-1.29872
H	-0.11769	-3.58936	-1.40633
H	-1.28373	-2.23955	-1.67100
H	0.46099	-1.96052	-1.90569
C	-1.41508	2.74218	-0.49848
H	-1.53136	2.36883	-1.52572
H	-2.34664	2.53814	0.04641
H	-1.23989	3.83862	-0.52372
C	-1.08132	-2.84361	0.92487
H	-1.03681	-2.46615	1.95584
H	-2.10543	-2.69652	0.55720
H	-0.83956	-3.92807	0.91737
C	-0.22951	2.43837	1.54951
H	-0.13215	3.54202	1.64551
H	-1.13274	2.10412	2.07882
H	0.64334	1.96125	2.01423

I2_Fe_L9

Fe	0.26473	0.72474	-0.00227
H	2.77495	2.49017	0.00723
P	-1.78843	1.25404	-0.00064

N	-2.71644	-0.21212	0.00483
C	-1.88862	-1.35290	0.00166
C	-2.44184	-2.64221	0.00216
C	-1.59798	-3.75918	-0.00006
C	-0.21024	-3.56890	-0.00345
C	0.31512	-2.26772	-0.00495
C	-0.48417	-1.08189	-0.00196
N	1.70050	-2.04252	-0.00933
P	2.09017	-0.33163	0.00062
C	-4.13309	-0.31788	0.00557
H	-4.52085	-0.85582	-0.88702
H	-4.58975	0.68319	0.00923
H	-4.51888	-0.86120	0.89561
C	2.61949	-3.12571	-0.00291
H	2.50736	-3.77485	0.89357
H	3.65434	-2.75396	-0.00879
H	2.50260	-3.78762	-0.88904
C	1.91621	3.23095	0.00098
O	0.77269	2.69876	-0.00435
O	2.20339	4.43455	0.00048
H	-2.01854	-4.77172	0.00082
C	-2.57497	2.22002	-1.39366
H	-3.65958	2.40266	-1.27075
H	-2.04971	3.18789	-1.44124
H	-2.38911	1.67965	-2.33308
C	3.35590	-0.28581	-1.38643
H	2.82442	-0.47634	-2.33029
H	3.78149	0.72974	-1.42784
H	4.18211	-1.01252	-1.27130
C	-2.57157	2.22848	1.38807
H	-2.38430	1.69362	2.33034
H	-2.04528	3.19609	1.42870
H	-3.65623	2.41150	1.26626
C	3.33803	-0.29632	1.40440
H	4.16543	-1.02245	1.29394
H	3.76300	0.71882	1.45971
H	2.79427	-0.49440	2.33966
H	0.45056	-4.44311	-0.00507
H	-3.52795	-2.78829	0.00469

I2_Fe_L10

Fe	0.59349	0.02320	-0.11059
H	3.31902	-1.14280	1.02396
O	0.19573	2.07854	-0.40566
C	-1.09618	2.41513	0.09843
C	-1.96939	1.19785	0.04403
C	-3.35345	1.20174	0.12706
C	-4.06621	-0.01835	0.19881
C	-3.34410	-1.23322	0.16525
C	-1.96218	-1.21986	0.04650
C	-1.21334	-0.00950	-0.00845
C	-1.09591	-2.42622	-0.13849
O	0.25246	-2.09070	0.20049
C	3.60918	-0.25432	0.37912
O	2.64861	0.30047	-0.21186
O	4.81479	0.04276	0.34849
H	-5.15949	-0.02146	0.27980
H	-1.41033	-3.30712	0.46619
H	-0.95998	2.74871	1.15306
H	-1.09333	-2.74153	-1.20785
H	-1.49471	3.28220	-0.47634
H	-3.89117	-2.18782	0.21973
H	-3.90845	2.15288	0.15291
C	1.17851	-3.01412	-0.27690
H	1.18559	-3.02557	-1.38627
H	2.17177	-2.70648	0.07679
H	0.94948	-4.03701	0.09584
C	1.18195	3.01954	-0.11429
H	1.29919	3.13666	0.98259
H	2.12715	2.63905	-0.52416
H	0.92873	4.00639	-0.55899

I2_Fe_L11

Fe	-0.53212	0.03782	0.04000
H	-3.39777	-1.14470	-0.87911
N	-0.16136	2.12447	0.06075
C	1.16761	2.31256	-0.57209
C	2.05559	1.14322	-0.25923
C	3.44106	1.11448	-0.26914
C	4.13047	-0.10987	-0.08446
C	3.38281	-1.29199	0.13790
C	1.99799	-1.24242	0.17892
C	1.27605	-0.03078	-0.03468
C	1.05976	-2.34518	0.57287
N	-0.29043	-2.08801	0.01067
C	-3.60629	-0.22399	-0.24534
O	-2.58575	0.32313	0.24022
O	-4.79802	0.10543	-0.12546
H	5.22634	-0.14000	-0.10587
H	1.40217	-3.37065	0.28804
H	0.97491	2.34279	-1.66182
H	0.92874	-2.33688	1.67246
H	1.59219	3.30526	-0.28232
H	3.91403	-2.24301	0.30141
H	4.01782	2.03720	-0.43967
C	-0.33258	-2.48792	-1.38478
H	-0.22702	-3.59927	-1.48245
H	-1.28372	-2.16683	-1.83415
H	0.48284	-1.98942	-1.92597
C	-1.18304	2.88954	-0.62313
H	-1.26973	2.52594	-1.65880
H	-2.14946	2.72386	-0.12665
H	-0.94311	3.98135	-0.62625
C	-1.30262	-2.79052	0.76969
H	-1.33426	-2.37985	1.79114
H	-2.28636	-2.62883	0.30652
H	-1.09914	-3.88946	0.81060
C	-0.11529	2.49053	1.46475
H	0.04210	3.59383	1.58268
H	-1.05721	2.19747	1.95060
H	0.70529	1.94831	1.95374

I2_Ni_L1

Ni	-0.05195	-0.33138	0.03408
H	1.60009	-2.75519	0.24170
P	-2.22708	-0.18797	-0.02137
N	-2.37550	1.54997	-0.13567
C	-1.22006	2.25409	-0.06623
N	-1.25912	3.58155	-0.08599
C	-0.08175	4.18679	-0.01714
N	1.10395	3.60296	0.06155
C	1.08812	2.27299	0.08182
N	-0.06025	1.55086	0.02465
N	2.24953	1.58622	0.16042
P	2.15945	-0.17218	0.05569
N	3.21678	-0.73667	1.22478
C	4.28583	-1.55659	0.65910
H	4.01729	-2.62855	0.71403
H	5.21071	-1.41601	1.23948
C	4.44844	-1.10593	-0.78441
H	5.18001	-0.27981	-0.87443
H	4.79804	-1.92658	-1.42882
N	3.12190	-0.67509	-1.21132

C	2.92671	-0.22253	-2.56747
H	1.86759	0.02345	-2.73515
C	2.78306	-1.02670	2.57474
N	-3.22216	-0.75250	1.17761
C	-4.37738	-1.48392	0.67056
H	-5.27566	-0.83741	0.66620
H	-4.58838	-2.34744	1.31906
C	-3.99481	-1.92000	-0.73414
H	-3.45432	-2.88471	-0.71561
H	-4.87457	-2.02934	-1.38581
N	-3.12459	-0.86500	-1.25855
C	-2.53140	-1.08618	-2.56271
H	-1.94198	-2.01898	-2.59063
C	-3.19129	-0.29657	2.54427
H	-4.01041	0.41350	2.75679
C	-3.65379	2.24253	-0.20244
H	-3.82805	2.82789	0.71072
H	-4.44723	1.49544	-0.31973
H	-3.67745	2.92563	-1.06080
C	3.51643	2.30068	0.20562
H	3.76697	2.72871	-0.77576
H	4.29953	1.59988	0.51657
H	3.46487	3.11842	0.93418
C	0.54358	-3.10256	0.07541
O	-0.33171	-2.13851	-0.03423
O	0.28453	-4.27247	0.00847
H	-0.09109	5.28271	-0.02890
H	-3.32355	-1.14255	-3.32220
H	3.20163	-1.01694	-3.27621
H	1.95817	-0.35873	2.86083
H	2.44261	-2.07218	2.68246
H	3.60947	-0.85682	3.27974
H	-3.28064	-1.14608	3.23741
H	-2.23702	0.20818	2.75391
H	-1.87645	-0.24328	-2.82780
H	3.53235	0.67296	-2.79832

I2_Ni_L2

Ni	-0.04115	-0.26821	-0.00105
H	1.70298	-2.63279	0.11379
P	-2.22831	-0.12337	-0.03772
O	-2.34338	1.62505	-0.15807
C	-1.22876	2.30623	-0.09160
N	-1.27335	3.62103	-0.10848
C	-0.09872	4.23586	-0.03957
N	1.09208	3.65421	0.04136
C	1.07880	2.33880	0.05688
N	-0.06438	1.60935	-0.00459
O	2.20785	1.68414	0.13226
P	2.15741	-0.07040	0.05736
N	3.15670	-0.56037	1.27338
C	4.42277	-1.09694	0.76858
H	4.38047	-2.20187	0.77303
H	5.25233	-0.78512	1.42033
C	4.57496	-0.55350	-0.64185
H	5.07069	0.43559	-0.64065
H	5.15855	-1.22833	-1.28406
N	3.21040	-0.43429	-1.16653
C	3.03352	0.08238	-2.50807
H	1.96466	0.10686	-2.76564
C	2.70918	-0.96087	2.59084
N	-3.24841	-0.53010	1.19012
C	-4.58038	-0.86380	0.67700
H	-5.21738	0.03985	0.65921
H	-5.05409	-1.60372	1.33795
C	-4.35542	-1.41360	-0.72218
H	-4.16870	-2.50187	-0.70293
H	-5.21661	-1.22287	-1.37969
N	-3.16924	-0.72748	-1.24305
C	-2.66263	-1.11757	-2.54374
H	-2.45815	-2.19993	-2.57936
C	-3.12590	0.00693	2.52779
H	-3.67525	0.95791	2.63756
C	0.64889	-3.01116	0.00436
O	-0.26520	-2.07179	-0.05937
O	0.41669	-4.18458	-0.04945
H	-0.11346	5.33110	-0.05135
H	-3.39349	-0.86145	-3.32348
H	3.53608	-0.57751	-3.22813
H	1.69392	-0.58598	2.78062
H	2.69792	-2.05985	2.68755
H	3.37282	-0.54626	3.36291
H	-3.51943	-0.71520	3.25625
H	-2.06744	0.18242	2.76859
H	-1.72992	-0.58122	-2.76538
H	3.44718	1.10089	-2.61125

I2_Ni_L3

Ni	-0.07094	-0.20614	0.04045
H	1.50041	-2.60011	-0.51417
P	-2.24776	-0.02280	-0.13826
C	-2.30170	1.68750	-0.88116
C	-1.08903	2.43086	-0.44360
N	-1.07443	3.75061	-0.49632
C	0.06398	4.34170	-0.15194
N	1.15485	3.71724	0.27468
C	1.07529	2.40177	0.37307
N	-0.02700	1.70638	-0.00659
C	2.21211	1.62078	0.91680
P	2.15346	-0.11650	0.21047
N	3.14308	-1.04927	1.19509
C	4.31743	-1.53480	0.47346
H	4.13811	-2.55799	0.09138
H	5.18604	-1.57390	1.14880
C	4.53160	-0.55937	-0.66783
H	5.09348	0.33434	-0.32619
H	5.09145	-1.01058	-1.50025
N	3.18930	-0.19513	-1.11866
C	3.07774	0.76780	-2.18892
H	2.02618	0.87947	-2.49208
C	2.63603	-1.85862	2.28347
N	-3.23472	-0.16327	1.20301
C	-4.47581	-0.86051	0.87510
H	-5.26558	-0.14513	0.57345
H	-4.83385	-1.41338	1.75633
C	-4.11228	-1.78825	-0.26754
H	-3.63511	-2.71310	0.10808
H	-4.99029	-2.06918	-0.86805
N	-3.17153	-1.03599	-1.10174
C	-2.58647	-1.75010	-2.22275
H	-2.07271	-2.67023	-1.89551
C	-3.20836	0.77795	2.29454
H	-3.83612	1.66856	2.10288
C	0.44885	-2.94770	-0.31741
O	-0.37193	-2.00946	0.07752
O	0.13600	-4.09713	-0.47492
H	0.10470	5.43393	-0.22363
H	-3.37763	-2.01146	-2.93920
H	3.63711	0.41338	-3.06593
H	1.68681	-1.44936	2.65725
H	2.45762	-2.90233	1.96951
H	3.35364	-1.86526	3.11675
H	-3.57230	0.29622	3.21324
H	-2.17650	1.10980	2.48510
H	-1.85977	-1.11181	-2.74622
H	3.47790	1.76346	-1.91117

H	3.16447	2.15771	0.79858
H	-2.24022	1.51773	-1.97203
H	2.05301	1.47262	2.00064
H	-3.21550	2.27291	-0.70224

I2_Ni_L4

Ni	-0.05380	-0.29135	0.03052
H	1.56243	-2.76876	0.21816
P	-2.21499	-0.17898	-0.02125
N	-2.38927	1.53557	-0.16337
C	-1.23815	2.28506	-0.08773
C	-1.26993	3.68054	-0.11565
C	-0.06850	4.36861	-0.03824
C	1.13654	3.69271	0.06660
C	1.11548	2.29505	0.09674
N	-0.05830	1.61105	0.01973
N	2.26660	1.55507	0.20259
P	2.14656	-0.18016	0.06032
N	3.18964	-0.80099	1.22018
C	4.21974	-1.65968	0.64404
H	3.90504	-2.72003	0.68065
H	5.14931	-1.57140	1.22764
C	4.40582	-1.19422	-0.79207
H	5.17731	-0.40279	-0.86760
H	4.72038	-2.02013	-1.44809
N	3.10373	-0.69434	-1.21304
C	2.94694	-0.17698	-2.54989
H	1.90001	0.11397	-2.72221
C	2.74407	-1.09105	2.56549
N	-3.19827	-0.75400	1.18679
C	-4.30750	-1.56238	0.69915
H	-5.24324	-0.97064	0.68495
H	-4.46754	-2.42492	1.36375
C	-3.90253	-2.00283	-0.69824
H	-3.30610	-2.93356	-0.66113
H	-4.77556	-2.17625	-1.34521
N	-3.09636	-0.90969	-1.24486
C	-2.48787	-1.13423	-2.54136
H	-1.84453	-2.03140	-2.53938
C	-3.18822	-0.27140	2.54312
H	-4.05014	0.38892	2.74836
C	-3.69203	2.16648	-0.24877
H	-3.89765	2.78564	0.63843
H	-4.45691	1.38423	-0.31335
H	-3.77614	2.79040	-1.15101
C	3.55603	2.21239	0.28820
H	3.79575	2.76109	-0.63670
H	4.32791	1.45495	0.46066
H	3.58926	2.91095	1.13729
C	0.49841	-3.08621	0.04527
O	-0.35250	-2.10138	-0.04781
O	0.21056	-4.24917	-0.04264
H	-0.07197	5.46018	-0.06211
H	-3.27351	-1.25988	-3.29944
H	3.20605	-0.94779	-3.29010
H	1.95079	-0.38999	2.86193
H	2.35513	-2.12106	2.65968
H	3.57781	-0.97091	3.27238
H	-3.21918	-1.10961	3.25543
H	-2.26749	0.29845	2.73563
H	-1.88230	-0.26266	-2.83026
H	3.58688	0.70669	-2.73191
H	2.07819	4.23430	0.11982
H	-2.21567	4.21146	-0.19944

I2_Ni_L5

Ni	-1.01604	0.14671	0.05390
H	-3.60678	-1.22261	0.28575
P	-0.63703	2.29377	-0.01357
N	1.11189	2.24852	-0.10913
C	1.68073	1.02483	-0.03959
N	3.00414	0.91395	-0.05715
C	3.46558	-0.32297	0.01052
N	2.76737	-1.43651	0.08153
C	1.44291	-1.27558	0.09935
N	0.85585	-0.05350	0.04847
N	0.63828	-2.35384	0.16773
P	-1.10661	-2.06932	0.06220
N	-1.77829	-3.08092	1.21215
C	-2.70138	-4.05193	0.62698
H	-3.74061	-3.67760	0.68836
H	-2.65472	-4.99646	1.19044
C	-2.26798	-4.23256	-0.81986
H	-1.52075	-5.04243	-0.92483
H	-3.11924	-4.48360	-1.47030
N	-1.70446	-2.94791	-1.22169
C	-1.24743	-2.76315	-2.57815
H	-0.90850	-1.72661	-2.72446
C	-2.01975	-2.64889	2.57259
N	-1.09783	3.36010	1.16569
C	-1.69203	4.58257	0.63497
H	-0.95001	5.40347	0.62828
H	-2.53368	4.89567	1.27060
C	-2.15260	4.23192	-0.77035
H	-3.17194	3.80322	-0.75759
H	-2.15461	5.10905	-1.43448
N	-1.19626	3.24097	-1.27063
C	-1.46793	2.65720	-2.56983
H	-2.45851	2.17117	-2.60023
C	-0.66776	3.29550	2.54011
H	0.12672	4.03226	2.75424
C	1.94368	3.44211	-0.17832
H	2.57172	3.52905	0.71838
H	1.28840	4.31699	-0.25694
H	2.59866	3.40612	-1.05803
C	1.20603	-3.69441	0.19699
H	1.55910	-3.99765	-0.79926
H	0.43339	-4.38942	0.54487
H	2.05469	-3.73126	0.88973
C	-3.83487	-0.13828	0.09387
O	-2.77888	0.62368	-0.02674
O	-4.96770	0.24757	0.01245
H	-1.43166	3.44070	-3.33930
H	-2.07019	-2.94795	-3.28360
H	-1.27067	-1.90285	2.87364
H	-3.02488	-2.20656	2.69165
H	-1.93344	-3.50348	3.25879
H	-1.51201	3.48861	3.21820
H	-0.27705	2.29332	2.76836
H	-0.69750	1.91250	-2.81830
H	-0.41320	-3.44188	-2.83287
C	4.99315	-0.43635	-0.01106
F	5.50350	0.27482	0.98565
F	5.39214	-1.68632	0.10874
F	5.44958	0.05237	-1.15813

I2_Ni_L6

Ni	-0.00071	0.68889	0.13313
H	-0.00601	2.86761	-1.61538
P	2.18568	0.52899	0.11291
N	2.32292	-1.20043	-0.02383
C	1.15848	-1.89338	-0.08104

N	1.18823	-3.21538	-0.19062
C	0.00543	-3.81147	-0.23913
N	-1.17900	-3.21868	-0.19001
C	-1.15290	-1.89659	-0.08047
N	0.00181	-1.18228	-0.02136
N	-2.31922	-1.20688	-0.02276
P	-2.18683	0.52304	0.11265
C	3.58930	-1.92356	-0.07825
H	3.65643	-2.64608	0.74536
H	4.41552	-1.20813	0.00687
H	3.68411	-2.46972	-1.02602
C	-3.58363	-1.93348	-0.07691
H	-3.67778	-2.47894	-1.02516
H	-4.41177	-1.22046	0.00972
H	-3.64803	-2.65702	0.74601
C	-0.00602	3.35406	-0.59834
O	-0.00263	2.50921	0.40239
O	-0.00907	4.55007	-0.48600
H	0.00693	-4.90359	-0.32905
C	3.09474	1.01440	1.59990
H	4.17357	0.82356	1.50482
H	2.93398	2.09523	1.73717
H	2.69453	0.48934	2.47688
C	-3.09688	1.00705	1.59950
H	-2.69559	0.48302	2.47661
H	-2.93816	2.08822	1.73649
H	-4.17535	0.81413	1.50458
C	3.12386	1.23017	-1.26867
H	2.69255	0.90810	-2.22555
H	3.04869	2.32667	-1.19858
H	4.18672	0.95127	-1.22273
C	-3.12763	1.22025	-1.26919
H	-4.18932	0.93697	-1.22322
H	-3.05700	2.31706	-1.19932
H	-2.69498	0.89977	-2.22600

I2_Ni_L7

Ni	-0.06056	-0.45108	0.01852
H	1.59733	-2.85064	0.02778
P	-2.21113	-0.26011	0.00958
N	-2.38861	1.43809	-0.04452
C	-1.22086	2.14374	-0.02432
N	-1.26291	3.46517	-0.04220
C	-0.08354	4.07296	-0.02088
N	1.10194	3.48381	0.01099
C	1.08696	2.15916	0.02973
N	-0.06122	1.43744	0.01406
N	2.25485	1.46171	0.06649
P	2.13204	-0.25840	0.02183
O	3.13674	-0.73380	1.20117
C	4.19949	-1.56113	0.70023
H	3.92462	-2.61626	0.84584
H	5.10038	-1.34276	1.28619
C	4.35432	-1.19992	-0.77432
H	5.07766	-0.38580	-0.93206
H	4.64269	-2.05872	-1.39163
O	3.05760	-0.75697	-1.20984
O	-3.09648	-0.84842	1.22123
C	-4.20494	-1.63910	0.74773
H	-5.12335	-1.04305	0.85548
H	-4.27662	-2.53470	1.37538
C	-3.88908	-1.96447	-0.70761
H	-3.27856	-2.87448	-0.80460
H	-4.78480	-2.04846	-1.33327
O	-3.12373	-0.84062	-1.18801
C	-3.66954	2.13808	-0.08081
H	-3.78110	2.77448	0.80573
H	-4.47425	1.39491	-0.10259
H	-3.73423	2.76374	-0.97947
C	3.54735	2.13975	0.05218
H	3.95606	2.17298	-0.96754
H	4.23858	1.60471	0.71535
H	3.42216	3.16297	0.41974
C	0.53022	-3.20696	-0.01114
O	-0.35928	-2.24385	-0.01063
O	0.26724	-4.37329	-0.04847
H	-0.09195	5.16838	-0.03210

I2_Ni_L8

Ni	-0.48959	0.09011	0.09913
H	-2.77748	0.35361	-1.57685
N	-0.08977	2.02453	0.10240
C	1.29505	2.23050	-0.40506
C	2.08947	0.97565	-0.24581
N	3.39927	0.88373	-0.33350
C	3.89375	-0.35127	-0.21574
N	3.18702	-1.46442	-0.00512
C	1.88483	-1.29596	0.08612
N	1.31955	-0.09454	-0.04312
C	0.89143	-2.36423	0.40605
N	-0.48687	-1.89718	0.08777
C	-3.17819	0.16688	-0.53980
O	-2.27785	0.25853	0.40603
O	-4.33737	-0.09406	-0.36406
H	4.98014	-0.46078	-0.29659
H	1.13305	-3.31300	-0.10162
H	1.25988	2.46367	-1.48273
H	0.96759	-2.56427	1.48839
H	1.78735	3.08884	0.08232
C	-0.83362	-2.29173	-1.29235
H	-0.84974	-3.39119	-1.37732
H	-1.82746	-1.90683	-1.54968
H	-0.09617	-1.88880	-2.00031
C	-1.04275	2.84065	-0.67034
H	-1.06671	2.49992	-1.71343
H	-2.04106	2.73764	-0.23254
H	-0.74165	3.90083	-0.63887
C	-1.45591	-2.48593	1.03445
H	-1.25165	-2.11870	2.04770
H	-2.47119	-2.18315	0.75660
H	-1.37645	-3.58534	1.01267
C	-0.16720	2.39302	1.53225
H	0.01110	3.47473	1.65196
H	-1.16416	2.14202	1.91389
H	0.58932	1.84173	2.10730

I2_Ni_L9

Ni	-0.00059	-0.68632	0.13481
H	-0.00157	-2.91312	-1.61311
P	-2.15167	-0.48248	0.11259
N	-2.40041	1.20051	-0.02293
C	-1.20523	1.93513	-0.08360
C	-1.21090	3.33249	-0.19859
C	0.00399	4.00780	-0.25487
C	1.21751	3.32997	-0.19941
C	1.20904	1.93263	-0.08454
C	0.00119	1.20786	-0.02378
N	2.40282	1.19568	-0.02488
P	2.15093	-0.48665	0.11295
C	-3.68562	1.84554	-0.05843
H	-3.81078	2.55182	0.78060
H	-4.48405	1.09578	0.01861
H	-3.83964	2.40192	-0.99938

C	3.68922	1.83835	-0.06020
H	3.84389	2.39521	-1.00074
H	4.48628	1.08704	0.01580
H	3.81601	2.54372	0.77937
C	-0.00349	-3.40796	-0.59580
O	-0.00278	-2.58239	0.39355
O	-0.00603	-4.61966	-0.52629
H	0.00508	5.09712	-0.34522
C	-3.05122	-1.04840	1.59228
H	-4.13812	-0.89891	1.50655
H	-2.84360	-2.12255	1.71419
H	-2.67025	-0.51669	2.47419
C	3.04880	-1.05177	1.59401
H	2.66761	-0.51868	2.47500
H	2.84035	-2.12562	1.71712
H	4.13587	-0.90317	1.50890
C	-3.08390	-1.26483	-1.24526
H	-2.65707	-0.95541	-2.20858
H	-2.98468	-2.35687	-1.14889
H	-4.15296	-1.00578	-1.21296
C	3.08251	-1.27268	-1.24318
H	4.15223	-1.01643	-1.21016
H	2.98031	-2.36436	-1.14580
H	2.65738	-0.96313	-2.20721
H	2.15427	3.88789	-0.24633
H	-2.14654	3.89234	-0.24496

I2_Ni_L10

Ni	0.57013	-0.06391	-0.19253
H	2.76864	-0.74193	1.48727
O	0.42821	1.86296	-0.17741
C	-0.87839	2.43375	-0.04227
C	-1.84126	1.29421	0.05730
C	-3.22477	1.37730	0.21457
C	-3.97248	0.20000	0.30037
C	-3.36466	-1.05637	0.23534
C	-1.98069	-1.13336	0.07791
C	-1.24353	0.04139	-0.01202
C	-1.15324	-2.37578	-0.00380
O	0.20862	-1.95624	-0.17149
C	3.23908	-0.20247	0.61124
O	2.46266	-0.08404	-0.41192
O	4.37901	0.20123	0.70478
H	-5.05629	0.26325	0.42373
H	-1.22017	-2.99600	0.91040
H	-0.88980	3.08129	0.85528
H	-1.42780	-3.01849	-0.86186
H	-1.07160	3.08106	-0.91916
H	-3.97481	-1.96149	0.30952
H	-3.72737	2.34734	0.27299
C	1.15442	-3.00257	-0.27663
H	0.86841	-3.68217	-1.09550
H	2.12493	-2.54611	-0.49651
H	1.20417	-3.56512	0.66982
C	1.48819	2.79826	-0.28872
H	1.54770	3.40373	0.62993
H	2.41512	2.23121	-0.43116
H	1.31125	3.45567	-1.15516

I2_Ni_L11

Ni	0.49184	-0.09035	0.11614
H	2.85059	-0.36296	-1.57557
N	0.09278	-2.02558	0.12236
C	-1.25454	-2.26682	-0.47851
C	-2.09264	-1.04144	-0.28483
C	-3.47816	-0.91458	-0.38191
C	-4.05533	0.35133	-0.23734
C	-3.27577	1.48533	0.01399
C	-1.89293	1.34442	0.12792
C	-1.32171	0.08700	-0.03058
C	-0.87711	2.38483	0.48488
N	0.47776	1.89413	0.08593
C	3.25730	-0.15750	-0.53827
O	2.38391	-0.25594	0.39819
O	4.43086	0.13340	-0.41727
H	-5.14005	0.45655	-0.32048
H	-1.06180	3.37956	0.03601
H	-1.10256	-2.45251	-1.55504
H	-0.86079	2.53092	1.57791
H	-1.69106	-3.19224	-0.05627
H	-3.75561	2.46210	0.12785
H	-4.11343	-1.78402	-0.57632
C	0.73552	2.25181	-1.31558
H	0.80328	3.35018	-1.42123
H	1.68273	1.80741	-1.64678
H	-0.07814	1.87760	-1.94992
C	1.10489	-2.83508	-0.55920
H	1.20113	-2.50817	-1.60296
H	2.07114	-2.70730	-0.05872
H	0.81782	-3.90215	-0.53770
C	1.50527	2.49706	0.94285
H	1.37890	2.14249	1.97353
H	2.50248	2.19723	0.60162
H	1.41881	3.59848	0.91748
C	0.07506	-2.36877	1.55162
H	-0.06438	-3.45829	1.67918
H	1.02619	-2.06937	2.01022
H	-0.74782	-1.84067	2.04964

4.4.3. I3

I3_Co_L1

Co	0.34795	-0.03756	-0.15154
H	0.84304	0.90067	2.65420
P	-1.38313	1.21159	-0.29204
N	-0.59645	2.80979	-0.07524
C	0.73909	2.82659	0.14565
N	1.40262	3.98135	0.28548
C	2.70761	3.85174	0.46910
N	3.41382	2.72953	0.46597
C	2.68662	1.61844	0.30758
N	1.34822	1.63099	0.21057
N	3.28992	0.40623	0.22118
P	2.26062	-0.97473	-0.25231
N	2.89597	-2.24723	0.66047
C	3.37852	-3.34073	-0.16487
H	2.58142	-4.09095	-0.33628
H	4.21294	-3.85130	0.34427
C	3.81631	-2.70946	-1.47407
H	4.86917	-2.36214	-1.42278
H	3.74725	-3.41974	-2.31399
N	2.90227	-1.60490	-1.68394
N	-2.71071	1.35996	0.73595
C	-3.98867	1.20519	0.06499
H	-4.73798	1.85333	0.55057
H	-4.35356	0.16410	0.12905
C	-3.75096	1.61473	-1.38135
H	-4.39813	1.04674	-2.07105
H	-3.96437	2.69135	-1.54157
N	-2.36093	1.30982	-1.65739
C	-1.35596	4.03810	-0.16782
H	-0.86415	4.82879	0.41040
H	-2.36024	3.86393	0.24142
H	-1.44785	4.37724	-1.21233
C	4.73074	0.30780	0.32866
H	5.23351	0.66718	-0.58313
H	4.99391	-0.74205	0.50587
H	5.09077	0.91028	1.17212
H	0.49976	0.23976	-1.60268
Si	-0.65356	-1.91404	-0.92294
H	-0.70033	-2.02737	-2.42362
H	0.09450	-3.13011	-0.44002
C	-4.07504	-2.47454	1.42079
C	-5.11156	-2.47837	0.48764
C	-2.75757	-2.30004	1.00446
H	-6.14474	-2.62089	0.81505
H	-1.95003	-2.27957	1.74460
C	-4.82385	-2.30197	-0.86418
C	-2.44604	-2.12905	-0.35471
H	-5.63096	-2.30682	-1.60218
C	-3.50336	-2.12624	-1.27578
H	-3.28673	-1.99768	-2.34260
H	-4.29407	-2.61026	2.48328
C	0.29760	-0.07907	2.78304
O	0.06695	-0.48461	3.90791
O	-0.01939	-0.66537	1.68984
C	-2.64159	1.13877	2.16078
H	-2.70638	0.07263	2.43711
H	-1.70149	1.53710	2.56632
H	-3.46695	1.68168	2.64676
C	-1.87677	1.43673	-3.00154
H	-0.82575	1.12022	-3.05415
H	-2.45790	0.79248	-3.68246
H	-1.94495	2.47768	-3.37229
C	2.42712	-2.57765	1.98961
H	1.48171	-3.14738	1.97812
H	3.19833	-3.17695	2.49695
H	2.26299	-1.67148	2.58380
C	3.02126	-0.83226	-2.88901
H	3.99783	-0.31495	-2.96420
H	2.91169	-1.48315	-3.77069
H	2.22477	-0.07563	-2.92884
H	3.27131	4.77986	0.62564

I3_Co_L2

Co	0.41430	-0.07034	-0.16195
H	0.98864	1.38581	2.43599
P	-1.41826	1.01030	-0.44454
O	-0.76058	2.68657	-0.31697
C	0.52617	2.82686	-0.14996
N	1.06263	4.03839	-0.07056
C	2.38207	4.04903	0.07377
N	3.19904	3.00196	0.11008
C	2.58994	1.82770	0.02173
N	1.25850	1.70676	-0.07033
O	3.28983	0.72238	0.01309
P	2.39704	-0.81730	-0.23857
N	3.20859	-1.81135	0.83214
C	4.49084	-2.20966	0.26717
H	4.82872	-3.14319	0.74207
H	5.26138	-1.43551	0.45563
C	4.24326	-2.38463	-1.22234
H	5.14934	-2.17287	-1.81393
H	3.92112	-3.41679	-1.45810
N	3.17788	-1.45492	-1.56534
N	-2.75639	1.17204	0.54083
C	-3.84772	1.81710	-0.17416
H	-3.76550	2.92008	-0.09751
H	-4.80679	1.51522	0.27493
C	-3.72956	1.35576	-1.61608
H	-4.29916	0.42096	-1.78102
H	-4.10520	2.11521	-2.32141
N	-2.31487	1.10059	-1.84656
H	0.49291	-0.03795	-1.64085
Si	-0.45524	-2.15902	-0.43745
H	-0.36748	-2.67401	-1.84912
H	0.27695	-3.12658	0.44305
C	-4.03979	-1.95178	1.68842
C	-5.01161	-1.99997	0.68927
C	-2.69090	-2.03708	1.35779
H	-6.07125	-1.93473	0.95050
H	-1.93340	-1.96863	2.14607
C	-4.62741	-2.14601	-0.64199
C	-2.28347	-2.17887	0.02180
H	-5.38502	-2.20020	-1.42908
C	-3.27517	-2.23843	-0.96705
H	-2.98514	-2.36787	-2.01642
H	-4.33606	-1.84587	2.73549
C	0.49493	0.42139	2.74588
O	0.31040	0.20410	3.92735
O	0.17139	-0.36359	1.78058
C	-2.65834	1.43682	1.95891
H	-3.63782	1.24794	2.42204
H	-1.93637	0.76088	2.42951
H	-2.36987	2.48366	2.16841
C	-1.93601	0.59471	-3.13865
H	-0.88077	0.29377	-3.14051
H	-2.54578	-0.28707	-3.40564
H	-2.07727	1.36038	-3.91872
C	3.13596	-1.62795	2.26747
H	3.58038	-2.50577	2.75744
H	3.68494	-0.72874	2.60351

H	2.09093	-1.55500	2.58946
C	2.74727	-1.39421	-2.93625
H	3.52385	-0.94850	-3.57954
H	2.52106	-2.40566	-3.31626
H	1.83637	-0.78838	-3.02472
H	2.85307	5.03498	0.16357

I3_Co_L3

Co	0.44918	0.12073	0.46213
H	1.46274	1.87651	3.78985
P	-1.38970	1.25104	0.15311
C	-0.71943	2.92877	-0.35930
C	0.67743	2.80227	-0.83323
N	1.20936	3.73122	-1.62571
C	2.47595	3.54103	-1.96421
N	3.25346	2.54256	-1.56116
C	2.66356	1.64823	-0.77446
N	1.36921	1.72312	-0.42571
C	3.42642	0.49146	-0.23771
P	2.25919	-0.95300	0.06974
N	3.15441	-1.99879	1.06646
C	3.55055	-3.21245	0.37471
H	2.83067	-4.02901	0.58488
H	4.54537	-3.54604	0.71583
C	3.54714	-2.86901	-1.10142
H	4.49808	-2.37023	-1.39135
H	3.43718	-3.76313	-1.73559
N	2.40893	-1.98562	-1.28376
N	-2.72894	1.56835	1.15169
C	-4.00128	1.39417	0.47252
H	-4.71127	2.18613	0.76820
H	-4.44891	0.42041	0.75280
C	-3.70742	1.42844	-1.01286
H	-4.45744	0.86409	-1.59173
H	-3.71486	2.47432	-1.39421
N	-2.40201	0.81857	-1.16881
H	0.04344	-0.42172	-0.84849
Si	-0.57385	-1.76717	1.15434
H	0.31857	-2.97692	1.03658
H	-0.99481	-1.69020	2.59563
C	-4.54623	-2.37847	-0.04144
C	-4.42398	-2.69054	-1.39260
C	-3.40497	-2.14211	0.72465
H	-5.31554	-2.87906	-1.99660
H	-3.51607	-1.90155	1.78834
C	-3.15589	-2.77408	-1.96918
C	-2.12508	-2.20007	0.16187
H	-3.05252	-3.03086	-3.02717
C	-2.02541	-2.53032	-1.19728
H	-1.03408	-2.58738	-1.66409
H	-5.53591	-2.32182	0.42064
C	0.90605	1.72226	2.83195
O	0.15654	2.60789	2.43017
O	1.14282	0.59901	2.28484
C	-2.74126	1.29865	2.56949
H	-3.15418	0.29453	2.78585
H	-1.72971	1.36887	2.98071
H	-3.36649	2.04204	3.08995
C	-1.88917	0.81408	-2.51025
H	-0.90460	0.32711	-2.54489
H	-2.56538	0.24135	-3.16451
H	-1.78953	1.83543	-2.93641
C	2.91136	-2.10763	2.48791
H	2.19639	-2.92040	2.71565
H	3.85433	-2.32159	3.01610
H	2.49623	-1.16780	2.87481
C	2.21365	-1.45669	-2.60797
H	3.07118	-0.84428	-2.95766
H	2.07685	-2.28122	-3.32395
H	1.30745	-0.83463	-2.63670
H	2.92682	4.27898	-2.63840
H	4.31604	0.26859	-0.84415
H	-1.33990	3.47435	-1.08458
H	3.76927	0.74804	0.78039
H	-0.69349	3.49794	0.58501

I3_Co_L5

Co	0.38950	0.53125	0.15384
H	-0.92422	-1.00262	2.65494
P	1.23664	-1.43653	0.11016
N	-0.28341	-2.40081	0.09793
C	-1.44008	-1.72412	-0.05013
N	-2.61256	-2.36698	-0.11861
C	-3.66187	-1.57250	-0.22695
N	-3.69235	-0.25713	-0.27999
C	-2.48436	0.32241	-0.20721
N	-1.34760	-0.38544	-0.11703
N	-2.36856	1.66525	-0.23153
P	-0.70871	2.33723	-0.22345
N	-0.84553	3.73563	0.69567
C	-0.56383	4.94379	-0.05931
H	0.49560	5.24366	0.06141
H	-1.18640	5.77262	0.31757
C	-0.87765	4.61852	-1.50953
H	-1.94244	4.82342	-1.74671
H	-0.26816	5.21866	-2.20462
N	-0.56694	3.21058	-1.66364
N	2.21191	-2.18149	1.25633
C	3.33294	-2.92076	0.69486
H	3.46893	-3.87905	1.22487
H	4.26061	-2.33282	0.82505
C	3.03451	-3.13876	-0.78370
H	3.95461	-3.06316	-1.38888
H	2.60162	-4.14249	-0.97059
N	2.10696	-2.09621	-1.17194
C	-0.26934	-3.84698	0.15249
H	-1.17859	-4.21405	0.64287
H	0.60326	-4.16738	0.73674
H	-0.21688	-4.29219	-0.85378
C	-3.55640	2.49240	-0.30135
H	-4.01632	2.45542	-1.30080
H	-3.27423	3.52414	-0.06121
H	-4.29841	2.15197	0.43203
H	0.76907	0.57899	-1.27936
Si	2.32834	1.68775	0.35290
H	2.32304	2.94353	-0.47883
H	2.55115	2.10886	1.77633
C	6.04415	-0.25396	0.46708
C	6.22741	-0.68585	-0.84418
C	4.89427	0.45685	0.81147
H	7.12913	-1.23878	-1.12046
H	4.76755	0.79976	1.84444
C	5.25883	-0.39788	-1.80650
C	3.90382	0.75101	-0.13574
H	5.40152	-0.72403	-2.84090
C	4.11376	0.30779	-1.45060
H	3.35899	0.52194	-2.21556
H	6.80373	-0.46448	1.22465
C	-0.94895	0.12541	2.71320
O	-1.85166	0.65724	3.33417
O	0.01583	0.72107	2.11224
C	2.37223	-1.65242	2.59342
H	3.37349	-1.20121	2.71039
H	1.62567	-0.87019	2.78519
H	2.25990	-2.44499	3.34998
C	1.63401	-2.08377	-2.52626

H	0.96396	-1.22677	-2.68621
H	2.48192	-1.98577	-3.22444
H	1.08247	-3.00774	-2.78824
C	-0.70746	3.79066	2.13638
H	0.33146	4.01421	2.43756
H	-1.36224	4.58399	2.53008
H	-1.00122	2.83565	2.59036
C	-0.81556	2.59909	-2.94055
H	-1.88466	2.64653	-3.22820
H	-0.22913	3.10129	-3.72552
H	-0.51174	1.54258	-2.91787
C	-4.99726	-2.30192	-0.36556
F	-5.09581	-3.29232	0.51215
F	-6.03091	-1.49454	-0.20037
F	-5.07672	-2.83054	-1.59143

I3_Co_L6

Co	0.16584	-0.32747	-0.37248
H	0.17708	-0.33738	3.55156
P	-0.92112	1.53846	-0.49484
N	0.40215	2.67467	-0.21890
C	1.64558	2.14767	-0.05208
N	2.68169	2.95319	0.19943
C	3.84441	2.32588	0.31782
N	4.07414	1.03020	0.18526
C	2.98900	0.29235	-0.06903
N	1.75871	0.81994	-0.16173
N	3.10401	-1.04238	-0.27691
P	1.59669	-1.93100	-0.44801
C	0.23106	4.11066	-0.11216
H	0.96300	4.63356	-0.74164
H	0.36809	4.45612	0.92385
H	-0.77683	4.38608	-0.44505
C	4.36623	-1.69804	0.00038
H	5.19565	-1.09917	-0.39320
H	4.38271	-2.68302	-0.48331
H	4.51129	-1.82588	1.08561
H	0.28725	-0.28502	-1.84646
Si	-1.55657	-1.70715	-0.87653
H	-1.80029	-1.91970	-2.34707
H	-1.34241	-3.09052	-0.30865
C	-4.68174	-0.64610	1.69849
C	-5.66306	-0.15367	0.83784
C	-3.47846	-1.12198	1.18598
H	-6.60684	0.22420	1.23981
H	-2.70095	-1.47456	1.87095
C	-5.43668	-0.15139	-0.53661
C	-3.22791	-1.13055	-0.19563
H	-6.20486	0.22333	-1.21839
C	-4.23327	-0.64172	-1.04281
H	-4.07693	-0.65662	-2.12798
H	-4.85531	-0.65256	2.77781
C	0.61442	-0.46043	2.52751
O	1.82104	-0.65634	2.41658
O	-0.25720	-0.37945	1.60108
H	4.71417	2.95478	0.54501
C	-2.13875	2.04738	0.75711
H	-3.09406	1.55437	0.52790
H	-1.79709	1.70128	1.74087
H	-2.28980	3.13602	0.75864
C	1.71326	-3.26958	0.78229
H	2.50050	-3.99247	0.52471
H	1.89831	-2.82654	1.76877
H	0.74449	-3.79000	0.79240
C	-1.66306	2.12570	-2.05253
H	-0.92510	2.03391	-2.86029
H	-2.51404	1.46509	-2.27860
H	-2.03205	3.15944	-1.98919

C	1.82743	-2.83553	-2.01545
H	2.72665	-3.46848	-2.00880
H	0.94779	-3.48266	-2.15922
H	1.87395	-2.12063	-2.84694

I3_Co_L7

Co	0.38371	-0.15931	-0.23166
H	-0.05988	1.15342	2.38335
P	-1.09774	1.31305	-0.44829
N	-0.16558	2.78980	-0.27431
C	1.16029	2.64184	-0.01379
N	1.96354	3.69943	0.11748
C	3.23764	3.40519	0.32779
N	3.78739	2.19840	0.35335
C	2.92398	1.19392	0.20499
N	1.60097	1.37830	0.09169
N	3.35930	-0.09587	0.13714
P	2.15260	-1.28905	-0.27446
O	2.56968	-2.50496	0.73896
C	2.88136	-3.69930	0.03618
H	1.96967	-4.30992	-0.06684
H	3.62432	-4.26000	0.61886
C	3.41439	-3.25124	-1.31939
H	4.50089	-3.06402	-1.29183
H	3.20206	-3.97553	-2.11644
O	2.73057	-2.03980	-1.61772
O	-2.34350	1.64719	0.56083
C	-3.59961	1.68528	-0.10364
H	-4.21305	2.46427	0.36941
H	-4.10143	0.71334	0.01276
C	-3.29513	2.00301	-1.56714
H	-3.97713	1.48130	-2.25198
H	-3.34261	3.08462	-1.77379
O	-1.97209	1.53860	-1.81535
C	-0.78048	4.09701	-0.39939
H	-0.06624	4.86219	-0.07772
H	-1.67394	4.14964	0.23820
H	-1.06421	4.29374	-1.44386
C	4.76989	-0.41441	0.25102
H	5.26028	-0.41862	-0.73452
H	4.87643	-1.40057	0.72053
H	5.26272	0.33341	0.88187
H	0.54067	0.04662	-1.69640
Si	-0.88797	-1.93155	-0.85604
H	-0.98773	-2.13371	-2.33845
H	-0.27883	-3.18202	-0.28439
C	-4.29445	-1.65388	1.56397
C	-5.33774	-1.63278	0.63804
C	-2.97369	-1.74164	1.13196
H	-6.37404	-1.57113	0.98079
H	-2.16318	-1.74822	1.86713
C	-5.05505	-1.69470	-0.72513
C	-2.66896	-1.80862	-0.23830
H	-5.86812	-1.68481	-1.45631
C	-3.73114	-1.77739	-1.15516
H	-3.51814	-1.83154	-2.22863
H	-4.50848	-1.60149	2.63418
C	-0.23631	0.05881	2.59461
O	-0.65654	-0.27645	3.68172
O	0.04481	-0.73058	1.61609
H	3.91578	4.25313	0.48109

I3_Co_L8

Co	0.27100	-0.33355	0.16059
H	2.35615	0.03132	3.53036

N	-0.31807	1.65306	0.35180
C	0.92584	2.45682	0.33643
C	1.98236	1.82595	-0.50928
N	3.06960	2.46229	-0.91302
C	3.99600	1.67542	-1.45904
N	3.96245	0.34723	-1.51574
C	2.84644	-0.19642	-1.05699
N	1.80348	0.52358	-0.66976
C	2.72215	-1.64780	-0.75194
N	1.32632	-2.04428	-0.42492
H	-0.63681	-0.51180	-1.00133
Si	-1.66577	-1.34353	0.76395
H	-1.71801	-2.78237	0.31384
H	-1.84803	-1.37090	2.25992
C	-5.33301	0.68805	0.43137
C	-5.60135	0.63657	-0.93374
C	-4.19184	0.06881	0.94065
H	-6.49573	1.11842	-1.33723
H	-4.00296	0.10244	2.01981
C	-4.72570	-0.03960	-1.78356
C	-3.28309	-0.59417	0.10461
H	-4.93539	-0.09249	-2.85519
C	-3.58400	-0.64300	-1.26562
H	-2.89594	-1.15639	-1.94816
H	-6.02062	1.20629	1.10506
C	2.24485	-0.09103	2.42135
O	3.25967	-0.06571	1.72410
O	1.03974	-0.24311	2.04716
H	4.88147	2.16815	-1.87518
H	3.14975	-2.28394	-1.54460
H	0.72957	3.50484	0.05093
H	3.35023	-1.76102	0.14770
H	1.34824	2.47427	1.35553
C	0.72188	-2.61030	-1.63980
H	-0.32084	-2.88595	-1.44773
H	0.74917	-1.86801	-2.44777
H	1.28051	-3.51040	-1.95577
C	-1.13735	2.06525	-0.79826
H	-0.57997	1.90291	-1.72973
H	-2.06249	1.47965	-0.83420
H	-1.39199	3.13726	-0.71196
C	1.36078	-3.07436	0.62346
H	0.34821	-3.46075	0.78739
H	2.01455	-3.91185	0.31916
H	1.73030	-2.63540	1.55663
C	-1.04694	1.94012	1.59553
H	-1.16032	3.03152	1.72904
H	-2.04790	1.49707	1.54030
H	-0.50137	1.50771	2.44234

I3_Co_L9

Co	0.16843	0.08296	-0.53056
H	-0.92525	-0.21100	3.26906
P	0.31304	2.20518	-0.48717
N	1.82436	2.51888	0.29528
C	2.60212	1.36415	0.50918
C	3.89396	1.42875	1.06062
C	4.60240	0.24224	1.24285
C	4.05870	-0.99038	0.88755
C	2.76385	-1.02866	0.33861
C	2.02250	0.14450	0.13903
N	2.15194	-2.22901	-0.04776
P	0.61432	-2.00502	-0.81449
C	2.35559	3.82102	0.56308
H	3.26158	4.03622	-0.03724
H	2.62696	3.94225	1.62814
H	1.60938	4.59226	0.32734
C	2.84943	-3.47391	0.05683
H	3.77861	-3.48684	-0.54742
H	2.21316	-4.29722	-0.29280
H	3.12720	-3.69728	1.10316
H	0.62101	0.19386	-1.93360
Si	-1.86780	0.03192	-1.55960
H	-2.23900	1.21464	-2.44406
H	-2.09449	-1.12588	-2.52017
C	-4.51577	-1.11288	1.49471
C	-5.58154	-0.22011	1.38049
C	-3.43712	-1.03053	0.61876
H	-6.42725	-0.27796	2.07262
H	-2.59388	-1.71702	0.75250
C	-5.55342	0.75315	0.38445
C	-3.38934	-0.06638	-0.40187
H	-6.38154	1.46296	0.28843
C	-4.46962	0.82323	-0.49193
H	-4.46438	1.59204	-1.27347
H	-4.51619	-1.87077	2.28358
C	-0.61007	-0.62816	2.27295
O	-0.39483	-1.83702	2.19370
O	-0.54148	0.25382	1.37056
H	5.60788	0.27970	1.67414
C	-0.91460	3.17617	0.45725
H	-1.86367	3.14696	-0.10094
H	-1.06248	2.67230	1.42118
H	-0.61771	4.22602	0.60217
C	-0.38513	-3.46211	-0.33044
H	0.06883	-4.40847	-0.66163
H	-0.52629	-3.43811	0.75660
H	-1.36091	-3.35679	-0.83054
C	0.42174	3.15962	-2.05027
H	1.26456	2.76440	-2.63445
H	-0.50611	3.00036	-2.62011
H	0.56045	4.23842	-1.87845
C	0.96921	-2.46348	-2.56436
H	1.32226	-3.50331	-2.65424
H	0.04965	-2.34218	-3.15726
H	1.73359	-1.77672	-2.95322
H	4.63813	-1.90366	1.04391
H	4.34622	2.38092	1.34962

I3_Co_L10

Co	-0.34923	0.39364	-0.08906
H	-1.87096	1.47663	3.43772
O	0.44454	-1.30598	0.75439
C	-0.49982	-2.35686	0.97958
C	-1.72940	-2.08773	0.17219
C	-2.78123	-2.98157	-0.03519
C	-3.92270	-2.54356	-0.71426
C	-4.02667	-1.22056	-1.15847
C	-2.96551	-0.34126	-0.94331
C	-1.80844	-0.78615	-0.31008
C	-2.96174	1.12000	-1.25547
O	-1.62616	1.61330	-1.08524
H	0.21994	0.00000	-1.39297
Si	1.61179	1.64541	-0.46928
H	1.63490	2.35253	-1.81343
H	2.05444	2.76590	0.46293
C	5.40217	-0.02006	0.39148
C	5.43245	-1.17969	-0.38055
C	4.30694	0.84044	0.31723
H	6.28831	-1.85954	-0.32625
H	4.29882	1.74795	0.93163
C	4.35600	-1.46617	-1.22178
C	3.20479	0.57198	-0.51083
H	4.36563	-2.37635	-1.82972
C	3.26346	-0.60507	-1.27777
H	2.41014	-0.86186	-1.91584

H	6.23969	0.21692	1.05568
C	-1.84586	1.47331	2.30968
O	-2.80185	1.98251	1.71917
O	-0.80499	0.93574	1.83918
H	-4.75037	-3.23845	-0.88571
H	-3.28967	1.35654	-2.28748
H	-0.01216	-3.32526	0.74931
H	-3.61463	1.65661	-0.54160
H	-0.75266	-2.36544	2.06021
H	-4.94259	-0.88599	-1.65919
H	-2.72812	-4.01349	0.33172
C	-1.58158	3.01631	-0.93931
H	-2.04937	3.30495	0.01549
H	-0.52943	3.32388	-0.95643
H	-2.11007	3.49029	-1.78597
C	1.38928	-1.21276	1.79722
H	2.15458	-0.48721	1.50449
H	0.89709	-0.87402	2.72411
H	1.86561	-2.19674	1.95362

I3_Co_L11

Co	0.34715	-0.30525	0.15620
H	2.27472	-0.63340	3.66997
N	-0.33468	1.59816	0.73261
C	0.86501	2.46826	0.85577
C	1.89320	2.06742	-0.15621
C	2.94431	2.84539	-0.64349
C	3.93913	2.23673	-1.41733
C	3.91875	0.85570	-1.64586
C	2.86171	0.09339	-1.14591
C	1.81628	0.71210	-0.46552
C	2.79445	-1.39792	-1.05733
N	1.38530	-1.84173	-0.83366
H	-0.36004	-0.09248	-1.11176
Si	-1.65633	-1.52465	0.58227
H	-1.77733	-2.86960	-0.12438
H	-2.03023	-1.88538	2.01219
C	-5.31151	0.61873	0.39195
C	-5.51806	0.77525	-0.97694
C	-4.22008	-0.11580	0.85630
H	-6.37262	1.34963	-1.34704
H	-4.08399	-0.23958	1.93742
C	-4.62077	0.18780	-1.87095
C	-3.28635	-0.69616	-0.01749
H	-4.77180	0.29935	-2.94922
C	-3.52837	-0.52920	-1.39284
H	-2.81985	-0.95966	-2.11084
H	-6.01101	1.06706	1.10489
C	2.17772	-0.89101	2.57581
O	3.11277	-1.50267	2.05406
O	1.08753	-0.50393	2.07024
H	4.76032	2.83995	-1.81669
H	3.21981	-1.93515	-1.92929
H	0.56042	3.53455	0.80820
H	3.35946	-1.69456	-0.15823
H	1.28145	2.29734	1.86270
H	4.74391	0.38229	-2.19058
H	3.00846	3.91546	-0.41329
C	0.76530	-2.05919	-2.14222
H	-0.29166	-2.32479	-2.02114
H	0.83891	-1.14323	-2.74175
H	1.28644	-2.88183	-2.67144
C	-1.19615	2.16583	-0.30858
H	-0.62568	2.26323	-1.24060
H	-2.06284	1.52015	-0.48648
H	-1.55054	3.16756	0.00582
C	1.39744	-3.11096	-0.10092
H	0.37326	-3.49333	-0.01195
H	2.00972	-3.85702	-0.64686
H	1.83168	-2.94830	0.89208
C	-1.05777	1.59040	2.00401
H	-1.23218	2.62788	2.35343
H	-2.03292	1.10629	1.87080
H	-0.47253	1.03167	2.74420

I3_Fe_L1

Fe	0.33351	-0.06384	-0.11971
H	0.81057	0.93612	2.71017
P	-1.31824	1.23002	-0.30077
N	-0.49947	2.83413	-0.07239
C	0.82515	2.79420	0.19201
N	1.52600	3.93004	0.35483
C	2.81756	3.75453	0.58183
N	3.48472	2.61115	0.59536
C	2.72387	1.51989	0.40577
N	1.38408	1.57016	0.27449
N	3.29453	0.29653	0.31478
P	2.19346	-1.02893	-0.26342
N	2.85264	-2.38761	0.55305
C	3.46413	-3.35072	-0.33575
H	2.73099	-4.13360	-0.62406
H	4.30915	-3.86062	0.16355
C	3.91647	-2.57572	-1.55876
H	4.93882	-2.15935	-1.41706
H	3.95531	-3.21873	-2.45607
N	2.93207	-1.53643	-1.72886
N	-2.70663	1.50884	0.67584
C	-3.95064	1.33269	-0.03904
H	-4.73850	1.96185	0.41462
H	-4.29987	0.28151	-0.00138
C	-3.65701	1.74977	-1.47122
H	-4.30172	1.20641	-2.18628
H	-3.84488	2.83588	-1.62390
N	-2.27468	1.41183	-1.70040
C	-1.19742	4.08821	-0.19577
H	-0.73789	4.84988	0.44739
H	-2.24107	3.93683	0.11355
H	-1.18382	4.46351	-1.23409
C	4.72014	0.15252	0.46825
H	5.27093	0.46387	-0.43667
H	4.93885	-0.90227	0.68211
H	5.08087	0.76724	1.30410
H	0.51576	0.28421	-1.61367
Si	-0.75963	-1.90724	-0.95023
H	-0.91285	-1.99734	-2.46006
H	-0.14400	-3.26147	-0.61738
C	-4.18324	-2.42939	1.44026
C	-5.24428	-2.40082	0.53388
C	-2.87081	-2.30032	0.99107
H	-6.27550	-2.49732	0.88677
H	-2.04662	-2.28593	1.71449
C	-4.97651	-2.24265	-0.82485
C	-2.57414	-2.14970	-0.37556
H	-5.79992	-2.21654	-1.54625
C	-3.65841	-2.12011	-1.26598
H	-3.46183	-2.00216	-2.33849
H	-4.38091	-2.54394	2.51059
C	0.30452	-0.06198	2.87453
O	0.14328	-0.44409	4.03045
O	-0.03525	-0.66981	1.81374
C	-2.69695	1.18697	2.08071
H	-2.83838	0.10973	2.28091
H	-1.74311	1.48761	2.53468
H	-3.50123	1.74713	2.58705
C	-1.75098	1.53595	-3.02465
H	-0.71037	1.18246	-3.04318

H	-2.33164	0.91997	-3.73477
H	-1.77344	2.58518	-3.38727
C	2.18994	-2.94863	1.70964
H	1.41803	-3.68601	1.41724
H	2.92952	-3.45367	2.35392
H	1.68924	-2.16963	2.29492
C	3.10700	-0.62821	-2.82332
H	4.05082	-0.04715	-2.74776
H	3.12486	-1.17837	-3.77945
H	2.26398	0.07749	-2.85106
H	3.40766	4.66371	0.76238

I3_Fe_L2

Fe	0.41869	-0.09868	-0.13020
H	1.04472	1.45029	2.44782
P	-1.36612	0.95344	-0.47236
O	-0.71809	2.68604	-0.42259
C	0.56153	2.80390	-0.23650
N	1.11627	4.01863	-0.22152
C	2.43329	4.02112	-0.07816
N	3.24130	2.97324	-0.00317
C	2.62055	1.79179	-0.02898
N	1.27946	1.67543	-0.07645
O	3.32328	0.69846	-0.02437
P	2.35105	-0.86213	-0.23284
N	3.26279	-1.81431	0.83409
C	4.55156	-2.13717	0.26152
H	4.97049	-3.03352	0.74897
H	5.27399	-1.30611	0.40740
C	4.28122	-2.36905	-1.21660
H	5.16813	-2.13983	-1.83518
H	4.01018	-3.42824	-1.40670
N	3.16951	-1.51030	-1.56180
N	-2.73872	1.23955	0.47491
C	-3.77995	1.90620	-0.27348
H	-3.63828	3.00837	-0.25637
H	-4.76392	1.68502	0.17447
C	-3.67275	1.36314	-1.68796
H	-4.29282	0.45109	-1.80432
H	-4.01397	2.10050	-2.43705
N	-2.27795	1.02880	-1.89104
H	0.52406	-0.03810	-1.66546
Si	-0.52447	-2.20022	-0.32003
H	-0.50110	-2.87500	-1.68233
H	0.05986	-3.24222	0.60896
C	-4.15291	-1.75961	1.73427
C	-5.12379	-1.83850	0.73449
C	-2.80777	-1.93132	1.42196
H	-6.18116	-1.69683	0.97829
H	-2.05057	-1.82249	2.20753
C	-4.73305	-2.10510	-0.57634
C	-2.38766	-2.19475	0.10646
H	-5.48548	-2.17720	-1.36882
C	-3.38297	-2.28769	-0.87715
H	-3.09187	-2.50230	-1.91297
H	-4.44778	-1.55055	2.76736
C	0.54807	0.50445	2.81567
O	0.40825	0.36052	4.02513
O	0.18730	-0.30797	1.90542
C	-2.62521	1.57224	1.87146
H	-3.59428	1.39889	2.36664
H	-1.88298	0.92742	2.35669
H	-2.33814	2.63109	2.02781
C	-1.94126	0.38127	-3.12636
H	-0.90309	0.02591	-3.09296
H	-2.59870	-0.49104	-3.30585
H	-2.04933	1.07301	-3.98073
C	3.20630	-1.57845	2.25686
H	3.64628	-2.43925	2.78466
H	3.76240	-0.66814	2.55655
H	2.16247	-1.47754	2.57992
C	2.65216	-1.60204	-2.89648
H	3.36815	-1.19754	-3.63445
H	2.43914	-2.65379	-3.16601
H	1.71375	-1.03666	-2.97201
H	2.91383	5.00759	-0.03043

I3_Fe_L4

Fe	0.29230	-0.07839	0.26578
H	1.45034	1.27694	3.83840
P	-1.40821	1.14279	0.05352
N	-0.62209	2.75372	-0.07681
C	0.73486	2.75668	-0.26698
C	1.46133	3.94069	-0.49041
C	2.83520	3.83809	-0.66636
C	3.46889	2.60703	-0.64510
C	2.68309	1.45604	-0.43468
N	1.34868	1.55423	-0.24005
N	3.21523	0.20153	-0.43942
P	2.05025	-1.13622	-0.17843
N	2.97205	-2.32870	0.65404
C	3.29889	-3.48315	-0.15069
H	2.54861	-4.28770	0.00233
H	4.28520	-3.89717	0.13447
C	3.28065	-3.01573	-1.59146
H	4.26784	-2.59030	-1.88728
H	3.07552	-3.84931	-2.28772
N	2.23346	-2.02938	-1.65659
N	-2.72569	1.48760	1.10472
C	-4.02394	1.36828	0.47423
H	-4.73346	2.10429	0.89774
H	-4.44964	0.36007	0.65259
C	-3.80841	1.59429	-1.01347
H	-4.54457	1.02608	-1.61251
H	-3.93198	2.66744	-1.28537
N	-2.47559	1.12877	-1.28878
C	-1.36057	3.98330	-0.12337
H	-0.95899	4.72564	0.58812
H	-2.39713	3.78175	0.17273
H	-1.35949	4.44110	-1.13136
C	4.64072	0.03361	-0.39779
H	5.12894	0.31711	-1.34954
H	4.86665	-1.01789	-0.18622
H	5.07990	0.62902	0.42177
H	-0.03442	-0.46892	-1.18725
Si	-0.82704	-2.00760	0.79958
H	-0.23043	-3.29887	0.25109
H	-1.00421	-2.34678	2.27266
C	-5.04879	-2.38866	0.59918
C	-5.32465	-2.23112	-0.75748
C	-3.72835	-2.35651	1.05165
H	-6.35707	-2.25578	-1.11990
H	-3.52837	-2.48730	2.12195
C	-4.26696	-2.04425	-1.64998
C	-2.64622	-2.16115	0.18028
H	-4.46926	-1.92063	-2.71929
C	-2.95670	-2.00417	-1.18198
H	-2.13845	-1.83145	-1.89072
H	-5.86717	-2.54088	1.31028
C	1.60407	0.77870	2.83990
O	2.76245	0.55354	2.48226
O	0.52058	0.51565	2.24443
C	-2.65404	1.06524	2.48386
H	-3.10464	0.06262	2.62132
H	-1.60473	1.01295	2.80709
H	-3.19620	1.77506	3.13338

C	-1.99585	1.24665	-2.62941
H	-0.97664	0.83781	-2.69496
H	-2.63870	0.67417	-3.32276
H	-1.97178	2.30048	-2.98211
C	2.71397	-2.60893	2.04573
H	1.90667	-3.35791	2.16784
H	3.62663	-3.00343	2.52530
H	2.43466	-1.68666	2.56533
C	2.11425	-1.33646	-2.90572
H	3.04986	-0.80272	-3.18312
H	1.87426	-2.04463	-3.71737
H	1.30266	-0.59732	-2.84432
H	3.42515	4.74439	-0.83201
H	4.54447	2.52761	-0.79327
H	0.96281	4.90833	-0.52070

I3_Fe_L5

Fe	0.36976	0.54027	0.21288
H	-1.15543	-0.82456	2.59088
P	1.19481	-1.39200	0.13061
N	-0.33416	-2.39501	0.11522
C	-1.48015	-1.70281	-0.00321
N	-2.65996	-2.34018	-0.11311
C	-3.70135	-1.53781	-0.21518
N	-3.72481	-0.22220	-0.25015
C	-2.51075	0.35266	-0.14170
N	-1.37585	-0.35811	-0.00795
N	-2.38882	1.69372	-0.18263
P	-0.67744	2.31874	-0.22177
N	-0.83173	3.78942	0.63488
C	-0.63820	4.95909	-0.19362
H	0.42148	5.28855	-0.15444
H	-1.25758	5.80120	0.16660
C	-1.01232	4.54863	-1.60606
H	-2.10029	4.69031	-1.79140
H	-0.47841	5.15387	-2.35998
N	-0.62903	3.16160	-1.71216
N	2.17826	-2.24966	1.23523
C	3.28586	-2.95627	0.62315
H	3.46936	-3.92120	1.13117
H	4.21213	-2.35392	0.71229
C	2.92608	-3.15902	-0.84115
H	3.82901	-3.12490	-1.47865
H	2.44670	-4.14840	-1.01014
N	2.03670	-2.07953	-1.18642
C	-0.33946	-3.83588	0.12229
H	-1.21455	-4.21558	0.66588
H	0.57155	-4.18353	0.62834
H	-0.36722	-4.25473	-0.89838
C	-3.55876	2.52645	-0.31386
H	-3.93320	2.55445	-1.35161
H	-3.29460	3.54299	0.00575
H	-4.37049	2.15454	0.32542
H	0.67965	0.52859	-1.30091
Si	2.37058	1.66957	0.38117
H	2.45867	2.96856	-0.40922
H	2.78725	2.09532	1.77473
C	6.10184	-0.32536	0.26684
C	6.20013	-0.76559	-1.05138
C	4.99417	0.42091	0.67302
H	7.06634	-1.34922	-1.37766
H	4.93566	0.76504	1.71245
C	5.18216	-0.44890	-1.95301
C	3.95105	0.74488	-0.20753
H	5.24822	-0.78649	-2.99271
C	4.07910	0.28719	-1.53013
H	3.27774	0.50756	-2.24492
H	6.89505	-0.56065	0.98339
C	-0.78525	0.16889	2.98724
O	-1.19724	0.54008	4.08078
O	0.04048	0.77635	2.23543
C	2.42470	-1.69384	2.54592
H	3.40895	-1.18912	2.58142
H	1.66029	-0.94527	2.79352
H	2.41171	-2.47989	3.31960
C	1.53818	-2.03780	-2.52581
H	0.88866	-1.15953	-2.65266
H	2.37255	-1.95323	-3.24503
H	0.95547	-2.94536	-2.78971
C	-0.37827	3.89794	2.00537
H	0.67275	4.24265	2.05341
H	-1.00415	4.62215	2.55330
H	-0.43420	2.92380	2.51102
C	-0.91112	2.48885	-2.94591
H	-1.99646	2.47184	-3.18135
H	-0.39185	2.98455	-3.78335
H	-0.55148	1.45108	-2.88944
C	-5.03622	-2.25258	-0.38256
F	-5.16043	-3.27296	0.46447
F	-6.07631	-1.44850	-0.20048
F	-5.13107	-2.75445	-1.62378

I3_Fe_L6

Fe	0.13074	0.04136	-0.60663
H	-0.56415	-0.31683	3.32612
P	0.04874	2.15290	-0.54446
N	1.63213	2.53482	0.19670
C	2.44613	1.46571	0.40718
N	3.66355	1.65931	0.93890
C	4.37734	0.55149	1.08000
N	4.03333	-0.67685	0.73658
C	2.80529	-0.79055	0.20294
N	1.97733	0.26013	0.04885
N	2.36227	-1.99096	-0.23696
P	0.66732	-2.01019	-0.80785
C	2.10935	3.85120	0.54308
H	3.02586	4.10831	-0.01195
H	2.34419	3.92417	1.61656
H	1.33619	4.59265	0.30415
C	3.11240	-3.18394	0.06717
H	4.18951	-2.98565	-0.00130
H	2.85403	-3.97483	-0.65078
H	2.88989	-3.54330	1.08725
H	0.56615	0.15900	-2.09430
Si	-1.92988	-0.15711	-1.56576
H	-2.39363	0.95282	-2.50899
H	-2.17589	-1.37993	-2.44848
C	-4.49473	-1.21954	1.60182
C	-5.59587	-0.37383	1.46185
C	-3.43645	-1.14233	0.70101
H	-6.42637	-0.42520	2.17290
H	-2.56358	-1.78841	0.84560
C	-5.62154	0.54312	0.41357
C	-3.44145	-0.23938	-0.37528
H	-6.47788	1.21548	0.29554
C	-4.55788	0.60214	-0.48854
H	-4.59828	1.32322	-1.31380
H	-4.45606	-1.93468	2.42936
C	-0.09772	-0.60108	2.34089
O	0.73248	-1.51578	2.34166
O	-0.52294	0.08686	1.37168
H	5.37280	0.67060	1.53007
C	-1.11040	3.05582	0.55118
H	-2.09659	3.02695	0.06056
H	-1.18367	2.49258	1.49111
H	-0.83579	4.10556	0.73611

C	-0.02514	-3.47391	0.06445
H	0.52772	-4.40335	-0.14028
H	-0.04324	-3.26155	1.14114
H	-1.05505	-3.59323	-0.30866
C	0.06108	3.24083	-2.02088
H	0.89021	2.93446	-2.67298
H	-0.88255	3.05912	-2.55849
H	0.13842	4.31241	-1.78004
C	0.87811	-2.74321	-2.48033
H	1.32586	-3.74932	-2.46474
H	-0.12447	-2.80531	-2.93213
H	1.48702	-2.06125	-3.08792

I3_Fe_L7

Fe	0.38828	-0.18826	-0.15780
H	-0.13964	1.21024	2.42285
P	-1.03698	1.28140	-0.44778
N	-0.09725	2.78305	-0.28159
C	1.21848	2.61104	-0.01157
N	2.04347	3.66374	0.08168
C	3.31434	3.35529	0.28049
N	3.84745	2.14350	0.31365
C	2.96337	1.14354	0.20431
N	1.63177	1.33508	0.13491
N	3.38902	-0.14400	0.12365
P	2.11569	-1.31284	-0.25814
O	2.64493	-2.55690	0.72441
C	2.82456	-3.74328	-0.01152
H	1.86163	-4.27690	-0.11004
H	3.53807	-4.38983	0.52078
C	3.34137	-3.29507	-1.37239
H	4.43968	-3.16318	-1.36088
H	3.08495	-4.00537	-2.17269
O	2.70873	-2.06244	-1.63159
O	-2.34533	1.76048	0.47512
C	-3.55582	1.73563	-0.24596
H	-4.22520	2.50888	0.16123
H	-4.04191	0.75244	-0.13387
C	-3.17886	2.01278	-1.69944
H	-3.84504	1.48766	-2.40083
H	-3.21076	3.09396	-1.92848
O	-1.86466	1.52935	-1.87191
C	-0.66783	4.09534	-0.46372
H	-0.03133	4.84753	0.01713
H	-1.66582	4.12067	-0.00371
H	-0.75984	4.34668	-1.53321
C	4.79465	-0.46666	0.16770
H	5.25023	-0.43721	-0.83632
H	4.91379	-1.47381	0.58896
H	5.32323	0.25212	0.80562
H	0.56089	0.03664	-1.67178
Si	-0.94374	-1.95721	-0.77659
H	-1.09007	-2.26207	-2.25259
H	-0.51785	-3.29424	-0.19307
C	-4.41967	-1.47825	1.54579
C	-5.45663	-1.49206	0.61091
C	-3.09824	-1.63176	1.13368
H	-6.49452	-1.37040	0.93578
H	-2.29204	-1.59549	1.87458
C	-5.15754	-1.65814	-0.74031
C	-2.77213	-1.81072	-0.22329
H	-5.96171	-1.66936	-1.48336
C	-3.83064	-1.81346	-1.14527
H	-3.60763	-1.94703	-2.21040
H	-4.63996	-1.33704	2.60778
C	-0.25293	0.12030	2.70538
O	-0.61523	-0.14847	3.84375
O	0.01905	-0.70219	1.77194
H	4.00708	4.19723	0.41167

I3_Fe_L8

Fe	0.32360	-0.33322	0.16474
H	2.29949	-0.35749	3.68952
N	-0.37696	1.65110	0.66161
C	0.84813	2.46366	0.71392
C	1.84353	1.98386	-0.29209
N	2.76992	2.74971	-0.84173
C	3.66872	2.10494	-1.58737
N	3.74678	0.77804	-1.72874
C	2.79019	0.08675	-1.13840
N	1.75652	0.65902	-0.49589
C	2.82346	-1.39319	-0.97255
N	1.48076	-1.94274	-0.70249
H	-0.46748	-0.25490	-1.16465
Si	-1.62537	-1.56193	0.62716
H	-1.72734	-2.92220	-0.06549
H	-2.03690	-1.93045	2.05168
C	-5.28637	0.56973	0.40695
C	-5.50141	0.69910	-0.96361
C	-4.19161	-0.15619	0.87811
H	-6.35862	1.26581	-1.33990
H	-4.04747	-0.25866	1.96080
C	-4.60911	0.09395	-1.85120
C	-3.26317	-0.75387	0.01062
H	-4.76731	0.18465	-2.93067
C	-3.51301	-0.61339	-1.36624
H	-2.80400	-1.05402	-2.07736
H	-5.98127	1.03276	1.11528
C	2.23077	-0.54030	2.57863
O	3.28226	-0.81171	1.98616
O	1.05997	-0.44092	2.12239
H	4.42426	2.70986	-2.10021
H	3.31957	-1.89680	-1.82233
H	0.63853	3.54457	0.59991
H	3.43395	-1.53669	-0.06325
H	1.30996	2.31778	1.70599
C	0.87103	-2.35383	-1.96466
H	-0.15774	-2.69087	-1.78839
H	0.84114	-1.50260	-2.65665
H	1.45630	-3.17877	-2.42291
C	-1.24180	2.17331	-0.39534
H	-0.70229	2.17227	-1.35145
H	-2.12973	1.54027	-0.50349
H	-1.55573	3.21042	-0.15520
C	1.58778	-3.09966	0.18419
H	0.58766	-3.52144	0.34692
H	2.23835	-3.88006	-0.26332
H	2.01104	-2.78418	1.14460
C	-1.07232	1.71688	1.94401
H	-1.24031	2.77142	2.24756
H	-2.04855	1.22517	1.84981
H	-0.47971	1.18983	2.70222

I3_Fe_L9

Fe	0.18104	0.05459	-0.52849
H	-1.03914	-0.10870	3.37464
P	0.28226	2.16362	-0.45626
N	1.82610	2.54646	0.29003
C	2.63113	1.40806	0.48771
C	3.94870	1.51191	0.97550
C	4.70553	0.34892	1.13029
C	4.17320	-0.89888	0.80080
C	2.85147	-0.97503	0.31792
C	2.04589	0.17058	0.15181
N	2.26769	-2.19785	-0.04761
P	0.67650	-2.00357	-0.77532

C	2.34835	3.85340	0.50838
H	3.22227	4.08178	-0.14077
H	2.68003	4.00232	1.55651
H	1.57728	4.61037	0.30236
C	3.04104	-3.39418	-0.04690
H	3.92270	-3.33578	-0.72333
H	2.42662	-4.24438	-0.37484
H	3.42500	-3.64092	0.96344
H	0.68446	0.18988	-1.99342
Si	-1.85068	-0.01855	-1.58845
H	-2.27603	1.12642	-2.53114
H	-2.15818	-1.19253	-2.53809
C	-4.60484	-1.04782	1.43912
C	-5.69706	-0.19757	1.25040
C	-3.50811	-0.98359	0.58332
H	-6.55695	-0.23761	1.92876
H	-2.64188	-1.62672	0.78024
C	-5.66842	0.71760	0.19852
C	-3.45805	-0.08423	-0.49834
H	-6.51342	1.39961	0.04295
C	-4.56432	0.76662	-0.65595
H	-4.55951	1.49116	-1.48034
H	-4.59567	-1.75457	2.27588
C	-0.74720	-0.62993	2.41092
O	-0.67106	-1.86631	2.45440
O	-0.56486	0.16209	1.45767
H	5.73123	0.41664	1.51237
C	-0.90026	3.16363	0.54998
H	-1.87274	3.13362	0.03085
H	-1.01117	2.64798	1.51400
H	-0.60068	4.21493	0.69723
C	-0.22389	-3.49304	-0.15621
H	0.31215	-4.44121	-0.33027
H	-0.42859	-3.33051	0.91219
H	-1.17935	-3.52724	-0.70623
C	0.33022	3.19953	-1.98783
H	1.17017	2.84608	-2.60318
H	-0.60501	3.01741	-2.54068
H	0.43379	4.27991	-1.78926
C	0.99488	-2.60018	-2.50400
H	1.33012	-3.65114	-2.54699
H	0.05765	-2.49227	-3.07289
H	1.75116	-1.94073	-2.95299
H	4.78720	-1.79640	0.92651
H	4.38907	2.48073	1.23322

I3_Fe_L10

Fe	0.32651	-0.41142	-0.13686
H	1.81413	-1.36366	3.54617
O	-0.52484	1.42388	0.71882
C	0.46392	2.41110	0.95224
C	1.68452	2.12075	0.12915
C	2.71630	3.03573	-0.07583
C	3.89106	2.62507	-0.72310
C	4.02995	1.28396	-1.11602
C	2.98756	0.38543	-0.90150
C	1.76592	0.79425	-0.33165
C	3.09112	-1.09246	-1.13706
O	1.78866	-1.65750	-1.14968
H	-0.20765	0.03489	-1.54819
Si	-1.62253	-1.76369	-0.48880
H	-1.67907	-2.41975	-1.87708
H	-2.25352	-2.93947	0.30602
C	-5.37958	0.02139	0.43388
C	-5.40023	1.17717	-0.34811
C	-4.32101	-0.88143	0.32446
H	-6.22704	1.89218	-0.26809
H	-4.32448	-1.78441	0.94774
C	-4.33924	1.40959	-1.22964
C	-3.23224	-0.67250	-0.54470
H	-4.33085	2.31893	-1.84220
C	-3.28287	0.50705	-1.31851
H	-2.42877	0.72820	-1.97008
H	-6.19914	-0.17869	1.13536
C	1.80645	-1.45644	2.41300
O	2.72492	-2.12915	1.91516
O	0.85245	-0.85623	1.87032
H	4.70364	3.33929	-0.89895
H	3.60525	-1.34768	-2.09120
H	0.02871	3.41698	0.75666
H	3.65722	-1.55942	-0.30341
H	0.73206	2.38450	2.03261
H	4.97012	0.94883	-1.57744
H	2.62406	4.07399	0.27568
C	1.79499	-3.04770	-0.98251
H	2.16376	-3.30525	0.02684
H	0.76058	-3.39659	-1.10280
H	2.43631	-3.52499	-1.75391
C	-1.47124	1.35235	1.74485
H	-2.26374	0.66367	1.42841
H	-1.00450	0.96750	2.67100
H	-1.91282	2.35172	1.93765

I3_Fe_L11

Fe	0.33821	-0.35991	0.11448
H	2.96642	0.07875	1.72172
N	-0.31748	1.64483	0.71759
C	0.89794	2.48593	0.71821
C	1.86424	2.03152	-0.33703
C	2.85983	2.80184	-0.93161
C	3.84685	2.18078	-1.71723
C	3.86114	0.77981	-1.83137
C	2.85701	0.02679	-1.22803
C	1.78948	0.63837	-0.54029
C	2.86452	-1.46119	-1.02983
N	1.49259	-1.97988	-0.84715
H	-0.38975	-0.13443	-1.26805
Si	-1.68419	-1.63647	0.46641
H	-1.82724	-2.87927	-0.44033
H	-2.21806	-2.26343	1.77264
C	-5.29000	0.62517	0.48499
C	-5.53424	0.87218	-0.86712
C	-4.21772	-0.17941	0.86986
H	-6.37325	1.50477	-1.17724
H	-4.04953	-0.36450	1.93887
C	-4.68128	0.30289	-1.81769
C	-3.33038	-0.75492	-0.06010
H	-4.85179	0.49086	-2.88424
C	-3.60795	-0.48736	-1.41669
H	-2.92865	-0.89362	-2.17596
H	-5.94564	1.06324	1.24711
C	2.31479	-0.47958	2.46318
O	2.80884	-0.73554	3.57178
O	1.16149	-0.76217	2.04085
H	4.62513	2.78152	-2.20133
H	3.38553	-2.02645	-1.83730
H	0.62075	3.56330	0.64806
H	3.41215	-1.67865	-0.09388
H	1.37729	2.35011	1.70530
H	4.68009	0.28786	-2.37571
H	2.89618	3.88934	-0.77394
C	0.91203	-2.25588	-2.15079
H	-0.14486	-2.52962	-2.03931
H	0.97014	-1.35224	-2.77093
H	1.46347	-3.08682	-2.65125
C	-1.25379	2.17696	-0.26147

H	-0.75933	2.23459	-1.23955
H	-2.12533	1.51944	-0.35557
H	-1.59154	3.19444	0.04368
C	1.50493	-3.18760	-0.03982
H	0.49161	-3.61107	-0.00634
H	2.19910	-3.95000	-0.46459
H	1.80673	-2.93967	0.98701
C	-0.92739	1.63110	2.03663
H	-1.10806	2.66698	2.40714
H	-1.89063	1.10556	1.98624
H	-0.27414	1.08975	2.73459

I3_Ni_L1

Ni	0.35626	-0.03392	-0.21099
H	0.75340	0.94301	2.59815
P	-1.43026	1.23492	-0.32908
N	-0.64421	2.80692	-0.15449
C	0.69732	2.85802	0.06222
N	1.32411	4.01977	0.23863
C	2.63315	3.92576	0.42719
N	3.36420	2.81780	0.41665
C	2.67563	1.69810	0.21704
N	1.34104	1.68090	0.08661
N	3.30445	0.49265	0.11975
P	2.35545	-0.92328	-0.28311
N	2.84804	-2.15501	0.70992
C	3.63946	-3.17495	0.03315
H	3.34067	-4.17637	0.37864
H	4.71079	-3.04485	0.27865
C	3.38583	-3.00097	-1.45726
H	4.27198	-3.26684	-2.05395
H	2.55005	-3.64039	-1.80056
N	3.06487	-1.59086	-1.65051
N	-2.70445	1.32403	0.73804
C	-4.00442	1.12038	0.11249
H	-4.74036	1.78077	0.59808
H	-4.34879	0.08052	0.24210
C	-3.82616	1.47139	-1.35912
H	-4.45558	0.83715	-2.00364
H	-4.09763	2.52424	-1.56434
N	-2.42203	1.23087	-1.66848
H	0.38313	0.08315	-1.66204
Si	-0.66473	-1.99442	-0.92361
H	-0.71755	-2.08448	-2.41227
H	0.26050	-3.02300	-0.36729
C	-3.94931	-2.47843	1.57831
C	-5.00616	-2.54795	0.67043
C	-2.64998	-2.27129	1.12582
H	-6.02287	-2.72288	1.02972
H	-1.82394	-2.21749	1.84142
C	-4.76681	-2.40232	-0.69458
C	-2.39295	-2.12802	-0.24839
H	-5.59253	-2.46521	-1.40718
C	-3.46850	-2.18725	-1.14912
H	-3.28630	-2.08746	-2.22479
H	-4.13708	-2.59748	2.64779
C	0.27334	-0.07132	2.69617
O	-0.00737	-0.50938	3.78652
O	0.07415	-0.68618	1.57482
C	-2.58846	1.26240	2.17652
H	-2.67132	0.23543	2.56865
H	-1.62604	1.67988	2.50364
H	-3.38295	1.87627	2.62630
C	-1.97478	1.36471	-3.02976
H	-0.91504	1.08419	-3.11514
H	-2.55509	0.69866	-3.68744
H	-2.08537	2.39925	-3.40208
C	2.79023	-2.14915	2.15653
H	1.98245	-2.78720	2.54418
H	3.75244	-2.50450	2.55739
H	2.62772	-1.13387	2.53990
C	2.73419	-1.14215	-2.98207
H	3.56711	-1.36549	-3.66405
H	1.82404	-1.62968	-3.37863
H	2.58496	-0.05339	-2.99175
H	3.17096	4.86434	0.60251
C	4.74122	0.35841	0.31418
H	4.96270	-0.09032	1.29352
H	5.15984	-0.27714	-0.47736
H	5.20439	1.34926	0.26329
C	-1.45560	4.01508	-0.16631
H	-2.13704	3.99431	-1.02807
H	-2.04897	4.09982	0.75527
H	-0.79935	4.88728	-0.25184

I3_Ni_L2

Ni	0.39732	-0.05373	-0.20302
H	0.75872	1.20604	2.53157
P	-1.47597	1.09606	-0.42241
O	-0.81198	2.70370	-0.14161
C	0.47843	2.85951	0.05348
N	0.98294	4.05691	0.28145
C	2.29980	4.07737	0.46935
N	3.12955	3.03593	0.43662
C	2.54449	1.87983	0.20100
N	1.22792	1.75311	0.01238
O	3.25496	0.77059	0.13990
P	2.46980	-0.74091	-0.26179
N	3.17628	-1.83427	0.75808
C	4.44681	-2.28632	0.17765
H	4.69222	-3.27890	0.58049
H	5.26387	-1.59353	0.45354
C	4.23456	-2.31713	-1.32704
H	5.16824	-2.11345	-1.87321
H	3.84788	-3.29488	-1.66561
N	3.24563	-1.27789	-1.61422
N	-2.80679	1.09040	0.56014
C	-3.94277	1.70550	-0.13235
H	-3.95038	2.79762	0.04501
H	-4.87717	1.28787	0.27042
C	-3.76840	1.38673	-1.60757
H	-4.27148	0.44053	-1.87821
H	-4.17162	2.18596	-2.24813
N	-2.32977	1.24370	-1.82746
H	0.41007	-0.08119	-1.65872
Si	-0.42943	-2.16538	-0.70425
H	-0.33922	-2.42312	-2.17000
H	0.50078	-3.03104	0.06657
C	-3.83239	-2.21056	1.65263
C	-4.85817	-2.17754	0.70845
C	-2.50458	-2.20415	1.24137
H	-5.90054	-2.19124	1.03567
H	-1.70405	-2.20810	1.98741
C	-4.55885	-2.14323	-0.65245
C	-2.18854	-2.17288	-0.12717
H	-5.36352	-2.13483	-1.39179
C	-3.23048	-2.14098	-1.06759
H	-3.00208	-2.13577	-2.13929
H	-4.06949	-2.24873	2.71818
C	0.36953	0.16649	2.71192
O	0.15374	-0.21980	3.83414
O	0.18319	-0.54719	1.64218
C	-2.74631	1.26130	1.99991
H	-3.72847	1.00843	2.42172
H	-2.01790	0.57539	2.44487
H	-2.50093	2.29954	2.28391

C	-1.86908	0.96686	-3.16491
H	-0.78912	0.76866	-3.17294
H	-2.38722	0.08577	-3.58061
H	-2.05697	1.82417	-3.82897
C	3.08158	-1.77105	2.20961
H	3.56159	-2.66730	2.62296
H	3.59304	-0.88418	2.62206
H	2.03418	-1.77420	2.52903
C	2.93699	-0.96105	-2.98658
H	3.78794	-0.46862	-3.48196
H	2.69016	-1.87736	-3.54738
H	2.07488	-0.28226	-3.04321
H	2.75071	5.05518	0.66975

I3_Ni_L3

Ni	0.38721	0.03799	0.35049
H	1.76859	1.04617	3.79694
P	-1.33878	1.41287	0.22651
C	-0.41940	3.02736	0.33598
C	0.95697	2.91527	-0.22214
N	1.60089	4.00534	-0.60760
C	2.85738	3.81997	-0.99969
N	3.50975	2.66601	-0.97304
C	2.80667	1.60993	-0.58735
N	1.50259	1.68742	-0.26729
C	3.48352	0.28940	-0.47534
P	2.26793	-1.06944	-0.11450
N	3.04655	-2.36804	0.62359
C	3.21501	-3.49881	-0.28831
H	2.42167	-4.24985	-0.11157
H	4.18452	-3.98447	-0.09548
C	3.12567	-2.94054	-1.68961
H	4.10603	-2.53481	-2.01946
H	2.82149	-3.70399	-2.42221
N	2.11860	-1.89488	-1.60881
N	-2.65509	1.54374	1.25704
C	-3.92195	1.49198	0.52888
H	-4.65289	2.15344	1.01939
H	-4.32917	0.46381	0.53837
C	-3.61109	1.94351	-0.88558
H	-4.32762	1.53451	-1.61495
H	-3.63756	3.04927	-0.96731
N	-2.27747	1.42921	-1.16885
H	-0.17774	-0.72512	-0.77284
Si	-0.77999	-1.92787	0.60471
H	0.01037	-3.06425	0.05727
H	-0.76983	-1.94341	2.09721
C	-4.91255	-2.23999	0.42988
C	-5.19464	-2.04249	-0.91942
C	-3.59426	-2.18273	0.87839
H	-6.22687	-2.09011	-1.27401
H	-3.38293	-2.34770	1.94046
C	-4.15902	-1.79444	-1.82087
C	-2.54100	-1.93332	-0.01236
H	-4.37844	-1.65154	-2.88169
C	-2.84593	-1.73946	-1.36780
H	-2.04127	-1.54855	-2.08747
H	-5.72053	-2.44609	1.13517
C	1.71116	0.64452	2.76021
O	2.73749	0.24978	2.20966
O	0.54307	0.64894	2.25638
C	-2.65752	1.08448	2.63074
H	-3.03640	0.04945	2.70571
H	-1.64290	1.11429	3.04630
H	-3.30779	1.73335	3.23561
C	-1.71283	1.67695	-2.46951
H	-0.74634	1.16154	-2.56972
H	-2.38321	1.27895	-3.24666

H	-1.55620	2.75393	-2.67457
C	2.95711	-2.73791	2.02283
H	2.08300	-3.38760	2.21204
H	3.86054	-3.30109	2.29935
H	2.90555	-1.85176	2.65860
C	1.86159	-1.17265	-2.82874
H	2.76683	-0.68438	-3.24239
H	1.47957	-1.86172	-3.59675
H	1.09720	-0.39812	-2.67079
H	3.40333	4.69761	-1.36287
H	4.06226	0.07596	-1.38810
H	-0.97683	3.86378	-0.11192
H	4.20159	0.34142	0.35664
H	-0.33188	3.23505	1.41715

I3_Ni_L5

Ni	-0.39637	0.46939	-0.26984
H	0.79010	0.35505	-3.89746
P	-1.17090	-1.57941	-0.03856
N	0.37859	-2.42004	-0.10593
C	1.52280	-1.69654	-0.00032
N	2.70401	-2.30726	0.03225
C	3.74091	-1.48506	0.11260
N	3.73103	-0.17123	0.17067
C	2.51205	0.38126	0.14928
N	1.39439	-0.36367	0.07466
N	2.35097	1.71513	0.22689
P	0.69624	2.33758	0.16512
N	0.66917	3.85744	-0.52610
C	0.38243	4.91539	0.44382
H	-0.68294	5.20258	0.36862
H	0.98325	5.80570	0.20011
C	0.70677	4.36835	1.81621
H	1.76025	4.56892	2.09531
H	0.07334	4.81917	2.59573
N	0.45210	2.93868	1.73057
N	-2.19649	-2.41504	-1.04680
C	-3.31907	-3.03110	-0.34303
H	-3.52595	-4.01869	-0.78542
H	-4.22506	-2.41143	-0.46565
C	-2.91555	-3.14351	1.11967
H	-3.78164	-3.01015	1.78777
H	-2.46971	-4.13070	1.34765
N	-1.95039	-2.07936	1.35103
C	0.44271	-3.87475	-0.09263
H	1.24222	-4.21924	-0.75875
H	-0.51301	-4.27273	-0.45205
H	0.64739	-4.25506	0.91919
C	3.47930	2.62887	0.16706
H	4.39721	2.07982	0.40221
H	3.35032	3.43676	0.89870
H	3.56194	3.06313	-0.83974
H	-0.79107	0.61615	1.11780
Si	-2.33894	1.64063	-0.58608
H	-2.21739	2.90931	0.19176
H	-2.37969	1.90831	-2.04921
C	-5.97306	-0.35988	-0.59910
C	-6.17734	-0.65916	0.74585
C	-4.81090	0.29991	-0.99440
H	-7.09152	-1.16898	1.05879
H	-4.66579	0.54350	-2.05228
C	-5.22161	-0.29717	1.69600
C	-3.84306	0.67663	-0.05232
H	-5.38817	-0.52027	2.75284
C	-4.06436	0.36261	1.29777
H	-3.32535	0.65282	2.05290
H	-6.72585	-0.63201	-1.34218
C	0.70453	0.69449	-2.84109

O	1.47976	1.53499	-2.40315
O	-0.24429	0.12940	-2.19666
C	-2.34898	-2.19313	-2.46929
H	-3.22748	-1.55769	-2.67678
H	-1.46020	-1.70134	-2.87759
H	-2.49321	-3.15741	-2.97909
C	-1.38586	-1.93640	2.66685
H	-0.69827	-1.07858	2.69912
H	-2.18412	-1.75531	3.40368
H	-0.82404	-2.83526	2.98061
C	0.34331	4.21693	-1.89397
H	-0.72318	4.48964	-1.98088
H	0.94198	5.09258	-2.18525
H	0.57918	3.40335	-2.58102
C	0.82061	2.15873	2.88743
H	1.90750	2.19650	3.09186
H	0.29180	2.53844	3.77341
H	0.53582	1.10519	2.75619
C	5.10022	-2.18739	0.16067
F	5.23363	-2.98502	-0.89124
F	6.09907	-1.32717	0.17055
F	5.16755	-2.92989	1.26256

I3_Ni_L7

Ni	0.38184	-0.15354	-0.24252
H	-0.06001	1.03170	2.44792
P	-1.11318	1.39370	-0.42381
N	-0.16043	2.82044	-0.22455
C	1.17839	2.67048	0.00800
N	1.97621	3.72208	0.14328
C	3.25656	3.43050	0.33615
N	3.80248	2.21915	0.34447
C	2.94175	1.22239	0.19396
N	1.62139	1.40904	0.08387
N	3.37465	-0.07541	0.12722
P	2.21543	-1.28391	-0.26506
O	2.55281	-2.47496	0.77256
C	2.89828	-3.69304	0.10084
H	2.00081	-4.32585	0.03136
H	3.65431	-4.20874	0.70548
C	3.42123	-3.27851	-1.27046
H	4.50443	-3.08390	-1.26315
H	3.19661	-4.01342	-2.05222
O	2.73101	-2.06092	-1.59353
O	-2.31242	1.61512	0.63641
C	-3.59974	1.68825	0.00940
H	-4.18109	2.45797	0.53232
H	-4.10051	0.71684	0.12108
C	-3.34282	2.05048	-1.45401
H	-4.03000	1.53612	-2.13699
H	-3.40102	3.13386	-1.63501
O	-2.00912	1.60719	-1.75466
C	-0.78124	4.13783	-0.30876
H	-0.05310	4.88950	0.01237
H	-1.65435	4.18000	0.35588
H	-1.08461	4.35483	-1.34223
C	4.79281	-0.39925	0.24738
H	5.27244	-0.44261	-0.74078
H	4.90060	-1.36200	0.76187
H	5.28387	0.37632	0.84419
H	0.47259	-0.02470	-1.69651
Si	-0.89102	-1.99294	-0.86003
H	-0.85381	-2.12253	-2.34143
H	-0.13510	-3.08845	-0.19609
C	-4.31359	-1.76634	1.47720
C	-5.31825	-1.66721	0.51414
C	-2.97840	-1.82671	1.09214
H	-6.36591	-1.63390	0.82218
H	-2.19897	-1.90610	1.85506
C	-4.99187	-1.61834	-0.84062
C	-2.63524	-1.78147	-0.27065
H	-5.77975	-1.55170	-1.59445
C	-3.65654	-1.66802	-1.23073
H	-3.40565	-1.64054	-2.29656
H	-4.57047	-1.80436	2.53762
C	-0.34320	-0.05277	2.53855
O	-0.91188	-0.46352	3.51580
O	0.00377	-0.79143	1.51935
H	3.93567	4.27618	0.48995

I3_Ni_L8

Ni	0.25767	-0.28431	0.17612
H	2.30002	-0.03146	3.47048
N	-0.28568	1.68394	0.30302
C	0.98937	2.46567	0.29059
C	2.05223	1.80512	-0.52610
N	3.18602	2.38830	-0.85901
C	4.09254	1.56554	-1.39468
N	3.98623	0.24209	-1.48942
C	2.82563	-0.24821	-1.09574
N	1.81406	0.52146	-0.73537
C	2.60273	-1.70220	-0.86636
N	1.20632	-2.03106	-0.40560
H	-0.51218	-0.38160	-1.03927
Si	-1.70777	-1.18988	1.00820
H	-1.56574	-2.64754	0.74197
H	-1.68046	-0.86294	2.45524
C	-5.28276	0.78451	0.25633
C	-5.49972	0.52937	-1.09560
C	-4.15750	0.26201	0.88849
H	-6.38181	0.93900	-1.59278
H	-4.00653	0.45513	1.95616
C	-4.59839	-0.25611	-1.81507
C	-3.23046	-0.51615	0.17812
H	-4.77717	-0.46605	-2.87159
C	-3.47556	-0.77357	-1.18129
H	-2.77527	-1.38943	-1.75650
H	-5.99742	1.38577	0.82220
C	2.17350	-0.16892	2.37088
O	3.15154	-0.27056	1.64687
O	0.93788	-0.20317	2.01015
H	5.01975	2.01912	-1.75960
H	2.86469	-2.30443	-1.74994
H	0.79725	3.50425	-0.02422
H	3.31616	-1.96320	-0.07011
H	1.38589	2.51071	1.31812
C	0.48924	-2.64682	-1.54374
H	-0.53658	-2.89821	-1.25391
H	0.47036	-1.95316	-2.39346
H	1.00413	-3.57283	-1.84692
C	-1.08357	2.08746	-0.87594
H	-0.50780	1.91679	-1.79375
H	-2.01613	1.51289	-0.91635
H	-1.32671	3.15952	-0.79946
C	1.31613	-3.02835	0.67959
H	0.32000	-3.36619	0.98097
H	1.88328	-3.90091	0.31598
H	1.83421	-2.59011	1.53708
C	-1.02199	2.04936	1.52907
H	-1.07870	3.14747	1.60711
H	-2.04525	1.66373	1.47031
H	-0.50672	1.63712	2.40336

I3_Ni_L9

Ni	-0.24511	0.07699	0.58984
H	1.45089	-0.03777	-3.03957
P	-0.59084	2.22539	0.69735
N	-1.93199	2.47122	-0.31805
C	-2.59252	1.27736	-0.63991
C	-3.80448	1.28406	-1.34447
C	-4.42061	0.07275	-1.63691
C	-3.86040	-1.13506	-1.24127
C	-2.64484	-1.13094	-0.53883
C	-1.99483	0.07509	-0.22076
N	-2.04162	-2.31741	-0.12366
P	-0.62433	-2.07854	0.79631
C	-2.51220	3.75406	-0.61031
H	-3.49062	3.89012	-0.11446
H	-2.65586	3.88911	-1.69492
H	-1.84515	4.55565	-0.26745
C	-2.67162	-3.59070	-0.34455
H	-3.66576	-3.64585	0.13466
H	-2.05510	-4.39625	0.07177
H	-2.79639	-3.79474	-1.42113
H	0.92727	0.40183	1.73435
Si	2.30648	-0.25501	1.87776
H	2.80841	0.56068	3.02465
H	2.12155	-1.66386	2.31678
C	4.46222	-1.03264	-1.57241
C	5.11655	0.17370	-1.80694
C	3.63431	-1.16928	-0.46297
H	5.75937	0.28430	-2.68384
H	3.09017	-2.10613	-0.31785
C	4.95401	1.24066	-0.92478
C	3.45237	-0.10396	0.42640
H	5.47147	2.18585	-1.10578
C	4.13164	1.09847	0.18716
H	4.01611	1.93969	0.87951
H	4.57931	-1.86652	-2.26783
C	1.07131	-0.35031	-2.02941
O	0.81490	-1.54585	-1.85586
O	0.96900	0.58871	-1.19146
H	-5.36453	0.07060	-2.18804
C	0.74095	3.30781	0.09714
H	1.58136	3.23870	0.80573
H	1.07240	2.92061	-0.87546
H	0.42772	4.35987	0.03043
C	0.48301	-3.45878	0.35729
H	0.05519	-4.42570	0.65978
H	0.67102	-3.41208	-0.72246
H	1.43428	-3.32968	0.89342
C	-1.03306	2.98278	2.30584
H	-1.91062	2.46459	2.71624
H	-0.19190	2.85845	3.00539
H	-1.25125	4.05743	2.20712
C	-1.11618	-2.54077	2.50755
H	-1.51041	-3.56828	2.54876
H	-0.24719	-2.47370	3.17975
H	-1.88717	-1.83915	2.85519
H	-4.36240	-2.07252	-1.48456
H	-4.26188	2.22104	-1.66594

4.4.4. I4

I4_Co_L1

Co	-0.50221	0.27647	-0.49831
H	-0.11847	-1.93091	-3.01478
P	-2.21966	-0.94504	-0.17297
N	-3.36145	0.27919	0.42966
C	-2.85290	1.52302	0.59714
N	-3.62481	2.49916	1.08346
C	-3.03220	3.67732	1.19726
N	-1.77572	3.96469	0.89905
C	-1.05545	2.94164	0.42741
N	-1.55601	1.69437	0.24984
N	0.24678	3.13108	0.11532
P	1.14350	1.67974	-0.37811
N	2.47248	1.76469	0.67665
C	3.75257	1.70284	0.00468
H	4.11878	0.65791	-0.05481
H	4.50208	2.28289	0.57039
C	3.52043	2.28445	-1.38590
H	3.77362	3.36184	-1.41906
H	4.14982	1.78057	-2.13804
N	2.12221	2.05031	-1.69358
N	-2.24191	-2.19016	0.99126
C	-3.23731	-3.21079	0.73555
H	-4.19297	-2.98414	1.25353
H	-2.88992	-4.18621	1.11455
C	-3.41703	-3.22976	-0.77332
H	-2.69181	-3.92341	-1.24527
H	-4.42753	-3.56970	-1.05950
N	-3.18497	-1.87176	-1.22662
C	-4.72691	-0.01078	0.80597
H	-4.82515	-0.17556	1.89132
H	-5.05234	-0.91129	0.26991
H	-5.38401	0.82357	0.53083
C	0.86235	4.42082	0.33452
H	0.28800	5.21500	-0.16063
H	1.87779	4.39837	-0.07706
H	0.92045	4.66234	1.40711
C	3.10972	-2.23905	3.23524
C	1.85712	-2.21381	2.62487
C	4.25895	-2.18733	2.45000
H	0.95400	-2.25611	3.23918
H	5.24663	-2.21080	2.91776
C	1.75027	-2.12825	1.23841
C	4.14421	-2.12066	1.06288
H	0.76124	-2.08274	0.77313
H	5.05688	-2.11029	0.45454
C	2.89396	-2.08503	0.42918
H	3.18906	-2.30320	4.32344
Si	2.86818	-2.04616	-1.45888
H	3.23363	-0.68776	-1.96275
H	3.98768	-2.94902	-1.86783
O	1.54282	-2.77803	-2.18030
C	0.33675	-2.07181	-2.00786
H	-0.35885	-2.71559	-1.41193
O	0.62995	-0.89757	-1.39306
C	2.39231	1.34610	2.05046
H	2.83298	0.34607	2.20580
H	2.91282	2.06382	2.70734
H	1.33923	1.29976	2.36767
C	1.69434	1.91584	-3.05947
H	1.73687	2.87421	-3.60515
H	2.32020	1.18111	-3.59500
H	0.65917	1.54698	-3.08617
C	-1.90250	-1.90878	2.35965
H	-1.49405	-2.80936	2.84543
H	-1.13542	-1.11979	2.39792
H	-2.77719	-1.56929	2.94941
C	-3.16519	-1.64663	-2.64971
H	-4.17563	-1.74632	-3.07818
H	-2.80354	-0.63101	-2.86599
H	-2.50019	-2.36355	-3.16734
H	-3.64578	4.50012	1.58482

I4_Co_L2

Co	-0.71133	0.16125	0.35268
H	-1.04879	-2.78219	0.95934
P	-2.58828	-0.81700	0.09377
O	-3.60633	0.57727	-0.33567
C	-3.00610	1.73824	-0.36116
N	-3.71744	2.82205	-0.63036
C	-3.02827	3.95472	-0.64212
N	-1.72788	4.09292	-0.42875
C	-1.07693	2.96814	-0.16790
N	-1.67775	1.75548	-0.10704
O	0.21162	3.01553	0.03440
P	1.02423	1.43295	0.22505
N	2.17380	1.73358	1.39115
C	3.49929	2.01756	0.85935
H	4.14545	1.13121	0.99977
H	3.96206	2.86501	1.39197
C	3.30459	2.32322	-0.61655
H	3.13030	3.40637	-0.78163
H	4.18182	2.02582	-1.21383
N	2.13967	1.55585	-1.02934
N	-3.60106	-1.55287	1.22687
C	-4.62220	-2.35154	0.56636
H	-5.48461	-1.71876	0.27121
H	-4.99406	-3.13249	1.24806
C	-3.93692	-2.93758	-0.65422
H	-3.39755	-3.87096	-0.39673
H	-4.65621	-3.17569	-1.45534
N	-2.99026	-1.92773	-1.09391
C	5.72159	-1.04032	0.53270
C	4.73406	-1.52251	1.38966
C	5.45153	-0.91098	-0.82873
H	4.94206	-1.62796	2.45792
H	6.22235	-0.53801	-1.50850
C	3.48129	-1.87204	0.88877
C	4.20085	-1.27348	-1.32251
H	2.70834	-2.23084	1.57282
H	4.00744	-1.18529	-2.39905
C	3.19469	-1.76019	-0.47694
H	6.70517	-0.76960	0.92543
Si	1.55548	-2.27416	-1.24458
H	0.90158	-1.07784	-1.85402
H	1.86490	-3.21426	-2.36265
O	0.54395	-3.16096	-0.26050
C	0.04384	-2.56053	0.92935
H	0.50497	-3.10179	1.78521
O	0.34214	-1.24412	0.98047
C	2.07519	1.23396	2.74065
H	2.79954	0.41636	2.90293
H	2.27308	2.03087	3.47539
H	1.06970	0.82996	2.91890
C	1.69773	1.72109	-2.38993
H	1.50863	2.78325	-2.63694
H	2.45856	1.33098	-3.08431
H	0.76903	1.15507	-2.55390
C	-3.99068	-0.83323	2.41415
H	-4.29384	-1.53955	3.20174
H	-3.13867	-0.24890	2.79141

H	-4.83283	-0.14110	2.22242
C	-2.12387	-2.24795	-2.20295
H	-2.71388	-2.38579	-3.12270
H	-1.41210	-1.42786	-2.37131
H	-1.54325	-3.16804	-2.00835
H	-3.59209	4.87089	-0.85367

I4_Co_L3

Co	0.68642	0.44008	0.68281
H	0.06532	-1.80596	3.07859
P	2.23677	-0.99821	0.24348
C	3.65043	0.13396	-0.21828
C	3.10609	1.47026	-0.56236
N	3.83379	2.30331	-1.29201
C	3.30531	3.49943	-1.51441
N	2.15837	3.93061	-1.00904
C	1.46934	3.05854	-0.28625
N	1.86730	1.76888	-0.08919
C	0.18719	3.46776	0.33284
P	-0.88721	1.93415	0.43072
N	-1.95759	2.12441	-0.90284
C	-3.34499	2.06832	-0.47142
H	-3.71618	1.02332	-0.46138
H	-3.97674	2.64316	-1.16952
C	-3.36381	2.65359	0.93052
H	-3.44025	3.75970	0.89675
H	-4.22072	2.27774	1.51415
N	-2.12902	2.20548	1.53532
N	1.99896	-2.03330	-1.11498
C	2.95600	-3.12350	-1.08344
H	3.94422	-2.81214	-1.48927
H	2.59648	-3.96317	-1.70023
C	3.07542	-3.50043	0.37962
H	2.23485	-4.16102	0.67826
H	4.01358	-4.04316	0.58771
N	3.02842	-2.24981	1.11193
C	-2.93835	-2.00795	-3.24234
C	-1.75218	-2.24402	-2.55062
C	-4.09152	-1.67037	-2.53729
H	-0.84784	-2.51339	-3.10141
H	-5.02808	-1.48525	-3.06973
C	-1.71077	-2.13512	-1.16145
C	-4.04875	-1.58691	-1.14758
H	-0.76170	-2.30180	-0.64299
H	-4.97007	-1.34923	-0.60078
C	-2.86311	-1.81170	-0.43404
H	-2.96431	-2.09108	-4.33187
Si	-2.93323	-1.75271	1.44852
H	-3.26866	-0.37391	1.90930
H	-4.08500	-2.62156	1.83654
O	-1.64138	-2.49694	2.20573
C	-0.38819	-1.87043	2.06390
H	0.26265	-2.54511	1.45163
O	-0.57292	-0.65432	1.49124
C	-1.64478	1.49203	-2.16243
H	-1.84218	0.40462	-2.15639
H	-2.24447	1.95042	-2.96436
H	-0.58451	1.64953	-2.41287
C	-1.94832	2.18425	2.95686
H	-1.83452	3.19388	3.39633
H	-2.80856	1.69594	3.44423
H	-1.05388	1.59219	3.20127
C	1.76890	-1.42026	-2.39758
H	1.42714	-2.17912	-3.11808
H	0.98480	-0.65331	-2.31204
H	2.68176	-0.94937	-2.82007
C	2.97227	-2.33442	2.54829
H	3.93451	-2.68368	2.95721
H	2.76220	-1.34444	2.97901
H	2.18225	-3.02899	2.89345
H	3.86765	4.18953	-2.15318
H	0.37741	3.72018	1.39307
H	4.23453	0.23006	0.71461
H	-0.24163	4.34483	-0.16979
H	4.34415	-0.22811	-0.99154

I4_Co_L4

Co	-0.53027	0.28617	-0.48838
H	0.01432	-1.79018	-3.04905
P	-2.13248	-1.07344	-0.17816
N	-3.37721	0.03879	0.38535
C	-2.99306	1.34436	0.56488
C	-3.88042	2.31532	1.04936
C	-3.42019	3.61530	1.19183
C	-2.11053	3.93743	0.87395
C	-1.26532	2.92003	0.40711
N	-1.70648	1.64321	0.24749
N	0.04964	3.16300	0.10235
P	1.02325	1.76925	-0.34303
N	2.33328	1.92333	0.73899
C	3.62331	1.86594	0.08546
H	4.00103	0.82426	0.04220
H	4.35994	2.45872	0.65523
C	3.40570	2.42848	-1.31523
H	3.65062	3.50794	-1.35781
H	4.05239	1.92002	-2.05009
N	2.01541	2.17993	-1.64195
N	-2.01598	-2.30631	1.00132
C	-2.86500	-3.45011	0.74798
H	-3.84494	-3.35229	1.26293
H	-2.39390	-4.37075	1.13127
C	-3.03488	-3.49670	-0.76096
H	-2.21345	-4.07832	-1.22771
H	-3.98345	-3.98083	-1.05164
N	-3.00520	-2.11960	-1.21104
C	-4.71640	-0.37966	0.71287
H	-4.91925	-0.31161	1.79598
H	-4.85083	-1.41848	0.39220
H	-5.46960	0.22395	0.18068
C	0.61644	4.47545	0.27832
H	0.10096	5.22529	-0.34344
H	1.66903	4.44896	-0.02440
H	0.57667	4.80524	1.33079
C	3.22898	-2.16064	3.20749
C	1.97938	-2.20341	2.59200
C	4.37470	-2.01217	2.42948
H	1.07919	-2.32114	3.20042
H	5.36035	-1.98102	2.90119
C	1.87028	-2.08761	1.20767
C	4.25946	-1.91715	1.04425
H	0.88122	-2.09253	0.74026
H	5.17099	-1.82883	0.44036
C	3.01122	-1.94596	0.40615
H	3.30890	-2.24682	4.29417
Si	2.98924	-1.84424	-1.47921
H	3.29670	-0.45466	-1.93381
H	4.15191	-2.67916	-1.91320
O	1.70479	-2.60610	-2.24044
C	0.46592	-1.96370	-2.04507
H	-0.20471	-2.67178	-1.49447
O	0.69808	-0.81400	-1.36424
C	2.23959	1.45533	2.09690
H	2.63313	0.43028	2.21445
H	2.79784	2.12361	2.77381
H	1.18711	1.45145	2.41964
C	1.62008	2.00906	-3.01331

H	1.70216	2.94885	-3.58625
H	2.23851	1.24078	-3.51010
H	0.57559	1.66895	-3.05686
C	-1.73896	-1.96612	2.36958
H	-1.22666	-2.79987	2.87641
H	-1.08125	-1.08391	2.40792
H	-2.66033	-1.73521	2.94172
C	-3.04200	-1.89152	-2.63269
H	-4.03198	-2.14583	-3.04535
H	-2.84675	-0.83105	-2.84865
H	-2.28290	-2.49404	-3.16691
H	-4.09431	4.39116	1.56226
H	-4.90391	2.05308	1.30948
H	-1.74086	4.95417	0.99117

I4_Co_L5

Co	-0.13925	0.27621	-0.63893
H	-2.16255	1.93828	-2.75985
P	0.49114	2.27855	-0.25627
N	2.22081	1.99613	0.07067
C	2.60900	0.70268	0.07954
N	3.87792	0.38754	0.35171
C	4.14562	-0.90446	0.32297
N	3.31343	-1.89305	0.07620
C	2.05343	-1.51508	-0.17944
N	1.65418	-0.21954	-0.19533
N	1.11675	-2.45445	-0.42240
P	-0.54731	-1.85220	-0.62056
N	-1.36197	-2.83278	0.50082
C	-2.50191	-3.52582	-0.06288
H	-3.42886	-2.92997	0.06173
H	-2.65302	-4.48561	0.46013
C	-2.17426	-3.73018	-1.53792
H	-1.71445	-4.72144	-1.71518
H	-3.08379	-3.67842	-2.15871
N	-1.27939	-2.64801	-1.90465
N	-0.07662	3.16965	1.07842
C	0.03599	4.60468	0.91093
H	0.99485	4.98595	1.32022
H	-0.77324	5.11665	1.45717
C	-0.06131	4.84532	-0.58656
H	-1.11873	4.96980	-0.89601
H	0.47729	5.76008	-0.88863
N	0.51569	3.67654	-1.22384
C	3.16173	3.04822	0.38850
H	3.29153	3.16236	1.47664
H	2.78785	3.98885	-0.03495
H	4.14353	2.82774	-0.04816
C	1.46400	-3.85793	-0.36509
H	2.31952	-4.07379	-1.01842
H	0.59955	-4.44491	-0.69463
H	1.73104	-4.16451	0.65764
C	-3.92935	-0.32630	3.67199
C	-3.04908	0.50782	2.98518
C	-4.89090	-1.04398	2.96462
H	-2.29565	1.07416	3.53871
H	-5.58929	-1.69758	3.49354
C	-3.12129	0.61936	1.59817
C	-4.96840	-0.91338	1.57960
H	-2.40984	1.26053	1.06963
H	-5.74907	-1.46147	1.03783
C	-4.08705	-0.08688	0.86824
H	-3.86767	-0.41272	4.75972
Si	-4.33119	0.04919	-0.99878
H	-3.86101	-1.19464	-1.67979
H	-5.81303	0.08934	-1.18732
O	-3.84297	1.49805	-1.68608
C	-2.44894	1.68884	-1.71292
H	-2.20996	2.57900	-1.07786
O	-1.86356	0.54710	-1.26709
C	-1.35501	-2.55085	1.91226
H	-2.27516	-2.03552	2.23762
H	-1.25085	-3.48292	2.49267
H	-0.50180	-1.90139	2.16108
C	-1.23066	-2.17445	-3.26172
H	-0.75154	-2.90173	-3.93926
H	-2.24641	-1.96306	-3.63766
H	-0.66298	-1.23406	-3.30319
C	0.03318	2.63535	2.40917
H	-0.76217	3.04492	3.05193
H	-0.07751	1.54030	2.38084
H	1.00814	2.87261	2.87896
C	0.42859	3.59666	-2.66029
H	1.09317	4.33531	-3.13654
H	0.73257	2.59675	-3.00187
H	-0.60219	3.78118	-3.01694
C	5.59714	-1.25761	0.62843
F	5.82322	-2.55969	0.56424
F	6.41309	-0.65342	-0.23563
F	5.92419	-0.84123	1.85283

I4_Co_L6

Co	-0.40418	-0.27616	-0.66936
H	1.39956	-2.49834	-2.73983
P	-1.32337	-2.13394	-0.06180
N	-2.83636	-1.53404	0.60616
C	-2.99519	-0.18680	0.58657
N	-4.11524	0.34200	1.08387
C	-4.18912	1.66200	1.01726
N	-3.27641	2.47687	0.51373
C	-2.18226	1.87995	0.03510
N	-1.98543	0.53853	0.05230
N	-1.19960	2.64339	-0.50578
P	0.21027	1.76328	-1.06890
C	-3.88287	-2.35481	1.18060
H	-4.05919	-2.09159	2.23357
H	-3.59170	-3.41019	1.12570
H	-4.83111	-2.22175	0.64022
C	-1.35866	4.08285	-0.53970
H	-2.28483	4.36257	-1.06070
H	-0.50804	4.52829	-1.06947
H	-1.40477	4.50464	0.47538
C	2.89356	0.83983	3.33820
C	1.92331	0.05833	2.71353
C	4.10281	1.08476	2.69000
H	0.97146	-0.13837	3.21596
H	4.87322	1.68944	3.17574
C	2.15631	-0.46260	1.44302
C	4.32918	0.55249	1.42256
H	1.36826	-1.03577	0.94449
H	5.28938	0.74590	0.92920
C	3.35886	-0.21912	0.76776
H	2.70946	1.25497	4.33261
Si	3.75118	-0.80095	-0.98464
H	3.62936	0.36150	-1.91690
H	5.19374	-1.18447	-0.96178
O	2.99580	-2.19740	-1.50225
C	1.59887	-2.12326	-1.70929
H	1.13368	-2.85604	-1.00087
O	1.17437	-0.85599	-1.51895
H	-5.09768	2.12638	1.41952
C	0.44127	2.33005	-2.78881
H	0.68581	3.39957	-2.87125
H	1.27588	1.73768	-3.19453
H	-0.45789	2.09886	-3.37491
C	-1.89780	-3.42563	-1.22905

H	-1.00839	-3.90167	-1.66777
H	-2.50898	-4.20405	-0.74839
H	-2.46751	-2.95073	-2.03882
C	1.59393	2.59564	-0.21569
H	1.51391	2.43297	0.86809
H	2.52456	2.11905	-0.55984
H	1.64157	3.67177	-0.43952
C	-0.68602	-3.15807	1.32254
H	-1.38169	-3.95785	1.61537
H	0.26283	-3.61883	1.01127
H	-0.48505	-2.50815	2.18542

I4_Co_L7

Co	-0.70488	-0.05300	-0.20658
H	-0.28979	-2.86334	-1.44685
P	-2.33543	-1.35868	0.10024
N	-3.66542	-0.25918	0.29248
C	-3.33350	1.05956	0.23253
N	-4.27604	1.98875	0.37008
C	-3.85113	3.23879	0.27620
N	-2.60401	3.63151	0.07786
C	-1.70758	2.65227	-0.03150
N	-2.02373	1.33501	0.02733
N	-0.39300	2.95268	-0.20235
P	0.68176	1.58477	-0.28534
O	1.82849	2.05415	0.80064
C	3.11643	2.21587	0.21916
H	3.70682	1.30338	0.38989
H	3.61285	3.06171	0.71501
C	2.88549	2.47408	-1.26875
H	2.84690	3.55149	-1.50157
H	3.66115	2.00631	-1.89124
O	1.63649	1.87986	-1.58975
O	-2.46280	-2.38115	1.39479
C	-3.15681	-3.58375	1.10426
H	-4.20494	-3.49979	1.43902
H	-2.67953	-4.40576	1.65448
C	-3.05966	-3.76329	-0.40594
H	-2.14923	-4.31825	-0.68926
H	-3.93307	-4.28161	-0.82517
O	-3.00504	-2.45515	-0.94827
C	-5.02129	-0.69244	0.56253
H	-5.15252	-0.94010	1.62712
H	-5.24520	-1.57769	-0.04749
H	-5.72026	0.10867	0.29973
C	0.07366	4.32059	-0.30266
H	0.26237	4.59513	-1.35159
H	1.00398	4.42704	0.27192
H	-0.68134	4.99811	0.10977
C	5.14288	-0.27093	2.28327
C	3.79004	-0.59170	2.37596
C	5.81827	-0.46573	1.07947
H	3.25523	-0.43953	3.31675
H	6.88148	-0.22399	1.00045
C	3.10997	-1.09389	1.26811
C	5.13581	-0.98172	-0.01997
H	2.04105	-1.31271	1.33762
H	5.68370	-1.15175	-0.95438
C	3.77019	-1.29873	0.04814
H	5.67360	0.12801	3.15177
Si	2.94936	-1.97824	-1.50995
H	2.59795	-0.86718	-2.43945
H	4.00490	-2.80068	-2.17308
O	1.72829	-3.09549	-1.24024
C	0.52185	-2.59529	-0.73081
H	0.31426	-3.11699	0.23598
O	0.64844	-1.24663	-0.56819
H	-4.60771	4.02616	0.37447

I4_Co_L8

Co	0.90222	0.45855	-0.48638
H	-0.88727	3.13506	-1.32470
N	1.76974	1.93451	0.67506
C	3.21224	1.62173	0.77044
C	3.44226	0.16376	0.56739
N	4.51935	-0.48363	0.95349
C	4.59972	-1.75963	0.56473
N	3.69957	-2.38416	-0.20170
C	2.64910	-1.67456	-0.54746
N	2.44877	-0.39720	-0.15198
C	1.58292	-2.12933	-1.48606
N	0.35257	-1.35483	-1.24225
C	-3.37086	-2.09209	2.35088
C	-2.92307	-0.78689	2.55303
C	-3.90211	-2.45852	1.11617
H	-2.50854	-0.49533	3.52172
H	-4.25467	-3.48017	0.95259
C	-3.00400	0.14413	1.52085
C	-3.97624	-1.52054	0.08807
H	-2.64226	1.16420	1.68488
H	-4.38151	-1.82603	-0.88410
C	-3.52411	-0.20758	0.26881
H	-3.30605	-2.82525	3.15909
Si	-3.59561	1.02587	-1.14897
H	-3.22766	0.30809	-2.40457
H	-4.99365	1.51650	-1.30571
O	-2.72587	2.40830	-0.85432
C	-1.32677	2.37334	-0.63701
H	-1.18280	2.75864	0.40724
O	-0.82355	1.14097	-0.82278
H	5.47308	-2.33286	0.88823
H	1.93369	-1.91091	-2.50999
H	3.72986	2.14592	-0.05213
H	1.41252	-3.21946	-1.42473
H	3.65370	1.98782	1.71511
C	1.14169	1.74645	1.98867
H	1.34964	0.73387	2.35988
H	1.53281	2.48384	2.71657
H	0.05469	1.86407	1.89956
C	-0.40978	-1.97805	-0.15419
H	0.22483	-2.08785	0.73615
H	-1.26448	-1.34419	0.09986
H	-0.77242	-2.97874	-0.45969
C	-0.46250	-1.29716	-2.45383
H	-1.38102	-0.74120	-2.24323
H	0.08722	-0.76447	-3.24084
H	-0.71403	-2.31653	-2.80351
C	1.62758	3.32634	0.24311
H	0.58374	3.64053	0.32552
H	2.24212	3.99507	0.87546
H	1.94606	3.42101	-0.80375

I4_Co_L9

Co	-0.38282	-0.22165	-0.58007
H	1.17726	-2.31686	-2.76266
P	-1.25072	-2.11020	-0.07971
N	-2.84039	-1.75199	0.49329
C	-3.10607	-0.37314	0.53714
C	-4.33729	0.11279	1.00791
C	-4.56014	1.48765	1.02017
C	-3.58025	2.36917	0.57006
C	-2.35619	1.85501	0.10953
C	-2.06867	0.46992	0.07807
N	-1.34357	2.70950	-0.35400
P	0.07903	1.86857	-0.85082

C	-3.77354	-2.70087	1.01526
H	-3.99280	-2.52490	2.08685
H	-3.37534	-3.72041	0.92406
H	-4.73847	-2.67558	0.47370
C	-1.52414	4.12727	-0.38567
H	-2.38365	4.42150	-1.01840
H	-0.63104	4.61528	-0.79894
H	-1.69648	4.55091	0.62296
C	1.66575	-2.06486	-1.79287
O	1.35044	-0.80998	-1.34599
O	3.05665	-2.00990	-1.93660
H	-5.51535	1.87895	1.38430
C	-0.53985	-3.10024	1.31341
H	-1.12492	-4.00532	1.54220
H	0.48880	-3.39926	1.06204
H	-0.50288	-2.45417	2.20270
C	1.41593	2.75100	0.06103
H	1.31767	2.54090	1.13571
H	2.38769	2.36185	-0.27766
H	1.38992	3.83901	-0.11010
C	-1.57101	-3.47275	-1.28726
H	-2.08245	-3.05027	-2.16322
H	-0.60672	-3.89060	-1.61270
H	-2.17937	-4.28820	-0.86427
C	0.39040	2.50190	-2.55208
H	0.52956	3.59446	-2.59291
H	1.30220	2.00997	-2.92322
H	-0.45175	2.20848	-3.19397
H	-3.77342	3.44466	0.58553
H	-5.11891	-0.56502	1.36010
Si	3.36531	-0.44907	-1.27731
H	4.87362	-0.52688	-1.46663
H	3.09246	0.82668	-2.02828
C	3.34167	-0.14379	0.60671
C	2.23875	-0.37474	1.44211
C	4.48901	0.40528	1.19739
C	2.27473	-0.06072	2.79898
C	4.53050	0.74115	2.54977
C	3.41864	0.50649	3.35733
H	1.30936	-0.76050	1.00406
H	5.37464	0.57862	0.57501
H	1.39165	-0.24384	3.41893
H	5.43577	1.18320	2.97770
H	3.44458	0.76587	4.42022
H	1.39740	-2.86524	-1.05712

I4_Co_L10

Co	0.60172	0.62032	0.38169
H	-1.47658	2.93021	2.09175
C	1.35699	-2.03558	1.36549
C	2.53510	-1.39119	0.69770
C	3.81580	-1.92910	0.59579
C	4.80434	-1.22674	-0.10920
C	4.50710	0.00376	-0.71408
C	3.22083	0.52539	-0.60421
C	2.22511	-0.15179	0.11751
C	2.71604	1.78278	-1.24816
C	-1.75047	1.89523	1.77747
O	-1.17608	1.52679	0.59289
O	-3.12887	1.82612	1.50393
H	5.81380	-1.64164	-0.18842
H	5.29007	0.53811	-1.26709
H	4.06315	-2.89322	1.05793
Si	-3.09923	1.40241	-0.16625
H	-4.61549	1.39785	-0.30963
H	-2.70647	2.42221	-1.19741
C	-2.75789	-0.36949	-0.76926
C	-1.53769	-0.74818	-1.35191
C	-3.73814	-1.35929	-0.62350
C	-1.30378	-2.06076	-1.75786
C	-3.51296	-2.67970	-1.01669
C	-2.28981	-3.03197	-1.58628
H	-0.72634	-0.01192	-1.44683
H	-4.70464	-1.08621	-0.18385
H	-0.33438	-2.32490	-2.19211
H	-4.29257	-3.43532	-0.87790
H	-2.10392	-4.06537	-1.89569
H	-1.48807	1.21650	2.62484
O	0.32225	-1.05998	1.50317
O	1.47274	2.13821	-0.63650
C	0.68512	3.01090	-1.40255
H	1.23078	3.95138	-1.60271
H	0.41091	2.54169	-2.36681
H	-0.23077	3.20777	-0.83268
C	-0.91703	-1.61186	1.86347
H	-1.64230	-0.79392	1.94036
H	-1.26700	-2.32719	1.09736
H	-0.83569	-2.12975	2.83681
H	2.52632	1.61970	-2.33082
H	0.95577	-2.87166	0.75283
H	3.41391	2.64101	-1.16630
H	1.58990	-2.45122	2.36775

I4_Co_L11

Co	0.52960	-0.47335	-0.26410
H	-1.51235	-2.94345	-1.94866
C	1.27009	2.15740	-1.22380
C	2.45213	1.56836	-0.50975
C	3.71694	2.12768	-0.35150
C	4.66778	1.46913	0.44505
C	4.33811	0.27005	1.09736
C	3.06732	-0.27354	0.92970
C	2.11842	0.34597	0.09838
C	2.48227	-1.46435	1.63424
C	-1.81274	-1.89895	-1.67851
O	-1.22469	-1.44951	-0.53935
O	-3.19156	-1.86735	-1.39598
H	5.66810	1.89688	0.56483
H	5.08286	-0.22045	1.73708
H	3.97952	3.07709	-0.83532
Si	-3.19914	-1.38349	0.25114
H	-4.71251	-1.41413	0.39076
H	-2.76375	-2.38036	1.28314
C	-2.88551	0.39720	0.83174
C	-1.67128	0.80076	1.41156
C	-3.88544	1.36654	0.68568
C	-1.46748	2.11639	1.82430
C	-3.68726	2.69116	1.08018
C	-2.47275	3.06714	1.65304
H	-0.83937	0.08703	1.49578
H	-4.84662	1.07497	0.24665
H	-0.50343	2.39868	2.25879
H	-4.48156	3.43087	0.93971
H	-2.30862	4.10334	1.96492
H	-1.60813	-1.26542	-2.57740
N	0.35278	1.06558	-1.64491
N	1.33922	-1.99948	0.84918
C	-0.98672	1.59132	-1.87208
H	-0.97619	2.37837	-2.65558
H	-1.65450	0.78129	-2.19471
H	-1.38435	2.01379	-0.94124
C	0.39897	-2.70144	1.70981
H	0.88949	-3.54763	2.23561
H	-0.01047	-2.00723	2.45671
H	-0.43565	-3.07430	1.10277
C	1.83306	-2.91183	-0.17844

H	1.00663	-3.20868	-0.83767
H	2.60113	-2.40962	-0.78015
H	2.27307	-3.82187	0.28778
C	0.86101	0.47203	-2.87984
H	1.90030	0.15145	-2.73603
H	0.25844	-0.40365	-3.15382
H	0.81946	1.21125	-3.71054
H	2.07112	-1.14470	2.60875
H	0.69565	2.79573	-0.52859
H	3.21145	-2.27583	1.84998
H	1.53342	2.79183	-2.09881

I4_Fe_L1

Fe	-0.46796	0.26265	-0.61215
H	0.02890	-1.77367	-3.33154
P	-2.04491	-1.03455	-0.14172
N	-3.38142	0.11064	0.19516
C	-2.99884	1.40875	0.26681
N	-3.88413	2.35344	0.59217
C	-3.39402	3.58332	0.67597
N	-2.12075	3.92521	0.53711
C	-1.28695	2.92828	0.23331
N	-1.68273	1.64373	-0.00235
N	0.05162	3.14143	0.16521
P	1.03242	1.67751	-0.10151
N	2.18962	1.82129	1.19579
C	3.55661	1.95782	0.75625
H	4.04367	0.96107	0.67362
H	4.14674	2.54740	1.48375
C	3.50019	2.63742	-0.60412
H	3.56403	3.74495	-0.50323
H	4.34969	2.32612	-1.24127
N	2.25943	2.21082	-1.18250
N	-2.06669	-2.12889	1.22837
C	-2.96539	-3.24543	1.10497
H	-3.97489	-3.01415	1.51611
H	-2.58833	-4.12020	1.66676
C	-3.04111	-3.53077	-0.38430
H	-2.20637	-4.20041	-0.69233
H	-3.98258	-4.04995	-0.65087
N	-2.94730	-2.25016	-1.03228
C	-4.73430	-0.25764	0.51876
H	-4.92432	-0.22873	1.60662
H	-4.91798	-1.27323	0.14444
H	-5.44583	0.43270	0.04378
C	0.63268	4.43822	0.38719
H	0.98851	4.88572	-0.55727
H	1.49354	4.33895	1.06667
H	-0.10912	5.11091	0.83554
C	3.00122	-2.61096	2.90903
C	1.78059	-2.46310	2.25361
C	4.18787	-2.42873	2.20105
H	0.84223	-2.59115	2.79983
H	5.15263	-2.53851	2.70535
C	1.74220	-2.12893	0.90140
C	4.14045	-2.12144	0.84219
H	0.77434	-1.96681	0.41820
H	5.08087	-2.01535	0.28760
C	2.92338	-1.96314	0.16403
H	3.02797	-2.86529	3.97268
Si	2.97829	-1.64329	-1.70264
H	3.29009	-0.22012	-2.02480
H	4.21415	-2.39718	-2.11913
O	1.81305	-2.46957	-2.59652
C	0.53442	-1.91236	-2.34915
H	-0.06656	-2.65451	-1.76399
O	0.73235	-0.75141	-1.67973
C	1.98515	1.11373	2.42815
H	2.57385	0.17696	2.47196
H	2.26444	1.73654	3.29787
H	0.92259	0.84249	2.52501
C	1.98670	2.47715	-2.55716
H	1.89014	3.56206	-2.77579
H	2.78563	2.07205	-3.20563
H	1.04400	1.98406	-2.84072
C	-1.89108	-1.57752	2.53803
H	-1.46412	-2.32625	3.22905
H	-1.20033	-0.72119	2.47942
H	-2.84568	-1.21924	2.98130
C	-2.91771	-2.25290	-2.46484
H	-3.90530	-2.51440	-2.88741
H	-2.64560	-1.25088	-2.83149
H	-2.17659	-2.97282	-2.87050
H	-4.10538	4.38937	0.89778

I4_Fe_L4

Fe	-0.46964	0.27948	-0.69641
H	0.35943	-1.53224	-3.52941
P	-1.84485	-1.20357	-0.19367
N	-3.34832	-0.25682	0.00678
C	-3.15417	1.09540	0.10104
C	-4.19472	1.98537	0.40353
C	-3.90361	3.33779	0.54076
C	-2.58643	3.76969	0.43371
C	-1.58776	2.83011	0.14262
N	-1.86702	1.51291	-0.09977
N	-0.25824	3.16221	0.11264
P	0.85419	1.78824	-0.04536
N	1.85685	1.99782	1.38621
C	3.26286	2.08047	1.06939
H	3.71495	1.06648	0.98602
H	3.81213	2.61619	1.86666
C	3.34936	2.80618	-0.26427
H	3.42725	3.90851	-0.11679
H	4.25040	2.49900	-0.82900
N	2.16071	2.42687	-0.96952
N	-1.73855	-2.15836	1.28445
C	-2.38431	-3.44020	1.24504
H	-3.43457	-3.39349	1.61791
H	-1.85435	-4.17284	1.88444
C	-2.35134	-3.85113	-0.21630
H	-1.37695	-4.33573	-0.45801
H	-3.14286	-4.58939	-0.45061
N	-2.53465	-2.63501	-0.96142
C	-4.63112	-0.82325	0.30411
H	-4.93964	-0.64456	1.35300
H	-4.58568	-1.90427	0.12498
H	-5.41902	-0.41307	-0.35234
C	0.17010	4.49751	0.40785
H	-0.24685	5.22445	-0.31189
H	1.26286	4.54457	0.33878
H	-0.11710	4.82055	1.42632
C	2.88721	-2.55994	2.84135
C	1.73225	-2.51945	2.06285
C	4.10858	-2.19008	2.27990
H	0.76216	-2.79608	2.48496
H	5.02124	-2.21384	2.88305
C	1.79494	-2.10234	0.73527
C	4.16521	-1.80316	0.94166
H	0.86812	-2.02186	0.16304
H	5.13702	-1.54517	0.50244
C	3.01455	-1.74711	0.14300
H	2.83590	-2.87703	3.88712
Si	3.19881	-1.28865	-1.68427
H	3.42364	0.17333	-1.87432
H	4.51181	-1.92405	-2.05789

O	2.15297	-2.10568	-2.71630
C	0.81493	-1.66797	-2.52188
H	0.25350	-2.49129	-2.01207
O	0.86111	-0.53122	-1.79390
C	1.53134	1.22684	2.55517
H	1.96967	0.20868	2.53050
H	1.89195	1.73634	3.46662
H	0.43914	1.11714	2.63246
C	2.01763	2.74430	-2.35196
H	1.92113	3.83643	-2.53473
H	2.88173	2.37723	-2.93685
H	1.11590	2.24932	-2.74442
C	-1.74922	-1.45599	2.53059
H	-1.24215	-2.03560	3.32325
H	-1.21505	-0.49999	2.40759
H	-2.77897	-1.23276	2.88769
C	-2.53540	-2.74449	-2.38962
H	-3.46604	-3.21676	-2.75586
H	-2.46455	-1.74083	-2.83726
H	-1.68249	-3.34267	-2.77288
H	-4.70118	4.05299	0.75969
H	-5.21183	1.61677	0.53384
H	-2.32687	4.81676	0.58881

I4_Fe_L6

Fe	-0.38162	-0.23599	-0.56716
H	1.10810	-2.42728	-2.71334
P	-1.28326	-2.10309	-0.05009
N	-2.86396	-1.60701	0.52666
C	-3.07357	-0.25538	0.52637
N	-4.23461	0.21618	0.96030
C	-4.37053	1.53943	0.93164
N	-3.43761	2.38880	0.50965
C	-2.30240	1.84433	0.09273
N	-2.02733	0.50084	0.06481
N	-1.30057	2.66248	-0.35275
P	0.16189	1.84118	-0.85177
C	-3.89681	-2.46608	1.04373
H	-4.13876	-2.21927	2.09051
H	-3.56593	-3.51153	0.99864
H	-4.82920	-2.36697	0.46473
C	-1.51275	4.08651	-0.34581
H	-0.63752	4.59063	-0.77557
H	-1.67307	4.46840	0.67607
H	-2.40138	4.35916	-0.93673
C	3.46501	0.50210	3.29977
C	2.32370	-0.10182	2.77617
C	4.55268	0.75175	2.46383
H	1.45988	-0.29766	3.41933
H	5.45613	1.22242	2.86406
C	2.26338	-0.43749	1.42512
C	4.48842	0.39377	1.11787
H	1.33190	-0.85278	1.01730
H	5.35593	0.57809	0.47319
C	3.34162	-0.19156	0.56220
H	3.50850	0.77771	4.35804
Si	3.33693	-0.53483	-1.31320
H	3.04952	0.72320	-2.08716
H	4.83885	-0.62869	-1.53241
O	2.99459	-2.11073	-1.91229
C	1.60428	-2.15031	-1.75548
H	1.33083	-2.92093	-0.99046
O	1.29924	-0.87836	-1.34847
H	-5.31977	1.96048	1.28272
C	-0.69274	-3.14399	1.37054
H	-1.36661	-3.98196	1.61157
H	0.29761	-3.55357	1.11946
H	-0.58115	-2.49478	2.25077
C	1.45392	2.76077	0.09763
H	1.34817	2.53396	1.16799
H	2.43965	2.39665	-0.23053
H	1.40803	3.84990	-0.06267
C	0.46587	2.57022	-2.52154
H	0.61144	3.66285	-2.51134
H	1.37576	2.09280	-2.91582
H	-0.37484	2.30802	-3.17833
C	-1.74971	-3.48041	-1.20362
H	-0.82261	-3.96889	-1.54063
H	-2.39510	-4.24519	-0.74183
H	-2.25314	-3.05003	-2.08022

I4_Fe_L7

Fe	-0.57731	-0.06542	-0.54483
H	-0.05426	-2.66290	-2.49910
P	-1.97001	-1.41920	0.17272
N	-3.48325	-0.49992	0.16741
C	-3.30714	0.82973	-0.02447
N	-4.33890	1.67190	0.06138
C	-4.02956	2.95468	-0.06343
N	-2.80824	3.45688	-0.16747
C	-1.82421	2.55738	-0.22937
N	-2.02584	1.21173	-0.27970
N	-0.52432	2.94239	-0.21340
P	0.62030	1.59941	-0.12206
O	1.44762	2.07925	1.27814
C	2.78981	2.43298	1.06434
H	3.44435	1.57128	1.28019
H	3.05999	3.25250	1.75094
C	2.89993	2.86243	-0.39844
H	2.78678	3.95860	-0.50488
H	3.87069	2.57587	-0.83476
O	1.87231	2.20016	-1.08563
O	-2.01255	-2.16527	1.70567
C	-2.65503	-3.41130	1.74020
H	-3.71026	-3.29724	2.05706
H	-2.15028	-4.06417	2.47014
C	-2.56692	-3.95918	0.32150
H	-1.61883	-4.51237	0.17402
H	-3.40082	-4.64177	0.08640
O	-2.61843	-2.84563	-0.52370
C	-4.77391	-1.02539	0.53234
H	-4.94862	-0.96413	1.62066
H	-4.83353	-2.07515	0.21551
H	-5.56510	-0.45324	0.03141
C	-0.13476	4.32557	-0.11901
H	0.47144	4.61684	-0.99134
H	0.45978	4.49327	0.79327
H	-1.03157	4.95491	-0.07949
C	4.39014	-0.67870	2.55873
C	3.04497	-0.94112	2.30985
C	5.29120	-0.63936	1.49508
H	2.32534	-0.95922	3.13249
H	6.35060	-0.43600	1.67870
C	2.59909	-1.15758	1.00721
C	4.83901	-0.87080	0.19755
H	1.53314	-1.32408	0.82411
H	5.56118	-0.86295	-0.62757
C	3.48716	-1.12950	-0.07828
H	4.73746	-0.50178	3.58106
Si	3.00021	-1.47547	-1.88073
H	2.81071	-0.22816	-2.67244
H	4.27929	-2.06712	-2.42067
O	1.96904	-2.79327	-2.13600
C	0.67810	-2.43716	-1.69452
H	0.41201	-3.06518	-0.80800
O	0.74476	-1.11028	-1.38339

H	-4.86215	3.66979	-0.05580

I4_Fe_L8

Fe	0.87578	0.48255	-0.54062
H	-0.88414	3.23211	-1.18081
N	1.86492	1.96797	0.66669
C	3.30040	1.58706	0.62157
C	3.42362	0.10856	0.55704
N	4.39111	-0.60907	1.04919
C	4.39818	-1.92664	0.73654
N	3.51874	-2.48663	-0.13366
C	2.57470	-1.72092	-0.59218
N	2.37720	-0.40096	-0.20126
C	1.59265	-2.06526	-1.65670
N	0.31864	-1.36295	-1.37653
C	-3.34664	-2.17924	2.30609
C	-2.68422	-0.96797	2.50106
C	-4.06749	-2.39245	1.13268
H	-2.10333	-0.80210	3.41230
H	-4.58121	-3.34411	0.96945
C	-2.73683	0.02112	1.52269
C	-4.12150	-1.39297	0.16312
H	-2.18001	0.95099	1.66527
H	-4.68303	-1.57553	-0.76165
C	-3.45306	-0.17477	0.33554
H	-3.29146	-2.96334	3.06646
Si	-3.51775	1.12425	-1.02629
H	-3.18542	0.48608	-2.33304
H	-4.95275	1.54643	-1.13782
O	-2.74548	2.55284	-0.67191
C	-1.32726	2.45988	-0.50730
H	-1.12899	2.79133	0.54691
O	-0.90613	1.21508	-0.76669
H	5.19436	-2.55380	1.14444
H	1.96327	-1.66491	-2.62027
H	3.69579	2.01584	-0.32083
H	1.44465	-3.15793	-1.76304
H	3.86255	2.02662	1.46936
C	1.34961	1.76778	2.01622
H	1.55021	0.73608	2.33525
H	1.83320	2.46572	2.73344
H	0.26368	1.93484	2.03041
C	-0.41480	-2.07954	-0.33902
H	0.22702	-2.21231	0.54283
H	-1.29868	-1.50383	-0.04729
H	-0.73383	-3.07991	-0.70231
C	-0.49080	-1.25125	-2.57668
H	-1.42435	-0.72725	-2.33962
H	0.05120	-0.66026	-3.32819
H	-0.72722	-2.25208	-2.99723
C	1.73425	3.36285	0.27813
H	0.70349	3.70370	0.42840
H	2.40504	4.01099	0.88206
H	1.99188	3.47147	-0.78529

I4_Fe_L9

Fe	-0.32149	-0.23769	-0.58398
H	1.31951	-2.36620	-2.78597
P	-1.20073	-2.09488	-0.08125
N	-2.82558	-1.77462	0.47428
C	-3.11117	-0.39958	0.49829
C	-4.35886	0.07085	0.93764
C	-4.61781	1.44522	0.93653
C	-3.62975	2.33054	0.49383
C	-2.38977	1.83032	0.06493
C	-2.05302	0.44338	0.04468
N	-1.38128	2.69827	-0.38616
P	0.06749	1.85183	-0.85200
C	-3.74351	-2.72427	1.00083
H	-3.98572	-2.53371	2.06924
H	-3.32737	-3.73976	0.93523
H	-4.70919	-2.72959	0.45169
C	-1.58221	4.10546	-0.42716
H	-2.43402	4.39088	-1.08093
H	-0.68711	4.60954	-0.81896
H	-1.79154	4.53551	0.57607
C	1.79516	-2.12325	-1.80770
O	1.47856	-0.86176	-1.36682
O	3.19114	-2.05071	-1.91802
H	-5.58798	1.82537	1.27707
C	-0.54883	-3.12349	1.34827
H	-1.14792	-4.02613	1.56604
H	0.48449	-3.43176	1.12247
H	-0.52664	-2.47489	2.23700
C	1.35804	2.82195	0.08400
H	1.23173	2.62484	1.15884
H	2.35276	2.45735	-0.21699
H	1.30784	3.90923	-0.10322
C	-1.53825	-3.54186	-1.22996
H	-2.04816	-3.15288	-2.12311
H	-0.57077	-3.96907	-1.53876
H	-2.14587	-4.34621	-0.77630
C	0.40877	2.59196	-2.53086
H	0.50603	3.69325	-2.52772
H	1.35067	2.14970	-2.89200
H	-0.40222	2.28872	-3.20841
H	-3.83469	3.40632	0.49182
H	-5.13504	-0.62232	1.27922
Si	3.34825	-0.44501	-1.24612
H	4.88415	-0.47796	-1.37651
H	3.12763	0.84132	-2.01174
C	3.29836	-0.10950	0.63885
C	2.16853	-0.34187	1.44391
C	4.41857	0.46668	1.25298
C	2.16373	0.00506	2.79638
C	4.41943	0.83025	2.60088
C	3.28288	0.59557	3.37844
H	1.24445	-0.74140	0.98930
H	5.31767	0.64146	0.64837
H	1.25930	-0.17028	3.38892
H	5.30571	1.29599	3.04681
H	3.27173	0.87679	4.43783
H	1.50076	-2.91467	-1.07406

I4_Fe_L10

Fe	-1.34002	0.53396	-0.11184
H	1.66832	0.91151	-0.95663
O	-2.77369	2.05480	-0.27152
C	-4.03904	1.62042	0.22257
C	-4.13312	0.13008	0.07791
C	-5.31435	-0.59944	0.11599
C	-5.27191	-2.00852	0.08017
C	-4.02304	-2.65637	-0.00383
C	-2.85333	-1.91011	-0.07464
C	-2.86187	-0.49363	-0.03100
C	-1.48935	-2.48889	-0.29323
O	-0.49348	-1.53786	0.06744
C	1.36017	1.38376	0.02845
O	0.14151	1.86465	0.02239
O	2.38469	2.40752	0.30273
H	-6.19801	-2.59254	0.12504
H	-1.31558	-3.42704	0.27915
H	-4.09249	1.90965	1.29619

H	-1.34111	-2.73564	-1.37044
H	-4.84641	2.17372	-0.30707
H	-3.98546	-3.75584	-0.03047
H	-6.28671	-0.08849	0.18329
C	0.78654	-1.94676	-0.33035
H	0.87471	-1.94331	-1.43529
H	1.52799	-1.25643	0.08879
H	1.00572	-2.96711	0.04112
C	-2.41467	3.34519	0.13004
H	-2.38975	3.41400	1.23530
H	-1.39923	3.52915	-0.24488
H	-3.13510	4.09102	-0.26520
Si	3.90322	2.03548	-0.25347
H	4.07914	2.30576	-1.72547
H	4.80611	2.98929	0.48184
C	4.33446	0.24739	0.00227
C	4.11996	-0.39292	1.26033
C	4.75502	-0.58284	-1.06405
C	4.35141	-1.75084	1.42943
C	4.98712	-1.93938	-0.90077
C	4.80027	-2.54623	0.36451
H	3.74119	0.19473	2.10452
H	4.88495	-0.13875	-2.05932
H	4.16130	-2.20931	2.40648
H	5.29761	-2.54429	-1.75997
H	4.96633	-3.61921	0.50144
H	1.53396	0.60120	0.81706

I4_Fe_L11

Fe	-1.50185	0.24107	0.17757
H	2.25836	1.14659	0.43545
N	-2.20164	2.22905	0.01690
C	-3.59986	2.23723	0.50926
C	-4.26040	0.92417	0.20018
C	-5.62166	0.67248	0.10824
C	-6.08619	-0.65340	-0.05552
C	-5.15113	-1.70870	-0.17094
C	-3.79254	-1.43615	-0.10911
C	-3.30302	-0.12166	0.10893
C	-2.66039	-2.38271	-0.38214
N	-1.40562	-1.89045	0.24236
C	1.54078	0.37965	0.03537
O	0.33624	0.85925	-0.18269
O	2.17212	-0.12279	-1.18169
H	-7.16096	-0.85828	-0.11780
H	-2.85358	-3.43597	-0.06837
H	-3.53281	2.35969	1.60792
H	-2.46453	-2.40493	-1.47079
H	-4.14303	3.12669	0.11025
H	-5.51255	-2.73599	-0.32898
H	-6.34982	1.49368	0.18519
C	-1.37222	-2.26709	1.64637
H	-1.31045	-3.37727	1.75738
H	-0.50241	-1.80397	2.13349
H	-2.27899	-1.89751	2.14307
C	-1.37339	3.17797	0.73320
H	-1.36766	2.91748	1.80202
H	-0.34380	3.09530	0.35761
H	-1.74599	4.22217	0.60813
C	-0.25411	-2.43982	-0.45091
H	-0.20929	-2.03170	-1.47024
H	0.66796	-2.14360	0.06473
H	-0.29804	-3.55250	-0.49291
C	-2.15702	2.50374	-1.40988
H	-2.46541	3.55587	-1.62114
H	-1.13536	2.33575	-1.77791
H	-2.83697	1.81792	-1.93301
Si	3.55035	-1.05242	-1.08963

H	3.22295	-2.31905	-0.34044
H	3.87193	-1.37057	-2.52040
C	5.05632	-0.33437	-0.28366
C	6.01661	0.43767	-1.00485
C	5.33863	-0.53929	1.09953
C	7.15408	0.94802	-0.40353
C	6.47361	-0.02967	1.70510
C	7.41216	0.72647	0.96652
H	5.85031	0.63088	-2.07245
H	4.62952	-1.11865	1.70463
H	7.86117	1.53430	-1.00301
H	6.64017	-0.21292	2.77312
H	8.30311	1.14308	1.44655
H	1.57176	-0.47938	0.77298

I4_Ni_L1

Ni	-0.50249	0.30181	-0.47207
H	-0.36199	-2.14924	-2.81164
P	-2.33071	-0.86721	-0.13721
N	-3.31100	0.40766	0.56811
C	-2.71423	1.61043	0.75326
N	-3.39663	2.60786	1.30567
C	-2.73599	3.75209	1.41858
N	-1.48916	3.98620	1.04214
C	-0.84738	2.94996	0.50575
N	-1.42465	1.72927	0.35306
N	0.43196	3.09173	0.09781
P	1.24652	1.63850	-0.48167
N	2.61387	1.57990	0.46674
C	3.84482	1.44607	-0.29594
H	4.16448	0.38859	-0.33550
H	4.64372	2.01746	0.20326
C	3.54303	1.99751	-1.68854
H	3.88630	3.04134	-1.79719
H	4.04330	1.40352	-2.46912
N	2.09725	1.90439	-1.87652
N	-2.35234	-2.18545	0.89059
C	-3.30980	-3.20949	0.49056
H	-4.26427	-3.08898	1.03893
H	-2.91458	-4.20718	0.73492
C	-3.49966	-3.03321	-1.00886
H	-2.77237	-3.64214	-1.57848
H	-4.50852	-3.33485	-1.33084
N	-3.29297	-1.61476	-1.28285
C	-4.68044	0.19634	1.00969
H	-4.73868	0.13313	2.10588
H	-5.04995	-0.73493	0.56517
H	-5.31959	1.02375	0.67884
C	1.11926	4.35950	0.28160
H	0.53230	5.17907	-0.15034
H	2.09076	4.30596	-0.22240
H	1.27824	4.57161	1.34867
C	3.01129	-2.09026	3.33306
C	1.75980	-2.07697	2.72005
C	4.16161	-2.14081	2.54961
H	0.85754	-2.05140	3.33666
H	5.14672	-2.16349	3.02114
C	1.65797	-2.10962	1.33095
C	4.05156	-2.18346	1.16114
H	0.66650	-2.10700	0.86667
H	4.96601	-2.25416	0.55989
C	2.80279	-2.16641	0.52358
H	3.08853	-2.07170	4.42249
Si	2.76925	-2.30772	-1.35439
H	3.18154	-1.01196	-1.97317
H	3.76363	-3.34037	-1.74180
O	1.31785	-2.89258	-1.94897
C	0.15189	-2.14263	-1.82587

H	-0.51386	-2.65268	-1.09109
O	0.48032	-0.86678	-1.43637
C	2.61642	1.34254	1.88872
H	3.04159	0.35639	2.13344
H	3.20029	2.11931	2.40896
H	1.58845	1.37169	2.27981
C	1.55295	1.78939	-3.20866
H	1.76234	2.69299	-3.80135
H	1.97651	0.91470	-3.72825
H	0.46397	1.65546	-3.16225
C	-1.95999	-2.09216	2.27549
H	-1.47144	-3.02434	2.59660
H	-1.23988	-1.26998	2.40791
H	-2.82408	-1.90651	2.93870
C	-3.34862	-1.16771	-2.65696
H	-4.36739	-1.28205	-3.05509
H	-3.08073	-0.10361	-2.72108
H	-2.65675	-1.73918	-3.30047
H	-3.27902	4.58971	1.87125

I4_Ni_L2

Ni	-0.63199	0.28367	0.24995
H	-0.91645	-2.37098	1.90132
P	-2.52127	-0.84263	0.09973
O	-3.41930	0.36291	-0.80678
C	-2.82389	1.49173	-1.09742
N	-3.49331	2.42713	-1.73801
C	-2.81861	3.54562	-1.97630
N	-1.57046	3.80739	-1.60970
C	-0.95258	2.83701	-0.96786
N	-1.53309	1.63661	-0.71232
O	0.27131	3.02622	-0.54800
P	1.05136	1.69917	0.30423
N	1.62813	2.33305	1.71412
C	3.08846	2.38557	1.75961
H	3.46194	1.55183	2.38180
H	3.41871	3.32596	2.22662
C	3.57593	2.26946	0.32129
H	3.75569	3.26191	-0.13005
H	4.50942	1.68835	0.25341
N	2.52530	1.56987	-0.41381
N	-3.40143	-1.18088	1.45734
C	-4.34654	-2.27512	1.23469
H	-5.32250	-1.88496	0.89228
H	-4.50623	-2.81926	2.17688
C	-3.69431	-3.14688	0.17465
H	-3.00675	-3.88834	0.62253
H	-4.43931	-3.69052	-0.42563
N	-2.94837	-2.23116	-0.68551
C	5.66619	-1.56326	-0.13828
C	4.72552	-1.13605	0.79844
C	5.24753	-2.22214	-1.29119
H	5.05828	-0.64351	1.71626
H	5.98074	-2.57091	-2.02169
C	3.36844	-1.34893	0.57587
C	3.88957	-2.44976	-1.50296
H	2.63340	-0.99586	1.30546
H	3.57659	-2.98616	-2.40521
C	2.92652	-2.01020	-0.58178
H	6.73105	-1.39542	0.03962
Si	1.13015	-2.28430	-1.01286
H	0.53980	-1.00781	-1.56552
H	1.02437	-3.31857	-2.07322
O	0.12910	-2.81109	0.22300
C	0.07888	-2.14630	1.46425
H	0.84856	-2.57503	2.13700
O	0.32630	-0.80420	1.35317
C	0.90546	2.29069	2.96653
H	1.24803	1.44966	3.59205
H	1.04713	3.23018	3.51967
H	-0.17003	2.16403	2.78291
C	2.74789	1.24610	-1.80361
H	2.97186	2.15082	-2.39263
H	3.58654	0.53857	-1.89274
H	1.85464	0.76763	-2.22835
C	-3.63039	-0.22512	2.51583
H	-3.72937	-0.74915	3.47695
H	-2.77127	0.45750	2.59463
H	-4.54239	0.37254	2.34591
C	-2.30074	-2.75370	-1.86610
H	-3.05502	-3.14661	-2.56299
H	-1.74939	-1.95642	-2.38390
H	-1.59513	-3.56024	-1.60676
H	-3.34727	4.33297	-2.52456

I4_Ni_L3

Ni	-0.72315	0.46244	-0.60934
H	-0.03314	-1.68803	-2.97456
P	-2.24105	-1.12868	-0.24559
C	-3.66252	-0.05503	0.29812
C	-3.17044	1.28432	0.71152
N	-3.93330	2.04609	1.47744
C	-3.46902	3.25923	1.74647
N	-2.35218	3.77587	1.25139
C	-1.61933	2.97509	0.49654
N	-1.95836	1.68214	0.25266
C	-0.37798	3.49961	-0.12851
P	0.77431	2.07772	-0.46447
N	1.94630	2.15577	0.74381
C	3.28537	2.03920	0.17111
H	3.60127	0.98158	0.12712
H	3.99787	2.58015	0.81307
C	3.20053	2.64672	-1.22245
H	3.42949	3.72665	-1.20710
H	3.90896	2.16026	-1.91096
N	1.83906	2.40578	-1.69212
N	-1.87562	-2.19610	1.01841
C	-2.71696	-3.38466	0.90831
H	-3.71279	-3.21513	1.36818
H	-2.24304	-4.22523	1.43684
C	-2.84489	-3.64373	-0.57998
H	-1.96401	-4.19565	-0.96115
H	-3.74189	-4.23611	-0.81822
N	-2.93197	-2.32778	-1.20633
C	3.13715	-1.81844	3.27664
C	1.95901	-2.14377	2.60814
C	4.26392	-1.44469	2.54771
H	1.08412	-2.45549	3.18316
H	5.19574	-1.19931	3.06244
C	1.89806	-2.08557	1.21629
C	4.20313	-1.40760	1.15719
H	0.96050	-2.34342	0.71204
H	5.10603	-1.13898	0.59465
C	3.02267	-1.71813	0.46636
H	3.18015	-1.86854	4.36701
Si	3.06523	-1.67865	-1.41217
H	3.36239	-0.28686	-1.86702
H	4.15985	-2.56350	-1.88406
O	1.69201	-2.31866	-2.11833
C	0.43912	-1.73775	-1.97049
H	-0.18205	-2.41125	-1.33391
O	0.58075	-0.48545	-1.41753
C	1.71176	1.59358	2.05590
H	1.81098	0.49350	2.07098
H	2.43422	2.01724	2.76816
H	0.70609	1.86715	2.41183

C	1.55568	2.26051	-3.09861
H	1.71685	3.20361	-3.64430
H	2.19223	1.47919	-3.54459
H	0.51056	1.95310	-3.24209
C	-1.63011	-1.67676	2.34305
H	-1.19685	-2.46780	2.97122
H	-0.90311	-0.85167	2.29966
H	-2.55271	-1.32050	2.84316
C	-2.95838	-2.27170	-2.65054
H	-3.90328	-2.68545	-3.03246
H	-2.88307	-1.23074	-2.99630
H	-2.12481	-2.84339	-3.09649
H	-4.06086	3.88845	2.42006
H	-0.64370	3.90192	-1.12401
H	-4.29047	0.08126	-0.60163
H	0.04994	4.32330	0.45924
H	-4.31560	-0.49532	1.06637

I4_Ni_L4

Ni	-0.53137	0.30765	-0.45490
H	-0.28464	-2.12395	-2.77375
P	-2.28492	-0.95488	-0.13367
N	-3.31623	0.24325	0.58558
C	-2.80996	1.51052	0.75632
C	-3.57146	2.53715	1.31838
C	-2.99486	3.79323	1.43775
C	-1.69700	4.02569	1.01454
C	-0.97316	2.96105	0.46625
N	-1.53000	1.72360	0.35022
N	0.31905	3.11408	0.03572
P	1.16884	1.68350	-0.48727
N	2.53489	1.66257	0.46875
C	3.77628	1.53974	-0.27559
H	4.13203	0.49311	-0.26900
H	4.55214	2.15557	0.20794
C	3.47567	2.02492	-1.69463
H	3.82999	3.05856	-1.85606
H	3.97216	1.38740	-2.44350
N	2.03092	1.93915	-1.88146
N	-2.22757	-2.29702	0.86696
C	-3.08168	-3.39186	0.42858
H	-4.04272	-3.38438	0.97952
H	-2.59439	-4.35666	0.63770
C	-3.29238	-3.18336	-1.06470
H	-2.52276	-3.71550	-1.65539
H	-4.27506	-3.55644	-1.39350
N	-3.20481	-1.74638	-1.29288
C	-4.66049	-0.07741	1.02069
H	-4.77156	0.04986	2.10896
H	-4.87731	-1.12088	0.76868
H	-5.40893	0.54701	0.50958
C	1.00083	4.38474	0.17008
H	0.47495	5.17985	-0.37868
H	2.00680	4.29160	-0.25370
H	1.10128	4.68145	1.22670
C	3.10549	-1.97098	3.34938
C	1.85258	-1.99722	2.73971
C	4.25487	-1.99301	2.56316
H	0.95098	-1.99243	3.35799
H	5.24146	-1.98437	3.03212
C	1.74814	-2.03912	1.35116
C	4.14238	-2.04649	1.17533
H	0.75659	-2.06332	0.88881
H	5.05691	-2.09354	0.57181
C	2.89199	-2.06679	0.54112
H	3.18503	-1.94377	4.43853
Si	2.85857	-2.20607	-1.33703
H	3.23738	-0.90077	-1.95624
H	3.88147	-3.21012	-1.72724
O	1.42598	-2.82756	-1.93805
C	0.23601	-2.11924	-1.79107
H	-0.40414	-2.66798	-1.06217
O	0.52519	-0.84331	-1.37495
C	2.52666	1.46218	1.89553
H	2.97588	0.49493	2.17148
H	3.08203	2.26743	2.40455
H	1.49288	1.47104	2.27269
C	1.49497	1.78554	-3.21218
H	1.72048	2.66645	-3.83293
H	1.91073	0.88945	-3.70165
H	0.40363	1.66907	-3.16977
C	-1.86134	-2.20612	2.25802
H	-1.31081	-3.10716	2.56835
H	-1.20550	-1.33668	2.41884
H	-2.74591	-2.09776	2.91214
C	-3.29468	-1.26616	-2.65314
H	-4.28974	-1.48330	-3.06846
H	-3.15067	-0.17663	-2.68059
H	-2.53577	-1.73374	-3.30571
H	-3.57373	4.61132	1.87109
H	-4.59184	2.35609	1.64861
H	-1.24608	5.01111	1.10780

I4_Ni_L5

Ni	-0.13624	0.30430	-0.63500
H	-2.23767	2.26313	-2.42549
P	0.53627	2.35559	-0.22467
N	2.21865	1.98823	0.14328
C	2.57611	0.68500	0.13779
N	3.82532	0.34035	0.43250
C	4.07771	-0.95593	0.37078
N	3.24259	-1.92222	0.05455
C	1.99716	-1.52707	-0.22274
N	1.61923	-0.22364	-0.17700
N	1.06682	-2.44166	-0.55327
P	-0.58570	-1.84700	-0.77897
N	-1.46018	-2.81244	0.25663
C	-2.60058	-3.45581	-0.37816
H	-3.52501	-2.88042	-0.18867
H	-2.73673	-4.45940	0.05520
C	-2.27318	-3.52423	-1.86866
H	-1.87003	-4.51186	-2.15319
H	-3.16917	-3.34459	-2.48262
N	-1.29722	-2.46951	-2.13587
N	-0.09233	3.25771	1.03226
C	-0.10450	4.69057	0.75968
H	0.78655	5.17941	1.19879
H	-0.99166	5.15082	1.22038
C	-0.12684	4.82346	-0.75621
H	-1.16368	4.85780	-1.14039
H	0.38154	5.74027	-1.09286
N	0.56494	3.64983	-1.28063
C	3.18691	3.01106	0.50974
H	3.38386	3.00009	1.59131
H	2.78818	3.98859	0.21553
H	4.13512	2.84335	-0.01485
C	1.40277	-3.85701	-0.57075
H	2.29689	-4.03026	-1.18142
H	0.55910	-4.40933	-0.99921
H	1.59826	-4.22866	0.44525
C	-3.58392	-0.63499	3.80849
C	-2.75072	0.24002	3.11381
C	-4.62400	-1.27397	3.13813
H	-1.94346	0.75165	3.64445
H	-5.28963	-1.95237	3.67658
C	-2.95271	0.47212	1.75496

C	-4.82637	-1.02888	1.78139
H	-2.29414	1.17000	1.22766
H	-5.66877	-1.51620	1.27586
C	-3.99764	-0.15591	1.06215
H	-3.42790	-0.80997	4.87534
Si	-4.41434	0.15798	-0.74757
H	-4.00920	-1.01649	-1.57757
H	-5.88772	0.30884	-0.84891
O	-3.81125	1.60954	-1.32390
C	-2.43997	1.81527	-1.42856
H	-2.13868	2.55734	-0.65303
O	-1.79397	0.61102	-1.27696
C	-1.36262	-2.77556	1.69520
H	-2.26872	-2.34637	2.15105
H	-1.20941	-3.79031	2.09669
H	-0.50687	-2.15627	2.00270
C	-1.21309	-1.88748	-3.45492
H	-0.91456	-2.64119	-4.19925
H	-2.18153	-1.45399	-3.75241
H	-0.46701	-1.08191	-3.46709
C	-0.07175	2.80256	2.40132
H	-0.97440	3.14394	2.92968
H	-0.05729	1.70225	2.43272
H	0.81282	3.17660	2.94736
C	0.63679	3.48130	-2.71536
H	1.25614	4.27253	-3.16218
H	1.09799	2.51459	-2.96197
H	-0.36409	3.51726	-3.18028
C	5.51989	-1.34039	0.71327
F	5.70928	-2.64267	0.64630
F	6.34814	-0.74269	-0.13512
F	5.80569	-0.92858	1.94293

I4_Ni_L6

Ni	-0.37935	-0.32506	-0.89248
H	1.11684	-3.51408	-2.08040
P	-2.14689	-1.42791	-0.10199
N	-2.88562	-0.10642	0.77272
C	-2.26921	1.09801	0.71440
N	-2.77395	2.12265	1.39601
C	-2.10433	3.26056	1.28867
N	-1.01116	3.46783	0.56994
C	-0.56066	2.41295	-0.10166
N	-1.15297	1.19215	-0.04653
N	0.53614	2.54177	-0.88552
P	1.06583	1.09375	-1.69097
C	-4.11059	-0.23342	1.55364
H	-3.91717	-0.03867	2.61697
H	-4.50814	-1.24842	1.44491
H	-4.86536	0.48345	1.20561
C	1.24305	3.81425	-0.93626
H	0.54431	4.63341	-1.14477
H	1.99479	3.77617	-1.73357
H	1.74533	4.01923	0.02051
C	2.53836	1.31462	2.62893
C	1.28848	0.76148	2.36166
C	3.69236	0.60246	2.30190
H	0.38034	1.30154	2.64579
H	4.67580	1.02233	2.52505
C	1.19734	-0.49119	1.75695
C	3.59054	-0.64565	1.69173
H	0.21002	-0.92989	1.58306
H	4.50853	-1.18457	1.43089
C	2.34241	-1.21230	1.39607
H	2.61384	2.29226	3.11061
Si	2.25901	-2.89958	0.56710
H	3.28107	-2.95015	-0.51840
H	2.49154	-3.96678	1.57113
O	0.73655	-3.17737	-0.07910
C	0.43823	-2.91784	-1.43625
H	-0.59250	-3.31025	-1.59365
O	0.56732	-1.60925	-1.78215
H	-2.49408	4.11654	1.85131
C	0.96402	1.42052	-3.46995
H	1.64710	2.22419	-3.78158
H	1.25260	0.48789	-3.97880
H	-0.06638	1.67074	-3.75304
C	-3.41277	-1.94402	-1.30154
H	-3.03223	-2.81650	-1.85269
H	-4.35066	-2.22870	-0.80281
H	-3.60284	-1.13414	-2.01780
C	2.83132	0.92998	-1.34937
H	3.01522	0.97361	-0.26779
H	3.13455	-0.06190	-1.71783
H	3.41662	1.70030	-1.87113
C	-2.10063	-2.77962	1.10837
H	-3.11176	-3.10749	1.38561
H	-1.54934	-3.61827	0.66202
H	-1.55230	-2.46136	2.00508

I4_Ni_L7

Ni	-0.76483	-0.01879	-0.03561
H	-0.05913	-2.43480	-1.59650
P	-2.45615	-1.39767	0.11876
N	-3.74682	-0.26828	0.23423
C	-3.40713	1.05205	0.18683
N	-4.34818	1.98004	0.25734
C	-3.92176	3.23272	0.17707
N	-2.66646	3.62638	0.03259
C	-1.76214	2.65811	-0.01916
N	-2.08913	1.34166	0.06148
N	-0.44435	2.96462	-0.15581
P	0.64682	1.62689	-0.21366
O	1.80010	2.03505	0.83691
C	3.09498	2.12856	0.21059
H	3.63598	1.19224	0.39556
H	3.62949	2.96363	0.67967
C	2.83235	2.35973	-1.27487
H	2.80418	3.42742	-1.54015
H	3.56760	1.84858	-1.90863
O	1.54131	1.78904	-1.54730
O	-2.61644	-2.42060	1.36489
C	-3.23980	-3.65684	0.98320
H	-4.30461	-3.61658	1.25822
H	-2.75628	-4.46666	1.54211
C	-3.03746	-3.78013	-0.52407
H	-2.10436	-4.30496	-0.77853
H	-3.87645	-4.27938	-1.02381
O	-2.96324	-2.43657	-1.02126
C	-5.12846	-0.70126	0.41380
H	-5.29384	-1.03888	1.44653
H	-5.34559	-1.51877	-0.28566
H	-5.79763	0.13792	0.20052
C	0.01675	4.34138	-0.28995
H	0.22731	4.57611	-1.34290
H	0.92672	4.47446	0.30929
H	-0.75741	5.02076	0.07997
C	5.57678	-0.06321	1.89155
C	4.33012	-0.58184	2.23659
C	6.03521	-0.17108	0.57876
H	3.97354	-0.50612	3.26650
H	7.01723	0.22260	0.30607
C	3.53844	-1.20267	1.27136
C	5.24590	-0.80248	-0.37934
H	2.55932	-1.60175	1.54709
H	5.63006	-0.90452	-1.40156

C	3.98433	-1.32320	-0.05174
H	6.19890	0.41825	2.64979
Si	2.99927	-2.20004	-1.38346
H	2.50352	-1.22873	-2.40487
H	3.86643	-3.21565	-2.03088
O	1.69480	-3.04330	-0.74067
C	0.45546	-2.45068	-0.60816
H	-0.13967	-3.08673	0.08900
O	0.60929	-1.16790	-0.10820
H	-4.68338	4.01822	0.23371

I4_Ni_L8

Ni	0.85830	0.45761	-0.50328
H	-0.95723	3.06473	-1.42366
N	1.57242	1.91482	0.69258
C	3.02801	1.69390	0.89949
C	3.39125	0.27839	0.61905
N	4.53060	-0.28380	0.96517
C	4.70412	-1.53222	0.52875
N	3.84815	-2.22056	-0.22773
C	2.73057	-1.59377	-0.53085
N	2.46693	-0.35564	-0.10216
C	1.66786	-2.12744	-1.42703
N	0.41920	-1.35882	-1.22788
C	-3.15883	-1.92918	2.54021
C	-2.82560	-0.57664	2.61167
C	-3.67133	-2.45581	1.35631
H	-2.44646	-0.15666	3.54718
H	-3.94842	-3.51122	1.30114
C	-3.00065	0.24148	1.49817
C	-3.83362	-1.63203	0.24399
H	-2.75540	1.30690	1.57009
H	-4.22717	-2.06262	-0.68428
C	-3.49537	-0.27300	0.29154
H	-3.03251	-2.57114	3.41536
Si	-3.67416	0.80127	-1.23819
H	-3.30674	-0.03543	-2.41744
H	-5.05545	1.31007	-1.40602
O	-2.74195	2.18094	-1.12266
C	-1.39189	2.24871	-0.80671
H	-1.32093	2.56269	0.26403
O	-0.76929	1.04922	-1.02630
H	5.63443	-2.03479	0.81266
H	2.02066	-1.99592	-2.46442
H	3.59504	2.31325	0.18369
H	1.51420	-3.21036	-1.28233
H	3.34826	2.01154	1.90628
C	0.85752	1.65145	1.96087
H	1.09699	0.64292	2.32593
H	1.15595	2.38935	2.72489
H	-0.22476	1.71645	1.79658
C	-0.35203	-1.95478	-0.11697
H	0.27038	-2.02118	0.78688
H	-1.22789	-1.33209	0.09225
H	-0.68569	-2.96988	-0.39255
C	-0.38302	-1.37354	-2.46015
H	-1.32648	-0.84976	-2.28361
H	0.15999	-0.85763	-3.26150
H	-0.58346	-2.41621	-2.75866
C	1.38554	3.32165	0.28947
H	0.32779	3.58849	0.32976
H	1.93470	3.98253	0.98085
H	1.75989	3.46880	-0.73146

I4_Ni_L9

Ni	-0.32333	-0.39157	-0.86722
H	1.85270	-3.27455	-2.07806
P	-1.83149	-1.77644	-0.09434
N	-2.86102	-0.75440	0.81644
C	-2.53012	0.60649	0.74008
C	-3.26719	1.57649	1.43767
C	-2.88999	2.91122	1.33551
C	-1.80775	3.30012	0.55234
C	-1.08567	2.31908	-0.14274
C	-1.42184	0.95251	-0.05442
N	0.01372	2.65634	-0.94480
P	0.75900	1.29625	-1.65721
C	-4.03157	-1.19762	1.52481
H	-3.97058	-0.96512	2.60210
H	-4.14251	-2.28529	1.43000
H	-4.95063	-0.73126	1.12785
C	0.46877	4.01310	-1.07401
H	-0.31587	4.67145	-1.48581
H	1.32808	4.05682	-1.75610
H	0.79096	4.43082	-0.10300
C	2.25184	1.79116	2.63364
C	1.14185	1.01887	2.30349
C	3.53515	1.31067	2.36825
H	0.12972	1.38800	2.49209
H	4.41017	1.91130	2.62971
C	1.31977	-0.22745	1.70473
C	3.69981	0.06932	1.75920
H	0.43841	-0.82355	1.45163
H	4.71398	-0.28462	1.53799
C	2.59511	-0.71912	1.40591
H	2.11758	2.77019	3.10068
Si	2.87703	-2.37098	0.55102
H	3.88489	-2.17316	-0.53601
H	3.41607	-3.33541	1.55120
O	1.48898	-3.05837	-0.06083
C	1.08836	-2.80483	-1.41759
H	0.14757	-3.39496	-1.53816
O	0.96086	-1.50421	-1.70756
H	-3.45779	3.67062	1.87955
C	0.64247	1.50131	-3.46595
H	1.16075	2.40740	-3.81550
H	1.10754	0.61670	-3.92671
H	-0.41452	1.53988	-3.76063
C	-2.90107	-2.55624	-1.35796
H	-2.30100	-3.26784	-1.94400
H	-3.74555	-3.09538	-0.90231
H	-3.28060	-1.77726	-2.03289
C	2.54604	1.44644	-1.35715
H	2.73489	1.54917	-0.27997
H	3.00843	0.51222	-1.70956
H	2.98688	2.29459	-1.90144
C	-1.46727	-3.17184	1.02689
H	-2.36874	-3.75600	1.26170
H	-0.71551	-3.82120	0.55745
H	-1.03269	-2.77976	1.95683
H	-4.12263	1.30065	2.05659
H	-1.53569	4.35500	0.48536

I4_Ni_L10

Ni	0.06877	-0.70024	0.76375
H	3.69199	-1.76248	1.50495
C	-2.15788	0.97503	1.02529
C	-3.48180	1.35371	0.80361
C	-4.32828	0.49184	0.10267
C	-3.86835	-0.73697	-0.37781
C	-2.54247	-1.10465	-0.15094
C	-1.70059	-0.24583	0.54748
C	-0.88504	1.94188	-2.17752

C	0.06885	2.74281	-1.55153
C	-0.58656	0.61423	-2.47043
H	-0.15373	3.79172	-1.33567
H	-1.33238	-0.01300	-2.96609
C	1.30962	2.20858	-1.21211
C	0.64999	0.08392	-2.10998
H	2.05773	2.85235	-0.73420
H	0.87119	-0.96657	-2.32931
C	1.62078	0.86632	-1.46974
H	-1.86318	2.35279	-2.44036
Si	3.29347	0.16505	-0.96751
H	3.47512	-1.16342	-1.61997
H	4.33135	1.12594	-1.43621
O	3.54270	0.11647	0.68669
C	2.93137	-0.95498	1.41781
H	2.76334	-0.55807	2.44614
O	1.82229	-1.40795	0.81594
H	-5.36666	0.78327	-0.07352
H	-3.85980	2.31398	1.16797
H	-4.54741	-1.39688	-0.92678
C	-1.88299	-2.35310	-0.63692
H	-2.40208	-3.27618	-0.31589
H	-1.82094	-2.38256	-1.74402
C	-1.11808	1.78801	1.71740
H	-0.99718	2.78755	1.25655
H	-1.33487	1.94073	2.79274
O	-0.55738	-2.35984	-0.10775
O	0.12683	1.08977	1.59566
C	1.21020	1.78178	2.18690
H	2.14686	1.36090	1.80701
H	1.15979	2.84535	1.90168
H	1.16352	1.70054	3.28545
C	0.27080	-3.38861	-0.61033
H	0.34598	-3.31205	-1.70904
H	1.25792	-3.24956	-0.15583
H	-0.15347	-4.36980	-0.34302

I4_Ni_L11

Ni	-0.15403	0.80734	0.49542
H	3.39108	2.16124	1.25354
C	-2.51970	0.53114	-0.98807
C	-3.73908	-0.06034	-1.32002
C	-4.16819	-1.18065	-0.60304
C	-3.39186	-1.71782	0.42991
C	-2.17744	-1.11522	0.75222
C	-1.75450	0.00873	0.04794
C	0.21461	-3.35713	-1.36098
C	0.01768	-2.05487	-1.81034
C	1.39332	-3.68997	-0.69286
H	-0.91155	-1.78679	-2.32064
H	1.55458	-4.71215	-0.34086
C	0.99463	-1.08684	-1.58227
C	2.36201	-2.71468	-0.46916
H	0.83413	-0.06629	-1.94137
H	3.27690	-2.98606	0.07109
C	2.17843	-1.39324	-0.90106
H	-0.55464	-4.11477	-1.53167
Si	3.50845	-0.10038	-0.58081
H	4.12438	-0.39532	0.75004
H	4.54314	-0.20878	-1.64724
O	2.93647	1.45862	-0.63315
C	2.50626	2.11799	0.57454
H	2.31442	3.16968	0.25076
O	1.48065	1.52054	1.17883
H	-5.12469	-1.64611	-0.85467
H	-4.35543	0.33635	-2.13287
H	-3.74051	-2.60345	0.97028
C	-1.15694	-1.59078	1.73602
H	-1.58020	-2.00623	2.67143
H	-0.54950	-2.38920	1.27542
C	-1.82987	1.64551	-1.70619
H	-1.35573	1.25876	-2.62503
H	-2.49706	2.46946	-2.02694
N	-0.23580	-0.46574	2.05028
N	-0.74033	2.19021	-0.84487
C	0.27301	2.81043	-1.70060
H	-0.20464	3.53656	-2.38399
H	1.01350	3.33365	-1.08913
H	0.79564	2.04811	-2.28964
C	-1.28988	3.21080	0.06108
H	-0.51352	3.52277	0.77213
H	-1.63122	4.09026	-0.51690
H	-2.14146	2.79714	0.61619
C	-0.82434	0.38874	3.09072
H	-0.89469	-0.16744	4.04454
H	-0.18958	1.27219	3.23418
H	-1.83124	0.70669	2.79116
C	1.04680	-0.98286	2.52895
H	1.51958	-1.58185	1.74145
H	1.70227	-0.14028	2.77348
H	0.88681	-1.61656	3.42088

4.4.5. I5

I5_Co_L1

Co	-0.73936	0.54071	-0.04068
H	1.49374	-1.15063	1.33649
P	-1.27355	-1.52296	-0.28163
N	0.29473	-2.14180	-0.89422
C	1.23227	-1.22329	-1.22929
N	2.39999	-1.60405	-1.76042
C	3.24295	-0.60891	-1.99588
N	3.04968	0.68322	-1.77355
C	1.84867	0.98994	-1.26932
N	0.91872	0.05953	-0.99925
N	1.49879	2.27500	-1.03098
P	-0.09136	2.55256	-0.28652
N	-0.75537	3.71179	-1.33953
C	-1.12432	4.94095	-0.66429
H	-2.17855	4.90741	-0.32279
H	-1.02275	5.79600	-1.35404
C	-0.17827	5.05446	0.51960
H	0.75243	5.59051	0.24215
H	-0.63648	5.60898	1.35457
N	0.08775	3.69244	0.93323
N	-2.39490	-2.12209	-1.39365
C	-2.96936	-3.39665	-1.02081
H	-2.44200	-4.23308	-1.52486
H	-4.02533	-3.43966	-1.34015
C	-2.84986	-3.48990	0.49437
H	-3.77630	-3.13510	0.98424
H	-2.67882	-4.53004	0.82284
N	-1.73795	-2.64034	0.88982
C	0.55706	-3.54967	-1.09449
H	0.44735	-3.84325	-2.15082
H	-0.14641	-4.12585	-0.48026
H	1.58080	-3.79320	-0.78086
C	2.44753	3.35478	-1.17628
H	2.91759	3.59807	-0.20926
H	1.92754	4.24406	-1.55826
H	3.23274	3.06669	-1.88414
C	5.76239	-3.22995	-0.29416
C	6.26628	-1.95952	-0.56052
C	4.73187	-3.38864	0.63074
H	7.07770	-1.83170	-1.28169
H	4.33655	-4.38483	0.84417
C	5.72871	-0.84970	0.08886
C	4.21045	-2.27739	1.28567
H	6.11819	0.14764	-0.14281
H	3.41004	-2.42035	2.02034
C	4.69252	-0.98586	1.02134
H	6.17578	-4.10187	-0.80751
Si	3.95153	0.50920	1.87861
H	4.07393	0.32626	3.35789
H	4.66779	1.73306	1.42398
O	2.34959	0.71971	1.49287
C	1.31444	-0.20261	1.90925
H	1.51059	-0.44184	2.98533
O	0.10040	0.31035	1.75358
C	-4.74207	-0.52064	1.81470
C	-4.15499	0.12588	0.71863
C	-5.82236	-1.38912	1.65656
H	-6.26402	-1.87665	2.53000
C	-4.70020	-0.12825	-0.54940
C	-6.34313	-1.62702	0.38682
H	-4.26304	0.36079	-1.42793
H	-7.19269	-2.30309	0.25807
C	-5.78104	-0.98787	-0.71893
H	-6.19089	-1.16165	-1.71827
H	-4.34799	-0.33448	2.82013
Si	-2.64961	1.26529	0.92549
H	-1.53107	0.76286	-1.28588
H	-3.10103	2.59849	0.37905
H	-2.48844	1.45506	2.40716
H	4.21082	-0.88879	-2.42892
C	0.68925	3.44386	2.22035
H	0.12449	3.98005	2.99987
H	0.65393	2.36970	2.43790
H	1.74230	3.78114	2.26071
C	-1.51372	3.33371	-2.50219
H	-1.07044	2.44571	-2.97362
H	-2.57183	3.10745	-2.26587
H	-1.49160	4.15144	-3.23975
C	-1.57255	-2.39487	2.30591
H	-2.55246	-2.20074	2.77784
H	-1.11638	-3.26381	2.80806
H	-0.94159	-1.50894	2.46411
C	-2.35477	-1.78675	-2.78735
H	-1.76423	-2.51345	-3.37977
H	-3.37661	-1.76243	-3.20194
H	-1.91032	-0.79019	-2.92325

I5_Co_L2

Co	0.73685	0.54962	-0.03834
H	-1.46431	-1.20986	-1.34648
P	1.30021	-1.50004	0.19001
O	-0.20432	-2.11471	0.96464
C	-1.12014	-1.23353	1.27996
N	-2.24367	-1.62448	1.86758
C	-3.10189	-0.64192	2.11592
N	-2.94234	0.65414	1.86726
C	-1.78684	0.96596	1.29783
N	-0.86054	0.04653	0.98916
O	-1.49828	2.21740	1.04502
P	0.06398	2.53556	0.25675
N	0.71271	3.66049	1.31828
C	0.69948	5.01545	0.79553
H	1.69888	5.27460	0.39164
H	0.46176	5.73537	1.59618
C	-0.34785	5.02281	-0.30245
H	-1.35634	5.23322	0.10605
H	-0.13282	5.77629	-1.07576
N	-0.28882	3.69414	-0.88838
N	2.41919	-2.14444	1.26056
C	2.64667	-3.55334	0.97484
H	1.90998	-4.17850	1.51933
H	3.65340	-3.84302	1.31625
C	2.49262	-3.71259	-0.53056
H	3.46537	-3.59222	-1.04398
H	2.09034	-4.70493	-0.79448
N	1.59484	-2.65581	-0.96629
C	-5.87231	-3.05539	0.67501
C	-6.33975	-1.75805	0.87276
C	-4.85746	-3.29660	-0.24914
H	-7.13631	-1.56660	1.59634
H	-4.48434	-4.31202	-0.40033
C	-5.78324	-0.70292	0.15266
C	-4.31863	-2.24023	-0.97669
H	-6.14032	0.31699	0.33118
H	-3.52682	-2.44589	-1.70595
C	-4.76503	-0.92405	-0.78402
H	-6.30004	-3.88390	1.24541
Si	-3.99196	0.49264	-1.73556
H	-4.16308	0.25488	-3.20216
H	-4.62806	1.76907	-1.30988

O	-2.36780	0.63809	-1.41199
C	-1.34442	-0.24353	-1.90552
H	-1.58687	-0.46849	-2.97378
O	-0.13310	0.30202	-1.80338
C	4.48792	-0.79971	-1.63851
C	4.09864	0.15680	-0.69136
C	5.47426	-1.74300	-1.35453
H	5.76347	-2.47704	-2.11230
C	4.74980	0.14493	0.55044
C	6.09865	-1.74316	-0.10887
H	4.46793	0.88206	1.31233
H	6.87582	-2.47829	0.11654
C	5.73766	-0.79032	0.84330
H	6.23183	-0.77770	1.81882
H	4.00605	-0.80785	-2.62283
Si	2.63290	1.30752	-1.01930
H	1.56218	0.76852	1.18421
H	3.04444	2.66700	-0.51287
H	2.47721	1.41230	-2.50728
H	-4.04944	-0.93456	2.58291
C	-1.02644	3.43747	-2.10484
H	-0.71013	4.15984	-2.87269
H	-0.80842	2.42130	-2.45422
H	-2.11667	3.53566	-1.95337
C	1.63399	3.36999	2.38326
H	1.59027	2.30712	2.65241
H	2.67474	3.60799	2.09341
H	1.38191	3.95879	3.28004
C	1.35705	-2.48940	-2.38094
H	2.30820	-2.57562	-2.93438
H	0.66790	-3.25952	-2.76516
H	0.92948	-1.49506	-2.57321
C	2.44216	-1.75987	2.64778
H	1.69396	-2.31675	3.24395
H	3.44035	-1.95447	3.06978
H	2.23641	-0.68476	2.74392

I5_Co_L5

Co	-1.147454000	0.623837000	0.066176000
H	0.833869000	-1.105100000	1.734071000
P	-1.165598000	-1.462890000	-0.426331000
N	0.570642000	-1.651839000	-0.876756000
C	1.289814000	-0.520023000	-1.012251000
N	2.560925000	-0.561407000	-1.448333000
C	3.149159000	0.614765000	-1.490555000
N	2.657598000	1.795046000	-1.162786000
C	1.380473000	1.764640000	-0.762124000
N	0.672609000	0.627401000	-0.701543000
N	0.735880000	2.901339000	-0.421330000
P	-0.950093000	2.740590000	0.135018000
N	-1.768050000	3.871292000	-0.831718000
C	-2.445850000	4.889452000	-0.049124000
H	-3.489972000	4.587943000	0.168492000
H	-2.481795000	5.837615000	-0.611522000
C	-1.643891000	5.024310000	1.233323000
H	-0.831877000	5.773175000	1.125810000
H	-2.274813000	5.349337000	2.076664000
N	-1.109605000	3.705164000	1.501649000
N	-1.958981000	-2.168115000	-1.737040000
C	-2.245159000	-3.576550000	-1.563504000
H	-1.484401000	-4.199719000	-2.076516000
H	-3.221592000	-3.818048000	-2.018813000
C	-2.261980000	-3.836637000	-0.061099000
H	-3.297373000	-3.824527000	0.327146000
H	-1.828854000	-4.823209000	0.180297000
N	-1.504228000	-2.769597000	0.573416000
C	1.158704000	-2.943883000	-1.157488000
H	1.075466000	-3.208012000	-2.223991000
H	0.640228000	-3.700384000	-0.553268000
H	2.219864000	-2.945836000	-0.879043000
C	1.418643000	4.176934000	-0.416062000
H	1.876207000	4.381174000	0.564676000
H	0.694540000	4.967693000	-0.651943000
H	2.209942000	4.183328000	-1.174646000
C	4.155540000	-4.126709000	0.833393000
C	4.853163000	-3.034902000	0.324784000
C	3.218941000	-3.942420000	1.850690000
H	5.586461000	-3.172929000	-0.473204000
H	2.672272000	-4.797967000	2.255474000
C	4.610123000	-1.760119000	0.832882000
C	2.986599000	-2.666361000	2.354111000
H	5.168288000	-0.910817000	0.426748000
H	2.257815000	-2.538961000	3.162648000
C	3.674506000	-1.548484000	1.852915000
H	4.344375000	-5.128693000	0.439027000
Si	3.305003000	0.181095000	2.487040000
H	3.319484000	0.153367000	3.981491000
H	4.302018000	1.138785000	1.940659000
O	1.805059000	0.692529000	1.964241000
C	0.650537000	-0.130867000	2.254789000
H	0.681049000	-0.348911000	3.354051000
O	-0.498767000	0.452086000	1.931334000
C	-4.937147000	-1.547470000	1.157045000
C	-4.423553000	-0.614878000	0.245824000
C	-5.736096000	-2.611684000	0.738256000
H	-6.128498000	-3.322120000	1.471125000
C	-4.749286000	-0.786309000	-1.108425000
C	-6.041485000	-2.762459000	-0.612375000
H	-4.361436000	-0.074816000	-1.847128000
H	-6.670924000	-3.592235000	-0.945000000
C	-5.548937000	-1.840890000	-1.537166000
H	-5.792841000	-1.947151000	-2.598234000
H	-4.712390000	-1.434445000	2.223559000
Si	-3.276900000	0.790270000	0.804978000
H	-1.813360000	0.812533000	-1.258928000
H	-3.950599000	2.057258000	0.336066000
H	-3.347078000	0.805748000	2.304324000
C	-0.385066000	3.494042000	2.731959000
H	-1.038233000	3.708472000	3.592599000
H	-0.067702000	2.446418000	2.792741000
H	0.501218000	4.153223000	2.799648000
C	-2.358560000	3.508219000	-2.094123000
H	-1.720327000	2.785723000	-2.620376000
H	-3.364302000	3.060798000	-1.976022000
H	-2.449202000	4.402991000	-2.729629000
C	-1.587050000	-2.658675000	2.013393000
H	-2.603197000	-2.925829000	2.350725000
H	-0.868705000	-3.329301000	2.515089000
H	-1.390900000	-1.624158000	2.327934000
C	-1.821048000	-1.676660000	-3.077699000
H	-0.993652000	-2.171375000	-3.623875000
H	-2.752558000	-1.849055000	-3.642017000
H	-1.628208000	-0.594349000	-3.065855000
C	4.596016000	0.656641000	-1.978971000
F	5.390730000	1.139015000	-1.021895000
F	5.051550000	-0.538965000	-2.321538000
F	4.703755000	1.457074000	-3.038153000

I5_Co_L6

Co	-1.09102	0.58761	-0.01716
H	1.36889	-0.80273	1.31832
P	-1.31065	-1.55221	-0.13475
N	0.21754	-1.96973	-0.92404
C	1.02015	-0.93723	-1.29526
N	2.19108	-1.18298	-1.89090
C	2.90090	-0.10048	-2.17092

N	2.57928	1.16016	-1.91899
C	1.38462	1.32498	-1.34051
N	0.57703	0.29868	-1.03227
N	0.93073	2.57045	-1.05350
P	-0.60317	2.66266	-0.18760
C	0.71856	-3.32412	-1.05331
H	1.14917	-3.48128	-2.05014
H	-0.10326	-4.03587	-0.90963
H	1.50437	-3.52854	-0.30746
C	1.82727	3.70052	-1.18362
H	2.54401	3.73497	-0.34615
H	1.24642	4.63140	-1.19195
H	2.39227	3.63015	-2.12055
C	5.40400	-2.84945	-0.27517
C	5.78096	-1.58633	-0.72227
C	4.51650	-2.97164	0.79282
H	6.48372	-1.48675	-1.55367
H	4.22719	-3.96191	1.15403
C	5.25741	-0.44851	-0.11068
C	4.00767	-1.83048	1.40539
H	5.55128	0.54108	-0.47844
H	3.32637	-1.94246	2.25653
C	4.35996	-0.54646	0.96025
H	5.80830	-3.74376	-0.75613
Si	3.64338	0.98382	1.77863
H	3.86145	0.87427	3.25351
H	4.30805	2.19385	1.22052
O	2.01536	1.14680	1.47655
C	1.07561	0.11420	1.89984
H	1.31183	-0.10262	2.97283
O	-0.18305	0.48222	1.75183
C	-4.97369	-1.11630	1.31622
C	-4.49709	-0.09684	0.47944
C	-5.98092	-1.98799	0.90201
H	-6.33406	-2.77427	1.57460
C	-5.08254	0.02491	-0.79009
C	-6.54297	-1.84888	-0.36477
H	-4.73070	0.80998	-1.46933
H	-7.33462	-2.52751	-0.69294
C	-6.09425	-0.83367	-1.20998
H	-6.53523	-0.71530	-2.20344
H	-4.55217	-1.22571	2.32236
Si	-3.05174	1.01520	1.00895
H	-1.93727	0.72295	-1.24444
H	-3.56029	2.42120	0.78442
H	-2.95437	0.84494	2.49857
H	3.86203	-0.26760	-2.67195
C	-2.52237	-2.43156	-1.17219
H	-2.57056	-1.94597	-2.15588
H	-2.26847	-3.49489	-1.28981
H	-3.50935	-2.35209	-0.69386
C	-1.56686	3.81562	-1.21749
H	-1.74512	3.35504	-2.19814
H	-2.53628	3.97516	-0.72254
H	-1.07001	4.78864	-1.34168
C	-1.28839	-2.54556	1.39267
H	-2.27611	-2.42811	1.86384
H	-1.11244	-3.61453	1.20667
H	-0.53449	-2.13605	2.07668
C	-0.21452	3.63297	1.29851
H	-1.13741	3.70139	1.89420
H	0.53190	3.07306	1.87596
H	0.13862	4.64703	1.06309

I5_Co_L7

Co	-0.78042	0.69299	-0.49898
H	0.67028	-1.04223	1.47896
P	-1.15610	-1.33190	-0.92048
N	0.42126	-1.89697	-1.42907
C	1.42339	-0.97295	-1.42741
N	2.65347	-1.29228	-1.83102
C	3.52422	-0.29585	-1.78139
N	3.28573	0.96240	-1.43520
C	2.02556	1.21040	-1.07930
N	1.08670	0.25462	-1.01116
N	1.61648	2.47633	-0.79384
P	-0.06379	2.65865	-0.38500
O	-0.50275	3.90795	-1.36857
C	-1.02746	4.99760	-0.62561
H	-2.11994	4.88320	-0.52805
H	-0.81394	5.92770	-1.16940
C	-0.33696	4.94054	0.73279
H	0.60465	5.51558	0.73575
H	-0.97809	5.31165	1.54384
O	-0.05476	3.57237	0.97268
O	-2.17978	-1.87147	-2.08494
C	-2.81386	-3.09945	-1.75141
H	-2.30763	-3.92487	-2.27901
H	-3.85860	-3.05210	-2.08741
C	-2.70655	-3.24046	-0.23477
H	-3.58237	-2.81700	0.28064
H	-2.58049	-4.28736	0.07464
O	-1.54160	-2.52172	0.14541
C	0.65627	-3.27052	-1.82622
H	0.18990	-3.48211	-2.80019
H	0.24218	-3.95061	-1.06874
H	1.73552	-3.43975	-1.90861
C	2.55376	3.57403	-0.67937
H	2.76598	3.79085	0.37889
H	2.13384	4.46839	-1.15984
H	3.48778	3.30530	-1.18403
C	4.92769	-3.33928	0.21577
C	5.57820	-2.10885	0.22792
C	3.71464	-3.48832	0.88565
H	6.53416	-1.98868	-0.28878
H	3.20500	-4.45518	0.88773
C	5.00589	-1.02744	0.89597
C	3.15909	-2.40889	1.56601
H	5.51949	-0.05970	0.88474
H	2.21657	-2.55096	2.10571
C	3.78811	-1.15341	1.57760
H	5.36934	-4.18746	-0.31348
Si	3.03953	0.31241	2.48708
H	2.85442	-0.06189	3.92256
H	3.96624	1.46881	2.34255
O	1.57929	0.77281	1.84543
C	0.40462	-0.06708	1.96508
H	0.26515	-0.26985	3.05386
O	-0.67294	0.50693	1.44528
C	-3.74958	-0.49148	1.85971
C	-4.02051	0.01321	0.57704
C	-4.57128	-1.45515	2.43780
H	-4.34723	-1.83055	3.43974
C	-5.13140	-0.49761	-0.11066
C	-5.67835	-1.94301	1.74228
H	-5.36376	-0.11988	-1.11254
H	-6.32628	-2.69539	2.19984
C	-5.95585	-1.46545	0.46304
H	-6.82278	-1.84150	-0.08731
H	-2.86720	-0.12523	2.39295
Si	-2.92321	1.34967	-0.19362
H	-1.09274	0.92596	-1.94204
H	-3.64490	1.78367	-1.43658
H	-2.94835	2.52569	0.74605

H	4.55201	-0.53271	-2.07986

I5_Co_L8

Co	-1.07020	0.56893	-0.00739
H	1.38427	-0.62826	1.70494
C	0.33884	-1.98338	-0.44893
C	1.09813	-0.95710	-1.23340
N	2.21344	-1.22361	-1.89860
C	2.84630	-0.14297	-2.35644
N	2.50371	1.12995	-2.15208
C	1.36538	1.29221	-1.48979
N	0.63198	0.26888	-1.09756
C	0.87083	2.60831	-0.99290
C	5.35592	-2.62626	-0.46373
C	5.70893	-1.35252	-0.89996
C	4.52115	-2.77505	0.64268
H	6.37394	-1.23271	-1.75944
H	4.25174	-3.77411	0.99425
C	5.21018	-0.22981	-0.24127
C	4.04476	-1.64916	1.30797
H	5.48326	0.76825	-0.60217
H	3.41359	-1.78051	2.19447
C	4.36797	-0.35439	0.87151
H	5.73705	-3.50854	-0.98388
Si	3.66338	1.15944	1.72982
H	3.91068	1.04378	3.19853
H	4.30677	2.37183	1.15112
O	2.03204	1.31239	1.44835
C	1.06014	0.39441	2.04980
H	1.25640	0.42838	3.15039
O	-0.18169	0.70371	1.75493
C	-4.97211	-1.09618	1.28509
C	-4.49153	-0.10478	0.41797
C	-5.92810	-2.02416	0.87282
H	-6.28036	-2.79189	1.56706
C	-5.03504	-0.05965	-0.87567
C	-6.44172	-1.96546	-0.42019
H	-4.68018	0.70335	-1.57871
H	-7.19305	-2.68925	-0.74704
C	-5.99772	-0.97267	-1.29431
H	-6.40374	-0.91527	-2.30780
H	-4.58803	-1.14445	2.31082
Si	-3.06939	1.05947	0.89582
H	-1.89585	0.50438	-1.25049
H	-3.55785	2.42273	0.47282
H	-3.04474	1.06763	2.39962
H	3.74794	-0.31949	-2.95290
H	0.30855	-2.95108	-0.97862
H	1.02076	3.40935	-1.73633
H	0.94266	-2.15043	0.45916
H	1.52113	2.83002	-0.13214
N	-1.02600	-1.54029	-0.05717
N	-0.54009	2.56663	-0.52101
C	-1.39315	3.03719	-1.62082
H	-1.14205	4.08419	-1.87371
H	-1.24133	2.40595	-2.50570
H	-2.44713	2.98147	-1.32881
C	-0.66427	3.48311	0.62381
H	-1.72100	3.58373	0.89660
H	-0.11248	3.06359	1.47301
H	-0.27474	4.48287	0.35773
C	-1.36385	-2.11385	1.25280
H	-1.18316	-3.20472	1.25779
H	-0.77283	-1.61819	2.03178
H	-2.42743	-1.94008	1.45418
C	-1.96582	-2.05729	-1.06492
H	-1.67900	-1.69403	-2.05983
H	-1.94695	-3.16300	-1.06251

H	-2.98263	-1.71432	-0.84761

I5_Co_L10

Co	0.67234	1.06781	0.70761
H	0.21338	-0.70902	-2.73604
C	-0.73661	3.07733	-0.74135
C	-1.86574	3.77361	-1.17374
C	-3.12745	3.39246	-0.69950
C	-3.26610	2.32448	0.19483
C	-2.12419	1.64690	0.62283
C	-0.87079	2.02914	0.16098
H	0.61561	1.84574	1.96610
Si	2.51116	0.20115	1.72490
H	3.45469	1.19168	2.39576
H	2.26841	-0.77419	2.86861
C	4.24480	-2.45851	-1.05041
C	5.61356	-2.20174	-0.96455
C	3.34273	-1.74749	-0.26181
H	6.32057	-2.75602	-1.59022
H	2.26912	-1.92840	-0.37466
C	6.06969	-1.22509	-0.08160
C	3.77911	-0.77021	0.65029
H	7.14105	-1.00992	-0.00740
C	5.15958	-0.52561	0.71222
H	5.53458	0.23218	1.41050
H	3.87380	-3.21293	-1.75142
C	0.04052	-0.52597	-1.64425
O	-0.04366	-1.89404	-1.05799
O	0.97814	0.18998	-1.09102
H	-4.01674	3.93382	-1.03611
H	-4.26183	2.03532	0.55000
H	-1.78150	4.60651	-1.88199
Si	-1.33618	-2.85345	-1.38547
H	-1.58361	-2.93478	-2.86348
H	-1.05679	-4.21190	-0.83293
C	-2.95859	-2.27761	-0.60841
C	-3.66190	-1.17468	-1.11561
C	-3.50816	-2.94774	0.49377
C	-4.85922	-0.75427	-0.54335
C	-4.70779	-2.53668	1.07264
C	-5.38475	-1.43511	0.55325
H	-3.25976	-0.62109	-1.97056
H	-2.98375	-3.81536	0.91051
H	-5.37702	0.11794	-0.95050
H	-5.11375	-3.07489	1.93359
H	-6.32394	-1.10449	1.00534
H	-0.99707	-0.10171	-1.53296
C	0.67471	3.28155	-1.19447
H	0.84308	2.77257	-2.16425
H	0.95690	4.34590	-1.31619
C	-2.05352	0.49140	1.56993
H	-2.03119	0.83410	2.62614
H	-2.88881	-0.22530	1.45842
O	-0.82432	-0.19190	1.29842
O	1.54768	2.68884	-0.22523
C	2.85551	2.50924	-0.72918
H	3.20256	3.44669	-1.19851
H	3.51205	2.26345	0.11388
H	2.86673	1.68467	-1.46081
C	-0.59811	-1.29715	2.13506
H	-1.49994	-1.93421	2.15562
H	0.24389	-1.86157	1.71828
H	-0.34989	-0.96610	3.15886

I5_Fe_L1

Fe	-0.72967	0.56250	0.00600
H	1.52848	-1.07574	1.43618
P	-1.19198	-1.47491	-0.25792
N	0.39288	-2.08428	-0.91198
C	1.29848	-1.13172	-1.22856
N	2.45406	-1.46576	-1.82902
C	3.26284	-0.44418	-2.06038
N	3.04395	0.83550	-1.80286
C	1.84990	1.09922	-1.24236
N	0.96746	0.13442	-0.91896
N	1.46358	2.37075	-1.00627
P	-0.17105	2.56982	-0.26559
N	-0.83948	3.74248	-1.34392
C	-1.18504	4.98440	-0.69045
H	-2.23335	4.96148	-0.32285
H	-1.10068	5.82895	-1.39975
C	-0.21978	5.12570	0.47480
H	0.70801	5.65680	0.16550
H	-0.66433	5.71227	1.29856
N	0.03498	3.78045	0.91283
N	-2.29245	-2.14257	-1.38714
C	-2.79754	-3.44446	-1.03687
H	-2.21200	-4.25416	-1.52709
H	-3.84387	-3.55242	-1.37866
C	-2.70775	-3.53200	0.47835
H	-3.64825	-3.16644	0.93925
H	-2.55853	-4.57542	0.81454
N	-1.59956	-2.69055	0.88230
C	0.67419	-3.47204	-1.17338
H	0.47638	-3.74838	-2.22411
H	0.03972	-4.08265	-0.51596
H	1.72880	-3.69660	-0.96056
C	2.36380	3.47811	-1.18663
H	2.82295	3.77681	-0.22783
H	1.80765	4.33557	-1.59411
H	3.16512	3.20541	-1.88444
C	5.56417	-3.36840	-0.32181
C	6.13364	-2.12942	-0.60222
C	4.53924	-3.46273	0.61789
H	6.93496	-2.04868	-1.34213
H	4.08480	-4.43236	0.83791
C	5.66824	-0.98850	0.04958
C	4.08926	-2.32026	1.27262
H	6.10441	-0.01407	-0.19745
H	3.28401	-2.41183	2.00955
C	4.64084	-1.05901	0.99825
H	5.91615	-4.26430	-0.84079
Si	4.01170	0.48704	1.87150
H	4.25072	0.29474	3.34088
H	4.80398	1.64977	1.37543
O	2.41913	0.78825	1.58855
C	1.34596	-0.13298	2.02149
H	1.58560	-0.36992	3.09488
O	0.15624	0.38803	1.87573
C	-4.82938	-0.64850	1.78458
C	-4.21858	0.03422	0.72174
C	-5.85669	-1.57119	1.57937
H	-6.30702	-2.08713	2.43356
C	-4.69989	-0.25078	-0.56801
C	-6.31259	-1.83244	0.28873
H	-4.23557	0.25011	-1.42619
H	-7.11844	-2.55351	0.12056
C	-5.73064	-1.15965	-0.78781
H	-6.07996	-1.35432	-1.80745
H	-4.48650	-0.45014	2.80715
Si	-2.71126	1.20664	0.96790
H	-1.49038	0.72541	-1.33461
H	-3.29358	2.52567	0.46684
H	-2.72729	1.40298	2.47422
H	4.22478	-0.68717	-2.53186
C	0.63044	3.53991	2.19798
H	0.07549	4.08941	2.97881
H	0.58034	2.46485	2.42294
H	1.69191	3.85859	2.24109
C	-1.68364	3.35164	-2.43876
H	-1.34050	2.39797	-2.86098
H	-2.74104	3.22053	-2.13130
H	-1.64694	4.11791	-3.23311
C	-1.50921	-2.38964	2.29319
H	-2.51175	-2.18589	2.71605
H	-1.06650	-3.23182	2.85385
H	-0.89682	-1.48807	2.44823
C	-2.25120	-1.80303	-2.77521
H	-1.61321	-2.49714	-3.36293
H	-3.26728	-1.83023	-3.20876
H	-1.85536	-0.78366	-2.89425

I5_Fe_L2

Fe	0.73506	0.57672	-0.12298
H	-1.50465	-1.11638	-1.41763
P	1.18498	-1.45122	0.11446
O	-0.35886	-2.02846	0.94852
C	-1.20169	-1.08580	1.26188
N	-2.31019	-1.39617	1.93777
C	-3.09337	-0.36225	2.21029
N	-2.87407	0.91642	1.93420
C	-1.73274	1.15559	1.28557
N	-0.89787	0.17259	0.89780
O	-1.38204	2.38436	1.04081
P	0.23370	2.57630	0.19983
N	0.96237	3.68531	1.25796
C	0.88034	5.05947	0.81575
H	1.84057	5.36497	0.34775
H	0.69437	5.73843	1.66791
C	-0.24564	5.10213	-0.19961
H	-1.22744	5.24804	0.30079
H	-0.11637	5.92080	-0.92790
N	-0.18741	3.82324	-0.86881
N	2.26520	-2.20390	1.18599
C	2.38708	-3.61974	0.90785
H	1.59701	-4.19104	1.44177
H	3.36537	-3.99039	1.25968
C	2.23777	-3.76835	-0.59777
H	3.22383	-3.68167	-1.10011
H	1.80900	-4.75095	-0.86724
N	1.38367	-2.68237	-1.02235
C	-5.67149	-3.12143	0.87499
C	-6.23214	-1.85330	1.00610
C	-4.65244	-3.33771	-0.05051
H	-7.02599	-1.67726	1.73753
H	-4.19534	-4.32586	-0.14169
C	-5.76544	-0.80376	0.21659
C	-4.20450	-2.28861	-0.84666
H	-6.19126	0.19733	0.34695
H	-3.39720	-2.47138	-1.56461
C	-4.74683	-1.00046	-0.72425
H	-6.02156	-3.94347	1.50559
Si	-4.08914	0.41959	-1.76686
H	-4.33574	0.08365	-3.20847
H	-4.84042	1.65198	-1.39048
O	-2.48531	0.70000	-1.52512
C	-1.40527	-0.16767	-2.01403
H	-1.68648	-0.42452	-3.07110
O	-0.23129	0.41255	-1.93555
C	4.56730	-1.05976	-1.52937
C	4.15961	-0.02386	-0.67528
C	5.48981	-2.02608	-1.12906

H	5.77961	-2.82435	-1.82058
C	4.74565	0.00936	0.60014
C	6.04697	-1.97416	0.14772
H	4.44783	0.79958	1.30011
H	6.77234	-2.72862	0.46688
C	5.67310	-0.94340	1.01088
H	6.10589	-0.88795	2.01503
H	4.13941	-1.11601	-2.53779
Si	2.71781	1.15616	-1.13395
H	1.54751	0.71301	1.18681
H	3.29285	2.51734	-0.76256
H	2.76412	1.15918	-2.64790
H	-4.02831	-0.59163	2.73823
C	-1.12933	3.58472	-1.93478
H	-0.96642	4.31921	-2.74099
H	-0.98100	2.57072	-2.32970
H	-2.17712	3.66980	-1.58442
C	2.01372	3.37050	2.18219
H	2.04457	2.28801	2.36097
H	3.00207	3.68049	1.79011
H	1.85018	3.88175	3.14769
C	1.21870	-2.45340	-2.43526
H	2.18963	-2.54539	-2.95737
H	0.52099	-3.18174	-2.88586
H	0.83181	-1.43655	-2.60486
C	2.31717	-1.83014	2.57129
H	1.53321	-2.33940	3.16796
H	3.30220	-2.09164	2.99260
H	2.17783	-0.74441	2.66616

I5_Fe_L3

Fe	0.53484	0.51875	-0.07953
H	-1.60971	-0.97850	-1.74862
P	0.98168	-1.54782	0.13292
C	-0.69294	-2.24880	0.69357
C	-1.49644	-1.14745	1.27985
N	-2.42435	-1.41112	2.19808
C	-3.11269	-0.36379	2.62893
N	-2.96971	0.88812	2.21510
C	-2.00233	1.09359	1.32077
N	-1.21075	0.10190	0.85326
C	-1.73382	2.45978	0.81651
P	0.08843	2.55702	0.29560
N	0.79587	3.58099	1.50261
C	1.01157	4.93270	1.03212
H	2.06945	5.06594	0.71847
H	0.80846	5.66990	1.83223
C	0.09552	5.12600	-0.16219
H	-0.93069	5.41832	0.16489
H	0.46048	5.92391	-0.83280
N	0.10602	3.85575	-0.83738
N	2.07771	-2.20813	1.29608
C	2.44728	-3.55770	0.95169
H	1.69324	-4.29623	1.31423
H	3.41184	-3.82103	1.42221
C	2.54022	-3.58163	-0.56213
H	3.53308	-3.20415	-0.88533
H	2.42854	-4.60818	-0.95925
N	1.49036	-2.70941	-1.04563
C	-5.21040	-3.22365	0.69151
C	-5.65972	-1.95178	1.03309
C	-4.43487	-3.40106	-0.45302
H	-6.26261	-1.80682	1.93390
H	-4.07541	-4.39756	-0.72274
C	-5.32014	-0.85792	0.23927
C	-4.11611	-2.30614	-1.25054
H	-5.66017	0.14190	0.53109
H	-3.51554	-2.46019	-2.15446
C	-4.54390	-1.01124	-0.91705
H	-5.45737	-4.08038	1.32404
Si	-4.07263	0.46646	-1.98277
H	-4.39416	0.10687	-3.40320
H	-4.89581	1.63040	-1.53940
O	-2.49039	0.89741	-1.83483
C	-1.38487	0.00101	-2.25953
H	-1.56099	-0.15754	-3.35704
O	-0.21184	0.50245	-1.99630
C	4.80440	-0.62059	-1.59444
C	4.10097	0.00170	-0.55249
C	5.84286	-1.52175	-1.35258
H	6.36665	-1.99074	-2.19177
C	4.50329	-0.31697	0.75559
C	6.21661	-1.81967	-0.04383
H	3.96856	0.13780	1.59802
H	7.03025	-2.52452	0.15327
C	5.54342	-1.20445	1.01350
H	5.82785	-1.42803	2.04712
H	4.52883	-0.39014	-2.63053
Si	2.59947	1.16619	-0.85975
H	1.19382	0.55123	1.32211
H	3.13743	2.46498	-0.27226
H	2.70332	1.41045	-2.35352
H	-3.87436	-0.55065	3.39707
C	-0.61449	3.74202	-2.07584
H	-0.18602	4.43159	-2.82307
H	-0.53414	2.71129	-2.45143
H	-1.69365	3.98913	-1.96730
C	1.75214	3.11427	2.46809
H	1.58381	2.05220	2.68836
H	2.79469	3.22046	2.10477
H	1.65708	3.68537	3.40956
C	1.59848	-2.28915	-2.42369
H	2.63934	-2.00557	-2.67666
H	1.28898	-3.09751	-3.10971
H	0.96458	-1.40686	-2.60144
C	1.92242	-1.93271	2.69137
H	1.15736	-2.57808	3.17799
H	2.88003	-2.09591	3.21737
H	1.62928	-0.88206	2.83341
H	-0.67346	-3.12405	1.36027
H	-2.09333	3.22205	1.52048
H	-1.17312	-2.56415	-0.24929
H	-2.27651	2.55897	-0.14278

I5_Fe_L6

Fe	-1.10369	0.58235	-0.01501
H	1.35666	-0.77920	1.38336
P	-1.37437	-1.52174	-0.05996
N	0.14868	-2.04688	-0.85578
C	0.99198	-1.04580	-1.21887
N	2.16407	-1.34214	-1.80272
C	2.90655	-0.29091	-2.10988
N	2.61254	0.98515	-1.91331
C	1.41831	1.20277	-1.33979
N	0.58670	0.21089	-0.96571
N	0.99421	2.47223	-1.12834
P	-0.56195	2.61410	-0.26042
C	0.59115	-3.41484	-0.96667
H	1.11677	-3.57796	-1.91695
H	-0.27671	-4.08615	-0.92503
H	1.28245	-3.68845	-0.14927
C	1.90668	3.56698	-1.34405
H	2.65416	3.63627	-0.53384
H	1.34443	4.50974	-1.37974
H	2.44607	3.44223	-2.29252
C	5.54413	-2.69470	-0.41153

C	5.98276	-1.40073	-0.67919
C	4.55223	-2.90558	0.54436
H	6.75746	-1.22858	-1.43177
H	4.19831	-3.91881	0.75186
C	5.41912	-0.32222	0.00084
C	4.00641	-1.82431	1.22925
H	5.75185	0.69422	-0.23692
H	3.23033	-2.00623	1.98109
C	4.42414	-0.51001	0.96815
H	5.97195	-3.54272	-0.95347
Si	3.66612	0.95176	1.88491
H	3.89526	0.71704	3.34953
H	4.39382	2.18100	1.45125
O	2.06126	1.15891	1.58218
C	1.05856	0.12299	1.99098
H	1.32125	-0.10560	3.06125
O	-0.16569	0.52334	1.83698
C	-5.12993	-0.96393	1.31319
C	-4.60941	0.01822	0.45516
C	-6.13668	-1.84165	0.90902
H	-6.51476	-2.59772	1.60477
C	-5.16270	0.08392	-0.83520
C	-6.66523	-1.75302	-0.37774
H	-4.77098	0.82777	-1.53909
H	-7.45499	-2.43800	-0.70102
C	-6.17391	-0.77919	-1.24908
H	-6.57911	-0.70024	-2.26310
H	-4.73221	-1.04064	2.33269
Si	-3.10875	1.11710	0.94989
H	-1.90415	0.67705	-1.35495
H	-3.68148	2.50237	0.64328
H	-3.21076	1.07086	2.46670
H	3.87479	-0.50198	-2.58273
C	-2.60427	-2.44930	-1.05462
H	-2.64390	-2.00774	-2.05952
H	-2.38399	-3.52587	-1.11951
H	-3.58830	-2.31638	-0.58031
C	-1.45823	3.80137	-1.33222
H	-1.63935	3.32384	-2.30456
H	-2.43131	3.99109	-0.85313
H	-0.93294	4.75961	-1.46648
C	-0.09857	3.68201	1.15553
H	-1.01682	3.85162	1.73945
H	0.60725	3.11227	1.77534
H	0.32606	4.65365	0.85966
C	-1.34107	-2.54890	1.46636
H	-2.31961	-2.42121	1.95459
H	-1.17634	-3.62126	1.27953
H	-0.57374	-2.14830	2.14226

I5_Fe_L7

Fe	-0.77990	0.74324	-0.46640
H	0.64749	-1.01337	1.52498
P	-1.13630	-1.23267	-0.95288
N	0.44825	-1.80207	-1.51938
C	1.44565	-0.88467	-1.44535
N	2.68693	-1.18813	-1.84800
C	3.56031	-0.20235	-1.73078
N	3.32046	1.03885	-1.33488
C	2.04771	1.27874	-0.99450
N	1.09921	0.32264	-0.95995
N	1.63770	2.53823	-0.70195
P	-0.08627	2.69250	-0.37995
O	-0.43310	3.97568	-1.40329
C	-1.03550	5.03286	-0.69485
H	-2.12465	4.86196	-0.61033
H	-0.86659	5.97296	-1.24132
C	-0.37652	5.02817	0.67985
H	0.55199	5.63088	0.67841
H	-1.04434	5.42199	1.46168
O	-0.07848	3.68492	0.96134
O	-2.15761	-1.77110	-2.15511
C	-2.76791	-3.01242	-1.88495
H	-2.26046	-3.81071	-2.45798
H	-3.82036	-2.96839	-2.20476
C	-2.64411	-3.23947	-0.38008
H	-3.51950	-2.84276	0.16071
H	-2.53339	-4.30767	-0.13488
O	-1.47877	-2.55792	0.01914
C	0.68421	-3.14359	-1.99202
H	0.22967	-3.29885	-2.98409
H	0.24891	-3.86604	-1.28548
H	1.76377	-3.32010	-2.06898
C	2.57163	3.62099	-0.52287
H	2.75001	3.80562	0.54940
H	2.16836	4.53535	-0.98198
H	3.52383	3.37037	-1.00494
C	4.74254	-3.50660	0.14400
C	5.48121	-2.32869	0.20685
C	3.51118	-3.58555	0.79161
H	6.44676	-2.25854	-0.30229
H	2.92399	-4.50647	0.74455
C	4.97857	-1.23024	0.90291
C	3.02583	-2.49032	1.49958
H	5.55572	-0.29879	0.92166
H	2.05857	-2.57301	2.00596
C	3.74470	-1.28562	1.56354
H	5.12446	-4.36494	-0.41567
Si	3.08059	0.21212	2.50086
H	2.96283	-0.18640	3.94337
H	4.08807	1.30388	2.35577
O	1.63790	0.76116	1.93411
C	0.39868	-0.04617	2.04018
H	0.29461	-0.26640	3.13500
O	-0.64092	0.57203	1.54779
C	-3.73359	-0.59812	1.86572
C	-4.07306	-0.03461	0.62181
C	-4.49266	-1.61941	2.43120
H	-4.20535	-2.03726	3.40104
C	-5.19509	-0.56195	-0.03608
C	-5.61141	-2.12056	1.76183
H	-5.48207	-0.14866	-1.01023
H	-6.20759	-2.92364	2.20596
C	-5.95786	-1.59054	0.52018
H	-6.83016	-1.97726	-0.01705
H	-2.83525	-0.22997	2.37405
Si	-2.97278	1.34163	-0.13856
H	-1.08430	0.93383	-1.97127
H	-3.80865	1.77462	-1.32764
H	-3.17307	2.48242	0.84608
H	4.59603	-0.43297	-2.01229

I5_Fe_L8

Fe	0.98880	0.65698	0.02770
H	-1.34841	-0.47322	-1.93933
C	-0.41511	-1.98529	0.32887
C	-1.11219	-0.99625	1.21283
N	-2.05668	-1.31286	2.08372
C	-2.65949	-0.26827	2.65837
N	-2.42265	1.02229	2.39702
C	-1.44594	1.24503	1.53081
N	-0.74258	0.26078	0.97195
C	-1.07052	2.59245	1.00392
C	-4.93821	-2.74402	0.54227
C	-5.27556	-1.47240	0.99490
C	-4.22767	-2.88994	-0.64808

H	-5.82699	-1.35209	1.93156
H	-3.95902	-3.88699	-1.00739
C	-4.88948	-0.34941	0.26568
C	-3.86160	-1.76372	-1.37938
H	-5.14291	0.64703	0.64463
H	-3.31770	-1.89179	-2.32227
C	-4.17580	-0.46947	-0.93394
H	-5.22183	-3.62579	1.12280
Si	-3.66188	1.05381	-1.91398
H	-3.96471	0.76499	-3.35492
H	-4.48776	2.19850	-1.42736
O	-2.07835	1.46058	-1.71733
C	-0.99849	0.55854	-2.24920
H	-1.13507	0.61860	-3.36201
O	0.18317	0.89177	-1.84474
C	4.92555	-0.95483	-1.31444
C	4.49559	-0.02088	-0.35711
C	5.81157	-1.98541	-1.00062
H	6.11433	-2.70096	-1.77218
C	5.03211	-0.15865	0.93549
C	6.31821	-2.10134	0.29294
H	4.71369	0.54383	1.71530
H	7.01426	-2.90706	0.54606
C	5.92721	-1.17447	1.26110
H	6.31948	-1.25220	2.28036
H	4.54430	-0.87407	-2.34032
Si	3.07756	1.23261	-0.71484
H	1.69778	0.51179	1.39865
H	3.67865	2.50005	-0.10193
H	3.29873	1.48954	-2.20199
H	-3.43007	-0.48960	3.40540
H	-0.43007	-2.99858	0.77347
H	-1.35323	3.39687	1.70838
H	-1.02209	-2.03307	-0.59343
H	-1.66513	2.71206	0.08207
N	0.95061	-1.55874	-0.02858
N	0.35469	2.66429	0.62858
C	1.30540	-2.05085	-1.35539
H	2.36705	-1.84370	-1.53929
H	1.14162	-3.14659	-1.43463
H	0.71690	-1.52131	-2.11516
C	1.87986	-2.10044	0.96131
H	1.58201	-1.77132	1.96504
H	1.87898	-3.21045	0.92183
H	2.89338	-1.73061	0.77078
C	0.52594	3.61563	-0.46780
H	1.59822	3.74531	-0.66527
H	0.05151	3.20792	-1.36960
H	0.09003	4.60316	-0.20777
C	1.12734	3.09490	1.78943
H	2.19771	3.06718	1.55309
H	0.83733	4.12560	2.08585
H	0.94628	2.41116	2.62899

I5_Ni_L1

Ni	-0.74896	0.53216	-0.04733
H	1.48928	-1.19397	1.32352
P	-1.28856	-1.58522	-0.28724
N	0.26823	-2.18372	-0.85229
C	1.20783	-1.27800	-1.23374
N	2.37699	-1.68884	-1.71912
C	3.23750	-0.71473	-1.98442
N	3.05787	0.58447	-1.78243
C	1.85975	0.91690	-1.31025
N	0.88712	0.01663	-1.09026
N	1.55237	2.21346	-1.04822
P	0.02349	2.56350	-0.28182
N	-0.66613	3.70062	-1.30935
C	-1.04567	4.92679	-0.62546
H	-2.10698	4.89820	-0.31287
H	-0.92076	5.78557	-1.30373
C	-0.11968	5.01992	0.57752
H	0.80996	5.56768	0.33264
H	-0.59634	5.54175	1.42077
N	0.17196	3.64550	0.97067
N	-2.42175	-2.10242	-1.40526
C	-3.09205	-3.33206	-1.01450
H	-2.61413	-4.20722	-1.49623
H	-4.14261	-3.30565	-1.34744
C	-2.99551	-3.40059	0.50370
H	-3.89367	-2.96793	0.98027
H	-2.89498	-4.43899	0.85874
N	-1.81903	-2.62850	0.89670
C	0.53046	-3.60433	-1.01221
H	0.47385	-3.90667	-2.06848
H	-0.20670	-4.16567	-0.42636
H	1.53441	-3.84276	-0.63864
C	2.54337	3.26662	-1.17984
H	3.03923	3.45950	-0.21602
H	2.05359	4.18348	-1.53238
H	3.29986	2.96195	-1.91061
C	5.79782	-3.21171	-0.23697
C	6.26741	-1.93632	-0.54058
C	4.78270	-3.37440	0.70450
H	7.07313	-1.80768	-1.26737
H	4.42456	-4.37579	0.95470
C	5.70959	-0.82395	0.08680
C	4.24079	-2.26088	1.33849
H	6.07923	0.17528	-0.16749
H	3.46334	-2.40807	2.09698
C	4.68655	-0.96462	1.03405
H	6.23255	-4.08559	-0.72803
Si	3.92167	0.52641	1.86113
H	3.98185	0.38403	3.34470
H	4.58409	1.77038	1.38604
O	2.30601	0.69026	1.43360
C	1.30885	-0.23209	1.86701
H	1.48128	-0.44631	2.94949
O	0.06880	0.25810	1.70200
C	-4.70972	-0.38568	1.79990
C	-4.16651	0.18930	0.64284
C	-5.82350	-1.22083	1.72815
H	-6.24346	-1.65054	2.64039
C	-4.76590	-0.10111	-0.59191
C	-6.40509	-1.49655	0.49312
H	-4.36367	0.34131	-1.51055
H	-7.28219	-2.14540	0.43512
C	-5.87607	-0.93446	-0.66953
H	-6.33984	-1.14040	-1.63749
H	-4.26528	-0.16565	2.77644
Si	-2.71720	1.35837	0.77176
H	-1.84297	0.87425	-0.98883
H	-3.04893	2.68072	0.16425
H	-2.31645	1.53492	2.19698
H	4.19935	-1.01730	-2.41224
C	0.78712	3.40286	2.25902
H	0.20418	3.91517	3.03872
H	0.79188	2.32958	2.47136
H	1.82370	3.78170	2.29370
C	-1.33632	3.33321	-2.53019
H	-0.85626	2.44984	-2.97626
H	-2.40932	3.11046	-2.37438
H	-1.26029	4.15349	-3.25897
C	-1.58766	-2.45374	2.31632
H	-2.51708	-2.13455	2.81902
H	-1.25646	-3.39927	2.77175
H	-0.83094	-1.67848	2.48544
C	-2.31977	-1.79079	-2.80670
H	-1.74988	-2.55591	-3.36629

H	-3.32498	-1.72080	-3.25122
H	-1.82061	-0.82030	-2.94708

I5_Ni_L3

Ni	0.57627	0.49343	-0.03062
H	-1.55480	-1.00152	-1.65549
P	1.02879	-1.66602	0.19534
C	-0.66590	-2.30499	0.67679
C	-1.51600	-1.23519	1.26707
N	-2.54075	-1.54635	2.04661
C	-3.28778	-0.52163	2.43956
N	-3.10554	0.75081	2.09789
C	-2.04720	0.99226	1.33921
N	-1.20445	0.02698	0.94165
C	-1.76578	2.37894	0.87880
P	0.00459	2.58548	0.32667
N	0.71276	3.53721	1.53028
C	1.26874	4.77414	0.99565
H	2.34420	4.66342	0.76006
H	1.16860	5.57854	1.74107
C	0.46204	5.06710	-0.25755
H	-0.46651	5.61930	-0.01213
H	1.02872	5.67150	-0.98159
N	0.16093	3.76643	-0.84497
N	2.09346	-2.21021	1.38616
C	2.59151	-3.53521	1.04274
H	1.90293	-4.32533	1.40799
H	3.56914	-3.69923	1.52318
C	2.69606	-3.55896	-0.47239
H	3.66857	-3.15683	-0.81053
H	2.59597	-4.58160	-0.86969
N	1.61197	-2.71823	-0.97192
C	-5.45495	-3.03819	0.51809
C	-5.88977	-1.74992	0.81725
C	-4.57294	-3.24668	-0.54100
H	-6.59861	-1.58642	1.63295
H	-4.24665	-4.25927	-0.78978
C	-5.42736	-0.66880	0.06867
C	-4.13251	-2.16507	-1.29853
H	-5.77508	0.33999	0.31639
H	-3.47676	-2.34995	-2.15757
C	-4.54091	-0.85393	-1.00164
H	-5.81442	-3.88685	1.10452
Si	-3.94488	0.59971	-2.01863
H	-4.12060	0.31470	-3.47030
H	-4.64688	1.83498	-1.57869
O	-2.31833	0.90440	-1.73132
C	-1.30593	-0.01940	-2.13491
H	-1.40368	-0.17119	-3.23535
O	-0.07639	0.42570	-1.84276
C	4.58650	-0.55653	-1.65648
C	3.99895	0.01667	-0.52017
C	5.66890	-1.42634	-1.53975
H	6.12585	-1.85329	-2.43535
C	4.52417	-0.30826	0.73952
C	6.17435	-1.73861	-0.27986
H	4.08889	0.13563	1.64199
H	7.02743	-2.41477	-0.18641
C	5.60237	-1.17695	0.86222
H	6.00786	-1.41063	1.84967
H	4.20589	-0.30286	-2.65167
Si	2.61735	1.25582	-0.70135
H	1.41906	0.63232	1.16433
H	2.95266	2.47963	0.08187
H	2.37492	1.57754	-2.13351
H	-4.12477	-0.74239	3.11082
C	-0.45980	3.70682	-2.15151
H	0.15263	4.27319	-2.86793
H	-0.51506	2.66405	-2.48526
H	-1.47740	4.13861	-2.14815
C	1.27374	2.97630	2.73207
H	0.63401	2.16677	3.11561
H	2.29568	2.57470	2.58448
H	1.32083	3.75063	3.51140
C	1.56405	-2.44836	-2.39362
H	2.56450	-2.17339	-2.77071
H	1.22063	-3.33583	-2.94666
H	0.88637	-1.60907	-2.60036
C	1.88099	-1.92118	2.78019
H	1.13769	-2.59495	3.25059
H	2.82880	-2.03092	3.32968
H	1.53899	-0.88267	2.90989
H	-0.61190	-3.18926	1.32927
H	-2.04117	3.12242	1.63921
H	-1.14926	-2.63529	-0.26044
H	-2.38205	2.55515	-0.02040

I5_Ni_L6

Ni	-1.06306	0.69178	-0.20847
H	0.99146	-0.90032	1.42174
P	-1.25471	-1.50437	-0.49213
N	0.28965	-1.85803	-1.22758
C	1.15403	-0.82810	-1.45454
N	2.35924	-1.07558	-1.95953
C	3.13875	-0.01098	-2.07798
N	2.84466	1.23440	-1.72389
C	1.61213	1.40193	-1.25485
N	0.72605	0.40155	-1.14114
N	1.19473	2.63993	-0.87084
P	-0.36671	2.77795	-0.12577
C	0.75562	-3.22467	-1.42678
H	1.33827	-3.28479	-2.35260
H	-0.10225	-3.90219	-1.50889
H	1.39734	-3.54755	-0.59171
C	2.15528	3.73298	-0.81345
H	2.78496	3.65596	0.08676
H	1.62494	4.69287	-0.79973
H	2.80064	3.70208	-1.69791
C	5.02702	-2.99183	-0.19816
C	5.55274	-1.71894	-0.39912
C	3.99621	-3.18058	0.72086
H	6.37656	-1.57156	-1.10189
H	3.60313	-4.18412	0.90318
C	5.03153	-0.63318	0.30218
C	3.48977	-2.09452	1.42879
H	5.45180	0.36427	0.13239
H	2.70980	-2.26841	2.17924
C	3.98913	-0.79748	1.22442
H	5.43397	-3.84466	-0.74633
Si	3.30619	0.66509	2.17054
H	3.30557	0.37115	3.63178
H	4.07495	1.89143	1.83370
O	1.71997	0.98985	1.71134
C	0.69027	0.03071	1.96712
H	0.71377	-0.21699	3.05493
O	-0.52728	0.48115	1.63484
C	-4.35189	-0.78074	1.45975
C	-4.40841	-0.03630	0.27179
C	-5.20556	-1.86266	1.65837
H	-5.16258	-2.42950	2.59143
C	-5.35149	-0.39069	-0.70355
C	-6.12610	-2.21290	0.67122
H	-5.42506	0.19033	-1.62922
H	-6.79713	-3.06053	0.82844
C	-6.20433	-1.47336	-0.50793
H	-6.93752	-1.73784	-1.27299

H	-3.63363	-0.50347	2.24004
Si	-3.26298	1.40423	0.00315
H	-2.03317	0.95924	-1.32990
H	-3.84216	2.32143	-1.01946
H	-3.03566	2.17062	1.26500
H	4.12939	-0.17533	-2.51607
C	-2.45653	-2.21416	-1.65365
H	-2.47390	-1.62217	-2.57851
H	-2.20931	-3.25856	-1.89034
H	-3.45352	-2.18589	-1.19037
C	-1.21534	4.04150	-1.11915
H	-1.42024	3.65827	-2.12758
H	-2.16796	4.29550	-0.63017
H	-0.61463	4.95963	-1.18795
C	-1.33433	-2.60132	0.95018
H	-2.34442	-2.52450	1.37506
H	-1.15705	-3.64487	0.65410
H	-0.60596	-2.29329	1.70889
C	-0.11288	3.55122	1.48611
H	-1.06827	3.52551	2.02910
H	0.62375	2.95939	2.04224
H	0.21062	4.59523	1.36957

I5_Ni_L7

Ni	-0.85125	0.47093	-0.33293
H	1.02468	-1.27829	1.46871
P	-1.72944	-1.48876	-0.43980
N	-0.38331	-2.49022	-0.82512
C	0.79741	-1.88304	-1.17268
N	1.84855	-2.61420	-1.51868
C	2.93637	-1.90774	-1.81543
N	3.07017	-0.59193	-1.74580
C	1.97838	0.06071	-1.35786
N	0.80720	-0.54721	-1.13308
N	2.00202	1.41252	-1.16947
P	0.61044	2.13542	-0.44184
O	0.22143	3.26809	-1.57392
C	0.26863	4.59602	-1.06163
H	-0.74390	4.90386	-0.75385
H	0.61458	5.26691	-1.85863
C	1.22793	4.54756	0.12029
H	2.26803	4.74385	-0.18273
H	0.95422	5.24613	0.92004
O	1.14183	3.22044	0.64487
O	-2.86955	-1.78485	-1.56971
C	-3.82805	-2.75260	-1.13346
H	-3.55415	-3.73812	-1.54110
H	-4.81070	-2.46650	-1.52838
C	-3.78348	-2.72278	0.39186
H	-4.50338	-2.00834	0.81704
H	-3.94965	-3.71272	0.83527
O	-2.46024	-2.30313	0.74809
C	-0.52143	-3.93799	-0.92874
H	-0.80304	-4.23647	-1.94906
H	-1.28052	-4.27956	-0.21570
H	0.43467	-4.40883	-0.67702
C	3.19015	2.20618	-1.44049
H	3.62864	2.57868	-0.50297
H	2.93302	3.05245	-2.09228
H	3.92556	1.57349	-1.94804
C	5.76280	-2.38426	0.27187
C	6.02271	-1.02649	0.10715
C	4.69412	-2.80023	1.06631
H	6.86471	-0.69962	-0.50689
H	4.49812	-3.86557	1.20689
C	5.20617	-0.08651	0.73045
C	3.89330	-1.85454	1.69914
H	5.42411	0.97863	0.59666
H	3.07853	-2.19765	2.34671
C	4.12752	-0.47958	1.53424
H	6.40408	-3.12600	-0.21090
Si	3.02028	0.78898	2.34190
H	2.87510	0.52873	3.80002
H	3.52573	2.15310	2.04150
O	1.47381	0.72050	1.66734
C	0.58864	-0.35643	1.92704
H	0.54942	-0.52449	3.02639
O	-0.65292	-0.08843	1.48266
C	-3.81377	0.68568	1.57946
C	-4.06974	0.96602	0.22703
C	-4.81335	0.15316	2.38759
H	-4.60725	-0.05427	3.43971
C	-5.34120	0.68464	-0.29746
C	-6.07561	-0.11106	1.85644
H	-5.56073	0.90442	-1.34711
H	-6.86139	-0.51899	2.49659
C	-6.33910	0.14820	0.51238
H	-7.32895	-0.05501	0.09745
H	-2.81951	0.87004	1.99889
Si	-2.80840	1.84005	-0.82840
H	-1.57917	1.00849	-1.60152
H	-3.39310	2.16623	-2.15623
H	-2.26479	3.06358	-0.18273
H	3.81283	-2.47378	-2.14963

4.5. Transition States

4.5.1. TS1

TS1_Co_L1

Co	-0.01770	-0.62525	-0.19389
H	-0.07194	-2.00208	0.44544
P	2.00170	-0.27038	0.29324
N	2.16886	1.43049	-0.30429
C	1.02263	2.03035	-0.68332
N	1.00942	3.32455	-1.03026
C	-0.18991	3.81332	-1.30341
N	-1.35329	3.19231	-1.17884
C	-1.26594	1.90529	-0.82111
N	-0.08781	1.26084	-0.67026
N	-2.38024	1.18807	-0.55784
P	-2.08066	-0.40732	0.22897
N	-3.41385	-1.31816	-0.32900
C	-4.33835	-1.69345	0.72308
H	-4.08535	-2.69473	1.12861
H	-5.36910	-1.74803	0.33139
C	-4.19735	-0.63349	1.79982
H	-4.86182	0.23381	1.60024
H	-4.46731	-1.02452	2.79510
N	-2.80024	-0.25762	1.77027
C	-2.35081	0.75774	2.68015
H	-1.26744	0.90621	2.55850
C	-3.25767	-2.25226	-1.41644
N	3.45052	-0.93214	-0.30793
C	4.40841	-1.33565	0.69469
H	5.43786	-1.14200	0.34225
H	4.33456	-2.42046	0.90909
C	4.07971	-0.51076	1.92991
H	4.33754	-1.04591	2.85930
H	4.64835	0.44342	1.93900
N	2.65345	-0.27664	1.86873
C	2.05166	0.49700	2.91587
H	0.97008	0.58286	2.73509
C	3.61551	-1.38734	-1.65835
H	4.56944	-1.02243	-2.07825
C	3.40642	2.17500	-0.28083
H	3.48804	2.81084	-1.17207
H	4.24191	1.46288	-0.27310
H	3.47770	2.82763	0.60471
C	-3.68195	1.80472	-0.67061
H	-3.89346	2.48056	0.17461
H	-4.43955	1.01145	-0.70309
H	-3.74379	2.39606	-1.59323
C	0.52615	-2.51951	-1.48077
O	-0.06847	-1.77949	-2.19462
O	1.17525	-3.44332	-1.16593
H	-0.22674	4.85508	-1.64687
H	2.19424	0.00961	3.89403
H	-2.53667	0.45470	3.72294
H	-2.55087	-1.85981	-2.15873
H	-2.88464	-3.23700	-1.07202
H	-4.22568	-2.41084	-1.91796
H	3.60276	-2.48942	-1.73802
H	2.80736	-0.98970	-2.29074
H	2.47428	1.52040	2.98095
H	-2.85056	1.73347	2.51329

TS1_Co_L2

Co	-0.00816	-0.62365	-0.07961
H	-0.06051	-1.84953	0.80707
P	1.99917	-0.14921	0.33756
O	2.14485	1.39506	-0.62739
C	1.03042	1.87067	-1.09606
N	1.01544	3.05631	-1.69521
C	-0.18774	3.48487	-2.05054
N	-1.35155	2.90078	-1.79855
C	-1.26352	1.72062	-1.19628
N	-0.08228	1.12397	-0.92105
O	-2.34429	1.10669	-0.81264
P	-2.06712	-0.31269	0.29960
N	-3.39832	-1.24844	-0.07834
C	-4.55110	-1.04628	0.77857
H	-4.66501	-1.90526	1.46837
H	-5.47528	-0.97701	0.17978
C	-4.27783	0.24060	1.53775
H	-4.65405	1.11891	0.97414
H	-4.75592	0.24821	2.52998
N	-2.83071	0.30998	1.67881
C	-2.30725	1.50518	2.29097
H	-1.20958	1.45579	2.31966
C	-3.39098	-2.37553	-0.97284
N	3.42925	-0.88472	-0.12595
C	4.54812	-0.68660	0.77405
H	5.46087	-0.43774	0.20590
H	4.75387	-1.61169	1.34562
C	4.13697	0.45314	1.69121
H	4.59175	0.36905	2.69091
H	4.43722	1.43229	1.26443
N	2.68837	0.36978	1.79360
C	2.04144	1.40174	2.56167
H	0.95182	1.26252	2.52837
C	3.60279	-1.69615	-1.29868
H	4.41107	-1.29567	-1.93390
C	0.47510	-2.72865	-0.85428
O	-0.07763	-2.15533	-1.74537
O	1.09323	-3.60074	-0.36762
H	-0.22684	4.43832	-2.59131
H	2.35839	1.34924	3.61449
H	-2.67136	1.59128	3.32589
H	-2.44403	-2.41650	-1.52490
H	-3.51679	-3.32524	-0.42229
H	-4.20878	-2.29629	-1.70821
H	3.84549	-2.74144	-1.04178
H	2.68362	-1.69601	-1.90065
H	2.27801	2.41346	2.17782
H	-2.60369	2.41964	1.74067

TS1_Co_L3

Co	0.11027	-0.65982	-0.32011
H	0.24893	-1.94071	0.47411
P	2.01978	-0.03225	0.28307
C	2.18185	1.60137	-0.65898
C	0.84824	2.05311	-1.12485
N	0.65185	3.31858	-1.47559
C	-0.58803	3.63282	-1.83758
N	-1.62718	2.81300	-1.84013
C	-1.37431	1.55713	-1.47514

N	-0.13538	1.12727	-1.13322
C	-2.47021	0.58718	-1.28525
P	-1.95711	-0.43821	0.25270
N	-3.20539	-1.60134	0.40343
C	-4.09032	-1.33400	1.52180
H	-3.77303	-1.90965	2.41575
H	-5.12611	-1.63329	1.28493
C	-3.97997	0.15225	1.78677
H	-4.63332	0.72332	1.09039
H	-4.28468	0.41569	2.81292
N	-2.58036	0.47690	1.57900
C	-2.26026	1.87876	1.66724
H	-1.19803	2.04710	1.43761
C	-2.94737	-2.99048	0.11339
N	3.64143	-0.61805	0.15305
C	4.37504	-0.55422	1.39901
H	5.43917	-0.32238	1.21389
H	4.33486	-1.52590	1.93481
C	3.69536	0.52295	2.21967
H	3.89039	0.40610	3.29870
H	4.06300	1.53206	1.92567
N	2.28745	0.35134	1.94370
C	1.36188	1.20268	2.63349
H	0.33603	0.84266	2.46775
C	4.00251	-1.68250	-0.74339
H	5.06379	-1.59058	-1.02909
C	0.52119	-2.48741	-1.80488
O	-0.42093	-1.88425	-2.18752
O	1.38820	-3.26295	-1.71714
H	-0.76760	4.66866	-2.14898
H	1.55964	1.17121	3.71652
H	-2.44280	2.24587	2.68944
H	-2.27279	-3.08363	-0.74731
H	-2.48685	-3.51996	0.96997
H	-3.88942	-3.50218	-0.14135
H	3.85242	-2.68584	-0.29928
H	3.40554	-1.63288	-1.66368
H	1.42226	2.26800	2.31620
H	-2.86637	2.49767	0.97339
H	-3.45123	1.08238	-1.27167
H	2.70809	2.41835	-0.14040
H	-2.46482	-0.18020	-2.07632
H	2.79708	1.34598	-1.53985

TS1_Co_L4

Co	-0.00817	-0.61168	-0.21154
H	-0.03943	-2.03257	0.32658
P	1.99620	-0.29790	0.29596
N	2.18188	1.40605	-0.24065
C	1.03589	2.05621	-0.60297
C	1.00381	3.42091	-0.93627
C	-0.22354	3.99153	-1.23769
C	-1.39610	3.25176	-1.16335
C	-1.29954	1.89560	-0.81686
N	-0.09522	1.31164	-0.60514
N	-2.39679	1.09484	-0.62935
P	-2.06169	-0.43589	0.23545
N	-3.38607	-1.40749	-0.24943
C	-4.24902	-1.78330	0.85342
H	-3.92171	-2.74446	1.30158
H	-5.28689	-1.92035	0.50276
C	-4.13673	-0.66168	1.86866
H	-4.84494	0.16259	1.63569
H	-4.37250	-1.00946	2.88868
N	-2.75987	-0.22748	1.78746
C	-2.35377	0.88875	2.59376
H	-1.28104	1.08135	2.44171
C	-3.21196	-2.38973	-1.29114
N	3.46808	-0.94548	-0.27741
C	4.37574	-1.41704	0.74033
H	5.42247	-1.23201	0.43661
H	4.27267	-2.50809	0.90601
C	4.01615	-0.64000	1.99804
H	4.23431	-1.21841	2.91196
H	4.60124	0.30282	2.06946
N	2.59864	-0.37990	1.89605
C	1.98055	0.38640	2.93828
H	0.90302	0.47880	2.73659
C	3.69581	-1.30409	-1.64637
H	4.68253	-0.93922	-1.98401
C	3.43395	2.11492	-0.19857
H	3.66082	2.59219	-1.16670
H	4.23825	1.39939	0.00737
H	3.44763	2.89657	0.58217
C	-3.72071	1.63700	-0.79121
H	-3.94947	2.42466	-0.04931
H	-4.44659	0.82254	-0.68158
H	-3.85421	2.06430	-1.79790
C	0.50411	-2.36432	-1.73413
O	-0.14854	-1.57407	-2.32884
O	1.18398	-3.29434	-1.52508
H	-0.27166	5.04876	-1.51057
H	2.10125	-0.11086	3.91454
H	-2.51319	0.67692	3.66311
H	-2.57477	-1.98879	-2.08994
H	-2.74930	-3.32363	-0.91479
H	-4.18897	-2.64545	-1.73137
H	3.65466	-2.39520	-1.81640
H	2.93661	-0.83153	-2.28812
H	2.40743	1.40771	3.02111
H	-2.90352	1.81986	2.34351
H	-2.36357	3.71306	-1.35356
H	1.91592	4.01521	-0.94768

TS1_Co_L5

Co	-1.06876	0.61541	-0.28926
H	-2.32280	1.40744	0.02756
P	-1.89433	-1.24756	0.25908
N	-0.42291	-2.29488	0.08906
C	0.73159	-1.64196	-0.12831
N	1.89833	-2.30058	-0.14814
C	2.95971	-1.52963	-0.28851
N	3.00896	-0.21418	-0.32041
C	1.80820	0.38502	-0.28584
N	0.64560	-0.30286	-0.29939
N	1.71766	1.72548	-0.19993
P	0.05983	2.34679	0.18002
N	0.08721	3.87196	-0.58117
C	0.03669	4.98548	0.34696
H	-1.00941	5.32211	0.49742
H	0.60479	5.84494	-0.04944
C	0.62382	4.47132	1.64874
H	1.72769	4.58955	1.66688
H	0.22614	5.01783	2.51996
N	0.23267	3.07860	1.70974
C	0.65453	2.30182	2.84144
H	0.24915	1.28236	2.75870
C	-0.53507	4.06870	-1.86732
N	-3.04771	-2.24167	-0.49999
C	-4.09035	-2.74079	0.36781
H	-4.38815	-3.76000	0.06280
H	-4.99594	-2.10428	0.31358
C	-3.49742	-2.72913	1.76827
H	-4.27154	-2.57564	2.53857
H	-2.99616	-3.69219	2.00145
N	-2.55913	-1.62732	1.77883

C	-1.83801	-1.36204	2.99042
H	-1.17774	-0.49461	2.84399
C	-3.24945	-2.27831	-1.92136
H	-3.36436	-3.31914	-2.26966
C	-0.42538	-3.72575	0.29456
H	0.25856	-4.21588	-0.41024
H	-1.44258	-4.09980	0.12191
H	-0.10675	-3.99355	1.31490
C	2.91440	2.53574	-0.15745
H	3.39971	2.50111	0.83168
H	2.63938	3.57127	-0.39412
H	3.64120	2.18095	-0.89900
C	-2.67575	0.94864	-1.94318
O	-1.61543	1.00916	-2.47924
O	-3.84363	0.88763	-1.85378
H	-2.53098	-1.12490	3.81375
H	0.27548	2.74241	3.77714
H	-0.42293	3.17040	-2.48755
H	-1.61589	4.29389	-1.77832
H	-0.05421	4.90860	-2.39341
H	-4.14207	-1.70755	-2.23546
H	-2.37413	-1.85560	-2.43704
H	-1.21466	-2.22236	3.30739
H	1.75774	2.22691	2.92122
C	4.28257	-2.28571	-0.37137
F	4.51345	-2.92760	0.77591
F	4.24111	-3.19537	-1.34333
F	5.30895	-1.48317	-0.60442

TS1_Co_L6

Co	0.06507	-0.90288	0.21818
H	0.16133	-2.32836	0.76402
P	2.09185	-0.42781	0.55167
N	2.13216	1.27234	0.02368
C	0.92546	1.80783	-0.27411
N	0.82875	3.10229	-0.59733
C	-0.40742	3.53244	-0.79874
N	-1.52664	2.83947	-0.65140
C	-1.35205	1.55467	-0.32370
N	-0.13785	0.97913	-0.20083
N	-2.42936	0.77094	-0.06860
P	-2.05246	-0.85699	0.53622
C	3.30131	2.12603	0.01449
H	3.42167	2.61343	-0.96305
H	4.19449	1.52383	0.21797
H	3.22770	2.91829	0.77540
C	-3.75312	1.35374	-0.13188
H	-3.91137	2.09302	0.66935
H	-4.50512	0.56146	-0.03455
H	-3.90447	1.86475	-1.09202
C	0.58657	-2.50340	-1.42726
O	-0.25170	-1.79794	-1.88735
O	1.41383	-3.33158	-1.39122
H	-0.51748	4.58081	-1.10326
C	-2.91211	-0.86743	2.15515
H	-3.98239	-0.62293	2.07287
H	-2.81143	-1.87859	2.57708
H	-2.41735	-0.16172	2.83555
C	2.81740	-0.31839	2.23388
H	2.12273	0.21603	2.89512
H	2.93004	-1.34602	2.61110
H	3.80277	0.17302	2.24406
C	3.52012	-1.12345	-0.36799
H	4.48273	-0.66992	-0.09038
H	3.55889	-2.20080	-0.14960
H	3.35276	-1.00884	-1.44817
C	-3.18281	-1.89507	-0.46282
H	-2.89871	-1.81699	-1.52045
H	-3.03659	-2.93879	-0.14604
H	-4.24446	-1.63664	-0.33640

TS1_Fe_L1

Fe	0.00774	-0.69924	-0.32319
H	0.15560	-2.02044	0.42024
P	2.01020	-0.41248	0.24145
N	2.35345	1.20402	-0.53426
C	1.24530	1.90987	-0.83822
N	1.33125	3.20492	-1.18204
C	0.16410	3.81787	-1.31994
N	-1.03382	3.32026	-1.06014
C	-1.04220	2.01740	-0.72636
N	0.07110	1.24521	-0.72935
N	-2.17437	1.40923	-0.33724
P	-1.95953	-0.30798	0.26780
N	-3.46078	-0.96989	-0.30117
C	-4.40625	-1.34281	0.71457
H	-4.30539	-2.41647	0.98581
H	-5.44639	-1.19817	0.36005
C	-4.10067	-0.45529	1.91117
H	-4.68109	0.49497	1.86146
H	-4.38361	-0.94482	2.86234
N	-2.68397	-0.22490	1.85600
C	-2.10495	0.61231	2.85599
H	-1.02348	0.70586	2.66464
C	-3.55000	-1.64783	-1.56347
N	3.40042	-1.30673	-0.28908
C	4.34575	-1.62592	0.75151
H	5.38030	-1.65383	0.35755
H	4.13579	-2.63153	1.17866
C	4.17961	-0.55852	1.81971
H	4.47676	-0.93458	2.81659
H	4.82560	0.32390	1.60883
N	2.78072	-0.22655	1.79406
C	2.31487	0.78522	2.68984
H	1.23250	0.92720	2.53885
C	3.23488	-2.30247	-1.31768
H	4.16445	-2.40954	-1.90476
C	3.65100	1.81850	-0.62178
H	3.72301	2.43194	-1.53079
H	4.40922	1.02449	-0.66614
H	3.86541	2.47853	0.23806
C	-3.41499	2.13549	-0.28813
H	-3.51562	2.72806	0.63880
H	-4.24197	1.41387	-0.34313
H	-3.48720	2.83101	-1.13567
C	-0.39057	-2.34647	-1.23416
O	0.13833	-1.68034	-2.15714
O	-0.94994	-3.42038	-1.14473
H	0.19770	4.86246	-1.66017
H	2.47747	0.49230	3.74236
H	-2.23700	0.18434	3.86642
H	-2.72614	-1.32996	-2.21872
H	-3.47721	-2.74650	-1.45870
H	-4.50316	-1.40119	-2.06825
H	2.98379	-3.29646	-0.89361
H	2.41838	-2.01783	-1.99550
H	2.81240	1.76672	2.53208
H	-2.54192	1.63535	2.86608

TS1_Fe_L2

Fe	-0.00796	-0.71333	-0.15368
H	-0.15158	-1.81554	0.87634
P	1.94474	-0.24675	0.35790

O	2.19267	1.31633	-0.68813
C	1.09542	1.81646	-1.14070
N	1.10273	3.01038	-1.74668
C	-0.09458	3.48097	-2.05870
N	-1.27262	2.94617	-1.76970
C	-1.20935	1.75288	-1.17182
N	-0.04282	1.10814	-0.94478
O	-2.29710	1.19153	-0.74647
P	-1.99181	-0.30102	0.33237
N	-3.37733	-1.19790	-0.06246
C	-4.53170	-0.99103	0.77320
H	-4.66390	-1.84341	1.47371
H	-5.45644	-0.91909	0.17002
C	-4.25613	0.29750	1.52915
H	-4.60010	1.17603	0.94074
H	-4.77469	0.32621	2.50373
N	-2.82154	0.33448	1.71120
C	-2.30016	1.52529	2.32084
H	-1.20048	1.48053	2.32331
C	-3.34747	-2.34609	-0.92453
N	3.41560	-1.00901	-0.01297
C	4.53495	-0.71252	0.84209
H	5.44864	-0.52300	0.24711
H	4.75582	-1.56887	1.51384
C	4.12542	0.51722	1.63577
H	4.61203	0.55289	2.62679
H	4.40834	1.44504	1.09163
N	2.68991	0.42533	1.77290
C	2.05313	1.54663	2.40058
H	0.96082	1.41673	2.35627
C	3.57866	-1.99681	-1.03875
H	4.38877	-1.70949	-1.73500
C	0.29088	-2.51171	-0.77758
O	-0.13412	-1.95729	-1.81920
O	0.73257	-3.60155	-0.48423
H	-0.11545	4.43792	-2.59871
H	2.34846	1.62260	3.46113
H	-2.64331	1.61062	3.36576
H	-2.39194	-2.38461	-1.46562
H	-3.46248	-3.28745	-0.35279
H	-4.16331	-2.30072	-1.66868
H	3.81798	-2.99077	-0.61792
H	2.65174	-2.09962	-1.61734
H	2.30751	2.50557	1.90239
H	-2.60773	2.44349	1.77896

TS1_Fe_L3

Fe	-0.04071	-0.64520	-0.38579
H	-0.04505	-1.69195	0.70905
P	1.96847	-0.33306	0.21110
C	2.44883	0.94061	-1.15225
C	1.28676	1.83264	-1.28270
N	1.44161	3.13840	-1.50957
C	0.33241	3.86040	-1.51194
N	-0.90051	3.39570	-1.30807
C	-0.99318	2.09266	-1.06808
N	0.07615	1.26040	-1.03356
C	-2.30039	1.47337	-0.76286
P	-2.00335	-0.14123	0.19871
N	-3.55023	-0.92508	-0.05091
C	-4.43385	-0.87886	1.08407
H	-4.36927	-1.81788	1.67839
H	-5.49102	-0.76659	0.77088
C	-3.97114	0.29730	1.92140
H	-4.39892	1.25004	1.52513
H	-4.30279	0.21110	2.97282
N	-2.53632	0.27169	1.83065
C	-1.84409	1.32946	2.49955
H	-0.76179	1.21670	2.33078
C	-3.66469	-2.08458	-0.89075
N	3.29362	-1.45589	0.12403
C	4.20180	-1.36090	1.24073
H	5.24020	-1.59117	0.93415
H	3.92746	-2.08414	2.04069
C	4.06846	0.05688	1.75780
H	4.42601	0.15457	2.79897
H	4.67662	0.75520	1.13572
N	2.65983	0.35352	1.66842
C	2.28288	1.69796	1.99690
H	1.20394	1.83129	1.82212
C	3.05659	-2.78355	-0.38603
H	4.00620	-3.22725	-0.73321
C	-0.25344	-2.36926	-1.16603
O	0.32561	-1.73279	-2.08962
O	-0.68171	-3.49574	-1.03032
H	0.43401	4.93702	-1.69838
H	2.48239	1.91428	3.06033
H	-2.02555	1.29128	3.58810
H	-2.87143	-2.08695	-1.65066
H	-3.56599	-3.03035	-0.32148
H	-4.64161	-2.09524	-1.40935
H	2.62123	-3.45856	0.37863
H	2.36304	-2.75012	-1.23715
H	2.82212	2.45698	1.38841
H	-2.14856	2.34234	2.14744
H	3.39113	1.50200	-1.06550
H	-2.99805	2.21278	-0.33872
H	-2.74426	1.10014	-1.70182
H	2.50954	0.24638	-2.00832

TS1_Fe_L4

Fe	-0.00061	-0.68726	-0.34872
H	-0.14271	-2.07468	0.27504
P	1.95329	-0.32799	0.27165
N	2.18833	1.38222	-0.31365
C	1.05354	2.04088	-0.66546
C	1.02436	3.41475	-0.97765
C	-0.20275	4.00368	-1.24603
C	-1.37896	3.26910	-1.14548
C	-1.28408	1.90355	-0.82393
N	-0.08045	1.29390	-0.67055
N	-2.37692	1.11800	-0.59138
P	-1.99502	-0.44277	0.25020
N	-3.36396	-1.39826	-0.22809
C	-4.26899	-1.72461	0.84552
H	-3.99491	-2.69642	1.31325
H	-5.30705	-1.82825	0.47398
C	-4.14377	-0.60230	1.86151
H	-4.83046	0.23994	1.61398
H	-4.42022	-0.94324	2.87715
N	-2.76165	-0.21222	1.80671
C	-2.34437	0.89340	2.61103
H	-1.26936	1.07235	2.44542
C	-3.17831	-2.41912	-1.22851
N	3.47126	-0.98898	-0.25855
C	4.37379	-1.40530	0.77745
H	5.42765	-1.27157	0.45995
H	4.24771	-2.48268	1.02249
C	4.04500	-0.53830	1.98370
H	4.29207	-1.05219	2.93255
H	4.64418	0.40301	1.97374
N	2.63643	-0.28327	1.88821
C	2.04461	0.56335	2.87150
H	0.97028	0.67449	2.64969
C	3.62384	-1.58638	-1.55334
H	4.60520	-1.31672	-1.98931

C	3.44513	2.07125	-0.28128
H	3.66077	2.56763	-1.24403
H	4.24161	1.33705	-0.10933
H	3.49111	2.84063	0.51417
C	-3.69748	1.65749	-0.72884
H	-3.92686	2.43998	0.02241
H	-4.42134	0.83964	-0.62050
H	-3.84705	2.09821	-1.72969
C	0.41084	-2.23916	-1.39773
O	-0.20299	-1.53902	-2.24098
O	1.01545	-3.29307	-1.41720
H	-0.24833	5.06604	-1.50386
H	2.14062	0.13488	3.88607
H	-2.49484	0.68779	3.68577
H	-2.40108	-2.11380	-1.94291
H	-2.86136	-3.38387	-0.78166
H	-4.12001	-2.59311	-1.77924
H	3.53567	-2.68850	-1.52735
H	2.83815	-1.21800	-2.22905
H	2.49791	1.57996	2.89452
H	-2.88422	1.83533	2.36800
H	-2.35024	3.73985	-1.29429
H	1.94349	3.99981	-0.99607

TS1_Fe_L5

Fe	1.09716	0.62848	-0.39338
H	2.26191	1.48559	0.08362
P	-0.06232	2.29130	0.17326
N	-1.73420	1.67747	-0.19415
C	-1.81381	0.33647	-0.30004
N	-3.00290	-0.27667	-0.31593
C	-2.94058	-1.59852	-0.28602
N	-1.86550	-2.34993	-0.15365
C	-0.70724	-1.67174	-0.15260
N	-0.62882	-0.32822	-0.33934
N	0.45866	-2.30197	0.06881
P	1.90482	-1.21064	0.22179
N	3.07945	-2.22034	-0.56545
C	4.16037	-2.65867	0.27668
H	5.00933	-1.94098	0.24846
H	4.55177	-3.63799	-0.06085
C	3.57822	-2.74411	1.67815
H	3.12440	-3.74507	1.86121
H	4.35687	-2.60425	2.45109
N	2.60328	-1.69011	1.73961
C	1.88816	-1.49531	2.95943
H	1.18593	-0.65536	2.83435
C	3.36842	-2.05507	-1.96422
N	-0.13372	3.85590	-0.56612
C	-0.15017	4.96211	0.35908
H	-0.74977	5.80473	-0.03612
H	0.87911	5.34876	0.52597
C	-0.72610	4.42362	1.65814
H	-0.35652	4.99338	2.53077
H	-1.83602	4.50910	1.67169
N	-0.28705	3.05490	1.71849
C	-0.69951	2.26598	2.83816
H	-0.28948	1.24883	2.73104
C	0.55926	4.07905	-1.80961
H	0.02942	4.83198	-2.41968
C	-2.93441	2.47086	-0.13894
H	-3.66491	2.11046	-0.87616
H	-2.67397	3.51157	-0.37504
H	-3.41956	2.43124	0.85230
C	0.49543	-3.72101	0.31000
H	0.27541	-3.96768	1.36350
H	1.49936	-4.08858	0.05723
H	-0.24533	-4.23660	-0.31554
C	2.59674	1.03298	-1.54579
O	1.64497	1.05318	-2.36093
O	3.80748	1.08337	-1.61231
H	-0.31994	2.68999	3.78439
H	2.57154	-1.24688	3.79144
H	2.50089	-1.61306	-2.47578
H	4.22894	-1.38228	-2.13863
H	3.57950	-3.03399	-2.43220
H	1.59420	4.44231	-1.64450
H	0.62713	3.14323	-2.38080
H	-1.80421	2.18256	2.92474
H	1.30319	-2.39104	3.26188
C	-4.28411	-2.29579	-0.36713
F	-5.05781	-1.98117	0.68358
F	-4.95992	-1.92768	-1.46363
F	-4.17902	-3.62059	-0.39414

TS1_Fe_L6

Fe	-0.06855	-0.96544	0.06604
H	-0.08790	-2.26100	0.89187
P	2.01600	-0.86061	0.51898
N	2.41684	0.79615	-0.03276
C	1.33803	1.58222	-0.29312
N	1.50933	2.88125	-0.56650
C	0.38616	3.57424	-0.70676
N	-0.84999	3.12338	-0.53714
C	-0.93990	1.81548	-0.26510
N	0.12816	0.98320	-0.21235
N	-2.13879	1.25469	0.01187
P	-2.05653	-0.46775	0.52428
C	3.72889	1.39124	-0.03730
H	3.89530	1.96105	-0.96296
H	4.48903	0.60160	0.02709
H	3.87337	2.08790	0.80732
C	-3.31604	2.08457	0.03305
H	-3.31794	2.78151	0.88963
H	-4.20847	1.44886	0.09786
H	-3.38271	2.69089	-0.88206
C	-0.55070	-2.37616	-1.10996
O	0.23430	-1.73740	-1.85940
O	-1.27623	-3.34591	-1.23760
H	0.49181	4.63486	-0.97144
C	-2.87936	-0.33549	2.19153
H	-3.87256	0.14596	2.16632
H	-2.99426	-1.36400	2.56893
H	-2.21121	0.20793	2.87399
C	2.96998	-0.93150	2.10783
H	2.53929	-0.20858	2.81457
H	2.82287	-1.94129	2.52128
H	4.05171	-0.74848	1.99110
C	3.16067	-1.85442	-0.53229
H	4.21065	-1.52134	-0.50582
H	3.11070	-2.89382	-0.17115
H	2.76837	-1.83625	-1.55852
C	-3.50123	-1.17057	-0.39379
H	-3.35925	-0.97078	-1.46599
H	-3.44387	-2.26258	-0.26633
H	-4.48711	-0.80736	-0.06198

TS1_Fe_L8

Fe	0.64408	-0.17855	-0.10225
H	1.29790	-0.37198	1.26540
C	-1.59895	1.50211	-0.17982
N	-2.82835	1.81319	0.10203
C	-3.68550	0.78294	0.32759

N	-3.34764	-0.51797	0.13961
C	-2.10294	-0.76516	-0.14212
N	-1.10959	0.20385	-0.18281
C	2.56317	-0.36233	0.24266
O	2.46488	-0.67246	-0.97167
O	3.51078	-0.13738	0.98037
H	-4.72548	1.01868	0.56494
C	-1.50993	-2.06538	-0.55631
H	-2.12726	-2.93637	-0.26295
H	-1.40194	-2.06230	-1.65934
C	-0.51224	2.41954	-0.61658
H	-0.42530	2.35447	-1.71949
H	-0.69748	3.47451	-0.33706
N	-0.12870	-2.16879	-0.01189
N	0.78702	1.93602	-0.07537
C	-0.19953	-2.58810	1.38323
H	-0.64706	-3.60097	1.46458
H	0.80703	-2.59893	1.82059
H	-0.82011	-1.87917	1.94773
C	0.66081	-3.11820	-0.77843
H	0.76795	-2.75809	-1.81012
H	1.66748	-3.19291	-0.34642
H	0.18855	-4.12289	-0.78064
C	0.91856	2.35865	1.31705
H	0.04717	2.00911	1.88686
H	1.82733	1.92304	1.75180
H	0.96867	3.46570	1.38252
C	1.89349	2.46218	-0.86130
H	1.86348	3.57090	-0.89842
H	2.84627	2.14630	-0.41568
H	1.84065	2.06033	-1.88221

TS1_Fe_L9

Fe	-0.07399	-1.01162	0.01253
H	-0.00196	-2.31777	0.87146
P	1.98056	-0.84007	0.48535
N	2.50198	0.74642	-0.09989
C	1.40213	1.59077	-0.33883
C	1.56208	2.97066	-0.57117
C	0.42677	3.77308	-0.71696
C	-0.85189	3.22100	-0.59147
C	-0.98348	1.83819	-0.36143
C	0.13214	0.96942	-0.27805
N	-2.22527	1.22882	-0.14655
P	-1.99879	-0.40786	0.52063
C	3.81356	1.28671	0.01086
H	4.12743	1.80635	-0.91718
H	4.54284	0.48263	0.19169
H	3.91573	2.02449	0.83986
C	-3.39218	2.02737	0.02124
H	-3.31716	2.74302	0.87332
H	-4.26714	1.38491	0.19924
H	-3.61086	2.63094	-0.88240
C	-0.63213	-2.43403	-1.11439
O	0.17692	-1.90313	-1.92684
O	-1.40402	-3.38946	-1.21829
H	0.54038	4.84761	-0.90675
C	-2.62813	-0.09200	2.27631
H	-3.64633	0.34146	2.33432
H	-2.61392	-1.05876	2.80476
H	-1.90477	0.57916	2.76452
C	2.84323	-0.90404	2.14886
H	2.41196	-0.11497	2.78282
H	2.60006	-1.87880	2.60046
H	3.94297	-0.78865	2.09893
C	3.16919	-1.93951	-0.43156
H	4.22240	-1.60608	-0.40637
H	3.09992	-2.94457	0.01601
H	2.80605	-2.00186	-1.46797
C	-3.54654	-1.23781	-0.10114
H	-3.54683	-1.16566	-1.19905
H	-3.44626	-2.30629	0.14566
H	-4.49161	-0.84023	0.31006
H	-1.73022	3.87138	-0.66614
H	2.55584	3.42784	-0.63027

TS1_Fe_L11

Fe	0.67796	0.20488	0.03326
H	1.49661	0.54348	1.33126
C	-2.10764	0.84159	0.24930
C	-3.46419	0.57060	0.30102
C	-3.93840	-0.75392	0.11423
C	-2.99567	-1.78652	-0.14142
C	-1.64626	-1.49413	-0.22601
C	-1.13620	-0.17438	-0.02522
C	2.63013	0.26871	0.22716
O	2.66188	0.95664	-0.84111
O	3.57597	-0.30724	0.80232
H	-5.01111	-0.97572	0.16139
H	-3.35389	-2.81651	-0.29975
H	-4.18654	1.37590	0.51294
C	-0.54041	-2.40810	-0.64837
H	-0.70817	-3.48557	-0.40389
H	-0.41573	-2.33430	-1.74842
C	-1.42469	2.12548	0.60880
H	-2.01260	3.04670	0.37375
H	-1.20860	2.13066	1.69783
N	0.75265	-1.95931	-0.07420
N	-0.09411	2.18650	-0.04431
C	0.87916	-2.41497	1.30150
H	0.93224	-3.53230	1.34005
H	1.78879	-1.98324	1.74139
H	0.00822	-2.07052	1.87460
C	1.85984	-2.46611	-0.86356
H	1.82048	-2.01965	-1.86871
H	2.80833	-2.17384	-0.39106
H	1.81410	-3.57794	-0.95442
C	0.76277	3.17131	0.58379
H	0.89607	2.91200	1.64330
H	1.74864	3.14276	0.09772
H	0.33391	4.19978	0.50258
C	-0.22901	2.49000	-1.45980
H	-0.58846	3.53747	-1.60685
H	0.74804	2.35911	-1.94645
H	-0.94641	1.79202	-1.91074

TS1_Ni_L1

Ni	-0.00603	-0.51899	-0.06426
H	-0.01809	-1.91279	0.52643
P	2.13769	-0.22320	0.21736
N	2.20875	1.48923	-0.20702
C	1.03524	2.11589	-0.46662
N	1.01384	3.42322	-0.71242
C	-0.19186	3.93685	-0.91670
N	-1.35111	3.30020	-0.83283
C	-1.26421	1.99693	-0.57372
N	-0.08076	1.35480	-0.45077
N	-2.38443	1.25680	-0.40215
P	-2.17101	-0.40230	0.19817
N	-3.39382	-1.25640	-0.55227
C	-4.35154	-1.83546	0.38132
H	-4.10738	-2.89249	0.59375
H	-5.35964	-1.81040	-0.06266

C	-4.26915	-0.98732	1.64126
H	-4.97226	-0.13243	1.60222
H	-4.51036	-1.56976	2.54334
N	-2.88685	-0.53095	1.70671
C	-2.46673	0.28768	2.81641
H	-1.38265	0.46706	2.75999
C	-3.35972	-1.72147	-1.91855
N	3.43871	-0.88035	-0.61066
C	4.34801	-1.60265	0.26955
H	5.37107	-1.55125	-0.13459
H	4.06440	-2.66997	0.33904
C	4.23555	-0.91367	1.61981
H	4.46208	-1.60003	2.44968
H	4.93253	-0.05663	1.69699
N	2.84859	-0.47637	1.70864
C	2.37944	0.17570	2.90512
H	1.29635	0.35478	2.83347
C	3.42150	-1.19783	-2.01862
H	4.40043	-0.97240	-2.46741
C	0.44937	-2.39129	-1.30996
O	-0.14172	-1.63959	-2.02677
O	1.10424	-3.28371	-0.94557
H	-0.23467	5.00390	-1.16398
H	2.55652	-0.46350	3.78234
H	-2.67196	-0.22747	3.76607
H	-2.64116	-1.13224	-2.50297
H	-3.07347	-2.78477	-1.99128
H	-4.35241	-1.60040	-2.37833
H	3.19616	-2.26314	-2.20173
H	2.67071	-0.58389	-2.53724
H	2.88074	1.14634	3.07560
H	-2.98094	1.26681	2.83727
C	-3.68952	1.88982	-0.49793
H	-3.73524	2.52474	-1.39087
H	-4.45186	1.10612	-0.57890
H	-3.89660	2.51901	0.38066
C	3.44591	2.25264	-0.17325
H	3.49298	2.89394	0.71894
H	4.28821	1.55153	-0.16994
H	3.52073	2.89369	-1.06019

TS1_Ni_L3

Ni	0.04450	-0.42291	-0.21323
H	0.13535	-1.78275	0.44579
P	2.13170	0.02927	0.23089
C	2.18092	1.82610	-0.29855
C	0.82926	2.33725	-0.65895
N	0.65397	3.63176	-0.87126
C	-0.57817	4.00944	-1.19239
N	-1.62567	3.20302	-1.31640
C	-1.39497	1.91738	-1.10434
N	-0.17417	1.44198	-0.76216
C	-2.49573	0.92668	-1.17493
P	-2.13353	-0.42188	0.08701
N	-3.30538	-1.60663	-0.16752
C	-4.19969	-1.72678	0.98211
H	-3.84924	-2.52145	1.66803
H	-5.21210	-1.99427	0.64171
C	-4.16914	-0.37580	1.66671
H	-4.85230	0.33924	1.16317
H	-4.46818	-0.43806	2.72370
N	-2.78015	0.06406	1.57248
C	-2.45793	1.36050	2.11822
H	-1.37419	1.54127	2.05654
C	-3.03262	-2.80728	-0.92761
N	3.59558	-0.58911	-0.34626
C	4.40976	-1.13418	0.73337
H	5.47636	-1.03026	0.48000
H	4.19775	-2.20954	0.89003
C	4.03372	-0.32344	1.95841
H	4.26044	-0.85377	2.89550
H	4.57709	0.64349	1.97439
N	2.59420	-0.13062	1.84198
C	1.87841	0.53340	2.90094
H	0.79487	0.47161	2.71547
C	3.76265	-1.12963	-1.67467
H	4.78957	-0.94951	-2.02559
C	0.61337	-2.32013	-1.37150
O	-0.03637	-1.64759	-2.11566
O	1.31978	-3.15003	-0.96166
H	-0.74528	5.07835	-1.36464
H	2.07771	0.03122	3.85867
H	-2.73907	1.39718	3.18020
H	-2.45980	-2.56948	-1.83393
H	-2.47386	-3.55951	-0.33950
H	-3.98235	-3.25938	-1.24669
H	3.56829	-2.21657	-1.71541
H	3.08327	-0.62863	-2.38130
H	2.16116	1.59853	3.01167
H	-2.98509	2.18664	1.59987
H	-3.48102	1.40661	-1.09051
H	2.65345	2.48106	0.45176
H	2.82768	1.89089	-1.19155
H	-2.45413	0.39649	-2.14388

TS1_Ni_L5

Ni	0.81869	-0.84894	-0.10728
H	1.81632	-1.93327	0.24012
P	2.21946	0.79578	0.21301
N	1.06341	2.13053	0.07754
C	-0.24053	1.80865	-0.07208
N	-1.16873	2.76175	-0.09780
C	-2.41099	2.32338	-0.21170
N	-2.82582	1.07315	-0.23588
C	-1.85225	0.15962	-0.19455
N	-0.54360	0.49550	-0.17994
N	-2.15457	-1.15415	-0.14590
P	-0.80743	-2.28057	0.17021
N	-1.21148	-3.63517	-0.71467
C	-1.42382	-4.82025	0.10821
H	-0.51335	-5.44594	0.14118
H	-2.23077	-5.43054	-0.32784
C	-1.79035	-4.30646	1.49238
H	-2.88344	-4.16365	1.59694
H	-1.47260	-5.00119	2.28442
N	-1.07773	-3.04261	1.63422
C	-1.18705	-2.30355	2.86759
H	-0.51641	-1.43165	2.84229
C	-1.03795	-3.77777	-2.14114
N	3.51077	1.31137	-0.72079
C	4.76490	1.31352	0.02261
H	5.42610	2.10013	-0.37293
H	5.28810	0.34465	-0.08626
C	4.37736	1.57169	1.46967
H	5.10214	1.13328	2.17206
H	4.31703	2.65562	1.68668
N	3.08092	0.92674	1.63675
C	2.44428	0.91815	2.93000
H	1.52538	0.31454	2.89053
C	3.54706	1.23904	-2.16235
H	4.05314	2.12627	-2.57098
C	2.26971	-1.73127	-1.65024
O	1.24970	-1.50552	-2.23066
O	3.38952	-1.97033	-1.43597
H	3.11017	0.46670	3.67973
H	-0.88343	-2.93494	3.71505

H	-0.97143	-2.79080	-2.61715
H	-0.12704	-4.34571	-2.39622
H	-1.90355	-4.30453	-2.57083
H	4.08285	0.34210	-2.51949
H	2.52511	1.22507	-2.56847
H	2.17684	1.93525	3.27027
H	-2.21663	-1.94612	3.05533
C	-3.54529	-1.58033	-0.13504
H	-4.11250	-1.03481	-0.89850
H	-3.58207	-2.65231	-0.36149
H	-4.01490	-1.39169	0.84180
C	1.45396	3.52382	0.23158
H	1.15601	3.91242	1.21611
H	2.54148	3.59507	0.11851
H	0.97528	4.13986	-0.53948
C	-3.47146	3.42593	-0.27688
F	-3.49047	4.07634	0.88275
F	-3.16722	4.28742	-1.23794
F	-4.67374	2.93742	-0.50878

TS1_Ni_L7

Ni	0.00813	-0.62221	0.05200
H	0.03279	-2.09953	0.38162
P	2.11810	-0.29847	0.30986
N	2.20551	1.40844	0.07867
C	1.01988	2.05524	-0.11844
N	0.98213	3.37085	-0.26964
C	-0.22887	3.88742	-0.43330
N	-1.37966	3.22850	-0.40736
C	-1.27620	1.91671	-0.24490
N	-0.08759	1.28424	-0.14274
N	-2.39246	1.13983	-0.15883
P	-2.14328	-0.52112	0.27040
O	-3.20613	-1.30164	-0.67701
C	-4.19276	-2.00788	0.08763
H	-3.88172	-3.05869	0.18363
H	-5.14188	-1.96454	-0.46105
C	-4.26002	-1.30367	1.43796
H	-4.99056	-0.48011	1.44391
H	-4.49030	-1.98846	2.26259
O	-2.94749	-0.76733	1.65869
O	3.23151	-0.85994	-0.73774
C	4.19427	-1.68569	-0.06880
H	5.14309	-1.61054	-0.61330
H	3.84568	-2.73014	-0.08922
C	4.27893	-1.13771	1.34929
H	4.52799	-1.90664	2.08982
H	4.99863	-0.30913	1.43233
O	2.96313	-0.64194	1.64905
C	0.43416	-2.23197	-1.52656
O	-0.19155	-1.37364	-2.07429
O	1.09694	-3.16121	-1.30615
H	-0.28464	4.96969	-0.59419
C	-3.72100	1.73425	-0.24166
H	-3.68723	2.60472	-0.90510
H	-4.41453	0.99634	-0.66294
H	-4.07144	2.05912	0.74865
C	3.47267	2.12648	0.14872
H	3.82667	2.18214	1.18770
H	4.21741	1.61286	-0.47293
H	3.32692	3.14231	-0.23215

TS1_Ni_L8

Ni	-0.70534	0.25474	0.29984
H	-2.03380	0.71094	0.75225
C	1.30383	-1.60607	-0.26210
N	2.51200	-2.04551	-0.55415
C	3.43790	-1.09274	-0.69442
N	3.24025	0.22353	-0.58710
C	2.00453	0.57950	-0.29412
N	1.03973	-0.31439	-0.10820
C	-2.16257	0.52943	-1.52736
O	-1.06473	0.73359	-1.93142
O	-3.29172	0.33125	-1.36544
H	4.45636	-1.42313	-0.92341
C	1.50517	1.98770	-0.20832
H	2.25207	2.66243	0.24401
H	1.35093	2.33440	-1.24413
C	0.07633	-2.45501	-0.14427
H	-0.25561	-2.68207	-1.17207
H	0.28871	-3.42433	0.33893
N	0.20537	2.03600	0.51659
N	-1.00626	-1.71248	0.56117
C	-0.55561	3.22179	0.09174
H	-0.79376	3.15195	-0.97610
H	-1.48691	3.27663	0.66701
H	0.03236	4.13886	0.26857
C	0.46246	2.12048	1.96838
H	0.99799	3.05611	2.20728
H	-0.49056	2.10184	2.50948
H	1.07519	1.27075	2.29847
C	-2.31362	-2.27248	0.18026
H	-2.35645	-3.34608	0.43145
H	-3.10616	-1.74169	0.71935
H	-2.48044	-2.15408	-0.89716
C	-0.82961	-1.87156	2.02069
H	0.16198	-1.51456	2.32912
H	-1.59607	-1.29022	2.54571
H	-0.92530	-2.93560	2.29948

TS1_Ni_L10

Ni	0.78630	0.11053	-0.50994
H	2.16187	0.23574	-1.02938
C	-1.54902	-1.37999	0.06009
C	-2.90386	-1.54105	0.35742
C	-3.70621	-0.40594	0.50013
C	-3.17934	0.88110	0.35234
C	-1.82175	1.02657	0.06759
C	-1.02426	-0.10176	-0.07628
C	2.34297	0.18468	1.25330
O	1.33236	0.64922	1.66925
O	3.41057	-0.27631	1.16956
H	-4.76867	-0.52686	0.72638
C	-1.07090	2.30968	-0.08911
H	-1.59594	3.06055	-0.70849
H	-0.86049	2.77615	0.89440
C	-0.55175	-2.47190	-0.16310
H	-0.47783	-3.17920	0.68467
H	-0.78270	-3.06683	-1.06850
H	-3.34478	-2.53613	0.47460
H	-3.83282	1.75225	0.46297
O	0.18850	2.00219	-0.71427
C	1.09562	3.08274	-0.69660
H	0.62789	3.97431	-1.14654
H	1.39810	3.31749	0.33892
H	1.97459	2.78714	-1.27841
O	0.73560	-1.85486	-0.34699
C	1.73102	-2.74242	-0.81439
H	1.81073	-3.60644	-0.13385

H	1.48282	-3.09877	-1.82827
H	2.67940	-2.19646	-0.83870

TS1_Ni_L11

Ni	-0.73401	0.15777	0.27231
H	-2.06285	0.41993	0.85800
C	1.48802	-1.50157	-0.31579
C	2.83594	-1.77072	-0.55521
C	3.72751	-0.69929	-0.67770
C	3.29124	0.62654	-0.57656
C	1.93965	0.87686	-0.33754
C	1.05517	-0.18641	-0.19877
C	-2.37185	0.32226	-1.40938
O	-1.34846	0.68283	-1.89196
O	-3.47929	-0.02316	-1.27759
H	4.78676	-0.90214	-0.85695
C	1.23995	2.20067	-0.26371
H	1.84817	3.01988	0.16901
H	0.94790	2.52017	-1.27819
C	0.34900	-2.47113	-0.22007
H	-0.03829	-2.69099	-1.22996
H	0.60730	-3.44503	0.24130
N	-0.02162	2.03971	0.51989
N	-0.76677	-1.83256	0.54383
C	-0.95433	3.11623	0.19373
H	-1.23955	3.05466	-0.86377
H	-1.85484	3.01801	0.81231
H	-0.49004	4.10371	0.38006
C	0.29025	2.09185	1.95365
H	0.67546	3.09403	2.22514
H	-0.61608	1.88299	2.53539
H	1.05400	1.34237	2.19624
C	-2.02776	-2.50905	0.24095
H	-1.95367	-3.58788	0.47599
H	-2.83542	-2.06033	0.83124
H	-2.27245	-2.39684	-0.82280
C	-0.49578	-1.95972	1.98338
H	0.49005	-1.53832	2.21555
H	-1.26273	-1.41902	2.55102
H	-0.50708	-3.02697	2.27779
H	3.20391	-2.79759	-0.64823
H	4.01016	1.44498	-0.68534

4.5.2. TS2

TS2_Co_L1

Co	0.40568	0.01345	0.05674
H	0.37636	-0.17627	3.02718
P	-1.31619	1.32711	0.04629
N	-0.51664	2.89865	0.19639
C	0.83703	2.88913	0.18557
N	1.50798	4.04179	0.24983
C	2.82618	3.92729	0.24702
N	3.51864	2.80059	0.18424
C	2.78510	1.68819	0.11924
N	1.43198	1.67555	0.10380
N	3.40187	0.48121	0.05796
P	2.34130	-0.91563	-0.11951
N	3.08193	-2.12765	0.80430
C	3.50566	-3.27035	0.01535
H	2.71638	-4.04814	-0.00245
H	4.40679	-3.72614	0.45950
C	3.77223	-2.73980	-1.38215
H	4.81416	-2.36796	-1.47910
H	3.63422	-3.52089	-2.14793
N	2.81060	-1.67345	-1.57260
N	-2.64810	1.45553	1.08413
C	-3.92500	1.33630	0.39811
H	-4.68199	1.95758	0.90630
H	-4.28215	0.28904	0.41845
C	-3.68573	1.79822	-1.02913
H	-4.36527	1.28908	-1.73418
H	-3.85563	2.88992	-1.13918
N	-2.31233	1.44939	-1.32221
C	-1.23635	4.15339	0.23309
H	-0.80789	4.82009	0.99226
H	-2.28132	3.94615	0.49319
H	-1.19268	4.67304	-0.73706
C	4.84633	0.40115	0.02447
H	5.24517	0.69255	-0.96020
H	5.14213	-0.63054	0.25050
H	5.28400	1.06895	0.77713
H	-0.06101	-0.55243	-1.41253
Si	-0.68141	-1.99995	-1.24157
H	-0.64068	-2.33931	-2.70520
H	0.13953	-3.00684	-0.52353
C	-4.30114	-2.46188	0.81007
C	-5.24504	-2.17507	-0.17435
C	-2.93939	-2.39320	0.52239
H	-6.31240	-2.23543	0.05400
H	-2.20479	-2.60123	1.30562
C	-4.82360	-1.81894	-1.45427
C	-2.49778	-2.04069	-0.76297
H	-5.55792	-1.59931	-2.23414
C	-3.46238	-1.75609	-1.74129
H	-3.14236	-1.49409	-2.75626
H	-4.62517	-2.74416	1.81502
C	-0.20898	-1.09301	2.71883
O	-0.69051	-1.80812	3.58074
O	-0.28948	-1.26790	1.45530
C	-2.60005	0.97562	2.44688
H	-2.82154	-0.10465	2.52203
H	-1.60613	1.15118	2.87981
H	-3.33251	1.52767	3.05626
C	-1.79294	1.75662	-2.62373
H	-0.73591	1.45786	-2.68876
H	-2.34741	1.20867	-3.40411
H	-1.85785	2.83797	-2.85749
C	2.68353	-2.38902	2.16982
H	1.80701	-3.05839	2.23103
H	3.52165	-2.84951	2.71557
H	2.42781	-1.44992	2.67810
C	2.85305	-0.93688	-2.80669
H	3.83182	-0.44242	-2.96812
H	2.66291	-1.60555	-3.66107
H	2.07337	-0.16027	-2.80746
H	3.40291	4.85866	0.29827

TS2_Co_L2

Co	0.46758	0.04763	0.05015
H	0.45287	-0.21458	3.03357
P	-1.28225	1.31262	0.10031
O	-0.51741	2.89594	0.31894
C	0.79074	2.92014	0.33944
N	1.41181	4.07881	0.49032
C	2.73564	4.01098	0.49756
N	3.47003	2.91379	0.36578
C	2.78125	1.79559	0.21852
N	1.43212	1.73890	0.19935
O	3.42940	0.66235	0.07764
P	2.43728	-0.77357	-0.15705
N	3.24965	-1.93822	0.71714
C	3.98863	-2.87604	-0.11143
H	3.40043	-3.80297	-0.25812
H	4.93760	-3.15249	0.37648
C	4.21790	-2.16113	-1.42910
H	5.12266	-1.52090	-1.37855
H	4.34589	-2.86307	-2.26767
N	3.02686	-1.34932	-1.63156
N	-2.58572	1.40735	1.13413
C	-3.86017	1.59451	0.45740
H	-4.49713	2.28395	1.03564
H	-4.38955	0.62624	0.37583
C	-3.52488	2.15591	-0.91262
H	-4.27360	1.86786	-1.66878
H	-3.47259	3.26349	-0.88955
N	-2.23114	1.59186	-1.25833
H	0.08445	-0.39085	-1.45756
Si	-0.55273	-1.86488	-1.32604
H	-0.50898	-2.09864	-2.80909
H	0.27424	-2.91715	-0.67994
C	-4.18923	-2.33342	0.69560
C	-5.11980	-1.95431	-0.26981
C	-2.82561	-2.30699	0.40846
H	-6.18877	-1.98301	-0.04263
H	-2.10207	-2.59070	1.17755
C	-4.68413	-1.54783	-1.53007
C	-2.36986	-1.90072	-0.85544
H	-5.40864	-1.25707	-2.29554
C	-3.32182	-1.52410	-1.81558
H	-2.99111	-1.21349	-2.81340
H	-4.52476	-2.65776	1.68406
C	-0.08538	-1.15056	2.69466
O	-0.54485	-1.90624	3.53125
O	-0.13792	-1.29949	1.42502
C	-2.60860	0.86712	2.47284
H	-3.02116	-0.15680	2.49512
H	-1.59678	0.83780	2.89386
H	-3.22342	1.51100	3.12050
C	-1.65930	1.97078	-2.52290
H	-0.66151	1.52240	-2.63746
H	-2.29135	1.60790	-3.34941
H	-1.55670	3.06844	-2.61856
C	2.89368	-2.34311	2.05813
H	2.17142	-3.17747	2.06270
H	3.80039	-2.65160	2.60040

H	2.44519	-1.50495	2.60434
C	3.00246	-0.50561	-2.80183
H	3.86536	0.18676	-2.82961
H	3.02072	-1.12284	-3.71251
H	2.07919	0.09223	-2.81624
H	3.27782	4.95505	0.62258

TS2_Co_L3

Co	0.50679	0.10282	0.23183
H	0.36177	-0.40205	3.16350
P	-1.16902	1.50210	0.27600
C	-0.31559	3.08596	0.76152
C	1.13402	2.98474	0.46498
N	1.85366	4.08590	0.31111
C	3.15652	3.91385	0.13033
N	3.77979	2.74327	0.18118
C	3.00875	1.67818	0.33606
N	1.65139	1.72997	0.39208
C	3.62471	0.33464	0.47589
P	2.38686	-0.94860	-0.05579
N	2.97325	-2.40610	0.60429
C	3.40650	-3.33676	-0.42368
H	2.58639	-4.02928	-0.70184
H	4.24920	-3.94641	-0.05635
C	3.80244	-2.48130	-1.61030
H	4.83011	-2.07689	-1.47628
H	3.78993	-3.04895	-2.55477
N	2.81449	-1.41928	-1.65103
N	-2.61011	1.53991	1.18496
C	-3.79022	1.64859	0.34174
H	-4.57085	2.23018	0.86071
H	-4.20780	0.64926	0.11289
C	-3.32808	2.33811	-0.92673
H	-3.99413	2.11913	-1.77854
H	-3.30708	3.44224	-0.79262
N	-2.00232	1.81242	-1.18610
H	0.09175	-0.19257	-1.31307
Si	-0.59620	-1.62653	-1.37204
H	-0.48744	-1.73689	-2.86657
H	0.14092	-2.76953	-0.77742
C	-4.31681	-2.28735	0.41713
C	-5.20650	-1.84015	-0.55732
C	-2.94143	-2.20634	0.20577
H	-6.28445	-1.91008	-0.38980
H	-2.24774	-2.54104	0.98310
C	-4.71787	-1.30834	-1.74987
C	-2.43336	-1.67778	-0.99097
H	-5.41030	-0.96020	-2.52116
C	-3.34404	-1.23081	-1.96031
H	-2.96984	-0.82319	-2.90648
H	-4.69381	-2.70543	1.35402
C	-0.24992	-1.25244	2.73420
O	-0.77795	-2.04574	3.49390
O	-0.30465	-1.27140	1.45794
C	-2.75601	0.78619	2.40994
H	-3.01216	-0.27394	2.23082
H	-1.82662	0.81320	2.99524
H	-3.54981	1.23977	3.02373
C	-1.30213	2.33647	-2.32595
H	-0.30851	1.87024	-2.41023
H	-1.85666	2.10779	-3.25059
H	-1.16594	3.43720	-2.27709
C	2.40034	-2.97893	1.80331
H	1.45587	-3.51930	1.61195
H	3.12402	-3.67619	2.25293
H	2.18801	-2.19559	2.54299
C	2.98394	-0.40971	-2.66295
H	3.95179	0.12729	-2.57736
H	2.94055	-0.86735	-3.66324
H	2.17039	0.32906	-2.59980
H	3.76554	4.80521	-0.05482
H	-0.43089	3.12490	1.86080
H	3.76374	0.13535	1.55493
H	-0.75712	4.00880	0.35774
H	4.61532	0.28185	0.00186

TS2_Co_L4

Co	0.41949	0.03598	0.07553
H	0.36399	-0.18686	3.04762
P	-1.27421	1.36780	0.05472
N	-0.46133	2.91641	0.17770
C	0.91041	2.90828	0.16213
C	1.65323	4.09628	0.20434
C	3.03628	4.01311	0.19993
C	3.66168	2.77657	0.15645
C	2.86406	1.62630	0.11266
N	1.50740	1.68982	0.10137
N	3.41739	0.36835	0.07290
P	2.30532	-0.96652	-0.11174
N	2.99613	-2.22728	0.79382
C	3.30802	-3.39804	-0.00456
H	2.45704	-4.10840	-0.00980
H	4.17641	-3.92884	0.42145
C	3.59036	-2.88683	-1.40636
H	4.65817	-2.60233	-1.52382
H	3.37577	-3.65361	-2.16958
N	2.71646	-1.74669	-1.57598
N	-2.61086	1.52159	1.08979
C	-3.88621	1.36942	0.40976
H	-4.65168	1.99089	0.90545
H	-4.23060	0.31860	0.45320
C	-3.65580	1.80337	-1.02786
H	-4.33103	1.26920	-1.71891
H	-3.84359	2.89022	-1.16158
N	-2.27890	1.46968	-1.31588
C	-1.18870	4.16104	0.20481
H	-0.89938	4.77913	1.06976
H	-2.25750	3.94306	0.30647
H	-1.03072	4.75051	-0.71481
C	4.84881	0.19454	0.06368
H	5.31002	0.60216	-0.85251
H	5.07375	-0.87590	0.12880
H	5.31708	0.68000	0.93473
H	-0.08067	-0.53235	-1.39227
Si	-0.71790	-1.96819	-1.23886
H	-0.68043	-2.31196	-2.70216
H	0.08123	-2.98967	-0.51705
C	-4.34052	-2.41441	0.80942
C	-5.28323	-2.11100	-0.17117
C	-2.97858	-2.35411	0.52057
H	-6.35083	-2.16413	0.05800
H	-2.24485	-2.57399	1.30133
C	-4.86018	-1.74745	-1.44857
C	-2.53544	-1.99351	-0.76192
H	-5.59360	-1.51461	-2.22557
C	-3.49880	-1.69296	-1.73649
H	-3.17755	-1.42369	-2.74923
H	-4.66561	-2.70252	1.81237
C	-0.24219	-1.08438	2.72467
O	-0.74191	-1.80147	3.57578
O	-0.32486	-1.23807	1.45928
C	-2.55788	1.07862	2.46387
H	-2.80042	0.00553	2.57110
H	-1.55475	1.24386	2.87994
H	-3.27213	1.66069	3.06758
C	-1.75742	1.79520	-2.61150

H	-0.69879	1.50186	-2.67711
H	-2.30712	1.25594	-3.40139
H	-1.82541	2.88003	-2.83159
C	2.60824	-2.46353	2.16649
H	1.69709	-3.08316	2.24541
H	3.42837	-2.96917	2.70003
H	2.41312	-1.51029	2.67586
C	2.79764	-1.00653	-2.80471
H	3.81192	-0.59593	-2.98424
H	2.53439	-1.64745	-3.66146
H	2.08814	-0.16536	-2.78344
H	3.63617	4.92531	0.22985
H	1.15197	5.06158	0.23726
H	4.74704	2.69898	0.15007

TS2_Co_L5

Co	0.25422	0.44958	0.03149
H	0.27783	0.50110	3.00692
P	0.96900	-1.59700	-0.00878
N	-0.57400	-2.46586	0.09716
C	-1.69112	-1.70841	0.08463
N	-2.89102	-2.29919	0.11334
C	-3.91418	-1.47162	0.10785
N	-3.87721	-0.15221	0.07253
C	-2.65242	0.37374	0.04249
N	-1.51717	-0.36631	0.03714
N	-2.50048	1.71798	0.00704
P	-0.83881	2.30118	-0.13152
N	-0.80449	3.69891	0.82068
C	-0.52077	4.90388	0.06142
H	0.56469	5.12622	0.07267
H	-1.03800	5.76744	0.51249
C	-1.00344	4.63341	-1.35300
H	-2.07496	4.89910	-1.47045
H	-0.44025	5.22217	-2.09553
N	-0.78478	3.21665	-1.56507
N	1.97798	-2.46555	1.03123
C	3.10415	-3.09471	0.35999
H	3.34245	-4.05429	0.84974
H	4.00266	-2.45370	0.42671
C	2.68386	-3.29383	-1.08726
H	3.55040	-3.23378	-1.76795
H	2.21264	-4.28659	-1.24336
N	1.75284	-2.22297	-1.37393
C	-0.66810	-3.91074	0.09852
H	-1.42026	-4.24674	0.82311
H	0.30763	-4.32047	0.38557
H	-0.95438	-4.29692	-0.89218
C	-3.66032	2.58405	-0.03502
H	-4.12855	2.58292	-1.03187
H	-3.34212	3.60166	0.22160
H	-4.41182	2.25285	0.69247
H	0.98649	0.69430	-1.40651
Si	2.30149	1.55362	-1.16145
H	2.48930	1.89257	-2.61317
H	2.15158	2.82864	-0.41662
C	5.50468	-0.14751	0.92905
C	6.15764	-0.87643	-0.06328
C	4.34481	0.56543	0.63219
H	7.07045	-1.42963	0.17277
H	3.82872	1.12018	1.42099
C	5.64852	-0.89002	-1.36064
C	3.82056	0.56512	-0.67030
H	6.15962	-1.45278	-2.14650
C	4.49244	-0.17254	-1.65687
H	4.11211	-0.17430	-2.68475
H	5.90128	-0.12979	1.94739
C	1.28301	0.94939	2.74774
O	2.05114	1.24639	3.64553
O	1.48890	1.09510	1.49390
C	2.17072	-2.08446	2.41178
H	2.96441	-1.32527	2.53100
H	1.24095	-1.67581	2.82939
H	2.44115	-2.97262	3.00376
C	1.18608	-2.14689	-2.69017
H	0.48204	-1.30352	-2.75040
H	1.97401	-1.98363	-3.44434
H	0.63874	-3.07016	-2.96506
C	-0.36738	3.67462	2.19922
H	0.72930	3.76522	2.29295
H	-0.84099	4.50213	2.74972
H	-0.67126	2.73513	2.67911
C	-1.19395	2.64830	-2.82137
H	-2.27769	2.78187	-3.00975
H	-0.64224	3.11456	-3.65268
H	-0.97450	1.57007	-2.83503
C	-5.31508	-2.07024	0.11982
F	-5.29772	-3.38839	0.23620
F	-6.02728	-1.57458	1.13038
F	-5.95272	-1.76429	-1.01264

TS2_Co_L6

Co	0.44012	-0.21036	-0.38593
H	-0.27779	-0.77067	3.22507
P	-0.46420	1.73899	-0.37511
N	0.90463	2.72215	0.14881
C	2.07531	2.05693	0.30033
N	3.17551	2.73239	0.64350
C	4.27762	2.00402	0.72613
N	4.39212	0.70875	0.47540
C	3.25350	0.09750	0.13942
N	2.05658	0.72264	0.07655
N	3.27102	-1.22181	-0.17282
P	1.71864	-1.90706	-0.65043
C	0.87447	4.15479	0.36309
H	1.24827	4.40750	1.36484
H	-0.15736	4.51437	0.27300
H	1.50074	4.68281	-0.37152
C	4.53047	-1.93736	-0.14089
H	5.24338	-1.52117	-0.86783
H	4.35645	-2.99260	-0.38211
H	4.99221	-1.87482	0.85447
H	-0.85942	-0.78352	-1.01862
Si	-1.72999	-1.92195	-0.08428
H	-1.56814	-2.71035	-1.38228
H	-1.87627	-3.11242	0.82709
C	-5.35035	0.05087	0.63225
C	-5.63229	0.63704	-0.59947
C	-4.20928	-0.73539	0.79024
H	-6.52408	1.25784	-0.72112
H	-3.98025	-1.17832	1.76458
C	-4.77580	0.42542	-1.67973
C	-3.33177	-0.94999	-0.28099
H	-4.99813	0.87439	-2.65186
C	-3.64001	-0.36338	-1.51658
H	-2.96880	-0.52617	-2.36877
H	-6.02219	0.20975	1.47973
C	-1.13894	-1.15583	2.62385
O	-2.12559	-1.60667	3.15354
O	-0.92020	-1.02814	1.34399
H	5.19299	2.52949	1.02475
C	2.07781	-2.67676	-2.27054
H	2.86110	-3.44804	-2.22017
H	1.14441	-3.14457	-2.61965
H	2.36088	-1.89691	-2.98955
C	-1.06287	2.61740	-1.86334

H	-0.31953	2.53587	-2.66683
H	-1.98651	2.11157	-2.18435
H	-1.29519	3.67509	-1.66821
C	-1.76261	2.17187	0.83671
H	-2.66240	1.58856	0.58424
H	-1.43883	1.87598	1.84361
H	-2.01815	3.24133	0.82401
C	1.59852	-3.38646	0.42479
H	0.68819	-3.93832	0.14187
H	2.45857	-4.06485	0.32623
H	1.49049	-3.06816	1.47078

TS2_Co_L7

Co	0.42636	-0.07743	-0.23165
H	0.48658	-0.35812	2.73108
P	-1.01151	1.46585	0.06358
N	-0.04641	2.90602	0.04859
C	1.29487	2.71303	-0.06780
N	2.12622	3.75166	-0.05262
C	3.41092	3.45018	-0.14801
N	3.92515	2.23289	-0.22904
C	3.03892	1.24269	-0.23756
N	1.69891	1.42677	-0.18796
N	3.46055	-0.05363	-0.28868
P	2.20197	-1.22623	-0.18211
O	2.68040	-2.11173	1.12305
C	2.67791	-3.49598	0.81271
H	1.66128	-3.90346	0.94786
H	3.36158	-4.01002	1.50045
C	3.13222	-3.57274	-0.63980
H	4.23261	-3.58277	-0.71800
H	2.72921	-4.44953	-1.16422
O	2.62771	-2.39779	-1.26478
O	-1.91132	1.68094	1.42036
C	-3.30607	1.70971	1.14446
H	-3.78900	2.36826	1.87867
H	-3.72062	0.69415	1.24319
C	-3.43560	2.23379	-0.28177
H	-4.29989	1.79937	-0.80256
H	-3.51055	3.33390	-0.30959
O	-2.25236	1.82678	-0.95839
C	-0.62983	4.22701	0.17498
H	0.16752	4.95866	0.34060
H	-1.31885	4.24568	1.03097
H	-1.17917	4.49603	-0.73926
C	4.86424	-0.41714	-0.31816
H	5.06001	-1.07696	-1.17513
H	5.14539	-0.93651	0.60911
H	5.46609	0.49170	-0.41858
H	-0.18033	-0.51692	-1.69316
Si	-1.00936	-1.85720	-1.53276
H	-1.12280	-2.13191	-3.00051
H	-0.30138	-3.00171	-0.90966
C	-4.51602	-1.86068	0.75475
C	-5.43664	-1.26805	-0.10788
C	-3.18986	-2.02853	0.36096
H	-6.47711	-1.14094	0.20317
H	-2.47015	-2.46605	1.05675
C	-5.02949	-0.84855	-1.37369
C	-2.76369	-1.61167	-0.90964
H	-5.74878	-0.39350	-2.06033
C	-3.70561	-1.02453	-1.76848
H	-3.39928	-0.69996	-2.76905
H	-4.82881	-2.19594	1.74664
C	-0.24522	-1.16366	2.42500
O	-0.79645	-1.82473	3.28829
O	-0.40246	-1.28214	1.16215
H	4.11755	4.28789	-0.15525

TS2_Co_L9

Co	0.28896	-0.33770	0.01054
H	0.17617	-0.62986	2.96160
P	-0.64905	1.56521	0.44682
N	0.61427	2.73916	0.33888
C	1.83807	2.20709	-0.09059
C	2.95043	3.03429	-0.31759
C	4.15853	2.45921	-0.70431
C	4.26894	1.07923	-0.85099
C	3.13692	0.27743	-0.63298
C	1.88080	0.80728	-0.27090
N	3.20466	-1.12841	-0.73921
P	1.81185	-1.85473	-0.01755
C	0.45760	4.14331	0.56512
H	1.19209	4.52013	1.30066
H	-0.54253	4.35537	0.96605
H	0.58050	4.73892	-0.36112
C	4.48046	-1.77535	-0.83257
H	5.00339	-1.49713	-1.76371
H	4.35445	-2.86617	-0.84734
H	5.15022	-1.51952	0.01426
H	-0.12624	-0.35797	-1.55285
Si	-1.32791	-1.45623	-1.49925
H	-1.39557	-1.66668	-2.99574
H	-1.17133	-2.83690	-0.95144
C	-4.88728	-0.38456	0.46078
C	-5.64522	0.19807	-0.55550
C	-3.62029	-0.89697	0.19134
H	-6.63722	0.60577	-0.33890
H	-3.02246	-1.33690	0.99668
C	-5.13123	0.25149	-1.84971
C	-3.07809	-0.84042	-1.10503
H	-5.71893	0.70089	-2.65604
C	-3.86503	-0.26688	-2.11532
H	-3.47185	-0.21822	-3.13724
H	-5.28446	-0.43889	1.47854
C	-0.71248	-1.24439	2.63091
O	-1.48054	-1.67734	3.48358
O	-0.80040	-1.40709	1.37098
H	5.03062	3.09612	-0.88163
C	1.66207	-3.44768	-0.92241
H	2.57168	-4.06515	-0.86766
H	0.83005	-4.00677	-0.46748
H	1.41180	-3.24941	-1.97464
C	-1.94686	2.31971	-0.62496
H	-1.64253	2.21981	-1.67700
H	-2.89593	1.78431	-0.48120
H	-2.10160	3.38423	-0.38917
C	-1.41806	1.86615	2.09023
H	-1.82924	2.88273	2.18600
H	-2.23669	1.14141	2.22179
H	-0.67539	1.68843	2.87977
C	2.41184	-2.47986	1.60923
H	1.56841	-2.94830	2.13764
H	3.22677	-3.21390	1.50665
H	2.75787	-1.61911	2.19991
H	5.22898	0.64016	-1.13221
H	2.88431	4.11768	-0.19122

TS2_Co_L10

Co	-0.47110	0.23201	-0.19608
H	-1.16346	1.33196	2.24754
C	-2.23068	-1.94258	0.31992
C	-3.45877	-2.59108	0.41496
C	-4.62179	-1.91890	0.02005
C	-4.55062	-0.59748	-0.43430

C	-3.31441	0.04381	-0.50754
C	-2.13838	-0.62909	-0.15881
H	-0.14592	-0.47472	-1.53120
Si	1.32229	0.50571	-1.77009
H	1.49851	-0.01647	-3.17719
H	1.33666	2.00138	-1.96673
C	4.60638	0.26674	0.83216
C	5.32497	-0.82798	0.34758
C	3.44804	0.69180	0.18672
H	6.23037	-1.16499	0.86144
H	2.85950	1.51650	0.60338
C	4.87841	-1.48689	-0.79696
C	2.97845	0.03888	-0.96765
H	5.43590	-2.34388	-1.18853
C	3.72463	-1.05016	-1.44525
H	3.38682	-1.57475	-2.34687
H	4.94689	0.78879	1.73145
C	-0.19156	1.89149	2.09176
O	0.12604	2.75347	2.90338
O	0.45161	1.53569	1.04902
H	-5.59150	-2.42082	0.08675
H	-5.47113	-0.06953	-0.71143
H	-3.52438	-3.61590	0.79915
C	-3.12215	1.48534	-0.86433
H	-3.61613	1.77654	-1.81444
H	-3.53924	2.14195	-0.07055
C	-0.89934	-2.50765	0.69753
H	-0.94193	-3.22023	1.54517
H	-0.42337	-3.03143	-0.16156
O	-1.72576	1.73784	-0.97258
O	-0.06871	-1.40274	1.04663
C	-1.39194	3.10026	-0.89190
H	-1.68295	3.51881	0.08817
H	-0.30551	3.18649	-1.00168
H	-1.90129	3.66379	-1.69448
C	1.25160	-1.76344	1.36094
H	1.76164	-2.20113	0.48236
H	1.78126	-0.85374	1.66578
H	1.25454	-2.49545	2.18818

TS2_Co_L11

Co	-0.55139	0.35706	0.00228
H	0.10649	2.14692	2.47740
N	-0.02917	-1.24098	1.34237
C	-0.69974	-2.44230	0.79849
C	-2.04306	-2.04698	0.26303
C	-3.20305	-2.81650	0.24426
C	-4.37888	-2.28358	-0.30359
C	-4.38658	-0.97987	-0.81902
C	-3.21730	-0.22348	-0.79303
C	-2.02988	-0.74051	-0.25110
C	-3.02853	1.17016	-1.31339
N	-1.99233	1.85011	-0.50476
H	-0.04078	-0.23491	-1.44212
Si	1.23910	0.57063	-1.91367
H	1.31890	0.18018	-3.37390
H	1.22366	2.05935	-1.91946
C	4.75546	-0.05314	0.30651
C	5.20442	-1.28051	-0.18011
C	3.60215	0.53447	-0.21003
H	6.10604	-1.74203	0.23411
H	3.23761	1.47656	0.21097
C	4.49562	-1.91542	-1.20020
C	2.86770	-0.09576	-1.22805
H	4.84035	-2.87687	-1.59336
C	3.34696	-1.32210	-1.71700
H	2.79725	-1.83102	-2.51803
H	5.30279	0.45170	1.10776
C	1.01260	2.19496	1.79708
O	2.01636	2.75872	2.22427
O	0.85444	1.64713	0.66593
H	-5.29650	-2.87966	-0.31227
H	-0.74190	-3.24634	1.56719
H	-3.96393	1.77315	-1.33524
H	-0.05907	-2.81051	-0.02468
H	-2.64247	1.13310	-2.34912
C	-1.46976	3.01361	-1.19848
H	-0.66304	3.46197	-0.60286
H	-1.05018	2.71321	-2.16791
H	-2.26384	3.77184	-1.36951
C	1.39184	-1.48703	1.53552
H	1.86081	-1.76140	0.58226
H	1.88069	-0.57464	1.90331
H	1.55742	-2.30439	2.26914
C	-2.55945	2.26761	0.77253
H	-1.76943	2.68137	1.41242
H	-3.34064	3.04630	0.61917
H	-3.01400	1.40620	1.27766
C	-0.62719	-0.89023	2.62611
H	-0.43749	-1.69022	3.37665
H	-0.19183	0.04643	2.99638
H	-1.71101	-0.76170	2.51336
H	-3.20797	-3.83145	0.66056
H	-5.31375	-0.56346	-1.23206

TS2_Fe_L2

Fe	0.44268	-0.03817	-0.10137
H	0.84490	-1.44630	2.59948
P	-1.25692	1.08452	0.53357
O	-0.64997	2.78267	0.40553
C	0.57555	2.90265	-0.01137
N	1.09657	4.11902	-0.14030
C	2.35834	4.15257	-0.54316
N	3.14572	3.10678	-0.75652
C	2.56607	1.92494	-0.58483
N	1.25617	1.75918	-0.27880
O	3.29035	0.84458	-0.67970
P	2.47984	-0.65704	-0.09782
N	3.56027	-1.14929	1.12265
C	4.59215	-2.07722	0.73239
H	4.37475	-3.09339	1.11985
H	5.57320	-1.77367	1.14358
C	4.60027	-2.05460	-0.78715
H	5.30335	-1.27848	-1.16246
H	4.91663	-3.02155	-1.21492
N	3.24433	-1.74560	-1.18396
N	-2.16027	1.09115	1.97198
C	-3.58374	1.28397	1.80777
H	-3.99447	1.90712	2.62305
H	-4.10920	0.30665	1.82994
C	-3.74497	1.94661	0.45365
H	-4.73190	1.73537	0.00632
H	-3.64176	3.05173	0.54273
N	-2.69220	1.39309	-0.36312
H	0.28889	-0.04124	-1.64952
Si	-0.63011	-1.48093	-1.70201
H	-0.73153	-1.28021	-3.20292
H	0.10681	-2.77421	-1.56874
C	-4.32515	-2.15207	0.24053
C	-5.24844	-1.70811	-0.70611
C	-2.96075	-2.13292	-0.03640
H	-6.31958	-1.71786	-0.48195
H	-2.24971	-2.47412	0.71969
C	-4.79272	-1.26654	-1.94587
C	-2.47708	-1.66531	-1.27108
H	-5.50466	-0.92806	-2.70545

C	-3.42613	-1.25758	-2.22013
H	-3.08561	-0.91044	-3.20221
H	-4.66983	-2.51923	1.21220
C	0.08131	-2.07940	2.06689
O	-0.43746	-3.01314	2.66517
O	-0.16014	-1.71928	0.86902
C	-1.71395	0.43563	3.16969
H	-2.18899	-0.55517	3.29928
H	-0.62612	0.28471	3.12522
H	-1.94212	1.04904	4.05908
C	-2.58948	1.91272	-1.69522
H	-1.71033	1.48209	-2.19961
H	-3.48415	1.64247	-2.28118
H	-2.48143	3.01657	-1.70451
C	3.46846	-0.74929	2.49296
H	3.31992	-1.61569	3.16446
H	4.38055	-0.21584	2.82002
H	2.60930	-0.07197	2.61926
C	3.03669	-1.59759	-2.59792
H	3.72200	-0.84516	-3.03913
H	3.19857	-2.55916	-3.11262
H	2.00574	-1.27479	-2.79899
H	2.79989	5.14429	-0.70404

TS2_Fe_L6

Fe	0.30865	-0.31527	-0.14268
H	0.54092	-0.25321	2.82190
P	-0.69307	1.60946	0.02202
N	0.65876	2.72417	-0.07867
C	1.89723	2.15025	-0.17943
N	2.97023	2.92917	-0.22383
C	4.14036	2.30046	-0.30351
N	4.29540	0.97870	-0.32116
C	3.17734	0.26741	-0.27375
N	1.91297	0.78426	-0.22486
N	3.25500	-1.10053	-0.27092
P	1.71798	-1.93555	-0.19819
C	0.57237	4.15963	0.02204
H	1.12438	4.53384	0.89915
H	-0.47887	4.46015	0.11652
H	1.00164	4.65000	-0.86593
C	4.55494	-1.72219	-0.29130
H	5.10361	-1.48297	-1.21677
H	4.44226	-2.81193	-0.22046
H	5.17399	-1.37788	0.55198
H	-0.53410	-0.65570	-1.62160
Si	-1.46064	-1.77873	-1.05937
H	-1.65618	-2.37428	-2.44277
H	-1.24172	-3.01032	-0.22670
C	-4.88015	-0.19630	0.81807
C	-5.65971	0.22204	-0.26089
C	-3.65432	-0.82357	0.60385
H	-6.61867	0.72013	-0.08834
H	-3.03556	-1.12560	1.45568
C	-5.20900	-0.00326	-1.56009
C	-3.17556	-1.05002	-0.69855
H	-5.81324	0.31613	-2.41480
C	-3.98442	-0.63614	-1.76852
H	-3.64014	-0.80777	-2.79544
H	-5.22784	-0.02905	1.84184
C	-0.45296	-0.77226	2.66578
O	-1.16170	-0.99906	3.64126
O	-0.70747	-1.05267	1.45131
H	5.04502	2.91736	-0.35119
C	-1.54982	2.14309	1.56364
H	-1.88982	3.18933	1.52907
H	-2.42386	1.48754	1.70074
H	-0.88165	1.99517	2.42322

C	1.91806	-3.08271	1.22971
H	2.10733	-2.49178	2.13635
H	0.95708	-3.60310	1.36418
H	2.71654	-3.82792	1.09214
C	-1.86011	2.30915	-1.21977
H	-1.47492	2.10467	-2.22824
H	-2.82532	1.79267	-1.09992
H	-2.02391	3.39080	-1.09441
C	1.87623	-3.15151	-1.57973
H	2.79887	-3.75094	-1.53060
H	1.01415	-3.83569	-1.52658
H	1.83436	-2.61000	-2.53507

TS2_Fe_L7

Fe	0.37442	-0.11065	-0.02055
H	0.19989	-0.85096	2.84240
P	-1.00098	1.37412	0.56763
N	-0.20691	2.87281	0.16111
C	1.09031	2.73637	-0.21457
N	1.82751	3.81934	-0.45431
C	3.09721	3.57879	-0.75509
N	3.68516	2.39192	-0.76927
C	2.88854	1.35479	-0.51179
N	1.54841	1.45915	-0.30156
N	3.38981	0.09742	-0.42219
P	2.21232	-1.11728	0.02497
O	3.01045	-1.88816	1.30806
C	3.14644	-3.26819	1.07669
H	2.21225	-3.79407	1.35193
H	3.96732	-3.66338	1.69433
C	3.41875	-3.39579	-0.41546
H	4.49861	-3.27559	-0.63150
H	3.09182	-4.36759	-0.81790
O	2.68138	-2.36737	-1.02168
O	-1.72788	1.74704	2.04405
C	-3.08945	1.37595	2.01924
H	-3.62452	1.92247	2.80946
H	-3.19383	0.29098	2.20777
C	-3.58144	1.73077	0.61920
H	-4.38697	1.06004	0.28061
H	-3.94711	2.77467	0.57659
O	-2.46877	1.58212	-0.22841
C	-0.79985	4.18332	0.27070
H	-0.32314	4.77405	1.06763
H	-1.86564	4.07059	0.49963
H	-0.69092	4.73650	-0.67295
C	4.78369	-0.19780	-0.64556
H	5.33973	0.74022	-0.75507
H	4.91115	-0.80305	-1.55688
H	5.18766	-0.76006	0.20957
H	-0.18881	0.07635	-1.45336
Si	-0.68037	-1.57828	-1.55697
H	-0.50721	-1.55562	-3.05681
H	-0.20636	-2.93611	-1.14347
C	-4.61846	-1.88652	-0.08361
C	-5.37336	-1.26547	-1.07855
C	-3.23526	-2.00446	-0.21678
H	-6.45897	-1.17460	-0.97255
H	-2.64949	-2.47308	0.57997
C	-4.73284	-0.76273	-2.21025
C	-2.57031	-1.50406	-1.34902
H	-5.31308	-0.27052	-2.99694
C	-3.35078	-0.88636	-2.33718
H	-2.85987	-0.48006	-3.22924
H	-5.11024	-2.28509	0.80901
C	-0.49298	-1.59745	2.36737
O	-1.11968	-2.38085	3.06926
O	-0.54723	-1.53202	1.09025

H	3.72253	4.44633	-0.99986

TS2_Fe_L9

Fe	0.28914	-0.31446	-0.14521
H	0.55353	-0.33096	2.86760
P	-0.60986	1.63003	0.00349
N	0.68092	2.79539	-0.12418
C	1.95064	2.19268	-0.18328
C	3.11923	2.96947	-0.20016
C	4.36811	2.34236	-0.26155
C	4.43510	0.94555	-0.30070
C	3.25077	0.19406	-0.27972
C	1.94729	0.76829	-0.22401
N	3.28309	-1.21231	-0.31568
P	1.68938	-1.90827	-0.24078
C	0.54816	4.20351	0.04254
H	1.04014	4.57031	0.96930
H	-0.51342	4.48385	0.10014
H	0.99242	4.76979	-0.80191
C	4.51544	-1.92423	-0.29903
H	5.14668	-1.70035	-1.18486
H	4.33024	-3.00836	-0.29411
H	5.12745	-1.68899	0.59780
H	-0.57040	-0.66736	-1.61149
Si	-1.50643	-1.76927	-1.02377
H	-1.72640	-2.40618	-2.39696
H	-1.43472	-3.01869	-0.16609
C	-4.92065	-0.12814	0.81989
C	-5.70274	0.29766	-0.25886
C	-3.70655	-0.77526	0.60408
H	-6.65316	0.81428	-0.08558
H	-3.08296	-1.07664	1.45428
C	-5.25255	0.06302	-1.55751
C	-3.22964	-1.02167	-0.69911
H	-5.85001	0.39555	-2.41406
C	-4.03736	-0.58973	-1.76641
H	-3.69447	-0.76581	-2.79374
H	-5.25813	0.05518	1.84553
C	-0.44515	-0.83212	2.68886
O	-1.13627	-1.13203	3.66937
O	-0.72279	-1.01330	1.46870
H	5.28657	2.94021	-0.27321
C	-1.46316	2.15980	1.57006
H	-1.83794	3.19633	1.53557
H	-2.30944	1.47695	1.74536
H	-0.75896	2.04978	2.40759
C	1.83876	-3.11320	1.17038
H	2.06467	-2.54629	2.08506
H	0.85499	-3.59093	1.30271
H	2.60395	-3.89296	1.01686
C	-1.84935	2.32160	-1.19320
H	-1.50375	2.10547	-2.21413
H	-2.80937	1.80672	-1.02817
H	-2.00606	3.40731	-1.07500
C	1.76214	-3.15514	-1.62425
H	2.67354	-3.77733	-1.60709
H	0.88409	-3.81697	-1.53774
H	1.70176	-2.61226	-2.57857
H	5.41316	0.45468	-0.34519
H	3.06921	4.06282	-0.16573

TS2_Ni_L1

Ni	0.45318	0.06957	0.02118
H	0.28203	-0.18218	2.95415
P	-1.27397	1.43121	0.02752
N	-0.40082	2.94916	0.17889
C	0.95016	2.89190	0.15825
N	1.66469	4.01285	0.21507
C	2.97871	3.85164	0.20720
N	3.63256	2.70008	0.14818
C	2.86796	1.61498	0.08847
N	1.51503	1.66338	0.07457
N	3.44230	0.38868	0.03289
P	2.36523	-0.98160	-0.12651
N	2.97183	-2.17823	0.86189
C	3.24711	-3.41232	0.13230
H	2.37901	-4.09507	0.18468
H	4.10382	-3.92745	0.59347
C	3.54144	-2.99739	-1.30070
H	4.61673	-2.77587	-1.44616
H	3.26647	-3.78545	-2.01827
N	2.73194	-1.80810	-1.53536
N	-2.56560	1.54082	1.07259
C	-3.85138	1.38934	0.39271
H	-4.60048	2.02200	0.89393
H	-4.19597	0.34163	0.45299
C	-3.62614	1.81722	-1.04887
H	-4.28100	1.26103	-1.73834
H	-3.82396	2.89699	-1.19319
N	-2.23251	1.50782	-1.33753
H	-0.14441	-0.62360	-1.31724
Si	-0.81381	-2.05964	-1.22473
H	-0.77441	-2.32148	-2.69759
H	0.06750	-3.02000	-0.52902
C	-4.40240	-2.34314	0.88811
C	-5.34263	-2.05037	-0.09877
C	-3.04175	-2.31635	0.59312
H	-6.40932	-2.08143	0.13563
H	-2.31262	-2.53961	1.37655
C	-4.92246	-1.73466	-1.38957
C	-2.60284	-2.00152	-0.70331
H	-5.65662	-1.52033	-2.17005
C	-3.56270	-1.71585	-1.68772
H	-3.24497	-1.49717	-2.71335
H	-4.72967	-2.60370	1.89720
C	-0.30123	-1.09326	2.62965
O	-0.84917	-1.78485	3.45851
O	-0.30626	-1.29720	1.35639
C	-2.50833	1.20164	2.47909
H	-2.77713	0.14694	2.65851
H	-1.50001	1.37969	2.87642
H	-3.20587	1.84311	3.03700
C	-1.71279	1.76880	-2.65477
H	-0.65208	1.48164	-2.71126
H	-2.26096	1.18095	-3.40726
H	-1.79286	2.83743	-2.92631
C	2.68801	-2.27340	2.27947
H	1.79118	-2.88248	2.48127
H	3.54877	-2.72560	2.79268
H	2.53355	-1.27247	2.70496
C	2.79472	-1.15622	-2.82065
H	3.81143	-0.78803	-3.05070
H	2.49188	-1.85278	-3.61614
H	2.10513	-0.29893	-2.84598
H	3.58749	4.76171	0.25279
C	4.89162	0.26263	0.00186
H	5.34107	0.84150	0.81794
H	5.15234	-0.79388	0.13096
H	5.29918	0.63338	-0.94937
C	-1.08110	4.23498	0.19183
H	-1.02897	4.71935	-0.79435
H	-2.12893	4.07144	0.46840
H	-0.61809	4.90076	0.92954

TS2_Ni_L2

Ni	0.53090	0.13567	0.02208
H	0.39787	-0.18942	2.97312

P	-1.20587	1.47353	0.05054
O	-0.34107	2.98669	0.22679
C	0.96798	2.95154	0.22595
N	1.65027	4.07185	0.33863
C	2.97085	3.93706	0.34120
N	3.64685	2.79722	0.24762
C	2.90978	1.71366	0.13724
N	1.55727	1.73895	0.10749
O	3.49076	0.54022	0.05085
P	2.46412	-0.85927	-0.14010
N	3.13493	-2.01051	0.82306
C	3.72044	-3.10929	0.05354
H	3.01564	-3.95917	0.01417
H	4.64161	-3.45359	0.54702
C	3.99718	-2.54694	-1.33042
H	4.99064	-2.06225	-1.37528
H	3.95908	-3.32267	-2.10864
N	2.94084	-1.55981	-1.56654
N	-2.45522	1.59964	1.11157
C	-3.75751	1.63890	0.44159
H	-4.41841	2.33528	0.97937
H	-4.21852	0.63561	0.46430
C	-3.49081	2.10102	-0.98245
H	-4.20338	1.65629	-1.69382
H	-3.55210	3.20087	-1.07352
N	-2.13932	1.64196	-1.30136
H	-0.05384	-0.55469	-1.32344
Si	-0.71072	-1.99723	-1.24292
H	-0.65173	-2.24103	-2.71700
H	0.18167	-2.94448	-0.54187
C	-4.33557	-2.13779	0.82847
C	-5.24706	-1.80073	-0.17125
C	-2.97173	-2.18299	0.55050
H	-6.31684	-1.77692	0.04989
H	-2.26595	-2.44221	1.34387
C	-4.79589	-1.51316	-1.45821
C	-2.50176	-1.89474	-0.74101
H	-5.50857	-1.26746	-2.24914
C	-3.43348	-1.56469	-1.73907
H	-3.09298	-1.36641	-2.76147
H	-4.68857	-2.37900	1.83376
C	-0.21454	-1.07264	2.62507
O	-0.78820	-1.76556	3.43260
O	-0.22270	-1.24596	1.34524
C	-2.41210	1.25042	2.51668
H	-2.78215	0.22673	2.69184
H	-1.38601	1.32271	2.89941
H	-3.03155	1.95808	3.08551
C	-1.61421	1.86877	-2.62614
H	-0.59212	1.47032	-2.70636
H	-2.23747	1.35304	-3.37207
H	-1.58567	2.94307	-2.87642
C	2.84325	-2.21120	2.22844
H	2.06613	-2.97792	2.37837
H	3.76026	-2.52221	2.74814
H	2.50208	-1.27372	2.68533
C	2.94204	-0.83229	-2.81738
H	3.87715	-0.26261	-2.95902
H	2.82577	-1.53230	-3.65643
H	2.09715	-0.12820	-2.84751
H	3.56117	4.85521	0.43044

TS2_Ni_L3

Ni	0.46137	0.09911	0.06903
H	0.78909	0.57818	2.88379
P	-1.22811	1.52577	0.04419
C	-0.33210	3.12692	0.37366
C	1.13427	2.99319	0.17016

N	1.88300	4.07885	0.05577
C	3.19213	3.87686	-0.02311
N	3.79249	2.69807	0.09467
C	2.99266	1.65102	0.20958
N	1.64660	1.74584	0.16623
C	3.56611	0.29896	0.44263
P	2.35911	-1.01680	-0.07925
N	2.83683	-2.42397	0.69566
C	3.23359	-3.45776	-0.25569
H	2.38821	-4.13523	-0.48084
H	4.04246	-4.06460	0.17972
C	3.68872	-2.71737	-1.49825
H	4.73906	-2.37622	-1.39176
H	3.62721	-3.34131	-2.40242
N	2.77795	-1.58215	-1.61810
N	-2.60578	1.60453	0.99705
C	-3.82543	1.55113	0.19448
H	-4.60297	2.16033	0.68088
H	-4.20097	0.51583	0.11581
C	-3.44545	2.10554	-1.16536
H	-4.10758	1.73187	-1.96212
H	-3.49592	3.21369	-1.17291
N	-2.08259	1.64520	-1.40383
H	-0.02125	-0.40399	-1.39947
Si	-0.69969	-1.84421	-1.29072
H	-0.58396	-2.12363	-2.75081
H	0.12163	-2.79029	-0.49927
C	-4.18309	-2.16991	0.92843
C	-5.17778	-1.99703	-0.03261
C	-2.84052	-2.08177	0.56941
H	-6.22961	-2.08030	0.25042
H	-2.06196	-2.21003	1.32825
C	-4.83338	-1.72867	-1.35676
C	-2.47999	-1.81931	-0.76188
H	-5.61280	-1.60200	-2.11167
C	-3.49221	-1.64026	-1.71753
H	-3.22903	-1.45321	-2.76452
H	-4.45379	-2.38519	1.96476
C	0.19283	-0.38635	2.88823
O	-0.11351	-0.89057	3.94385
O	-0.09021	-0.84404	1.71714
C	-2.67411	1.15982	2.37330
H	-2.88100	0.07964	2.45668
H	-1.73290	1.36651	2.90042
H	-3.47237	1.71331	2.88832
C	-1.44378	2.03172	-2.63638
H	-0.42071	1.62805	-2.68355
H	-2.00090	1.62428	-3.49383
H	-1.38781	3.13075	-2.75883
C	2.37249	-2.84240	2.00428
H	1.40725	-3.37399	1.95948
H	3.12552	-3.50632	2.45198
H	2.24577	-1.98110	2.67176
C	2.99550	-0.65575	-2.70399
H	3.97887	-0.14935	-2.64386
H	2.94935	-1.19019	-3.66351
H	2.20607	0.11144	-2.71465
H	3.82953	4.75361	-0.18059
H	-0.49883	3.35445	1.44302
H	3.68667	0.16715	1.53448
H	-0.74926	3.97796	-0.18633
H	4.56662	0.18996	-0.00160

TS2_Ni_L6

Ni	0.40464	-0.29429	-0.02467
H	0.29129	-0.54761	2.91939
P	-0.66422	1.63495	0.17733
N	0.68901	2.71624	-0.01103

C	1.90723	2.15520	-0.19126
N	2.97442	2.93614	-0.32215
C	4.13042	2.30476	-0.45541
N	4.31396	0.99220	-0.44137
C	3.21175	0.26501	-0.30943
N	1.97152	0.80195	-0.22003
N	3.30826	-1.08676	-0.25185
P	1.82096	-1.96837	-0.09051
H	-0.43209	-0.72927	-1.34362
Si	-1.59029	-1.80703	-1.21756
H	-1.64016	-2.18515	-2.66263
H	-1.14087	-2.98557	-0.44288
C	-4.90168	-0.12694	0.71737
C	-5.64584	0.33893	-0.36569
C	-3.69525	-0.79095	0.50915
H	-6.59148	0.85921	-0.19578
H	-3.12333	-1.15112	1.36821
C	-5.19316	0.12316	-1.66569
C	-3.21402	-0.99601	-0.79439
H	-5.78258	0.46748	-2.51830
C	-3.98971	-0.54435	-1.87583
H	-3.65010	-0.71594	-2.90272
H	-5.26756	0.01690	1.73667
C	-0.72402	-0.91532	2.59049
O	-1.63751	-0.95776	3.38168
O	-0.78997	-1.25793	1.34351
H	5.02293	2.92749	-0.58315
C	4.62352	-1.71210	-0.33608
H	5.29369	-1.29382	0.42520
H	4.52522	-2.79038	-0.16565
H	5.07431	-1.54335	-1.32354
C	0.57980	4.16978	0.04839
H	0.86965	4.62106	-0.90991
H	-0.45492	4.44832	0.27755
H	1.23598	4.57102	0.83116
C	-1.85968	2.18266	-1.06902
H	-1.48301	1.96624	-2.07789
H	-2.79914	1.63153	-0.91329
H	-2.05968	3.25920	-0.96925
C	1.82455	-3.12507	-1.48956
H	2.73369	-3.74341	-1.49015
H	0.95372	-3.79054	-1.39819
H	1.75402	-2.57067	-2.43526
C	-1.42199	2.05545	1.76653
H	-1.86237	3.06213	1.73998
H	-2.21661	1.32074	1.97152
H	-0.68015	1.99072	2.57374
C	1.97974	-3.00231	1.38390
H	1.01222	-3.50471	1.53551
H	2.76710	-3.76050	1.26881
H	2.18306	-2.37436	2.26137

TS2_Ni_L7

Ni	0.52049	-0.08565	-0.37660
H	0.28631	0.49458	2.61225
P	-1.00768	1.46044	-0.42905
N	-0.05562	2.87109	-0.17935
C	1.27415	2.67199	0.01719
N	2.07930	3.69577	0.25911
C	3.35370	3.38964	0.44757
N	3.88224	2.17512	0.40403
C	3.03278	1.19157	0.15300
N	1.70852	1.39183	-0.04574
N	3.47829	-0.09530	0.08152
P	2.30552	-1.28886	-0.29519
O	2.56144	-2.43464	0.81675
C	2.84564	-3.70485	0.21746
H	1.90802	-4.27506	0.13231
H	3.53448	-4.24408	0.87881
C	3.45505	-3.38917	-1.14304
H	4.54555	-3.25069	-1.08866
H	3.22744	-4.14794	-1.90061
O	2.85069	-2.15184	-1.55625
O	-2.16849	1.61172	0.67900
C	-3.47650	1.74875	0.10485
H	-4.01382	2.51105	0.68302
H	-4.00023	0.78832	0.19829
C	-3.26772	2.16712	-1.35181
H	-3.98202	1.68372	-2.02919
H	-3.32210	3.25712	-1.48984
O	-1.94770	1.72796	-1.71711
C	-0.65908	4.19781	-0.13933
H	0.08653	4.91498	0.21790
H	-1.51212	4.19262	0.55213
H	-0.99131	4.49550	-1.14333
C	4.88689	-0.43384	0.25697
H	5.35883	-0.63647	-0.71467
H	4.96870	-1.31505	0.90581
H	5.39759	0.40939	0.73230
H	-0.28597	-0.98138	-1.43173
Si	-1.17303	-2.19190	-0.84810
H	-1.32516	-2.72098	-2.23689
H	-0.31363	-3.10982	-0.07230
C	-4.58014	-1.50081	1.44072
C	-5.54904	-1.33572	0.45131
C	-3.24477	-1.69045	1.09541
H	-6.59591	-1.19622	0.73112
H	-2.49497	-1.80351	1.88369
C	-5.18701	-1.35962	-0.89505
C	-2.86821	-1.72605	-0.25725
H	-5.94572	-1.24261	-1.67234
C	-3.85520	-1.56000	-1.24604
H	-3.58440	-1.61272	-2.30593
H	-4.86404	-1.48622	2.49498
C	-0.35952	-0.42124	2.47447
O	-1.06166	-0.80493	3.38083
O	-0.25844	-0.96997	1.30832
H	4.03706	4.21978	0.65674

TS2_Ni_L8

Ni	0.67440	-0.33569	-0.49378
H	0.78155	0.22908	2.60788
C	2.31670	1.89734	-0.31487
N	3.35810	2.59340	0.08847
C	4.33085	1.88294	0.66416
N	4.32185	0.56417	0.86948
C	3.24345	-0.06332	0.45091
N	2.24422	0.57623	-0.15909
H	-0.58270	-1.17627	-1.29982
Si	-1.93833	-1.86877	-0.91914
H	-2.28491	-2.32712	-2.29563
H	-1.61105	-2.99917	-0.02959
C	-4.34247	0.49318	1.49395
C	-5.09912	1.20299	0.56310
C	-3.40360	-0.43986	1.06463
H	-5.83119	1.93947	0.90310
H	-2.78787	-0.95434	1.80622
C	-4.93709	0.96610	-0.80209
C	-3.21023	-0.67022	-0.30673
H	-5.54927	1.50397	-1.52934
C	-4.00596	0.02663	-1.23266
H	-3.90314	-0.17187	-2.30570
H	-4.47319	0.66878	2.56382
C	-0.22804	-0.25771	2.41763
O	-1.09115	-0.13454	3.26118
O	-0.31053	-0.87465	1.29558

H	5.21503	2.43288	1.00200
C	2.94877	-1.51158	0.63759
H	3.84975	-2.13965	0.53424
H	2.57040	-1.64226	1.66588
C	1.12794	2.43390	-1.03167
H	0.88245	3.46188	-0.72094
H	1.39206	2.47872	-2.10227
N	1.88049	-1.92086	-0.31152
N	-0.03449	1.51594	-0.84759
C	2.46095	-2.16593	-1.64585
H	3.15789	-3.01977	-1.60268
H	1.65777	-2.39909	-2.35589
H	3.00440	-1.28266	-2.00428
C	1.25947	-3.16238	0.18400
H	0.68894	-2.94388	1.09308
H	0.58859	-3.56405	-0.58324
H	2.04455	-3.90962	0.38559
C	-0.82977	1.98426	0.31128
H	-1.25653	2.97384	0.07943
H	-1.63850	1.27659	0.52252
H	-0.18848	2.06587	1.19788
C	-0.86644	1.56396	-2.06463
H	-0.32809	1.10904	-2.90616
H	-1.80564	1.03019	-1.88649
H	-1.10949	2.61120	-2.31065

TS2_Ni_L9

Ni	0.38968	-0.30683	0.14007
H	-0.16637	-1.10822	2.89643
P	-0.54670	1.60716	0.69021
N	0.69998	2.74526	0.41798
C	1.87577	2.19355	-0.10218
C	2.97106	3.00125	-0.44374
C	4.13277	2.40431	-0.91930
C	4.23539	1.02355	-1.04368
C	3.12997	0.22840	-0.71146
C	1.92114	0.79562	-0.26543
N	3.18800	-1.17619	-0.77265
P	1.89537	-1.86963	0.10908
H	-0.26074	-0.40441	-1.34201
Si	-1.42657	-1.44476	-1.61995
H	-1.39509	-1.50216	-3.11831
H	-1.14131	-2.80621	-1.11838
C	-5.01358	-0.35521	0.27829
C	-5.64317	0.39610	-0.71291
C	-3.76676	-0.93064	0.04278
H	-6.61938	0.84943	-0.52130
H	-3.28021	-1.50584	0.83479
C	-5.02916	0.55732	-1.95293
C	-3.12565	-0.76399	-1.19560
H	-5.52128	1.13402	-2.74030
C	-3.78607	-0.02392	-2.18900
H	-3.31684	0.10529	-3.17039
H	-5.49600	-0.49775	1.24844
C	-1.07566	-1.51342	2.36381
O	-2.02696	-1.89923	3.01561
O	-0.98308	-1.51366	1.08189
H	4.98672	3.03197	-1.18619
C	4.44507	-1.84356	-0.99540
H	5.20420	-1.57398	-0.23699
H	4.30384	-2.93150	-0.96729
H	4.85129	-1.59406	-1.98789
C	0.57465	4.16132	0.64038
H	0.63671	4.73532	-0.30103
H	-0.39370	4.38679	1.10469
H	1.36062	4.53218	1.31986
C	-1.97983	2.27717	-0.21913
H	-1.76831	2.26149	-1.29716
H	-2.86217	1.65149	-0.02331
H	-2.19785	3.30806	0.09730
C	1.58788	-3.49299	-0.65277
H	2.45316	-4.16423	-0.55200
H	0.72896	-3.94723	-0.13692
H	1.33648	-3.36706	-1.71454
C	-1.08337	1.80751	2.42255
H	-1.43689	2.82962	2.62320
H	-1.91319	1.10773	2.61188
H	-0.25583	1.56045	3.10053
C	2.57318	-2.32779	1.74401
H	1.78253	-2.80140	2.34450
H	3.41541	-3.02982	1.64664
H	2.90788	-1.41484	2.25654
H	5.16753	0.57576	-1.39104
H	2.92309	4.08616	-0.33879

TS2_Ni_L10

Ni	0.71054	-0.37129	-0.43157
H	-0.70331	-0.32498	3.22706
C	2.46739	1.76313	-0.29493
C	3.72277	2.34632	-0.12954
C	4.82443	1.53662	0.15595
C	4.68888	0.15336	0.28597
C	3.43253	-0.43019	0.11965
C	2.35001	0.38463	-0.18105
H	-0.72864	-1.14866	-1.34131
Si	-2.02735	-1.83400	-0.93985
H	-2.51746	-2.36043	-2.25023
H	-1.74622	-2.95358	-0.01304
C	-4.34565	0.60425	1.51884
C	-5.12808	1.30305	0.60223
C	-3.43672	-0.35106	1.07348
H	-5.83690	2.05786	0.95211
H	-2.81611	-0.88910	1.79450
C	-5.01351	1.03330	-0.76030
C	-3.29120	-0.61969	-0.29397
H	-5.63616	1.56989	-1.48052
C	-4.10617	0.07426	-1.20081
H	-4.02611	-0.13347	-2.27386
H	-4.43788	0.81002	2.58766
C	-0.39825	-0.04545	2.18199
O	-0.22869	1.14613	1.92779
O	-0.27706	-1.03000	1.38672
H	5.80886	1.99374	0.28138
C	3.08878	-1.86899	0.28913
H	3.84340	-2.56220	-0.12572
H	2.95302	-2.11554	1.36121
C	1.17718	2.46256	-0.53143
H	0.76672	2.84236	0.42223
H	1.24069	3.29301	-1.25726
H	5.56161	-0.46446	0.51748
H	3.84698	3.42945	-0.21840
O	1.83940	-2.08134	-0.37831
O	0.26301	1.47665	-1.04178
C	1.24277	-3.31563	-0.03067
H	1.99381	-4.11895	-0.09562
H	0.81935	-3.26102	0.98486
H	0.44296	-3.52076	-0.75075
C	-1.08254	1.92431	-1.00615
H	-1.68283	1.23742	-1.61198
H	-1.44793	1.93911	0.03360
H	-1.13893	2.93210	-1.44728

4.5.3. TS3

TS3_Co_L1

Co	-0.43602	0.06272	-0.00076
H	0.10734	-0.90554	-3.43828
P	1.09191	1.54256	0.16314
N	0.19406	2.97791	-0.40341
C	-1.12587	2.79530	-0.62689
N	-1.90316	3.83690	-0.94152
C	-3.18973	3.55716	-1.07380
N	-3.77367	2.38977	-0.85368
C	-2.94058	1.39337	-0.53827
N	-1.59477	1.53097	-0.49614
N	-3.43208	0.17486	-0.21922
P	-2.23621	-1.00843	0.37432
N	-2.79912	-2.44349	-0.35149
C	-3.17780	-3.47420	0.59234
H	-2.33261	-4.16257	0.79389
H	-4.00559	-4.07993	0.18424
C	-3.59199	-2.73728	1.85662
H	-4.67328	-2.48578	1.83987
H	-3.41508	-3.34581	2.75892
N	-2.76965	-1.54662	1.89118
N	2.53853	1.69012	-0.71911
C	3.72022	1.81107	0.11337
H	4.47439	2.43855	-0.39231
H	4.17718	0.81972	0.29614
C	3.24851	2.43879	1.41552
H	3.87048	2.11440	2.26742
H	3.30091	3.54691	1.37613
N	1.88830	1.97694	1.59243
C	0.78168	4.29572	-0.49305
H	0.37611	4.83851	-1.35602
H	1.86593	4.18280	-0.61952
H	0.57881	4.89390	0.41001
C	-4.85816	-0.06241	-0.21937
H	-5.34497	0.37368	0.66773
H	-5.02896	-1.14624	-0.23411
H	-5.32041	0.38286	-1.10955
H	0.50280	-0.87283	0.80122
Si	0.89057	-2.33225	-0.00796
H	0.33824	-2.89596	1.29305
H	0.62386	-3.53660	-0.87197
C	5.04538	-2.14744	-0.46171
C	5.47243	-1.69634	0.78524
C	3.68599	-2.33577	-0.71028
H	6.53840	-1.55348	0.98165
H	3.35169	-2.68840	-1.69184
C	4.53603	-1.42870	1.78367
C	2.73218	-2.07626	0.28297
H	4.86604	-1.07531	2.76475
C	3.18012	-1.61490	1.52878
H	2.44814	-1.39519	2.31478
H	5.77630	-2.35878	-1.24655
C	0.73746	-1.55669	-2.78192
O	1.47642	-2.39297	-3.23940
O	0.56958	-1.26967	-1.51908
C	2.70174	1.09681	-2.02226
H	3.08938	0.06236	-1.96803
H	1.73765	1.08037	-2.54980
H	3.40254	1.69948	-2.62203
C	1.17613	2.32936	2.78623
H	0.18962	1.84188	2.78364
H	1.72231	1.98521	3.67976
H	1.02201	3.42267	2.88127
C	-2.42608	-2.83340	-1.68642
H	-1.55286	-3.51261	-1.70408
H	-3.26849	-3.34188	-2.18305
H	-2.17857	-1.94125	-2.27927
C	-2.87981	-0.65480	3.00996
H	-3.89138	-0.21085	3.09995
H	-2.65605	-1.18272	3.95084
H	-2.15335	0.16416	2.89971
H	-3.84354	4.38386	-1.37835

TS3_Co_L2

Co	0.43680	-0.02546	-0.03963
H	0.02759	-1.11950	3.16468
P	-0.92155	1.60378	-0.25042
O	-0.12924	2.78326	0.87692
C	1.05334	2.45031	1.30291
N	1.75815	3.31297	2.02389
C	2.98470	2.91587	2.33125
N	3.58735	1.80759	1.92323
C	2.82888	0.99299	1.20152
N	1.51890	1.22700	0.95800
O	3.35045	-0.07154	0.66705
P	2.29505	-0.93759	-0.53048
N	2.83995	-2.50259	-0.30853
C	3.87473	-2.93404	-1.22978
H	3.45873	-3.64923	-1.96450
H	4.68379	-3.45060	-0.68573
C	4.37738	-1.66918	-1.90580
H	5.22082	-1.22551	-1.33791
H	4.72649	-1.85665	-2.93337
N	3.24374	-0.75714	-1.92093
N	-2.50443	1.74370	0.25443
C	-3.33163	2.62372	-0.54816
H	-3.95651	3.26014	0.10091
H	-4.01229	2.02639	-1.18543
C	-2.36438	3.45062	-1.37749
H	-2.79459	3.75160	-2.34557
H	-2.06799	4.37291	-0.83698
N	-1.20513	2.59604	-1.58973
H	-0.44397	-0.84233	-1.01915
Si	-1.03159	-2.30928	-0.31896
H	-0.62491	-2.77502	-1.70622
H	-0.74716	-3.57076	0.43826
C	-5.11283	-2.09177	0.47382
C	-5.60767	-1.35401	-0.59955
C	-3.74900	-2.36391	0.56395
H	-6.67694	-1.13749	-0.66943
H	-3.35919	-2.92300	1.42100
C	-4.73658	-0.89382	-1.58666
C	-2.86139	-1.91414	-0.42269
H	-5.12176	-0.31881	-2.43339
C	-3.37616	-1.17707	-1.49741
H	-2.69879	-0.81636	-2.28048
H	-5.79301	-2.45431	1.24869
C	-0.65723	-1.72737	2.52274
O	-1.35580	-2.59857	2.97452
O	-0.59990	-1.35710	1.26901
C	-3.10337	1.03517	1.35525
H	-3.97357	0.44723	1.02062
H	-2.38021	0.33986	1.79776
H	-3.43298	1.73659	2.14052
C	-0.09277	3.18075	-2.29379
H	0.73753	2.46161	-2.33939
H	-0.38245	3.43229	-3.32534
H	0.26977	4.10274	-1.79947
C	2.34467	-3.43464	0.66827
H	1.89782	-4.32263	0.18902
H	3.15497	-3.77213	1.33615

H	1.57250	-2.96475	1.29092
C	3.48836	0.56239	-2.44470
H	4.30044	1.08167	-1.89919
H	3.76894	0.50455	-3.50729
H	2.57383	1.16758	-2.36661
H	3.57127	3.58575	2.97130

TS3_Co_L3

Co	-0.50811	0.02074	-0.23976
H	0.22909	-0.97431	-3.63046
P	0.77168	1.75527	-0.02195
C	-0.13084	2.91241	-1.22335
C	-1.56747	2.58830	-1.15158
N	-2.47511	3.54937	-1.28744
C	-3.73939	3.17753	-1.15536
N	-4.15674	1.93958	-0.92423
C	-3.20858	1.02016	-0.79730
N	-1.88312	1.29820	-0.86969
C	-3.57732	-0.38641	-0.52684
P	-2.14882	-1.23127	0.35798
N	-2.49478	-2.88457	0.04728
C	-2.86043	-3.62557	1.23549
H	-1.97647	-4.12755	1.68006
H	-3.60168	-4.40711	0.99299
C	-3.42603	-2.59363	2.19069
H	-4.49325	-2.38600	1.95366
H	-3.37980	-2.92729	3.24020
N	-2.60294	-1.41538	2.00290
N	2.40537	2.00572	-0.40952
C	3.13140	2.81021	0.54973
H	3.81104	3.51114	0.03320
H	3.75378	2.16757	1.20417
C	2.07291	3.54872	1.34817
H	2.42545	3.82299	2.35572
H	1.78021	4.49051	0.83359
N	0.95005	2.63321	1.44457
H	0.54620	-0.75312	0.58688
Si	1.14408	-2.17476	-0.14486
H	0.34280	-2.82653	0.96039
H	1.36858	-3.39365	-0.99947
C	5.27747	-1.83850	0.13614
C	5.47380	-0.99577	1.22778
C	3.98378	-2.17440	-0.25951
H	6.48726	-0.73267	1.54245
H	3.83187	-2.83318	-1.12084
C	4.37404	-0.48876	1.91939
C	2.86739	-1.67109	0.42118
H	4.52365	0.16821	2.78093
C	3.08414	-0.81745	1.51095
H	2.22708	-0.39648	2.04865
H	6.13630	-2.23554	-0.41100
C	0.93314	-1.51697	-2.95015
O	1.76228	-2.28217	-3.37673
O	0.73899	-1.19326	-1.70046
C	3.15451	1.25179	-1.37929
H	3.92707	0.62577	-0.89963
H	2.49037	0.58057	-1.93848
H	3.65210	1.92276	-2.10107
C	-0.23339	3.14474	2.08266
H	-1.00991	2.36582	2.10372
H	-0.01253	3.42911	3.12306
H	-0.64726	4.03819	1.56874
C	-1.89787	-3.61640	-1.03799
H	-1.00896	-4.19511	-0.72249
H	-2.62436	-4.32114	-1.47560
H	-1.58290	-2.92851	-1.83700
C	-2.96291	-0.24583	2.75632
H	-3.99609	0.10075	2.54121
H	-2.89611	-0.44971	3.83636
H	-2.26659	0.57383	2.52495
H	-4.50505	3.95643	-1.24678
H	0.27851	2.58079	-2.19441
H	-3.63054	-0.93004	-1.48692
H	0.04755	3.99224	-1.12325
H	-4.56790	-0.46006	-0.05460

TS3_Co_L4

Co	-0.44922	0.08330	0.01177
H	0.10517	-0.83944	-3.47390
P	1.04472	1.58330	0.18207
N	0.13587	2.98550	-0.39670
C	-1.20721	2.80226	-0.59560
C	-2.06278	3.86508	-0.92127
C	-3.41754	3.60354	-1.05484
C	-3.91653	2.32875	-0.83484
C	-3.01193	1.30774	-0.51105
N	-1.67575	1.53677	-0.44143
N	-3.42492	0.03194	-0.22612
P	-2.18558	-1.07547	0.37548
N	-2.67868	-2.54951	-0.33112
C	-2.94574	-3.60014	0.62759
H	-2.04018	-4.20950	0.82127
H	-3.72502	-4.27959	0.24044
C	-3.39954	-2.88736	1.89223
H	-4.49849	-2.72323	1.88854
H	-3.16453	-3.47365	2.79636
N	-2.67446	-1.63613	1.90511
N	2.50868	1.75190	-0.67436
C	3.67507	1.83988	0.18219
H	4.45150	2.45726	-0.30201
H	4.11081	0.83926	0.36603
C	3.18764	2.46458	1.47991
H	3.79418	2.13107	2.33969
H	3.25244	3.57307	1.44696
N	1.82084	2.01582	1.63005
C	0.72581	4.29392	-0.52160
H	0.53233	4.73131	-1.51449
H	1.81248	4.19937	-0.41531
H	0.35114	4.99401	0.24574
C	-4.82424	-0.30971	-0.25474
H	-5.39856	0.21808	0.52700
H	-4.92448	-1.39004	-0.09919
H	-5.27490	-0.07668	-1.23297
H	0.54500	-0.84466	0.75767
Si	0.94060	-2.28601	-0.07923
H	0.37244	-2.89209	1.19564
H	0.72485	-3.48233	-0.97055
C	5.09544	-2.04544	-0.49560
C	5.51316	-1.64658	0.77216
C	3.73876	-2.23413	-0.75829
H	6.57698	-1.50277	0.97955
H	3.41157	-2.54664	-1.75588
C	4.57011	-1.43302	1.77739
C	2.77800	-2.02601	0.24038
H	4.89277	-1.12149	2.77495
C	3.21682	-1.61861	1.50781
H	2.47971	-1.44273	2.29975
H	5.83152	-2.21538	-1.28571
C	0.76826	-1.47286	-2.83317
O	1.53353	-2.27411	-3.31080
O	0.60563	-1.20561	-1.56557
C	2.68936	1.18566	-1.98687
H	3.06928	0.14781	-1.95022
H	1.73320	1.18560	-2.52922
H	3.40372	1.79664	-2.56218
C	1.07338	2.42496	2.78333

H	0.08595	1.93859	2.77337
H	1.59040	2.12456	3.70921
H	0.91801	3.52239	2.82010
C	-2.33992	-2.91298	-1.68184
H	-1.41910	-3.52286	-1.74443
H	-3.16389	-3.48673	-2.13738
H	-2.19267	-2.00576	-2.28557
C	-2.87833	-0.72533	2.99437
H	-3.92314	-0.35678	3.04755
H	-2.63920	-1.20734	3.95599
H	-2.21350	0.14385	2.87623
H	-4.10173	4.41487	-1.31413
H	-4.98392	2.12624	-0.89878
H	-1.67122	4.87173	-1.05478

TS3_Co_L5

Co	-0.20684	0.36967	0.09536
H	-0.74348	0.62557	-3.42561
P	-0.78304	-1.66570	0.37448
N	0.77739	-2.47354	0.02980
C	1.83177	-1.65375	-0.14610
N	3.06252	-2.16567	-0.28553
C	4.02264	-1.27081	-0.37850
N	3.91537	0.04091	-0.28001
C	2.66475	0.48961	-0.13920
N	1.57967	-0.32262	-0.15114
N	2.43454	1.80483	0.04842
P	0.74438	2.24532	0.43010
N	0.57852	3.70079	-0.43691
C	0.27266	4.84882	0.39191
H	-0.82125	5.01218	0.46310
H	0.71468	5.76140	-0.04386
C	0.86530	4.53421	1.75675
H	1.91878	4.87685	1.82663
H	0.30778	5.03144	2.56747
N	0.76436	3.09625	1.89432
N	-1.84977	-2.60600	-0.55467
C	-2.89274	-3.24596	0.22541
H	-3.17013	-4.20875	-0.23694
H	-3.80247	-2.61667	0.26149
C	-2.31047	-3.43754	1.61728
H	-3.09692	-3.40483	2.39060
H	-1.79773	-4.41698	1.71365
N	-1.38964	-2.33571	1.80292
C	0.95232	-3.90869	0.07861
H	1.68033	-4.23271	-0.67519
H	-0.01558	-4.38065	-0.13248
H	1.31338	-4.23974	1.06547
C	3.53490	2.74295	0.09731
H	4.06903	2.69382	1.05943
H	3.13377	3.75381	-0.04738
H	4.25697	2.52836	-0.70055
H	-1.56361	0.74609	0.73974
Si	-2.55489	1.72388	-0.27324
H	-2.51917	2.58496	0.97984
H	-2.82160	2.82237	-1.26648
C	-5.93574	-0.63369	-0.97156
C	-6.24305	-1.11927	0.29750
C	-4.83867	0.20805	-1.15202
H	-7.10575	-1.77554	0.43967
H	-4.59807	0.58794	-2.15073
C	-5.44866	-0.76386	1.38734
C	-4.03265	0.57895	-0.06725
H	-5.68717	-1.14064	2.38595
C	-4.35365	0.07573	1.20111
H	-3.73154	0.34984	2.06111
H	-6.55737	-0.90826	-1.82765
C	-1.69184	0.91869	-2.90983
O	-2.67977	1.23378	-3.52458
O	-1.57202	0.84970	-1.61010
C	-2.13571	-2.29934	-1.93358
H	-2.98622	-1.60139	-2.04259
H	-1.25221	-1.84842	-2.40725
H	-2.37307	-3.22595	-2.48008
C	-0.73421	-2.16991	3.06795
H	-0.12983	-1.25063	3.05179
H	-1.47423	-2.07481	3.87927
H	-0.06666	-3.01927	3.31353
C	0.21834	3.73531	-1.83085
H	-0.87270	3.82625	-1.98992
H	0.71132	4.58701	-2.32657
H	0.56474	2.81761	-2.32755
C	1.18783	2.48064	3.11999
H	2.26698	2.63385	3.31947
H	0.62343	2.88797	3.97392
H	0.99679	1.39821	3.07304
C	5.44619	-1.77715	-0.58566
F	5.49526	-3.08972	-0.74667
F	5.99044	-1.20798	-1.66027
F	6.20203	-1.46124	0.46749

TS3_Co_L6

Co	0.44002	-0.21022	-0.38627
H	-0.27667	-0.77062	3.22458
P	-0.46422	1.73912	-0.37541
N	0.90479	2.72231	0.14802
C	2.07531	2.05694	0.30008
N	3.17545	2.73229	0.64367
C	4.27746	2.00380	0.72660
N	4.39192	0.70853	0.47585
C	3.25333	0.09740	0.13955
N	2.05649	0.72264	0.07637
N	3.27079	-1.22191	-0.17270
P	1.71846	-1.90694	-0.65074
C	0.87469	4.15487	0.36288
H	1.24446	4.40705	1.36630
H	-0.15649	4.51520	0.26858
H	1.50439	4.68267	-0.36889
C	4.53016	-1.93759	-0.14041
H	5.24335	-1.52143	-0.86711
H	4.35611	-2.99279	-0.38176
H	4.99160	-1.87516	0.85509
H	-0.85941	-0.78369	-1.01892
Si	-1.72999	-1.92209	-0.08407
H	-1.56807	-2.71058	-1.38200
H	-1.87643	-3.11251	0.82736
C	-5.35009	0.05101	0.63282
C	-5.63226	0.63699	-0.59894
C	-4.20901	-0.73526	0.79070
H	-6.52406	1.25778	-0.72051
H	-3.97983	-1.17807	1.76505
C	-4.77599	0.42517	-1.67934
C	-3.33171	-0.95004	-0.28067
H	-4.99851	0.87399	-2.65150
C	-3.64017	-0.36361	-1.51628
H	-2.96912	-0.52653	-2.36858
H	-6.02176	0.21003	1.48041
C	-1.13806	-1.15583	2.62373
O	-2.12459	-1.60645	3.15380
O	-0.91968	-1.02841	1.34376
H	5.19279	2.52916	1.02553
C	2.07795	-2.67664	-2.27077
H	2.86101	-3.44814	-2.22022
H	1.14453	-3.14417	-2.62021
H	2.36145	-1.89682	-2.98965
C	-1.06343	2.61735	-1.86350

H	-0.32023	2.53602	-2.66714
H	-1.98696	2.11117	-2.18430
H	-1.29612	3.67497	-1.66844
C	-1.76219	2.17206	0.83687
H	-2.66227	1.58916	0.58447
H	-1.43823	1.87573	1.84359
H	-2.01730	3.24164	0.82460
C	1.59761	-3.38638	0.42436
H	0.68714	-3.93786	0.14107
H	2.45743	-4.06508	0.32596
H	1.48937	-3.06817	1.47034

TS3_Co_L7

Co	0.44079	-0.14236	-0.23055
H	0.16009	0.56977	2.93383
P	-0.85929	1.45734	-0.77879
N	-0.01177	2.81774	-0.05582
C	1.26658	2.56683	0.32235
N	2.07035	3.55642	0.71173
C	3.31667	3.19420	0.97649
N	3.84460	1.99111	0.81437
C	2.98724	1.04720	0.42430
N	1.66465	1.27669	0.25810
N	3.40823	-0.20959	0.15056
P	2.17233	-1.27094	-0.51968
O	2.52925	-2.66864	0.31215
C	2.94964	-3.72468	-0.53072
H	2.07890	-4.34330	-0.80732
H	3.66763	-4.35217	0.01618
C	3.57043	-3.05800	-1.75161
H	4.64552	-2.85825	-1.60282
H	3.45037	-3.65696	-2.66443
O	2.87252	-1.83508	-1.91478
O	-2.31573	1.61215	-0.00609
C	-3.38666	1.74097	-0.92548
H	-4.20189	2.29058	-0.43582
H	-3.75537	0.73912	-1.20804
C	-2.80314	2.48638	-2.11759
H	-3.30639	2.23614	-3.06098
H	-2.84326	3.57951	-1.97508
O	-1.44649	2.06667	-2.20220
C	-0.55966	4.15882	-0.03005
H	-0.00596	4.76600	0.69416
H	-1.61381	4.10917	0.27340
H	-0.48540	4.63763	-1.01848
C	4.80198	-0.58468	0.26485
H	5.30391	-0.54965	-0.71447
H	4.87061	-1.60155	0.67425
H	5.31182	0.10956	0.94167
H	-0.54677	-1.16309	-0.81398
Si	-1.05198	-2.17674	0.55648
H	-0.73906	-3.22262	-0.50043
H	-0.65007	-2.92990	1.78391
C	-5.04002	-1.06651	1.14075
C	-5.61926	-1.34779	-0.09564
C	-3.68362	-1.30595	1.34911
H	-6.68421	-1.15986	-0.25722
H	-3.23332	-1.07859	2.32022
C	-4.83930	-1.87325	-1.12436
C	-2.88583	-1.84094	0.32855
H	-5.28913	-2.09875	-2.09492
C	-3.48480	-2.11825	-0.90766
H	-2.87677	-2.53703	-1.71765
H	-5.64996	-0.65854	1.95072
C	-0.56135	-0.26221	2.74475
O	-1.21341	-0.76684	3.62079
O	-0.60981	-0.58114	1.47572
H	3.98678	3.97827	1.34888

TS3_Co_L8

Co	0.73842	-0.13975	-0.31952
H	-0.05501	-1.62950	3.01417
C	2.56290	1.88903	0.11982
N	3.73749	2.43640	0.34305
C	4.77076	1.58903	0.39259
N	4.68917	0.26418	0.23828
C	3.47827	-0.21272	0.04557
N	2.38078	0.56319	-0.02439
H	-0.67186	-0.63574	-1.01217
Si	-1.80146	-1.69555	-0.32911
H	-1.68862	-2.35343	-1.69825
H	-2.27606	-2.92243	0.41489
C	-4.84309	1.01412	0.62033
C	-5.20269	1.52184	-0.62590
C	-3.85960	0.03012	0.71896
H	-5.97529	2.29171	-0.70260
H	-3.58718	-0.37417	1.69929
C	-4.57983	1.03941	-1.77686
C	-3.20977	-0.45227	-0.42554
H	-4.86396	1.42924	-2.75797
C	-3.59300	0.06327	-1.67166
H	-3.09446	-0.29830	-2.57833
H	-5.33654	1.38208	1.52363
C	-1.00753	-1.54228	2.43166
O	-2.08369	-1.63675	2.96658
O	-0.77277	-1.30136	1.16767
H	5.76233	2.01480	0.57095
C	3.13697	-1.66218	-0.06535
H	3.94463	-2.24097	-0.55048
H	3.02524	-2.05744	0.95967
C	1.29588	2.64156	-0.10657
H	1.25178	3.57305	0.48629
H	1.28578	2.93309	-1.17146
N	1.84076	-1.82218	-0.76113
N	0.12725	1.76716	0.13730
C	2.04733	-1.77218	-2.21364
H	1.07416	-1.81234	-2.71922
H	2.54494	-0.83517	-2.49441
H	2.66991	-2.62570	-2.54591
C	1.26080	-3.11793	-0.40340
H	1.00989	-3.13245	0.66466
H	0.34693	-3.28610	-0.98836
H	1.96881	-3.93757	-0.62648
C	-0.20974	1.78931	1.56636
H	-0.45929	2.81811	1.88669
H	-1.07466	1.14128	1.74785
H	0.63817	1.42359	2.16175
C	-1.01536	2.26769	-0.63355
H	-0.81968	2.14134	-1.70650
H	-1.91195	1.69594	-0.37333
H	-1.19512	3.33672	-0.41248

TS3_Co_L9

Co	0.47430	-0.23047	-0.27836
H	-0.68614	-0.65658	3.53309
P	-0.28330	1.75868	-0.24785
N	1.05822	2.75107	0.21758
C	2.26747	2.03620	0.25075
C	3.49474	2.68185	0.47916
C	4.66542	1.92587	0.48500
C	4.63150	0.55171	0.25631
C	3.39192	-0.06911	0.02763
C	2.17322	0.64455	0.02670
N	3.29876	-1.44756	-0.22501
P	1.67305	-1.96276	-0.52309

C	1.03672	4.17526	0.34252
H	1.46781	4.50751	1.30504
H	0.00241	4.54413	0.30687
H	1.60297	4.68201	-0.46504
C	4.46652	-2.26828	-0.30135
H	5.15957	-1.93727	-1.09974
H	4.18735	-3.30861	-0.51770
H	5.03576	-2.27088	0.64802
H	-0.96380	-0.80219	-0.71713
Si	-1.86303	-1.85230	0.15465
H	-1.56310	-2.85671	-0.96296
H	-2.40251	-2.90097	1.12142
C	-5.60754	0.05871	0.17392
C	-5.65398	0.67794	-1.07323
C	-4.50270	-0.71408	0.53330
H	-6.51820	1.28786	-1.35384
H	-4.45540	-1.17778	1.52344
C	-4.58894	0.52146	-1.96067
C	-3.41536	-0.86895	-0.33605
H	-4.61775	1.00344	-2.94294
C	-3.48402	-0.23887	-1.58678
H	-2.63471	-0.33601	-2.27450
H	-6.43655	0.18395	0.87696
C	-1.50818	-0.88811	2.81073
O	-2.66519	-0.92015	3.17427
O	-1.04426	-1.07419	1.61522
H	5.62558	2.41963	0.66563
H	5.56301	-0.02023	0.25553
H	3.54643	3.76036	0.64907
C	1.77717	-2.80991	-2.15779
H	2.50184	-3.64059	-2.17277
H	0.77746	-3.20505	-2.39583
H	2.04748	-2.06514	-2.91895
C	-0.90051	2.57331	-1.78445
H	-0.15831	2.43082	-2.58189
H	-1.84028	2.08331	-2.08099
H	-1.09529	3.64901	-1.64349
C	-1.61854	2.26200	0.91500
H	-1.85534	3.33647	0.86463
H	-2.52600	1.68824	0.66441
H	-1.31961	1.99933	1.93903
C	1.50889	-3.44131	0.57062
H	0.53612	-3.91804	0.36948
H	2.30237	-4.18863	0.41109
H	1.52093	-3.10643	1.61712

TS3_Fe_L1

Fe	-0.34396	-0.15599	0.08884
H	-0.07612	-1.01718	-2.95688
P	0.93514	1.46635	0.48136
N	0.32077	2.68378	-0.73788
C	-0.85575	2.36580	-1.30908
N	-1.49069	3.24248	-2.10344
C	-2.69120	2.85790	-2.50877
N	-3.34888	1.77614	-2.12310
C	-2.65639	0.95054	-1.32128
N	-1.34874	1.13830	-1.00522
N	-3.24830	-0.11239	-0.75140
P	-2.23986	-0.92717	0.54745
N	-2.92778	-2.52511	0.44581
C	-3.76496	-2.92793	1.54323
H	-3.18907	-3.51790	2.28998
H	-4.59376	-3.57665	1.19580
C	-4.28883	-1.64333	2.16369
H	-5.24467	-1.33043	1.68416
H	-4.50772	-1.77326	3.24029
N	-3.24472	-0.67628	1.96483
N	2.62199	1.60651	0.15523
C	3.42889	2.35547	1.07587
H	4.10736	3.04531	0.53265
H	4.08001	1.68681	1.67763
C	2.45892	3.13256	1.96302
H	2.82774	3.20319	3.00328
H	2.33933	4.17997	1.60379
N	1.21227	2.41456	1.91627
C	0.94687	3.94923	-1.01380
H	0.82285	4.21642	-2.07254
H	2.02013	3.86930	-0.79034
H	0.52004	4.77150	-0.41252
C	-4.63725	-0.38688	-1.00784
H	-5.30896	0.28955	-0.44955
H	-4.84635	-1.42471	-0.71515
H	-4.86190	-0.26314	-2.07650
H	0.52269	-1.05531	1.08923
Si	1.17146	-2.41107	0.30730
H	1.10752	-3.01511	1.72202
H	0.68973	-3.62841	-0.42797
C	5.17867	-2.25096	-0.93695
C	5.82303	-1.67570	0.15683
C	3.79913	-2.45435	-0.90838
H	6.90338	-1.50404	0.13198
H	3.29080	-2.87706	-1.78233
C	5.08192	-1.31336	1.28174
C	3.04081	-2.11145	0.21894
H	5.57945	-0.85206	2.14051
C	3.70809	-1.54170	1.31065
H	3.13011	-1.26219	2.19991
H	5.75342	-2.53271	-1.82429
C	0.62929	-1.72456	-2.45401
O	1.15964	-2.63417	-3.05309
O	0.79144	-1.42819	-1.19875
C	3.22383	1.09055	-1.03476
H	4.10331	0.46069	-0.81210
H	2.49867	0.46665	-1.57565
H	3.54997	1.90507	-1.71620
C	0.10559	2.98323	2.61782
H	-0.78398	2.35394	2.45794
H	0.29941	3.03213	3.70450
H	-0.13391	4.01343	2.27415
C	-2.29917	-3.57232	-0.30179
H	-1.70738	-4.25503	0.34115
H	-3.04967	-4.18198	-0.83872
H	-1.61507	-3.14723	-1.04965
C	-3.50467	0.66261	2.39018
H	-4.40099	1.10381	1.90111
H	-3.65939	0.71628	3.48273
H	-2.63701	1.29282	2.13500
H	-3.20729	3.52134	-3.21647

TS3_Fe_L2

Fe	0.37584	-0.14840	0.03498
H	-0.14864	-0.36436	3.31921
P	-0.78932	1.53824	-0.34070
O	0.02975	2.76258	0.82907
C	1.18958	2.38043	1.25080
N	1.96207	3.23501	1.92895
C	3.17697	2.78610	2.20567
N	3.71978	1.64512	1.80913
C	2.89616	0.83777	1.13193
N	1.58134	1.11715	0.94881
O	3.35530	-0.24105	0.59328
P	2.13322	-1.08589	-0.57613
N	2.66862	-2.68652	-0.32669
C	3.73573	-3.16051	-1.16885
H	3.35815	-3.88968	-1.91610
H	4.50971	-3.67982	-0.57205

C	4.29953	-1.92150	-1.84512
H	5.11634	-1.48032	-1.23300
H	4.71780	-2.14684	-2.84225
N	3.19085	-1.00049	-1.95253
N	-2.40773	1.91425	0.02833
C	-3.03606	2.94026	-0.76271
H	-3.60637	3.63844	-0.12144
H	-3.75826	2.49469	-1.47981
C	-1.90431	3.64809	-1.48597
H	-2.22828	4.07021	-2.45382
H	-1.51198	4.48740	-0.87076
N	-0.88733	2.64042	-1.68155
H	-0.65470	-1.11461	-0.74956
Si	-1.20843	-2.35047	0.23560
H	-0.54850	-3.27350	-0.76729
H	-1.49040	-3.40487	1.29791
C	-5.38408	-2.13042	0.10063
C	-5.64350	-1.31931	-1.00206
C	-4.06745	-2.41675	0.45827
H	-6.67487	-1.08802	-1.28493
H	-3.86546	-3.04458	1.33227
C	-4.58121	-0.79516	-1.73850
C	-2.98597	-1.89926	-0.26575
H	-4.77682	-0.15309	-2.60236
C	-3.26945	-1.07749	-1.36585
H	-2.44121	-0.63661	-1.93202
H	-6.21184	-2.53513	0.69035
C	-0.88701	-1.00222	2.77546
O	-1.93610	-1.33968	3.26869
O	-0.45765	-1.29769	1.57773
C	-3.19057	1.28231	1.04809
H	-4.11414	0.84043	0.63122
H	-2.62511	0.47439	1.52707
H	-3.48011	2.00244	1.83625
C	0.34013	3.10796	-2.25755
H	1.07343	2.28743	-2.26698
H	0.18329	3.44262	-3.29724
H	0.77543	3.95389	-1.68559
C	2.13932	-3.56402	0.67140
H	1.65623	-4.45672	0.23112
H	2.93289	-3.91066	1.36032
H	1.38848	-3.03585	1.27539
C	3.51822	0.29811	-2.46621
H	4.33148	0.78501	-1.88838
H	3.83768	0.23391	-3.52042
H	2.62885	0.94389	-2.41634
H	3.81458	3.44562	2.81060

TS3_Fe_L3

Fe	0.46028	-0.06545	0.32009
H	-0.23903	-0.31138	3.54186
P	-0.75262	1.64191	-0.10282
C	0.05468	2.91529	1.05733
C	1.48841	2.58678	1.12426
N	2.38255	3.55554	1.27084
C	3.65986	3.19670	1.21298
N	4.08830	1.95179	1.02810
C	3.15556	1.01986	0.89457
N	1.80768	1.26946	0.91100
C	3.54671	-0.37610	0.62285
P	2.14105	-1.20788	-0.33174
N	2.57413	-2.88510	-0.02255
C	3.04194	-3.60596	-1.17309
H	2.21292	-4.14961	-1.68072
H	3.80215	-4.36131	-0.89135
C	3.62209	-2.54983	-2.09205
H	4.66069	-2.29205	-1.77408
H	3.68035	-2.89578	-3.14033

N	2.73342	-1.42334	-1.97294
N	-2.43350	1.99768	0.14792
C	-3.07065	2.79002	-0.86620
H	-3.74562	3.54657	-0.41813
H	-3.69698	2.15709	-1.53338
C	-1.94353	3.44730	-1.64055
H	-2.24410	3.70939	-2.67152
H	-1.63637	4.39765	-1.14279
N	-0.86915	2.48624	-1.64846
H	-0.57352	-0.96717	-0.53112
Si	-1.17795	-2.22662	0.36899
H	-0.46133	-3.13067	-0.61370
H	-1.54962	-3.30308	1.38284
C	-5.33532	-1.98737	-0.07672
C	-5.50274	-1.18875	-1.20620
C	-4.05251	-2.27101	0.38995
H	-6.50721	-0.96081	-1.57548
H	-3.92212	-2.89128	1.28296
C	-4.38309	-0.67320	-1.85882
C	-2.91571	-1.76168	-0.24919
H	-4.50621	-0.04158	-2.74376
C	-3.10634	-0.94946	-1.37576
H	-2.23402	-0.51490	-1.87703
H	-6.20932	-2.38523	0.44749
C	-0.97492	-0.90191	2.94086
O	-2.08618	-1.14918	3.34616
O	-0.46336	-1.24947	1.79308
C	-3.27453	1.35627	1.11178
H	-4.07310	0.75868	0.63129
H	-2.69564	0.67235	1.74537
H	-3.76121	2.09497	1.77846
C	0.36090	2.95202	-2.21904
H	1.13620	2.18057	-2.08995
H	0.24692	3.14537	-3.29973
H	0.72757	3.89021	-1.74563
C	1.96660	-3.65065	1.02337
H	1.15069	-4.30681	0.65623
H	2.71139	-4.29425	1.52919
H	1.53368	-2.98240	1.78277
C	3.11373	-0.24405	-2.69057
H	4.13057	0.11741	-2.41703
H	3.10506	-0.42379	-3.77966
H	2.39487	0.56106	-2.47278
H	4.41551	3.98250	1.32966
H	-0.44858	2.66565	2.00901
H	3.53448	-0.95717	1.56170
H	-0.09953	3.98588	0.85462
H	4.56359	-0.43448	0.20509

TS3_Fe_L4

Fe	-0.36060	-0.15348	-0.03626
H	0.34098	-0.34541	-3.37400
P	0.93020	1.41540	0.47423
N	0.30859	2.70106	-0.66059
C	-0.96988	2.50808	-1.09107
C	-1.72234	3.51325	-1.72433
C	-3.05354	3.25347	-2.02434
C	-3.63323	2.04819	-1.65328
C	-2.82648	1.07821	-1.03018
N	-1.49469	1.27832	-0.83148
N	-3.32226	-0.10018	-0.55954
P	-2.17733	-1.06217	0.45885
N	-2.79330	-2.66576	0.09316
C	-3.34205	-3.37715	1.21544
H	-2.56056	-3.97058	1.74121
H	-4.12389	-4.09185	0.89079
C	-3.90953	-2.31082	2.13684
H	-4.96004	-2.06199	1.85636

H	-3.94142	-2.65723	3.18741
N	-3.02811	-1.18786	1.99061
N	2.62366	1.63736	0.22982
C	3.36429	2.31983	1.25252
H	4.07842	3.04173	0.80507
H	3.97197	1.60838	1.85170
C	2.33430	3.03513	2.11917
H	2.65948	3.09020	3.17506
H	2.19285	4.08839	1.78251
N	1.12129	2.27194	1.99553
C	0.97014	3.94986	-0.90112
H	0.97350	4.20191	-1.97618
H	2.01646	3.86334	-0.58148
H	0.50803	4.79791	-0.35751
C	-4.71810	-0.40175	-0.68843
H	-5.35787	0.26925	-0.08202
H	-4.88254	-1.43689	-0.36557
H	-5.05253	-0.33003	-1.73803
H	0.56447	-1.22512	0.72240
Si	1.09807	-2.43771	-0.30111
H	0.61115	-3.35826	0.82236
H	1.08700	-3.50434	-1.38358
C	5.28256	-2.33432	-0.53134
C	5.65500	-1.70985	0.65726
C	3.93294	-2.53147	-0.82253
H	6.71269	-1.55007	0.88811
H	3.64221	-2.99804	-1.76905
C	4.67086	-1.28102	1.54817
C	2.92963	-2.11138	0.05981
H	4.95460	-0.78382	2.48101
C	3.32543	-1.47591	1.24540
H	2.55785	-1.11518	1.94005
H	6.04800	-2.66187	-1.24121
C	1.02367	-1.04203	-2.82589
O	2.01780	-1.50379	-3.33452
O	0.59806	-1.24012	-1.60957
C	3.32856	1.16177	-0.91948
H	4.18556	0.52124	-0.64271
H	2.66298	0.56061	-1.55186
H	3.71452	1.99997	-1.53714
C	-0.04924	2.82424	2.60265
H	-0.90863	2.16759	2.39059
H	0.06317	2.90114	3.69880
H	-0.29229	3.83983	2.21737
C	-2.18119	-3.45886	-0.93183
H	-1.41422	-4.15455	-0.53393
H	-2.93663	-4.06367	-1.46701
H	-1.69123	-2.80560	-1.66890
C	-3.35064	0.00840	2.69886
H	-4.34377	0.42235	2.41450
H	-3.35782	-0.15622	3.79157
H	-2.58941	0.77347	2.47479
H	-3.65780	4.01674	-2.52320
H	-4.69175	1.85875	-1.82753
H	-1.27417	4.47980	-1.95235

TS3_Fe_L5

Fe	-0.33769	0.48285	-0.01600
H	-0.18664	-0.18174	-3.22770
P	-0.85382	-1.45518	0.64068
N	0.60167	-2.38873	0.04557
C	1.67189	-1.62291	-0.23378
N	2.87288	-2.17845	-0.44496
C	3.86344	-1.31523	-0.57131
N	3.80623	-0.00263	-0.42001
C	2.57732	0.48742	-0.22542
N	1.45092	-0.27879	-0.24774
N	2.39251	1.79535	0.03867
P	0.69693	2.24889	0.48478
N	0.64402	3.82179	-0.27679
C	0.49340	4.93380	0.62255
H	-0.57939	5.18761	0.77372
H	0.98565	5.84046	0.21969
C	1.12664	4.49072	1.93069
H	2.21545	4.72496	1.94552
H	0.67971	5.01396	2.79627
N	0.87841	3.07661	2.00576
N	-2.10703	-2.55417	0.20204
C	-2.68704	-3.34734	1.25120
H	-2.84957	-4.39082	0.91333
H	-3.68356	-2.94928	1.53891
C	-1.71073	-3.29168	2.41745
H	-2.23488	-3.35087	3.38929
H	-1.00608	-4.15326	2.38857
N	-1.01809	-2.03618	2.28477
H	-1.78514	1.06004	0.40523
Si	-2.56804	1.71259	-0.92887
H	-2.51645	3.01596	-0.15316
H	-3.04041	2.30747	-2.25010
C	-6.25035	-0.26859	-1.12346
C	-6.38194	-0.85443	0.13376
C	-5.11320	0.47899	-1.42665
H	-7.27178	-1.44295	0.37663
H	-5.00734	0.93187	-2.41798
C	-5.36957	-0.69281	1.07954
C	-4.08381	0.65109	-0.49264
H	-5.46455	-1.15120	2.06858
C	-4.22982	0.04147	0.76130
H	-3.42495	0.13732	1.49888
H	-7.03487	-0.40070	-1.87431
C	-1.22038	0.16439	-2.98048
O	-2.17302	-0.09007	-3.67685
O	-1.22595	0.84321	-1.86473
C	-2.78056	-2.54278	-1.06164
H	-3.84782	-2.27084	-0.95658
H	-2.32151	-1.81253	-1.73905
H	-2.72824	-3.53357	-1.55365
C	0.05935	-1.79838	3.19595
H	0.53086	-0.83295	2.95056
H	-0.30104	-1.75095	4.23849
H	0.84343	-2.58533	3.14535
C	0.10885	3.97953	-1.59777
H	-0.95958	4.27828	-1.59211
H	0.66960	4.74797	-2.16068
H	0.18841	3.03243	-2.15029
C	1.35933	2.37250	3.15208
H	2.46117	2.45295	3.27316
H	0.89264	2.74890	4.07989
H	1.10431	1.30559	3.05184
C	3.51760	2.69026	0.12253
H	4.26756	2.43594	-0.63826
H	3.16032	3.71425	-0.05220
H	4.01250	2.64328	1.10842
C	0.73180	-3.82204	0.09035
H	-0.27074	-4.26647	0.02180
H	1.33939	-4.17882	-0.75258
H	1.21790	-4.17089	1.01860
C	5.24418	-1.85982	-0.87284
F	5.24802	-3.17293	-1.07995
F	5.77033	-1.28365	-1.96250
F	6.09432	-1.61514	0.13658

TS3_Fe_L6

Fe	0.32756	-0.37224	-0.23444
H	0.23940	0.58315	2.74222
P	-0.49008	1.52676	-0.77502

N	0.75511	2.63023	-0.09982
C	1.92058	2.02500	0.23596
N	2.98304	2.76134	0.57857
C	4.09828	2.07600	0.79276
N	4.26114	0.76955	0.63157
C	3.15579	0.09892	0.29024
N	1.93031	0.66824	0.16376
N	3.22677	-1.22610	0.00750
P	1.71110	-1.93821	-0.62676
C	0.71196	4.07033	-0.14497
H	1.08028	4.49905	0.79787
H	-0.32289	4.40037	-0.29824
H	1.33737	4.47877	-0.95830
C	4.51301	-1.87493	0.03609
H	5.16411	-1.54396	-0.79224
H	4.37800	-2.96141	-0.03943
H	5.03973	-1.65197	0.97492
H	-0.98693	-1.13533	-0.74439
Si	-1.64877	-1.93615	0.54997
H	-1.49747	-3.19119	-0.30502
H	-2.01796	-2.65859	1.84159
C	-5.45654	-0.15832	0.76402
C	-5.75916	0.10609	-0.57024
C	-4.24881	-0.76935	1.09975
H	-6.70535	0.58781	-0.83542
H	-4.00595	-0.95714	2.14999
C	-4.84444	-0.23908	-1.56565
C	-3.31160	-1.11189	0.11872
H	-5.07269	-0.03275	-2.61585
C	-3.63224	-0.82938	-1.21675
H	-2.90014	-1.06298	-1.99927
H	-6.16399	0.12132	1.55052
C	-0.70521	0.08677	2.40927
O	-1.79467	0.48240	2.76682
O	-0.45441	-0.94087	1.64613
H	4.97737	2.64873	1.11604
C	2.34093	-2.67018	-2.22085
H	3.18773	-3.36877	-2.10270
H	1.49578	-3.21299	-2.67327
H	2.61815	-1.85014	-2.89800
C	-0.66420	2.25921	-2.48142
H	0.25789	2.06440	-3.04651
H	-1.48988	1.72195	-2.97548
H	-0.89476	3.33903	-2.48473
C	-2.02939	2.27844	-0.07018
H	-2.87895	1.70530	-0.47422
H	-2.04176	2.15626	1.02179
H	-2.17131	3.33766	-0.33775
C	1.68026	-3.52631	0.34146
H	0.81554	-4.10796	-0.01689
H	2.58292	-4.15047	0.24065
H	1.50937	-3.28500	1.40084

TS3_Fe_L10

Fe	-0.75099	0.36134	-0.44112
H	-0.72074	-0.62746	2.66782
C	-1.49855	-2.50637	-0.63645
C	-2.70824	-1.69821	-0.32005
C	-3.94496	-2.19962	0.05455
C	-5.03413	-1.32317	0.26867
C	-4.82601	0.06852	0.13217
C	-3.58282	0.55482	-0.24244
C	-2.47157	-0.30221	-0.49201
C	-3.24489	1.98144	-0.49464
H	0.89521	0.88922	-0.74889
Si	1.70854	1.40985	0.51470
H	1.74565	2.83065	-0.06631
H	2.23861	1.83140	1.89598
C	5.52890	-0.37668	0.44993
C	5.71569	-0.71828	-0.89266
C	4.34157	0.22852	0.85806
H	6.64214	-1.20464	-1.21769
H	4.19242	0.48529	1.91316
C	4.70079	-0.44250	-1.81375
C	3.30594	0.50690	-0.04685
H	4.83386	-0.70797	-2.86874
C	3.51334	0.14893	-1.39163
H	2.70510	0.33036	-2.11173
H	6.31346	-0.59522	1.18344
C	0.32222	-0.33888	2.38767
O	1.29310	-0.87226	2.89250
O	0.31818	0.57785	1.47128
H	-6.00988	-1.71296	0.57933
H	-3.32355	2.20976	-1.58481
H	-1.40856	-2.65939	-1.73876
H	-1.47202	-3.49966	-0.13951
H	-3.87664	2.70642	0.06290
H	-5.66049	0.76187	0.31822
H	-4.08896	-3.28329	0.18196
O	-1.86694	2.19520	-0.14173
O	-0.33830	-1.75624	-0.24071
C	-1.37926	3.41177	-0.62255
H	-1.40261	3.42857	-1.73058
H	-1.98302	4.25875	-0.23217
H	-0.33625	3.51910	-0.29500
C	0.85315	-2.31000	-0.71875
H	0.89611	-2.23863	-1.82416
H	1.69414	-1.74737	-0.29152
H	0.93690	-3.37360	-0.41592

TS3_Ni_L2

Ni	0.48438	0.17526	-0.13866
H	0.33503	-1.11816	3.34814
P	-1.22347	1.56844	-0.15845
O	-0.43994	2.87457	0.73014
C	0.84074	2.77587	0.96145
N	1.48527	3.78476	1.51242
C	2.78988	3.60875	1.67476
N	3.49359	2.54232	1.30927
C	2.79553	1.56761	0.76961
N	1.45528	1.62401	0.59919
O	3.41022	0.48103	0.36484
P	2.44593	-0.77750	-0.35899
N	3.09835	-2.12762	0.32439
C	3.66425	-3.04001	-0.66597
H	2.93515	-3.82668	-0.93370
H	4.55048	-3.53222	-0.23837
C	4.01856	-2.17472	-1.86496
H	5.03354	-1.74791	-1.77150
H	3.96792	-2.73460	-2.80997
N	3.01183	-1.11232	-1.87630
N	-2.70690	1.48903	0.53248
C	-3.76340	2.03728	-0.31905
H	-4.45467	2.63047	0.29880
H	-4.33578	1.20985	-0.77468
C	-3.06533	2.89328	-1.36358
H	-3.61514	2.92077	-2.31538
H	-2.93796	3.93433	-1.01056
N	-1.75748	2.26604	-1.56868
H	-0.20582	-0.65572	-1.27406
Si	-0.71359	-2.13251	-0.86403
H	-0.60859	-2.49208	-2.31588
H	0.28038	-2.91443	-0.10584
C	-4.39311	-2.58441	1.00497
C	-5.26602	-2.05954	0.05291
C	-3.01948	-2.57430	0.77973

H	-6.34300	-2.06916	0.23651
H	-2.33014	-2.96839	1.53277
C	-4.76918	-1.53572	-1.13959
C	-2.51001	-2.05905	-0.42065
H	-5.45392	-1.13908	-1.89335
C	-3.39819	-1.54841	-1.37980
H	-3.01777	-1.16949	-2.33575
H	-4.78580	-3.00678	1.93246
C	-0.10094	-1.64948	2.45880
O	-0.24935	-2.85909	2.50103
O	-0.40739	-0.88059	1.47723
C	-3.06800	0.85570	1.78455
H	-3.80419	0.05688	1.60688
H	-2.18229	0.40564	2.24545
H	-3.50061	1.59995	2.47004
C	-0.83726	2.88458	-2.49700
H	0.08397	2.28706	-2.56695
H	-1.28812	2.92579	-3.49817
H	-0.57010	3.91209	-2.19114
C	2.90188	-2.56661	1.69093
H	2.12433	-3.34218	1.77361
H	3.85177	-2.95488	2.08512
H	2.60206	-1.71669	2.31942
C	2.94547	-0.19417	-2.98896
H	3.88044	0.37954	-3.10357
H	2.75598	-0.74428	-3.92194
H	2.11971	0.51722	-2.84080
H	3.34393	4.42632	2.14843

TS3_Ni_L4

Ni	-0.43845	0.11885	0.09638
H	-0.29233	-0.26809	-3.52802
P	1.22393	1.50399	0.19789
N	0.34793	2.95315	-0.23448
C	-1.01666	2.87099	-0.36374
C	-1.80893	3.99585	-0.61471
C	-3.18203	3.82469	-0.70012
C	-3.76264	2.57748	-0.52391
C	-2.92277	1.48704	-0.27901
N	-1.57648	1.64239	-0.22747
N	-3.41060	0.21864	-0.07084
P	-2.27430	-1.03353	0.36706
N	-2.80646	-2.39275	-0.46229
C	-3.10198	-3.51836	0.41347
H	-2.22790	-4.19178	0.49378
H	-3.93616	-4.10439	-0.00445
C	-3.45699	-2.91360	1.76338
H	-4.54166	-2.70064	1.83995
H	-3.19771	-3.58903	2.59303
N	-2.67636	-1.68852	1.85810
N	2.60018	1.57850	-0.74954
C	3.83468	1.46816	0.01713
H	4.61263	2.08378	-0.46206
H	4.19479	0.42462	0.03624
C	3.50601	1.96595	1.41927
H	4.08656	1.42155	2.18132
H	3.73283	3.04222	1.53653
N	2.08826	1.70151	1.60999
C	1.01887	4.23297	-0.34269
H	0.84618	4.69143	-1.32840
H	2.09766	4.07659	-0.23577
H	0.68579	4.93229	0.44016
C	-4.83878	-0.02541	-0.08611
H	-5.35181	0.50931	0.72944
H	-5.01591	-1.09990	0.02938
H	-5.28386	0.27824	-1.04557
H	0.58852	-0.93819	0.58021
Si	0.83573	-2.24100	-0.50356
H	-0.15637	-2.89347	0.41046
H	0.96341	-3.29636	-1.56248
C	4.96955	-2.26383	-0.31740
C	5.25524	-1.93650	1.00663
C	3.64544	-2.33130	-0.74707
H	6.29213	-1.90245	1.34921
H	3.42153	-2.61593	-1.78101
C	4.21760	-1.66305	1.89763
C	2.59256	-2.05325	0.13499
H	4.44037	-1.42092	2.94031
C	2.89816	-1.70433	1.45754
H	2.08891	-1.47836	2.16134
H	5.78113	-2.48294	-1.01476
C	0.40728	-1.00910	-3.06716
O	1.08510	-1.74529	-3.74162
O	0.39037	-0.93702	-1.76381
C	2.63238	1.28879	-2.16245
H	2.90673	0.23956	-2.36844
H	1.64935	1.48870	-2.61171
H	3.36549	1.94339	-2.65749
C	1.50726	1.73031	2.92465
H	0.46709	1.37353	2.88292
H	2.06472	1.06635	3.60577
H	1.50335	2.74376	3.36124
C	-2.49623	-2.65801	-1.84760
H	-1.60390	-3.30037	-1.96441
H	-3.34506	-3.16485	-2.33027
H	-2.32300	-1.71423	-2.38316
C	-2.80450	-0.86460	3.03348
H	-3.82850	-0.46281	3.15237
H	-2.55628	-1.44369	3.93497
H	-2.10641	-0.01603	2.97508
H	-3.81884	4.68961	-0.89666
H	-4.84218	2.45202	-0.56846
H	-1.35965	4.97965	-0.73172

TS3_Ni_L7

Ni	0.51271	0.07558	-0.24335
H	0.33270	-1.34092	2.77063
P	-0.84120	1.74710	-0.08016
N	0.27342	3.02949	0.18006
C	1.59237	2.68929	0.18617
N	2.52329	3.61945	0.33399
C	3.77518	3.18641	0.31097
N	4.16821	1.93225	0.14106
C	3.19816	1.04330	0.00060
N	1.88444	1.37456	0.03401
N	3.49255	-0.27534	-0.18625
P	2.14855	-1.32972	-0.31215
O	2.37218	-2.37962	0.90024
C	2.28944	-3.73580	0.43201
H	1.24762	-4.07856	0.53096
H	2.93190	-4.34972	1.07370
C	2.77263	-3.68845	-1.01468
H	3.86316	-3.81209	-1.09296
H	2.27580	-4.42700	-1.65450
O	2.43107	-2.37683	-1.50880
O	-1.90419	1.90684	1.12417
C	-3.24929	2.00628	0.62183
H	-3.81777	2.63243	1.31940
H	-3.69166	0.99893	0.58369
C	-3.11901	2.63289	-0.76055
H	-3.89436	2.28516	-1.45335
H	-3.12361	3.73283	-0.72558
O	-1.84299	2.19810	-1.26754
C	-0.17263	4.41047	0.33025
H	0.66087	5.01603	0.69962
H	-0.99528	4.44778	1.05595

H	-0.50823	4.81240	-0.63585
C	4.86181	-0.78017	-0.19568
H	5.03676	-1.35175	-1.11678
H	5.03344	-1.42261	0.67843
H	5.55149	0.06893	-0.16313
H	-0.40109	-0.62383	-1.37780
Si	-1.31530	-1.90242	-0.98551
H	-1.45480	-2.08388	-2.47710
H	-0.54596	-3.08853	-0.54460
C	-4.90011	-1.42696	1.11035
C	-5.72888	-0.87332	0.13398
C	-3.57047	-1.71589	0.81890
H	-6.77187	-0.64692	0.36777
H	-2.92990	-2.15503	1.58697
C	-5.23203	-0.62699	-1.14413
C	-3.05504	-1.47421	-0.46367
H	-5.88324	-0.21112	-1.91695
C	-3.90662	-0.93736	-1.44173
H	-3.53124	-0.77048	-2.45716
H	-5.29468	-1.63837	2.10650
C	-0.31653	-1.87085	2.03020
O	-0.66439	-3.01681	2.20437
O	-0.64552	-1.14038	1.00348
H	4.56137	3.93790	0.44132

TS3_Ni_L9

Ni	0.50653	-0.16662	-0.42326
H	-0.36865	-1.11623	3.12030
P	-0.39390	1.79592	-0.28466
N	0.90597	2.80178	0.19546
C	2.12832	2.12758	0.31032
C	3.30839	2.80116	0.65942
C	4.49585	2.08134	0.73925
C	4.53974	0.71743	0.46879
C	3.35194	0.05861	0.12013
C	2.12536	0.74427	0.04968
N	3.33473	-1.31109	-0.18273
P	1.78546	-1.90041	-0.59532
C	0.80829	4.21894	0.41993
H	1.10663	4.48982	1.44736
H	-0.22741	4.55378	0.27927
H	1.44224	4.78956	-0.28144
C	4.53651	-2.10100	-0.15994
H	5.28909	-1.71813	-0.87111
H	4.31310	-3.13825	-0.44136
H	4.99417	-2.11983	0.84433
H	-0.79988	-0.80530	-1.04651
Si	-1.84872	-1.90084	-0.28629
H	-1.82205	-2.46890	-1.70493
H	-1.88647	-3.21436	0.43924
C	-5.32877	0.18230	0.76838
C	-5.66446	0.82290	-0.42270
C	-4.21333	-0.65151	0.82634
H	-6.53411	1.48412	-0.46410
H	-3.94359	-1.13984	1.76776
C	-4.89309	0.61128	-1.56458
C	-3.42125	-0.86907	-0.30973
H	-5.15862	1.10212	-2.50486
C	-3.78912	-0.23548	-1.50476
H	-3.19220	-0.40905	-2.40839
H	-5.93781	0.33707	1.66263
C	-1.20870	-1.47703	2.47617
O	-2.15067	-2.07367	2.94145
O	-1.02029	-1.15764	1.22546
H	5.41751	2.60094	1.01389
H	5.48797	0.18071	0.52967
H	3.30770	3.87264	0.86623
C	1.95108	-2.69086	-2.23211
H	2.68789	-3.50810	-2.22676
H	0.96955	-3.10016	-2.51685
H	2.24322	-1.93356	-2.97163
C	-1.08193	2.55009	-1.79853
H	-0.33717	2.50209	-2.60377
H	-1.97119	1.97366	-2.09483
H	-1.38158	3.59570	-1.63211
C	-1.71855	2.08802	0.93099
H	-1.98804	3.15326	0.97987
H	-2.60685	1.50905	0.63620
H	-1.38859	1.74285	1.91981
C	1.49503	-3.32870	0.50512
H	0.52555	-3.78805	0.25847
H	2.28241	-4.08952	0.39953
H	1.45798	-2.97922	1.54568

TS3_Ni_L10

Ni	0.81383	-0.20765	-0.60667
H	0.21714	-0.57267	3.01402
C	2.59916	1.81835	0.10559
C	3.86090	2.33716	0.39627
C	4.96242	1.47839	0.41784
C	4.82401	0.11371	0.15384
C	3.55890	-0.39776	-0.13624
C	2.46767	0.46196	-0.16339
H	-0.65392	-0.80367	-1.05916
Si	-1.73652	-1.65593	-0.05255
H	-1.82992	-2.51290	-1.31109
H	-1.94111	-2.76677	0.94030
C	-4.80205	1.09508	0.70085
C	-5.24768	1.40306	-0.58309
C	-3.78253	0.16279	0.88467
H	-6.04549	2.13705	-0.72529
H	-3.43429	-0.07495	1.89438
C	-4.67957	0.76649	-1.68558
C	-3.18910	-0.47524	-0.21324
H	-5.03010	0.99936	-2.69457
C	-3.66365	-0.16669	-1.49615
H	-3.21648	-0.66005	-2.36676
H	-5.25421	1.58407	1.56767
C	-0.74966	-0.83067	2.51607
O	-1.74989	-1.05503	3.15242
O	-0.62924	-0.83062	1.21409
H	5.95213	1.88215	0.64432
H	5.70198	-0.53867	0.17741
H	3.99498	3.40233	0.60699
C	3.21758	-1.82388	-0.41030
H	3.84931	-2.28800	-1.19014
H	3.30096	-2.44859	0.50025
C	1.31245	2.57168	0.07093
H	0.98927	2.87229	1.08699
H	1.35380	3.48814	-0.54660
O	1.85127	-1.85507	-0.85551
O	0.31727	1.69559	-0.47789
C	1.29132	-3.15504	-0.87223
H	1.13384	-3.51831	0.15745
H	0.33186	-3.10943	-1.40025
H	1.96877	-3.84092	-1.40421
C	-1.00429	2.17821	-0.29750
H	-1.06442	3.21443	-0.66547
H	-1.68832	1.54154	-0.86707
H	-1.28221	2.14621	0.76858

4.5.4. TS4

TS4_Co_L2

Co	-0.14466	0.36474	-0.59340
H	1.11775	-1.51244	-2.17944
P	1.85289	0.88275	-0.18837
O	1.69953	2.68765	-0.21614
C	0.47870	3.14080	-0.18694
N	0.26113	4.43734	-0.00732
C	-1.01369	4.77375	0.13495
N	-2.04900	3.94964	0.21438
C	-1.75804	2.66812	0.02615
N	-0.52044	2.23793	-0.30425
O	-2.67837	1.76430	0.19677
P	-2.10937	0.03918	0.16867
N	-3.42547	-0.68648	-0.55931
C	-4.41911	-1.22690	0.34530
H	-4.38164	-2.33438	0.33115
H	-5.43453	-0.92866	0.03228
C	-4.07515	-0.67532	1.72055
H	-4.60748	0.27971	1.90801
H	-4.34645	-1.37299	2.52896
N	-2.63646	-0.45705	1.70615
N	3.17188	0.66644	-1.19769
C	4.45109	0.53253	-0.53319
H	5.21945	1.14508	-1.03579
H	4.79237	-0.52094	-0.57132
C	4.22221	0.98918	0.89991
H	4.87245	0.45634	1.61363
H	4.42527	2.07475	1.00697
N	2.82810	0.69964	1.18405
H	0.25527	-1.08180	-0.50935
Si	-0.85948	-3.50563	-0.88057
H	-2.13847	-3.35521	-0.12995
H	-0.82732	-4.79416	-1.62012
C	1.43668	-2.54849	2.44779
C	2.74051	-2.67684	1.97569
C	0.36431	-2.86265	1.61811
H	3.58388	-2.40815	2.61799
H	-0.65880	-2.70778	1.97825
C	2.97164	-3.15053	0.68582
C	0.57387	-3.32413	0.31076
H	3.99422	-3.26464	0.31763
C	1.89542	-3.48127	-0.13301
H	2.08924	-3.85004	-1.14697
H	1.25368	-2.18292	3.46170
C	0.05880	-1.19112	-2.05170
O	-0.33783	-0.14266	-2.62628
O	-0.77972	-2.29414	-2.04432
H	-1.23112	5.84485	0.22462
C	3.08777	0.45613	-2.61812
H	3.48143	-0.53911	-2.89618
H	2.04115	0.52238	-2.94975
H	3.67046	1.21477	-3.16745
C	2.32995	1.08288	2.47671
H	2.85655	0.52734	3.26935
H	2.45604	2.16605	2.66935
H	1.25919	0.84095	2.54457
C	-3.60272	-0.84609	-1.97963
H	-3.73372	-1.91136	-2.23921
H	-4.49109	-0.29370	-2.33062
H	-2.71597	-0.47919	-2.51352
C	-2.06552	0.15235	2.87905
H	-2.55478	1.11419	3.12806
H	-2.16386	-0.51865	3.74694
H	-0.99487	0.34365	2.71388

TS4_Co_L3

Co	-0.40133	0.24919	-0.73420
H	1.53147	-0.96387	-2.31838
P	1.05890	1.70222	-0.23306
C	0.09513	3.28937	-0.53850
C	-1.35330	2.99370	-0.46555
N	-2.21623	3.96243	-0.18981
C	-3.49192	3.59691	-0.11097
N	-3.95469	2.37015	-0.27993
C	-3.04512	1.43849	-0.56368
N	-1.71973	1.70583	-0.65903
C	-3.42588	0.02569	-0.66937
P	-2.03299	-0.99247	0.16121
N	-2.59552	-2.58204	-0.08005
C	-3.13397	-3.21956	1.09941
H	-2.40017	-3.92087	1.54812
H	-4.03215	-3.80902	0.84139
C	-3.46740	-2.09213	2.05742
H	-4.48409	-1.69194	1.84873
H	-3.45382	-2.41702	3.11077
N	-2.45297	-1.07801	1.83328
N	2.55383	2.04564	-1.02458
C	3.64307	2.31968	-0.10907
H	4.30000	3.11512	-0.50364
H	4.26402	1.41213	0.03259
C	2.99903	2.72144	1.20415
H	3.66839	2.53658	2.06258
H	2.75202	3.80682	1.21055
N	1.81617	1.89829	1.30560
H	0.73527	-0.76995	-0.64004
Si	1.05624	-3.27539	-0.24494
H	0.00548	-3.22390	0.81174
H	1.39203	-4.68252	-0.58869
C	3.59255	-0.87941	2.02574
C	4.78552	-0.91131	1.30891
C	2.48476	-1.58283	1.56079
H	5.65663	-0.35911	1.67268
H	1.54185	-1.53393	2.11695
C	4.86850	-1.63897	0.12214
C	2.55192	-2.33575	0.38088
H	5.80223	-1.65817	-0.44532
C	3.76006	-2.34737	-0.33265
H	3.83604	-2.92498	-1.26097
H	3.52172	-0.29657	2.94769
C	0.51184	-1.23893	-1.96836
O	-0.48473	-0.75135	-2.57952
O	0.48933	-2.60976	-1.67499
H	-4.22180	4.38178	0.11954
C	2.91592	1.38342	-2.25033
H	3.44350	0.42405	-2.06596
H	2.01971	1.17918	-2.85408
H	3.58441	2.02426	-2.84855
C	1.01082	2.04397	2.48157
H	1.61262	1.84282	3.38341
H	0.57458	3.06035	2.59202
H	0.18878	1.31355	2.45811
C	-2.45651	-3.35139	-1.28492
H	-1.81185	-4.23858	-1.13537
H	-3.44157	-3.71062	-1.63383
H	-1.99630	-2.74624	-2.07627
C	-2.64912	0.15844	2.54308
H	-3.63221	0.62480	2.32104
H	-2.59403	-0.00833	3.63015
H	-1.86125	0.87700	2.27223
H	-4.45236	-0.15140	-0.32069
H	0.35191	4.15867	0.08598

H	0.34990	3.54960	-1.58032
H	-3.31937	-0.32659	-1.71059

TS4_Co_L4

Co	-0.35692	0.21230	-0.49995
H	1.69965	-0.86628	-2.03074
P	1.20451	1.56903	-0.09691
N	0.30383	3.09349	-0.17435
C	-1.06020	2.98598	-0.19515
C	-1.90820	4.10049	-0.10966
C	-3.27753	3.88156	-0.07629
C	-3.79250	2.59388	-0.07539
C	-2.89343	1.51891	-0.15455
N	-1.55961	1.72860	-0.27953
N	-3.28896	0.21368	-0.07709
P	-1.98740	-0.98600	0.20885
N	-2.66402	-2.38995	-0.44499
C	-3.14716	-3.37017	0.49773
H	-2.46582	-4.24387	0.53893
H	-4.13767	-3.75330	0.18659
C	-3.22656	-2.66692	1.84881
H	-4.25016	-2.27886	2.04029
H	-2.98978	-3.35318	2.67969
N	-2.25868	-1.59145	1.79416
N	2.55444	1.90115	-1.09694
C	3.82906	1.79250	-0.42297
H	4.56575	2.48166	-0.87213
H	4.23919	0.76382	-0.51349
C	3.55721	2.12927	1.03414
H	4.26938	1.61622	1.70528
H	3.66299	3.21906	1.22411
N	2.21337	1.66691	1.28152
C	0.93852	4.38087	-0.06515
H	0.61082	5.06083	-0.86805
H	2.02122	4.24913	-0.17485
H	0.73469	4.86637	0.90594
C	-4.68806	-0.11386	0.00787
H	-5.14662	0.21346	0.95922
H	-4.80365	-1.20097	-0.07942
H	-5.25167	0.34327	-0.82123
H	0.67570	-0.91588	-0.47168
Si	0.97367	-3.42925	-0.57174
H	-0.09688	-3.50334	0.46386
H	1.26420	-4.77401	-1.13562
C	3.57281	-1.37319	1.96294
C	4.79548	-1.46793	1.30287
C	2.43914	-1.96840	1.41612
H	5.68687	-1.00306	1.73368
H	1.47615	-1.86686	1.92944
C	4.88341	-2.15518	0.09328
C	2.50714	-2.67394	0.20632
H	5.84112	-2.22912	-0.42790
C	3.74717	-2.75385	-0.44493
H	3.82623	-3.29696	-1.39350
H	3.50176	-0.83135	2.90960
C	0.61887	-1.10465	-1.88427
O	-0.24291	-0.42561	-2.51496
O	0.46546	-2.50095	-1.87127
H	-3.95835	4.73449	-0.02086
C	2.52173	1.65459	-2.51528
H	3.08587	0.74003	-2.78345
H	1.48416	1.51845	-2.85512
H	2.96573	2.49589	-3.07363
C	1.66002	1.79856	2.59434
H	2.28701	1.28028	3.34004
H	1.55973	2.85610	2.91328
H	0.65880	1.34080	2.61653
C	-2.70112	-2.68135	-1.85231
H	-2.22737	-3.65526	-2.07002
H	-3.74198	-2.71669	-2.22690
H	-2.14257	-1.91554	-2.40931
C	-2.22082	-0.67944	2.90158
H	-3.18144	-0.14118	3.04539
H	-1.98942	-1.21206	3.83805
H	-1.43403	0.07192	2.73279
H	-4.86393	2.41837	0.00265
H	-1.49949	5.10835	-0.06344

TS4_Co_L6

Co	-0.37861	-0.11633	-0.09009
H	1.90256	-0.04547	-1.65210
P	0.15911	1.92688	0.17180
N	-1.39846	2.69850	-0.17106
C	-2.45881	1.85531	-0.22274
N	-3.69660	2.34431	-0.34225
C	-4.65831	1.43347	-0.31127
N	-4.51581	0.13155	-0.12067
C	-3.24950	-0.28577	-0.00920
N	-2.18878	0.53859	-0.11837
N	-2.98629	-1.58675	0.25189
P	-1.27829	-2.00417	0.51645
H	1.07269	-0.48461	0.04057
Si	2.80111	-2.41641	-0.21770
H	2.12834	-2.88659	1.02827
H	3.64999	-3.48880	-0.79784
C	4.26046	1.05621	1.56931
C	5.21343	1.50245	0.65540
C	3.56328	-0.12468	1.32583
H	5.75920	2.43049	0.84391
H	2.79971	-0.45584	2.03786
C	5.47487	0.76112	-0.49512
C	3.80948	-0.88393	0.17306
H	6.22384	1.10678	-1.21179
C	4.78120	-0.42409	-0.72739
H	4.99512	-1.00022	-1.63454
H	4.06254	1.63110	2.47801
C	1.09584	-0.79648	-1.48689
O	-0.00045	-0.65107	-2.08944
O	1.64889	-2.07179	-1.39022
H	-5.68415	1.80045	-0.44079
C	-4.09707	-2.49970	0.42571
H	-4.79216	-2.42535	-0.42098
H	-3.72210	-3.52818	0.48004
H	-4.66314	-2.27943	1.34452
C	-1.63044	4.12651	-0.21482
H	-2.12253	4.48864	0.70205
H	-0.67296	4.64852	-0.33146
H	-2.27585	4.38410	-1.06474
C	-1.36456	-2.79208	2.17654
H	-2.06319	-3.64196	2.22202
H	-0.35539	-3.15672	2.42127
H	-1.64527	-2.03658	2.92281
C	0.68002	2.82310	1.69160
H	-0.00010	2.56807	2.51528
H	1.68869	2.46797	1.95105
H	0.71745	3.91585	1.55899
C	1.28579	2.70548	-1.04935
H	1.30537	3.80426	-1.00219
H	2.30128	2.33317	-0.83155
H	1.00234	2.37473	-2.05841
C	-1.10474	-3.48447	-0.54827
H	-1.16456	-3.16545	-1.59739
H	-0.09818	-3.89682	-0.38673
H	-1.84643	-4.26731	-0.33344

TS4_Co_L7

Co	-0.39902	0.09645	-0.40069
H	1.70151	-0.30386	-2.15678
P	0.64054	1.84664	0.03150
N	-0.62259	3.05460	-0.10456
C	-1.87863	2.54252	-0.14779
N	-2.94685	3.33766	-0.09306
C	-4.11028	2.70462	-0.05337
N	-4.30088	1.39750	0.03458
C	-3.18907	0.66109	-0.01820
N	-1.96629	1.19663	-0.21632
N	-3.23147	-0.68033	0.15935
P	-1.66010	-1.47802	0.32016
O	-1.98894	-2.87893	-0.49992
C	-2.05862	-4.02750	0.31897
H	-1.11101	-4.58664	0.24327
H	-2.87154	-4.67430	-0.04376
C	-2.30956	-3.53504	1.74348
H	-3.38577	-3.51942	1.98640
H	-1.79648	-4.15334	2.49319
O	-1.78439	-2.22147	1.80362
O	1.78713	2.47727	-0.99812
C	3.02516	2.67951	-0.34247
H	3.57896	3.46923	-0.86840
H	3.62086	1.74709	-0.36411
C	2.66469	3.06316	1.08466
H	3.44758	2.78046	1.80324
H	2.47547	4.14688	1.17789
O	1.48416	2.33968	1.38331
C	-0.38622	4.47749	0.01454
H	-1.23058	5.02607	-0.41740
H	0.52869	4.73559	-0.53519
H	-0.27524	4.77762	1.06821
C	-4.49094	-1.35902	0.37938
H	-4.72566	-1.43572	1.45279
H	-4.43752	-2.36437	-0.05878
H	-5.29789	-0.80197	-0.10942
H	0.93111	-0.65172	-0.50361
Si	1.85696	-2.98574	-0.62126
H	0.89883	-3.30780	0.47842
H	2.44808	-4.22065	-1.19577
C	3.93257	-0.31696	1.81897
C	5.12083	-0.10723	1.12224
C	2.97037	-1.18173	1.30395
H	5.88095	0.56688	1.52726
H	2.03282	-1.32576	1.85122
C	5.34260	-0.75781	-0.09067
C	3.17672	-1.85414	0.09008
H	6.27340	-0.59370	-0.63907
C	4.37664	-1.62343	-0.59849
H	4.56259	-2.13347	-1.55026
H	3.74949	0.19487	2.76702
C	0.77384	-0.87108	-1.90411
O	-0.32157	-0.49289	-2.41563
O	1.04822	-2.24186	-1.88346
H	-5.00991	3.33163	-0.07024

TS4_Co_L8

Co	-0.73269	-0.17463	-0.33873
H	1.75918	-0.08306	-1.56382
C	-2.76219	1.70921	-0.15457
N	-3.93105	2.19409	0.18870
C	-4.81470	1.30763	0.66768
N	-4.60376	-0.01350	0.74223
C	-3.41277	-0.42544	0.37885
N	-2.41760	0.40820	-0.00363
H	0.70284	-0.39388	0.04943
Si	2.73609	-2.14626	0.16035
H	2.04058	-2.55888	1.41583
H	3.73900	-3.16916	-0.23300
C	3.82631	1.50767	1.84360
C	4.57984	2.10699	0.83664
C	3.29677	0.23366	1.64884
H	4.99113	3.10897	0.98360
H	2.68592	-0.22067	2.43669
C	4.81258	1.42617	-0.35761
C	3.51873	-0.46818	0.45683
H	5.40523	1.89277	-1.14813
C	4.29185	0.14770	-0.53877
H	4.48048	-0.37893	-1.48131
H	3.64646	2.03758	2.78237
C	0.98879	-0.87099	-1.40659
O	-0.00658	-0.89316	-2.17826
O	1.64334	-2.07353	-1.10970
H	-5.78611	1.68618	0.99609
C	-2.99641	-1.84811	0.22774
H	-3.54720	-2.52404	0.90719
H	-3.23396	-2.15117	-0.80769
C	-1.67467	2.47160	-0.83371
H	-1.82253	2.36726	-1.92351
H	-1.70189	3.54979	-0.59163
N	-0.36911	1.86204	-0.50016
N	-1.53125	-1.96426	0.39894
C	0.62204	2.25945	-1.49978
H	0.62922	3.35768	-1.62672
H	1.62143	1.94670	-1.16922
H	0.39116	1.77540	-2.45828
C	0.05667	2.33578	0.82154
H	-0.70833	2.09551	1.57143
H	0.99356	1.84188	1.10567
H	0.21124	3.43288	0.80458
C	-1.22428	-2.01622	1.83124
H	-1.67788	-2.91625	2.29228
H	-0.13762	-2.04595	1.97338
H	-1.61749	-1.12215	2.33328
C	-1.08127	-3.19684	-0.25054
H	-1.16500	-3.08633	-1.33901
H	-0.02833	-3.37441	-0.01331
H	-1.68199	-4.05795	0.09717

TS4_Co_L9

Co	-0.38176	-0.12261	-0.24386
H	1.91751	0.07806	-1.83838
P	0.00767	1.93508	0.00986
N	-1.50453	2.73070	-0.31985
C	-2.58286	1.83112	-0.21930
C	-3.91682	2.27112	-0.18066
C	-4.93262	1.32916	-0.02496
C	-4.63612	-0.02548	0.12002
C	-3.29254	-0.43824	0.07993
C	-2.23551	0.46935	-0.12365
N	-2.91978	-1.77777	0.27035
P	-1.19519	-1.98881	0.42604
H	1.09198	-0.38685	-0.11060
Si	2.76146	-2.26939	-0.23931
H	2.00187	-2.73612	0.95550
H	3.68952	-3.35182	-0.68732
C	4.22698	1.13986	1.69119
C	5.30693	1.51149	0.89112
C	3.47619	0.01212	1.36831
H	5.88851	2.40457	1.13684
H	2.60294	-0.25109	1.97540
C	5.63849	0.74722	-0.22512
C	3.79429	-0.77199	0.25017

H	6.48134	1.03791	-0.85847
C	4.88939	-0.38679	-0.53464
H	5.15586	-0.98169	-1.41590
H	3.95942	1.74170	2.56423
C	1.14026	-0.69791	-1.63713
O	0.03739	-0.63133	-2.24208
O	1.79403	-1.96122	-1.55649
H	-5.97633	1.65916	0.00062
C	-3.90199	-2.75502	0.62243
H	-4.68356	-2.84345	-0.15449
H	-3.43844	-3.74534	0.72629
H	-4.41420	-2.52065	1.57883
C	-1.72679	4.13907	-0.21156
H	-2.19762	4.42804	0.75182
H	-0.77537	4.68243	-0.29774
H	-2.38302	4.50718	-1.02046
C	-1.05346	-2.68488	2.14128
H	-1.63771	-3.60815	2.29449
H	0.00777	-2.89980	2.34006
H	-1.38791	-1.91267	2.84907
C	0.52142	2.72753	1.60902
H	-0.19585	2.42129	2.38420
H	1.51567	2.34202	1.88107
H	0.56938	3.82893	1.55855
C	1.18617	2.80924	-1.11330
H	1.17208	3.90635	-1.01274
H	2.20248	2.45174	-0.87542
H	0.94958	2.52706	-2.14944
C	-0.92931	-3.54004	-0.53875
H	-1.01768	-3.29003	-1.60504
H	0.09651	-3.89565	-0.35856
H	-1.63168	-4.34576	-0.27265
H	-5.44765	-0.74244	0.26970
H	-4.16949	3.33141	-0.26473

TS4_Fe_L1

Fe	-0.187820000	0.291091000	-0.688770000
H	1.412710000	-1.253070000	-2.254550000
P	1.720181000	1.001020000	-0.205540000
N	1.430051000	2.779420000	-0.076600000
C	0.128731000	3.135511000	-0.051070000
N	-0.227218000	4.401961000	0.206180000
C	-1.531078000	4.602141000	0.326710000
N	-2.473819000	3.671091000	0.332780000
C	-2.041939000	2.427781000	0.078120000
N	-0.760839000	2.130641000	-0.262080000
N	-2.874309000	1.373561000	0.188060000
P	-2.066160000	-0.253199000	0.072250000
N	-3.349100000	-1.159678000	-0.651410000
C	-4.130610000	-2.008448000	0.201320000
H	-3.874531000	-3.076948000	0.029280000
H	-5.216010000	-1.911468000	-0.009630000
C	-3.813300000	-1.596778000	1.636910000
H	-4.580260000	-0.890878000	2.029200000
H	-3.823300000	-2.470838000	2.316270000
N	-2.507390000	-0.993979000	1.599620000
N	3.158211000	1.021720000	-1.202530000
C	4.360761000	0.554099000	-0.571790000
H	5.253951000	1.078179000	-0.966590000
H	4.516010000	-0.533131000	-0.761500000
C	4.177761000	0.810239000	0.916340000
H	4.754750000	0.087249000	1.524960000
H	4.550861000	1.823269000	1.193820000
N	2.769481000	0.666730000	1.151570000
C	2.462741000	3.750160000	0.166670000
H	2.633761000	3.916650000	1.245500000
H	2.191412000	4.716490000	-0.279440000
H	3.395241000	3.390260000	-0.290720000
C	-4.255289000	1.571072000	0.537480000
H	-4.404579000	1.660162000	1.628810000
H	-4.839459000	0.716672000	0.167120000
H	-4.636289000	2.492452000	0.076540000
H	0.366120000	-1.132069000	-0.596230000
Si	-0.381601000	-3.512209000	-1.002320000
H	-1.658021000	-3.404959000	-0.238180000
H	-0.322641000	-4.858209000	-1.650610000
C	1.770270000	-2.749380000	2.480760000
C	3.096840000	-2.779690000	2.056120000
C	0.745240000	-3.004180000	1.574940000
H	3.903360000	-2.555910000	2.760710000
H	-0.298080000	-2.912269000	1.897090000
C	3.393869000	-3.080150000	0.728800000
C	1.022419000	-3.314030000	0.236120000
H	4.433369000	-3.090681000	0.388360000
C	2.363899000	-3.355020000	-0.167950000
H	2.607619000	-3.576250000	-1.213520000
H	1.530870000	-2.493670000	3.517010000
C	0.318130000	-1.112769000	-2.091220000
O	-0.260210000	-0.154289000	-2.708430000
O	-0.273220000	-2.416109000	-2.242260000
H	-1.861038000	5.640731000	0.465750000
C	-3.411870000	-1.376658000	-2.067330000
H	-3.284070000	-2.449288000	-2.316800000
H	-4.381610000	-1.048718000	-2.490410000
H	-2.598120000	-0.826659000	-2.564460000
C	-2.017770000	-0.422689000	2.814220000
H	-2.693190000	0.359051000	3.226210000
H	-1.887040000	-1.193649000	3.596220000
H	-1.036870000	0.042321000	2.620340000
C	3.053171000	0.859010000	-2.623650000
H	3.352760000	-0.160450000	-2.946490000
H	2.011031000	1.016290000	-2.943690000
H	3.698891000	1.577420000	-3.163020000
C	2.287611000	0.828160000	2.484160000
H	1.193941000	0.687570000	2.486700000
H	2.732240000	0.077750000	3.164020000
H	2.506071000	1.834490000	2.904090000

TS4_Fe_L2

Fe	-0.311520000	0.235420000	-0.640680000
H	1.461268000	-1.122313000	-2.196060000
P	1.424162000	1.271017000	-0.189070000
O	0.833815000	3.015408000	-0.245670000
C	-0.459565000	3.127500000	-0.283180000
N	-1.017193000	4.332761000	-0.161510000
C	-2.339303000	4.326413000	-0.067580000
N	-3.126225000	3.263764000	0.018460000
C	-2.500697000	2.091483000	-0.109240000
N	-1.181157000	1.986541000	-0.403690000
O	-3.151739000	0.990814000	0.094880000
P	-2.069611000	-0.523468000	0.179250000
N	-3.191203000	-1.634656000	-0.455060000
C	-4.031214000	-2.319684000	0.489050000
H	-3.696296000	-3.372635000	0.617160000
H	-5.081754000	-2.354713000	0.141830000
C	-3.900313000	-1.542235000	1.788880000
H	-4.655151000	-0.726643000	1.832790000
H	-4.055414000	-2.184714000	2.674090000
N	-2.560682000	-0.998487000	1.777700000
N	2.849362000	1.450205000	-1.121750000
C	4.076772000	1.666383000	-0.405940000
H	4.697824000	2.441132000	-0.895650000
H	4.683441000	0.734502000	-0.371460000
C	3.656193000	2.091713000	0.991770000
H	4.406033000	1.807632000	1.753230000
H	3.532675000	3.196513000	1.044280000

N	2.404032000	1.419495000	1.233900000
H	0.506368000	-1.041282000	-0.474800000
Si	0.178684000	-3.481491000	-0.622620000
H	-0.997066000	-3.415519000	0.295550000
H	0.326372000	-4.880341000	-1.127470000
C	2.685867000	-1.952465000	2.338760000
C	3.909217000	-1.827267000	1.684220000
C	1.593866000	-2.498053000	1.671610000
H	4.759858000	-1.367509000	2.195980000
H	0.617866000	-2.526112000	2.168380000
C	4.041196000	-2.270407000	0.370410000
C	1.703375000	-2.949484000	0.348460000
H	4.996476000	-2.164539000	-0.151590000
C	2.948825000	-2.836786000	-0.284120000
H	3.058955000	-3.166536000	-1.323660000
H	2.572717000	-1.588115000	3.363210000
C	0.366978000	-1.157131000	-1.987390000
O	-0.393491000	-0.359930000	-2.630240000
O	-0.001494000	-2.544191000	-1.985320000
H	-2.832792000	5.307004000	-0.028780000
C	-3.203274000	-2.051526000	-1.827160000
H	-2.975985000	-3.131976000	-1.922250000
H	-4.192393000	-1.873794000	-2.289830000
H	-2.438593000	-1.498107000	-2.391490000
C	-2.231380000	-0.125997000	2.867710000
H	-2.951029000	0.713414000	2.967910000
H	-2.216211000	-0.679657000	3.822310000
H	-1.230310000	0.301591000	2.699760000
C	2.903352000	1.178924000	-2.525410000
H	3.534460000	0.294713000	-2.750600000
H	1.888711000	0.984736000	-2.905180000
H	3.321423000	2.035944000	-3.086530000
C	1.769002000	1.691336000	2.486880000
H	0.791922000	1.183678000	2.514160000
H	2.380082000	1.313455000	3.326260000
H	1.597584000	2.776447000	2.648080000

TS4_Fe_L3

Fe	-0.498750000	0.238690000	-0.757700000
H	1.681770000	-0.813631000	-2.004220000
P	0.996501000	1.687990000	-0.338650000
C	0.067042000	3.302740000	-0.632450000
C	-1.367828000	3.048101000	-0.390830000
N	-2.154737000	4.034091000	0.009620000
C	-3.419678000	3.713782000	0.276970000
N	-3.929498000	2.492642000	0.159410000
C	-3.100739000	1.542492000	-0.250330000
N	-1.770689000	1.744041000	-0.524770000
C	-3.552650000	0.142962000	-0.320330000
P	-2.055320000	-0.955789000	0.081260000
N	-2.676381000	-2.512708000	-0.368350000
C	-3.121881000	-3.349948000	0.708240000
H	-2.373422000	-4.141978000	0.937880000
H	-4.067662000	-3.870128000	0.453970000
C	-3.307301000	-2.424188000	1.898600000
H	-4.329511000	-1.976837000	1.883080000
H	-3.208651000	-2.960588000	2.860360000
N	-2.288430000	-1.412139000	1.769040000
N	2.509561000	2.059179000	-1.160820000
C	3.636962000	2.235968000	-0.285160000
H	4.348222000	2.983668000	-0.688690000
H	4.199581000	1.283368000	-0.157400000
C	3.055652000	2.678239000	1.042860000
H	3.751882000	2.481668000	1.881560000
H	2.858882000	3.777199000	1.035630000
N	1.846811000	1.916689000	1.189990000
H	0.558950000	-0.814180000	-0.392340000
Si	1.045969000	-3.246580000	-0.312870000

H	-0.060281000	-3.298490000	0.683430000
H	1.455958000	-4.634471000	-0.687190000
C	3.420090000	-0.831312000	2.130230000
C	4.686140000	-0.952622000	1.563370000
C	2.343700000	-1.527031000	1.585920000
H	5.529320000	-0.396283000	1.983920000
H	1.342470000	-1.392131000	2.010310000
C	4.873619000	-1.765772000	0.446360000
C	2.511179000	-2.358761000	0.470210000
H	5.863639000	-1.852183000	-0.010190000
C	3.793029000	-2.462632000	-0.089460000
H	3.946519000	-3.097272000	-0.970170000
H	3.263280000	-0.171462000	2.987400000
C	0.616010000	-1.102660000	-1.866390000
O	-0.256950000	-0.581950000	-2.637420000
O	0.587669000	-2.527690000	-1.740940000
H	-4.087417000	4.515882000	0.612560000
C	-2.469611000	-3.105198000	-1.654950000
H	-1.818452000	-4.001649000	-1.597530000
H	-3.427972000	-3.421098000	-2.113190000
H	-1.970871000	-2.389429000	-2.324540000
C	-2.365750000	-0.340288000	2.717950000
H	-3.360110000	0.159932000	2.724410000
H	-2.166300000	-0.702869000	3.741330000
H	-1.607399000	0.419361000	2.470670000
C	2.792631000	1.512059000	-2.455010000
H	3.415021000	0.592198000	-2.395100000
H	1.855321000	1.252709000	-2.970930000
H	3.341252000	2.238888000	-3.083260000
C	1.061262000	2.187290000	2.352600000
H	0.134421000	1.592670000	2.309440000
H	1.604511000	1.907749000	3.274200000
H	0.776222000	3.259500000	2.445080000
H	0.409153000	4.204310000	-0.100570000
H	-4.486260000	-0.009597000	0.242050000
H	0.241992000	3.456050000	-1.711600000
H	-3.721320000	-0.152488000	-1.371410000

TS4_Fe_L4

Fe	-0.19614	0.30124	-0.67735
H	1.45363	-1.18621	-2.31506
P	1.68045	1.08199	-0.21523
N	1.33286	2.83645	-0.11839
C	0.00810	3.15782	-0.04339
C	-0.43742	4.46163	0.23148
C	-1.80011	4.68049	0.38740
C	-2.68514	3.60998	0.33624
C	-2.17681	2.32908	0.06270
N	-0.85910	2.12028	-0.21567
N	-2.94903	1.20529	0.09673
P	-2.03383	-0.35597	0.08115
N	-3.23835	-1.38318	-0.61264
C	-3.98139	-2.24231	0.26155
H	-3.66841	-3.30211	0.13517
H	-5.06830	-2.21202	0.03463
C	-3.70640	-1.75765	1.68405
H	-4.51901	-1.08312	2.04077
H	-3.67632	-2.60349	2.39778
N	-2.43863	-1.07998	1.63600
N	3.12760	1.13994	-1.21448
C	4.30535	0.59220	-0.60176
H	5.22447	1.05812	-1.00870
H	4.38783	-0.50406	-0.79036
C	4.15634	0.85719	0.88817
H	4.71009	0.10821	1.48820
H	4.57938	1.85336	1.15957
N	2.74860	0.77384	1.14045
C	2.34041	3.83631	0.07938

H	2.33881	4.24604	1.10828
H	2.21706	4.68118	-0.62073
H	3.32116	3.38858	-0.12297
C	-4.35876	1.30649	0.33223
H	-4.60420	1.63372	1.36252
H	-4.81898	0.32585	0.15886
H	-4.83138	2.01774	-0.36745
H	0.45858	-1.10216	-0.68013
Si	-0.20877	-3.54179	-0.99917
H	-1.50176	-3.52267	-0.25623
H	-0.03930	-4.88326	-1.63868
C	1.85526	-2.53911	2.47796
C	3.19291	-2.62441	2.09723
C	0.85088	-2.84063	1.56333
H	3.98293	-2.36610	2.80901
H	-0.19948	-2.70716	1.84688
C	3.52254	-3.02241	0.80404
C	1.16155	-3.25303	0.25917
H	4.57110	-3.07577	0.49661
C	2.51240	-3.34255	-0.10158
H	2.78113	-3.64535	-1.12042
H	1.59105	-2.20563	3.48563
C	0.36037	-1.10491	-2.10302
O	-0.29235	-0.18123	-2.69875
O	-0.16209	-2.44382	-2.24117
H	-2.17276	5.68924	0.58730
C	-3.34505	-1.57646	-2.02748
H	-3.19664	-2.63995	-2.30238
H	-4.33865	-1.26785	-2.41198
H	-2.56341	-0.99393	-2.53994
C	-2.01762	-0.40532	2.82310
H	-2.75724	0.34685	3.17784
H	-1.84411	-1.11675	3.65202
H	-1.07112	0.12207	2.61566
C	3.00681	1.00168	-2.63707
H	3.25886	-0.02393	-2.98038
H	1.96953	1.20588	-2.94591
H	3.67798	1.70174	-3.16932
C	2.28289	0.96842	2.47274
H	1.18692	0.84401	2.48764
H	2.72199	0.22587	3.16531
H	2.51886	1.98001	2.87165
H	-3.74861	3.75346	0.52565
H	0.27657	5.27888	0.33071

TS4_Fe_L5

Fe	-0.320380000	0.090231000	-0.750670000
H	-2.470640000	-0.841859000	-2.142920000
P	-0.325600000	-1.967739000	-0.322210000
N	1.426010000	-2.327420000	-0.229870000
C	2.224530000	-1.236270000	-0.209430000
N	3.532510000	-1.358140000	0.010100000
C	4.193360000	-0.213670000	0.111190000
N	3.671951000	1.002310000	0.122940000
C	2.355741000	1.050830000	-0.091980000
N	1.591230000	-0.039390000	-0.388790000
N	1.683261000	2.216750000	0.015910000
P	-0.116019000	2.065021000	-0.032420000
N	-0.536639000	3.569311000	-0.760710000
C	-1.011619000	4.615581000	0.100660000
H	-2.103799000	4.766981000	-0.038340000
H	-0.530708000	5.587101000	-0.135270000
C	-0.698689000	4.183111000	1.531580000
H	0.244051000	4.651140000	1.894080000
H	-1.492979000	4.507461000	2.230910000
N	-0.598519000	2.746861000	1.504950000
N	-0.855620000	-3.287439000	-1.334400000
C	-1.758880000	-4.211939000	-0.704390000
H	-1.645661000	-5.232129000	-1.119760000
H	-2.818880000	-3.910829000	-0.871030000
C	-1.427210000	-4.171389000	0.778670000
H	-2.305470000	-4.436349000	1.397770000
H	-0.629111000	-4.907699000	1.027810000
N	-1.016080000	-2.818599000	1.030970000
C	1.975800000	-3.639240000	-0.007900000
H	2.136890000	-3.846510000	1.064690000
H	2.945430000	-3.738240000	-0.514070000
H	1.279210000	-4.382990000	-0.418410000
C	2.387611000	3.432420000	0.328430000
H	2.586491000	3.536550000	1.409850000
H	1.784331000	4.286540000	-0.008970000
H	3.356711000	3.454220000	-0.187740000
H	-1.833000000	0.057201000	-0.516260000
Si	-3.844439000	1.678471000	-0.803650000
H	-3.255139000	2.871591000	-0.130730000
H	-5.168529000	2.057452000	-1.383430000
C	-3.600490000	-0.482829000	2.745120000
C	-4.169160000	-1.711419000	2.415120000
C	-3.538439000	0.531271000	1.794480000
H	-4.198630000	-2.517669000	3.154240000
H	-3.031849000	1.472811000	2.035310000
C	-4.686860000	-1.916898000	1.138560000
C	-4.057469000	0.346141000	0.505420000
H	-5.122930000	-2.883998000	0.872580000
C	-4.639410000	-0.890809000	0.197680000
H	-5.038100000	-1.066238000	-0.808230000
H	-3.175260000	-0.324459000	3.740440000
C	-1.932979000	0.128041000	-2.016110000
O	-0.878029000	0.320541000	-2.713980000
O	-2.929349000	1.153971000	-2.083430000
C	-0.786939000	3.682941000	-2.169150000
H	-1.847149000	3.936351000	-2.368670000
H	-0.160409000	4.468141000	-2.634340000
H	-0.583589000	2.720211000	-2.662100000
C	-0.182809000	2.101551000	2.711610000
H	0.801591000	2.465580000	3.077320000
H	-0.916659000	2.260511000	3.523230000
H	-0.098889000	1.017761000	2.529290000
C	-1.000080000	-3.088149000	-2.749450000
H	-2.063500000	-2.962379000	-3.041450000
H	-0.465560000	-2.176169000	-3.057340000
H	-0.598650000	-3.944449000	-3.321670000
C	-0.646740000	-2.461539000	2.363550000
H	-0.375050000	-1.393599000	2.384830000
H	-1.488940000	-2.614069000	3.062330000
H	0.219080000	-3.046760000	2.741770000
C	5.686280000	-0.350081000	0.289200000
F	6.237790000	-1.088471000	-0.686120000
F	6.315711000	0.822249000	0.297560000
F	5.991010000	-0.967861000	1.444840000

TS4_Fe_L6

Fe	-0.38833	-0.12805	-0.16121
H	1.83614	0.05414	-1.78078
P	0.08399	1.92229	0.08517
N	-1.51067	2.67785	-0.19357
C	-2.55301	1.80628	-0.20241
N	-3.80667	2.26168	-0.26284
C	-4.74945	1.32945	-0.18624
N	-4.55455	0.02833	-0.00761
C	-3.27497	-0.35258	0.04249
N	-2.22470	0.49299	-0.10665
N	-2.96101	-1.64925	0.29558
P	-1.21440	-1.98625	0.50066
C	-1.78380	4.09169	-0.19689
H	-2.20075	4.43922	0.76538

H	-2.51395	4.34263	-0.97948
H	-0.85599	4.64471	-0.39389
C	-4.03083	-2.58139	0.54353
H	-4.54634	-2.37743	1.49870
H	-3.63007	-3.60256	0.57505
H	-4.78704	-2.52719	-0.25315
H	1.12672	-0.51837	-0.07421
Si	2.82374	-2.32762	-0.26627
H	2.15159	-2.84454	0.96331
H	3.74698	-3.37579	-0.79848
C	4.25770	1.13268	1.60140
C	5.34084	1.49409	0.80129
C	3.52058	-0.01121	1.30334
H	5.91229	2.39856	1.02900
H	2.64461	-0.26534	1.91060
C	5.68945	0.70406	-0.29167
C	3.85438	-0.82048	0.20693
H	6.53567	0.98548	-0.92492
C	4.95244	-0.44417	-0.57830
H	5.23149	-1.05794	-1.44253
H	3.97765	1.75341	2.45718
C	1.07576	-0.71471	-1.50012
O	-0.04246	-0.69554	-2.11250
O	1.76786	-1.98805	-1.49889
H	-5.79043	1.66935	-0.26369
C	-1.26797	-2.85859	2.14730
H	-1.90940	-3.75701	2.16811
H	-0.23527	-3.15727	2.38576
H	-1.59948	-2.14133	2.91153
C	0.63237	2.92706	1.56532
H	-0.01767	2.67389	2.41472
H	1.65712	2.60583	1.80855
H	0.63634	4.01980	1.40294
C	1.14024	2.81609	-1.16000
H	1.10435	3.91593	-1.09521
H	2.18134	2.49619	-0.98064
H	0.84747	2.48680	-2.16750
C	-1.04594	-3.49218	-0.56579
H	-0.05390	-3.93020	-0.37395
H	-1.81109	-4.26312	-0.38148
H	-1.06489	-3.16161	-1.61355

TS4_Fe_L8

Fe	-0.72214	0.22279	0.38788
H	1.86771	0.23989	1.56539
C	-2.46803	-1.85226	-0.31885
N	-3.60895	-2.37126	-0.65986
C	-4.54936	-1.52396	-1.15110
N	-4.31050	-0.20660	-1.38842
C	-3.15239	0.25701	-1.02950
N	-2.18266	-0.49111	-0.37029
H	0.74209	0.43866	0.00189
Si	2.69991	2.09462	-0.49655
H	1.92897	2.37622	-1.74341
H	3.76301	3.13076	-0.32787
C	3.62283	-1.76148	-1.80888
C	4.62147	-2.16821	-0.92489
C	3.06620	-0.48968	-1.69077
H	5.05021	-3.17120	-1.00635
H	2.25459	-0.19206	-2.36275
C	5.06542	-1.29839	0.06892
C	3.50140	0.40350	-0.70207
H	5.84298	-1.61667	0.76904
C	4.51229	-0.02283	0.17103
H	4.86496	0.65399	0.95799
H	3.26263	-2.44488	-2.58235
C	1.06911	0.97094	1.28422
O	0.10700	1.12684	2.09326
O	1.77096	2.17083	0.87917
H	-5.51995	-1.93493	-1.43781
C	-2.61126	1.61396	-1.30540
H	-1.96462	1.56344	-2.20553
H	-3.40922	2.36236	-1.48132
C	-1.24528	-2.58708	0.09624
H	-1.46735	-3.59756	0.49270
H	-0.57147	-2.68450	-0.78076
N	-1.71889	2.01469	-0.18990
N	-0.49662	-1.76531	1.08191
C	-0.90276	3.15212	-0.57664
H	-1.53614	4.02709	-0.83622
H	-0.23304	3.41943	0.25118
H	-0.29249	2.88455	-1.44989
C	-2.51639	2.36775	0.98315
H	-1.84544	2.53764	1.83466
H	-3.11820	3.27927	0.78375
H	-3.19968	1.54367	1.22769
C	-1.17598	-1.79189	2.37513
H	-0.71501	-1.04987	3.04066
H	-2.23483	-1.53496	2.23780
H	-1.11427	-2.80277	2.83047
C	0.85863	-2.26882	1.22703
H	1.41447	-2.12760	0.28897
H	1.37278	-1.71922	2.02713
H	0.85853	-3.34643	1.49240

TS4_Fe_L9

Fe	-0.375001000	-0.154080000	-0.382310000
H	1.876839000	0.009540000	-1.975120000
P	0.103269000	1.915010000	-0.345820000
N	-1.394831000	2.778130000	-0.102020000
C	-2.486481000	1.917250000	0.095460000
C	-3.772601000	2.414020000	0.362290000
C	-4.821881000	1.517931000	0.593530000
C	-4.579881000	0.139761000	0.569840000
C	-3.285511000	-0.329810000	0.292800000
C	-2.184951000	0.528940000	0.028790000
N	-2.983891000	-1.703560000	0.290180000
P	-1.251091000	-1.993520000	0.239900000
C	-1.541240000	4.189040000	0.010080000
H	-1.917310000	4.499000000	1.008790000
H	-2.248560000	4.601430000	-0.740250000
H	-0.572930000	4.686600000	-0.145350000
C	-3.958771000	-2.651879000	0.713000000
H	-4.295791000	-2.493089000	1.762530000
H	-3.555782000	-3.672580000	0.643180000
H	-4.865661000	-2.617329000	0.077260000
H	1.145899000	-0.533010000	-0.254660000
Si	2.776079000	-2.315691000	-0.276700000
H	2.073739000	-2.976230000	0.869150000
H	3.790548000	-3.312501000	-0.785100000
C	4.372479000	0.605119000	2.250240000
C	5.045979000	1.470359000	1.363200000
C	3.686379000	-0.495771000	1.757860000
H	5.549199000	2.367849000	1.736130000
H	3.104129000	-1.117261000	2.448550000
C	5.059319000	1.167249000	0.002790000
C	3.670379000	-0.812981000	0.386520000
H	5.579509000	1.830859000	-0.697000000
C	4.405269000	0.034409000	-0.480110000
H	4.423569000	-0.181251000	-1.554540000
H	4.340489000	0.832379000	3.320340000
C	1.113779000	-0.752940000	-1.674060000
O	0.021919000	-0.772320000	-2.343590000
O	1.850859000	-2.019000000	-1.625900000
H	-5.830261000	1.894901000	0.801020000
C	-1.003871000	-2.845030000	1.888790000

H	-1.612042000	-3.760270000	2.018660000
H	0.062608000	-3.102450000	1.977910000
H	-1.249751000	-2.116430000	2.675400000
C	1.265329000	2.741240000	0.860910000
H	0.924349000	2.516180000	1.881460000
H	2.256469000	2.277030000	0.724900000
H	1.362760000	3.835130000	0.724670000
C	0.770889000	2.776570000	-1.870520000
H	0.742340000	3.879040000	-1.816100000
H	1.820199000	2.459690000	-1.998400000
H	0.194099000	2.425200000	-2.738500000
C	-1.180252000	-3.508320000	-0.833750000
H	-0.147172000	-3.888680000	-0.806450000
H	-1.871112000	-4.313160000	-0.529560000
H	-1.386072000	-3.187280000	-1.864520000
H	-5.402931000	-0.553609000	0.772150000
H	-3.966450000	3.491331000	0.398920000

TS4_Fe_L10

Fe	-0.79457	-0.14324	-0.23247
H	1.52982	0.04491	-1.72033
C	-2.93631	1.73585	0.06215
C	-4.26728	2.12060	0.13914
C	-5.26069	1.16420	0.44772
C	-4.88001	-0.17885	0.66709
C	-3.54444	-0.54425	0.58238
C	-2.52667	0.39148	0.26221
H	0.76050	-0.31669	0.09539
Si	2.59541	-1.98670	0.11744
H	1.99994	-2.42431	1.41931
H	3.53927	-3.08765	-0.32315
C	4.48937	1.29986	1.86468
C	5.14892	1.91377	0.76928
C	3.70580	0.17421	1.66072
H	5.73575	2.82754	0.91016
H	3.15178	-0.25349	2.50531
C	5.03580	1.33107	-0.49237
C	3.56341	-0.42457	0.39185
H	5.54441	1.79125	-1.34846
C	4.28163	0.17389	-0.68471
H	4.20941	-0.26311	-1.68762
H	4.55856	1.74070	2.86496
C	0.78806	-0.71949	-1.37244
O	-0.29321	-0.82950	-2.06783
O	1.54358	-1.94337	-1.18384
H	-6.31535	1.45695	0.49928
C	-2.98556	-1.89824	0.87857
H	-2.70462	-1.97594	1.95747
H	-3.68484	-2.73250	0.65056
C	-1.77294	2.65258	-0.12349
H	-2.00146	3.56616	-0.71353
H	-1.38620	2.97395	0.87263
H	-5.64981	-0.92653	0.91132
H	-4.55706	3.16919	-0.02640
O	-1.79108	-2.06530	0.12145
C	-1.07901	-3.21901	0.44307
H	-0.74344	-3.19147	1.50020
H	-1.70583	-4.12277	0.29026
H	-0.20159	-3.25680	-0.21693
O	-0.70894	1.93634	-0.76260
C	0.51994	2.59814	-0.66107
H	0.45178	3.61495	-1.09878
H	0.83461	2.67539	0.39739
H	1.27448	2.01544	-1.20497

TS4_Fe_L11

Fe	-0.945660000	0.366570000	0.776750000
H	1.691830000	0.574151000	1.390420000
C	-2.211659000	-2.088160000	-0.012700000
C	-3.087859000	-2.870970000	-0.752370000
C	-4.045089000	-2.254780000	-1.589700000
C	-4.136810000	-0.844610000	-1.623480000
C	-3.252050000	-0.078880000	-0.876540000
C	-2.223080000	-0.674380000	-0.105860000
H	0.331740000	0.561360000	-0.115330000
Si	2.461890000	2.091231000	-0.872000000
H	1.729650000	2.176641000	-2.181310000
H	3.385890000	3.286201000	-0.805450000
C	3.954361000	-1.653139000	-1.882860000
C	4.706221000	-2.040899000	-0.734700000
C	3.298660000	-0.435959000	-1.904900000
H	5.210641000	-3.011529000	-0.693340000
H	2.700380000	-0.173409000	-2.786960000
C	4.749930000	-1.166189000	0.355880000
C	3.339440000	0.476831000	-0.819950000
H	5.304771000	-1.454799000	1.257260000
C	4.096800000	0.062221000	0.330730000
H	4.151370000	0.716481000	1.208710000
H	3.871961000	-2.327829000	-2.742050000
C	0.868770000	1.287211000	1.130390000
O	0.052330000	1.598970000	2.072300000
O	1.465110000	2.402381000	0.448270000
H	-4.723569000	-2.866410000	-2.195300000
C	-3.366070000	1.385080000	-0.575870000
H	-3.822170000	1.990630000	-1.393870000
H	-4.012740000	1.500780000	0.316260000
C	-1.327269000	-2.545150000	1.107150000
H	-1.916649000	-2.508770000	2.045450000
H	-0.945399000	-3.586570000	0.996840000
N	-2.046010000	1.948840000	-0.202420000
N	-0.192489000	-1.608890000	1.296210000
C	-1.329150000	2.318930000	-1.413440000
H	-1.890880000	3.099590000	-1.976310000
H	-0.340000000	2.709150000	-1.149550000
H	-1.205630000	1.432660000	-2.050200000
C	-2.190730000	3.111210000	0.654730000
H	-2.686630000	2.811910000	1.588960000
H	-1.193430000	3.490590000	0.915410000
H	-2.785340000	3.911210000	0.154490000
C	0.297661000	-1.664420000	2.661320000
H	0.574481000	-2.704410000	2.952600000
H	1.186350000	-1.024919000	2.760230000
H	-0.478730000	-1.281750000	3.340430000
C	0.870311000	-1.980279000	0.365600000
H	0.468591000	-1.990790000	-0.656470000
H	1.700750000	-1.266549000	0.405570000
H	1.266011000	-2.990229000	0.609030000
H	-4.931460000	-0.366610000	-2.215010000
H	-3.065439000	-3.967300000	-0.669630000

TS4_Ni_L1

Ni	0.086029000	0.341803000	-0.444758000
H	0.634485000	-1.713863000	-2.147245000
P	2.213615000	0.055655000	-0.116960000
N	2.659555000	1.771961000	-0.047429000
C	1.646526000	2.663486000	0.066448000
N	1.898644000	3.948567000	0.302240000
C	0.826262000	4.712298000	0.460524000
N	-0.437005000	4.313786000	0.477315000
C	-0.627756000	3.018656000	0.229774000
N	0.391589000	2.176344000	-0.051819000
N	-1.872046000	2.490701000	0.267276000

P	-2.017052000	0.730302000	0.088928000
N	-3.383771000	0.544905000	-0.856755000
C	-4.421438000	-0.242893000	-0.213033000
H	-4.341432000	-1.309319000	-0.504392000
H	-5.413196000	0.111547000	-0.536407000
C	-4.214452000	-0.061674000	1.282964000
H	-4.771174000	0.815524000	1.666304000
H	-4.557212000	-0.939806000	1.854031000
N	-2.779308000	0.104929000	1.448894000
N	3.261853000	-0.620557000	-1.221599000
C	4.256818000	-1.515577000	-0.649447000
H	5.245979000	-1.306935000	-1.090606000
H	4.010194000	-2.567862000	-0.881732000
C	4.255196000	-1.262470000	0.856210000
H	4.430512000	-2.188889000	1.425881000
H	5.050459000	-0.549722000	1.149581000
N	2.936331000	-0.734604000	1.171353000
H	-0.122201000	-1.145458000	-0.510147000
Si	-1.628177000	-3.297950000	-1.614140000
H	-3.097555000	-3.482449000	-1.582307000
H	-0.935465000	-4.175570000	-2.591458000
C	-0.885500000	-3.047582000	2.464079000
C	0.467673000	-3.367903000	2.551195000
C	-1.526543000	-3.074044000	1.229199000
H	0.966181000	-3.359734000	3.523765000
H	-2.587781000	-2.807160000	1.171260000
C	1.177734000	-3.724278000	1.406563000
C	-0.831101000	-3.433724000	0.065818000
H	2.234605000	-3.993191000	1.476999000
C	0.527689000	-3.768469000	0.177266000
H	1.087529000	-4.073220000	-0.714251000
H	-1.443403000	-2.791528000	3.367822000
C	-0.242981000	-1.026883000	-2.059492000
O	-0.155840000	0.140284000	-2.525262000
O	-1.432286000	-1.678744000	-2.123109000
H	1.005203000	5.782922000	0.613279000
C	3.258542000	-0.332606000	-2.636005000
H	3.255966000	-1.263234000	-3.226028000
H	2.357413000	0.238116000	-2.903672000
H	4.146500000	0.253615000	-2.929314000
C	2.610971000	-0.419014000	2.537289000
H	2.723623000	-1.311640000	3.171431000
H	3.255061000	0.379812000	2.949932000
H	1.562787000	-0.090786000	2.603346000
C	-3.422288000	0.715778000	-2.291546000
H	-3.507860000	-0.255219000	-2.805504000
H	-4.278153000	1.347629000	-2.575486000
H	-2.496107000	1.187940000	-2.643761000
C	-2.228445000	0.258021000	2.769052000
H	-2.554621000	1.195224000	3.257160000
H	-2.535243000	-0.584048000	3.408028000
H	-1.129109000	0.256442000	2.717032000
C	-3.009985000	3.338531000	0.578946000
H	-3.926743000	2.806604000	0.298948000
H	-3.040198000	3.591308000	1.649329000
H	-2.952862000	4.273213000	0.008208000
C	4.039273000	2.210812000	0.060977000
H	4.343333000	2.341725000	1.110727000
H	4.686502000	1.462361000	-0.414731000
H	4.168174000	3.169786000	-0.454410000

TS4_Ni_L3

Ni	-0.06010	0.43793	-0.72524
H	0.99411	-1.43590	-2.39036
P	2.01749	0.91961	-0.30991
C	1.91755	2.76285	-0.63206
C	0.53851	3.23786	-0.35563
N	0.32550	4.48475	0.02994

C	-0.94137	4.80785	0.27297
N	-1.98311	3.99945	0.14143
C	-1.71726	2.76391	-0.26317
N	-0.45605	2.33447	-0.49602
C	-2.80120	1.77566	-0.42558
P	-2.16873	0.06240	0.08459
N	-3.38440	-0.99126	-0.39674
C	-4.17955	-1.51546	0.70306
H	-3.89391	-2.55838	0.93856
H	-5.24588	-1.52414	0.42356
C	-3.92050	-0.59064	1.87855
H	-4.61215	0.27744	1.85658
H	-4.05351	-1.09441	2.84810
N	-2.53475	-0.15703	1.73324
N	3.39954	0.40928	-1.13514
C	4.42828	-0.09231	-0.23513
H	5.42751	0.15234	-0.62925
H	4.36025	-1.19415	-0.14183
C	4.16653	0.57845	1.09938
H	4.56594	-0.00752	1.94272
H	4.63515	1.58371	1.13636
N	2.71711	0.66554	1.20694
H	0.37071	-1.00106	-0.66782
Si	-0.41241	-3.67611	-1.16160
H	-1.71900	-4.31048	-0.86906
H	0.43902	-4.46416	-2.08904
C	0.62143	-2.28480	2.60939
C	2.01228	-2.37541	2.60733
C	-0.10094	-2.71299	1.50089
H	2.58066	-2.05843	3.48590
H	-1.19336	-2.63088	1.51634
C	2.67869	-2.88638	1.49691
C	0.55163	-3.22532	0.36884
H	3.76810	-2.97547	1.50483
C	1.95223	-3.30424	0.38438
H	2.48448	-3.71278	-0.48229
H	0.09846	-1.89461	3.48592
C	-0.05965	-1.15481	-2.15779
O	-0.55644	-0.12705	-2.69048
O	-0.85659	-2.22320	-1.93705
H	-1.13921	5.83006	0.61380
C	3.37659	-0.09929	-2.48539
H	3.32660	-1.20480	-2.50906
H	2.51268	0.30252	-3.03537
H	4.28596	0.20668	-3.02390
C	2.17135	1.29436	2.38117
H	2.50516	0.76046	3.28338
H	2.47696	2.35476	2.48029
H	1.07338	1.24839	2.35654
C	-3.71263	-1.35699	-1.75276
H	-3.63677	-2.44624	-1.90446
H	-4.74122	-1.04449	-1.99994
H	-3.01743	-0.88392	-2.45702
C	-2.06250	0.81227	2.68993
H	-2.63209	1.76314	2.65270
H	-2.15030	0.40953	3.70936
H	-1.00059	1.03323	2.51050
H	-3.73060	2.11014	0.05529
H	2.66969	3.37141	-0.10886
H	2.11221	2.85708	-1.71583
H	-2.99523	1.63249	-1.50467

TS4_Ni_L4

Ni	0.06739	0.37746	-0.40710
H	0.67361	-1.66255	-2.10801
P	2.19333	0.11022	-0.12427
N	2.61400	1.81483	-0.09463
C	1.59539	2.71860	0.08938

C	1.82626	4.07214	0.35103
C	0.72817	4.89128	0.57095
C	-0.56142	4.38235	0.56327
C	-0.73406	3.01965	0.29418
N	0.33328	2.23046	0.02430
N	-1.96817	2.41827	0.30018
P	-2.04648	0.67923	0.08849
N	-3.38788	0.46850	-0.89794
C	-4.36206	-0.44920	-0.33661
H	-4.16369	-1.48971	-0.66446
H	-5.37108	-0.18095	-0.68812
C	-4.23011	-0.31245	1.17227
H	-4.87511	0.49916	1.56312
H	-4.52139	-1.23791	1.69579
N	-2.82303	-0.03426	1.40244
N	3.22025	-0.57351	-1.24975
C	4.23708	-1.45712	-0.70272
H	5.21773	-1.22022	-1.15034
H	4.01609	-2.51057	-0.95378
C	4.24984	-1.23161	0.80876
H	4.41350	-2.17242	1.35922
H	5.06205	-0.54098	1.11065
N	2.94689	-0.68534	1.14794
H	-0.09587	-1.11557	-0.46838
Si	-1.50892	-3.33129	-1.58655
H	-2.96779	-3.58497	-1.54368
H	-0.78691	-4.16954	-2.57764
C	-0.73684	-3.08004	2.48575
C	0.62640	-3.35866	2.55908
C	-1.38788	-3.12055	1.25671
H	1.13330	-3.33841	3.52714
H	-2.45684	-2.88524	1.20882
C	1.33667	-3.68707	1.40656
C	-0.69211	-3.45227	0.08522
H	2.40266	-3.92113	1.46606
C	0.67684	-3.74564	0.18299
H	1.23751	-4.02707	-0.71540
H	-1.29359	-2.84390	3.39561
C	-0.22577	-1.00437	-2.01753
O	-0.17573	0.16380	-2.48624
O	-1.39184	-1.70153	-2.08282
H	0.88355	5.95300	0.77319
C	3.21062	-0.25736	-2.65732
H	3.20518	-1.17555	-3.26663
H	2.30829	0.31814	-2.90902
H	4.09822	0.33410	-2.94373
C	2.66447	-0.35793	2.51977
H	2.79270	-1.24598	3.15785
H	3.32543	0.44119	2.90575
H	1.62092	-0.02372	2.61673
C	-3.40070	0.72056	-2.31996
H	-3.32506	-0.21379	-2.89894
H	-4.33027	1.24091	-2.59918
H	-2.54541	1.34838	-2.60104
C	-2.34177	0.12995	2.74707
H	-2.76366	1.02655	3.23909
H	-2.60372	-0.74870	3.35662
H	-1.24531	0.22137	2.74166
C	-3.15567	3.19884	0.57860
H	-4.03547	2.55664	0.46614
H	-3.14325	3.60949	1.60118
H	-3.26239	4.02877	-0.13650
C	3.99334	2.25110	-0.07262
H	4.30349	2.60252	0.92562
H	4.63723	1.41266	-0.36321
H	4.16146	3.05932	-0.80019
H	-1.41638	5.02349	0.76663
H	2.83854	4.46862	0.39429

TS4_Ni_L5

Ni	0.329670000	0.070941000	0.014520000
H	2.324509000	1.105362000	-2.756070000
P	0.076489000	2.201000000	0.074440000
N	-1.707951000	2.231379000	-0.045180000
C	-2.347661000	1.038769000	-0.067520000
N	-3.678491000	0.971998000	-0.082290000
C	-4.170720000	-0.255382000	-0.074290000
N	-3.511829000	-1.395502000	-0.008280000
C	-2.181039000	-1.266291000	0.008300000
N	-1.573800000	-0.065851000	-0.062730000
N	-1.386219000	-2.353650000	0.111100000
P	0.379381000	-2.070799000	0.243370000
N	1.028742000	-3.287999000	-0.689890000
C	1.785833000	-4.279578000	0.057320000
H	2.873272000	-4.117398000	-0.063510000
H	1.561873000	-5.285869000	-0.334380000
C	1.360582000	-4.140259000	1.514930000
H	0.542763000	-4.842629000	1.769350000
H	2.193403000	-4.349648000	2.205060000
N	0.937272000	-2.757059000	1.667360000
N	0.569428000	3.359741000	-1.020320000
C	1.119207000	4.568221000	-0.424190000
H	0.691367000	5.455381000	-0.921580000
H	2.214027000	4.609092000	-0.570060000
C	0.754667000	4.533501000	1.058170000
H	1.565677000	4.936071000	1.686030000
H	-0.147513000	5.139240000	1.270030000
N	0.538998000	3.132661000	1.383920000
H	1.763640000	0.179142000	0.250490000
Si	4.392310000	-0.605627000	-1.888850000
H	4.985921000	-1.953146000	-2.022920000
H	4.961000000	0.413733000	-2.806800000
C	3.931050000	-0.446327000	2.214410000
C	3.793219000	0.918513000	2.457480000
C	4.184410000	-0.896807000	0.922850000
H	3.600069000	1.272773000	3.472880000
H	4.280331000	-1.972617000	0.739500000
C	3.920049000	1.833783000	1.413890000
C	4.307820000	0.007633000	-0.140850000
H	3.820228000	2.904673000	1.609150000
C	4.183629000	1.379593000	0.125550000
H	4.286909000	2.106323000	-0.688390000
H	3.844931000	-1.160667000	3.036450000
C	1.876390000	0.094202000	-2.591530000
O	0.683500000	-0.080389000	-2.607190000
O	2.756680000	-0.860368000	-2.392880000
C	0.381198000	3.280541000	-2.446860000
H	1.323638000	3.487271000	-2.981200000
H	0.038909000	2.275130000	-2.730830000
H	-0.367713000	4.013310000	-2.795240000
C	0.251718000	2.757231000	2.743410000
H	1.040598000	3.130691000	3.414310000
H	-0.716662000	3.157210000	3.096930000
H	0.227639000	1.661171000	2.829780000
C	0.875912000	-3.422849000	-2.117540000
H	1.854712000	-3.475478000	-2.619230000
H	0.307383000	-4.336239000	-2.364160000
H	0.343772000	-2.553959000	-2.525010000
C	0.504331000	-2.288019000	2.958000000
H	-0.431038000	-2.774220000	3.292930000
H	1.280481000	-2.484339000	3.712580000
H	0.338431000	-1.201109000	2.922080000
C	-1.973018000	-3.679781000	0.195280000
H	-1.196547000	-4.421620000	-0.029160000
H	-2.384908000	-3.875971000	1.196640000
H	-2.784738000	-3.785371000	-0.534830000
C	-2.451992000	3.477949000	-0.029150000
H	-2.644612000	3.818209000	0.999940000
H	-1.873433000	4.247389000	-0.558540000

H	-3.414702000	3.346898000	-0.535880000
C	-5.699490000	-0.322973000	-0.111830000
F	-6.157000000	0.365717000	-1.150040000
F	-6.136859000	-1.563983000	-0.201850000
F	-6.187900000	0.220117000	1.000070000

TS4_Ni_L6

Ni	-0.390009000	-0.117366000	-0.004035000
H	1.870570000	-0.096221000	-1.490927000
P	0.270488000	1.952862000	0.179017000
N	-1.259774000	2.732199000	-0.132344000
C	-2.353697000	1.928561000	-0.184797000
N	-3.560244000	2.462763000	-0.334229000
C	-4.563509000	1.594097000	-0.328397000
N	-4.476803000	0.285818000	-0.145094000
C	-3.241050000	-0.188139000	0.001072000
N	-2.146994000	0.600859000	-0.059317000
N	-3.046297000	-1.505501000	0.238764000
P	-1.396985000	-2.051556000	0.499302000
H	1.042361000	-0.554646000	0.124545000
Si	2.850243000	-2.523898000	-0.274097000
H	2.254616000	-3.091856000	0.968281000
H	3.631957000	-3.518214000	-1.039705000
C	4.285208000	0.888637000	1.625409000
C	5.062838000	1.489305000	0.637039000
C	3.648227000	-0.323458000	1.367223000
H	5.568290000	2.436458000	0.840249000
H	3.037950000	-0.786634000	2.150322000
C	5.215959000	0.871011000	-0.603722000
C	3.788476000	-0.959938000	0.125053000
H	5.838282000	1.332636000	-1.373402000
C	4.589613000	-0.347119000	-0.851592000
H	4.727318000	-0.829543000	-1.825754000
H	4.190032000	1.358446000	2.607902000
C	1.055105000	-0.849110000	-1.405768000
O	-0.031708000	-0.645404000	-2.009104000
O	1.536848000	-2.110351000	-1.283633000
H	-5.568634000	2.004461000	-0.478675000
C	-4.212036000	-2.375491000	0.330679000
H	-4.799145000	-2.328653000	-0.595488000
H	-3.886501000	-3.409300000	0.489150000
H	-4.860933000	-2.073842000	1.164162000
C	-1.439182000	4.174690000	-0.228717000
H	-2.034604000	4.554480000	0.613111000
H	-0.459091000	4.665296000	-0.223631000
H	-1.958446000	4.433262000	-1.160298000
C	-1.459512000	-2.831279000	2.143712000
H	-2.188030000	-3.653951000	2.185726000
H	-0.461144000	-3.239774000	2.360694000
H	-1.701995000	-2.080595000	2.907136000
C	0.879543000	2.718677000	1.709805000
H	0.206321000	2.481561000	2.543602000
H	1.872811000	2.293967000	1.921237000
H	0.977720000	3.809358000	1.607043000
C	1.398277000	2.578650000	-1.099265000
H	1.480281000	3.674277000	-1.079552000
H	2.398132000	2.155326000	-0.904725000
H	1.053510000	2.246208000	-2.088310000
C	-1.225956000	-3.448970000	-0.645026000
H	-1.220973000	-3.068044000	-1.674665000
H	-0.254714000	-3.927575000	-0.455709000
H	-2.021783000	-4.193332000	-0.505568000

TS4_Ni_L7

Ni	0.14476	-0.25773	-0.41723
H	-1.41087	0.94531	-2.27881
P	-1.58319	-1.47170	-0.07669
N	-0.84818	-3.03597	-0.16294
C	0.51352	-3.05686	-0.08383
N	1.16439	-4.20302	0.04655
C	2.47987	-4.08924	0.18628
N	3.16780	-2.96181	0.28546
C	2.46028	-1.84664	0.14477
N	1.13624	-1.86078	-0.12034
N	3.05017	-0.62912	0.28212
P	2.00591	0.75891	0.24118
O	2.87103	1.77231	-0.69742
C	3.11890	3.01020	-0.03681
H	2.29895	3.71217	-0.26425
H	4.05734	3.42471	-0.42506
C	3.18845	2.67227	1.44586
H	4.19723	2.35311	1.75013
H	2.86985	3.50151	2.08975
O	2.27291	1.58548	1.62885
O	-2.80329	-1.56742	-1.15344
C	-4.08479	-1.40790	-0.53867
H	-4.80028	-2.05161	-1.06548
H	-4.39919	-0.35773	-0.64270
C	-3.90320	-1.80217	0.92252
H	-4.54891	-1.22646	1.59773
H	-4.08387	-2.87529	1.08947
O	-2.54240	-1.49822	1.24031
H	-0.71989	0.96619	-0.53456
Si	-0.96953	3.62578	-1.49534
H	-0.05298	4.77461	-1.32166
H	-1.98463	3.81505	-2.56103
C	-1.52324	2.35416	2.40530
C	-2.79734	1.79012	2.37413
C	-1.02140	2.98310	1.27098
H	-3.19589	1.29653	3.26368
H	-0.01350	3.41321	1.30496
C	-3.56579	1.86216	1.21411
C	-1.77011	3.04919	0.08602
H	-4.57711	1.44632	1.20085
C	-3.05426	2.48506	0.07810
H	-3.66525	2.53514	-0.83036
H	-0.92148	2.30211	3.31507
C	-0.32460	1.06007	-2.03946
O	0.48400	0.18413	-2.44624
O	0.07611	2.34828	-1.95159
H	3.05096	-5.02253	0.24362
C	4.47387	-0.51429	0.57141
H	4.86974	0.37384	0.06410
H	4.64907	-0.43712	1.65409
H	4.99248	-1.40080	0.19268
C	-1.61724	-4.27163	-0.10970
H	-1.84531	-4.55340	0.92861
H	-2.54767	-4.13744	-0.67515
H	-1.03674	-5.07732	-0.57141

TS4_Ni_L8

Ni	-0.514233000	-0.035373000	-0.287854000
H	1.308590000	0.352350000	-2.267168000
C	-3.030273000	1.135658000	-0.262737000
N	-4.332147000	1.227011000	-0.077749000
C	-4.919288000	0.114629000	0.368711000
N	-4.309866000	-1.038294000	0.649063000
C	-3.007522000	-1.050520000	0.440196000
N	-2.355478000	0.016093000	-0.015905000
H	0.980642000	0.004005000	-0.440488000

Si	3.353952000	-1.356581000	-1.403507000
H	3.843284000	-2.708405000	-1.055882000
H	3.977363000	-0.771400000	-2.616817000
C	3.251048000	0.330619000	2.397187000
C	3.375568000	1.694920000	2.136737000
C	3.301603000	-0.581828000	1.347096000
H	3.351399000	2.412022000	2.960815000
H	3.224989000	-1.653236000	1.566251000
C	3.562613000	2.141462000	0.829839000
C	3.467824000	-0.152799000	0.021415000
H	3.690830000	3.208056000	0.628414000
C	3.611357000	1.222394000	-0.216080000
H	3.775446000	1.583097000	-1.238292000
H	3.135759000	-0.021499000	3.425224000
C	0.815512000	-0.573596000	-1.878716000
O	-0.367021000	-0.824398000	-2.232885000
O	1.691585000	-1.593737000	-1.716779000
H	-6.001889000	0.154991000	0.527134000
C	-2.163942000	2.225289000	-0.807145000
H	-2.243655000	2.176357000	-1.906927000
H	-2.522273000	3.223650000	-0.500319000
C	-2.115904000	-2.231973000	0.637146000
H	-2.478890000	-2.882455000	1.453053000
H	-2.157245000	-2.822874000	-0.294770000
N	-0.720630000	-1.789263000	0.850866000
N	-0.750512000	1.995437000	-0.422417000
C	-0.533806000	-1.382971000	2.252078000
H	-0.667934000	-2.247568000	2.927775000
H	0.477614000	-0.977806000	2.383231000
H	-1.261733000	-0.608982000	2.530918000
C	0.187800000	-2.897678000	0.538297000
H	0.135822000	-3.134996000	-0.530302000
H	1.214224000	-2.600254000	0.782469000
H	-0.073991000	-3.787942000	1.137358000
C	0.137537000	2.649557000	-1.388962000
H	-0.090925000	3.727330000	-1.459446000
H	1.177824000	2.531834000	-1.059814000
H	0.009250000	2.192221000	-2.379035000
C	-0.501060000	2.536460000	0.924617000
H	-1.204460000	2.099650000	1.645947000
H	0.522872000	2.290853000	1.233831000
H	-0.630188000	3.634036000	0.928099000

TS4_Ni_L9

Ni	-0.415855000	-0.112622000	-0.131400000
H	1.923952000	0.061870000	-1.584990000
P	0.049418000	1.985112000	0.047836000
N	-1.446734000	2.761311000	-0.249830000
C	-2.539388000	1.879435000	-0.175919000
C	-3.863343000	2.341356000	-0.187011000
C	-4.896169000	1.416107000	-0.071469000
C	-4.638941000	0.057008000	0.076733000
C	-3.307110000	-0.386256000	0.089258000
C	-2.240012000	0.512194000	-0.066471000
N	-2.981073000	-1.736663000	0.272884000
P	-1.296666000	-2.045238000	0.402710000
H	1.036287000	-0.458152000	0.043802000
Si	2.833782000	-2.343333000	-0.235612000
H	2.130967000	-2.830441000	0.987118000
H	3.683747000	-3.410042000	-0.822132000
C	4.328379000	1.041067000	1.687193000
C	5.296845000	1.502602000	0.797768000
C	3.605317000	-0.111783000	1.390015000
H	5.863798000	2.407982000	1.028820000
H	2.836266000	-0.460164000	2.088257000
C	5.545396000	0.806934000	-0.383835000
C	3.841060000	-0.826850000	0.207247000
H	6.305946000	1.165629000	-1.081577000
C	4.825669000	-0.350029000	-0.670459000
H	5.032194000	-0.892416000	-1.599833000
H	4.138741000	1.581106000	2.618631000
C	1.126209000	-0.710532000	-1.492232000
O	0.051923000	-0.562876000	-2.132546000
O	1.700761000	-1.973998000	-1.420346000
H	-5.931873000	1.765872000	-0.084904000
C	-4.006809000	-2.715131000	0.510955000
H	-4.710809000	-2.775868000	-0.336357000
H	-3.562260000	-3.710539000	0.637000000
H	-4.590402000	-2.489617000	1.422296000
C	-1.641846000	4.185202000	-0.237903000
H	-2.156662000	4.529708000	0.678441000
H	-0.674157000	4.700732000	-0.295247000
H	-2.237969000	4.513019000	-1.105453000
C	-1.086650000	-2.780488000	2.068773000
H	-1.703910000	-3.680997000	2.208939000
H	-0.029223000	-3.053295000	2.202301000
H	-1.353819000	-2.029687000	2.824886000
C	0.659160000	2.687818000	1.625346000
H	-0.022696000	2.391290000	2.433973000
H	1.658305000	2.276021000	1.832046000
H	0.729480000	3.785823000	1.585097000
C	1.218701000	2.698446000	-1.159335000
H	1.258894000	3.796024000	-1.106519000
H	2.225074000	2.303938000	-0.944367000
H	0.920576000	2.384435000	-2.169344000
C	-1.015542000	-3.476574000	-0.693285000
H	-1.144961000	-3.143781000	-1.731717000
H	0.021732000	-3.819900000	-0.576385000
H	-1.698064000	-4.308596000	-0.467195000
H	-5.469270000	-0.643081000	0.184243000
H	-4.092526000	3.404391000	-0.280702000

TS4_Ni_L11

Ni	-0.684907000	-0.142607000	-0.153083000
H	1.643416000	-0.032643000	-1.629896000
C	-2.778041000	1.719041000	-0.363780000
C	-4.102252000	2.146432000	-0.272155000
C	-5.104953000	1.206052000	-0.016063000
C	-4.798239000	-0.148466000	0.146933000
C	-3.469490000	-0.561971000	0.053078000
C	-2.472452000	0.371992000	-0.199428000
H	0.783134000	-0.381571000	0.064293000
Si	2.779321000	-2.151016000	-0.058415000
H	2.202715000	-2.583164000	1.248860000
H	3.731565000	-3.164969000	-0.577850000
C	3.951194000	1.491927000	1.585693000
C	4.757150000	2.046398000	0.593481000
C	3.381406000	0.235494000	1.393172000
H	5.202861000	3.033593000	0.739323000
H	2.738489000	-0.188310000	2.172647000
C	4.995528000	1.341058000	-0.584787000
C	3.608730000	-0.491527000	0.216263000
H	5.627544000	1.773688000	-1.364079000
C	4.429624000	0.081631000	-0.765693000
H	4.625370000	-0.464921000	-1.694977000
H	3.765031000	2.041963000	2.511659000
C	0.895813000	-0.832997000	-1.423291000
O	-0.181346000	-0.831788000	-2.076321000
O	1.564025000	-2.038222000	-1.210921000
H	-6.143938000	1.535988000	0.064413000
C	-1.565477000	2.540768000	-0.665570000
H	-1.390577000	2.559553000	-1.754800000
H	-1.628885000	3.594245000	-0.325968000
C	-2.927409000	-1.951524000	0.150435000
H	-3.522570000	-2.629057000	0.796099000
H	-2.894985000	-2.404300000	-0.855139000

N	-1.522563000	-1.895197000	0.635524000
N	-0.380264000	1.879346000	-0.052622000
C	-1.506983000	-1.711878000	2.087993000
H	-1.947027000	-2.593331000	2.596210000
H	-0.472268000	-1.582206000	2.433635000
H	-2.088414000	-0.821510000	2.359592000
C	-0.858372000	-3.149415000	0.292670000
H	-0.737764000	-3.218130000	-0.795199000
H	0.132213000	-3.184235000	0.759071000
H	-1.449541000	-4.010006000	0.660761000
C	0.839415000	2.343201000	-0.709125000
H	0.908786000	3.446395000	-0.662228000
H	1.719039000	1.916001000	-0.209591000
H	0.831571000	2.033275000	-1.762535000
C	-0.316918000	2.205995000	1.375322000
H	-1.261073000	1.931060000	1.861876000
H	0.505487000	1.651688000	1.845221000
H	-0.145292000	3.291865000	1.513580000
H	-5.597218000	-0.868759000	0.349625000
H	-4.362058000	3.202801000	-0.393739000

4.5.5. TS5

TS5_Co_L2

Co	-1.09784	0.03760	-0.11649
H	-0.36677	-1.72104	-2.75961
P	-0.45547	2.10297	-0.42216
O	-1.97391	2.79412	-1.03810
C	-3.02149	2.01323	-1.02679
N	-4.17650	2.48682	-1.46809
C	-5.18522	1.62829	-1.44557
N	-5.13606	0.36565	-1.04201
C	-3.95323	-0.03963	-0.61298
N	-2.85557	0.75152	-0.56184
O	-3.81902	-1.28068	-0.21062
P	-2.22149	-1.71285	0.40392
N	-2.09033	-3.34190	0.06985
C	-2.41472	-4.20346	1.19459
H	-1.47870	-4.57767	1.65401
H	-2.99784	-5.07788	0.86149
C	-3.19235	-3.34054	2.16772
H	-4.27289	-3.33541	1.91440
H	-3.09169	-3.69103	3.20681
N	-2.62962	-2.00521	2.02989
N	0.69652	2.92866	-1.30188
C	1.29672	4.05094	-0.59723
H	1.40228	4.91434	-1.27517
H	2.30746	3.76499	-0.24866
C	0.37777	4.35835	0.56795
H	0.92704	4.77248	1.42872
H	-0.40208	5.09284	0.27765
N	-0.23387	3.09141	0.93324
H	-0.74090	0.31524	1.51429
Si	0.53029	-0.41488	2.02510
H	0.27971	-0.39877	3.50391
H	0.63816	-1.83143	1.58716
C	3.78659	1.54051	0.28465
C	4.34615	2.22325	1.36376
C	2.65000	0.75747	0.45827
H	5.24253	2.83278	1.22262
H	2.20819	0.22511	-0.39080
C	3.76370	2.11678	2.62405
C	2.05957	0.62911	1.72510
H	4.19688	2.64654	3.47661
C	2.63146	1.32276	2.80041
H	2.18876	1.23811	3.79824
H	4.24829	1.60764	-0.70430
C	0.27579	-0.88383	-2.37458
O	1.53330	-0.98921	-3.02642
O	0.40934	-0.89304	-1.03621
C	1.40995	2.43328	-2.45360
H	2.44940	2.17194	-2.18905
H	0.93504	1.53563	-2.86034
H	1.43516	3.19965	-3.24476
C	-1.21446	3.15981	1.98562
H	-1.68911	2.18155	2.13942
H	-0.73087	3.45180	2.93144
H	-2.00962	3.89681	1.75951
C	-1.24302	-3.89338	-0.95992
H	-0.31794	-4.31264	-0.52631
H	-1.76742	-4.69357	-1.50557
H	-0.95388	-3.11549	-1.67431
C	-3.28160	-0.97323	2.80009
H	-4.36233	-0.90374	2.56954
H	-3.16900	-1.18094	3.87477
H	-2.81967	0.00295	2.59696
H	-6.15596	1.99667	-1.79629
Si	2.52393	-2.28809	-2.68094
H	1.67060	-3.50563	-2.52078
H	3.42846	-2.40745	-3.85798
C	3.60185	-2.09704	-1.16076
C	3.16740	-2.52866	0.09965
C	4.86618	-1.50024	-1.25197
C	3.95753	-2.35600	1.23158
C	5.66182	-1.31894	-0.12236
C	5.20490	-1.74433	1.12209
H	2.17767	-2.98576	0.19687
H	5.23755	-1.16659	-2.22768
H	3.59350	-2.68737	2.20817
H	6.64095	-0.84139	-0.21271
H	5.82142	-1.59568	2.01241
H	-0.19301	0.04192	-2.78233

TS5_Co_L3

Co	-1.15264	-0.00647	-0.20890
H	-0.26730	-1.65105	-2.80452
P	-0.69103	2.12942	-0.48372
C	-2.22106	2.70126	-1.38467
C	-3.33865	1.76482	-1.11478
N	-4.58787	2.17444	-1.27535
C	-5.53376	1.26282	-1.09294
N	-5.30723	-0.02295	-0.85443
C	-4.04059	-0.37509	-0.70130
N	-3.00058	0.50395	-0.72786
C	-3.69706	-1.80604	-0.50290
P	-2.05265	-1.90115	0.36071
N	-1.55161	-3.52012	0.19248
C	-1.64665	-4.26838	1.43307
H	-0.65985	-4.29837	1.93875
H	-1.95220	-5.31072	1.23863
C	-2.65667	-3.53369	2.29067
H	-3.69388	-3.83007	2.01816
H	-2.52481	-3.74988	3.36326
N	-2.42116	-2.12581	2.03022
N	0.55763	3.03415	-1.20099
C	1.09278	4.06922	-0.33493
H	1.33599	4.96975	-0.92558
H	2.02448	3.72519	0.15443
C	0.01352	4.35051	0.69086
H	0.42950	4.76207	1.62531
H	-0.72005	5.08992	0.29904
N	-0.62134	3.07269	0.95237
H	-0.86737	0.29672	1.43141
Si	0.46149	-0.29736	1.97099
H	0.16080	-0.27360	3.44120
H	0.70190	-1.70974	1.57525
C	3.77664	1.69243	0.38850
C	4.28753	2.36334	1.49814
C	2.61994	0.92680	0.49807
H	5.20010	2.95824	1.40749
H	2.21300	0.40398	-0.37375
C	3.63284	2.26835	2.72353
C	1.95936	0.80525	1.72965
H	4.02466	2.79271	3.59919
C	2.48000	1.49272	2.83510
H	1.98154	1.41342	3.80664
H	4.29079	1.75752	-0.57402
C	0.34221	-0.78064	-2.42856
O	1.60563	-0.84120	-3.08091
O	0.46720	-0.76529	-1.09428
C	1.39907	2.57521	-2.27692
H	2.38978	2.24848	-1.91540
H	0.94695	1.72355	-2.79849

H	1.55305	3.38305	-3.01206
C	-1.75373	3.11174	1.83812
H	-2.18113	2.10637	1.96144
H	-1.44095	3.46473	2.83362
H	-2.55665	3.78925	1.47733
C	-0.49074	-3.90396	-0.70782
H	0.48511	-3.95568	-0.19006
H	-0.69706	-4.89253	-1.14904
H	-0.39218	-3.17356	-1.51913
C	-3.31479	-1.20483	2.68291
H	-4.37886	-1.37284	2.41410
H	-3.22499	-1.30447	3.77570
H	-3.05014	-0.17014	2.42078
H	-6.57649	1.59044	-1.16369
H	-1.95344	2.60891	-2.45377
H	-3.50185	-2.25015	-1.49667
H	-2.51442	3.74750	-1.21461
H	-4.52741	-2.36621	-0.04949
Si	2.67036	-2.07612	-2.73317
H	1.89739	-3.34846	-2.58990
H	3.59016	-2.13533	-3.90340
C	3.73373	-1.83945	-1.20747
C	3.29760	-2.25216	0.05896
C	4.99672	-1.24063	-1.30359
C	4.08486	-2.06296	1.18999
C	5.79035	-1.04347	-0.17488
C	5.33224	-1.45314	1.07442
H	2.30847	-2.70844	0.16086
H	5.36987	-0.92045	-2.28298
H	3.71764	-2.37969	2.17008
H	6.76921	-0.56633	-0.27034
H	5.94672	-1.29195	1.96398
H	-0.15690	0.12448	-2.85476

TS5_Co_L4

Co	-1.01087	0.06309	0.07435
H	-0.50292	-1.40755	-2.71606
P	-0.47435	2.15070	-0.10271
N	-1.92735	2.77182	-0.88166
C	-2.98320	1.90579	-1.01831
C	-4.20455	2.31406	-1.57289
C	-5.22759	1.38569	-1.68052
C	-5.03918	0.08335	-1.24335
C	-3.79748	-0.26430	-0.69722
N	-2.78600	0.63543	-0.57777
N	-3.54090	-1.53585	-0.23960
P	-1.99284	-1.77862	0.54244
N	-1.60974	-3.40333	0.21954
C	-1.52673	-4.23011	1.40738
H	-0.48046	-4.28015	1.77252
H	-1.84462	-5.26242	1.18037
C	-2.42409	-3.57483	2.44094
H	-3.47282	-3.93042	2.34728
H	-2.09770	-3.80473	3.46901
N	-2.31942	-2.15537	2.18259
N	0.77023	3.02790	-0.84387
C	1.47871	3.94297	0.03029
H	1.72645	4.86939	-0.51863
H	2.43140	3.49678	0.37144
C	0.55215	4.22154	1.20289
H	1.12061	4.39934	2.13161
H	-0.06808	5.12642	1.02496
N	-0.27694	3.04421	1.34139
H	-0.54465	0.21558	1.69401
Si	0.78724	-0.49939	2.04726
H	0.63758	-0.56567	3.53914
H	0.89026	-1.89538	1.54128
C	4.04126	1.39798	0.24165
C	4.72002	1.94546	1.32921
C	2.85164	0.70190	0.43069
H	5.65798	2.48544	1.17543
H	2.31262	0.27561	-0.42200
C	4.20008	1.79776	2.61236
C	2.32518	0.52524	1.71976
H	4.72364	2.22595	3.47135
C	3.01327	1.09072	2.80253
H	2.62205	0.96707	3.81755
H	4.44751	1.50621	-0.76748
C	0.19289	-0.60787	-2.33618
O	1.37787	-0.67646	-3.12858
O	0.45759	-0.71904	-1.02813
C	1.41620	2.65388	-2.07538
H	2.40086	2.18555	-1.90123
H	0.80645	1.93497	-2.63486
H	1.56568	3.54307	-2.71151
C	-1.33294	3.10289	2.31271
H	-1.91826	2.17248	2.29065
H	-0.92070	3.22088	3.32829
H	-2.02677	3.94834	2.12575
C	-0.76019	-3.77646	-0.88440
H	0.29852	-3.85559	-0.57781
H	-1.07457	-4.74835	-1.29829
H	-0.81810	-3.02560	-1.68117
C	-3.12967	-1.27265	2.97606
H	-4.21258	-1.49265	2.88133
H	-2.85859	-1.35147	4.04113
H	-2.96531	-0.23155	2.66152
H	-6.18801	1.68223	-2.10785
Si	2.38240	-1.99817	-2.99357
H	1.54443	-3.23107	-2.87339
H	3.17963	-2.01356	-4.25254
C	3.60769	-1.96433	-1.57355
C	3.26830	-2.45008	-0.30343
C	4.89762	-1.44867	-1.75489
C	4.17523	-2.41054	0.75046
C	5.81139	-1.40087	-0.70331
C	5.44839	-1.88040	0.55234
H	2.26116	-2.84217	-0.13343
H	5.19593	-1.07442	-2.74078
H	3.88264	-2.78170	1.73659
H	6.80978	-0.98552	-0.86383
H	6.15782	-1.83593	1.38281
H	-0.29196	0.35452	-2.63026
C	-4.56668	-2.54789	-0.27874
H	-4.94782	-2.69226	-1.30225
H	-4.13267	-3.50102	0.04374
H	-5.41864	-2.30074	0.37868
C	-2.06719	4.15524	-1.26189
H	-1.09651	4.65154	-1.15225
H	-2.36702	4.25514	-2.31762
H	-2.80920	4.68285	-0.63737
H	-5.83882	-0.65115	-1.31570
H	-4.34576	3.33995	-1.90687

TS5_Co_L5

Co	0.38934	0.18447	0.25290
H	-0.05410	1.41102	-2.69753
P	0.20872	-1.98724	0.14513
N	1.87215	-2.39518	-0.34423
C	2.75560	-1.37409	-0.36889
N	4.03423	-1.61440	-0.68054
C	4.81287	-0.55322	-0.68143
N	4.46951	0.69254	-0.40853
C	3.18363	0.86626	-0.10360
N	2.27871	-0.14367	-0.05748
N	2.72633	2.10580	0.19365

P	1.02969	2.19142	0.68742
N	0.50256	3.70892	0.15443
C	0.17534	4.62355	1.23332
H	-0.91176	4.58846	1.44870
H	0.41998	5.66019	0.94547
C	0.97792	4.16543	2.43644
H	1.98753	4.62757	2.44619
H	0.48703	4.44125	3.38415
N	1.05769	2.72398	2.31128
N	-0.73338	-3.09975	-0.70635
C	-1.38909	-4.09753	0.12125
H	-1.37882	-5.07505	-0.39242
H	-2.44678	-3.82002	0.28799
C	-0.62030	-4.14660	1.43063
H	-1.28284	-4.38666	2.27918
H	0.17311	-4.92330	1.40812
N	-0.04851	-2.82759	1.60306
H	-0.23135	0.02404	1.81906
Si	-1.68863	0.53247	1.98582
H	-1.72122	0.69205	3.47710
H	-1.94335	1.86414	1.37521
C	-4.33785	-1.98532	-0.03572
C	-5.01192	-2.62109	1.00580
C	-3.32533	-1.07031	0.23467
H	-5.81162	-3.33375	0.78801
H	-2.79256	-0.57186	-0.58157
C	-4.66563	-2.34030	2.32501
C	-2.97825	-0.76105	1.55895
H	-5.18766	-2.83505	3.14833
C	-3.65822	-1.41523	2.59602
H	-3.40554	-1.19038	3.63740
H	-4.61054	-2.19567	-1.07337
C	-0.64162	0.53124	-2.31512
O	-1.74017	0.34547	-3.20065
O	-1.04483	0.67981	-1.04379
C	-1.27963	-2.88767	-2.02361
H	-2.34820	-2.61374	-1.98347
H	-0.75187	-2.08134	-2.54438
H	-1.18109	-3.80592	-2.62621
C	0.80005	-2.63105	2.74523
H	1.22117	-1.61591	2.73737
H	0.22586	-2.75138	3.67884
H	1.64258	-3.35129	2.76781
C	-0.23175	3.87983	-1.07644
H	-1.32318	3.84303	-0.90850
H	0.01750	4.84914	-1.53669
H	0.02147	3.08460	-1.78676
C	1.84145	2.01878	3.29036
H	2.89812	2.35295	3.30431
H	1.42188	2.16905	4.29740
H	1.82714	0.94008	3.07733
Si	-2.94537	1.49613	-3.27313
H	-2.31561	2.85054	-3.19815
H	-3.61254	1.28086	-4.58708
C	-4.26866	1.37292	-1.95065
C	-4.11793	2.00392	-0.70825
C	-5.43757	0.63367	-2.17367
C	-5.08841	1.88881	0.28133
C	-6.41336	0.50955	-1.18611
C	-6.23651	1.13516	0.04489
H	-3.20704	2.57592	-0.50669
H	-5.58902	0.14097	-3.14081
H	-4.94325	2.37843	1.24827
H	-7.31376	-0.08013	-1.37701
H	-6.99439	1.03306	0.82593
H	0.02282	-0.34901	-2.48715
C	3.64244	3.22621	0.23207
H	4.28463	3.22650	-0.65771
H	3.05655	4.15306	0.24782
H	4.29421	3.18483	1.11912
C	2.32646	-3.74957	-0.57933
H	1.44803	-4.38913	-0.72646
H	2.95485	-3.79565	-1.47791
H	2.91978	-4.12788	0.26796
C	6.28832	-0.75240	-1.00115
F	6.56241	-1.99670	-1.35992
F	6.66948	0.05614	-1.98893
F	7.03193	-0.45929	0.06941

TS5_Co_L6

Co	-1.07459	-0.05359	0.27371
H	-0.48972	-1.94300	-2.28351
P	-0.81921	1.98499	-0.51725
N	-2.50010	2.42320	-0.80274
C	-3.43715	1.49502	-0.48790
N	-4.72641	1.77765	-0.69274
C	-5.56824	0.79771	-0.40715
N	-5.24782	-0.41360	0.02144
C	-3.94252	-0.61977	0.20484
N	-2.99226	0.32123	0.01170
N	-3.51780	-1.84814	0.60336
P	-1.79256	-2.01360	0.86072
H	-0.73060	0.46065	1.84466
Si	0.75094	-0.03327	2.10898
H	0.74361	0.09439	3.60726
H	1.06007	-1.46404	1.83225
C	3.47258	2.13786	-0.20991
C	4.05797	2.99342	0.72243
C	2.50560	1.22102	0.18912
H	4.81882	3.71226	0.40722
H	2.04641	0.54135	-0.53664
C	3.67587	2.92124	2.05986
C	2.09704	1.14515	1.52981
H	4.13497	3.58353	2.79859
C	2.70443	2.00364	2.45713
H	2.41109	1.95824	3.51088
H	3.77812	2.17700	-1.25923
C	0.13025	-1.02876	-2.07923
O	1.31803	-1.14830	-2.85418
O	0.40984	-0.87220	-0.77225
H	-6.63591	1.00668	-0.54508
Si	2.45936	-2.28230	-2.41300
H	1.76222	-3.51903	-1.94058
H	3.23781	-2.54406	-3.65609
C	3.67149	-1.73104	-1.09545
C	3.46126	-2.03206	0.25587
C	4.80732	-0.98287	-1.43320
C	4.33911	-1.58748	1.23946
C	5.69030	-0.53086	-0.45493
C	5.45292	-0.82946	0.88438
H	2.57554	-2.60653	0.54488
H	5.00448	-0.74390	-2.48453
H	4.14552	-1.81899	2.29058
H	6.56474	0.06130	-0.73749
H	6.13667	-0.46644	1.65617
H	-0.42723	-0.17939	-2.53503
C	-4.49249	-2.89697	0.82672
H	-5.15058	-3.00566	-0.04558
H	-3.97152	-3.84756	0.99328
H	-5.12601	-2.67955	1.70022
C	-2.92914	3.69326	-1.35451
H	-2.05226	4.26309	-1.68356
H	-3.59057	3.53729	-2.21733
H	-3.48201	4.28789	-0.61172
C	-0.26715	3.37863	0.53691
H	-0.68787	3.26475	1.54555
H	0.83003	3.35747	0.60510
H	-0.57644	4.34625	0.11522
C	-1.73029	-2.59231	2.59979

H	-2.04066	-1.77674	3.26790
H	-2.36883	-3.47159	2.76967
H	-0.69150	-2.86657	2.83777
C	-0.01948	2.39820	-2.11483
H	0.94572	1.87399	-2.16307
H	-0.64265	2.03322	-2.94239
H	0.15505	3.47736	-2.22787
C	-1.31530	-3.52437	-0.03934
H	-0.21503	-3.56048	-0.01260
H	-1.72364	-4.44292	0.40489
H	-1.61992	-3.43968	-1.09073

TS5_Co_L7

Co	1.03832	0.05812	-0.28355
H	0.91106	-1.12801	2.42492
P	0.73081	2.12546	0.19593
N	2.35179	2.69137	0.48962
C	3.33903	1.76524	0.38106
N	4.60817	2.09786	0.61388
C	5.48356	1.11462	0.48646
N	5.21142	-0.14590	0.17896
C	3.92378	-0.40357	-0.03105
N	2.94328	0.52239	0.03084
N	3.53467	-1.67542	-0.32588
P	1.83855	-1.88169	-0.55940
O	1.52486	-3.21825	0.34512
C	0.77457	-4.15620	-0.41259
H	-0.29521	-3.88869	-0.37172
H	0.90804	-5.14959	0.03417
C	1.32296	-4.05475	-1.83043
H	2.19919	-4.70812	-1.97838
H	0.56788	-4.30135	-2.58946
O	1.71047	-2.69853	-1.99398
O	-0.13083	2.80786	1.42033
C	-0.77914	4.02028	1.06488
H	-0.20980	4.86928	1.47772
H	-1.78438	4.02033	1.50828
C	-0.83102	4.05622	-0.46285
H	-1.78515	3.66353	-0.84773
H	-0.67710	5.07162	-0.85467
O	0.23304	3.23572	-0.91980
H	0.74909	0.36146	-1.85031
Si	-0.77366	-0.19132	-1.98045
H	-0.77488	-0.08383	-3.47644
H	-1.00510	-1.62841	-1.67467
C	-3.66530	1.92332	0.21857
C	-4.37182	2.58676	-0.78257
C	-2.58445	1.10716	-0.10356
H	-5.22456	3.22089	-0.52621
H	-2.04203	0.57058	0.68020
C	-3.99152	2.42884	-2.11350
C	-2.18711	0.93479	-1.43910
H	-4.54067	2.94177	-2.90731
C	-2.90919	1.61114	-2.43438
H	-2.62338	1.49333	-3.48407
H	-3.96509	2.02718	1.26477
C	-0.07331	-0.66480	2.16314
O	-1.06542	-1.29788	2.94420
O	-0.35725	-0.77850	0.83815
H	6.53716	1.36493	0.65620
Si	-1.98547	-2.54661	2.33805
H	-1.16574	-3.58342	1.64543
H	-2.60598	-3.13184	3.56028
C	-3.37397	-2.01303	1.19621
C	-3.36933	-2.32502	-0.16758
C	-4.45219	-1.26990	1.69582
C	-4.38846	-1.89349	-1.01269
C	-5.47745	-0.83623	0.85958
C	-5.44264	-1.14264	-0.49909
H	-2.53868	-2.90459	-0.58248
H	-4.48608	-1.01874	2.76224
H	-4.35366	-2.13288	-2.07902
H	-6.30480	-0.24830	1.26649
H	-6.23793	-0.79051	-1.16124
H	-0.03928	0.39247	2.51413
C	4.52100	-2.73367	-0.42317
H	4.00475	-3.68700	-0.57931
H	5.20979	-2.54913	-1.25907
H	5.10909	-2.79726	0.50168
C	2.62459	4.06792	0.85049
H	2.25122	4.28503	1.86204
H	3.70587	4.23784	0.82775
H	2.13919	4.74303	0.13135

TS5_Co_L8

Co	-1.36206	0.14699	0.35331
H	-0.99920	-2.17716	-1.51837
C	-3.69504	0.83784	-1.03933
N	-4.96496	0.80290	-1.38078
C	-5.70305	-0.12589	-0.76741
N	-5.21547	-1.06813	0.04396
C	-3.93608	-0.96480	0.33178
N	-3.15400	0.04878	-0.08934
H	-0.93696	1.09900	1.64629
Si	0.60828	0.93299	1.98155
H	0.56056	1.56779	3.34166
H	1.07920	-0.46149	2.16398
C	3.38068	2.17223	-0.86503
C	3.49173	3.53934	-0.61759
C	2.53245	1.38602	-0.09087
H	4.15177	4.15553	-1.23381
H	2.40416	0.32531	-0.32109
C	2.76469	4.12012	0.42003
C	1.78976	1.95524	0.95402
H	2.85585	5.18999	0.62404
C	1.92956	3.32843	1.20447
H	1.37494	3.78995	2.02960
H	3.95137	1.71151	-1.67492
C	-0.18871	-1.41166	-1.69967
O	0.86145	-2.08954	-2.38434
O	0.23950	-0.82460	-0.57743
H	-6.77642	-0.14146	-0.97649
Si	1.90354	-3.03490	-1.49500
H	1.13614	-3.87608	-0.52350
H	2.57617	-3.89223	-2.51083
C	3.24530	-2.13706	-0.54131
C	3.19318	-2.01656	0.85290
C	4.34236	-1.57452	-1.20790
C	4.19233	-1.35343	1.55978
C	5.34424	-0.90359	-0.51027
C	5.26841	-0.79147	0.87601
H	2.34150	-2.43995	1.39549
H	4.41560	-1.66426	-2.29772
H	4.12674	-1.26343	2.64737
H	6.18883	-0.46642	-1.04943
H	6.05016	-0.26172	1.42627
H	-0.60987	-0.71075	-2.46676
C	-3.14363	-2.03103	1.00687
H	-3.73393	-2.58326	1.75961
H	-2.86852	-2.75312	0.21832
C	-2.65416	1.62443	-1.75791
H	-2.33765	1.00773	-2.61772
H	-3.04824	2.57284	-2.16494
N	-1.89556	-1.47994	1.57677
N	-1.47410	1.84253	-0.89647
C	-2.19525	-0.97959	2.92249

H	-1.29309	-0.56236	3.38610
H	-2.95811	-0.19133	2.86976
H	-2.56799	-1.79894	3.56478
C	-0.90292	-2.55604	1.65992
H	-0.60266	-2.86014	0.65132
H	-0.01101	-2.19561	2.18272
H	-1.31862	-3.41829	2.21312
C	-0.31315	2.11389	-1.75579
H	0.51068	2.50076	-1.15023
H	0.01845	1.18893	-2.24187
H	-0.57529	2.86944	-2.51864
C	-1.73688	3.01962	-0.06012
H	-0.87888	3.21472	0.59367
H	-1.90170	3.91076	-0.69382
H	-2.62795	2.85280	0.55980

TS5_Co_L9

Co	1.02644	-0.01132	-0.19805
H	0.40714	-1.10604	2.49116
P	0.90616	1.95654	0.72105
N	2.54385	2.39520	1.07764
C	3.48899	1.51129	0.53910
C	4.86417	1.79419	0.59581
C	5.77104	0.86945	0.08458
C	5.32338	-0.32654	-0.46920
C	3.94269	-0.57485	-0.53288
C	2.97809	0.33785	-0.05447
N	3.43898	-1.78208	-1.06136
P	1.79071	-1.98549	-0.56705
H	0.98954	0.54609	-1.70448
Si	-0.57390	0.14153	-2.02772
H	-0.48038	0.43221	-3.51088
H	-1.07911	-1.26740	-1.99630
C	-3.48419	2.23167	0.17199
C	-3.99677	3.14195	-0.75269
C	-2.49583	1.33032	-0.20690
H	-4.77367	3.85273	-0.45575
H	-2.09276	0.60806	0.50902
C	-3.51609	3.13257	-2.06011
C	-1.98436	1.31182	-1.51505
H	-3.91295	3.83931	-2.79548
C	-2.52178	2.22667	-2.43164
H	-2.14799	2.23442	-3.46104
H	-3.86345	2.21746	1.19795
C	-0.60071	-1.07984	2.01431
O	-1.24443	-2.31701	2.35555
O	-0.57408	-0.94524	0.68111
H	6.84401	1.08017	0.12904
Si	-2.16600	-2.95465	1.11655
H	-1.46642	-3.44245	-0.10555
H	-2.74603	-4.16716	1.78256
C	-3.61495	-1.86453	0.61762
C	-3.90909	-1.58523	-0.72205
C	-4.45161	-1.31963	1.60169
C	-4.99953	-0.79306	-1.07252
C	-5.54267	-0.52351	1.26011
C	-5.81872	-0.26068	-0.08057
H	-3.24820	-1.96729	-1.50713
H	-4.23880	-1.51907	2.65830
H	-5.19212	-0.56384	-2.12422
H	-6.17715	-0.09857	2.04347
H	-6.66259	0.37967	-0.35278
H	-1.18839	-0.27379	2.53153
C	4.33272	-2.88001	-1.28975
H	4.89815	-3.16610	-0.37849
H	3.77346	-3.76039	-1.63336
H	5.06995	-2.63856	-2.07425
C	2.95323	3.64821	1.63524
H	2.08780	4.18877	2.04030
H	3.66697	3.50567	2.46680
H	3.44135	4.30657	0.88883
C	0.34269	3.43795	-0.23147
H	0.81834	3.41304	-1.22296
H	-0.74801	3.40701	-0.36259
H	0.61159	4.37693	0.27715
C	1.12321	-3.12688	-1.84040
H	1.15532	-2.63213	-2.82175
H	1.66431	-4.08416	-1.88678
H	0.07325	-3.32904	-1.58395
C	0.03574	2.29093	2.31182
H	-1.04339	2.15424	2.14638
H	0.37289	1.56866	3.06763
H	0.20366	3.31560	2.67774
C	1.90849	-3.12154	0.87901
H	0.91467	-3.24039	1.33482
H	2.30508	-4.11141	0.60308
H	2.57486	-2.65005	1.61687
H	6.04698	-1.05218	-0.84759
H	5.23327	2.72176	1.03964

TS5_Co_L11

Co	-1.43889	0.07824	0.19543
H	-0.93180	-2.36825	-1.54022
C	-3.86253	1.01516	-0.99565
C	-5.24110	1.07097	-1.18434
C	-6.07160	0.17496	-0.49638
C	-5.50111	-0.81895	0.31160
C	-4.12016	-0.85505	0.48525
C	-3.27774	0.11434	-0.08848
H	-1.07720	1.10123	1.45301
Si	0.45218	0.96483	1.86546
H	0.42856	1.73834	3.16636
H	0.89513	-0.40111	2.27692
C	3.39620	1.94457	-0.95978
C	3.99025	3.08419	-0.41780
C	2.34587	1.31567	-0.29938
H	4.82123	3.57256	-0.93568
H	1.86524	0.42747	-0.72737
C	3.51913	3.59549	0.78939
C	1.85576	1.81409	0.92072
H	3.97692	4.48939	1.22431
C	2.46213	2.96554	1.44631
H	2.10624	3.37233	2.39912
H	3.75724	1.53451	-1.90689
C	-0.15853	-1.56725	-1.71440
O	0.96393	-2.23729	-2.34367
O	0.20777	-0.92994	-0.60913
H	-7.15709	0.22253	-0.62507
Si	2.02103	-3.01239	-1.32863
H	1.29929	-3.81178	-0.28976
H	2.77623	-3.93837	-2.23180
C	3.34110	-1.99181	-0.45952
C	3.20092	-1.60831	0.87983
C	4.50958	-1.60598	-1.13017
C	4.17929	-0.85749	1.52491
C	5.49315	-0.84982	-0.49506
C	5.32699	-0.47294	0.83574
H	2.28667	-1.87421	1.41825
H	4.65145	-1.89968	-2.17682
H	4.02941	-0.54607	2.56253
H	6.38974	-0.54539	-1.04289
H	6.08642	0.13704	1.33308
H	-0.57399	-0.91925	-2.52689
H	-6.14288	-1.57540	0.78027
H	-5.67978	1.79252	-1.88485
C	-3.33491	-1.98043	1.08112

H	-3.84965	-2.51803	1.90738
H	-3.14012	-2.72774	0.29143
C	-2.83029	1.70081	-1.83278
H	-2.62681	1.07457	-2.72011
H	-3.13298	2.70064	-2.21411
N	-2.00981	-1.49319	1.53844
N	-1.54926	1.80978	-1.09159
C	-2.18481	-0.93828	2.87797
H	-1.23219	-0.56329	3.27227
H	-2.90719	-0.11287	2.84132
H	-2.55881	-1.71857	3.57266
C	-1.07035	-2.60642	1.58791
H	-0.89732	-2.98903	0.57520
H	-0.10753	-2.26652	1.98773
H	-1.46148	-3.42123	2.23157
C	-0.45847	1.97357	-2.04701
H	0.46511	2.23910	-1.52161
H	-0.29095	1.03637	-2.59227
H	-0.69237	2.78047	-2.77141
C	-1.62273	3.00209	-0.24961
H	-0.69847	3.11898	0.33102
H	-1.75527	3.90775	-0.87656
H	-2.47228	2.91401	0.43955

TS5_Fe_L4

Fe	-0.95614	0.11587	0.25222
H	-0.82751	-1.37245	-2.51491
P	-0.41147	2.16497	0.14890
N	-1.63374	2.80093	-0.97045
C	-2.63233	1.93189	-1.32568
C	-3.69689	2.32178	-2.14812
C	-4.70318	1.40576	-2.43069
C	-4.64634	0.13708	-1.86506
C	-3.55938	-0.19422	-1.04724
N	-2.52707	0.67110	-0.80451
N	-3.47675	-1.40197	-0.40306
P	-2.16548	-1.53826	0.77789
N	-1.80801	-3.23832	0.68528
C	-2.12791	-4.00434	1.85945
H	-1.23517	-4.10999	2.51630
H	-2.45600	-5.03019	1.60044
C	-3.22438	-3.23431	2.57854
H	-4.23189	-3.53563	2.20920
H	-3.21627	-3.43938	3.66520
N	-2.94652	-1.84822	2.31291
N	0.98864	2.94971	-0.49489
C	1.59329	3.98173	0.29854
H	1.88258	4.84426	-0.33637
H	2.52116	3.62235	0.79169
C	0.54615	4.39410	1.32873
H	1.01461	4.68457	2.28707
H	-0.03065	5.28245	0.98121
N	-0.31135	3.25142	1.50704
H	-0.36113	0.16327	1.83579
Si	0.94924	-0.64033	2.05207
H	0.86262	-0.70133	3.55344
H	1.03156	-2.06026	1.60129
C	4.17437	1.23830	0.17307
C	4.97551	1.64154	1.24093
C	2.97041	0.58034	0.40257
H	5.92156	2.15785	1.05317
H	2.33401	0.26239	-0.43164
C	4.56620	1.38118	2.54606
C	2.54845	0.29768	1.71134
H	5.18554	1.69650	3.39124
C	3.36210	0.71476	2.77384
H	3.04999	0.50936	3.80320
H	4.49587	1.43022	-0.85422
C	-0.01278	-0.63604	-2.26637
O	1.07702	-0.91558	-3.17738
O	0.37677	-0.69123	-0.99497
C	1.54265	2.63125	-1.77276
H	2.63594	2.48487	-1.71661
H	1.10863	1.69836	-2.15724
H	1.35370	3.43014	-2.52144
C	-1.46038	3.42998	2.34088
H	-2.05205	2.50010	2.34077
H	-1.16870	3.65750	3.38154
H	-2.11393	4.25716	1.98444
C	-0.69900	-3.68052	-0.11522
H	0.14189	-4.03054	0.51847
H	-0.97441	-4.51064	-0.79138
H	-0.31948	-2.83935	-0.71500
C	-3.88035	-0.88309	2.80896
H	-4.91147	-1.04868	2.42650
H	-3.92476	-0.90278	3.91210
H	-3.55537	0.12209	2.49317
H	-5.53914	1.68548	-3.07688
Si	1.99564	-2.27038	-2.93353
H	1.13292	-3.43749	-2.57041
H	2.66653	-2.51916	-4.24775
C	3.38403	-2.16649	-1.67070
C	3.20906	-2.62590	-0.35921
C	4.63683	-1.64494	-2.02013
C	4.23970	-2.55638	0.57369
C	5.67366	-1.56932	-1.09216
C	5.47417	-2.02509	0.20883
H	2.23422	-3.01925	-0.05581
H	4.80455	-1.28586	-3.04226
H	4.06865	-2.89576	1.59922
H	6.63873	-1.14317	-1.38138
H	6.27732	-1.94833	0.94711
H	-0.38812	0.36167	-2.60066
C	-1.62513	4.14471	-1.47318
H	-0.68318	4.62686	-1.18429
H	-1.67925	4.16542	-2.57601
H	-2.46338	4.75017	-1.07961
C	-4.48514	-2.40307	-0.60052
H	-4.62842	-2.62000	-1.67303
H	-4.15609	-3.33350	-0.12210
H	-5.46613	-2.11031	-0.17900
H	-5.44251	-0.58630	-2.03672
H	-3.73908	3.33553	-2.54489

TS5_Fe_L9

Fe	1.01251	-0.10425	-0.00199
H	0.46922	-0.82427	2.67479
P	0.84455	1.91480	0.73576
N	2.47158	2.48550	1.04261
C	3.43617	1.64236	0.46864
C	4.79122	2.01363	0.44519
C	5.74151	1.13091	-0.07128
C	5.33266	-0.11798	-0.54527
C	3.96914	-0.45211	-0.53128
C	2.94259	0.41516	-0.06023
N	3.53482	-1.71608	-0.97172
P	1.87393	-2.01228	-0.52070
H	0.63056	0.43376	-1.56032
Si	-0.76185	-0.02309	-2.07254
H	-0.65707	0.11932	-3.57977
H	-1.19829	-1.43812	-1.87734
C	-3.63235	2.19181	0.02388
C	-4.22270	2.99576	-0.95629
C	-2.63794	1.28386	-0.31497
H	-5.00752	3.71125	-0.69004
H	-2.16668	0.66679	0.45628

C	-3.79617	2.87211	-2.28078
C	-2.19565	1.13348	-1.64605
H	-4.24425	3.49735	-3.06142
C	-2.80191	1.95422	-2.61455
H	-2.48093	1.86976	-3.65924
H	-3.95322	2.27158	1.06737
C	-0.56131	-0.87952	2.26649
O	-1.17633	-2.09616	2.63149
O	-0.57956	-0.90868	0.89352
H	6.80103	1.41042	-0.09258
Si	-1.70929	-2.61869	1.08504
H	-1.01361	-3.10905	-0.14728
H	-2.20088	-3.97413	1.60943
C	-3.37537	-1.80936	0.65812
C	-3.90249	-1.74980	-0.63799
C	-4.14361	-1.25465	1.69254
C	-5.13332	-1.15038	-0.89859
C	-5.37521	-0.65197	1.44306
C	-5.87369	-0.59440	0.14219
H	-3.31555	-2.14627	-1.47345
H	-3.75139	-1.28783	2.71558
H	-5.50133	-1.08751	-1.92746
H	-5.94412	-0.20862	2.26717
H	-6.82609	-0.09557	-0.06365
H	-1.15218	-0.02442	2.68390
C	2.81703	3.81816	1.41293
H	1.94672	4.33954	1.83462
H	3.60906	3.83349	2.18670
H	3.19067	4.42284	0.55682
C	4.48777	-2.73867	-1.25442
H	5.14420	-2.96846	-0.38573
H	3.97899	-3.66875	-1.54360
H	5.15282	-2.45816	-2.09407
C	1.35203	-3.05788	-1.97351
H	1.36896	-2.42203	-2.87183
H	1.99056	-3.94189	-2.13952
H	0.31947	-3.39666	-1.80126
C	0.26223	3.28727	-0.39761
H	0.78811	3.16453	-1.35678
H	-0.81990	3.20021	-0.57474
H	0.47486	4.29135	0.00768
C	-0.03487	2.53326	2.26580
H	-1.11226	2.35605	2.11619
H	0.30264	1.94908	3.13404
H	0.11176	3.60888	2.46370
C	2.06669	-3.39569	0.72557
H	1.07628	-3.64525	1.13531
H	2.52535	-4.31446	0.31811
H	2.68631	-3.00430	1.54666
H	6.08140	-0.81908	-0.92663
H	5.11747	2.98171	0.83744

TS5_Ni_L2

Ni	-0.98418	0.18254	0.13620
H	-1.00993	-1.71181	-2.37817
P	-0.11725	2.10729	-0.48323
O	-1.45900	2.68659	-1.47124
C	-2.56418	1.98712	-1.50606
N	-3.56593	2.38466	-2.26741
C	-4.61911	1.57763	-2.28147
N	-4.73363	0.41629	-1.64455
C	-3.69833	0.08643	-0.90182
N	-2.61209	0.86967	-0.75196
O	-3.69705	-1.06549	-0.26684
P	-2.31674	-1.44235	0.73150
N	-2.14040	-3.07268	0.65543
C	-2.70643	-3.76139	1.81594
H	-1.88042	-4.10438	2.46495
H	-3.27648	-4.64645	1.49567
C	-3.58781	-2.74796	2.52527
H	-4.62009	-2.76722	2.12491
H	-3.63987	-2.92914	3.60848
N	-2.97408	-1.44146	2.26984
N	1.22738	2.50525	-1.33236
C	1.85709	3.73914	-0.85480
H	2.10732	4.38559	-1.70977
H	2.79438	3.47511	-0.33497
C	0.86491	4.40762	0.08334
H	1.36972	4.94067	0.90300
H	0.22739	5.13485	-0.45568
N	0.03930	3.33219	0.63152
H	-0.75440	0.59210	1.74877
Si	0.49283	-0.16167	2.36568
H	0.09175	-0.02208	3.79375
H	0.50442	-1.58755	1.94836
C	4.00164	1.20085	0.63012
C	4.41394	2.19624	1.51354
C	2.82065	0.50073	0.86358
H	5.34793	2.73325	1.33141
H	2.49888	-0.27727	0.16271
C	3.64395	2.49975	2.63549
C	2.04786	0.78319	1.99907
H	3.96947	3.27525	3.33241
C	2.46946	1.79408	2.87703
H	1.88139	2.02176	3.77243
H	4.61280	0.95175	-0.24071
C	-0.15660	-0.99718	-2.23517
O	0.91141	-1.40155	-3.03555
O	0.22762	-0.91226	-0.92437
C	1.95472	1.69249	-2.28905
H	3.02847	1.75010	-2.05535
H	1.66167	0.63875	-2.22649
H	1.80179	2.05096	-3.31784
C	-1.04108	3.71655	1.50941
H	-1.62745	2.83623	1.80869
H	-0.63403	4.17525	2.42250
H	-1.72324	4.44074	1.02805
C	-1.20107	-3.78116	-0.19178
H	-0.55876	-4.42439	0.43050
H	-1.71584	-4.41293	-0.93038
H	-0.55460	-3.06148	-0.70568
C	-3.68488	-0.28364	2.77124
H	-4.71152	-0.22246	2.36659
H	-3.74566	-0.33443	3.86710
H	-3.14803	0.63886	2.50800
H	-5.47568	1.89405	-2.88627
Si	1.83481	-2.76561	-2.69850
H	0.90546	-3.88140	-2.35138
H	2.57221	-3.01769	-3.95965
C	3.06054	-2.55236	-1.30143
C	2.77207	-2.99651	-0.00300
C	4.32319	-1.99087	-1.53926
C	3.70521	-2.88230	1.02271
C	5.26273	-1.87228	-0.51691
C	4.95315	-2.31755	0.76542
H	1.79717	-3.44589	0.20991
H	4.58742	-1.65936	-2.54972
H	3.46457	-3.24234	2.02628
H	6.24563	-1.44177	-0.72445
H	5.69017	-2.23155	1.56737
H	-0.50141	-0.02454	-2.66162

TS5_Ni_L3

Ni	-1.19128	0.06260	0.00767
H	-0.36738	-1.61311	-2.58563
P	-0.70906	2.18994	-0.39315

C	-2.17917	2.67080	-1.43301
C	-3.28536	1.68897	-1.30152
N	-4.50408	2.02348	-1.69625
C	-5.42336	1.07033	-1.62463
N	-5.19143	-0.18861	-1.27302
C	-3.95617	-0.46614	-0.89067
N	-2.98025	0.46680	-0.81333
C	-3.58599	-1.86614	-0.55759
P	-2.07143	-1.89007	0.52079
N	-1.46277	-3.45024	0.42729
C	-1.52636	-4.14250	1.70934
H	-0.56316	-4.05145	2.24743
H	-1.71976	-5.21375	1.54299
C	-2.64714	-3.47506	2.48090
H	-3.63264	-3.89258	2.18781
H	-2.53859	-3.60207	3.56846
N	-2.54887	-2.06128	2.13652
N	0.58618	2.96868	-1.12086
C	1.20107	3.95628	-0.23586
H	1.55838	4.80308	-0.84240
H	2.06693	3.52874	0.29740
C	0.10963	4.37775	0.72824
H	0.52003	4.75811	1.67650
H	-0.52536	5.17482	0.28863
N	-0.66934	3.17200	0.98023
H	-0.86921	0.34439	1.57754
Si	0.53815	-0.31685	1.91649
H	0.24284	-0.37041	3.38243
H	0.70111	-1.69718	1.41005
C	3.90500	1.66299	0.46759
C	4.25221	2.47440	1.54570
C	2.77234	0.85702	0.53336
H	5.14808	3.09772	1.49500
H	2.49585	0.22026	-0.30957
C	3.45840	2.48750	2.69131
C	1.97391	0.84497	1.68435
H	3.72729	3.12124	3.53952
C	2.32972	1.67544	2.75964
H	1.72718	1.67393	3.67390
H	4.52411	1.64984	-0.43275
C	0.24895	-0.72600	-2.26814
O	1.46568	-0.76926	-2.95924
O	0.42861	-0.68319	-0.92476
C	1.36206	2.45653	-2.23050
H	2.15187	1.75578	-1.91581
H	0.72191	1.92815	-2.94901
H	1.82565	3.30400	-2.75543
C	-1.80791	3.28557	1.85581
H	-2.30386	2.31055	1.97109
H	-1.48190	3.61108	2.85494
H	-2.55566	4.01567	1.48799
C	-0.46525	-3.88722	-0.52257
H	0.54126	-3.90100	-0.07114
H	-0.70034	-4.90126	-0.87909
H	-0.43022	-3.21213	-1.38580
C	-3.51687	-1.16148	2.71506
H	-4.55486	-1.38885	2.40119
H	-3.47458	-1.22479	3.81173
H	-3.28543	-0.12312	2.43508
H	-6.45132	1.33861	-1.89123
H	-1.83488	2.65039	-2.48319
H	-3.28389	-2.36959	-1.49490
H	-2.52385	3.69933	-1.24524
H	-4.43951	-2.43170	-0.15507
Si	2.52962	-2.05938	-2.80138
H	1.70724	-3.29722	-2.64502
H	3.32111	-2.06216	-4.05660
C	3.70073	-1.90376	-1.35283
C	3.37284	-2.40435	-0.08496
C	4.94296	-1.27395	-1.51060
C	4.24429	-2.27101	0.99074
C	5.82200	-1.13655	-0.43793
C	5.47095	-1.63335	0.81435
H	2.41065	-2.90346	0.06465
H	5.23580	-0.89183	-2.49480
H	3.97070	-2.66976	1.97107
H	6.78831	-0.64725	-0.58204
H	6.15876	-1.52955	1.65686
H	-0.29559	0.16271	-2.67399

TS5_Ni_L4

Ni	-0.90371	0.13946	0.16285
H	-0.83415	-1.38149	-2.67989
P	-0.30563	2.21759	-0.24933
N	-1.78013	2.76595	-1.01706
C	-2.86792	1.92862	-1.03544
C	-4.09416	2.31296	-1.59340
C	-5.12949	1.39286	-1.59874
C	-4.95760	0.11881	-1.07550
C	-3.71291	-0.20272	-0.52931
N	-2.70995	0.70294	-0.48521
N	-3.44440	-1.45161	-0.00762
P	-1.88142	-1.72261	0.69041
N	-1.47005	-3.29686	0.33751
C	-1.19334	-4.10441	1.51566
H	-0.10854	-4.12843	1.73161
H	-1.51473	-5.14180	1.33137
C	-1.97647	-3.46660	2.65390
H	-2.99483	-3.89747	2.73081
H	-1.48284	-3.61956	3.62629
N	-2.04003	-2.04641	2.34192
N	0.94938	2.90446	-1.11047
C	1.74673	3.84428	-0.32653
H	2.03170	4.69629	-0.96514
H	2.67550	3.35786	0.01591
C	0.88789	4.28585	0.84845
H	1.50029	4.46651	1.74683
H	0.34189	5.22523	0.62947
N	-0.04434	3.19875	1.09279
H	-0.58077	0.43824	1.78377
Si	0.76621	-0.25386	2.24307
H	0.49215	-0.23869	3.71006
H	0.86791	-1.64412	1.73871
C	4.15755	1.24623	0.39837
C	4.66673	2.12990	1.34827
C	2.96579	0.56834	0.64042
H	5.60839	2.65084	1.15974
H	2.56559	-0.11993	-0.11134
C	3.97929	2.34482	2.54182
C	2.27623	0.75764	1.84737
H	4.37791	3.03560	3.28848
C	2.79308	1.65927	2.78952
H	2.27343	1.81261	3.74160
H	4.69629	1.07383	-0.53666
C	-0.05887	-0.62916	-2.37377
O	1.05051	-0.77234	-3.21835
O	0.30302	-0.76182	-1.07139
C	1.58810	2.32173	-2.27237
H	2.43247	1.66890	-1.99363
H	0.88214	1.72410	-2.85810
H	1.96554	3.13038	-2.91568
C	-1.03646	3.38107	2.11926
H	-1.71594	2.51715	2.15936
H	-0.55615	3.47893	3.10616
H	-1.64949	4.28555	1.94461
C	-1.11405	-3.80374	-0.96473
H	-0.04074	-4.04103	-1.03644
H	-1.68771	-4.71895	-1.18239
H	-1.34277	-3.06207	-1.73958

C	-2.83912	-1.19931	3.19331
H	-3.89888	-1.51623	3.21692
H	-2.45252	-1.21744	4.22325
H	-2.80282	-0.15811	2.83952
H	-6.09374	1.67173	-2.02824
Si	2.00053	-2.15215	-3.12601
H	1.07584	-3.32519	-3.08737
H	2.82375	-2.12751	-4.36061
C	3.15587	-2.25187	-1.65459
C	2.72924	-2.74982	-0.41422
C	4.49912	-1.87404	-1.78173
C	3.60722	-2.86798	0.65671
C	5.38446	-1.98091	-0.70947
C	4.93864	-2.47986	0.51048
H	1.67861	-3.02737	-0.28246
H	4.86769	-1.50034	-2.74329
H	3.25574	-3.26435	1.61341
H	6.42878	-1.68334	-0.83135
H	5.63040	-2.57108	1.35112
H	-0.50737	0.36343	-2.61808
C	-4.47943	-2.46514	0.03884
H	-4.84931	-2.70175	-0.97044
H	-4.05890	-3.38468	0.46027
H	-5.32838	-2.14734	0.66468
C	-1.89938	4.12462	-1.50640
H	-0.91712	4.60631	-1.46175
H	-2.22276	4.14239	-2.55796
H	-2.61180	4.71103	-0.90361
H	-5.76794	-0.60679	-1.09214
H	-4.22845	3.30679	-2.01462

H	2.21124	-2.66117	1.38183
H	4.29358	-1.74675	-2.28036
H	3.99941	-1.53933	2.67827
H	6.07273	-0.61716	-0.98194
H	5.92888	-0.50428	1.49782
H	-1.04147	-1.04923	-2.20207
C	-3.07151	-1.82357	1.26963
H	-3.64957	-2.26326	2.09908
H	-2.82517	-2.65487	0.58602
C	-2.54624	1.43221	-1.89301
H	-2.28027	0.73871	-2.70999
H	-2.90152	2.35769	-2.37513
N	-1.80723	-1.20919	1.75030
N	-1.33998	1.68369	-1.06281
C	-2.09759	-0.56226	3.04539
H	-2.50605	-1.30540	3.74943
H	-1.18214	-0.15168	3.48432
H	-2.83034	0.24458	2.91407
C	-0.81799	-2.28499	1.95084
H	-0.57076	-2.74684	0.99003
H	0.09812	-1.86888	2.37995
H	-1.23133	-3.03887	2.64140
C	-0.16731	1.75811	-1.95959
H	0.69268	2.13529	-1.40177
H	0.07722	0.76388	-2.34873
H	-0.38921	2.44912	-2.78952
C	-1.52931	2.99326	-0.40570
H	-2.41278	2.96943	0.24577
H	-0.64251	3.25203	0.18080
H	-1.67121	3.77255	-1.17221

TS5_Ni_L8

Ni	-1.26047	0.24099	0.39224
H	-1.03852	-2.47412	-1.12193
C	-3.61494	0.73715	-1.12280
N	-4.83777	0.55537	-1.58131
C	-5.57252	-0.32178	-0.89840
N	-5.10929	-1.12532	0.05851
C	-3.87531	-0.87481	0.44896
N	-3.16203	0.15095	-0.01464
H	-0.95386	1.38091	1.58704
Si	0.55490	1.33089	2.06176
H	0.38772	2.10052	3.32791
H	0.94795	-0.06510	2.36052
C	3.44100	2.02358	-0.81923
C	3.54418	3.41284	-0.83301
C	2.54979	1.39687	0.04754
H	4.24019	3.90047	-1.51946
H	2.44774	0.30760	0.02636
C	2.77000	4.18324	0.03409
C	1.75644	2.15993	0.91780
H	2.86399	5.27132	0.03565
C	1.89006	3.55769	0.91127
H	1.30349	4.16840	1.60705
H	4.05400	1.41931	-1.49075
C	-0.39848	-1.62329	-1.48931
O	0.64036	-2.15000	-2.25177
O	0.08781	-0.87091	-0.46654
H	-6.62593	-0.42453	-1.17809
Si	1.78222	-3.15038	-1.52458
H	1.03441	-4.04896	-0.59097
H	2.40280	-3.88102	-2.65529
C	3.10714	-2.26675	-0.54584
C	3.05287	-2.20069	0.85352
C	4.21034	-1.68862	-1.18950
C	4.05490	-1.57208	1.58728
C	5.21671	-1.05574	-0.46305
C	5.13753	-0.99469	0.92609

TS5_Ni_L10

Ni	-1.21996	0.44761	0.75405
H	-0.83067	0.81124	-2.17317
C	-3.52628	1.33118	-0.54873
C	-4.85729	1.21534	-0.95056
C	-5.57854	0.06559	-0.62865
C	-4.99213	-0.96450	0.10765
C	-3.66236	-0.84962	0.51075
C	-2.94152	0.28957	0.16437
H	0.17156	0.81820	1.86664
Si	1.63229	0.39781	1.70140
H	2.05420	0.47433	3.14117
H	1.78120	-0.99539	1.23990
C	3.58099	2.67702	-1.19666
C	3.88141	3.84918	-0.50386
C	2.93674	1.62908	-0.54650
H	4.37847	4.67619	-1.01766
H	2.67079	0.72035	-1.08893
C	3.55708	3.96214	0.84629
C	2.58796	1.73147	0.80632
H	3.80208	4.87491	1.39531
C	2.92395	2.90511	1.49615
H	2.67836	2.99924	2.55968
H	3.84446	2.58264	-2.25300
C	0.10570	0.24252	-1.94254
O	-0.00693	-0.98627	-2.66732
O	0.29948	0.02819	-0.63504
H	-6.62072	-0.02298	-0.94406
Si	0.02771	-2.38330	-1.76894
H	-1.03610	-2.50395	-0.72485
H	-0.24210	-3.44921	-2.77768
C	1.68288	-2.73061	-0.94735
C	1.79592	-3.68072	0.07423
C	2.83643	-2.03301	-1.32374
C	3.00864	-3.90639	0.72347
C	4.05260	-2.24960	-0.68234
C	4.13887	-3.18292	0.34996

H	0.91504	-4.26072	0.37650
H	2.77508	-1.29608	-2.13134
H	3.07330	-4.64845	1.52370
H	4.93830	-1.68436	-0.98451
H	5.09077	-3.34995	0.86058
H	0.94433	0.80462	-2.42754
C	-2.91101	-1.85045	1.31690
H	-3.49001	-2.23989	2.17452
H	-2.58846	-2.71285	0.70160
C	-2.64689	2.50534	-0.81125
H	-2.34637	2.55400	-1.87672
H	-3.12922	3.46907	-0.55901
H	-5.57596	-1.85080	0.37295
H	-5.33688	2.02441	-1.50950
O	-1.74184	-1.18681	1.81426
O	-1.47508	2.34133	-0.01426
C	-0.80508	-2.06917	2.39903
H	-0.27154	-2.64510	1.62544
H	-0.08720	-1.47481	2.97612
H	-1.32749	-2.75499	3.08460
C	-0.41812	3.19822	-0.39689
H	-0.75853	4.24543	-0.34916
H	0.41844	3.05207	0.29379
H	-0.08073	2.96655	-1.42067

4.5.6. TS6

TS6_Co_L1

Co	-1.34435	0.03601	0.06045
H	0.50094	-2.95839	-1.80083
P	-0.30135	1.87785	0.27757
N	-1.18700	2.88541	-0.90580
C	-2.27295	2.31184	-1.46750
N	-3.06637	3.01968	-2.28003
C	-4.14313	2.38170	-2.70829
N	-4.54885	1.17672	-2.34027
C	-3.71583	0.53019	-1.51749
N	-2.50980	1.01757	-1.14363
N	-4.07398	-0.66038	-0.98862
P	-2.99187	-1.28259	0.29695
N	-3.12678	-2.96825	0.04288
C	-3.71778	-3.68661	1.15237
H	-2.93273	-4.06999	1.83577
H	-4.29140	-4.55677	0.78724
C	-4.60919	-2.68391	1.86510
H	-5.63065	-2.67252	1.42922
H	-4.71816	-2.92485	2.93580
N	-3.94521	-1.40922	1.70344
N	1.30705	2.25393	-0.14302
C	2.07887	2.82229	0.94405
H	2.83686	3.51826	0.54413
H	2.61413	2.03363	1.50783
C	1.07245	3.53534	1.83448
H	1.39894	3.53626	2.88883
H	0.94023	4.59510	1.53248
N	-0.15565	2.78397	1.69968
C	-0.87274	4.27670	-1.13614
H	-1.15595	4.56735	-2.15479
H	0.20890	4.41519	-1.00671
H	-1.40707	4.93408	-0.43019
C	-5.35823	-1.23908	-1.31309
H	-6.18455	-0.71492	-0.80606
H	-5.35029	-2.29293	-1.00819
H	-5.54119	-1.18159	-2.39387
H	-0.55575	-0.47799	1.29078
Si	0.37408	-1.88408	0.98545
H	-0.67954	-2.54174	1.84716
H	1.05827	-3.14462	0.48498
C	4.08426	-0.44265	2.21144
C	3.79253	0.32709	3.33562
C	3.05955	-1.11325	1.54565
H	4.59425	0.84591	3.86782
H	3.29314	-1.72368	0.66708
C	2.47392	0.43810	3.77702
C	1.72841	-1.00794	1.96811
H	2.24004	1.04194	4.65869
C	1.45282	-0.20936	3.08586
H	0.41518	-0.08758	3.41777
H	5.11180	-0.52451	1.84721
C	0.86839	-1.91229	-1.80533
O	2.26993	-1.97676	-1.75680
O	0.36430	-1.19073	-0.75653
C	2.01093	1.49172	-1.13867
H	2.54681	0.62635	-0.70377
H	1.30121	1.10899	-1.88556
H	2.74075	2.13407	-1.65905
C	-1.26367	3.07250	2.55905
H	-2.07424	2.35335	2.36683
H	-0.97105	2.97831	3.61911
H	-1.66532	4.09373	2.40642
C	-2.19519	-3.68240	-0.78837
H	-1.35023	-4.10466	-0.20927
H	-2.70016	-4.51337	-1.30770
H	-1.78285	-3.00969	-1.55400
C	-4.53666	-0.24570	2.29970
H	-5.54305	-0.02196	1.89181
H	-4.63502	-0.37528	3.38961
H	-3.89130	0.62702	2.11763
H	-4.77932	2.91696	-3.42445
Si	3.38652	-1.31001	-2.79375
H	3.70907	-2.29037	-3.87064
H	2.85193	-0.06284	-3.41783
C	4.90868	-0.93341	-1.77491
C	5.49539	-1.93783	-0.99045
C	5.49412	0.33855	-1.76579
C	6.62709	-1.67993	-0.22292
C	6.62878	0.60355	-1.00158
C	7.19657	-0.40715	-0.23052
H	5.05215	-2.93930	-0.97151
H	5.05770	1.14106	-2.36962
H	7.06817	-2.47460	0.38374
H	7.07108	1.60271	-1.00769
H	8.08682	-0.20310	0.36993
H	0.53686	-1.43121	-2.74992

TS6_Co_L4

Co	-1.364632000	0.048149000	0.092061000
H	0.526911000	-2.856408000	-1.931980000
P	-0.331208000	1.878770000	0.371569000
N	-1.165585000	2.887837000	-0.825742000
C	-2.282753000	2.348859000	-1.403610000
C	-3.109248000	3.088774000	-2.263505000
C	-4.257399000	2.485936000	-2.753278000
C	-4.602137000	1.200296000	-2.364295000
C	-3.736407000	0.512633000	-1.500732000
N	-2.567532000	1.060644000	-1.082483000
N	-4.030848000	-0.730741000	-1.007241000
P	-2.971866000	-1.318918000	0.292015000
N	-3.053802000	-3.009779000	0.032068000
C	-3.604652000	-3.753406000	1.143610000
H	-2.802653000	-4.099011000	1.827680000
H	-4.137229000	-4.650500000	0.780718000
C	-4.542186000	-2.791815000	1.853840000
H	-5.562567000	-2.828014000	1.414648000
H	-4.645265000	-3.038436000	2.924077000
N	-3.938254000	-1.488600000	1.692646000
N	1.295296000	2.261068000	0.035018000
C	2.021514000	2.825414000	1.152480000
H	2.781571000	3.538893000	0.787546000
H	2.552104000	2.038524000	1.722057000
C	0.975955000	3.513747000	2.017655000
H	1.266433000	3.504681000	3.082784000
H	0.845102000	4.578375000	1.727538000
N	-0.242362000	2.759474000	1.826222000
C	-0.791033000	4.253422000	-1.086421000
H	-0.656852000	4.437491000	-2.164915000
H	0.171820000	4.451256000	-0.601716000
H	-1.537881000	4.970929000	-0.702692000
C	-5.264768000	-1.381271000	-1.366048000
H	-6.150166000	-0.840883000	-0.985462000
H	-5.260194000	-2.394767000	-0.948820000
H	-5.363136000	-1.477215000	-2.459366000
H	-0.536863000	-0.499644000	1.281396000
Si	0.390799000	-1.889553000	0.903928000
H	-0.631233000	-2.589832000	1.770074000
H	1.068105000	-3.127220000	0.337641000
C	4.139643000	-0.581074000	2.172859000
C	3.866867000	0.146052000	3.329602000

C	3.098344000	-1.193483000	1.477270000
H	4.681344000	0.619237000	3.884581000
H	3.316260000	-1.771724000	0.573229000
C	2.550747000	0.273568000	3.774324000
C	1.769610000	-1.070042000	1.902283000
H	2.331839000	0.845081000	4.680942000
C	1.513732000	-0.314894000	3.054455000
H	0.479233000	-0.178897000	3.389556000
H	5.165322000	-0.675071000	1.805977000
C	0.860026000	-1.799479000	-1.887154000
O	2.263910000	-1.821319000	-1.826306000
O	0.325242000	-1.141455000	-0.814028000
C	2.040829000	1.563329000	-0.975152000
H	2.635972000	0.728143000	-0.558194000
H	1.350803000	1.144554000	-1.720559000
H	2.726785000	2.256608000	-1.491968000
C	-1.424770000	3.168951000	2.524087000
H	-2.242490000	2.466250000	2.304825000
H	-1.257852000	3.166535000	3.614004000
H	-1.756672000	4.186534000	2.232845000
C	-2.133676000	-3.690494000	-0.837237000
H	-1.268188000	-4.114093000	-0.290912000
H	-2.639256000	-4.516675000	-1.364730000
H	-1.749155000	-2.992968000	-1.595225000
C	-4.620990000	-0.350995000	2.238757000
H	-5.626060000	-0.204332000	1.792165000
H	-4.747985000	-0.457141000	3.328311000
H	-4.028538000	0.557191000	2.051617000
H	-4.911713000	3.039538000	-3.430983000
Si	3.369845000	-1.116307000	-2.847179000
H	3.655747000	-2.035308000	-3.987576000
H	2.852557000	0.175583000	-3.389902000
C	4.912794000	-0.829797000	-1.830656000
C	5.502143000	-1.897488000	-1.137037000
C	5.507548000	0.433589000	-1.727675000
C	6.645520000	-1.709446000	-0.366761000
C	6.654227000	0.628979000	-0.960361000
C	7.224619000	-0.443865000	-0.280709000
H	5.050640000	-2.893940000	-1.191510000
H	5.067588000	1.285041000	-2.257407000
H	7.088144000	-2.552816000	0.168860000
H	7.103113000	1.622828000	-0.891229000
H	8.123774000	-0.294068000	0.322490000
H	0.522459000	-1.288544000	-2.813829000
H	-5.527485000	0.741099000	-2.706532000
H	-2.858213000	4.114238000	-2.528264000

TS6_Co_L7

Co	-1.10978	-0.25713	0.51061
H	0.28882	-1.38027	-3.13040
P	0.10614	1.10088	1.56339
N	-0.62850	2.63505	1.13564
C	-1.80709	2.53831	0.47171
N	-2.51303	3.62988	0.17631
C	-3.68175	3.40087	-0.40300
N	-4.23192	2.22039	-0.64158
C	-3.47044	1.17142	-0.32947
N	-2.21478	1.28843	0.15432
N	-3.93637	-0.09520	-0.46690
P	-2.85411	-1.35136	0.12176
O	-3.00087	-2.42844	-1.14512
C	-3.55884	-3.66661	-0.75231
H	-2.74947	-4.36994	-0.49012
H	-4.12876	-4.08371	-1.59491
C	-4.44046	-3.36037	0.45149
H	-5.46421	-3.08479	0.14564
H	-4.50085	-4.20257	1.15394
O	-3.82457	-2.26484	1.10843
O	1.66820	1.40850	1.11057
C	2.58868	1.32146	2.18187
H	3.39602	2.04862	2.00864
H	3.02391	0.30967	2.21144
C	1.78962	1.64024	3.44258
H	2.15211	1.08002	4.31572
H	1.81273	2.71733	3.67961
O	0.45262	1.24388	3.17452
C	-0.04930	3.91666	1.47503
H	-0.44405	4.68678	0.80292
H	1.04114	3.85957	1.35043
H	-0.28305	4.20247	2.51200
C	-5.27265	-0.36259	-0.95528
H	-5.96082	-0.58559	-0.12526
H	-5.24440	-1.21738	-1.64477
H	-5.64657	0.51666	-1.49095
H	-0.12032	-1.37184	0.93239
Si	0.59967	-2.19992	-0.38333
H	-0.42094	-3.26778	-0.12027
H	1.25255	-2.75146	-1.63898
C	4.49601	-2.02413	1.08239
C	4.34890	-2.15272	2.46113
C	3.37285	-2.03965	0.25654
H	5.22963	-2.15589	3.10864
H	3.50036	-1.94684	-0.82717
C	3.07304	-2.27512	3.01334
C	2.08534	-2.15889	0.79155
H	2.95033	-2.37633	4.09517
C	1.95469	-2.25812	2.18438
H	0.95533	-2.32114	2.62951
H	5.49124	-1.91580	0.64358
C	-0.18152	-0.55171	-2.56039
O	0.12904	0.66202	-3.16172
O	0.27233	-0.56760	-1.24585
H	-4.26338	4.28213	-0.69893
Si	1.23731	1.74785	-2.53568
H	1.29939	2.79465	-3.59148
H	0.78581	2.37877	-1.26450
C	2.91905	0.96064	-2.28668
C	3.36332	-0.06288	-3.13386
C	3.77602	1.39592	-1.26876
C	4.62631	-0.62848	-2.97481
C	5.03988	0.83627	-1.10387
C	5.46777	-0.17530	-1.96075
H	2.70912	-0.42619	-3.93333
H	3.43821	2.18072	-0.58515
H	4.95611	-1.42661	-3.64446
H	5.69299	1.18574	-0.29940
H	6.45946	-0.61825	-1.83377
H	-1.28091	-0.68164	-2.60156

TS6_Co_L8

Co	1.73991	0.67456	0.00078
H	0.78021	-1.42956	-1.75775
C	2.21684	-1.90062	1.05711
N	2.92568	-2.98708	1.30204
C	4.11707	-3.05450	0.70992
N	4.64991	-2.10458	-0.06380
C	3.88548	-1.05234	-0.26649
N	2.65360	-0.91257	0.25237
H	1.09314	2.11992	0.15135
Si	0.09972	2.19672	-1.14063
H	0.56775	3.66192	-1.18629
H	0.27020	1.92268	-2.60952
C	-3.89086	1.86427	0.13494
C	-4.01073	2.94295	1.00961
C	-2.68926	1.63466	-0.53076
H	-4.95019	3.11449	1.54202

H	-2.59250	0.76681	-1.18989
C	-2.93216	3.80463	1.19670
C	-1.59087	2.48434	-0.35137
H	-3.02126	4.65657	1.87636
C	-1.74240	3.58110	0.50723
H	-0.90139	4.27036	0.64477
H	-4.73468	1.18880	-0.02279
C	0.10528	-0.58610	-2.03386
O	-1.10460	-1.12358	-2.51718
O	-0.08371	0.23864	-0.96448
H	4.71590	-3.95370	0.88377
Si	-1.85248	-2.43513	-1.81197
H	-2.38318	-3.26206	-2.92855
H	-0.82723	-3.17789	-1.01154
C	-3.27515	-2.00843	-0.67383
C	-4.51081	-1.60185	-1.19926
C	-3.16321	-2.13985	0.71620
C	-5.59319	-1.33334	-0.36688
C	-4.23876	-1.86547	1.55761
C	-5.45610	-1.46457	1.01428
H	-4.63128	-1.50105	-2.28361
H	-2.21631	-2.47216	1.15506
H	-6.54849	-1.02045	-0.79572
H	-4.12774	-1.96958	2.63974
H	-6.30409	-1.25275	1.67048
H	0.56910	-0.04957	-2.88850
C	4.27574	0.12413	-1.09900
H	5.37418	0.24790	-1.13693
H	3.92335	-0.05773	-2.12922
C	0.86095	-1.65407	1.63656
H	0.11539	-1.97563	0.88750
H	0.71117	-2.27830	2.54114
N	3.58581	1.32403	-0.58562
N	0.66794	-0.22764	1.87634
C	4.29450	1.83987	0.58948
H	5.31263	2.18231	0.31561
H	3.73162	2.68164	1.01138
H	4.38060	1.05867	1.35621
C	3.54088	2.35887	-1.61920
H	2.98853	1.98629	-2.49197
H	3.01585	3.24117	-1.23060
H	4.56248	2.65243	-1.92903
C	-0.74292	0.09256	2.03168
H	-1.18899	-0.45139	2.89004
H	-0.86052	1.17171	2.20174
H	-1.28172	-0.16141	1.11198
C	1.42010	0.20983	3.04024
H	2.48358	-0.05040	2.93822
H	1.34126	1.30128	3.13700
H	1.03714	-0.25893	3.97132

TS6_Co_L9

Co	-1.485689000	0.228559000	0.708445000
H	-0.012541000	-2.249644000	-0.588309000
P	-1.550761000	2.288467000	0.162555000
N	-2.670063000	2.359617000	-1.164290000
C	-3.387496000	1.164724000	-1.327912000
C	-4.445956000	1.057379000	-2.245935000
C	-5.150819000	-0.142341000	-2.328977000
C	-4.833006000	-1.218540000	-1.503159000
C	-3.766326000	-1.088985000	-0.597326000
C	-3.000665000	0.090958000	-0.496978000
N	-3.417848000	-2.121578000	0.294298000
P	-2.344269000	-1.517887000	1.532159000
C	-3.052662000	3.546284000	-1.864472000
H	-2.999656000	3.409080000	-2.960236000
H	-2.375715000	4.373119000	-1.609725000
H	-4.085734000	3.868972000	-1.621866000
C	-4.304927000	-3.231481000	0.474389000
H	-5.324034000	-2.919657000	0.786039000
H	-3.910291000	-3.914234000	1.238859000
H	-4.410290000	-3.816137000	-0.456102000
H	-0.334566000	0.488816000	1.711976000
Si	1.108361000	-0.361936000	1.353597000
H	1.277459000	-0.452072000	2.881196000
H	1.432994000	-1.819317000	1.089465000
C	3.928636000	2.211596000	-0.430760000
C	4.463849000	2.889978000	0.664055000
C	2.937402000	1.252975000	-0.243234000
H	5.237483000	3.649828000	0.516847000
H	2.504904000	0.736181000	-1.104350000
C	4.007061000	2.593203000	1.946026000
C	2.465463000	0.934135000	1.038402000
H	4.418423000	3.122527000	2.811195000
C	3.024021000	1.619909000	2.124275000
H	2.669932000	1.385512000	3.134253000
H	4.284383000	2.434880000	-1.440832000
C	0.220441000	-1.335726000	-1.182920000
O	1.341138000	-1.632748000	-2.018379000
O	0.425074000	-0.257424000	-0.388864000
H	-5.975445000	-0.234874000	-3.043011000
Si	2.575594000	-2.686212000	-1.682824000
H	2.129927000	-3.732779000	-0.714216000
H	2.951278000	-3.307719000	-2.988413000
C	4.122111000	-1.834917000	-1.042556000
C	4.414088000	-1.714659000	0.322887000
C	5.004412000	-1.240927000	-1.956875000
C	5.532948000	-1.012030000	0.761548000
C	6.124153000	-0.534061000	-1.526919000
C	6.385164000	-0.414942000	-0.163293000
H	3.736068000	-2.156293000	1.060408000
H	4.802568000	-1.323275000	-3.031109000
H	5.724404000	-0.906821000	1.832421000
H	6.790515000	-0.065341000	-2.256406000
H	7.252083000	0.155633000	0.181396000
H	-0.629280000	-1.142855000	-1.861283000
C	-2.154453000	3.626330000	1.287350000
H	-3.142827000	3.338605000	1.672150000
H	-1.458451000	3.695096000	2.137447000
H	-2.215717000	4.612599000	0.797383000
C	-3.475221000	-1.364345000	2.993083000
H	-3.983090000	-2.309405000	3.251421000
H	-2.886548000	-1.023791000	3.858157000
H	-4.220829000	-0.590990000	2.757611000
C	-0.061149000	3.127398000	-0.522957000
H	0.691635000	3.209689000	0.278205000
H	0.359502000	2.485910000	-1.309653000
H	-0.265882000	4.132935000	-0.923180000
C	-1.450342000	-3.053738000	2.042178000
H	-0.744499000	-2.774390000	2.840607000
H	-2.109153000	-3.850002000	2.423807000
H	-0.866575000	-3.434496000	1.191776000
H	-5.414214000	-2.141669000	-1.568873000
H	-4.729204000	1.896687000	-2.886221000

TS6_Co_L11

Co	-1.892107000	0.218683000	0.440413000
H	-0.130658000	-2.345419000	-0.215854000
C	-3.931092000	0.772266000	-1.457018000
C	-5.199298000	0.646419000	-2.015489000
C	-6.072763000	-0.342848000	-1.536068000
C	-5.658652000	-1.211022000	-0.513949000
C	-4.386035000	-1.068945000	0.031576000
C	-3.510784000	-0.058754000	-0.403112000
H	-0.833288000	0.801686000	1.409359000
Si	0.692757000	0.028415000	1.322167000

H	0.830475000	0.273687000	2.846933000
H	1.103771000	-1.433091000	1.410824000
C	3.620407000	2.170279000	-0.835148000
C	4.111611000	3.067987000	0.112661000
C	2.599755000	1.285881000	-0.498779000
H	4.911498000	3.766529000	-0.151841000
H	2.209237000	0.590680000	-1.247542000
C	3.577217000	3.066002000	1.399062000
C	2.052838000	1.257226000	0.793219000
H	3.952683000	3.768310000	2.150077000
C	2.565814000	2.164095000	1.729830000
H	2.158102000	2.156012000	2.746614000
H	4.038035000	2.158959000	-1.846246000
C	0.061299000	-1.551304000	-0.977363000
O	1.257281000	-1.920004000	-1.679888000
O	0.118829000	-0.324218000	-0.427339000
H	-7.075116000	-0.441183000	-1.964256000
Si	2.505564000	-2.837907000	-1.093727000
H	2.040474000	-3.682370000	0.047804000
H	2.964239000	-3.701541000	-2.224582000
C	3.998650000	-1.824790000	-0.576788000
C	4.166840000	-1.341224000	0.728761000
C	4.969554000	-1.489657000	-1.531308000
C	5.253515000	-0.539201000	1.064560000
C	6.058243000	-0.684840000	-1.204403000
C	6.196893000	-0.205206000	0.096301000
H	3.415986000	-1.573533000	1.491356000
H	4.865802000	-1.859573000	-2.557885000
H	5.348556000	-0.150581000	2.081514000
H	6.797090000	-0.424421000	-1.967534000
H	7.040058000	0.441297000	0.355669000
H	-0.755593000	-1.588463000	-1.723652000
C	-3.731710000	-1.953202000	1.048885000
H	-4.434829000	-2.412283000	1.779023000
H	-3.212963000	-2.785010000	0.537876000
C	-2.830717000	1.690523000	-1.898116000
H	-2.208701000	1.183384000	-2.658217000
H	-3.185145000	2.642318000	-2.354162000
N	-2.683135000	-1.184388000	1.768201000
N	-1.935615000	1.974205000	-0.751438000
C	-3.313754000	-0.357077000	2.796655000
H	-3.788790000	-1.000958000	3.571636000
H	-2.557848000	0.282557000	3.270604000
H	-4.077668000	0.282853000	2.339530000
C	-1.758325000	-2.104930000	2.414601000
H	-1.226592000	-2.697357000	1.658436000
H	-1.019636000	-1.540893000	3.001588000
H	-2.295835000	-2.796842000	3.097645000
C	-0.659338000	2.479634000	-1.235135000
H	-0.806652000	3.362974000	-1.891492000
H	-0.028289000	2.776096000	-0.386093000
H	-0.133333000	1.688688000	-1.783635000
C	-2.556892000	2.979410000	0.105675000
H	-3.556213000	2.644442000	0.409645000
H	-1.948011000	3.125488000	1.007217000
H	-2.646276000	3.948859000	-0.435254000
H	-6.339296000	-1.994932000	-0.158410000
H	-5.522194000	1.306837000	-2.830169000

TS6_Fe_L6

Fe	1.31626	0.19364	-0.76370
H	0.12046	-2.40489	0.39149
P	1.40292	2.30119	-0.41508
N	2.36768	2.33494	1.10973
C	2.97931	1.17052	1.41890
N	3.83930	1.11236	2.44633
C	4.46281	-0.04782	2.58675
N	4.37988	-1.09557	1.77877
C	3.50595	-0.96180	0.77229
N	2.70274	0.11775	0.61401
N	3.41274	-1.91472	-0.18341
P	2.39585	-1.42835	-1.59230
C	2.69630	3.51166	1.87487
H	2.57957	3.32080	2.95138
H	2.02671	4.33268	1.59160
H	3.73913	3.83470	1.71008
C	4.31372	-3.03825	-0.14081
H	5.35541	-2.74757	-0.36565
H	3.99422	-3.79024	-0.87373
H	4.31051	-3.49783	0.85766
H	0.34624	0.22560	-2.05501
Si	-1.01284	-0.33732	-1.35277
H	-1.49737	-0.34312	-2.83709
H	-1.28864	-1.81746	-1.17452
C	-3.66505	2.18207	0.79244
C	-4.18034	3.00147	-0.21101
C	-2.72755	1.19932	0.48462
H	-4.90895	3.78109	0.03217
H	-2.30365	0.58139	1.28011
C	-3.76235	2.81406	-1.52723
C	-2.29469	0.98832	-0.83219
H	-4.15968	3.44989	-2.32489
C	-2.84397	1.80899	-1.82685
H	-2.53344	1.65054	-2.86570
H	-3.99302	2.31343	1.82820
C	-0.10520	-1.56951	1.09276
O	-1.21385	-1.95413	1.89828
O	-0.32646	-0.40698	0.43143
H	5.13579	-0.14403	3.44931
Si	-2.47850	-2.91968	1.42138
H	-2.02778	-3.91103	0.39881
H	-2.92180	-3.61841	2.66552
C	-3.96336	-1.96463	0.78788
C	-4.20821	-1.75633	-0.57619
C	-4.83665	-1.37105	1.71071
C	-5.26983	-0.96359	-1.00339
C	-5.89963	-0.57620	1.29174
C	-6.11152	-0.36610	-0.06918
H	-3.53345	-2.19126	-1.32018
H	-4.66895	-1.51976	2.78381
H	-5.41965	-0.78672	-2.07150
H	-6.55556	-0.10414	2.02851
H	-6.92880	0.27898	-0.40339
H	0.74915	-1.46109	1.78635
C	2.30665	3.63263	-1.35640
H	3.30069	3.25545	-1.63469
H	1.73873	3.81144	-2.28311
H	2.40147	4.58774	-0.81080
C	3.74022	-1.43678	-2.89602
H	4.32162	-2.37477	-2.94333
H	3.24935	-1.27497	-3.86876
H	4.41098	-0.58612	-2.70861
C	-0.02690	3.34653	0.13032
H	-0.73720	3.41359	-0.70839
H	-0.54305	2.83137	0.95249
H	0.24795	4.36809	0.43747
C	1.66372	-3.07250	-2.06566
H	2.39155	-3.81985	-2.42162
H	1.09595	-3.47888	-1.21644
H	0.94532	-2.87431	-2.87703

TS6_Fe_L7

Fe	-1.31932	-0.37979	0.53322
H	-1.06097	0.61633	-2.18641
P	0.01450	0.59016	1.80648
N	-0.23600	2.30307	1.40295
C	-1.31837	2.54293	0.62829

N	-1.70003	3.79348	0.34786
C	-2.82606	3.89285	-0.34353
N	-3.62484	2.90063	-0.70409
C	-3.18067	1.67556	-0.39796
N	-1.97755	1.44145	0.18862
N	-3.92772	0.57890	-0.64125
P	-3.24747	-0.93833	0.01915
O	-3.61368	-1.92820	-1.33244
C	-4.51196	-2.96112	-1.04464
H	-3.96454	-3.85839	-0.69639
H	-5.06996	-3.23150	-1.95645
C	-5.42589	-2.42491	0.05129
H	-6.29462	-1.89183	-0.38268
H	-5.81225	-3.22969	0.69800
O	-4.63814	-1.54925	0.81082
O	1.67407	0.54143	1.42852
C	2.46909	0.16511	2.51831
H	3.48894	0.56095	2.37747
H	2.53634	-0.93781	2.58152
C	1.77706	0.75510	3.74080
H	1.98160	0.17803	4.65626
H	2.10114	1.79985	3.91160
O	0.39925	0.71099	3.45942
C	0.60765	3.36575	1.88374
H	0.37394	4.29188	1.34503
H	1.66300	3.10266	1.71086
H	0.46076	3.53863	2.96216
C	-5.24022	0.67497	-1.22689
H	-6.02704	0.59410	-0.45854
H	-5.37397	-0.13571	-1.95814
H	-5.34761	1.64120	-1.73397
H	-0.75463	-1.81168	0.98479
Si	-0.18135	-2.42046	-0.51022
H	-0.83605	-3.70526	0.02144
H	-0.32812	-2.78343	-1.97896
C	3.99964	-2.25697	-0.17678
C	4.25798	-2.98849	0.98134
C	2.68849	-2.09902	-0.62150
H	5.28588	-3.10864	1.33712
H	2.49207	-1.51037	-1.52378
C	3.19926	-3.56220	1.68519
C	1.60969	-2.66407	0.06839
H	3.39365	-4.13613	2.59660
C	1.89279	-3.39889	1.22708
H	1.06246	-3.84676	1.78581
H	4.82045	-1.79543	-0.73238
C	-0.30487	-0.19002	-2.25371
O	0.86928	0.31803	-2.84436
O	-0.04910	-0.68594	-0.99876
H	-3.13691	4.90513	-0.63359
Si	1.67782	1.62524	-2.18832
H	1.75811	2.65155	-3.27269
H	0.96298	2.18533	-1.01035
C	3.44413	1.18859	-1.72724
C	4.29962	0.60316	-2.67150
C	3.96767	1.50514	-0.46804
C	5.63447	0.34516	-2.37202
C	5.30474	1.25651	-0.16411
C	6.14012	0.67772	-1.11570
H	3.90999	0.33818	-3.66073
H	3.30657	1.93163	0.29194
H	6.28386	-0.11754	-3.12019
H	5.69428	1.50505	0.82697
H	7.18734	0.47482	-0.87477
H	-0.68697	-0.98155	-2.92549

TS6_Fe_L9

Fe	1.31528	0.17172	-0.78166
H	0.06322	-2.46148	0.18725
P	1.52973	2.23712	-0.40480
N	2.16941	2.32772	1.26008
C	2.86104	1.14631	1.56809
C	3.74497	1.05340	2.66077
C	4.46574	-0.12665	2.85769
C	4.34518	-1.19212	1.96248
C	3.45583	-1.07829	0.87721
C	2.65415	0.07052	0.67261
N	3.33141	-2.07597	-0.10458
P	2.51265	-1.37112	-1.53264
C	2.54627	3.53677	1.91667
H	2.34816	3.48915	3.00494
H	1.96560	4.38256	1.52256
H	3.62732	3.77990	1.79640
C	4.31672	-3.10591	-0.16889
H	5.35534	-2.71460	-0.27196
H	4.11923	-3.76819	-1.02442
H	4.30211	-3.73937	0.73901
H	0.38111	0.28841	-2.09435
Si	-1.00453	-0.28951	-1.45944
H	-1.52737	-0.14451	-2.93620
H	-1.37036	-1.76441	-1.37803
C	-3.54131	2.21505	0.88234
C	-4.13809	3.03466	-0.07708
C	-2.62060	1.24096	0.50366
H	-4.85557	3.80707	0.22083
H	-2.13336	0.62259	1.26176
C	-3.80781	2.85632	-1.41930
C	-2.27709	1.03365	-0.84339
H	-4.26199	3.49525	-2.18543
C	-2.90264	1.86110	-1.78737
H	-2.65741	1.71495	-2.84577
H	-3.79212	2.34068	1.94088
C	-0.15655	-1.65639	0.92648
O	-1.30116	-2.08321	1.70756
O	-0.36776	-0.46572	0.33578
H	5.15104	-0.20811	3.70985
Si	-2.58491	-2.95799	1.15891
H	-2.21483	-3.86018	0.02633
H	-3.04133	-3.78736	2.32376
C	-4.09557	-1.93639	0.68653
C	-4.33252	-1.48333	-0.61947
C	-5.00869	-1.56073	1.68303
C	-5.42863	-0.67802	-0.91605
C	-6.10618	-0.75203	1.39628
C	-6.31507	-0.30662	0.09255
H	-3.62221	-1.73521	-1.41391
H	-4.84647	-1.89792	2.71367
H	-5.56550	-0.30580	-1.93473
H	-6.79218	-0.45437	2.19543
H	-7.15916	0.35124	-0.13556
H	0.68144	-1.59808	1.64196
C	2.80802	3.34088	-1.24205
H	3.77051	2.80732	-1.19257
H	2.52731	3.44182	-2.30206
H	2.91282	4.34791	-0.79302
C	4.05599	-1.11684	-2.59829
H	4.67915	-2.02162	-2.74231
H	3.72921	-0.73878	-3.57964
H	4.64949	-0.32854	-2.10808
C	0.18658	3.52738	-0.29937
H	-0.24309	3.62588	-1.30947
H	-0.60885	3.15946	0.36540
H	0.52425	4.52420	0.03424
C	1.94787	-2.93862	-2.38792
H	2.75468	-3.62336	-2.70623
H	1.25350	-3.47065	-1.72016

H	1.38306	-2.62395	-3.28062
H	4.94791	-2.09353	2.11178
H	3.88425	1.89032	3.35217

TS6_Fe_L10

Fe	-1.74694	0.29528	0.90532
H	-0.59142	-2.44945	-0.17850
C	-3.69072	1.34192	-0.93715
C	-4.73040	1.20900	-1.84739
C	-5.54279	0.05534	-1.82996
C	-5.27349	-0.96000	-0.89092
C	-4.22974	-0.80917	0.01234
C	-3.39676	0.33955	0.01995
H	-0.69221	0.43637	2.12067
Si	0.61104	-0.21423	1.38899
H	1.28915	-0.12469	2.80373
H	0.81011	-1.72350	1.34335
C	2.77769	2.38267	-1.19354
C	3.50262	3.16655	-0.29510
C	1.94411	1.36601	-0.72730
H	4.15777	3.96582	-0.65949
H	1.35738	0.77055	-1.43011
C	3.38935	2.91623	1.07131
C	1.81460	1.09172	0.64224
H	3.95406	3.52247	1.78846
C	2.56410	1.88654	1.52468
H	2.48748	1.68252	2.59848
H	2.86082	2.56451	-2.27042
C	-0.44938	-1.63289	-0.92414
O	0.59217	-2.03709	-1.81626
O	-0.19414	-0.44529	-0.33079
H	-6.35337	-0.06273	-2.55710
Si	1.98484	-2.81989	-1.34226
H	1.65969	-3.87871	-0.33188
H	2.44800	-3.46422	-2.61771
C	3.40349	-1.79719	-0.71027
C	3.74881	-1.72388	0.65688
C	4.19993	-1.05554	-1.61545
C	4.80714	-0.94340	1.09837
C	5.25748	-0.27022	-1.17796
C	5.57358	-0.20305	0.18354
H	3.13833	-2.25815	1.39269
H	3.95787	-1.07828	-2.68517
H	5.01633	-0.87767	2.17060
H	5.82938	0.32133	-1.90097
H	6.38014	0.44791	0.53382
H	-1.36963	-1.56264	-1.53460
C	-2.83294	2.55312	-0.77883
H	-2.64328	3.09718	-1.72864
H	-3.31733	3.26848	-0.07218
C	-3.89954	-1.74770	1.12042
H	-4.40767	-1.42517	2.06068
H	-4.17432	-2.80479	0.91923
H	-4.93234	2.00253	-2.58148
H	-5.89899	-1.86423	-0.87394
O	-2.49012	-1.67389	1.37605
C	-2.12916	-2.30964	2.56815
H	-2.55024	-1.76805	3.43770
H	-2.49398	-3.35671	2.57378
H	-1.03335	-2.30812	2.63860
O	-1.58405	2.17558	-0.19999
C	-0.87038	3.28388	0.26861
H	0.09707	2.94076	0.65230
H	-0.69702	4.00866	-0.55241
H	-1.42679	3.78478	1.08500

TS6_Ni_L9

Ni	-0.93819	-0.81680	0.58605
H	-0.12231	-1.51473	-3.38241
P	0.37246	0.33318	1.83008
N	-0.27722	1.91976	1.77263
C	-1.55477	1.95429	1.19521
C	-2.30886	3.13518	1.14234
C	-3.56924	3.10105	0.55304
C	-4.09881	1.92421	0.03313
C	-3.33350	0.74995	0.09791
C	-2.04704	0.74816	0.66227
N	-3.81621	-0.47305	-0.39695
P	-2.75575	-1.79024	-0.11143
C	0.22452	3.02967	2.53583
H	0.32120	3.93465	1.91297
H	1.22565	2.79958	2.92229
H	-0.42947	3.27405	3.39291
C	-5.18147	-0.60292	-0.83268
H	-5.89640	-0.32984	-0.03543
H	-5.38915	-1.63863	-1.12965
H	-5.38766	0.03595	-1.70760
H	0.16395	-1.92948	0.64250
Si	0.59420	-2.65490	-0.83024
H	-0.20540	-3.82409	-0.29472
H	0.72677	-3.13772	-2.26482
C	4.67560	-2.01533	-0.32482
C	4.94664	-2.57093	0.92357
C	3.38443	-2.07159	-0.84641
H	5.95871	-2.52784	1.33455
H	3.17050	-1.62686	-1.82437
C	3.92313	-3.18156	1.64843
C	2.34352	-2.67351	-0.13062
H	4.13300	-3.62184	2.62684
C	2.63367	-3.22376	1.12399
H	1.82824	-3.68688	1.70681
H	5.47548	-1.53618	-0.89534
C	-0.26115	-0.66465	-2.68601
O	0.29108	0.46303	-3.29253
O	0.32046	-0.91510	-1.46160
H	-4.16072	4.01903	0.50445
C	0.33813	-0.15359	3.59426
H	-0.70236	-0.16405	3.94661
H	0.75530	-1.16741	3.68858
H	0.93422	0.53292	4.21504
C	-3.56946	-2.84015	1.14828
H	-3.64630	-2.27477	2.08699
H	-4.57069	-3.16800	0.82865
H	-2.94270	-3.72824	1.32214
C	2.15062	0.47742	1.50266
H	2.59496	-0.52232	1.62365
H	2.31488	0.80666	0.46915
H	2.64132	1.17339	2.19852
C	-2.90525	-2.83701	-1.60388
H	-2.22503	-3.69534	-1.49612
H	-3.92693	-3.22108	-1.73567
H	-2.61402	-2.26545	-2.49489
H	-1.92661	4.06985	1.55688
H	-5.09495	1.92839	-0.41261
Si	0.15536	1.97086	-2.58026
H	0.03694	2.94076	-3.70345
H	-1.05510	2.02652	-1.71187
C	1.69666	2.37176	-1.59313
C	1.68065	3.38232	-0.62355
C	2.90963	1.72219	-1.85681
C	2.84174	3.74397	0.05514
C	4.07349	2.07685	-1.17862
C	4.04113	3.09115	-0.22353
H	0.73865	3.89337	-0.39098
H	2.93560	0.91979	-2.60016
H	2.81286	4.53803	0.80649

H	5.01145	1.55942	-1.39425
H	4.95355	3.37250	0.30830
H	-1.35494	-0.49058	-2.55469

TS6_Ni_L10

Ni	0.57617	-1.32230	-0.86221
H	-0.49873	-1.59627	3.16678
C	2.34298	0.76348	-1.43361
C	3.56458	1.43255	-1.36420
C	4.66029	0.79613	-0.77571
C	4.55240	-0.49425	-0.25225
C	3.32383	-1.15158	-0.31474
C	2.23777	-0.51689	-0.90082
H	-0.91457	-1.80252	-0.91892
Si	-1.60904	-2.31437	0.54255
H	-1.73319	-3.62718	-0.21704
H	-1.63829	-2.92872	1.92887
C	-4.67111	0.51971	0.60443
C	-5.40708	0.22092	-0.54107
C	-3.57613	-0.26809	0.95209
H	-6.26399	0.84035	-0.81897
H	-2.99387	-0.02280	1.84651
C	-5.05248	-0.87536	-1.32585
C	-3.19070	-1.36172	0.16587
H	-5.63191	-1.11825	-2.22054
C	-3.95579	-1.65726	-0.96974
H	-3.67445	-2.50928	-1.59938
H	-4.95183	1.37128	1.23013
C	0.07365	-0.98860	2.44023
O	0.22368	0.28523	2.99528
O	-0.55807	-0.92332	1.22130
H	5.61850	1.31855	-0.72176
Si	1.24292	1.42901	2.32965
H	1.76930	2.25327	3.45245
H	2.37036	0.72506	1.65130
C	0.33418	2.57165	1.15125
C	0.94722	3.75174	0.70709
C	-0.97542	2.30533	0.73386
C	0.27659	4.63973	-0.13034
C	-1.65727	3.19752	-0.09119
C	-1.03227	4.36416	-0.52704
H	1.96947	3.98778	1.02701
H	-1.45833	1.37965	1.05651
H	0.77001	5.55571	-0.46510
H	-2.68527	2.97573	-0.39268
H	-1.56736	5.06547	-1.17262
H	1.08469	-1.45231	2.32845
C	3.02209	-2.50146	0.24250
H	3.69649	-3.29028	-0.14093
H	3.08889	-2.51717	1.34903
C	1.08316	1.29374	-2.02910
H	0.80436	2.28690	-1.63188
H	1.14403	1.37299	-3.13197
H	5.42293	-0.97218	0.20732
H	3.66949	2.44595	-1.76363
O	0.04220	0.36506	-1.69346
C	-1.19767	0.62295	-2.32659
H	-1.38706	1.70707	-2.32512
H	-1.18315	0.24953	-3.36351
H	-1.98815	0.11868	-1.75767
O	1.67676	-2.83264	-0.13204
C	1.24518	-4.06354	0.41093
H	1.95347	-4.86153	0.13647
H	1.17892	-3.99838	1.51092
H	0.25780	-4.29724	-0.00357

TS6_Ni_L11

Ni	1.33763	-0.82525	-0.51001
H	-0.21016	-1.26753	3.19869
C	2.48857	1.68957	-0.95829
C	3.50329	2.64331	-0.90814
C	4.70302	2.32779	-0.26188
C	4.89221	1.08309	0.34342
C	3.86620	0.13829	0.28903
C	2.68339	0.44273	-0.37097
H	0.12546	-1.77922	-0.78882
Si	-0.69589	-2.38693	0.56582
H	-0.29286	-3.72184	-0.06250
H	-0.71555	-2.89154	1.99412
C	-4.79184	-1.64168	0.51354
C	-5.15916	-1.88579	-0.80825
C	-3.45954	-1.77398	0.89897
H	-6.20271	-1.77892	-1.11642
H	-3.17494	-1.57268	1.93842
C	-4.19295	-2.27447	-1.73520
C	-2.47232	-2.16213	-0.01537
H	-4.47856	-2.47605	-2.77100
C	-2.86728	-2.42070	-1.33310
H	-2.11829	-2.75445	-2.06177
H	-5.54704	-1.34467	1.24664
C	0.01395	-0.41151	2.53137
O	-0.69994	0.68802	3.03491
O	-0.31643	-0.68029	1.23066
H	5.50515	3.06941	-0.22694
Si	-0.53825	2.19899	2.34687
H	-0.68671	3.19095	3.44555
H	0.82121	2.32648	1.73126
C	-1.83569	2.49979	1.02862
C	-1.73971	3.60820	0.17580
C	-2.90340	1.61406	0.84640
C	-2.66968	3.81658	-0.83934
C	-3.83477	1.81175	-0.17036
C	-3.71740	2.91322	-1.01531
H	-0.91405	4.32045	0.29731
H	-2.98550	0.73500	1.48995
H	-2.57593	4.68377	-1.49813
H	-4.64400	1.09027	-0.31015
H	-4.44400	3.06761	-1.81702
H	1.10366	-0.18266	2.60755
C	3.82975	-1.19989	0.95124
H	4.79131	-1.74954	0.92692
H	3.56567	-1.07849	2.01525
C	1.11516	1.83764	-1.52546
H	0.44354	2.28712	-0.77486
H	1.05259	2.47160	-2.43155
N	2.76164	-2.03547	0.32766
N	0.58133	0.47839	-1.82253
C	3.29233	-2.69760	-0.87352
H	4.08792	-3.41472	-0.59476
H	2.48504	-3.24311	-1.38050
H	3.71076	-1.95067	-1.55954
C	2.35637	-3.06272	1.28989
H	1.87962	-2.59454	2.16071
H	1.65008	-3.76041	0.82253
H	3.23870	-3.63367	1.63275
C	1.10496	0.02128	-3.11651
H	2.20025	0.08369	-3.12401
H	0.80246	-1.01930	-3.29263
H	0.70314	0.65238	-3.93149
C	-0.88233	0.53112	-1.88659
H	-1.20030	1.31813	-2.59407
H	-1.27650	-0.43304	-2.22532
H	-1.28908	0.74744	-0.89350
H	5.83406	0.86225	0.85490
H	3.37145	3.62865	-1.36506

4.5.7. TS7

TS7_Co_L4

Co	-0.867530000	-0.510840000	0.233020000
H	0.804500000	-0.801980000	3.766410000
P	-2.687120000	0.560600000	0.469320000
N	-3.851260000	-0.744799000	0.157440000
C	-3.369340000	-1.998269000	-0.128710000
C	-4.203921000	-3.099399000	-0.382640000
C	-3.598241000	-4.311619000	-0.684630000
C	-2.217831000	-4.430700000	-0.773050000
C	-1.446771000	-3.283180000	-0.525420000
N	-2.032120000	-2.126150000	-0.168470000
N	-0.081211000	-3.254240000	-0.662450000
P	0.723660000	-1.671860000	-0.600330000
N	1.363730000	-1.483240000	-2.166990000
C	2.791950000	-1.218520000	-2.145650000
H	2.989840000	-0.130240000	-2.077870000
H	3.257350000	-1.590340000	-3.074350000
C	3.336820000	-1.936490000	-0.920590000
H	3.693739000	-2.956381000	-1.176840000
H	4.189620000	-1.392401000	-0.484320000
N	2.244600000	-2.004490000	0.028090000
N	-3.131470000	1.863531000	-0.503320000
C	-3.893860000	2.901441000	0.151320000
H	-4.973700000	2.829351000	-0.096380000
H	-3.548579000	3.890391000	-0.196550000
C	-3.653400000	2.719381000	1.642380000
H	-2.791040000	3.327950000	1.982620000
H	-4.530440000	3.026801000	2.238260000
N	-3.393290000	1.308371000	1.831860000
C	6.608960000	0.092049000	-0.485920000
C	6.509510000	-0.446311000	0.794310000
C	5.660400000	1.015669000	-0.925540000
H	7.253400000	-1.165111000	1.147140000
H	5.738670000	1.446569000	-1.927200000
C	5.456750000	-0.064241000	1.625510000
C	4.615780000	1.393959000	-0.087110000
H	5.391660000	-0.490661000	2.632960000
H	3.875610000	2.117619000	-0.446180000
C	4.489170000	0.856859000	1.203560000
H	7.431490000	-0.202971000	-1.142710000
Si	3.077720000	1.370870000	2.342060000
H	3.408600000	0.819910000	3.695900000
H	3.062280000	2.867090000	2.386770000
O	1.606340000	0.799870000	1.891490000
C	0.088250000	-0.414800000	3.012370000
H	-0.322570000	0.595170000	3.188250000
O	-0.322500000	-1.144640000	2.122200000
C	-0.640359000	3.918470000	-0.364840000
C	0.208670000	2.861030000	-0.711170000
C	-0.910189000	4.966140000	-1.243440000
H	-1.574049000	5.782560000	-0.941260000
C	0.812520000	2.921430000	-1.973570000
C	-0.323829000	4.980050000	-2.508890000
H	1.518440000	2.129220000	-2.254160000
H	-0.530849000	5.799180000	-3.203250000
C	0.548161000	3.954150000	-2.871170000
H	1.030311000	3.968490000	-3.852990000
H	-1.091999000	3.918290000	0.634580000
Si	0.524170000	1.341090000	0.471960000
H	-0.949560000	0.096910000	-1.102910000
H	1.709300000	0.913480000	-0.415010000
H	-0.325330000	2.024690000	1.587610000
H	-4.222991000	-5.187819000	-0.875370000
C	-5.271970000	-0.501269000	0.152030000
H	-5.797440000	-1.124169000	0.895000000
H	-5.457260000	0.545571000	0.414840000
H	-5.714150000	-0.694749000	-0.840190000
C	0.627019000	-4.436510000	-1.084730000
H	0.310819000	-4.776780000	-2.085520000
H	1.699199000	-4.215210000	-1.127560000
H	0.481859000	-5.263930000	-0.371130000
C	-3.129690000	0.852931000	3.167550000
H	-2.305940000	1.417420000	3.646460000
H	-4.027150000	0.967411000	3.798140000
H	-2.863120000	-0.214270000	3.156620000
C	-3.059810000	1.870241000	-1.936560000
H	-4.056460000	1.722081000	-2.396590000
H	-2.643470000	2.826180000	-2.296070000
H	-2.397810000	1.065530000	-2.285600000
C	2.502690000	-2.507930000	1.345480000
H	2.996269000	-3.497730000	1.297170000
H	3.161170000	-1.828550000	1.914840000
H	1.562230000	-2.621580000	1.899130000
C	0.594290000	-0.847440000	-3.205360000
H	-0.450960000	-1.186840000	-3.167250000
H	0.601060000	0.256650000	-3.128440000
H	1.003080000	-1.129930000	-4.188280000
H	-5.288121000	-3.008849000	-0.344290000
H	-1.752691000	-5.377320000	-1.042770000

TS7_Co_L5

Co	0.412130000	0.226201000	-0.361229000
H	-1.240950000	-0.616400000	-3.806319000
P	1.545419000	2.020391000	-0.566519000
N	3.213610000	1.347822000	-0.414869000
C	3.328220000	0.020612000	-0.197419000
N	4.533570000	-0.548948000	-0.019209000
C	4.485361000	-1.842608000	0.223031000
N	3.422281000	-2.611898000	0.376551000
C	2.256281000	-1.970999000	0.209431000
N	2.187980000	-0.681319000	-0.155849000
N	1.085861000	-2.600189000	0.439751000
P	-0.401139000	-1.583729000	0.456151000
N	-0.963189000	-1.784240000	2.043411000
C	-2.331889000	-2.273620000	2.080981000
H	-3.049040000	-1.429220000	2.085421000
H	-2.494449000	-2.862430000	2.999221000
C	-2.512039000	-3.121600000	0.831991000
H	-2.284609000	-4.189050000	1.032191000
H	-3.546849000	-3.071090000	0.458241000
N	-1.598169000	-2.581220000	-0.155659000
N	1.475249000	3.307501000	0.510041000
C	1.726409000	4.609201000	-0.069029000
H	2.763138000	4.947371000	0.133021000
H	1.047388000	5.349961000	0.386551000
C	1.469699000	4.461071000	-1.561369000
H	0.419499000	4.712871000	-1.811999000
H	2.118808000	5.127531000	-2.155199000
N	1.751089000	3.076111000	-1.881539000
C	-6.368149000	-2.927321000	0.622251000
C	-6.086019000	-3.260141000	-0.699999000
C	-5.968399000	-1.689511000	1.126491000
H	-6.403519000	-4.225031000	-1.103109000
H	-6.195060000	-1.421081000	2.161691000
C	-5.398689000	-2.356031000	-1.509289000
C	-5.286190000	-0.792221000	0.309851000
H	-5.189669000	-2.625641000	-2.550769000
H	-4.976670000	0.176279000	0.718811000
C	-4.982170000	-1.109581000	-1.023039000
H	-6.907919000	-3.630582000	1.261661000
Si	-4.076600000	0.109529000	-2.137619000

H	-4.194080000	-0.425881000	-3.531739000
H	-4.781710000	1.424709000	-2.027249000
O	-2.480810000	0.294430000	-1.787059000
C	-0.721250000	0.024760000	-3.065259000
H	-0.829890000	1.116280000	-3.192909000
O	0.075400000	-0.468219000	-2.276219000
C	-1.647261000	4.014490000	0.594281000
C	-1.962181000	2.685640000	0.901341000
C	-1.781062000	5.039070000	1.528761000
H	-1.532302000	6.070010000	1.257391000
C	-2.462911000	2.433030000	2.184611000
C	-2.247051000	4.751680000	2.811941000
H	-2.764420000	1.408340000	2.437851000
H	-2.356842000	5.551190000	3.549811000
C	-2.597351000	3.442080000	3.136981000
H	-2.986731000	3.212270000	4.132921000
H	-1.296691000	4.247600000	-0.418569000
Si	-1.684670000	1.240240000	-0.376919000
H	0.308750000	0.721681000	1.018591000
H	-2.482130000	0.282820000	0.527301000
H	-1.342391000	2.277310000	-1.484049000
C	4.375929000	2.209692000	-0.409029000
H	4.189769000	3.056992000	-1.081829000
H	4.587109000	2.594032000	0.601911000
H	5.257220000	1.660032000	-0.759519000
C	1.084981000	-3.985669000	0.858901000
H	1.538111000	-4.109069000	1.853601000
H	0.049391000	-4.341849000	0.895371000
H	1.651661000	-4.602029000	0.147851000
C	1.547779000	2.651391000	-3.239639000
H	0.516509000	2.855091000	-3.587289000
H	2.243969000	3.176601000	-3.913629000
H	1.744140000	1.573531000	-3.331689000
C	1.467839000	3.186841000	1.941471000
H	2.450969000	3.447321000	2.377401000
H	0.700739000	3.848871000	2.376461000
H	1.223689000	2.156201000	2.233351000
C	-1.631659000	-3.103730000	-1.492309000
H	-1.494979000	-4.201990000	-1.491699000
H	-2.596569000	-2.880980000	-1.978789000
H	-0.829599000	-2.657480000	-2.093519000
C	-0.549410000	-0.899240000	3.102381000
H	0.526170000	-0.685309000	3.024231000
H	-1.095290000	0.062880000	3.092761000
H	-0.721660000	-1.388620000	4.073421000
C	5.815821000	-2.575578000	0.385241000
F	5.890491000	-3.591808000	-0.472489000
F	6.856331000	-1.785077000	0.176981000
F	5.915461000	-3.073498000	1.617921000

TS7_Co_L6

Co	-0.830659000	-0.277640000	0.161349000
H	0.813071000	-0.775761000	3.727019000
P	-2.694339000	0.727070000	0.540699000
N	-3.821299000	-0.653090000	0.516899000
C	-3.314139000	-1.849260000	0.117559000
N	-4.105559000	-2.921260000	-0.017511000
C	-3.495010000	-4.004260000	-0.473991000
N	-2.223170000	-4.124560000	-0.825701000
C	-1.495379000	-3.013480000	-0.651991000
N	-2.000309000	-1.881500000	-0.141391000
N	-0.188179000	-2.995220000	-1.008541000
P	0.721991000	-1.501011000	-0.689331000
C	6.080591000	-1.309401000	-0.887451000
C	6.094121000	-1.391451000	0.502719000
C	5.300391000	-0.340091000	-1.517771000
H	6.711171000	-2.143661000	1.001159000
H	5.296451000	-0.265261000	-2.608701000
C	5.320061000	-0.509971000	1.256029000
C	4.531161000	0.535379000	-0.756611000
H	5.336921000	-0.584751000	2.349139000
H	3.914821000	1.289269000	-1.259561000
C	4.514651000	0.460789000	0.644929000
H	6.686191000	-1.997611000	-1.483291000
Si	3.366011000	1.547479000	1.675089000
H	3.880761000	1.477669000	3.077859000
H	3.463201000	2.948689000	1.161419000
O	1.807341000	1.023419000	1.638139000
C	0.164941000	-0.293220000	2.965979000
H	-0.079909000	0.776110000	3.107169000
O	-0.289799000	-0.951020000	2.049359000
C	-0.838489000	4.198360000	0.077689000
C	-0.184419000	3.138200000	-0.559621000
C	-1.423508000	5.243180000	-0.640741000
H	-1.919598000	6.064120000	-0.114571000
C	-0.113609000	3.180790000	-1.958881000
C	-1.372538000	5.238460000	-2.032611000
H	0.420031000	2.377949000	-2.484421000
H	-1.834568000	6.048320000	-2.604101000
C	-0.711659000	4.200380000	-2.693391000
H	-0.660579000	4.195870000	-3.786211000
H	-0.890019000	4.201730000	1.174379000
Si	0.522041000	1.609319000	0.429769000
H	-0.958669000	0.273060000	-1.196931000
H	1.612321000	1.419789000	-0.648001000
H	-0.177489000	2.151460000	1.709059000
H	-4.115320000	-4.903240000	-0.579681000
C	-5.221779000	-0.512760000	0.857619000
H	-5.353989000	-0.345250000	1.937829000
H	-5.668869000	0.334250000	0.317249000
H	-5.756949000	-1.426740000	0.578249000
C	0.408910000	-4.200891000	-1.549101000
H	-0.205890000	-4.604060000	-2.364501000
H	1.406160000	-3.970761000	-1.941821000
H	0.504500000	-4.982561000	-0.779701000
C	-3.445319000	1.850690000	-0.679951000
H	-2.856049000	2.779470000	-0.693141000
H	-3.379539000	1.387820000	-1.673871000
H	-4.490149000	2.093130000	-0.439991000
C	1.467191000	-1.118501000	-2.304061000
H	0.668231000	-0.958181000	-3.040151000
H	2.020121000	-0.175701000	-2.174511000
H	2.163531000	-1.894901000	-2.650861000
C	2.121661000	-2.140601000	0.283129000
H	2.708811000	-2.888821000	-0.268241000
H	2.773251000	-1.291111000	0.527219000
H	1.739581000	-2.570791000	1.218289000
C	-3.122679000	1.516220000	2.130199000
H	-2.421589000	2.352560000	2.270169000
H	-4.152909000	1.899260000	2.147199000
H	-2.978379000	0.797730000	2.949469000

TS7_Fe_L3

Fe	-0.908970000	-0.779330000	0.476950000
H	0.472780000	-0.472100000	4.204550000
P	-2.848510000	0.083569000	0.432890000
C	-3.940640000	-1.395111000	-0.031140000
C	-3.113580000	-2.587561000	-0.355240000
N	-3.655470000	-3.600941000	-1.028420000
C	-2.846309000	-4.620261000	-1.287920000
N	-1.565429000	-4.698811000	-0.949040000
C	-1.079050000	-3.649990000	-0.288940000
N	-1.822840000	-2.562991000	0.041560000
C	0.353630000	-3.559800000	0.043550000
P	0.846290000	-1.736560000	-0.280590000
N	1.348750000	-1.753510000	-1.932870000

C	2.790140000	-1.679760000	-2.070190000
H	3.108280000	-0.629009000	-2.232360000
H	3.132690000	-2.272409000	-2.938750000
C	3.374630000	-2.195159000	-0.770980000
H	3.435940000	-3.308809000	-0.779650000
H	4.393650000	-1.807299000	-0.602890000
N	2.479920000	-1.717000000	0.253490000
N	-3.303491000	1.342959000	-0.650740000
C	-4.485571000	2.048359000	-0.232680000
H	-5.419261000	1.545189000	-0.580400000
H	-4.484181000	3.067089000	-0.660560000
C	-4.425651000	2.074959000	1.280300000
H	-3.773871000	2.908659000	1.622160000
H	-5.426081000	2.233789000	1.727450000
N	-3.880281000	0.798119000	1.666350000
C	6.527100000	0.019511000	-0.593420000
C	6.423970000	0.001361000	0.795220000
C	5.538079000	0.649381000	-1.349430000
H	7.194360000	-0.489889000	1.396820000
H	5.610899000	0.664881000	-2.441120000
C	5.336919000	0.617181000	1.416580000
C	4.453879000	1.252451000	-0.717660000
H	5.276919000	0.614431000	2.511510000
H	3.673699000	1.731731000	-1.319260000
C	4.327939000	1.251331000	0.679440000
H	7.378420000	-0.456138000	-1.089100000
Si	2.928789000	2.190780000	1.554510000
H	3.517859000	2.501781000	2.901000000
H	2.778949000	3.463740000	0.778310000
O	1.496459000	1.447250000	1.784790000
C	-0.193870000	-0.213710000	3.355430000
H	-0.749891000	0.742810000	3.393350000
O	-0.244990000	-0.949960000	2.382260000
C	-1.330281000	3.749039000	-0.189850000
C	-0.329891000	2.850500000	-0.585070000
C	-1.769162000	4.782729000	-1.014190000
H	-2.556862000	5.462939000	-0.671460000
C	0.237249000	3.065320000	-1.848630000
C	-1.199942000	4.957170000	-2.277160000
H	1.041289000	2.393030000	-2.174910000
H	-1.536412000	5.769829000	-2.928910000
C	-0.186631000	4.093720000	-2.690550000
H	0.277749000	4.226630000	-3.673390000
H	-1.774461000	3.618629000	0.804500000
Si	0.202309000	1.327750000	0.526730000
H	-1.199430000	-0.389540000	-0.972100000
H	1.358209000	1.030550000	-0.511060000
H	-0.765711000	1.838150000	1.680550000
H	-3.273019000	-5.471951000	-1.832550000
C	-3.518211000	0.667039000	3.047700000
H	-4.415971000	0.705459000	3.691360000
H	-3.025860000	-0.302151000	3.220680000
H	-2.825951000	1.469159000	3.381540000
C	2.815910000	-2.013830000	1.613660000
H	2.072330000	-1.569940000	2.287690000
H	2.867580000	-3.107740000	1.813210000
H	3.800560000	-1.582149000	1.859190000
C	0.611160000	-1.053580000	-2.952390000
H	0.909290000	-1.435400000	-3.943660000
H	-0.467460000	-1.218620000	-2.832550000
H	0.793160000	0.040050000	-2.927060000
C	-3.052501000	1.307079000	-2.059850000
H	-2.074411000	0.851889000	-2.260900000
H	-3.828031000	0.739189000	-2.620010000
H	-3.026441000	2.336429000	-2.456490000
H	-4.665260000	-1.203121000	-0.839510000
H	0.942571000	-4.339750000	-0.458150000
H	0.481930000	-3.631990000	1.139250000
H	-4.535180000	-1.613301000	0.872920000

TS7_Fe_L4

Fe	-0.857200000	-0.588490000	0.310850000
H	0.644110000	-0.566891000	4.001690000
P	-2.690090000	0.428970000	0.437550000
N	-3.837390000	-0.921989000	0.095900000
C	-3.299740000	-2.157120000	-0.132010000
C	-4.090511000	-3.286309000	-0.422990000
C	-3.446471000	-4.487269000	-0.685100000
C	-2.060091000	-4.560650000	-0.711150000
C	-1.329441000	-3.390640000	-0.431470000
N	-1.950830000	-2.241370000	-0.079220000
N	0.033739000	-3.330171000	-0.533130000
P	0.761460000	-1.682881000	-0.475930000
N	1.463130000	-1.552351000	-2.049090000
C	2.887400000	-1.292141000	-1.991230000
H	3.082720000	-0.203041000	-1.910640000
H	3.382910000	-1.660052000	-2.908780000
C	3.397010000	-2.008182000	-0.753700000
H	3.709029000	-3.050732000	-0.993690000
H	4.278080000	-1.501032000	-0.329600000
N	2.302580000	-1.985951000	0.181330000
N	-3.183479000	1.696110000	-0.595940000
C	-4.053569000	2.690451000	-0.029480000
H	-5.110309000	2.543071000	-0.349450000
H	-3.753259000	3.695011000	-0.380350000
C	-3.912519000	2.564091000	1.478600000
H	-3.114499000	3.237960000	1.857410000
H	-4.849849000	2.840291000	1.999170000
N	-3.581779000	1.184551000	1.726510000
C	6.733160000	-0.298863000	-0.232730000
C	6.408710000	-0.473902000	1.110970000
C	5.966030000	0.559128000	-1.020670000
H	7.003940000	-1.145903000	1.735970000
H	6.211911000	0.698108000	-2.077610000
C	5.324900000	0.213028000	1.656840000
C	4.881521000	1.233918000	-0.464540000
H	5.090730000	0.082268000	2.720180000
H	4.278801000	1.899448000	-1.092460000
C	4.537081000	1.079608000	0.886110000
H	7.583470000	-0.831983000	-0.667620000
Si	3.149001000	2.104219000	1.679190000
H	3.680631000	2.384808000	3.056430000
H	3.129161000	3.379999000	0.892720000
O	1.670021000	1.442839000	1.837780000
C	-0.076380000	-0.224641000	3.229930000
H	-0.620849000	0.721500000	3.410740000
O	-0.191030000	-0.850220000	2.187260000
C	-0.860119000	3.944480000	-0.276860000
C	0.056651000	2.948909000	-0.640310000
C	-1.201718000	4.991480000	-1.132030000
H	-1.923268000	5.751040000	-0.810240000
C	0.643711000	3.078289000	-1.907740000
C	-0.621138000	5.076480000	-2.397730000
H	1.391341000	2.332369000	-2.207910000
H	-0.883068000	5.896260000	-3.074270000
C	0.311912000	4.112659000	-2.781420000
H	0.786402000	4.173889000	-3.766550000
H	-1.314939000	3.884620000	0.719390000
Si	0.436461000	1.408469000	0.509680000
H	-1.045510000	-0.115610000	-1.130620000
H	1.646711000	1.043799000	-0.453380000
H	-0.534969000	1.999180000	1.628580000
H	-4.038521000	-5.381649000	-0.900080000
C	-3.338869000	0.816400000	3.088330000
H	-4.273089000	0.852981000	3.678720000
H	-2.950750000	-0.212420000	3.137400000
H	-2.603219000	1.486620000	3.578530000
C	2.524800000	-2.444051000	1.517220000
H	1.589500000	-2.394811000	2.089790000

H	2.888679000	-3.493841000	1.534720000
H	3.276600000	-1.819612000	2.032940000
C	0.752510000	-0.867641000	-3.096620000
H	1.182280000	-1.147811000	-4.073670000
H	-0.307460000	-1.157150000	-3.092100000
H	0.801420000	0.234229000	-2.993130000
C	-3.028509000	1.694090000	-2.018300000
H	-2.284709000	0.940830000	-2.311660000
H	-3.984479000	1.464621000	-2.535670000
H	-2.670229000	2.678230000	-2.368740000
C	-5.255730000	-0.725089000	0.000480000
H	-5.634850000	-0.921349000	-1.019700000
H	-5.813170000	-1.372319000	0.701370000
H	-5.488740000	0.312221000	0.265130000
C	0.778279000	-4.486681000	-0.943500000
H	0.636329000	-5.328801000	-0.243470000
H	0.502589000	-4.831641000	-1.957330000
H	1.845499000	-4.237941000	-0.954100000
H	-5.177491000	-3.217749000	-0.443720000
H	-1.552371000	-5.490990000	-0.963180000

TS7_Fe_L11

Fe	-1.334510000	0.054980000	0.005590000
H	0.227509000	-1.471440000	3.360520000
C	-4.211880000	-0.010770000	-0.260380000
C	-5.454231000	-0.604469000	-0.468790000
C	-5.524821000	-1.969999000	-0.791770000
C	-4.343901000	-2.724040000	-0.891820000
C	-3.113001000	-2.117460000	-0.649550000
C	-3.018341000	-0.748780000	-0.336670000
C	4.362879000	-3.980190000	-0.812060000
C	3.865909000	-3.997850000	0.488870000
C	4.547129000	-2.753960000	-1.458960000
H	3.705569000	-4.952910000	1.000430000
H	4.927949000	-2.729760000	-2.485480000
C	3.567569000	-2.798270000	1.136770000
C	4.221009000	-1.567050000	-0.809940000
H	3.185629000	-2.823280000	2.165280000
H	4.322629000	-0.610770000	-1.336280000
C	3.723379000	-1.557740000	0.501940000
H	4.594029000	-4.917700000	-1.328450000
Si	3.318160000	0.059660000	1.423120000
H	4.025640000	-0.217580000	2.743540000
H	4.127570000	1.127960000	0.749490000
O	1.793010000	0.441800000	1.884500000
C	-0.342771000	-0.661690000	2.856540000
H	-0.389130000	0.315340000	3.378350000
O	-0.850041000	-0.846300000	1.755930000
C	1.635180000	4.023050000	0.296150000
C	1.542370000	2.771480000	-0.334110000
C	2.067050000	5.169010000	-0.379440000
H	2.131990000	6.127280000	0.150820000
C	1.918390000	2.730800000	-1.688790000
C	2.421210000	5.096760000	-1.724840000
H	1.865760000	1.763420000	-2.204910000
H	2.763750000	5.989910000	-2.260240000
C	2.338970000	3.861950000	-2.380620000
H	2.606950000	3.789360000	-3.441520000
H	1.353160000	4.088720000	1.355050000
Si	0.794560000	1.175340000	0.523400000
H	-1.387110000	0.783610000	-1.388520000
H	1.934210000	0.324260000	-0.380020000
H	0.198550000	2.081770000	1.714750000
H	-6.496911000	-2.444319000	-0.968210000
H	-6.378590000	-0.013759000	-0.398810000
H	-4.401991000	-3.791350000	-1.148240000
C	-3.944290000	1.449120000	-0.059190000
H	-3.796960000	1.909640000	-1.052900000

H	-4.777280000	1.999540000	0.438280000
C	-1.777881000	-2.804770000	-0.620000000
H	-1.588251000	-3.189670000	0.397730000
H	-1.713811000	-3.679270000	-1.309190000
N	-2.681330000	1.665990000	0.676370000
N	-0.695681000	-1.844400000	-0.911660000
C	-2.190080000	3.007220000	0.402700000
H	-1.281600000	3.190940000	0.992770000
H	-1.933530000	3.085740000	-0.662320000
H	-2.959300000	3.770480000	0.662220000
C	-2.889930000	1.495550000	2.102740000
H	-1.934800000	1.645920000	2.624620000
H	-3.629760000	2.234240000	2.487880000
H	-3.259840000	0.480360000	2.304140000
C	0.577049000	-2.361260000	-0.436660000
H	0.818969000	-3.338180000	-0.911620000
H	1.369929000	-1.638410000	-0.665920000
H	0.542449000	-2.489930000	0.653040000
C	-0.610151000	-1.611920000	-2.344510000
H	-1.579641000	-1.268140000	-2.724770000
H	0.131239000	-0.826470000	-2.539760000
H	-0.311361000	-2.548480000	-2.870480000

TS7_Ni_L2

Ni	-0.538509000	0.343119000	-0.004081000
H	1.935661000	-1.029540000	-2.403201000
P	-2.566869000	0.213549000	-0.899941000
O	-2.674730000	1.830819000	-1.606971000
C	-1.858750000	2.767949000	-1.174711000
N	-2.088840000	4.040479000	-1.454851000
C	-1.228040000	4.887209000	-0.896971000
N	-0.220510000	4.587229000	-0.078901000
C	-0.068500000	3.292510000	0.133519000
N	-0.832460000	2.363879000	-0.430461000
O	0.869910000	2.858990000	0.955789000
P	1.129690000	1.136580000	1.146479000
N	1.433780000	0.975740000	2.766359000
C	2.856890000	0.856320000	3.067199000
H	3.110031000	-0.204540000	3.260649000
H	3.104770000	1.433250000	3.971579000
C	3.577370000	1.386950000	1.842999000
H	3.685940000	2.488230000	1.893919000
H	4.580250000	0.952270000	1.719919000
N	2.738150000	1.013100000	0.705159000
N	-4.004889000	0.116989000	-0.060451000
C	-5.098069000	-0.243251000	-0.962351000
H	-5.536460000	0.669279000	-1.411161000
H	-5.889739000	-0.756961000	-0.395851000
C	-4.486949000	-1.136731000	-2.031051000
H	-4.546029000	-2.203831000	-1.749491000
H	-4.995019000	-1.014781000	-3.000891000
N	-3.091879000	-0.731401000	-2.148161000
C	6.812471000	-0.395800000	-1.230261000
C	6.063601000	-0.851720000	-2.313431000
C	6.368891000	-0.619730000	0.072049000
H	6.419051000	-0.688560000	-3.332861000
H	6.967261000	-0.278300000	0.920299000
C	4.866971000	-1.527180000	-2.092681000
C	5.167541000	-1.287110000	0.288219000
H	4.299481000	-1.900710000	-2.951561000
H	4.829951000	-1.469700000	1.314299000
C	4.395311000	-1.744770000	-0.789681000
H	7.756001000	0.127091000	-1.401711000
Si	2.827281000	-2.672260000	-0.460791000
H	2.528781000	-3.671820000	-1.518511000
H	2.802801000	-3.228220000	0.912939000
O	1.450701000	-1.596810000	-0.468721000
C	1.000531000	-1.109710000	-1.803561000

H	0.395771000	-1.959640000	-2.211661000
O	0.360591000	0.033290000	-1.681281000
C	-2.653089000	-3.133121000	0.355679000
C	-1.863889000	-2.300241000	1.159069000
C	-3.974769000	-3.409571000	0.697509000
H	-4.576179000	-4.067791000	0.064979000
C	-2.427649000	-1.762051000	2.323259000
C	-4.517779000	-2.872841000	1.861749000
H	-1.828169000	-1.118011000	2.974079000
H	-5.547389000	-3.105751000	2.143439000
C	-3.737349000	-2.057561000	2.681349000
H	-4.152529000	-1.657191000	3.609469000
H	-2.221969000	-3.597861000	-0.538121000
Si	-0.035079000	-2.125060000	0.730029000
H	-1.195339000	0.495309000	1.299199000
H	0.763941000	-1.888460000	1.961159000
H	0.155991000	-3.480400000	0.118009000
H	-1.371090000	5.949359000	-1.124311000
C	-2.277769000	-1.262201000	-3.213221000
H	-1.296729000	-0.771141000	-3.209151000
H	-2.141209000	-2.353651000	-3.114271000
H	-2.752919000	-1.066011000	-4.186581000
C	-4.312600000	1.056219000	0.992659000
H	-4.687500000	2.015979000	0.590809000
H	-5.079750000	0.630709000	1.655549000
H	-3.416310000	1.254139000	1.598289000
C	3.155650000	1.504490000	-0.596951000
H	4.102600000	1.027690000	-0.883761000
H	2.392120000	1.260260000	-1.348901000
H	3.299320000	2.599430000	-0.581421000
C	0.476360000	0.701900000	3.805979000
H	-0.546110000	0.784709000	3.414379000
H	0.614501000	-0.311940000	4.220719000
H	0.577790000	1.428150000	4.627009000

TS7_Ni_L4

Ni	-0.603241000	0.257970000	-0.077150000
H	1.692240000	-0.879359000	-2.831390000
P	-2.530781000	-0.196751000	-0.982670000
N	-2.972861000	1.399729000	-1.574720000
C	-2.289282000	2.515969000	-1.127320000
C	-2.691462000	3.831499000	-1.398030000
C	-1.928752000	4.855379000	-0.848600000
C	-0.829362000	4.595830000	-0.038530000
C	-0.503252000	3.251280000	0.188870000
N	-1.210462000	2.276950000	-0.381830000
N	0.527568000	2.844890000	1.017780000
P	0.928659000	1.139650000	1.175350000
N	1.174389000	0.916800000	2.823290000
C	2.556449000	0.698961000	3.213600000
H	2.721039000	-0.357789000	3.496660000
H	2.798869000	1.310801000	4.099730000
C	3.420549000	1.097191000	2.019100000
H	3.806318000	2.131121000	2.125970000
H	4.301039000	0.443492000	1.915390000
N	2.579639000	0.983201000	0.838640000
N	-3.846201000	-0.826761000	-0.146340000
C	-4.749080000	-1.628012000	-0.948070000
H	-5.643560000	-1.042942000	-1.240990000
H	-5.113250000	-2.483382000	-0.354240000
C	-3.953830000	-2.090461000	-2.170180000
H	-3.626630000	-3.140161000	-2.064460000
H	-4.569190000	-2.042162000	-3.085940000
N	-2.813550000	-1.199331000	-2.305070000
C	6.766029000	-0.008908000	-1.241700000
C	6.073539000	-0.162098000	-2.441190000
C	6.309829000	-0.635178000	-0.082690000
H	6.438409000	0.320912000	-3.350000000
H	6.862389000	-0.524708000	0.853400000
C	4.919420000	-0.939238000	-2.480270000
C	5.155680000	-1.410418000	-0.124770000
H	4.389200000	-1.063138000	-3.430170000
H	4.808220000	-1.909888000	0.786840000
C	4.441980000	-1.568948000	-1.322170000
H	7.674849000	0.596333000	-1.211180000
Si	2.924920000	-2.625399000	-1.334120000
H	2.603400000	-3.174799000	-2.675180000
H	2.987810000	-3.673479000	-0.289730000
O	1.521190000	-1.670699000	-0.900290000
C	0.855110000	-0.946280000	-2.099650000
H	0.128350000	-1.721860000	-2.445650000
O	0.380439000	0.200820000	-1.741210000
C	-2.199210000	-3.118521000	1.190740000
C	-1.046350000	-2.425700000	1.577850000
C	-3.230830000	-3.341941000	2.098940000
H	-4.117019000	-3.904501000	1.793260000
C	-0.948770000	-1.965220000	2.898520000
C	-3.126520000	-2.864751000	3.403060000
H	-0.039620000	-1.446970000	3.218830000
H	-3.933530000	-3.044751000	4.117120000
C	-1.980320000	-2.176371000	3.804160000
H	-1.886710000	-1.821921000	4.833860000
H	-2.279250000	-3.515541000	0.173040000
Si	0.462450000	-2.398180000	0.467590000
H	-1.332201000	0.249060000	1.192980000
H	1.611170000	-2.384129000	1.414350000
H	0.254790000	-3.644880000	-0.333360000
H	-2.208413000	5.892229000	-1.048210000
C	1.282308000	3.842190000	1.745030000
H	1.930208000	3.351171000	2.478980000
H	1.914028000	4.452021000	1.078480000
H	0.610558000	4.513420000	2.302540000
C	-4.179521000	1.574539000	-2.353030000
H	-4.982342000	2.056168000	-1.769670000
H	-3.985222000	2.182409000	-3.250140000
H	-4.540291000	0.598268000	-2.694940000
C	-2.202860000	-1.017121000	-3.596860000
H	-1.427081000	-0.241490000	-3.538490000
H	-1.738960000	-1.947311000	-3.964910000
H	-2.949451000	-0.697711000	-4.346430000
C	-4.358281000	-0.209571000	1.046810000
H	-5.172071000	0.506538000	0.823710000
H	-4.746030000	-0.973332000	1.738960000
H	-3.554271000	0.333199000	1.565670000
C	3.098069000	1.530081000	-0.396840000
H	4.039529000	1.032491000	-0.663120000
H	2.372109000	1.370251000	-1.206200000
H	3.297018000	2.615551000	-0.309460000
C	0.167889000	1.143770000	3.824060000
H	-0.834241000	1.099390000	3.372570000
H	0.214079000	0.374210000	4.611810000
H	0.282488000	2.130230000	4.309820000
H	-3.566532000	4.052669000	-2.006620000
H	-0.250543000	5.410700000	0.392810000

TS7_Ni_L6

Ni	-0.704300000	0.235540000	0.189651000
H	1.874170000	-0.581760000	-2.392789000
P	-2.530890000	0.193511000	-1.059099000
N	-2.706210000	1.899721000	-1.428349000
C	-1.913529000	2.791301000	-0.770369000
N	-2.042559000	4.100101000	-0.996969000
C	-1.211609000	4.859971000	-0.294359000
N	-0.292759000	4.454800000	0.574991000
C	-0.237749000	3.132190000	0.737091000
N	-1.038240000	2.286760000	0.095561000

N	0.662990000	2.578430000	1.600881000
P	0.782650000	0.832220000	1.684191000
C	6.495980000	0.210818000	-0.592799000
C	5.730170000	0.242939000	-1.757129000
C	6.177550000	-0.679102000	0.430631000
H	5.991530000	0.928369000	-2.565959000
H	6.789550000	-0.717922000	1.334351000
C	4.639400000	-0.609311000	-1.893139000
C	5.080909000	-1.524481000	0.293641000
H	4.060610000	-0.583501000	-2.821949000
H	4.841669000	-2.223231000	1.102921000
C	4.286159000	-1.494311000	-0.862679000
H	7.356970000	0.875308000	-0.489359000
Si	2.786939000	-2.581761000	-0.939919000
H	2.531839000	-3.198161000	-2.266249000
H	2.795049000	-3.551511000	0.180601000
O	1.375399000	-1.591760000	-0.631139000
C	0.945580000	-0.693680000	-1.791739000
H	0.240839000	-1.357910000	-2.354649000
O	0.466690000	0.427070000	-1.341899000
C	-2.537441000	-3.547989000	-0.199439000
C	-1.869431000	-2.732309000	0.723301000
C	-3.838371000	-3.987939000	0.042171000
H	-4.345222000	-4.622359000	-0.688549000
C	-2.531511000	-2.384799000	1.909101000
C	-4.476371000	-3.642309000	1.230581000
H	-2.030581000	-1.753239000	2.649741000
H	-5.487951000	-4.001188000	1.433151000
C	-3.815051000	-2.849779000	2.170391000
H	-4.306191000	-2.594249000	3.112091000
H	-2.023251000	-3.874929000	-1.110309000
Si	-0.036451000	-2.369120000	0.425081000
H	-1.563060000	0.041281000	1.349011000
H	0.688709000	-2.307170000	1.722391000
H	0.258919000	-3.640790000	-0.319179000
H	-1.290289000	5.942211000	-0.450599000
C	1.571471000	3.452560000	2.328461000
H	1.995831000	2.918300000	3.186781000
H	2.392421000	3.804590000	1.685711000
H	1.026691000	4.328340000	2.699501000
C	-3.715180000	2.418001000	-2.341649000
H	-4.442899000	3.045101000	-1.807739000
H	-3.246489000	3.030621000	-3.122849000
H	-4.247800000	1.588641000	-2.819619000
C	-4.169710000	-0.255789000	-0.417569000
H	-4.965840000	0.032382000	-1.119079000
H	-4.208121000	-1.344149000	-0.266149000
H	-4.329280000	0.240131000	0.549201000
C	0.543920000	0.468230000	3.450451000
H	-0.459480000	0.797130000	3.752171000
H	0.614330000	-0.620570000	3.590791000
H	1.300760000	0.948520000	4.085971000
C	2.552980000	0.536589000	1.407601000
H	3.173140000	1.117679000	2.104431000
H	2.757770000	-0.530421000	1.572101000
H	2.813470000	0.790309000	0.370811000
C	-2.471950000	-0.584919000	-2.703319000
H	-1.600050000	-0.201619000	-3.249809000
H	-2.370731000	-1.671439000	-2.566899000
H	-3.386410000	-0.398029000	-3.282689000

C	0.329889000	3.038540000	0.918749000
N	-0.850271000	2.573850000	0.530949000
C	5.713870000	-0.549539000	1.055129000
C	5.329390000	0.228921000	-0.036311000
C	5.167210000	-1.817619000	1.240789000
H	5.770739000	1.216291000	-0.187731000
H	5.479390000	-2.430509000	2.089089000
C	4.394320000	-0.262700000	-0.941721000
C	4.225660000	-2.303800000	0.337719000
H	4.108690000	0.353480000	-1.800881000
H	3.797290000	-3.299740000	0.493449000
C	3.822190000	-1.531830000	-0.761311000
H	6.454650000	-0.167239000	1.761309000
Si	2.439060000	-2.120690000	-1.841141000
H	2.604520000	-1.824770000	-3.283941000
H	2.093440000	-3.537870000	-1.581151000
O	1.019080000	-1.210660000	-1.369711000
C	0.781850000	0.110630000	-2.120561000
H	0.055610000	-0.201430000	-2.910711000
O	0.404409000	1.035810000	-1.293891000
C	-3.209780000	-2.848190000	-0.612791000
C	-2.155130000	-2.417640000	0.202429000
C	-4.440800000	-3.208440000	-0.065531000
H	-5.251150000	-3.541131000	-0.717831000
C	-2.355960000	-2.397880000	1.592149000
C	-4.625530000	-3.161031000	1.312529000
H	-1.537980000	-2.102380000	2.256089000
H	-5.586830000	-3.446271000	1.745709000
C	-3.574520000	-2.768660000	2.144489000
H	-3.708150000	-2.761910000	3.228339000
H	-3.063490000	-2.933670000	-1.694941000
Si	-0.453450000	-2.167440000	-0.583041000
H	-1.667380000	0.003650000	1.181779000
H	0.488410000	-2.844820000	0.347679000
H	-0.690270000	-2.814490000	-1.908351000
H	0.152949000	6.055570000	-0.207201000
C	-2.365871000	2.476000000	-1.306501000
H	-3.352401000	2.958890000	-1.417081000
H	-1.886001000	2.525430000	-2.296821000
C	1.263719000	2.003870000	1.463159000
H	2.046409000	1.900000000	0.693689000
H	1.763969000	2.340730000	2.388079000
N	-2.522391000	1.050310000	-0.915521000
N	0.615859000	0.687170000	1.665279000
C	-3.687371000	0.929280000	-0.016811000
H	-4.600531000	1.262009000	-0.541141000
H	-3.812340000	-0.115500000	0.290719000
H	-3.537511000	1.550950000	0.873939000
C	-2.798670000	0.288850000	-2.141461000
H	-1.928430000	0.317940000	-2.807211000
H	-3.040400000	-0.746130000	-1.882201000
H	-3.665561000	0.718880000	-2.673441000
C	-0.077121000	0.713990000	2.967619000
H	-0.856371000	1.484590000	2.970389000
H	-0.543340000	-0.257570000	3.161789000
H	0.648979000	0.928050000	3.771409000
C	1.669980000	-0.331540000	1.720979000
H	2.408990000	-0.080880000	2.502629000
H	1.230500000	-1.307790000	1.961789000
H	2.180880000	-0.391320000	0.756059000

TS7_Ni_L8

Ni	-0.812230000	0.498770000	0.143449000
H	1.760890000	0.303790000	-2.611061000
C	-1.450461000	3.264470000	-0.424081000
N	-1.133611000	4.512030000	-0.733231000
C	-0.082201000	4.994140000	-0.075461000
N	0.746109000	4.269970000	0.675289000

5. References

[1] *Gaussian 16, Revision A.03.*: Wallingford, CT, 2016.

[2] a) Y. Zhao, D. G. Truhlar, *Acc. Chem. Res.* **2008**, *41*, 157-167; b) Y. Zhao, D. G. Truhlar, *Theor. Chem. Acc.* **2008**, *120*, 215-241.

[3] a) F. Weigend, R. Ahlrichs, *Phys. Chem. Chem. Phys.* **2005**, *7*, 3297-3305; b) D. Rappoport, F. Furche, *J. Chem. Phys.* **2010**, *133*, 134105.

[4] A. V. Marenich, C. J. Cramer, D. G. Truhlar, *J. Phys. Chem. B* **2009**, *113*, 6378-6396.

[5] a) M. Busch, M. D. Wodrich, C. Corminboeuf, *Chem. Sci.* **2015**, *6*, 6754-6761; b) M. D. Wodrich, M. Busch, C. Corminboeuf, *Chem. Sci.* **2016**, *7*, 5723-5735; c) M. D. Wodrich, B. Sawatlon, E. Solel, S. Kozuch, C. Corminboeuf, *ACS Catal.* **2019**, *9*, 5716-5725.

[6] a) G. K. Rao, I. Korobkov, B. Gabidullin, D. Richeson, *Polyhedron* **2018**, *143*, 62-69; b) H. H. Cramer, S. Ye, F. Neese, C. Werle, W. Leitner, *JACS Au* **2021**, *1*, 2058-2069.

[7] R. Crabtree, *The organometallic chemistry of the transition metals*; John Wiley & Sons, Hoboken, New Jersey, **2005**.

[8] S. Bonnet, M. A. Siegler, J. H. van Lenthe, M. Lutz, A. L. Spek, G. van Koten, R. J. M. Klein Gebbink, *Eur. J. Inorg. Chem.* **2010**, *2010*, 4667-4677.

[9] a) M. Contel, M. Stol, M. A. Casado, G. P. M. van Klink, D. D. Ellis, A. L. Spek, G. van Koten, *Organometallics* **2002**, *21*, 4556-4559; b) J.-P. Cloutier, D. Zargarian, *Organometallics* **2018**, *37*, 1446-1455.

[10] M. L. Scheuermann, S. P. Semproni, I. Pappas, P. J. Chirik, *Inorg. Chem.* **2014**, *53*, 9463-9465.
